# Seasonal Expression of Prolactin Receptor in the Scented Gland of Male Muskrat (*Ondatra zibethicus*)

**DOI:** 10.1038/srep15036

**Published:** 2015-10-19

**Authors:** Han Cao, Liang Wang, Shuo Zhang, Lu Lu, Xia Sheng, Yingying Han, Zhengrong Yuan, Qiang Weng

**Affiliations:** 1 Laboratory of Animal Physiology, College of Biological Sciences and Technology, Beijing Forestry University, Beijing 100083, People’s Republic of China

## Abstract

Prolactin (PRL) has numerous actions in mammalian biological systems including mammary development and biological processes. The aim of this study was to investigate the seasonal changes of prolactin receptor (PRLR) expression in the scented gland of muskrat during the breeding and nonbreeding seasons. Histologically, glandular cells, interstitial cells and excretory tubules were identified in the scented glands in both seasons, whereas epithelial cells were sparse in the nonbreeding season. PRLR was observed in glandular cells of scented glands during the breeding and nonbreeding seasons with stronger immunostaining during the breeding season. Consistent with the immunohistochemical results, both the mean of protein and mRNA levels of PRLR were higher in the scented glands of the breeding season, and relatively lower level in the nonbreeding season. In addition, differential seasonal changes were also detected in the expression profile of microRNAs (miRNAs) in the scented gland of muskrat. Besides, plasma PRL concentration was remarkably higher in the breeding season than that in the nonbreeding season. These results suggested that muskrat scented gland was the direct target organ of PRL, and stronger expression of PRLR in scented glands during the breeding season indicated that PRL may directly regulate scented glandular function of the muskrats.

Prolactin (PRL) is mainly synthesized and secreted by the lactotrop cells of the pituitary^1^. Native PRL is secreted as a protein of approximately 23 kDa, cleavage of the full-length product by cathepsin D results in 16 kDa N-terminal PRL^2^. More than 300 separate actions have been reported in various vertebrates, including effects on water and salt balance, growth and development, endocrinology and metabolism, brain and behavior, reproduction, and immune regulation and protection^3^,^4^,^5^,^6^. Circulating PRL is also detected in males, although it is present at lower levels than in females. In males, PRL is known to influence reproductive functions but the significance and mechanisms of PRL action in male organs and tissues are poorly understood.

PRL mediates its physiologic functions through the engagement of prolactin receptor (PRLR), a member of the cytokine receptor superfamily. PRLR is a transmembrane protein comprising an extracellular domain, a transmembrane domain and an intracellular domain. Multiple isoforms of membrane-bound PRLR resulting from alternative splicing of the primary transcript have been identified^3^. These different PRLR isoforms (short and long) differ in the length and composition of their cytoplasmic tail. Most of them are similar in their extracellular domain, but differ in the intracellular part^7^. Thus, multiple isoforms potentially can activate distinct intracellular signaling events. Generally, the most abundant PRLR is the long isoform, whereas other intermediate and short forms also exist. The long receptor isoform was studied in detail, and specific functions of the other PRLR isoforms are relatively less investigated^6^. Upon ligand binding and sequential dimerisation, it activates multiple signaling systems including JAK2/STAT5, STAT3, MAPK p44/42 and PI3K pathways^3^,^7^,^8^,^9^,^10^. PRLR expression has been reported in a wide variety of cells and tissues. In male, experimental studies in animals and human suggest that PRL may via PRLR promote the function of the testis, prostate and reproductive accessory tissues^11^,^12^,^13^, and including the reproductive processes of species that breed seasonally such as golden hamsters, black bears (spring), sheep, rams and deer (autumn)^14^,^15^,^16^.

The muskrat (*Ondatra zibethicus*) is a medium-sized, semi-aquatic rodent that lives throughout North America, except in parts of the South where tidal fluctuation, periodic flooding, or drought limit their distribution^17^. The common name of the muskrat is derived from the conspicuous odor of secretions from paired perineal musk glands found beneath the skin at the ventral base of the tail^18^. The muskrat is a seasonal breeder with sexually active period of about 8 months from March to October. Increasing daylength of the breeding period is accompanied by increased release of pituitary gonadotrophins and marked testicular recrudescence, leading to enhanced testosterone production, spermatogenesis and pronounced testicular growth^19^. Additionally, to attract female muskrat during the breeding season, the males’ scented glands secrete musk (perfume substances), which is also a widely-used and costly ingredient in traditional Chinese medicine^20^. Our previous studies showed that as the target organ of androgens and estrogens, scented glands of the muskrats are capable of synthesizing androgens, estrogens as well as inhibins during the breeding season, which may play important autocrine or paracrine roles in mediating scented gland function^17^,^21^,^22^. Moreover, seasonal change of androgen receptor (AR) expression in scented glands during the breeding season and the nonbreeding season suggested that androgen may directly influence the glandular function^19^. Extensive studies have demonstrated that microRNAs (miRNAs) are important regulators of target gene expression at the post-transcriptional level, and are involved in a broad range of biological processes such as metabolism, tissue morphogenesis, development, differentiation, reproduction, and occurrence of several diseases^23^,^24^,^25^. Thus, miRNAs might also play an important role in regulating the development of scented gland of muskrat. However, up to date, the seasonal changes in the expression profile of miRNAs in the scented gland of muskrat during the breeding and nonbreeding seasons remain largely unknown. In this study, we investigated PRLR expression and localization as well as expression profile of miRNAs of the scented gland of muskrats during the breeding and nonbreeding seasons, to gain insight into the relationship of PRL and PRLR with regard to scented gland function of muskrats.

## Materials and Methods

### Animals

Twelve adult male muskrats were obtained in January (the nonbreeding season) and April (the breeding season) 2012 from Xichuan Wangnong Muskrat Breeding Farm, Beijing, China. All the animals were treated in accordance with the National Animal Welfare Legislation. All experimental procedures were approved by the Animal Ethic Committee at Experimental Center of Beijing Forestry University in accordance to the guidelines. Each pair of scented glands and testes was excised from the male muskrats after sacrifice. Weights and sizes of scented glands and testes were recorded after measured. One side of scented glands and testes were fixed immediately for 12 h in Bouin’s solution or 4% paraformaldehyde in 0.05 M phosphate buffered saline (PBS), pH 7.4 for histological and immunohistochemical observations; the other side of scented glands and testes were immediately stored at −80 °C for western blotting and reverse transcription-polymerase chain reaction (RT-PCR) detections. Blood samples were collected and centrifuged at 3000 g for 20 min to separate serum from blood cells, which were collected and stored at −20 °C for hormonal analysis.

### Histology

The scented glandular and testicular samples were dehydrated in ethanol series and embedded in paraffin wax. Serial sections (4 μm) were mounted on slides coated with poly-L-lysine. Some sections were stained with hematoxylin-eosin (HE) for observations of general histology. The rest of the sections were processed for immunohistochemistry.

### Immunohistochemistry

The serial sections of tests and scented glands were incubated with 10% normal goat serum to reduce background staining caused by the second antibody. The sections were then incubated with primary antibodies (1:1000) raised against PRLR (H-300) (Santa Cruz Biotechnology, Santa Cruz, CA, USA) for 12 h under 4 °C. The sections were then incubated with a secondary antibody, goat anti-rabbit lgG conjugated with biotin and peroxidase with avidin, using a rabbit ExtrAvidin staining kit (Sigma, MO, USA), followed by visualizing with 30 mg 3,3-diaminobenzidine (Wako, Tokyo, Japan) solution in 150 ml of 0.05 mol Tris–HCl l^−1^ buffer, pH 7.6, plus 30 μl H_2_O_2_. To value the specificity of the polyclonal antibodies, PRLR antibody was performed in the mammary of muskrat, which are known to express the protein. Sections treated with pre-absorped primary antibodies were used as negative controls. The control sections were also treated with normal rabbit serum instead of the primary antisera.

### Western blotting

Scented glandular tissue was divided into small pieces with a clean razor blade. The tissue was homogenized in a homogenizer containing 300 μl of 10 mg/ml PMSF stock and incubated on ice for 30 min to maintain the temperature at 4 °C throughout all the procedures. Following centrifugation at 12000 g for 10 min at 4 °C, the supernatant was collected. Protein extracts (25 μg) were mixed with an equal volume of 2× Laemmli sample buffer. Equal amounts of each sample were loaded and run on a 12% sodium dodecyl sulfate-polyacrylamide gel electrophoresis (SDS–PAGE) gel at 18 V/cm and transferred to nitrocellulose membranes using a wet transblotting apparatus (Bio-Rad, Richmond, CA, USA). Membranes were blocked with 3% albumin from bovine serum (BSA) for 1 h at room temperature. The membranes were washed and incubated with a 1:1000 PRLR primary antibody for 1 h. Secondary incubation of the membrane was then carried out using a 1:1000 dilution of goat anti-rabbit IgG tagged with horseradish peroxidase for 60 min. Finally, the membrane was colored with 10 mg 3,3-diaminobenzidine solution in 50 ml phosphate buffer (0.03 M) plus 3 μl H_2_O_2_. Antibodies pre-absorptions were also performed here as a negative control.

### RT-PCR

The first-strand cDNA from total RNA (six samples for each season) was synthesized using StarScript II Reverse Transcriptase and Oligo (dT)18 by TIANScript RT Kit (Tiangen, Beijing, China). The 20 μl of reaction mixture contained 3 μg of total RNA, 1 μl of Oligo (dT)18, 1 μl of 10 mM deoxy-ribonucleoside triphosphate (dNTP), 4 μl of 250 Mm Tris–HCl (pH 8.3), 375 mM KCl and 15 mM MgCl_2_, 2 μl of 0.1 M dithiothreitol, 0.5 μl of RNase Inhibitor and 200 U of StarScript II enzyme. The 25 μl of reaction mixture contained 2 μl of first-strand cDNA, 0.5 μM each primer, 1.5 mM MgCl_2_, 0.2 mM dNTP, 20 mM Tris–HCl (pH 8.4) and 2.5 U of Taq polymerase (Tiangen, Beijing, China). The amplification was under the following condition: 94 °C for 3 min for the initial denaturation of the RNA/ cDNA hybrid, 35 cycles of 94 °C for 30 s, 51 °C for 30 s and 72 °C for 1 min with a final extension of 10 min at 72 °C. The *PRLR* cDNA fragment was amplified by primers 5′-CGCTCTCCTGACAAGGAAAC -3′ and 5′-GGGACCATTTTACCCACAGA -3′. The primer set was intron spanning. The PCR product was electrophoresed in the 1% agarose gel and individual bands were visualized by ethidium bromide (EB) staining. Breast tissues of muskrat were used as positive control and water, instead of cDNA, was used as negative control. The housekeeping gene, Actb (the gene which encodes β-actin), was selected as the endogenous control. The bands were quantified using Quantity One software (Version 4.5, Bio-Rad Laboratories) and expression ratios were calculated.

### MicroRNAs-sequencing and bioinformatic analysis

The small RNA (sRNA) libraries for the scented gland of muskrat from breeding season (named: SGB1) and nonbreeding season (named: SGNB2) were constructed from total RNAs using the Illumina Truseq Small RNA Preparation kit (RS-930–1012, Illumina Inc., USA), and were sequenced on the Illumina GAIIx platform following the vendor’s recommended protocol at Beijing Yuanquanyike Biological Technology Co., Ltd (Beijing, China). A proprietary pipeline script, ACGT101-miR v4.2 (LC Sciences, Houston, TX, USA), was utilized to analyze the sequencing data. The sRNAs were annotated by comparison with the deposited sequences in the NCBI GenBank (http://www.ncbi.nlm.nih.gov/) and the Rfam11.0 databases (http://rfam.sanger.ac.uk/). The remaining sequences were used to BLAST search against miRBase (version 20, http://www.mirbase.org/) to identify known miRNAs. Potential novel miRNAs candidates were predicted by Mireap (version 0.2, http://sourceforge.net/projects/mireap/). Potential target genes regulated by miRNAs were predicted using the miRanda (version 3.3a, http://www.microrna.org/microrna/). R software was utilized to analyze the correlation between differential expression profile of miRNAs and their targeted genes. The biological functions of miRNA-targeted gene candidates were revealed by Gene Ontology enrichment (GO, http://www.geneontology.org/) and Kyoto Encyclopedia of Genes and Genomes (KEGG, http://www.genome.jp/kegg/) analyses.

### Hormone Assays

The plasma samples from each animal were analyzed by the enzyme linked immunosorbent assay (ELISA) to detect the plasma PRL concentrations using the commercial Prolactin Rat ELISA Kit (CSB-E06881r).

### Statistical analysis

Statistical comparisons were made with the Students t-test and One-way analysis of variance. A value of p < 0.05 was considered indication of statistical significance.

## Results

### Histology and Immunohistochemistry

**Figure 1:**
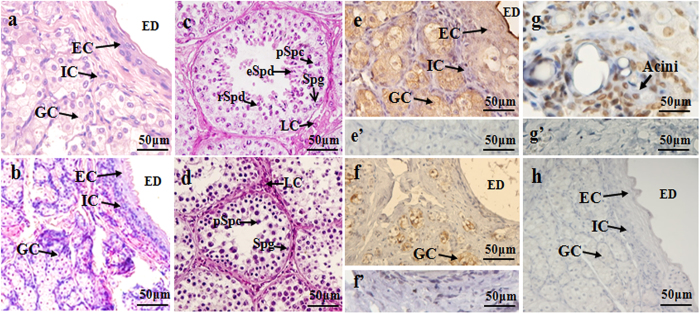
Histological structure of the muskrat scented glands and testes, and immunolocalization for PRLR in scented glands of the muskrat. (**a**) Hematoxylin-eosin (HE) staining of scented gland during the breeding season. (**b**) HE staining of scented gland during the nonbreeding season. (**c**) HE staining of testis during the nonbreeding season. (**d**) HE staining of testis during the breeding season. (**e**) Immunolocalization for PRLR in scented glands during the breeding season. (**f**) Immunolocalization for PRLR in scented glands during the nonbreeding season. (**g**) PRLR detected in the mammary of muskrat was used as a positive control. (**e’–f’**) No signal was detected in negative control sections in which the primary antibody was pre-absorbed by a respective antigen. (**h**) Negative control sections were also treated with normal rabbit serum instead of primary antisera. GC, glandular cells; EC, epithelial cells; IC, interstitial cells; ED, excretory ducts; LC, Leydig cell; Spg, spermatogonium; pSpc, primary spermatocyte; rSpd, round spermatid; eSpd, elongate spermatid. Scale bars represent 50 μm.

Glandular cells, interstitial cells and epithelial cells of the excretory duct were observed in the scented glands of male muskrats both during the breeding and nonbreeding seasons (Fig. 1a,b), which was in accordance with our previous studies^19^,^21^. Glandular cells which secreted musk were the main cell type in the scented glands of male muskrat. During the nonbreeding season, epithelial cells were sparser and interstitial cells among glandular cells turned thicker than the breeding season. HE staining also revealed that all types of germ cells were shown in the breeding season in muskrat testes, while only spermatogonia and primary spermatocytes could be identified in the nonbreeding season (Fig. 1c,d). Immunolocalization for PRLR was detected in scented glands of muskrat in the breeding and nonbreeding seasons (Fig. 1e,f). Immunoreactivity for PRLR was present in glandular and epithelial cells during the breeding season (Fig. 1e), while only in glandular cells in the nonbreeding season (Fig. 1f). For positive control, PRLR was expressed in breast cells of the muskrat (Fig. 1g). The specificity of the antibody was confirmed, as a substantial decrease in immunostaining intensity was observed after pre-absorption with the specific recombinant proteins (Fig. 1e’), which is comparable to the negative control treated with normal rabbit serum instead of primary antisera (Fig. 1h).

### Western Boltting and RT-PCR

**Figure 2:**
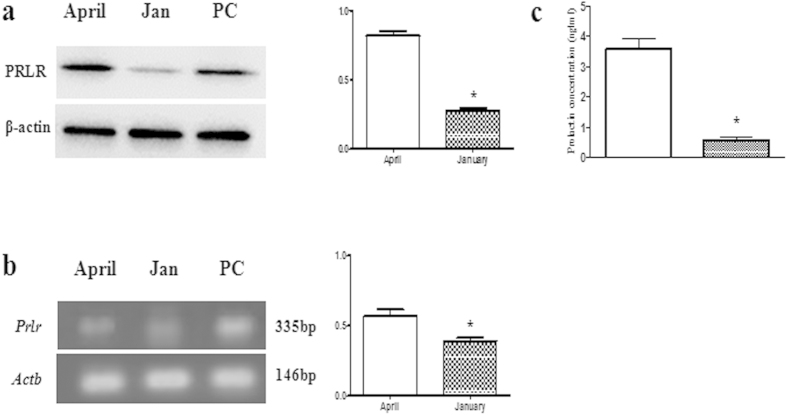
Protein and mRNA expression of PRLR in the scented gland and plasma level profile of prolactin. The seasonal profiles of protein (**a**) and mRNA (**b**) expression of PRLR in the scented glands of the muskrat. Each bar represents the means ± standard deviation of 6 muskrats per group. Means within the two columns are significantly different from each other (p < 0.05). (**c**) The seasonal profiles of serum prolactin concentration. Bars represent means ± standard deviation for independent experiments. Asterisks denote statistically significant values (p < 0.01).

The protein level of PRLR in scented gland of muskrats during the breeding and nonbreeding seasons was shown in Fig. 2a. A PRLR-positive band of 75 kDa was identified in protein extracted from scented gland during the breeding and nonbreeding seasons. The intensity of PRLR in the breeding season was significantly higher than the nonbreeding season. Protein extracted from breast of the breeding season in the muskrat and the primary antibody pre-absorbed with an excessive amount of the antigen was used as positive control (PC) and negative control (NC), respectively (Fig. 2a, land PC). Meanwhile, the expression of mRNA of PRLR during the breeding and nonbreeding seasons was shown in Fig. 2b, which was generally in line with protein expression level. The mRNA extracted from the breast in the breeding season was used as a positive control (PC) (Fig. 2b, land PC). The results were normalized to β-actin (Actb), and primers specific for PRLR were used to amplify a RT-PCR product of 335 bp. Densitometric analysis revealed a significant increase in mRNA level during the breeding season as compared to the nonbreeding season. Compared with rat, mouse, bovine and human sequences, the PRLR cDNA nucleotide sequence identity was 86.04%, 84.33%, 81.25%, 74.93%.

### Plasma level profile of PRL

The concentrations of prolactin in the plasma of muskrats during the breeding and nonbreeding seasons were measured by ELISA. There was a significant decrease of PRL concentration from the breeding season (3.624 ± 0.322 ng/ml in April, p > 0.05) to the nonbreeding season (0.564 ± 0.106 ng/ml in January, p < 0.01) (Fig. 2c).

### Seasonal changes in expression profile of microRNAs

**Figure 3:**
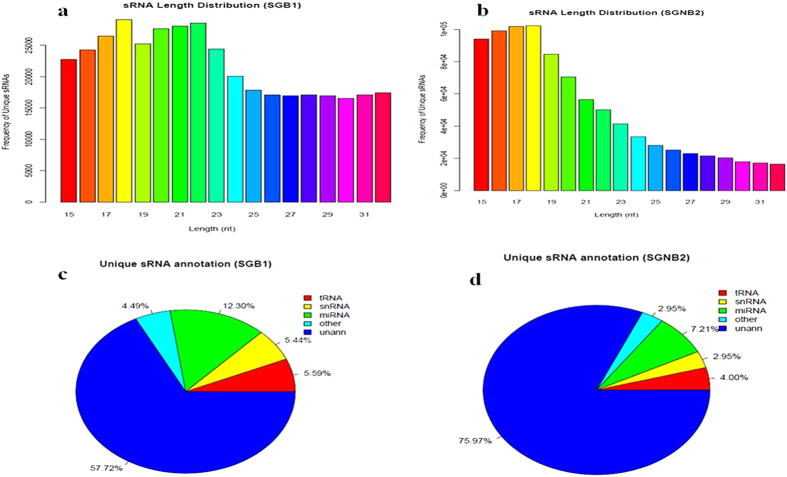
Sequence length distribution and annotation of unique small RNA (sRNA). (**a**) Sequence length distribution of unique sRNA for SGB1; (**b**) Sequence length distribution of unique sRNA for SGNB2; (**c**) Annotation of unique sRNA for SGB1; (**d**) Annotation of unique sRNA for SGNB2.

In total, 56,843,144 raw reads (for SGB1: 28,127,272 raw reads; for SGNB2: 28,715,872 raw reads) were obtained by high-throughput sequencing using Illumina GAIIx platform, and 32,880,214 clean reads (for SGB1: 15,217,382 clean reads, 54.10%; for SGNB2: 17,662,832 clean reads, 61.50%) remained for the further sRNAs digitalization analyses. As shown in Fig. 3a,b, sRNAs were distributed in the 15–32 nt length. All of the clean reads are summarized as the following categories: sum, anno (annotated), ribosomal RNA (rRNA), transfer RNA (tRNA), small nuclear RNA (snRNA), miRNA, other, unann (unannotated) (Table 1, additional file 1: Table S1; Fig. 3c,d). As for SGB1, the miRNAs have 5,270,234 total reads and 48,428 unique reads, representing 45.65% of total reads and 12.30% of unique clean reads. As for SGNB2, the miRNAs have 7,178,426 total reads and 65,132 unique reads, representing 55.48% of total reads and 7.21% of unique clean reads. Our data indicated that miRNA expression is abundant in the scented gland of muskrat, as a total of 2,464 known miRNAs and 161 predicted novel miRNAs were detected in the two stages. MiRNAs-sequencing in the scented gland of muskrat collected from the breeding and nonbreeding seasons revealed seasonal changes in the expression profile of miRNAs. Of the sixteen differentially expressed miRNAs, five miRNAs was significantly down-regulated during the breeding season compared to the nonbreeding season, while expression of eleven miRNAs was significantly up-regulated (Table 2).

### Bioinformatic prediction for regulation of biological functions by differential microRNAs expression

GO enrichment and KEGG pathway analyses were performed to investigate the regulation of biological functions by these differentially expressed miRNAs. Some miRNA-targeted gene candidates of these differential expressed miRNAs were enriched in related circadian rhythm and reproduction pathways, such as melanogenesis, MAPK, Notch, NF-kappa B, Wnt, Calcium signaling pathways (Additional file 2, Table S2; Additional file 3, Fig. S1; Additional file 4, Fig. S2). For instance, mmu-miR-1b-5p shows differential seasonal expressions in the scented gland of muskrat, and its target gene candidates were predominantly enriched in circadian rhythm, retinol metabolism and melanogenesis (Additional file 5, Table S3). The miRNA-target gene prediction indicated that the PRL and PRLR genes might be regulated by the differential seasonal expressions of miRNAs, such as mmu-miR-1b–5p, mmu-miR-5119, rno-miR-144–5p (Additional file 6, Table S4).

## Discussion

The present study was the first to investigate the expression and distribution patterns of PRLR in male muskrat scented glands during the breeding and nonbreeding season. Our results demonstrated that immunoreactivities of PRLR was higher in muskrat scented glands of the breeding season when compared to that in the nonbreeding season. The differential expression of the scented gland of muskrat between the breeding and nonbreeding seasons might be regulated by miRNAs, which are significant post-transcriptional regulators of miRNAs-target genes, such as PRL and PRLR gene. In addition, the expression patterns of PRLR in musk scented glands were correlated with the changes of plasma concentrations of PRL and the histological results. These findings suggest that PRLR may be involved in the regulation of seasonal changes in the scented glandular function of muskrat.

PRL acts at several levels of male reproduction in many mammals, playing a role in steroidogenesis and gametogenesis in the testis and influencing the reproductive tract, sexual behavior and seasonal breeding^15^. The release of PRL depends on the photoperiod, with long days being stimulatory and short days inhibitory. Seasonal changes for plasma PRL levels in the male muskrats were first observed in this study, and it showed that plasma PRL concentrations were higher in the breeding season and lower in the nonbreeding season, which is not unique to the male muskrats. Other seasonal breeders, such as golden hamsters, Suffolk rams, black bears and polar bears, showed similar seasonal changes in circulating PRL concentration in accordance with the annual reproductive cycles. In male golden hamster, the increasing serum PRL concentration were observed during longer days, PRL acts at multiple levels of the hypothalamic-pituitary-testicular axis, and both directly and indirectly regulate seasonal transitions between periods of full testicular activity and testicular quiescence^15^,^26^. In the young adult Suffolk rams, the spring increase in prolactin secretion could target both the testes and the hypothalamic-pituitary system and were involved in the seasonal regulation of sexual function^27^. In the black bear, serum PRL levels were lowest in the nonbreeding season (December), and increased gradually from January until May, coincided with testicular recrudescence and the onset of the breeding season and preceded peak testosterone concentrations, which were achieved in the breeding season^28^,^29^. Similar study in polar bears also showed that highest levels of PRL were measured in the breeding season, and testes reached maximal size in this period^30^. In our previous study, scented glandular recrudescence and regression of the muskrats synchronized with testicular recrudescence and regression^19^, both of which corresponded with the change of PRL level, as revealed by the present study. These findings implied that the changes of scented glandular function of muskrats might be involved in role of PRL, which speculated that PRL might play a regulatory role in the scented gland function of muskrats.

PRLR expression has been reported in a wide variety of cells and tissues. In male, PRLR was expressed in the testes and in various male accessory glands include vas deferens, epididymis, prostates and seminal vesicles^11^,^13^,^31^, which demonstrated that these organs might be direct targets of PRL, suggesting multiple regulatory roles for PRL in male reproductive tract. The present study demonstrated for the first time the expression of the PRLR gene and protein in the scented glands of the male muskrats during the breeding and nonbreeding seasons. PRLR was shown to possess a cytoplasmic localization in glandular tissues in each examined period, and its cellular distribution was found to be restricted to glandular cells. These findings suggested that PRL may be required for not only growth and differentiation of glandular tissues, but also the secretory process of the mature gland.

Seasonal changes in testicular function are not only involved changes in circulating PRL but also involved seasonal changes in testicular PRLR^32^. Previous studies have demonstrated the testicular PRLR expression increased during testicular recrudescence among several species with seasonal changes in reproduction^28^,^32^,^33^,^34^. In the hamster, testicular abundance of PRLR mRNA increased in the breed season, coincident with a significant increase in serum PRL concentrations^33^. This finding is consistent with other studies that demonstrated a positive relationship between PRLR mRNA and serum PRL concentrations, and it suggested that PRL up-regulates its own receptor in the breeding season^28^,^35^,^36^. In addition, the histological results of scented glands also showed that epithelial cells, interstitial cells and a large number of glandular cells were observed in scented glands during the breeding season. These findings suggested that the rise in PRLR expression level in the breeding season might be an adaptation to intensified PRLR action. The multifunction of PRL may range from growth stimulation to the initiation and maintenance of musk synthesis. In this study, for the first time, we profiled the expression of miRNAs in the scented gland of muskrats during the breeding and nonbreeding seasons, which showed significant seasonal differences. This suggests that miRNAs are regulated throughout the seasonal cycle of the secretory musk gland, and lay the foundation for further functional analysis of candidate miRNAs in this particular process. As seasonal variations in miRNA expression correlates to changes in PRL level, it would also be of interest to dissect the functions of miRNAs with regard to PRL regulation. The present study demonstrated the seasonal pattern of scented glandular PRLR profile regulated by miRNAs, mRNA and protein expression is correlated with PRL levels and mass of scented glands, which reached high levels in the breeding period with lower levels in the non-breeding period. PRL signaling pathway displays significant crosstalk with the AR signaling pathway in prostate function^13^. PRL could induce expression of AR, and enhanced androgen action^13^,^37^,^38^. Our recent studies demonstrated that steroidogenic enzymes were immunolocalized in muskrat testes and scented glands during the breeding season, muskrat scented glands were capable of locally synthesizing androgen^21^. Moreover, muskrat scented glands was identified to be the direct target organ of androgen, and stronger expression of AR in scented glands and higher levels of testosterone (T) were observed in the breeding season, which suggested that androgens may directly influence scented glandular function of the muskrats^19^. Indeed, previous studies have demonstrated a positive relationship between PRL and T production^12^,^30^,^39^,^40^. PRL could stimulate steroidogenesis not only by up-regulating luteinizing hormone receptor (LHR) but also by increasing stores of esterfied cholesterol and increasing 3beta-Hydroxysteroid dehydrogenase (3β-HSD) and 17β-HSD activity in the mouse^41^,^42^,^43^. Taking together, our results suggest that the cooperative action of PRL and androgen may be involved in the regulation of seasonal changes in the scented glands of muskrats. As evidence in breast and prostate tumors showed that tumors are capable of local secretion of PRL, which in turn favors the tumor development in an autocrine/paracrine mechanism^44^, future studies will investigate whether the scented gland of the muskrat synthesizes PRL.

In conclusion, the present study provided new evidence supporting that muskrat scented gland is a target organ of PRL. High PRL level and strong expression of PRLR in the scented gland during the breeding season may be associated with the expression of specific miRNAs. The data presented here links PRL to the musk gland, a typical exocrine gland, thus represent a valuable addition to the current awareness of the PRL function.

## Additional Information

**How to cite this article**: Cao, H. *et al.* Seasonal Expression of Prolactin Receptor in the Scented Gland of Male Muskrat (*Ondatra zibethicus*). *Sci. Rep.* **5**, 15036; doi: 10.1038/srep15036 (2015).

## Supplementary Material

Supplementary Information

## Figures and Tables

**Table 1:** Annotation of total small RNAs (sRNAs).

Samples	SGB1	%	SGNB2	%
Sum	11,545,606	100.00	12,938,262	100.00
anno	10,495,734	90.91	11,025,300	85.21
rRNA	476,070	4.12	574,536	4.44
tRNA	4,534,718	39.28	2,986,060	23.08
snRNA	120,728	1.05	157,446	1.22
miRNA	5,270,234	45.65	7,178,426	55.48
other	93,984	0.81	128,832	1.00
unann	1,049,872	9.09	1,912,962	14.79

Note: sRNAs, small RNAs; anno, annotated; rRNA, ribosomal RNA; tRNA, transfer RNA; snRNA, small nuclear RNA, miRNA, microRNAs; unann, unannotated.

**Table 2:** Differential expressions of microRNAs (miRNAs) in the scented gland of muskrat collected from the breeding and nonbreeding season.

miRNAs	Fold Change	p-value
mmu-miR-8112	−4.6293	0.0222
hsa-miR-4454	−3.8752	0.0072
mmu-miR-6937–5p	−2.8517	0.0196
hsa-miR-5100	−2.7549	0.0441
rno-miR-147	−2.7097	0.0386
mmu-miR-126b–5p	2.4633	0.0341
hsa-miR-1	2.9242	0.0170
mmu-miR-7058–3p	2.9386	0.0334
hsa-miR-202–5p	3.0086	0.0324
hsa-miR-4485	3.0683	0.0147
rno-miR-144–5p	3.0834	0.0229
mmu-miR-1b–5p	3.0912	0.0139
rno-miR-451–5p	3.0991	0.0127
hsa-miR-762	3.4448	0.0215
rno-miR-144–3p	3.4485	0.0096
mmu-miR-5119	3.7935	0.0202
