# Supplementary material for: Seasonal Expression of Prolactin Receptor in the Scented Gland of Male Muskrat (Ondatra zibethicus)
Source: Sci Rep. 2015 Oct 19;5:15036. doi: 10.1038/srep15036 (PMC4609948; doi:10.1038/srep15036)
Supplement: Supplementary Information [file srep15036-s1.pdf]

Seasonal Expression of Prolactin Receptor in the Scented Gland of Male Muskrat (*Ondatra zibethicus*)

Han Cao, Liang Wang, Shuo Zhang, Lu Lu, Xia Sheng, Yingying Han, Zhengrong Yuan, Qiang Weng

Additional files

Additional file 1: Table S1. Annotation of unique small RNAs (sRNAs).

Additional file 2: Table S2. KEGG pathways for the miRNA-targeted genes of differential expressed miRNAs.

Additional file 3: Fig. S1. Circadian rhythm pathway in which miRNA-targeted genes of differential expressed miRNAs enriched.

Additional file 4: Fig. S2. MAPK signaling pathway in which miRNA-targeted genes of differential expressed miRNAs enriched.

Additional file 5: Table S3. KEGG pathways for the miRNA-targeted genes of mmu-miR-1b-5p.

Additional file 6: Table S4. The miRNA-targeted genes of mmu-miR-1b-5p, mmu-miR-5119, rno-miR-144-5p.

Seasonal Expression of Prolactin Receptor in the Scented Gland of Male Muskrat (*Ondatra zibethicus*)

Han Cao, Liang Wang, Shuo Zhang, Lu Lu, Xia Sheng, Yingying Han, Zhengrong Yuan, Qiang Weng

Additional file 1:

Table S1. Annotation of unique small RNAs (sRNAs).

Samples	**SGB1**	**%**	**SGNB2**	**%**
Sum	393,564	100.00	902,832	100.00
anno	166,412	42.28	216,954	24.03
rRNA	56,930	14.47	62,430	6.91
tRNA	21,990	5.59	36,084	4.00
snRNA	21,400	5.44	26,652	2.95
miRNA	48,428	12.30	65,132	7.21
other	17,664	4.49	26,656	2.95
unann	227,152	57.72	685,878	75.97

Note: sRNAs, small RNAs; anno, annotated; rRNA, ribosomal RNA; tRNA, transfer RNA; snRNA, small nuclear RNA, miRNA, microRNAs**;** unan, unannotated.

Seasonal Expression of Prolactin Receptor in the Scented Gland of Male Muskrat (*Ondatra zibethicus*)

Han Cao, Liang Wang, Shuo Zhang, Lu Lu, Xia Sheng, Yingying Han, Zhengrong Yuan, Qiang Weng

Additional file 2: Table S2. KEGG pathways for the miRNA-targeted genes of differential expressed miRNAs.

SGB1 vs. SGNB2_down

Pathway	Name	Class	Over represented p-value	Adjust_pvalue
ko04144	Endocytosis	Transport and catabolism	0	0
ko05200	Pathways in cancer	Cancers	0	0
ko04512	ECM-receptor interaction	Signaling molecules and interaction	0	0
ko04510	Focal adhesion	Cell communication	0	0
ko05166	HTLV-I infection	Infectious diseases	0	0
ko04010	MAPK signaling pathway	Signal transduction	0	0
ko04540	Gap junction	Cell communication	0	0
ko05416	Viral myocarditis	Cardiovascular diseases	0	0.001
ko04910	Insulin signaling pathway	Endocrine system	0	0.001
ko00330	Arginine and proline metabolism	Amino acid metabolism	0	0.002
ko04150	mTOR signaling pathway	Signal transduction	0	0.002
ko04920	Adipocytokine signaling pathway	Endocrine system	0	0.002
ko05220	Chronic myeloid leukemia	Cancers	0	0.002
ko05221	Acute myeloid leukemia	Cancers	0	0.002
ko04730	Long-term depression	Nervous system	0	0.002
ko04916	Melanogenesis	Endocrine system	0	0.003
ko05414	Dilated cardiomyopathy (DCM)	Cardiovascular diseases	0	0.004

ko04514	Cell adhesion molecules (CAMs)	Signaling molecules and interaction	0	0.004
ko05145	Toxoplasmosis	Infectious diseases	0	0.004
ko04012	ErbB signaling pathway	Signal transduction	0	0.005
ko04310	Wnt signaling pathway	Signal transduction	0	0.005
ko05412	Arrhythmogenic right ventricular cardiomyopathy (ARVC)	Cardiovascular diseases	0	0.006
ko04666	Fc gamma R-mediated phagocytosis	Immune system	0.001	0.007
ko04810	Regulation of actin cytoskeleton	Cell motility	0.001	0.008
ko04360	Axon guidance	Development	0.001	0.011
ko05218	Melanoma	Cancers	0.001	0.011
ko05215	Prostate cancer	Cancers	0.001	0.011
ko04660	T cell receptor signaling pathway	Immune system	0.001	0.012
ko05211	Renal cell carcinoma	Cancers	0.001	0.012
ko04070	Phosphatidylinositol signaling system	Signal transduction	0.001	0.014
ko04340	Hedgehog signaling pathway	Signal transduction	0.002	0.018
ko05212	Pancreatic cancer	Cancers	0.002	0.02
ko04962	Vasopressin-regulated water reabsorption	Excretory system	0.002	0.022
ko04064	NF-kappa B signaling pathway	Signal transduction	0.003	0.027
ko04940	Type I diabetes mellitus	Endocrine and metabolic diseases	0.003	0.027
ko05410	Hypertrophic cardiomyopathy (HCM)	Cardiovascular diseases	0.003	0.027
ko05216	Thyroid cancer	Cancers	0.004	0.029
ko04724	Glutamatergic synapse	Nervous system	0.004	0.029
ko00604	Glycosphingolipid biosynthesis - ganglio series	Glycan biosynthesis and metabolism	0.004	0.032
ko05169	Epstein-Barr virus infection	Infectious diseases	0.004	0.032

ko04930	Type II diabetes mellitus	Endocrine and metabolic diseases	0.005	0.033
ko05161	Hepatitis B	Infectious diseases	0.004	0.033
ko04720	Long-term potentiation	Nervous system	0.005	0.034
ko04330	Notch signaling pathway	Signal transduction	0.005	0.035
ko00562	Inositol phosphate metabolism	Carbohydrate metabolism	0.006	0.038
ko04141	Protein processing in endoplasmic reticulum	Folding, sorting and degradation	0.006	0.038
ko04145	Phagosome	Transport and catabolism	0.006	0.038
ko05217	Basal cell carcinoma	Cancers	0.006	0.041
ko04380	Osteoclast differentiation	Development	0.007	0.043

SGB1 vs. SGNB2_up

Pathway	Name	Class	Over represented p-value	Adjust p-value
ko05200	Pathways in cancer	Cancers	0	0
ko04360	Axon guidance	Development	0	0
ko04510	Focal adhesion	Cell communication	0	0
ko04010	MAPK signaling pathway	Signal transduction	0	0
ko04310	Wnt signaling pathway	Signal transduction	0	0
ko05030	Cocaine addiction	Substance dependence	0	0.003
ko04512	ECM-receptor interaction	Signaling molecules and interaction	0	0.004
ko05166	HTLV-I infection	Infectious diseases	0	0.011
ko04662	B cell receptor signaling pathway	Immune system	0	0.013
ko04070	Phosphatidylinositol signaling system	Signal transduction	0.001	0.016
ko05146	Amoebiasis	Infectious diseases	0.001	0.016
ko05217	Basal cell carcinoma	Cancers	0.001	0.017
ko05031	Amphetamine addiction	Substance dependence	0.001	0.025

ko00562	Inositol phosphate metabolism	Carbohydrate metabolism	0.001	0.028
ko04380	Osteoclast differentiation	Development	0.001	0.028
ko05221	Acute myeloid leukemia	Cancers	0.002	0.028
ko05222	Small cell lung cancer	Cancers	0.001	0.028
ko04012	ErbB signaling pathway	Signal transduction	0.002	0.03
ko04520	Adherens junction	Cell communication	0.002	0.03
ko04722	Neurotrophin signaling pathway	Nervous system	0.002	0.03
ko05211	Renal cell carcinoma	Cancers	0.002	0.03
ko04020	Calcium signaling pathway	Signal transduction	0.002	0.03
ko04064	NF-kappa B signaling pathway	Signal transduction	0.002	0.03
ko05220	Chronic myeloid leukemia	Cancers	0.002	0.03
ko04120	Ubiquitin mediated proteolysis	Folding, sorting and degradation	0.003	0.041

Seasonal Expression of Prolactin Receptor in the Scented Gland of Male Muskrat (*Ondatra zibethicus*)

Han Cao, Liang Wang, Shuo Zhang, Lu Lu, Xia Sheng, Yingying Han, Zhengrong Yuan, Qiang Weng

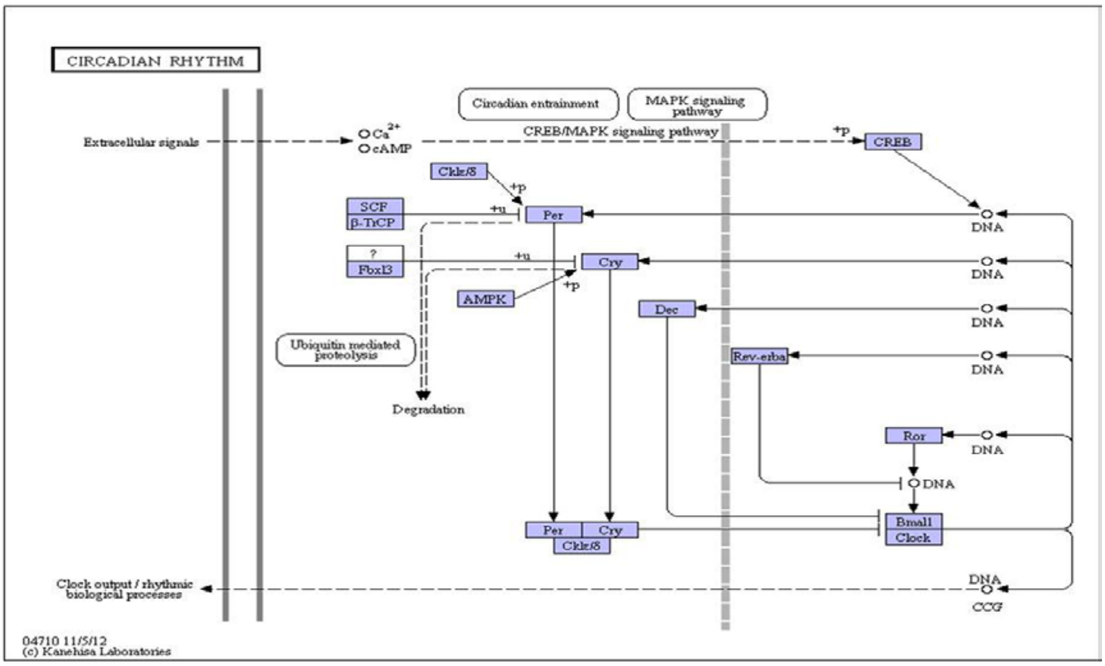

Additional file 3: Fig. S1. Circadian rhythm pathway in which miRNA-targeted genes of differential expressed miRNAs enriched.

Seasonal Expression of Prolactin Receptor in the Scented Gland of Male Muskrat (*Ondatra zibethicus*)

Han Cao, Liang Wang, Shuo Zhang, Lu Lu, Xia Sheng, Yingying Han, Zhengrong Yuan, Qiang Weng

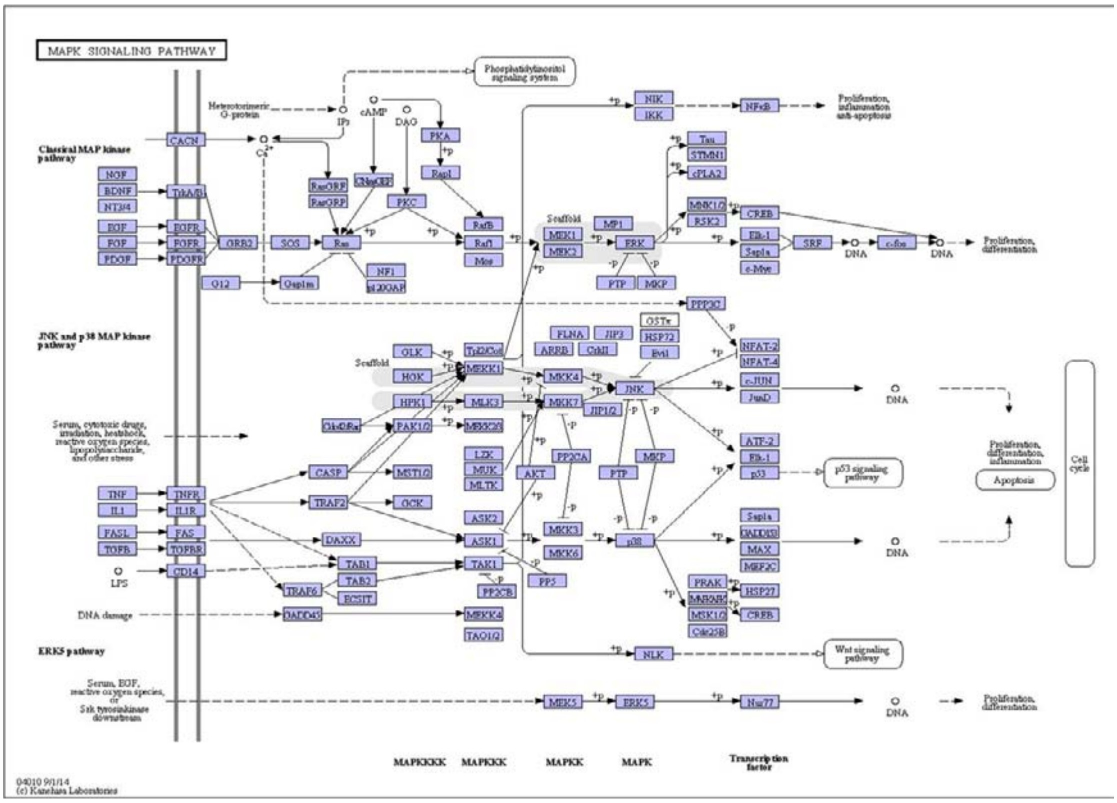

Additional file 4: Fig. S2. MAPK signaling pathway in which miRNA-targeted genes of differential expressed miRNAs enriched.

Seasonal Expression of Prolactin Receptor in the Scented Gland of Male Muskrat (*Ondatra zibethicus*)

Han Cao, Liang Wang, Shuo Zhang, Lu Lu, Xia Sheng, Yingying Han, Zhengrong Yuan, Qiang Weng

Additional file 5: Table S3. KEGG pathways for the miRNA-targeted genes of mmu-miR-1b-5p.

Pathway	Name	Class	Over represented p-value	Adjust p-value
ko04910	Insulin signaling pathway	Endocrine system	0	0.003
ko04142	Lysosome	Transport and catabolism	0	0.008
ko04520	Adherens junction	Cell communication	0	0.008
ko04710	Circadian rhythm	Environmental adaptation	0	0.008
ko05213	Endometrial cancer	Cancers	0	0.023
ko00830	Retinol metabolism	Metabolism of cofactors and vitamins	0	0.025
ko05215	Prostate cancer	Cancers	0.001	0.028
ko05218	Melanoma	Cancers	0.001	0.028
ko04916	Melanogenesis	Endocrine system	0.001	0.032

Seasonal Expression of Prolactin Receptor in the Scented Gland of Male Muskrat(*Ondatra zibethicus*)

Han Cao, Liang Wang, Shuo Zhang, Lu Lu, Xia Sheng, Yingying Han, Zhengrong Yuan, Qiang Weng

Additional file 6: Table S4. The miRNA-targeted genes of mmu-miR-1b-5p, mmu-miR-5119, rno-miR-144-5p.

mmu-miR-1b-5p			mmu-miR-5119			rno-miR-144-		
mRNA	Gene	Length	mRNA	Gene	Length	mRNA	Gene	Length
NM 011683	Vmn1r51	2359	NM 001177545	Zfp600	4155	NM 030739	Vmn1r58	2683
NM 030735	Vmn1r172	1234	NM 053151	Klra21	1150	NM 030738	Vmn1r65	2685
NM 001025615	Ccdc50	6972	NM 001167161	Gm10670	966	NM 001025615	Ccdc50	6972
NM 001289436	Ccdc50	7063	NM 011683	Vmn1r51	2359	NM 001289436	Ccdc50	7063
NM 026202	Ccdc50	7017	NM 011684	Vmn1r45	3249	NM 026202	Ccdc50	7017
NM_009016	Raet1a	1546	NM_001025615	Ccdc50	6972	NM_001167918	D830031N03Rik	8311
NM_001167918	D830031N03Rik	8311	NM_001289436	Ccdc50	7063	NM_001243046	Dlg2	6250
NM 001289457	Treml1	1241	NM 026202	Ccdc50	7017	NM 001289457	Treml1	1241
NM 027763	Treml1	1256	NM 146737	Olfr494	945	NM 027763	Treml1	1256
NM 008325	Idua	4430	NM 146733	Olfr482	972	NM 008325	Idua	4430
NM 010815	Grap2	5475	NM 146310	Olfr493	945	NM 010815	Grap2	5475
NM 001289451	Treml1	1138	NM 146652	Olfr13	933	NM 001289451	Treml1	1138
NM 001289473	Erbb2ip	6530	NM 146496	Olfr486	945	NM 008604	Mme	5722
NM_001289476	Psma7	1591	NM_001167918	D830031N03Rik	8311	NM_001289495	Txk	2277
NM 001289475	Erbb2ip	6557	NM 001243046	Dlg2	6250	NM 001289529	Ankib1	6253
NM 001289474	Erbb2ip	6206	NM 008325	Idua	4430	NM 013698	Txk	2344
NM 001005868	Erbb2ip	6674	NM 011256	Pitpnm2	6970	NM 001289516	Poli	2868
NM 011969	Psma7	952	NM 001289503	Ispd	2400	NM 001289494	Txk	2194
NM 008604	Mme	5722	NM 001289495	Txk	2277	NM 178748	Egflam	4864
NM 001289495	Txk	2277	NM 013698	Txk	2344	NM 001136090	Poli	2937
NM 001163517	Pex5l	2200	NM 001289494	Txk	2194	NM 001289498	Egflam	4883
NM 013698	Txk	2344	NM 001289521	Zeb2	8898	NM 001122754	Txk	2260
NM 001289516	Poli	2868	NM 178629	Ispd	2818	NM 001289504	Ispd	3246
NM 001289536	Stx18	2441	NM 001122754	Txk	2260	NM 001289527	Ankib1	6644
NM 001289526	Tcerg1	4397	NM 175336	Tecpr2	7955	NM 001163516	Pex5l	3078
NM 001289507	Tfr2	3578	NM 019636	Tbc1d1	5383	NM 001289528	Ankib1	6508
NM 001289494	Txk	2194	NM 001289502	Ispd	2668	NM 001289496	Egflam	4888
NM 001289521	Zeb2	8898	NM 015753	Zeb2	9235	NM 001289515	Poli	3582
NM 001136090	Poli	2937	NM 001289573	Spata7	1570	NM 001003909	Ankib1	6626
NM 001122754	Txk	2260	NM 001289590	Zfp12	5346	NM 021483	Pex5l	3188
NM 001163516	Pex5l	3078	NM 028349	Sass6	4309	NM 001289505	Pex5l	3059
NM 175336	Tecpr2	7955	NM 177681	Zfp12	5180	NM 001045486	Zfp180	3924
NM 001289515	Poli	3582	NM 001289601	Pear1	4510	NM 001289640	Zfp180	3919
NM 019636	Tbc1d1	5383	NM 178914	Spata7	2071	NM 022332	St7	2286
NM 015753	Zeb2	9235	NM 033476	Tfcp2	3301	NM 033476	Tfcp2	3301
NM 015799	Tfr2	3537	NM 001289603	Tfcp2	3295	NM 001083315	St7	2217
NM 021483	Pex5l	3188	NM 031394	Sytl2	4659	NM 001289603	Tfcp2	3295
NM 001289505	Pex5l	3059	NM 001289584	Sytl2	4698	NM 031394	Sytl2	4659
NM 001040087	Sytl2	2938	NM 001289574	Spata7	2137	NM 001289614	Btn2a2	2956
NM 001040088	Sytl2	2866	NM 001289561	Mpv17l	3357	NM 001289584	Sytl2	4698
NM 001045486	Zfp180	3924	NM 001040085	Sytl2	4731	NM 001040085	Sytl2	4731
NM 001289640	Zfp180	3919	NM 028707	Psd2	4628	NM 001289634	Zfp180	4028
NM 001289601	Pear1	4510	NM 001289571	Sass6	4248	NM 001289583	Sytl2	4779
NM 033476	Tfcp2	3301	NM 001289602	Psd2	4622	NM 001289625	St7	2195
NM 001289586	Sytl2	2986	NM 001289583	Sytl2	4779	NM 001289594	Kynu	2807
NM 001289603	Tfcp2	3295	NM 001289568	Sass6	4392	NM 001289637	Zfp180	3843
NM 144911	Rpap2	2322	NM 001289596	Slc25a40	2819	NM 001289641	Zfp180	3884
NM 031394	Sytl2	4659	NM 001289600	Pear1	4269	NM 172483	Zfp180	3728
NM 001289584	Sytl2	4698	NM 001289714	Cpvl	1490	NM 001289683	Prrg1	3973
NM 001289561	Mpv17l	3357	NM 001289683	Prrg1	3973	NM 001289663	Fam71f1	1596
NM 001040085	Sytl2	4731	NM 027749	Cpvl	1708	NM 207258	Fam71f1	1587
NM 001289634	Zfp180	4028	NM 001289653	Nell2	3168	NM 001289656	Agbl3	5261

NM 027552	Kynu	3070	NM 001289685	Iqsec3	6575	NM 001289706	Clec2i	2406
NM 001289583	Sytl2	4779	NM 001289701	Pde4dip	8413	NM 001289653	Nell2	3168
NM 001289594	Kynu	2807	NM 001289702	Pde4dip	8260	NM 020257	Clec2i	2319
NM 001289593	Kynu	1604	NM 027322	Prrg1	3904	NM 001289665	Fam71f1	1591
NM_001289637	Zfp180	3843	NM_026054	28104740 19Rik	6094	NM_027322	Prrg1	3904
NM 001289641	Zfp180	3884	NM 001033354	Iqsec3	6861	NM 001289664	Fam71f1	1590
NM 001289585	Sytl2	3031	NM 001164275	Prrg1	3877	NM 199059	Tbpl2	1734
NM 001289600	Pear1	4269	NM 008673	Nat1	1347	NM 001289708	Clec2i	2248
NM 172483	Zfp180	3728	NM 133982	Rpp25	1361	NM 001289657	Agbl3	5235
NM 001289656	Agbl3	5261	NM 178757	Irf2bp1	2741	NM 001289658	Agbl3	1328
NM 001289666	Tmem241	3206	NM 146364	Olfr495	993	NM 001164275	Prrg1	3877
NM 001289659	Upp2	2256	NM 199158	Tas2r134	897	NM 001289689	Tbpl2	1731
NM 001289685	Iqsec3	6575	NM 146498	Olfr490	945	NM 178630	Agbl3	5246
NM 001289701	Pde4dip	8413	NM 184109	Rtl1	5235	NM 147011	Olfr1044	945
NM 001289702	Pde4dip	8260	NM 146425	Olfr470	945	NM 146497	Olfr492	945
NM 001001160	Fbxo41	6688	NM 001001452	Tas2r143	882	NM 207017	Tas2r109	951
NM 199059	Tbpl2	1734	NM 001018087	Ldoc1	1397	NM 181749	Gpr142	1098
NM_026054	28104740 19Rik	6094	NM_001025385	Tas2r137	1002	NM_146861	Olfr9	939
NM 029692	Upp2	2202	NM 146499	Olfr484	966	NM 001008499	Taar4	1044
NM 001289657	Agbl3	5235	NM 146738	Olfr497	945	NM 207024	Tas2r121	918
NM 001033354	Iqsec3	6861	NM 025724	Ccer1	1868	NM 020291	Olfr480	1029
NM 001289667	Tmem241	3046	NM 029383	Cldn22	994	NM 203396	Fam115e	3110
NM 178801	Tmem241	3255	NM 001010831	Taar9	1047	NM 001001451	Tas2r138	996
NM 001289689	Tbpl2	1731	NM 146743	Olfr507	951	NM 001010828	Taar6	1038
NM 178630	Agbl3	5246	NM 011865	Pcbp1	1711	NM 207026	Tas2r124	930
NM 008673	Nat1	1347	NM 146734	Olfr478	945	NM 020502	Tas2r108	894
NM 172126	Adam1a	3114	NM 027113	Wdr5b	1806	NM 183296	Krtap16-3	570
NM 032399	Gpr87	1163	NM 025725	Ccdc96	3584	NM 207027	Tas2r125	936
NM 138742	Nap1l3	2861	NM 198103	Exoc8	7427	NM 146592	Olfr1086	933
NM 021545	Naip7	4841	NM 146184	B3gnt8	1867	NM 027113	Wdr5b	1806
NM 001025385	Tas2r137	1002	NM 011438	Sox12	4436	NM 025725	Ccdc96	3584
NM 144790	Ankrd33	1457	NM 009436	Tssk2	1403	NM 022816	Acot10	1557
NM 031367	Ifi44l	2279	NM 207023	Tas2r120	888	NM 177749	Kir3dl1	1557
NM 198425	Eid2	1299	NM 009435	Tssk1	1466	NM 021317	Dnajb7	1186
NM 011746	Mkrn3	2565	NM 053008	Olig3	2086	NM 001098723	Yy2	2867
NM 025725	Ccdc96	3584	NM 009633	Adra2b	3936	NM 146184	B3gnt8	1867
NM 027438	Pnma1	2360	NM 146311	Olfr510	945	NM 028712	Rap2b	4197
NM 198103	Exoc8	7427	NM 001013811	Fam169b	2831	NM 146762	Olfr1013	918
NM 008057	Fzd7	4840	NM 173427	Klhdc7a	5871	NM 011438	Sox12	4436
NM_172125	Adam1b	2884	NM_015777	Igbp1b	1356	NM_133359	Krtap19-9b	461
NM 053201	Magee1	3532	NM 008151	Gpr12	2271	NM 009435	Tssk1	1466
NM 175439	Mars2	2882	NM 177271	Samd5	2495	NM 146739	Olfr502	945
NM 146311	Olfr510	945	NM 011839	Mab21l2	2703	NM 009633	Adra2b	3936
NM 001013811	Fam169b	2831	NM 008301	Hspa2	2595	NM 207278	Tigd4	3459
NM 177648	Dolk	2126	NM 177013	Tmem229a	4985	NM 001011533	Olfr688	966
NM 153762	Rnf26	2294	NM 146307	Olfr498	993	NM 053201	Magee1	3532
NM 146140	Tram1l1	2225	NM 001289733	Dach2	5708	NM 172871	Klhl9	4217
NM 023266	Zfp120	4032	NM 133197	Mcf2	3671	NM 138682	Lrrc4	3636
NM 138655	Tmc2	3216	NM 001289730	Mcf2	3719	NM 172896	Nlrp4a	3575
NM 181266	Zfp120	4096	NM 001289729	Xpnpep2	3699	NM 031873	Tas1r2	3060
NM_175249	Psapl1	2548	NM_177656	6820408C 15Rik	1701	NM_146311	Olfr510	945
NM_009028	Rasl2-9	1039	NM_001289738	6820408C 15Rik	1704	NM_198414	Paqr9	2305
NM 177013	Tmem229a	4985	NM 010553	Il18rap	4889	NM 207025	Tas2r123	1002
NM 011881	Grk1	7462	NM 021470	Rnf32	1578	NM 026642	Trmt12	4133
NM 001103199	Skint6	3824	NM 001289822	Lrrc43	2024	NM 011321	Sbp	839
NM 001085421	Tspyl5	4040	NM 178403	Pus7	3907	NM 175497	Actbl2	2737
NM 001289736	Nxf2	2319	NM 001289780	Pus7	3925	NM 146140	Tram1l1	2225

NM_031259	Nxf2	2377	NM_001033461	Lrrc43	2046	NM_023266	Zfp120	4032
NM_001289733	Dach2	5708	NM_001289781	Pus7	4258	NM_001048219	Nlrp9a	3307
NM_001289729	Xpnpep2	3699	NM_001289859	Eps15l1	2727	NM_001048220	Nlrp9a	3142
NM_001289748	Tgm6	3308	NM_025271	Actl7b	1424	NM_181266	Zfp120	4096
NM_001289747	Tgm6	3471	NM_053139	Pcdhb14	3438	NM_177013	Tmem229a	4985
NM_001289749	Tgm6	2965	NM_144848	Eppk1	21627	NM_147013	Olfr1038-ps	1212
NM_177726	Tgm6	3327	NM_001033380	Itpripl2	6891	NM_025720	Krtap3-2	1031
NM_010553	Il18rap	4889	NM_013915	Zbtb18	4954	NM_001085421	Tspyl5	4040
NM_001013372	Nrp	1170	NM_001080817	Prdm10	3408	NM_001289736	Nxf2	2319
NM_030705	Mesdc1	3847	NM_172481	Nlrp4b	3274	NM_172930	Tmem255a	3431
NM_198022	Tnrc6c	8740	NM_177115	3-Mar	1754	NM_001289727	Tmem255a	1797
NM_016879	Krt85	2594	NM_198624	Ubqlnl	2298	NM_031259	Nxf2	2377
NM_144848	Eppk1	21627	NM_175936	Vmn2r81	3305	NM_001289733	Dach2	5708
NM_001033380	Itpripl2	6891	NM_183312	Synm	4513	NM_133197	Mcf2	3671
NM_013915	Zbtb18	4954	NM_013877	Cabp5	1745	NM_001289730	Mcf2	3719
NM_177115	3-Mar	1754	NM_027934	Rnf180	3785	NM_177656	6820408C15Rik	1701
NM_001013804	Flg2	7755	NM_199065	Slitrk1	4266	NM_001289738	6820408C15Rik	1704
NM_178715	Tmem30b	3282	NM_010694	Lcn3	684	NM_183307	Ccdc63	2287
NM_152821	Purg	1207	NM_020287	Insm2	3102	NM_030705	Mesdc1	3847
NM_199065	Slitrk1	4266	NM_146426	Olfr469	945	NM_028547	Kif2b	2270
NM_181564	Dsg4	3478	NM_011954	Prl2c4	869	NM_028927	Tktl2	2039
NM_181276	Tas2r136	984	NM_025613	Eid1	2559	NM_053139	Pcdhb14	3438
NM_001010834	Slc10a5	3923	NM_027105	Krtap26-1	973	NM_001033380	Itpripl2	6891
NM_020287	Insm2	3102	NM_011105	Pkdrej	7058	NM_172481	Nlrp4b	3274
NM_011105	Pkdrej	7058	NM_176834	Rnf208	1351	NM_001013804	Flg2	7755
NM_176834	Rnf208	1351	NM_001014398	Trcg1	2727	NM_017471	Pbsn	805
NM_001033474	Atxn7l3b	3607	NM_178376	Rraga	1653	NM_031249	Cstf2t	3764
NM_172939	Sowahc	4513	NM_010727	Lnx1	2726	NM_001011748	Olfr867	996
NM_001145826	Speccl1	6307	NM_009611	Actl7a	1513	NM_175936	Vmn2r81	3305
NM_177124	Tnrc6b	17125	NM_134252	Trpm8	3869	NM_013877	Cabp5	1745
NM_009611	Actl7a	1513	NM_178244	Teddm1	1415	NM_172980	Slc28a2	3904
NM_146245	Lrit1	4337	NM_010750	Mab21l1	2793	NM_181490	Cldn17	1172
NM_001033338	Rimbp3	5848	NM_001160252	Cabp2	775	NM_020287	Insm2	3102
NM_026234	Pigm	7569	NM_010353	Gsg2	2818	NM_001008429	Taar3	1032
NM_080455	Tshz2	5139	NM_012050	Omd	1751	NM_011105	Pkdrej	7058
NM_026520	Fam122a	1696	NM_028974	Kbtbd13	2969	NM_146321	Olfr186	930
NM_018778	Cldn8	2368	NM_178786	Skint4	3997	NM_053222	Vmn1r52	930
NM_010750	Mab21l1	2793	NM_031389	Nlrp4c	3614	NM_177010	-	1933
NM_182698	Myo15	7953	NM_008671	Nap1l2	2606	NM_001145826	Speccl1	6307
NM_001103171	Myo15	11715	NM_177721	Ranbp6	4867	NM_177124	Tnrc6b	17125
NM_001163557	Ppfibp2	3850	NM_001168223	Clec2g	2292	NM_146245	Lrit1	4337
NM_011154	Ppp2r3d	4180	NM_001289926	2010111I01Rik	6778	NM_026234	Pigm	7569
NM_177292	Wscd2	4332	NM_011283	Rp1	7508	NM_080455	Tshz2	5139
NM_010862	Myo15	11769	NM_053249	Krt82	2100	NM_134252	Trpm8	3869
NM_007605	Capza3	1265	NM_001168224	Clec2g	2404	NM_001039220	AI429214	1974
NM_001039223	Gm14137	3067	NM_001163587	Etnppl	2666	NM_001163557	Ppfibp2	3850
NM_001101535	Ccdc8	2058	NM_177878	Mblac1	1308	NM_023396	Rprm	1460
NM_012050	Omd	1751	NM_028235	Ttc30b	2634	NM_001146087	Il1f5	1848
NM_010192	Fem1a	6834	NM_029823	Arxes2	1540	NM_010353	Gsg2	2818
NM_028974	Kbtbd13	2969	NM_001081228	Ttc30a2	2008	NM_001103179	Brwd1	7547
NM_178786	Skint4	3997	NM_030188	Ttc30a1	2862	NM_198412	Dnajc6	5139
NM_015737	Galnt4	5136	NM_080847	Asb15	4268	NM_001165935	Aven	1102
NM_026286	Ftmt	1461	NM_019943	Papolb	2337	NM_177721	Ranbp6	4867
NM_001163415	Ppp2r3d	4414	NM_024190	Chmp1b	2529	NM_001168223	Clec2g	2292
NM_001201460	H2-Q9	1539	NM_023511	Krtap3-1	604	NM_001289895	Tns1	9905
NM_198412	Dnajc6	5139	NM_001177977	Acsm2	6617	NM_001289926	2010111I01Rik	6778

NM 008671	Nap1l2	2606	NM 010102	S1pr4	2393	NM 001195662	Rp1	3047
NM 028908	Map10	3548	NM 001163271	Fscb	3566	NM 001168224	Clec2g	2404
NM 177721	Ranbp6	4867	NM 146365	Olfr1094	1147	NM 175520	Hcar1	3285
NM 145836	Irf2bpl	4112	NM 001172054	Il11ra1	1847	NM 053211	Tas2r103	939
NM 001168223	Clec2g	2292	NM 019461	Usp27x	3240	NM 028514	Actrt1	1339
NM 001289878	Per3	6048	NM 177305	Arl4c	3967	NM 029823	Arxes2	1540
NM 011067	Per3	5996	NM 183148	Iffo2	5352	NM 080847	Asb15	4268
NM 001289877	Per3	5999	NM 175402	Rbm15b	3016	NM 024190	Chmp1b	2529
NM 001195662	Rp1	3047	NM 001252616	Fut8	2106	NM 145890	Grhl1	3100
NM 053249	Krt82	2100	NM 175204	Psmb11	4247	NM 001204820	Myadml2	1128
NM 001168224	Clec2g	2404	NM 001252449	Strada	2396	NM 011509	Gm3258	710
NM 175520	Hcar1	3285	NM 013779	Magel2	4662	NM 001252208	Kcnj16	3695
NM 029823	Arxes2	1540	NM 033606	Dqx1	3158	NM 011663	Zrsr1	4492
NM 134420	Slc26a6	3329	NM 026006	Sft2d3	2810	NM 146869	Olfr147	1175
NM 080847	Asb15	4268	NM 153100	Rtp3	2460	NM 001163271	Fscb	3566
NM 024190	Chmp1b	2529	NM 198224	Ms4a13	1375	NM 019461	Usp27x	3240
NM 145890	Grhl1	3100	NM 029307	Hspb9	726	NM 001167936	Zyg11a	2289
NM 001252208	Kcnj16	3695	NM 008763	Olfr16	1050	NM 177305	Arl4c	3967
NM 001177977	Acsm2	6617	NM 001205099	Npy2r	3022	NM 183148	Iffo2	5352
NM 010658	Mafb	3389	NM 153072	Hus1b	1186	NM 019468	G6pd2	1635
NM 001168248	Rmi1	3607	NM 001146275	Iigp1	3149	NM 175116	Lpar6	2468
NM_011663	Zrsr1	4492	NM_001033335	Serpina3f	2213	NM_028117	Chst14	2089
NM 001166552	Zbed6	5007	NM 001201378	Ccdc136	3225	NM 001177397	Anks1b	7246
NM 001252519	Zfp82	2030	NM 008272	Hoxc9	1719	NM 001252616	Fut8	2106
NM 001172054	Il11ra1	1847	NM 001177881	Mfap3l	6480	NM 146436	Olfr998	1012
NM 183148	Iffo2	5352	NM 008022	Foxd4	2345	NM 177753	Sox21	3798
NM 001195084	Plscr2	1900	NM 145574	Ccdc136	3507	NM 033606	Dqx1	3158
NM 019468	G6pd2	1635	NM 001166532	Sfmbt1	7837	NM 011667	Uba1y	3981
NM 175204	Psmb11	4247	NM 008088	Gas7	6911	NM 178059	Etl4	5942
NM 001252449	Strada	2396	NM 021442	Mecom	1075	NM 183174	Homez	5633
NM 026006	Sft2d3	2810	NM 008127	Gjb4	1445	NM 181347	Dennd1b	8072
NM 178059	Etl4	5942	NM 009027	Rasgrf2	7595	NM 001205099	Npy2r	3022
NM 183174	Homez	5633	NM 028274	Exosc6	1346	NM 001146275	Iigp1	3149
NM 198224	Ms4a13	1375	NM 001286567	Eml4	5364	NM 026751	Myadml2	2325
NM 008763	Olfr16	1050	NM 010592	Jund	2894	NM 001177881	Mfap3l	6480
NM 020597	Msmb	522	NM 009259	Spn	3751	NM 001164519	Ctnna3	2840
NM 001163218	Prl3c1	865	NM 001286101	Fam65b	3401	NM 207154	Olfr1102	1063
NM 133940	Fbxl14	4219	NM 001286959	Sptssb	1567	NM 001171640	Actl10	1501
NM 181347	Dennd1b	8072	NM 001037810	Spn	1481	NM 146580	Olfr1020	1079
NM 001205099	Npy2r	3022	NM 001286758	Tiam2	2550	NM 001166532	Sfmbt1	7837
NM 001168538	Cdadc1	3166	NM 001284344	Gas2l3	5760	NM 029895	Etl4	5909
NM 001177881	Mfap3l	6480	NM 001285466	Dennd6a	6512	NM 033077	D1Pas1	3212
NM_001167996	1110032F04Rik	2578	NM_001285467	Dennd6a	6727	NM_009027	Rasgrf2	7595
NM 001164519	Ctnna3	2840	NM 001289741	Mturn	3347	NM 145151	Crebzf	5263
NM 021350	Chml	6117	NM 001172154	Dnase1l1	1518	NM 001284521	Larp4	6527
NM 001166532	Sfmbt1	7837	NM 022989	Arl6ip6	2553	NM 009999	Cyp2b10	1878
NM 029895	Etl4	5909	NM 007379	Abca2	8061	NM 001285412	Calu	3046
NM 021442	Mecom	1075	NM 022993	Lrp10	3013	NM 001284344	Gas2l3	5760
NM 009027	Rasgrf2	7595	NM 001037756	Brms1l	2566	NM 001285466	Dennd6a	6512
NM 145151	Crebzf	5263	NM 001037759	Sgk3	5398	NM 027979	Chit1	1815
NM 001286567	Eml4	5364	NM 001172207	Lrtm2	3408	NM 001285467	Dennd6a	6727
NM 009259	Spn	3751	NM 007387	Acp2	4669	NM 009599	Ache	2224
NM 001289833	Apoc3	459	NM 023041	Pex19	3131	NM 007376	Pzp	4681
NM 001284521	Larp4	6527	NM 023042	Recql	3389	NM 001172154	Dnase1l1	1518
NM 009999	Cyp2b10	1878	NM 023045	Xpo7	15118	NM 022989	Arl6ip6	2553
NM 001285412	Calu	3046	NM 023046	Asb1	5772	NM 001037759	Sgk3	5398
NM 008527	Klrb1c	2632	NM 001037841	Cklf	1871	NM 001172207	Lrtm2	3408
NM 194064	Nanos2	1439	NM 023053	Twsg1	4071	NM 023045	Xpo7	15118
NM 001289741	Mturn	3347	NM 001037863	Atp11c	6070	NM 023046	Asb1	5772
NM 007377	Aatk	5706	NM 001037878	Tcf25	2851	NM 023053	Twsg1	4071

NM_001172154	Dnase1l1	1518	NM_007406	Adcy7	6121	NM_001037863	Atp11c	6070
NM_022989	Arl6ip6	2553	NM_001173459	LOC100038947	1456	NM_001037865	Col28a1	4230
NM_007379	Abca2	8061	NM_001173460	Sirpb1b	1496	NM_007404	Adam9	4003
NM_022993	Lrp10	3013	NM_001173477	Speg	1423	NM_007406	Adcy7	6121
NM_001037756	Brms1l	2566	NM_001173506	Map1a	11553	NM_023063	Lima1	4117
NM_001037757	Wash	2887	NM_001037909	C130026I21Rik	1887	NM_007407	Adcyap1r1	6262
NM_022995	Pmepa1	4531	NM_001037916	Ccdc17	2003	NM_023065	Ifi30	997
NM_001037758	Btrc	6358	NM_001174049	Cacna2d2	5512	NM_001173500	G630090E17Rik	1782
NM_001037759	Sgk3	5398	NM_001174050	Cacna2d2	5515	NM_001173506	Map1a	11553
NM_007387	Acp2	4669	NM_025275	Amz2	2816	NM_001037909	C130026I21Rik	1887
NM_023041	Pex19	3131	NM_025276	Evpl	6398	NM_007422	Adss	2553
NM_007388	Acp5	1367	NM_001037937	Deptor	8371	NM_001037917	Gm6377	2801
NM_001037801	Cd6	2917	NM_001174107	Map3k9	10619	NM_001174049	Cacna2d2	5512
NM_023044	Slc15a3	2345	NM_025280	Kin	1379	NM_001174050	Cacna2d2	5515
NM_007391	Acrv1	1096	NM_001037957	Dyrk1b	2524	NM_001037928	Gm11992	2400
NM_023045	Xpo7	15118	NM_001037987	Edil3	5358	NM_025278	Gng12	4259
NM_001172481	Aspn	2345	NM_001037997	Fert2	3120	NM_001037929	Gm14092	1814
NM_023048	Asb4	3286	NM_007440	Alox12	2991	NM_001174107	Map3k9	10619
NM_023056	Tmem176b	1670	NM_023127	Polr2k	494	NM_001037941	Dnajb6	1120
NM_001037846	Cnot2	2816	NM_025283	Mob4	2845	NM_001037955	Dusp22	3058
NM_001037847	Cnot2	2746	NM_001174155	Rasgrp4	4636	NM_007440	Alox12	2991
NM_007402	Adam7	3469	NM_001038602	Marveld2	3061	NM_025283	Mob4	2845
NM_001037863	Atp11c	6070	NM_025289	Tbrg1	1583	NM_001038230	Anapc11	3384
NM_007404	Adam9	4003	NM_001177313	Orc4	1397	NM_001038590	Cldn19	888
NM_001037877	Tcf25	3079	NM_024431	Morf4l1	1896	NM_023134	Sftpa1	2775
NM_023065	Ifi30	997	NM_001177319	Tfpi	3027	NM_001038602	Marveld2	3061
NM_007414	Adprh	1583	NM_001177320	Tfpi	2993	NM_001177319	Tfpi	3027
NM_001173506	Map1a	11553	NM_025292	Synj2bp	10398	NM_001177320	Tfpi	2993
NM_001173553	Cdc14a	4338	NM_001045540	Gm12185	5287	NM_025291	Sra1	979
NM_007421	Adssl1	1806	NM_025294	Natd1	4159	NM_025292	Synj2bp	10398
NM_007422	Adss	2553	NM_007463	Speg	10795	NM_001038604	Clec5a	3820
NM_023120	Gnb1l	3567	NM_025296	Ciao1	3075	NM_001177349	Pydc4	2687
NM_001037923	Lekr1	2463	NM_007466	Api5	3702	NM_001177350	Pydc4	2635
NM_001037928	Gm11992	2400	NM_007467	Aplp1	2408	NM_025299	Txnl4a	4071
NM_025278	Gng12	4259	NM_025299	Txnl4a	4071	NM_001038621	Rabgap1l	6338
NM_023122	Gpm6b	2932	NM_001038621	Rabgap1l	6338	NM_001177371	Dbn1	3044
NM_007435	Abcd1	3421	NM_001177371	Dbn1	3044	NM_001177372	Dbn1	2909
NM_001037955	Dusp22	3058	NM_001177372	Dbn1	2909	NM_007478	Arf3	3481
NM_001037987	Edil3	5358	NM_025304	Lcmt1	1369	NM_001038625	Sertad2	5519
NM_001037997	Fert2	3120	NM_001038625	Sertad2	5519	NM_023143	C1ra	2762
NM_001038015	Gnpda2	1867	NM_001177374	Ubr2	7696	NM_001177379	Cpeb2	6757
NM_025283	Mob4	2845	NM_001038635	Stk35	5454	NM_001038643	Slco3a1	2857
NM_001038492	Ctsa	3548	NM_001177379	Cpeb2	6757	NM_001177389	4933403008Rik	1520
NM_146327	Olfr129	1238	NM_001177380	Cypt15	576	NM_025311	Tmem254a	1980
NM_001038676	Gm4745	1288	NM_007487	Arl4a	3657	NM_001177396	Anks1b	4905
NM_024431	Morf4l1	1896	NM_007488	Arnt2	6073	NM_001177398	Anks1b	4728
NM_025290	Rsph1	1164	NM_001038660	Slc7a15	2350	NM_007495	Astn1	7217
NM_025291	Sra1	979	NM_007495	Astn1	7217	NM_025332	Gtpbp8	1318
NM_025292	Synj2bp	10398	NM_007501	Neurod4	3376	NM_007508	Atp6v1a	3946
NM_001045540	Gm12185	5287	NM_007504	Atp2a1	3486	NM_001038846	Rcsd1	2580
NM_025294	Natd1	4159	NM_001038710	Tmod2	9957	NM_007513	Slc7a1	7195
NM_007463	Speg	10795	NM_001038846	Rcsd1	2580	NM_001039000	Kif5a	6334
NM_001038608	Txnl4a	1683	NM_025335	Tmem167	10809	NM_001039039	Kctd21	2975
NM_001177349	Pydc4	2687	NM_007511	Atp7b	4711	NM_001039050	Pkib	5581
NM_025296	Ciao1	3075	NM_007514	Slc7a2	7731	NM_001039056	Kcnj15	5046
NM_001177350	Pydc4	2635	NM_001039000	Kif5a	6334	NM_001039057	Kcnj15	5138
NM_007467	Aplp1	2408	NM_001039038	Nhlrc4	2141	NM_007525	Bard1	5448

NM_007473	Aqp7	2524	NM_001039045	Pigc	3288	NM_001039061	Klhl15	2363
NM_023141	Tor3a	3268	NM_007528	Bcl6b	3426	NM_007530	Bcap29	1954
NM_001038624	Ric3	5303	NM_025351	Chchd6	1126	NM_025356	Ube2d3	2619
NM_007478	Arf3	3481	NM_007532	Bcat1	7868	NM_007532	Bcat1	7868
NM_025305	Mrps7	1354	NM_001177408	Gm15319	2514	NM_001177408	Gm15319	2514
NM_001177374	Ubr2	7696	NM_025359	Tspan13	1923	NM_025359	Tspan13	1923
NM_007486	Arhgdib	1100	NM_025366	Chchd1	619	NM_001177437	Mroh4	3549
NM_007487	Arl4a	3657	NM_001177437	Mroh4	3549	NM_025373	1110008L16Rik	3117
NM_001038651	Zfp953	4641	NM_007546	Bik	943	NM_007549	Blk	2475
NM_001177392	Gm13547	629	NM_007551	Cxcr5	2636	NM_001177464	Zfp516	7703
NM_025311	Tmem254a	1980	NM_001177464	Zfp516	7703	NM_146409	Olfr1080	979
NM_001038653	Slc16a3	2481	NM_025381	Atp6v1f	635	NM_025381	Atp6v1f	635
NM_025312	Sostdc1	1751	NM_025387	Tmem14c	969	NM_001177480	Gm14391	946
NM_001038654	Slc16a3	2386	NM_007562	Bnc1	4663	NM_007563	Bpgm	2068
NM_001038660	Slc7a15	2350	NM_025391	Nip7	3036	NM_025389	Anapc11	3229
NM_007494	Ass1	1631	NM_007575	Ciita	5254	NM_025391	Nip7	3036
NM_007496	Zfhx3	16439	NM_025401	Ubl5	1939	NM_007569	Btg1	5010
NM_007501	Neurod4	3376	NM_001177505	Zkscan7	2228	NM_001039088	Sehl1	3542
NM_001038703	Gpr146	4145	NM_001039103	Rasa4	2795	NM_007588	Calcr	3831
NM_001038710	Tmod2	9957	NM_025412	Pycrl	1353	NM_007592	Car8	5722
NM_001177399	Etohi1	3345	NM_001039114	Acsbg2	2608	NM_025419	1110059G10Rik	3361
NM_025335	Tmem167	10809	NM_001039115	Zkscan4	2471	NM_001177529	Esp6	663
NM_001177400	Etohi1	1158	NM_007592	Car8	5722	NM_001039126	Asb1	5971
NM_007514	Slc7a2	7731	NM_001177524	Gml	895	NM_001177535	Gm4340	1258
NM_001039000	Kif5a	6334	NM_025416	Them5	1251	NM_001039137	Scoc	1841
NM_001039045	Pigc	3288	NM_025420	Lce1m	877	NM_007615	Ctnnd1	5475
NM_001039056	Kcnj15	5046	NM_001177527	Zfp964	1740	NM_025437	Eif1ax	926
NM_001039057	Kcnj15	5138	NM_001177533	Apol11a	1132	NM_007626	Cbx5	8802
NM_025351	Chchd6	1126	NM_001039126	Asb1	5971	NM_023182	Ctrl	919
NM_001039071	Ldb3	4854	NM_007602	Capn5	4411	NM_001177556	Gng12	4194
NM_001039072	Ldb3	4908	NM_001177536	Prdm11	1870	NM_025446	Aig1	1054
NM_001039073	Ldb3	4737	NM_007607	Car4	1316	NM_001177557	Gng12	4206
NM_001039074	Ldb3	4954	NM_001039147	Morf4l1	2013	NM_001177558	Gng12	4227
NM_001039075	Ldb3	1506	NM_025432	Trappc2	765	NM_001039181	Npr3	6924
NM_001039076	Ldb3	1623	NM_001177543	0610010B08Rik	4539	NM_001177559	Gng12	4247
NM_025356	Ube2d3	2619	NM_025433	Rpl7l1	2606	NM_001177560	Gng12	4264
NM_007534	Bcl2a1b	764	NM_001039157	Ube2j2	3383	NM_025453	Tm4sf20	1503
NM_007536	Bcl2a1d	796	NM_001039158	Ube2j2	3357	NM_025454	Ing5	4539
NM_001039080	Rbms2	5390	NM_001039159	Ube2j2	3220	NM_001177564	Gm4027	998
NM_007543	Ceacam2	3234	NM_001177546	Zfp600	4248	NM_001177565	Gm8300	1872
NM_146409	Olfr1080	979	NM_025441	Nemf	3737	NM_025459	Fam134b	2929
NM_025385	Prr13	1229	NM_007626	Cbx5	8802	NM_001039188	Rreb1	7469
NM_025387	Tmem14c	969	NM_007627	Cckbr	2483	NM_025464	Tmem218	1057
NM_007569	Btg1	5010	NM_001039179	Bicd2	6376	NM_025467	Gkn2	777
NM_001039086	Rapgef1	6233	NM_001039180	Bicd2	4555	NM_001177573	BB287469	1851
NM_007574	C1qc	1063	NM_001039184	Cep350	13307	NM_001177574	Gm2022	1822
NM_001039087	Rapgef1	6251	NM_025456	Eapp	1143	NM_007676	Psg16	2289
NM_007576	C4bp	1883	NM_025458	Tmed6	863	NM_001039244	Gm7120	1829
NM_001039094	Negr1	5002	NM_007644	Scarb2	2110	NM_025509	Ostc	1081
NM_025408	Acer3	4058	NM_025464	Tmem218	1057	NM_007684	Cetn3	1013
NM_001039106	Ddhd1	5015	NM_007647	Entpd5	4914	NM_001177600	Adam23	2880
NM_001177527	Zfp964	1740	NM_023186	Chia1	1557	NM_001039364	Mobp	3207
NM_025423	1110059E24Rik	1428	NM_001039200	Emc1	6228	NM_007688	Cfl2	2940
NM_001177538	Duxbl2	2334	NM_024173	Atp6v1g1	1109	NM_001039386	Nsmf	2949
NM_001177539	Duxbl3	2334	NM_007662	Cdh15	2843	NM_001039387	Nsmf	2943
NM_007606	Car3	1537	NM_025483	Senp7	4883	NM_023200	Ppp1r7	3653
NM_001039147	Morf4l1	2013	NM_007677	Psg17	2126	NM_001039389	Wdr37	3802

NM_001177543	0610010B08Rik	4539	NM_001039364	Mobp	3207	NM_025534	Ccdc82	4665
NM_001039157	Ube2j2	3383	NM_001039365	Mobp	1499	NM_001177621	Slc5a6	3455
NM_001039158	Ube2j2	3357	NM_001039368	Polr2k	626	NM_001177622	Slc5a6	3237
NM_001039159	Ube2j2	3220	NM_001039376	Pde4dip	8420	NM_001177623	Arpp21	2642
NM_001039160	Gvin1	8846	NM_007692	Chkb	1768	NM_001177624	Slc5a12	5092
NM_001039162	Clip2	4908	NM_001039388	Wdr37	4634	NM_025546	Rsl1d1	1839
NM_025439	Tmem9	1460	NM_001039389	Wdr37	3802	NM_001039472	Kif21b	9119
NM_007626	Cbx5	8802	NM_001039390	Pkig	1139	NM_001177628	Slc45a3	3267
NM_001177556	Gng12	4194	NM_001039391	Pkig	1053	NM_001039482	Klhl20	4037
NM_001039179	Bicd2	6376	NM_001039394	Rab43	4605	NM_007713	Clk3	2494
NM_001177557	Gng12	4206	NM_025534	Ccdc82	4665	NM_001039485	Piezo2	10742
NM_001177558	Gng12	4227	NM_025538	Alkbh7	939	NM_001177630	Etl4	5714
NM_001039181	Npr3	6924	NM_007708	Cit	6898	NM_001177631	Etl4	7406
NM_001177559	Gng12	4247	NM_025545	Aptx	5689	NM_001039495	Ccdc108	5718
NM_001177560	Gng12	4264	NM_001039472	Kif21b	9119	NM_025575	Sys1	1623
NM_025452	Tmem54	1102	NM_001039478	Lrif1	1612	NM_007729	Col11a1	7275
NM_025453	Tm4sf20	1503	NM_007713	Clk3	2494	NM_001177654	Nsmf	2934
NM_001039188	Rreb1	7469	NM_001039488	Lrif1	3370	NM_007733	Col19a1	10542
NM_025464	Tmem218	1057	NM_001039493	Plekhm3	8562	NM_025581	Ska1	2669
NM_007647	Entpd5	4914	NM_007721	Ccr10	1725	NM_001177655	Nsmf	2853
NM_001039200	Emc1	6228	NM_001039511	Ivns1abp	2793	NM_007737	Col5a2	6615
NM_001039214	Mex3c	4080	NM_001039512	Ivns1abp	3387	NM_001177666	Gm7120	928
NM_007653	Cd63	918	NM_001039515	Arl4a	3693	NM_025585	1700029F12Rik	1128
NM_025479	Mplkip	2672	NM_025581	Ska1	2669	NM_001039552	Ermard	2513
NM_025483	Senp7	4883	NM_001039534	Pstk	1130	NM_001039553	4930467E23Rik	2590
NM_001039231	Zfp951	2903	NM_007735	Col4a4	7808	NM_001039555	Cyp2c68	1778
NM_007677	Psg17	2126	NM_007741	Col9a2	2746	NM_025590	Acot11	5668
NM_007684	Cetn3	1013	NM_025586	Rpl15	1857	NM_001177696	Ablim2	3726
NM_001177600	Adam23	2880	NM_001039553	4930467E23Rik	2590	NM_001177697	Ablim2	3585
NM_025522	Dhrs7	1318	NM_001039555	Cyp2c68	1778	NM_001039562	Ankrd37	921
NM_001039376	Pde4dip	8420	NM_001039558	Smco3	2052	NM_025594	Zmat2	2257
NM_001039388	Wdr37	4634	NM_001177696	Ablim2	3726	NM_007752	Cp	3889
NM_025530	Cutc	1296	NM_001177697	Ablim2	3585	NM_001177698	Ablim2	3555
NM_001177615	Arpp21	3419	NM_007752	Cp	3889	NM_001177699	Ablim2	3552
NM_007699	Chrm4	2768	NM_001177698	Ablim2	3555	NM_001177700	Ablim2	3453
NM_025534	Ccdc82	4665	NM_001177699	Ablim2	3552	NM_001039644	Edem3	6604
NM_001177620	Arpp21	3366	NM_001177700	Ablim2	3453	NM_001039578	Evi5l	1542
NM_025539	Nudt2	929	NM_007754	Cpd	9152	NM_001177705	Homez	5788
NM_025545	Aptx	5689	NM_001177706	Arfgap1	2852	NM_001177706	Arfgap1	2852
NM_001177625	Ect2	4002	NM_001177707	Arfgap1	2729	NM_001177707	Arfgap1	2729
NM_001039474	Tcerg1	4409	NM_001177708	Arfgap1	2448	NM_001177708	Arfgap1	2448
NM_001177626	Ect2	4040	NM_001177709	Arfgap1	2442	NM_001177709	Arfgap1	2442
NM_025548	Tbcb	1173	NM_001177710	Arfgap1	2398	NM_001177710	Arfgap1	2398
NM_001039478	Lrif1	1612	NM_025601	1700029H14Rik	1343	NM_001039646	Gbp10	3528
NM_001177628	Slc45a3	3267	NM_007762	Crhr1	2428	NM_025610	Asrgl1	2274
NM_001039483	Tmco1	4474	NM_001177731	Mrap2	2093	NM_025611	Cul7	5546
NM_025554	Polr2e	1022	NM_025611	Cul7	5546	NM_007766	Pcdha4	5322
NM_007714	Clk4	1892	NM_001039695	Rhox4d	759	NM_007767	Pcdha6	5242
NM_001177630	Etl4	5714	NM_001039658	Mtl5	2037	NM_025614	Rwdd1	1082
NM_001039488	Lrif1	3370	NM_001039669	Iffo1	2833	NM_023217	Pgpep1	4882
NM_007717	Cmah	9402	NM_025619	1700019L03Rik	1675	NM_025615	2810004N23Rik	1382
NM_001039493	Plekhm3	8562	NM_007769	Dmbt1	6259	NM_025618	Sri	2439
NM_001177631	Etl4	7406	NM_001039677	Slc30a2	3313	NM_007772	Hivep1	8753
NM_001039495	Ccdc108	5718	NM_025621	2310050C09Rik	1067	NM_001039701	Il1rn	2480
NM_001039511	Ivns1abp	2793	NM_007772	Hivep1	8753	NM_023220	Sppl2a	5629

NM 001039512	Ivns1abp	3387	NM 001039692	Arhgap12	5034	NM 025631	Bpifb2	1730
NM 001039515	Arl4a	3693	NM 007774	Cryga	628	NM 025635	Zwint	2676
NM 146557	Olfr869	998	NM 001039700	Pkd1l3	6606	NM 001039951	Zfp606	2322
NM 007729	Col11a1	7275	NM 007775	Crygc	626	NM 007789	Ncan	7184
NM 007735	Col4a4	7808	NM 023220	Sppl2a	5629	NM 001040106	Aak1	19363
NM 001177669	Golph3l	2657	NM 001177750	Gm10767	1652	NM 023229	Fastk	1840
NM 001177670	Golph3l	2226	NM 001039951	Zfp606	2322	NM 007797	Ctla2b	856
NM 025591	Fam136a	1634	NM 001040106	Aak1	19363	NM 001040397	Filip1l	3946
NM 025594	Zmat2	2257	NM 025649	Mad2l1bp	1288	NM 025656	Gemin2	1106
NM 007752	Cp	3889	NM 023233	Trim13	1578	NM 007805	Cyb561	2474
NM_007754	Cpd	9152	NM_025653	3110001I22Rik	1611	NM_025659	Abi3	4038
NM 001039644	Edem3	6604	NM 001040403	Flot2	2627	NM 025662	Pigk	4744
NM 007755	Cpeb1	3138	NM 025660	Ribc1	1512	NM 025664	Snx9	2078
NM 001177705	Homez	5788	NM 001040426	Thsd4	8796	NM 001177784	Dis3l	3536
NM 025611	Cul7	5546	NM 007814	Cyp2b19	2720	NM 007813	Cyp2b13	1876
NM 023217	Pgpep1	4882	NM 001040682	Clmn	11869	NM 025668	Spcs2	2815
NM 007769	Dmbt1	6259	NM 001177789	Phactr3	4861	NM 007817	Cyp2f2	1842
NM 007772	Hivep1	8753	NM 001040683	Med15	3293	NM 001040682	Clmn	11869
NM 001039692	Arhgap12	5034	NM 001177790	Phactr3	4858	NM 025671	Ogfod2	1548
NM 001039700	Pkd1l3	6606	NM 007820	Cyp3a16	1730	NM 007820	Cyp3a16	1730
NM 023220	Sppl2a	5629	NM 001177791	Phactr3	4695	NM 025673	Golph3	2657
NM 001039889	Smok3b	2331	NM 001040686	Zfp692	1887	NM 001040690	Rap1gds1	3674
NM 001177750	Gm10767	1652	NM 007822	Cyp4a14	2503	NM 007822	Cyp4a14	2503
NM 025635	Zwint	2676	NM 007823	Cyp4b1	1871	NM 001040695	Uev1d	4607
NM 025636	Ntpcr	1155	NM 001040696	Nlrp1b	4159	NM 025681	Lix1	3124
NM 007786	Csn3	1597	NM 001040699	Mtmr7	3446	NM 025682	Pspc1	2375
NM 007789	Ncan	7184	NM 007828	Dapk3	1696	NM 001040696	Nlrp1b	4159
NM 001040106	Aak1	19363	NM 025687	Tex12	647	NM 001040699	Mtmr7	3446
NM 001040187	Ddx17	4766	NM 001042411	Lepre1	2844	NM 001042438	Zhx1	5231
NM_025653	3110001I22Rik	1611	NM_025690	Sltm	3691	NM_001042485	Tmem183a	3166
NM 001040403	Flot2	2627	NM 001042421	Kntc1	6961	NM 025696	Sorcs3	5634
NM 025658	Ms4a4d	1480	NM 001042438	Zhx1	5231	NM 025698	Tmed7	3452
NM 025662	Pigk	4744	NM 025693	Tmem41a	1359	NM 025700	Pgm1	2179
NM 007813	Cyp2b13	1876	NM 001042485	Tmem183a	3166	NM 007843	Defb1	932
NM 025668	Spcs2	2815	NM 007835	Dctn1	4437	NM 025706	Tbc1d15	3779
NM 001040682	Clmn	11869	NM 001042489	Hvcn1	2790	NM 025708	Tmem186	2878
NM 025670	Cdip1	3898	NM 007841	Ddx6	6008	NM 025709	Gapvd1	5966
NM 007820	Cyp3a16	1730	NM 025698	Tmed7	3452	NM 007853	Degs1	2005
NM 025675	Dph6	2700	NM 001042501	Fam133b	2304	NM 001042542	Akap4	2859
NM 001040690	Rap1gds1	3674	NM 007847	-	484	NM 001177841	Otub2	3239
NM 001040699	Mtmr7	3446	NM 025712	Snx31	2695	NM 001042592	Arrdc4	3903
NM 001042408	Txnl4a	1659	NM 007853	Degs1	2005	NM 025721	Spesp1	3013
NM 001177798	Taf6l	2366	NM 025714	Odf2l	2291	NM 001177850	Asph	2925
NM 025691	Srp72	3605	NM 001042557	Map2k7	1720	NM 001042630	Lcn5	916
NM 001042438	Zhx1	5231	NM 001042565	Wsb1	2346	NM 001177851	Asph	2877
NM 007834	Dscr3	2419	NM 001042592	Arrdc4	3903	NM 001177852	Asph	2726
NM 001042484	Golga7	1818	NM 007869	Dnajc1	5497	NM 001042634	Clk1	1830
NM 001042485	Tmem183a	3166	NM 007873	Doc2b	4275	NM 001177853	Asph	2693
NM 023243	Ccnh	1976	NM 025739	Rnf220	2910	NM 001177854	Asph	2555
NM 025696	Sorcs3	5634	NM 001042707	Ilf3	3564	NM 007874	Reep5	2910
NM 025698	Tmed7	3452	NM 001042708	Ilf3	3777	NM 007875	Dpagt1	1920
NM 025705	Dcbld1	3025	NM 001042709	Ilf3	3738	NM 001177858	Trim43c	1990
NM 025709	Gapvd1	5966	NM 001042714	Ankdd1b	3315	NM 001177868	Rreb1	7198
NM_025711	Aspn	2362	NM_025763	4933436I01Rik	1426	NM_007880	Arid3a	5361
NM 025712	Snx31	2695	NM 001177882	Mfap3l	6248	NM 001042670	Mterf1b	1368
NM 001042542	Akap4	2859	NM 025769	Efcab1	1128	NM 001177869	Rreb1	8403
NM 001042556	Rpf2	1830	NM 001177884	Whsc1	7806	NM 001177871	Filip1l	3253
NM 001177843	Frmd4a	6780	NM 007896	Mapre1	7330	NM 007883	Dsg2	5730
NM 025717	Rbm4b	1840	NM 023256	Krt20	1930	NM 007886	Dtnb	2326

NM_001177844	Frmd4a	5836	NM_001080709	4930402F06Rik	1371	NM_001042707	Ilf3	3564
NM_001042565	Wsb1	2346	NM_007899	Ecm1	1900	NM_001042708	Ilf3	3777
NM_001042580	Cd63	959	NM_025779	Ccdc109b	1332	NM_001042709	Ilf3	3738
NM_001042591	Arrdc3	4102	NM_007908	Eef2k	6366	NM_001042710	Prss57	2693
NM_007867	Dlx4	1820	NM_007914	Ehf	4387	NM_001042714	Ankdd1b	3315
NM_025727	Klhl10	2012	NM_001080712	Gm7534	2338	NM_001042725	Calcr	3471
NM_007869	Dnajc1	5497	NM_025793	Wdr45b	2344	NM_001177882	Mfap3l	6248
NM_007870	Dnase1l3	3702	NM_001044371	Rad17	2735	NM_007892	E2f5	1763
NM_025734	Kcng4	3822	NM_001177937	Eda	4949	NM_025770	Atg10	1609
NM_001177868	Rreb1	7198	NM_007928	Mark2	4664	NM_025771	Cntnap2	3449
NM_007881	Atn1	4426	NM_007929	Emp2	3440	NM_001043228	Dntt	2073
NM_007883	Dsg2	5730	NM_001177938	Eda	4946	NM_001177884	Whsc1	7806
NM_025747	4933411K20Rik	4539	NM_001044386	Zfx	7012	NM_007896	Mapre1	7330
NM_025751	4933425L06Rik	2157	NM_007930	Enc1	4752	NM_001079865	Ces2f	1823
NM_001177874	Gps1	1860	NM_001177939	Eda	4940	NM_025780	Thap2	3408
NM_001042714	Ankdd1b	3315	NM_001177940	Eda	4931	NM_025781	Tmem170	1943
NM_001042715	Ccdc135	2832	NM_001177941	Eda	4916	NM_001080711	Dfnb59	1217
NM_001042719	Ddhd1	5201	NM_001177942	Eda	4913	NM_001080776	Gm1123	1337
NM_001177882	Mfap3l	6248	NM_025818	1200014J11Rik	6284	NM_007914	Ehf	4387
NM_025770	Atg10	1609	NM_001177943	Eda	5105	NM_007917	Eif4e	2882
NM_025771	Cntnap2	3449	NM_001044720	Abcc9	7499	NM_025794	Etfdh	2281
NM_001177884	Whsc1	7806	NM_007938	Epha6	4213	NM_001177896	9830107B12Rik	3730
NM_007896	Mapre1	7330	NM_001044740	Slc7a2	7720	NM_001177897	9830107B12Rik	3583
NM_025778	Bcl2l14	2220	NM_001177944	Eda	5088	NM_025800	Ppp1r2	4098
NM_007900	Ect2	4095	NM_007943	Eps15	5193	NM_001044308	Cacna1i	9833
NM_007903	Edn3	2978	NM_025822	Rsrc1	3232	NM_001044371	Rad17	2735
NM_025783	Chmp3	2592	NM_001044747	Zfp68	4635	NM_001177937	Eda	4949
NM_007908	Eef2k	6366	NM_007944	Eps15l1	3159	NM_001177938	Eda	4946
NM_001081653	Cntnap5c	3918	NM_001045481	Ifi203	3633	NM_001044386	Zfx	7012
NM_007914	Ehf	4387	NM_025825	Appbp2	2407	NM_001177939	Eda	4940
NM_001077425	Cntnap5a	3915	NM_025828	Lman2	4315	NM_001177940	Eda	4931
NM_001080712	Gm7534	2338	NM_007955	Ptprv	6080	NM_001177941	Eda	4916
NM_007917	Eif4e	2882	NM_001177964	Dcdc2c	1434	NM_001177942	Eda	4913
NM_025799	Fuca2	3954	NM_025833	Baiap2l1	3248	NM_025818	1200014J11Rik	6284
NM_025804	Tcf25	3076	NM_001045807	Rbm15	3270	NM_001177943	Eda	5105
NM_001044308	Cacna1i	9833	NM_007961	Etv6	5541	NM_001044720	Abcc9	7499
NM_007926	Aimp1	1122	NM_007964	Evi5	5849	NM_025820	Crnkl1	3111
NM_024181	Dnajc10	4054	NM_001177985	Zmym3	5294	NM_001177944	Eda	5088
NM_007928	Mark2	4664	NM_001177986	Zmym3	1964	NM_001177950	Rpgr	3680
NM_001044697	Zfp2	3117	NM_023277	Jam3	1986	NM_001177951	Rpgr	3187
NM_001044698	Zfp2	3113	NM_001177987	Zmym3	1902	NM_001177952	Rpgr	2795
NM_001044699	Zfp2	3073	NM_001047159	Net1	3874	NM_001177953	Rpgr	2711
NM_001044700	Zfp2	3069	NM_001177988	Zmym3	1516	NM_023268	Qsox1	2533
NM_007935	Epc1	3832	NM_024184	Asf1b	1599	NM_007947	Lcn5	699
NM_025818	1200014J11Rik	6284	NM_146787	Olfr920	1960	NM_007950	Ereg	4136
NM_025820	Crnkl1	3111	NM_025851	1700010I14Rik	1878	NM_025828	Lman2	4315
NM_001044740	Slc7a2	7720	NM_025852	Rexo1	5249	NM_024183	Fip1l1	2974
NM_024182	Riok3	3661	NM_025853	Dsn1	2283	NM_001081656	Neurl1b	6195
NM_025821	Carhsp1	2849	NM_025857	Aagab	2600	NM_025837	Mpi	1757
NM_001177950	Rpgr	3680	NM_025860	Ddx18	2249	NM_007963	Mecom	4429
NM_007943	Eps15	5193	NM_025864	Tmem206	4204	NM_025840	Bzw2	1889
NM_001044747	Zfp68	4635	NM_025870	Swsap1	1846	NM_025844	Chordc1	2205
NM_025824	Bzw1	2833	NM_024186	Ssbp2	6566	NM_001177985	Zmym3	5294

NM 001177952	Rpgr	2795	NM 001185173	Zscan4b	1754	NM 001047159	Net1	3874
NM 001045481	Ifi203	3633	NM 008007	Fgf3	1210	NM 001178012	Sfxn3	2875
NM 001177955	Gpm6b	3387	NM 001190179	Nup35	2752	NM 007977	F8	7522
NM 001177956	Gpm6b	4948	NM 008013	Fgl2	3769	NM 007983	Faf1	4452
NM 001177957	Gpm6b	3478	NM 001076676	Usp33	4173	NM 025860	Ddx18	2249
NM 001177958	Gpm6b	3267	NM 008018	Sh3pxd2a	10464	NM 007986	Fap	2704
NM_001177959	Gpm6b	4828	NM_001076681	Lyrm9	5053	NM_025865	2310030G06Rik	1296
NM 001177960	Gpm6b	3358	NM 001076789	Cbx5	8801	NM 007992	Fbln2	4477
NM 001177961	Gpm6b	4493	NM 025897	Rrp8	2856	NM 025872	Goltlb	2796
NM 001177962	Gpm6b	3023	NM 008028	Flot2	2718	NM 001048189	Agbl4	1966
NM 025832	Naa16	3911	NM 001190297	Gpr155	4969	NM 024186	Ssbp2	6566
NM 007955	Ptprv	6080	NM 001077237	BC003331	3159	NM 025879	Trappc13	3563
NM 025833	Baiap2l1	3248	NM 025902	Cisd2	2885	NM 001048208	Med14	6861
NM 024183	Fip1l1	2974	NM 025907	Mettl6	1905	NM 023284	Nuf2	2405
NM 001177967	Lclat1	4455	NM 025909	Oma1	1866	NM 025882	Pole4	1694
NM 001045559	Zfp952	3008	NM 001077398	Ldb2	2661	NM 001048250	Smim15	1864
NM 007958	Smarcad1	5098	NM 023290	Mkrn2	2241	NM 008013	Fgl2	3769
NM 001177968	Lclat1	4444	NM 001190352	Tmem132b	7964	NM 001190187	Nrg3	4009
NM 001081656	Neurl1b	6195	NM 025922	Itpa	1264	NM 001048267	Tnpo1	5385
NM 007964	Evi5	5849	NM 025923	Fancl	1798	NM 001076676	Usp33	4173
NM 025841	Kdelr2	1870	NM 025926	Dnajb4	2548	NM 008018	Sh3pxd2a	10464
NM 025842	Vps28	885	NM 025932	Syap1	2316	NM 001076681	Lyrm9	5053
NM 025844	Chordc1	2205	NM 025934	Riok2	3201	NM 001190188	Nrg3	3964
NM 001099277	Zfp541	4561	NM 001077591	Tsks	1833	NM 001076789	Cbx5	8801
NM 001047159	Net1	3874	NM 001190401	Usp34	12200	NM 001077184	Bsg	1404
NM 025846	Rras2	2289	NM 008060	Ganab	3859	NM 025895	Med28	4568
NM 007970	Ezh1	4211	NM 001077631	Vwa9	2896	NM 001190297	Gpr155	4969
NM 146787	Olfr920	1960	NM 008063	Slc37a4	2109	NM 001190310	Clec4b1	784
NM 025852	Rexo1	5249	NM 008065	Gabpa	5004	NM 008032	Aff2	4486
NM 007977	F8	7522	NM 001190409	Magt1	4524	NM 025904	Yae1d1	3436
NM 025853	Dsn1	2283	NM 001077687	Lrba	8860	NM 008033	Fnta	1881
NM 025857	Aagab	2600	NM 001077688	Lrba	9387	NM 025909	Oma1	1866
NM 001184706	Tfdp2	7383	NM 025951	Pi4k2b	3143	NM 001190325	Igll1	870
NM 001184708	Tfdp2	7109	NM 001077698	Fmnl1	3774	NM 023290	Mkrn2	2241
NM_001184709	Tfdp2	7007	NM_025952	Magt1	4115	NM_025921	2610002M06Rik	5842
NM 001184710	Tfdp2	6883	NM 001080808	Ccdc64	1853	NM 001077410	Gimap8	3656
NM 007986	Fap	2704	NM 008074	Gabrg3	1673	NM 001190352	Tmem132b	7964
NM 001184711	Tfdp2	6990	NM 001077707	Shprh	6966	NM 001190371	Ankrd29	3256
NM_025865	2310030G06Rik	1296	NM_025958	Cand2	5453	NM_025932	Syap1	2316
NM 001048176	Cerkl	1759	NM 008081	B4galnt2	4086	NM 025934	Riok2	3201
NM 025866	Cdca7	2361	NM 001190473	Dapk3	1676	NM 001190401	Usp34	12200
NM 007992	Fbln2	4477	NM 001190474	Dapk3	1728	NM 023292	Pus3	2012
NM 007994	Fbp2	1294	NM 001190483	Pcsk5	6675	NM 001190402	Pkib	5485
NM_025872	Goltlb	2796	NM_025967	D16Ertd472e	5960	NM_008062	G6pdx	2639
NM 001185153	Zpbp	3674	NM 001079686	Syne1	10876	NM 008065	Gabpa	5004
NM 001048204	Zfp455	2712	NM 001079690	Slc12a1	4660	NM 025950	Cdc37l1	2517
NM 001185173	Zscan4b	1754	NM 001079814	Glyr1	3365	NM 025951	Pi4k2b	3143
NM 025879	Trappc13	3563	NM 025975	Dynlt3	2153	NM 001077698	Fmnl1	3774
NM 023284	Nuf2	2405	NM 025977	Ccdc159	1531	NM 001077707	Shprh	6966
NM 001190156	Snx7	2018	NM 008096	Gc	1812	NM 008076	Gabrr2	1835
NM 001048250	Smim15	1864	NM 025982	Tspan31	1543	NM 025965	Ssr1	9306
NM 025886	Rassf7	1530	NM 001079876	Gas2l3	6748	NM 001190483	Pcsk5	6675
NM 001190187	Nrg3	4009	NM 025989	Gp2	1978	NM 001190717	Dbf4	2441
NM 025888	Kctd20	2400	NM 025994	Efhd2	2381	NM 001190718	Dcaf12l1	3498
NM 001076676	Usp33	4173	NM 008116	Ggt1	2022	NM 008096	Gc	1812
NM 008018	Sh3pxd2a	10464	NM 001190911	Kirrel3	3737	NM 001190804	Dnajb11	2004
NM 001076681	Lyrm9	5053	NM 025997	Fam103a1	1423	NM 001190805	Dnajb11	1628
NM 025891	Smarcd3	1743	NM 001190912	Kirrel3	3734	NM 001190817	Dnajc1	5106

NM_001076789	Cbx5	8801	NM_026000	Psmd9	2610	NM_001190846	Kat2b	4100
NM_025893	Zcchc18	2804	NM_008123	Gja8	6989	NM_025985	Ube2g1	3837
NM_001077184	Bsg	1404	NM_008124	Gjb1	1519	NM_001079876	Gas2l3	6748
NM_025895	Med28	4568	NM_001190914	Kirrel3	3802	NM_025989	Gp2	1978
NM_025897	Rrp8	2856	NM_001080381	Fam65b	3434	NM_025991	Kbtbd4	2393
NM_008028	Flot2	2718	NM_001190978	Kif17	3098	NM_026000	Psmd9	2610
NM_025898	Napa	2332	NM_001190984	Lancl1	4243	NM_008123	Gja8	6989
NM_001190302	Gm14326	2744	NM_001080388	Mark2	4502	NM_026004	Nt5c3	1687
NM_025902	Cisd2	2885	NM_001190985	Lancl1	4461	NM_026005	2610301B20Rik	2133
NM_025904	Yae1d1	3436	NM_001080389	Mark2	4529	NM_008132	Glrp1	1957
NM_008033	Fnta	1881	NM_001080390	Mark2	4381	NM_001080548	Usp6nl	4824
NM_025905	Ttc23	2357	NM_001191001	Lins	5228	NM_001191001	Lins	5228
NM_025907	Mettl6	1905	NM_001080708	Fam65c	2935	NM_001191004	Lsm6	3801
NM_001190325	Igll1	870	NM_008136	Gnl1	2744	NM_008135	Slc6a9	3272
NM_001190332	Frmd7	4647	NM_023311	Yipf5	2329	NM_026013	Dram2	2722
NM_025917	Use1	874	NM_001080743	Grk4	3240	NM_001080706	Btaf1	8509
NM_025919	Rpl11	594	NM_001080747	Gtf2i	4394	NM_023311	Yipf5	2329
NM_025921	2610002M06Rik	5842	NM_001080748	Gtf2i	4337	NM_001080742	Vamp5	1538
NM_001190352	Tmem132b	7964	NM_001080749	Gtf2i	4349	NM_026017	Ctdnep1	1615
NM_001080710	Sdr16c6	1181	NM_008150	Gpc4	2325	NM_023314	Eif4e2	3394
NM_001077496	Evi2b	3529	NM_001080781	1700029H14Rik	1432	NM_026028	Ccdc77	2669
NM_001190374	Adamtsl3	7285	NM_001081658	Dytn	2427	NM_001191032	Cyp4t14	508
NM_001190401	Usp34	12200	NM_026034	Armc10	2014	NM_001191044	Ddx43	2209
NM_001077595	Shroom3	6838	NM_008166	Grid1	3445	NM_008159	Gpr33	1403
NM_001077596	Shroom3	6886	NM_001080813	Rab11fip1	7965	NM_001080777	1700029F12Rik	1140
NM_025938	Rpp14	2467	NM_024195	Cyb5r4	2653	NM_001193309	Morc4	4108
NM_024189	Yaf2	2248	NM_001080814	Fat3	18468	NM_008166	Grid1	3445
NM_001190443	Abcf2	2570	NM_023320	Plekho1	1370	NM_001080798	Aff1	8312
NM_025951	Pi4k2b	3143	NM_008168	Grik5	3861	NM_026036	Cmtm6	3352
NM_024191	Arl2bp	2095	NM_001080820	Cass4	3705	NM_001080814	Fat3	18468
NM_001077707	Shprh	6966	NM_026045	Prpf18	3431	NM_008168	Grik5	3861
NM_001190466	Dact1	3761	NM_001080925	Rapgefl1	4163	NM_026040	Srfbp1	1463
NM_025964	Mettl21a	2666	NM_001080929	Cdr2l	3700	NM_026042	Med29	2162
NM_025965	Ssr1	9306	NM_026053	Gemin6	1086	NM_001080820	Cass4	3705
NM_001190483	Pcsk5	6675	NM_001080931	Med13	11787	NM_026046	Zfp329	7933
NM_008087	Gas2	2137	NM_001080934	Slc16a5	3020	NM_001080930	Atxn1l	7526
NM_001190717	Dbf4	2441	NM_001080965	Aurkc	1168	NM_001080931	Med13	11787
NM_001079824	Hnrnph3	2170	NM_001080966	Aurkc	1454	NM_023324	Peli1	3501
NM_025975	Dynlt3	2153	NM_001080977	Rsbn1l	5562	NM_001080948	Larp4	6553
NM_025977	Ccdc159	1531	NM_001080981	Ddx23	2814	NM_001080974	Sri	2399
NM_001190786	Ddx19b	6596	NM_026072	Cwc27	1884	NM_026067	Eri1	5067
NM_025979	Mastl	4652	NM_008205	H2-M9	1029	NM_008194	Gyk	4357
NM_001079844	Gpc6	6780	NM_001081007	Zfp382	2193	NM_001080981	Ddx23	2814
NM_001190817	Dnajc1	5106	NM_026078	Pigc	3301	NM_001080995	Ddias	3450
NM_001190846	Kat2b	4100	NM_001081011	Srgap2	8040	NM_001080999	Trmt2a	3637
NM_025988	Acbd4	2081	NM_001081012	Ccdc171	4525	NM_001081000	Trmt2a	3684
NM_025994	Efhd2	2381	NM_001081013	Rlf	6585	NM_008206	H2-Oa	1064
NM_025997	Fam103a1	1423	NM_001081016	Zc3h7b	5958	NM_026075	Sreklip1	1715
NM_008120	Gja4	1686	NM_008217	Has3	5912	NM_008207	H2-T24	2182
NM_001080388	Mark2	4502	NM_001081022	D430042O09Rik	6191	NM_001081007	Zfp382	2193
NM_001080389	Mark2	4529	NM_001081025	Maats1	2765	NM_001081009	Parp8	3088
NM_001080390	Mark2	4381	NM_001081027	Kcnt2	3957	NM_001081011	Srgap2	8040
NM_001191001	Lins	5228	NM_024199	Cstf1	1820	NM_001081012	Ccdc171	4525
NM_001191004	Lsm6	3801	NM_026101	Herc4	4019	NM_026083	Zc3h13	6151
NM_008136	Gnl1	2744	NM_001081037	Srgap1	7787	NM_001081016	Zc3h7b	5958
NM_001191012	G6b	2382	NM_001195031	Pag1	8256	NM_001081017	Unc79	8870
NM_023311	Yipf5	2329	NM_001081038	Btbd16	3459	NM_001081021	Zfp780b	5511

NM_008146	Golga3	4833	NM_026104	Tmco5	1187	NM_026086	Nanp	1392
NM_001191051	Lysmd4	3311	NM_001081039	Dock9	7910	NM_001193659	Cldn12	3804
NM_026032	Lsm1	2360	NM_001081041	Vps51	2631	NM_001193660	Cldn12	3752
NM_008156	Gpld1	4659	NM_001195036	Ect2l	2900	NM_001193661	Cldn12	3639
NM_026033	Gatad1	2547	NM_008240	Foxj1	2599	NM_026087	Ceacam12	1056
NM_001080798	Aff1	8312	NM_008243	Mst1	2286	NM_001081024	Setdb2	3034
NM_001080814	Fat3	18468	NM_026110	Paxbp1	3809	NM_026090	Iqcf4	863
NM_026037	Mboat2	2802	NM_001195065	Phactr2	8540	NM_008222	Hccs	2383
NM_001080818	Cdc14a	4511	NM_001195066	Phactr2	8475	NM_001081027	Kcnt2	3957
NM_001080819	Arid1a	8175	NM_026116	Bbs2	2972	NM_026092	Lyzl1	850
NM_001080944	Atp8b4	5529	NM_001081051	D130043K22Rik	4977	NM_001194940	Dlc1	7636
NM_026045	Prpf18	3431	NM_001195086	Ppfia1	5259	NM_001081032	Gm8909	1357
NM_026046	Zfp329	7933	NM_026119	Med4	1304	NM_001194941	Dlc1	6266
NM_026047	Rnf219	3456	NM_001081053	Itga10	5044	NM_026100	Tctex1d1	2029
NM_023323	Rpf2	1480	NM_001195094	Ccdc42b	921	NM_001081035	Nav3	7782
NM_001080930	Atxn1l	7526	NM_026121	Bag4	4798	NM_001195025	Nuak2	3132
NM_023324	Peli1	3501	NM_001195096	Phactr2	8296	NM_001081037	Srgap1	7787
NM_001080948	Larp4	6553	NM_001081057	Tecpr2	7830	NM_026103	Vsig1	2482
NM_008189	Guca1a	917	NM_001195097	3425401B19Rik	5553	NM_001195033	Abhd12b	1080
NM_001080977	Rsbn1l	5562	NM_001081061	Bdp1	9921	NM_001195036	Ect2l	2900
NM_026067	Eri1	5067	NM_001081064	Pdzd2	13413	NM_026106	Dr1	3080
NM_008194	Gyk	4357	NM_026130	Srpr	2934	NM_001081045	Kansl1	5510
NM_001080999	Trmt2a	3637	NM_001195258	Tgfbr3l	1075	NM_026111	Qpctl	2100
NM_001081000	Trmt2a	3684	NM_001081068	Ltn1	7737	NM_008246	Hiat1	2795
NM_008208	H2-T3	2178	NM_001081073	Cep76	3979	NM_001195075	Klhl3	2142
NM_026078	Pigc	3301	NM_001195284	Fsd1l	7491	NM_026116	Bbs2	2972
NM_001081009	Parp8	3088	NM_001081074	A1cf	3828	NM_001081058	Cdk13	7081
NM_001081011	Srgap2	8040	NM_001081076	Gucy2g	3889	NM_026123	Unc50	1412
NM_026083	Zc3h13	6151	NM_001081077	Cwf19l1	3581	NM_001081061	Bdp1	9921
NM_001081016	Zc3h7b	5958	NM_026138	Soga3	3948	NM_001195205	Trmt2a	3520
NM_001081017	Unc79	8870	NM_001081078	Lct	6166	NM_001195229	Nek10	3348
NM_001081021	Zfp780b	5511	NM_026139	Armcx2	3790	NM_001081066	Dennd3	5299
NM_001193659	Cldn12	3804	NM_001195431	Islr	2111	NM_024435	Nts	1237
NM_001193660	Cldn12	3752	NM_026144	Dhdds	3110	NM_001195268	Dos	2978
NM_001193661	Cldn12	3639	NM_001081084	Cubn	11339	NM_146959	Olfr631	1489
NM_001081024	Setdb2	3034	NM_008298	Dnaja1	5583	NM_001081077	Cwf19l1	3581
NM_001081027	Kcnt2	3957	NM_001081101	Uvssa	7343	NM_026139	Armcx2	3790
NM_001081029	Tmem243	988	NM_001081102	Whsc1	6936	NM_001081079	Ogfrl1	4844
NM_001081030	Sbf1	6190	NM_008313	Htr4	4657	NM_001081084	Cubn	11339
NM_001194940	Dlc1	7636	NM_001081109	Lmtk2	8114	NM_001081087	Klhl41	2538
NM_001194941	Dlc1	6266	NM_001081112	Ankrd26	5704	NM_001081092	Taf4a	4422
NM_026097	Rffl	3533	NM_026170	Ergic1	2734	NM_001081093	Arfip1	3023
NM_001081034	Fbxo11	4013	NM_001081120	Fam89a	1315	NM_026150	4921536K21Rik	2618
NM_026101	Herc4	4019	NM_008328	Ifi203	3619	NM_008309	Htr1d	2921
NM_001195025	Nuak2	3132	NM_001195687	Gm4975	3739	NM_008310	Htr1f	2509
NM_001081037	Srgap1	7787	NM_001195693	Plscr5	1117	NM_008314	Htr5a	5576
NM_026104	Tmco5	1187	NM_026174	Entpd4	3084	NM_023343	Ilkap	1368
NM_001081039	Dock9	7910	NM_001081127	Adamts14	5188	NM_008315	Htr7	3095
NM_001195036	Ect2l	2900	NM_026177	Gpalpp1	5448	NM_008316	Hus1	4581
NM_001081043	Ptpn23	5384	NM_001081128	Mtr	4487	NM_001081109	Lmtk2	8114
NM_008243	Mst1	2286	NM_001081129	Cntnap3	4868	NM_001081113	Ipo8	5359
NM_008246	Hiat1	2795	NM_026178	Mmd	2780	NM_001081114	Clip3	3300
NM_001195065	Phactr2	8540	NM_001081130	Ogdhl	3449	NM_001081116	Arhgef17	10250
NM_001195066	Phactr2	8475	NM_001195774	Gprc5b	2558	NM_026169	Frmd8	3166
NM_023333	2210010C04Rik	2142	NM_001081133	Kif16b	5446	NM_001081119	Abhd13	5074
NM_001195083	Phc2	2549	NM_001081135	Prrg3	5898	NM_008327	Ifi202b	1796
NM_026116	Bbs2	2972	NM_023348	Snap29	3432	NM_026173	Poc5	3033
NM_001195086	Ppfia1	5259	NM_001081140	Kcna10	1826	NM_001195727	Gm5901	1002

NM_001081056	Xpot	5989	NM_026188	1700028P14Rik	868	NM_026176	Pdcl	2859
NM 026121	Bag4	4798	NM 001081148	Cyp2b23	2182	NM 001081128	Mtr	4487
NM 001195096	Phactr2	8296	NM 026198	Tmem167b	2864	NM 001081129	Cntnap3	4868
NM 001081057	Tecpr2	7830	NM 026200	Kcnv1	4496	NM 026178	Mmd	2780
NM_001195097	3425401B19Rik	5553	NM_001081160	Mdga1	7566	NM_001081130	Ogdhl	3449
NM 001081058	Cdk13	7081	NM 001081162	Slc4a11	3201	NM 001081133	Kif16b	5446
NM 001195130	Phc2	3949	NM 001081163	Chsy1	4176	NM 001081135	Prrg3	5898
NM 001081061	Bdp1	9921	NM 001081164	Otud4	7324	NM 001081137	Sis	5887
NM 001195205	Trmt2a	3520	NM 001198587	Nrxn3	7933	NM 001081139	Ankrd35	4054
NM 026126	Fundc2	3261	NM 001081167	B3gnt6	2367	NM 026186	Cwc25	3326
NM 001081064	Pdzd2	13413	NM 033560	Vps37a	6379	NM 026191	Dhx40	3724
NM 026130	Srpr	2934	NM 026213	Ttc33	1832	NM 001081148	Cyp2b23	2182
NM 026131	Pdlim7	912	NM 026218	Fgfr1op2	2828	NM 008354	Il12rb2	2947
NM 001081068	Ltn1	7737	NM 001081173	Lrch2	3616	NM 001081151	Gan	2652
NM 001081070	Pdia2	1721	NM 008379	Kpnb1	5909	NM 001081152	Npat	7347
NM 001081071	Lclat1	4476	NM 026220	Mfap1a	3419	NM 001081153	Unc13c	8081
NM 001195284	Fsd1l	7491	NM 026221	Ppfibp1	4860	NM 001081154	Marf1	7765
NM 001081074	A1cf	3828	NM 001081176	Polr3g	3060	NM 026200	Kcnv1	4496
NM 001081080	Phf3	7561	NM 001081179	Heatr5b	6437	NM 001198570	Abi2	4821
NM 026141	Ppil4	2910	NM 001081182	Atp8b2	5459	NM 001198571	Abi2	5892
NM 001081083	Armc3	3004	NM 001081184	Focad	5796	NM 001081160	Mdga1	7566
NM 001195431	Islr	2111	NM 001081185	Flnc	9166	NM 001198573	Usp40	5316
NM 001081084	Cubn	11339	NM 001081186	Tmem52b	2875	NM 001081161	Fam171a1	4146
NM 001081086	Ppig	6297	NM 001081193	Lemd3	4754	NM 001081164	Otud4	7324
NM 001195485	Srsf7	2292	NM 026240	Gramd3	2611	NM 001198587	Nrxn3	7933
NM 001081087	Klhl41	2538	NM 001081194	Kcnh4	3784	NM 001081169	Aspg	2732
NM 001195486	Srsf7	2268	NM 026243	Mgat4c	3757	NM 026214	Kctd4	3015
NM 001195487	Srsf7	2256	NM 001081198	Tmem182	1096	NM 008379	Kpnb1	5909
NM 008297	Hsf2	2623	NM 001081200	Crnn	1628	NM 026221	Ppfibp1	4860
NM 008298	Dnaja1	5583	NM 008405	Itgb2l	2618	NM 026223	Glipr1l2	1879
NM 001081093	Arfip1	3023	NM 008409	Itm2a	1632	NM 026225	Cog6	2920
NM_001081095	1700009N14Rik	1264	NM_008410	Itm2b	1773	NM_024203	Fam120b	4540
NM 001081097	Grik3	8860	NM 001081208	Hs3st5	2958	NM 001081179	Heatr5b	6437
NM 001081100	Morn1	1661	NM 026259	Rnf41	3341	NM 001081180	Spink5	4765
NM 001081101	Uvssa	7343	NM 001081213	Ermp1	5394	NM 008388	Eif3e	1541
NM 001081102	Whsc1	6936	NM 021275	Kcna4	4844	NM 008389	Ipp	2118
NM 008314	Htr5a	5576	NM 001081214	Pprc1	5244	NM 001081182	Atp8b2	5459
NM_001081109	Lmtk2	8114	NM_001081215	Ddx60	5989	NM_026233	4933434I20Rik	1292
NM 026163	Pkp2	2891	NM 008421	Kcnc1	7788	NM 008391	Irf2	2484
NM 026165	Slc25a46	4371	NM 001081217	Zfp174	5942	NM 024204	Ankrd22	1754
NM 026166	Ikbip	3251	NM 008423	Kcnd1	1956	NM 001081190	Gabrr3	1456
NM 001195633	Epg5	9672	NM 001081219	Myo1a	3353	NM 001081191	Eml5	10136
NM 001081116	Arhgef17	10250	NM 001081220	Gpr179	9276	NM 026240	Gramd3	2611
NM 026168	Ergic2	3935	NM 001081221	Ercc6	8422	NM 026242	Mrfap1	1630
NM 008328	Ifi203	3619	NM 001081224	Prr16	2224	NM 001081195	Arid4a	5780
NM 001195687	Gm4975	3739	NM 026272	Narf	4426	NM 026244	Slc39a9	5247
NM_026173	Poc5	3033	NM_001081227	6330403A02Rik	6557	NM_008403	Itgb1bp1	1874
NM 026176	Pdcl	2859	NM 008438	Kera	1950	NM 026247	Alg13	1640
NM 001081130	Ogdhl	3449	NM 008440	Kif1a	8257	NM 026250	Gin1	2041
NM 001195774	Gprc5b	2558	NM 001081229	Tsc22d2	8829	NM 008407	Itih3	2832
NM 001081132	Upf2	5174	NM 008442	Kif2a	4250	NM 008408	Stt3a	2697
NM 001081135	Prrg3	5898	NM 008444	Kif3b	5647	NM 008409	Itm2a	1632
NM 001081140	Kcna10	1826	NM 001198968	Itsn2	6082	NM 026254	Tbc1d23	3664
NM 026186	Cwc25	3326	NM 008447	Kif5a	4304	NM 001081206	Dgki	4598
NM 001081148	Cyp2b23	2182	NM 001081239	Lilra5	2109	NM 026256	Poteg	1440
NM 026193	Ap4b1	2914	NM 026298	Ift172	5440	NM 021275	Kcna4	4844
NM 008354	Il12rb2	2947	NM 001081241	Fam65a	4152	NM 008421	Kcnc1	7788

NM 001081150	Lonrf1	3930	NM 008449	Kif5c	6867	NM 008423	Kcnd1	1956
NM 001081151	Gan	2652	NM 026303	Alkbh8	2300	NM 001081222	Esco1	4540
NM 026195	Atic	2611	NM 008451	Klc2	3032	NM 001081224	Prr16	2224
NM 001081152	Npat	7347	NM 001081243	Filip1	4207	NM 001081225	Fam178a	6880
NM 001081153	Unc13c	8081	NM 001199003	Rgs7	2502	NM 147049	Olfr658	2912
NM_001081154	Marf1	7765	NM_001081246	1700017N19Rik	1690	NM_026276	Aasdhppt	2870
NM 026200	Kcnv1	4496	NM 001199009	Dcaf11	2610	NM 026278	Lrp2bp	3420
NM 001198570	Abi2	4821	NM 008457	Klk1b8	848	NM 026281	Tm7sf3	3312
NM 001198571	Abi2	5892	NM 001081254	Fam186b	3187	NM 008440	Kif1a	8257
NM_001081160	Mdga1	7566	NM_026315	1700011L22Rik	978	NM_001081229	Tsc22d2	8829
NM 026209	Saysd1	2314	NM 008465	Kpna1	4022	NM 026283	Samd8	6997
NM 001198587	Nrxn3	7933	NM 147153	Vps39	4395	NM 147093	Olfr558	2923
NM_033560	Vps37a	6379	NM_008467	Kpna4	3726	NM_001081232	D5Ertd579e	6808
NM 026213	Ttc33	1832	NM 001081268	Prss53	2531	NM 001081235	Mn1	6128
NM 008374	Il9r	3083	NM 008468	Kpna6	5711	NM 008445	Kif3c	6849
NM 001081173	Lrch2	3616	NM 026321	Fam174a	2107	NM 001081237	Klhl42	6571
NM 001198785	Aatk	5423	NM 001199060	Wdr12	3124	NM 026298	Ift172	5440
NM 001198787	Aatk	5195	NM 008470	Krt16	1580	NM 001081247	Polr3a	4683
NM 008379	Kpnb1	5909	NM 001199061	Wdr12	2856	NM 001199009	Dcaf11	2610
NM_026221	Ppfibp1	4860	NM_001081273	1600015I10Rik	2493	NM_001081251	Pbrm1	8117
NM 008382	Inhbe	2135	NM 026324	Kirrel3	3701	NM 001081253	Fbxo43	4035
NM 026223	Glipr1l2	1879	NM 001081276	Clasp1	7827	NM 026312	Pbdc1	2646
NM 026229	Gpr89	1765	NM 026329	Polr2g	868	NM 001081263	Slc44a5	4098
NM_001081181	9430016H08Rik	1325	NM_001081281	Trim55	1787	NM_001081264	Alg6	3114
NM 008389	Ipp	2118	NM 001081282	Ibtk	5679	NM 001081265	Heatr2	3445
NM_001081182	Atp8b2	5459	NM_001199105	Trp53inp1	5430	NM_008466	Kpna3	4167
NM 008392	Irg1	2588	NM 147220	Abca9	6268	NM 001081267	Rsf1	11129
NM 001081185	Flnc	9166	NM 026334	Lipf	1353	NM 001199060	Wdr12	3124
NM 001081186	Tmem52b	2875	NM 001081286	Fat1	14627	NM 001199061	Wdr12	2856
NM 001081191	Eml5	10136	NM 008485	Lamc2	5182	NM 008474	Krt84	2570
NM 001198872	Dync1i2	2674	NM 001199122	Ghitm	2953	NM 001081276	Clasp1	7827
NM 001198873	Dync1i2	2659	NM 001081287	Mpp7	4975	NM 008476	Krt6a	2287
NM 001198874	Dync1i2	2605	NM 001081288	Taf2	5033	NM 147218	Abca6	5240
NM 026244	Slc39a9	5247	NM 026337	Sltm	3569	NM 001081281	Trim55	1787
NM 001198875	Dync1i2	2563	NM 026341	Nudt13	2523	NM 001199105	Trp53inp1	5430
NM 001198876	Dync1i2	2545	NM 001081291	Ccdc88b	4959	NM 147220	Abca9	6268
NM 008403	Itgb1bp1	1874	NM 001199141	Zmynd11	4132	NM 147219	Abca5	8339
NM 001198877	Dync1i2	2663	NM 001081293	Rprd2	8419	NM 001199118	Lpin3	3396
NM_026248	4930430A15Rik	1838	NM_152800	Tor2a	1550	NM_008485	Lamc2	5182
NM 001198878	Dync1i2	2648	NM 001081298	Lphn2	6052	NM 001199122	Ghitm	2953
NM 026250	Gin1	2041	NM 026348	Itgb3bp	2738	NM 001081287	Mpp7	4975
NM 001081203	Sbno1	10071	NM 001199151	Sncaip	3624	NM 001081291	Ccdc88b	4959
NM 001081213	Ermp1	5394	NM 001199153	Sncaip	3529	NM 026343	Stx17	5777
NM 021275	Kcna4	4844	NM 001199154	Sncaip	3728	NM 001081293	Rprd2	8419
NM 001081215	Ddx60	5989	NM 026353	Slc48a1	2526	NM 001081295	Arhgef26	5205
NM 001081217	Zfp174	5942	NM 148933	Slco4a1	3100	NM 148929	Slc9a8	4510
NM 008424	Kcne1	3155	NM 001081302	Trio	11521	NM 001081299	Cdh18	2744
NM 001081218	Hcfc2	2386	NM 008503	Rps2	1005	NM 001199151	Sncaip	3624
NM 001081220	Gpr179	9276	NM 001081310	Tmem236	3658	NM 148930	Rbm5	3104
NM 001081221	Ercc6	8422	NM 026361	Pkp4	4642	NM 001199153	Sncaip	3529
NM 001081222	Esco1	4540	NM 153057	Nomo1	4277	NM 001199154	Sncaip	3728
NM 001081223	Rbbp8	3618	NM 026362	Plgrkt	843	NM 153055	Sec63	6058
NM 008429	Kcnj9	3270	NM 001081316	Dsel	5763	NM 153056	Sirt7	1715
NM_001081224	Prr16	2224	NM_001081319	C030046E11Rik	6282	NM_001081310	Tmem236	3658

NM 026272	Narf	4426	NM 026368	Caap1	2109	NM 001081315	Brpf3	5973
NM 147049	Olfr658	2912	NM 026369	Arpc5	1897	NM 026366	N6amt1	1791
NM 008440	Kif1a	8257	NM 153059	Tmem5	1393	NM 001081318	Gm6614	1953
NM 001081229	Tsc22d2	8829	NM 001081322	Myo5c	6733	NM 026367	Gpatch2	4565
NM 026283	Samd8	6997	NM 026371	Loh12cr1	1777	NM 148413	Myo3a	5082
NM 008442	Kif2a	4250	NM 001081323	Mphosph9	7760	NM 001081323	Mphosph9	7760
NM 001198955	Gm7694	3833	NM 023386	Rtp4	1573	NM 001081326	Agl	9625
NM_001081232	D5Ertd579e	6808	NM_001081326	Agl	9625	NM_001081327	Hs3st2	2270
NM_026291	4930522H14Rik	859	NM_001199234	Sptbn4	4740	NM_001081329	Zkscan2	4584
NM 001198968	Itsn2	6082	NM 001199236	Sptbn4	4724	NM 026380	Rgs8	5827
NM_008445	Kif3c	6849	NM_001081328	Chsy3	3882	NM_001081331	D7Ertd443e	2425
NM 001198969	Itsn2	2350	NM 001081329	Zkscan2	4584	NM 026382	Snrnp48	1789
NM 008446	Kif4	4712	NM 026380	Rgs8	5827	NM 153068	Ehd2	3058
NM_008447	Kif5a	4304	NM_001081331	D7Ertd443e	2425	NM_026384	Dgat2	2251
NM_026296	4930548H24Rik	1427	NM_026384	Dgat2	2251	NM_001081337	Sipa1l2	6365
NM 026298	Ift172	5440	NM 148940	Prss44	1854	NM 148937	Plcd4	2981
NM 001081241	Fam65a	4152	NM 001081341	Scaper	5259	NM 026386	Snx2	2013
NM 026302	Dctn4	3714	NM 008530	Ly6f	877	NM 024205	Jkamp	1976
NM 001081243	Filip1	4207	NM 001081342	Gpr133	5163	NM 001081341	Scaper	5259
NM 001199003	Rgs7	2502	NM 026390	Ubxn4	4093	NM 148942	Serpinb6c	1390
NM_001081247	Polr3a	4683	NM_148942	Serpinb6c	1390	NM_001081344	Stxbp5	9074
NM 001081250	Myh13	5817	NM 001081344	Stxbp5	9074	NM 148944	Chrnb4	3486
NM 001081251	Pbrm1	8117	NM 001081345	Chd2	9071	NM 008534	Ly9	2499
NM 001081253	Fbxo43	4035	NM 001081346	Rtkn2	3932	NM 001081345	Chd2	9071
NM 008466	Kpna3	4167	NM 001081348	Hecw1	9460	NM 001081346	Rtkn2	3932
NM_147153	Vps39	4395	NM_001081353	2210408I21Rik	1749	NM_001199272	Gopc	4319
NM 001081267	Rsf1	11129	NM 001199275	Tnip1	2996	NM 001081354	Mamld1	3457
NM 008467	Kpna4	3726	NM 001199276	Tnip1	2666	NM 026396	Brix1	2654
NM 008468	Kpna6	5711	NM 026399	Wdr83	1238	NM 026399	Wdr83	1238
NM 026321	Fam174a	2107	NM 001199283	Slc43a2	7170	NM 026400	Dnajb11	2534
NM 001199060	Wdr12	3124	NM 001199284	Slc43a2	6952	NM 001081355	Prdm2	7323
NM 001199061	Wdr12	2856	NM 001199293	Tex14	4795	NM 026402	Atg3	2013
NM 008474	Krt84	2570	NM 008547	Mak	3385	NM 008548	Man1a	3889
NM 001081276	Clasp1	7827	NM 026404	Slc35a4	2861	NM 001199301	Cacng5	3771
NM 147218	Abca6	5240	NM 001081359	Ubr5	9791	NM 008549	Man2a1	6091
NM 026327	Pcnx14	4420	NM 026408	Sncaip	3553	NM 148950	Pknox2	3445
NM_001199090	4930522H14Rik	882	NM_001081362	Trrap	12476	NM_001081359	Ubr5	9791
NM 001081282	Ibtk	5679	NM 001081363	Cenpf	11130	NM 026407	Tmem39a	2869
NM 147222	Rdh19	1889	NM 001199310	Lig1	3194	NM 026408	Sncaip	3553
NM_001199105	Trp53inp1	5430	NM_001081371	Dmxl1	12211	NM_001081363	Cenpf	11130
NM_023377	Stard5	2500	NM_008564	Mcm2	3381	NM_001199306	2810007J24Rik	2097
NM 147220	Abca9	6268	NM 001081373	Cep164	5504	NM 008558	Max	2005
NM_026333	2010106E10Rik	1257	NM_153082	Dnajc27	4713	NM_024209	Ppp6c	1583
NM 147219	Abca5	8339	NM 026418	Rgs10	875	NM 153078	Ehbp1	4978
NM 008484	Lamb3	4055	NM 008566	Mcm5	3422	NM 001081371	Dmxl1	12211
NM 001081286	Fat1	14627	NM 153085	Wac	5217	NM 001081372	Ces1b	1965
NM 008485	Lamc2	5182	NM 001199349	Cd99l2	3617	NM 026417	Yipf4	2133
NM 001199122	Ghitm	2953	NM 001199351	Pnck	1642	NM 026418	Rgs10	875
NM 001081287	Mpp7	4975	NM 001199352	Pnck	1506	NM 008566	Mcm5	3422
NM 001081288	Taf2	5033	NM 001199360	Tmem164	5373	NM 008568	Mcm7	2396
NM 001199141	Zmynd11	4132	NM 026423	Mri1	2149	NM 153086	Gjd4	2661

NM 001081293	Rprd2	8419	NM 153089	Ppp1r16b	6370	NM 153089	Ppp1r16b	6370
NM 026344	Dph2	2526	NM 153090	Fcrl1	2042	NM 153091	St7l	5739
NM_001081295	Arhgef26	5205	NM_001199484	4931406C07Rik	2757	NM_023397	Mdp1	1733
NM_026348	Itgb3bp	2738	NM_001199485	4931406C07Rik	2653	NM_026429	Tpbpb	736
NM 001081299	Cdh18	2744	NM 023397	Mdp1	1733	NM 001199556	AW551984	4303
NM_001199151	Sncaip	3624	NM_026431	1810043G02Rik	1696	NM_001199568	Xylb	3908
NM 001199153	Sncaip	3529	NM 001199593	Pkn1	3325	NM 001081377	Pcdh9	5682
NM 001199154	Sncaip	3728	NM 001081377	Pcdh9	5682	NM 153098	Cd109	5883
NM 148933	Slco4a1	3100	NM 153098	Cd109	5883	NM 026433	Tmem100	1764
NM 001081302	Trio	11521	NM 153099	Prss42	1595	NM 001199632	Fbxl13	2655
NM_153055	Sec63	6058	NM_001081378	Kidins220	7402	NM_001081382	Zfp777	3111
NM_026359	4930578I06Rik	1066	NM_026434	Rbm18	2563	NM_026440	Rnmt	5018
NM 153056	Sirt7	1715	NM 026438	Ppa1	1292	NM 153103	Kif1c	6764
NM 026361	Pkp4	4642	NM 001199695	Txlna	4586	NM 001081386	Cdh19	3054
NM 023383	Aadac	1294	NM 153102	Zfp352	2261	NM 001199736	Topaz1	4969
NM 001081316	Dsel	5763	NM 026440	Rnmt	5018	NM 153105	Cldn19	4236
NM 026367	Gpatch2	4565	NM 001081386	Cdh19	3054	NM 153106	Padi6	2333
NM 148413	Myo3a	5082	NM 153105	Cldn19	4236	NM 001199785	Zfp839	3502
NM 153063	Zfp472	2337	NM 153106	Padi6	2333	NM 001081388	Rimbp2	4264
NM 001081328	Chsy3	3882	NM 008596	Sypl2	3247	NM 153107	Cpz	2189
NM 026377	Sfr1	1583	NM 026447	Ppm1m	1844	NM 008599	Cxcl9	2905
NM 026382	Snrnp48	1789	NM 153112	Cadm4	2155	NM 026447	Ppm1m	1844
NM 001081333	Plekhg4	4167	NM 008602	Pias2	4968	NM 008602	Pias2	4968
NM 008528	Blnk	2097	NM 001081392	Mdn1	17959	NM 026448	Klhl7	3427
NM 001081337	Sipa1l2	6365	NM 001081393	Armc4	3477	NM 001081392	Mdn1	17959
NM_001081341	Scaper	5259	NM_001199941	D7Ertd443e	2559	NM_026450	Zfp169	4202
NM_026390	Ubxn4	4093	NM_008606	Mmp11	2247	NM_001199941	D7Ertd443e	2559
NM 026391	Ppp2r2d	2079	NM 001199945	Serpine3	1402	NM 001081396	Tbc1d31	3318
NM 001081344	Stxbp5	9074	NM 152229	Nr2e1	3233	NM 026455	Fam32a	1921
NM 001081345	Chd2	9071	NM 001081397	Myo16	6296	NM 152802	Defb12	997
NM 008535	Lyl1	1818	NM 026455	Fam32a	1921	NM 153116	Gtpbp10	2932
NM_024207	Derl1	3145	NM_001081400	1810013L24Rik	4074	NM_026458	Abca14	5384
NM 148946	Slc8a2	4252	NM 026457	Spert	1473	NM 008614	Mobp	3286
NM_001081348	Hecw1	9460	NM_008612	Mnat1	2505	NM_026461	1700129C05Rik	1067
NM 001081352	Ttc37	5381	NM 001081401	Adamts3	7579	NM 001081407	Plb1	4853
NM 026399	Wdr83	1238	NM 026458	Abca14	5384	NM 001081409	Phf20l1	6670
NM 026400	Dnajb11	2534	NM 008613	Mns1	1837	NM 001081411	Sclt1	3020
NM 001199293	Tex14	4795	NM 001081402	Wdr70	2151	NM 001081412	Bcr	6537
NM 001199301	Cacng5	3771	NM 008614	Mobp	3286	NM 008620	Gbp4	4475
NM 026403	Nop9	3828	NM 153119	Plekho2	5127	NM 001199988	Tti2	4978
NM 001081359	Ubr5	9791	NM 153121	Lysmd1	2401	NM 153127	Mmrn2	3935
NM 026406	Rnf115	2265	NM 001081409	Phf20l1	6670	NM 026474	Sugt1	1951
NM 026408	Sncaip	3553	NM 001081411	Sclt1	3020	NM 001081418	Gltscr1	5360
NM 001081363	Cenpf	11130	NM 153124	St8sia5	2006	NM 026476	U2surp	7604
NM 001081364	Arhgap21	6944	NM 153388	Lrfn4	2930	NM 001081421	Galnt16	3920
NM 008558	Max	2005	NM 023403	Mesdc2	4492	NM 001081422	Bod1l	10552
NM_024209	Ppp6c	1583	NM_008620	Gbp4	4475	NM_001200013	1700013H16Rik	1224
NM 153078	Ehbp1	4978	NM 008621	Mpp1	2458	NM 026482	Atp2b1	7130
NM 001081371	Dmxl1	12211	NM 001081413	Unc13b	6374	NM 026484	Ccny	4017
NM 026418	Rgs10	875	NM 026470	Spata6	2454	NM 001081426	Dip2c	8026
NM 153083	Thtpa	2839	NM 153389	Atp10d	6021	NM 001081429	Ccdc15	3525
NM 153085	Wac	5217	NM 153127	Mmrn2	3935	NM 152809	Csnk1g3	4394

NM_026420	Paip2	1424	NM_026474	Sugt1	1951	NM_026492	Ssxb1	875
NM_001199351	Pnck	1642	NM_153129	Pacs1	4361	NM_008634	Map1b	11852
NM_001199352	Pnck	1506	NM_001081419	Dip2a	6370	NM_001081433	Ankrd44	6156
NM_001199360	Tmem164	5373	NM_008623	Mpz	1993	NM_026496	Grhl2	4950
NM_001199432	Dnmt1	5251	NM_152799	Pear1	4466	NM_001081437	Fbln2	4336
NM_153091	St7l	5739	NM_001081422	Bod1l	10552	NM_008642	Mttp	3962
NM_001199433	Dnmt1	5876	NM_001081423	Ttll5	4858	NM_001081441	Wdr86	1805
NM_001199556	AW551984	4303	NM_001200013	1700013H16Rik	1224	NM_008645	Mug1	4657
NM_153097	Trim60	3633	NM_001081426	Dip2c	8026	NM_008646	Mug2	4544
NM_026432	Tmem66	1914	NM_008633	Map4	5673	NM_152815	Lins	5243
NM_153098	Cd109	5883	NM_008634	Map1b	11852	NM_152817	Ttc27	2839
NM_001081378	Kidins220	7402	NM_026496	Grhl2	4950	NM_001081453	Nin	9675
NM_026435	Ufm1	4887	NM_152811	Ugt2b1	2560	NM_001081456	Plcd4	2734
NM_001081382	Zfp777	3111	NM_001201330	5730507C01Rik	2542	NM_023420	Col4a3bp	5363
NM_001199695	Txlna	4586	NM_152813	Plcd3	3031	NM_153505	Nckap1l	4717
NM_026439	Ccdc80	3648	NM_152814	Zfp566	1692	NM_001201413	Apbb2	6665
NM_001199736	Topaz1	4969	NM_008646	Mug2	4544	NM_001201414	Apbb2	6668
NM_001081388	Rimbp2	4264	NM_152815	Lins	5243	NM_008664	Myom2	4953
NM_153107	Cpz	2189	NM_001081448	Vmn2r84	2550	NM_001201415	Apbb2	6602
NM_026448	Klhl7	3427	NM_026505	Bambi	5040	NM_001201416	Apbb2	4768
NM_001081392	Mdn1	17959	NM_001081453	Nin	9675	NM_026518	Rnf146	4339
NM_001081393	Armc4	3477	NM_026507	Zwilch	2784	NM_008667	Nab1	5098
NM_001081394	Tmem248	3607	NM_152823	Unc5cl	2838	NM_153135	Unc5d	9220
NM_001199945	Serpine3	1402	NM_001081457	Ppp2r5c	4121	NM_001201569	Atp9b	5477
NM_026453	Mak16	1857	NM_001201395	4930468A15Rik	1003	NM_153139	Slc36a1	5280
NM_001081397	Myo16	6296	NM_153505	Nckap1l	4717	NM_026524	Mid1ip1	2056
NM_026455	Fam32a	1921	NM_001081462	Gtf2ird1	4711	NM_001081475	Nasp	2118
NM_024213	Anapc4	2668	NM_001081463	Gtf2ird1	4630	NM_001081477	Brwd3	6434
NM_153115	Spag11a	435	NM_026513	Pdf	1547	NM_008676	Nbr1	4682
NM_153116	Gtpbp10	2932	NM_008664	Myom2	4953	NM_153145	Abca8a	5629
NM_001081401	Adamts3	7579	NM_008666	Myt1l	7192	NM_023423	Akirin1	2383
NM_001081403	Klhl14	4018	NM_001081467	Gtf2ird1	4402	NM_001081557	Camta1	8448
NM_153119	Plekho2	5127	NM_153134	Irgq	6159	NM_001204165	Rapgef4	4220
NM_008616	Zfp239	2648	NM_008667	Nab1	5098	NM_001204166	Rapgef4	4112
NM_001081407	Plb1	4853	NM_153135	Unc5d	9220	NM_153157	Olfm3	4436
NM_001081409	Phf20l1	6670	NM_153391	Wdr19	4403	NM_001204223	Clec12b	1609
NM_001081411	Sclt1	3020	NM_026521	Zfp706	4485	NM_008688	Nfic	6295
NM_153127	Mmrn2	3935	NM_153136	Nudt18	3716	NM_001204241	Clec4a3	1192
NM_001081416	Fndc1	5846	NM_001201569	Atp9b	5477	NM_153525	Tmem41b	3619
NM_153128	Klhl12	3196	NM_026522	Chid1	3981	NM_008694	Ngp	1176
NM_153129	Pacs1	4361	NM_153140	Rab11fip3	3271	NM_001099276	Pik3c2b	7929
NM_026476	U2surp	7604	NM_001081477	Brwd3	6434	NM_153167	Dcaf10	7226
NM_001081419	Dip2a	6370	NM_001081490	Fbxo9	1867	NM_001081668	Nup62cl	1144
NM_153390	Pxt1	1015	NM_153514	Rhobtb2	5231	NM_008702	Nlk	4507
NM_152799	Pear1	4466	NM_008682	Nedd1	3542	NM_001204335	Cyp4f14	2225
NM_026480	Ooep	1207	NM_001081557	Camta1	8448	NM_001204336	Cyp4f14	2205
NM_001081426	Dip2c	8026	NM_153153	Svil	7424	NM_001204340	Gucy1b2	2672
NM_026485	Trabd	2393	NM_026539	Chdl1	2989	NM_023431	Mum1	3717
NM_026487	Atad1	2813	NM_001204167	Rapgef4	3891	NM_001204371	Oprk1	4707
NM_152809	Csnk1g3	4394	NM_001081566	Pik3r6	3237	NM_026576	Etaa1	4192
NM_001081431	Zscan25	1996	NM_153392	Ttc39a	2343	NM_001204904	4930558K02Rik	980
NM_026495	Nacc2	6370	NM_008688	Nfic	6295	NM_153171	Rgs13	1497
NM_008640	Laptm4a	2130	NM_008690	Nfkbie	2422	NM_001204906	Recql	3067
NM_152811	Ugt2b1	2560	NM_001081650	Rgs3	4001	NM_026580	Otub2	2813
NM_001201323	5430421N21Rik	1775	NM_026552	Arpc4	2271	NM_152839	Igj	2111

NM_026502	1110004E09Rik	1163	NM_001204273	Lsm2	901	NM_026585	Fam21	4305
NM_152815	Lins	5243	NM_001081665	Ccdc129	3284	NM_153395	Mon2	9279
NM_001081448	Vmn2r84	2550	NM_008697	Nin	6818	NM_001204915	Reep3	5550
NM_001081453	Nin	9675	NM_001099276	Pik3c2b	7929	NM_001081756	Nckap5	7246
NM_001081458	Ppp2r5c	2383	NM_153166	Cpne5	4366	NM_153399	Syne1	6982
NM_023420	Col4a3bp	5363	NM_153167	Dcaf10	7226	NM_026597	Fam212a	1077
NM_001201413	Apbb2	6665	NM_001081668	Nup62cl	1144	NM_008728	Npr3	6927
NM_001201414	Apbb2	6668	NM_023431	Mum1	3717	NM_026598	Ebpl	2077
NM_008664	Myom2	4953	NM_001204371	Oprk1	4707	NM_001081961	2300005B03Rik	464
NM_001201415	Apbb2	6602	NM_026576	Etaa1	4192	NM_008729	Ctnnd2	5959
NM_001201416	Apbb2	4768	NM_001081684	Zbtb21	5868	NM_008730	Nptx1	5284
NM_153133	Rdh9	2698	NM_001081685	Zbtb21	5411	NM_001081963	9430020K01Rik	6748
NM_008666	Myt1l	7192	NM_153171	Rgs13	1497	NM_152915	Dner	3664
NM_153134	Irgq	6159	NM_001204906	Recql	3067	NM_008731	Npy2r	3361
NM_026518	Rnf146	4339	NM_001204907	Recql	2163	NM_153177	Ago4	6527
NM_153135	Unc5d	9220	NM_008715	Ints6	5019	NM_008734	Nrg3	4012
NM_153391	Wdr19	4403	NM_026578	Gar1	1271	NM_153405	Rbm45	1904
NM_001081475	Nasp	2118	NM_001204914	Reep2	1920	NM_026617	Tmbim4	808
NM_153140	Rab11fip3	3271	NM_026588	Stx19	1205	NM_153197	Clec4a3	1328
NM_153144	Ggnbp2	2967	NM_001204915	Reep3	5550	NM_153198	Hbp1	2941
NM_153514	Rhobtb2	5231	NM_153399	Syne1	6982	NM_153406	Speccl1	6078
NM_026535	Serpina12	3690	NM_001204979	Sars	3632	NM_001082483	Efr3b	6547
NM_153153	Svil	7424	NM_026599	Cgnl1	6754	NM_153408	Neurl3	2583
NM_026538	Ddx56	3287	NM_026602	Bcas2	1422	NM_026622	3110057012Rik	2492
NM_153155	C1ql3	3163	NM_026603	Denr	2236	NM_001082485	Zfp266	6305
NM_153157	Olfm3	4436	NM_001081963	9430020K01Rik	6748	NM_153384	Clrn1	3083
NM_153158	E130308A19Rik	3237	NM_152915	Dner	3664	NM_153385	Clrn1	3029
NM_153164	Cnot1	8240	NM_008731	Npy2r	3361	NM_153386	Clrn1	2849
NM_001081678	Zfp800	3895	NM_026604	Fam135a	6467	NM_153412	Phldb2	5496
NM_008697	Nin	6818	NM_001205011	Mptx2	694	NM_153414	Ints9	2695
NM_001099276	Pik3c2b	7929	NM_026605	Sympk	4121	NM_153415	Pomt2	5059
NM_153167	Dcaf10	7226	NM_153177	Ago4	6527	NM_008764	Tnfrsf11b	2818
NM_001081668	Nup62cl	1144	NM_026611	Rnaset2b	1031	NM_008765	Orc2	3561
NM_001204340	Gucy1b2	2672	NM_001081977	Rnf144a	5189	NM_026639	Art4	2384
NM_023431	Mum1	3717	NM_008738	Nrtn	1023	NM_001082976	Tc2n	5313
NM_001081671	1700042B14Rik	1084	NM_026616	Rnaseh2c	646	NM_001205067	Jkamp	1958
NM_001114754	Gm6121	893	NM_008739	Nsd1	12784	NM_026643	Cep57l1	2151
NM_001081682	Gnb1l	3625	NM_026619	Gsto2	1305	NM_001083119	Ptpru	5528
NM_026574	Ino80	6296	NM_001082483	Efr3b	6547	NM_008773	P2ry2	3000
NM_153170	Slc36a2	2431	NM_153407	Csrnp2	4126	NM_153512	Kcng3	3356
NM_008715	Ints6	5019	NM_001082484	Snx27	5479	NM_026647	Zdhhc21	8751
NM_008717	Zfml	6385	NM_001205036	LOC100504608	699	NM_001083312	Gbp7	5604
NM_001081750	Zfp664	4139	NM_008752	Nxph2	2579	NM_153421	Phc3	11066
NM_001081756	Nckap5	7246	NM_001205043	Jarid2	5997	NM_026656	Mcoln2	2519
NM_001081956	Akap17b	6153	NM_153412	Phldb2	5496	NM_153422	Pde5a	6728
NM_026593	D730048I06Rik	1334	NM_001205044	Jarid2	5670	NM_001205076	Jph2	4155
NM_153399	Syne1	6982	NM_001205049	Acr	1496	NM_001083319	Ubp1	3857
NM_008728	Npr3	6927	NM_001082573	Crygc	629	NM_153458	Olfm3	4689
NM_026598	Ebpl	2077	NM_026636	Babam1	1592	NM_001083331	Nup88	2485
NM_008729	Ctnnd2	5959	NM_008765	Orc2	3561	NM_026666	Ubn1	6455
NM_026603	Denr	2236	NM_026640	Fam111a	3199	NM_008791	Pcp4	669

NM_001081963	9430020K01Rik	6748	NM_008768	Orm1	768	NM_153527	Dnajb13	1172
NM 152915	Dner	3664	NM 026643	Cep57l1	2151	NM 001083342	Ptchd2	8689
NM 008731	Npy2r	3361	NM 001083119	Ptpru	5528	NM 153528	Gramd1c	3506
NM 026604	Fam135a	6467	NM 001205068	Jmjd4	4409	NM 026669	Tmbim6	2397
NM 026605	Sympk	4121	NM 008776	Pafah1b3	892	NM 026670	Zmym1	3832
NM 153402	Ago3	7635	NM 001083188	Lig1	3186	NM 001083616	Cacna1d	9079
NM 153177	Ago4	6527	NM 153417	Trpm6	6520	NM 001083618	Ttll9	2939
NM 001081977	Rnf144a	5189	NM 153419	Grwd1	1905	NM 153532	Zfp280c	4366
NM 008734	Nrg3	4012	NM 008779	Cntn3	5183	NM 001083628	Greb1l	8421
NM 008739	Nsd1	12784	NM 001083317	Slc35a4	2753	NM 001205095	Gm4944	4880
NM 153198	Hbp1	2941	NM 001205076	Jph2	4155	NM 008801	Pde6d	1109
NM 153406	Speccl1	6078	NM 153424	Nphp4	5047	NM 026679	Tmem254a	2149
NM 001082483	Efr3b	6547	NM 001083321	Magi1	5105	NM 008802	Pde7a	6028
NM 153408	Neurl3	2583	NM 026665	Cep57	2529	NM 153537	Phldb1	5486
NM 001082484	Snx27	5479	NM 153484	Tef	4174	NM 026680	Golt1a	1842
NM 001082485	Zfp266	6305	NM 026666	Ubn1	6455	NM 008804	Pde9a	2064
NM_008752	Nxph2	2579	NM_001083342	Ptchd2	8689	NM_001083890	Spata31d1c	3390
NM 001205043	Jarid2	5997	NM 153528	Gramd1c	3506	NM 008812	Padi2	4758
NM 023438	Tmem132e	4105	NM 026670	Zmym1	3832	NM 153547	Gnl3	1935
NM_001205044	Jarid2	5670	NM_001083616	Cacna1d	9079	NM_153548	E430025E21Rik	4038
NM 026633	Fam195a	816	NM 008795	Cdk18	3061	NM 008820	Pepd	1856
NM 008765	Orc2	3561	NM 153533	Tenc1	4707	NM 001083919	Xirp2	11969
NM 001205057	Gm14295	1324	NM 001083628	Greb1l	8421	NM 001083934	Myom1	5319
NM 026641	Ift80	4090	NM 026675	Nudt22	999	NM 008832	Phka1	6115
NM 001083114	Pphln1	3759	NM 008800	Pde1b	3262	NM 001205173	Iffo2	5732
NM 001083119	Ptpru	5528	NM 001205098	Mgat4c	4279	NM 008843	Pip	571
NM 008775	Pafah1b2	4241	NM 153537	Phldb1	5486	NM 001205204	Astn1	7241
NM 153417	Trpm6	6520	NM 008803	Pde8a	2683	NM 153569	Gak	4798
NM 026647	Zdhhc21	8751	NM 008804	Pde9a	2064	NM 153570	Noc4l	2039
NM 153421	Phc3	11066	NM 026682	Cpsf4l	1234	NM 001085355	Arid1b	11325
NM 153422	Pde5a	6728	NM 001083901	Hiatl1	3375	NM 153572	Katnal1	6173
NM 001083318	Etv3	5143	NM 153542	Lrrc20	2636	NM 153574	Fam13a	4300
NM_024222	Stt3b	4236	NM_153548	E430025E21Rik	4038	NM_001205219	Sorbs2	6094
NM 001083320	Magi1	7181	NM 008820	Pepd	1856	NM 026735	Mob1b	3171
NM 153457	Rtn1	3632	NM 001083919	Xirp2	11969	NM 008850	Pitpna	3724
NM_026662	Prps2	3638	NM_153556	Pms1	3045	NM_026739	9530077C05Rik	2173
NM 153458	Olfm3	4689	NM 001083925	Oas1b	1837	NM 001085373	Mcc	7875
NM 026664	Vps53	2816	NM 153562	Rrnad1	2847	NM 153581	Gpm6a	3351
NM 026665	Cep57	2529	NM 001083935	Pth1r	2236	NM 001085374	Mcc	7843
NM 001083341	Mboat2	2706	NM 153564	Gbp5	3021	NM 172144	Ppp2r3a	4778
NM 026669	Tmbim6	2397	NM 001083936	Pth1r	2269	NM 001085393	Spaca5	554
NM 001083628	Greb1l	8421	NM 008832	Phka1	6115	NM 153585	Cnot10	2990
NM 172049	Tmem18	2971	NM 001083938	Rnaset2a	962	NM 001205241	Kat6b	7223
NM 008800	Pde1b	3262	NM 026718	Ankrd13a	3316	NM 001085412	Gm13078	2110
NM 026679	Tmem254a	2149	NM 001205173	Iffo2	5732	NM 153588	Mkl2	8295
NM 008802	Pde7a	6028	NM 001205204	Astn1	7241	NM 008862	Pkia	3683
NM 153541	Zbtb8b	3288	NM 008845	Pip4k2a	3471	NM 026748	Ints1	7133
NM_026686	0610011F06Rik	825	NM_153570	Noc4l	2039	NM_001085409	Steap3	3142
NM_153548	E430025E21Rik	4038	NM_153571	Hscb	825	NM_153592	Erlin2	3951
NM 001083918	Gm13139	2812	NM 001085355	Arid1b	11325	NM 001085419	Gm13102	2405
NM 153555	Dcaf8	3760	NM 153574	Fam13a	4300	NM 026756	Nfic	6218
NM 153556	Pms1	3045	NM 023464	Ssna1	864	NM 001085448	Ctnnd1	5294
NM 026698	Tmem129	2259	NM 008850	Pitpna	3724	NM 001085449	Ctnnd1	5047
NM 001205132	Yes1	4146	NM 001085370	Speg	9885	NM 001085450	Ctnnd1	5357
NM 001083937	Slc35a2	2426	NM 153578	Nipa1	1885	NM 001085453	Ctnnd1	5046

NM 001205173	Iffo2	5732	NM 001085371	Speg	3410	NM 026765	Uckl1	1811
NM_172146	Ppat	4008	NM_001085385	1600014C10Rik	3357	NM_153600	Ttc26	4162
NM 001085355	Arid1b	11325	NM 001205241	Kat6b	7223	NM 001205286	Tmem39a	2617
NM 153572	Katnal1	6173	NM 026747	Ptx4	1582	NM 001205287	Tmem39a	2665
NM 001085378	Myh7b	6132	NM 001205253	Thsd1	4289	NM 001085499	Gm595	2122
NM 153573	Fkbp14	2714	NM 153589	Ano2	3949	NM 172152	Slc24a4	2092
NM 001205226	Cnot1	8369	NM 026756	Nfic	6218	NM 026775	Tmed10	3526
NM 001085370	Speg	9885	NM 026763	Col6a4	7439	NM 001085507	Zbtb34	6602
NM 153581	Gpm6a	3351	NM 001085472	Acin1	2390	NM 153760	Mill2	1075
NM 001085376	Pappa2	8602	NM 153598	Ugt2b34	3048	NM 153761	Mill2	1120
NM 172144	Ppp2r3a	4778	NM 153599	Cdk8	2586	NM 001085515	AI464131	4192
NM 153588	Mkl2	8295	NM 026770	Cgref1	1350	NM 001085518	Gm14085	3740
NM 153589	Ano2	3949	NM 026772	Cdc42ep2	1491	NM 153777	Lrrc56	2140
NM 001205268	Gm14781	1360	NM 172152	Slc24a4	2092	NM 026785	Ube2c	931
NM 026763	Col6a4	7439	NM 001205330	Map4	5776	NM 001205349	Lipk	2240
NM 153601	Lgsn	1911	NM 001085507	Zbtb34	6602	NM 026788	Mthfd2l	2256
NM 008877	Plg	2755	NM 001205331	Map4	3517	NM 001205361	Dcun1d1	4474
NM 001085499	Gm595	2122	NM 001085508	Tmem8b	4787	NM 001205362	Dcun1d1	4434
NM 172153	Lcorl	4983	NM 026779	Mocos	2884	NM 008894	Pold2	1634
NM 001085503	Aadacl3	3153	NM 001085509	Myom3	5578	NM 026790	Ifi27	1053
NM 008880	Plscr2	1895	NM 170684	Cpne7	2421	NM 026791	Fbxw9	1866
NM 026775	Tmed10	3526	NM 001205332	Map4	5556	NM 153785	Cdkl3	2765
NM 001085507	Zbtb34	6602	NM 001085513	Fam131c	1460	NM 008905	Ppfibp2	3551
NM 001085508	Tmem8b	4787	NM 001085515	AI464131	4192	NM 153794	Fam210a	9669
NM 001085513	Fam131c	1460	NM 153774	Ipo9	6236	NM 001085541	Gm13128	1745
NM 001085516	Gm12794	1452	NM 001205341	Ppfia2	6454	NM 153801	Tecrl	2373
NM 153774	Ipo9	6236	NM 023476	Tinagl1	2095	NM 153803	Glb1l2	3710
NM 001085522	Gm13251	3691	NM 024228	Gdpd3	1081	NM 008915	Ppp3cc	1948
NM 001085529	Slc2a7	1578	NM 026789	Wdr65	3895	NM 026815	Upk1a	1308
NM 001205353	Gramd4	4310	NM 001205361	Dcun1d1	4474	NM 008921	Prim1	1533
NM 001205355	Fhdc1	6001	NM 001205362	Dcun1d1	4434	NM 026819	Dhrs1	1486
NM 026789	Wdr65	3895	NM 153782	Fam20a	2614	NM 172143	Ofcc1	2816
NM 001085530	Gm13298	4130	NM 153784	Ccdc64b	1936	NM 001093759	Trappc13	3560
NM 153781	Pygb	3860	NM 026793	Myct1	2792	NM 001093760	Trappc13	3545
NM 001205369	Casc4	4215	NM 001205369	Casc4	4215	NM 170597	Creg2	5799
NM 001205370	Casc4	4047	NM 001205370	Casc4	4047	NM 008930	Prl7a1	974
NM 153788	Acap1	2482	NM 001205371	Casc4	3805	NM 170598	Rbm12	3720
NM 001205371	Casc4	3805	NM 153794	Fam210a	9669	NM 026830	Rreb1	7437
NM 008905	Ppfibp2	3551	NM 001206335	Itfg3	2863	NM 026832	Cgrrf1	1202
NM 008906	Ctsa	3301	NM 008908	Ppic	1286	NM 008941	Tmprss15	4194
NM 001085542	Gm13124	1221	NM 153798	Polr2b	3812	NM 008942	Npepps	4161
NM_001205396	Gprc5d	1392	NM_008909	Ppl	6277	NM_001242345	B230216G23Rik	1536
NM 008909	Ppl	6277	NM 153801	Tecrl	2373	NM 008950	Psmc5	1277
NM 153800	Arhgap22	2598	NM 153804	Plekhg3	4955	NM 001098168	Asap2	5566
NM 153803	Glb1l2	3710	NM 153805	Pkn3	2948	NM 026854	Dtwd2	3145
NM 153805	Pkn3	2948	NM 026811	Lce1e	729	NM 170758	Cd300a	4642
NM 008916	Inpp5k	2660	NM 026812	Hddc3	860	NM 026860	Gkn3	793
NM 153808	Smc5	5730	NM 153808	Smc5	5730	NM 171826	Cldn25	2194
NM 153820	Arhgap15	2663	NM 001085549	Trabd2b	6617	NM 172053	Adamts16	4986
NM 026819	Dhrs1	1486	NM 001093750	Ptchd1	6489	NM 008958	Ptch2	3549
NM 172134	Pdxk	5110	NM 170598	Rbm12	3720	NM 026868	Abhd13	5181
NM 001093754	Dennd2d	4050	NM 001093775	Myt1l	7198	NM 008961	Pter	3620
NM 001093759	Trappc13	3560	NM 170671	Mycbpap	2944	NM 001242378	Slc4a10	5514
NM 001093760	Trappc13	3545	NM 026827	Tmem219	962	NM 001098476	Grsf1	2425
NM 170756	Spata2	3996	NM 170673	Cpne9	2779	NM 001098669	Klrc2	707
NM_170598	Rbm12	3720	NM_001093776	Myt1l	7104	NM_001242388	9830147E19Rik	5011
NM 001093775	Myt1l	7198	NM 001093778	Myt1l	7189	NM 008976	Ptpn14	10766
NM 026827	Tmem219	962	NM 008942	Npepps	4161	NM 001242411	Srgap1	7718
NM 001093776	Myt1l	7104	NM 001097621	Kif26a	6918	NM 172256	Dync2li1	1378

NM_001093778	Myt1l	7189	NM_026836	Taf1l	1220	NM_001242419	4933426M11Rik	5088
NM_026830	Rreb1	7437	NM_026837	Tmem53	999	NM_008978	Ptpn20	3090
NM_001109969	Gm10058	889	NM_001097644	Ccnyl1	2940	NM_001242558	Ncoa6	3896
NM_001109970	Gm10486	924	NM_001098168	Asap2	5566	NM_008981	Ptprg	9190
NM_026833	Wash	2904	NM_001242360	Trnt1	1916	NM_172262	Kdm1b	4987
NM_026835	Ms4a6d	1401	NM_170758	Cd300a	4642	NM_026907	Sectm1b	2322
NM_026840	Pdgfrl	1545	NM_001242363	Fam160a2	10944	NM_172263	Pde8b	4352
NM_001097977	Gm14151	2672	NM_026856	Zfp644	5700	NM_026908	Cab39l	3037
NM_170727	Scgb3a1	645	NM_001242364	Fam160a2	10895	NM_172380	Poglut1	2641
NM_001097980	Gm16390	1081	NM_170760	U2af1l4	864	NM_008984	Ptprm	5569
NM_026854	Dtwd2	3145	NM_001098225	Adam22	9068	NM_172266	Lpgat1	7048
NM_170757	Ccdc186	2754	NM_001242365	Fam160a2	10301	NM_008988	Igdcc3	3146
NM_170758	Cd300a	4642	NM_171824	Pgbd5	2854	NM_172271	Slc6a17	6326
NM_001242363	Fam160a2	10944	NM_172015	Iars	4396	NM_026917	Zdhhc3	6987
NM_026856	Zfp644	5700	NM_172051	Tmcc3	5528	NM_008991	Abcd3	3399
NM_001242364	Fam160a2	10895	NM_026862	Cd177	2733	NM_172383	Tmem125	1879
NM_170760	U2af1l4	864	NM_001098237	Zbtb3	2037	NM_172274	Cc2d2a	5463
NM_001098225	Adam22	9068	NM_172054	Txndc9	4596	NM_172275	Trafd1	2486
NM_001242365	Fam160a2	10301	NM_026866	Displ	5030	NM_008999	Rab23	4321
NM_171826	Cldn25	2194	NM_001242378	Slc4a10	5514	NM_172280	2210018M11Rik	4031
NM_172015	Iars	4396	NM_026876	Trmt1l	2681	NM_026936	Oxa1l	2534
NM_008961	Pter	3620	NM_001098528	Kcnb2	5309	NM_009009	Rad21	3632
NM_001242378	Slc4a10	5514	NM_172204	Noxa1	1673	NM_009015	Rad54l	3093
NM_026872	Ubap2	4384	NM_172253	Twistnb	2324	NM_173741	Wdr24	3099
NM_172121	Zc3h3	3338	NM_001242388	9830147E19Rik	5011	NM_172302	Cpsf7	3604
NM_172124	B3gat2	4367	NM_001242389	Trip10	2458	NM_172304	Tex10	3203
NM_001242389	Trip10	2458	NM_026888	Phkg2	1644	NM_172310	Tarsl2	3194
NM_001098836	Atxn7l3	3723	NM_001098836	Atxn7l3	3723	NM_173051	Serpinb1c	1599
NM_001242390	Trip10	2455	NM_172255	Wdr11	4566	NM_172393	Aim1	7416
NM_001098837	Atxn7l3	3702	NM_001242390	Trip10	2455	NM_172394	Nup88	2452
NM_001242391	Trip10	2287	NM_001098837	Atxn7l3	3702	NM_001099633	Dnah9	13774
NM_026891	Cdan1	6384	NM_001242391	Trip10	2287	NM_026981	Dtwd1	1360
NM_173731	Hmgcll1	3615	NM_026893	Dcaf12	3454	NM_001099637	Cep170	6930
NM_172378	Slc22a22	2368	NM_008976	Ptpn14	10766	NM_026984	Kmt2e	7258
NM_008976	Ptpn14	10766	NM_001242411	Srgap1	7718	NM_172405	Fam175a	2499
NM_001242411	Srgap1	7718	NM_172256	Dync2li1	1378	NM_001099738	Dnajc28	3535
NM_001242419	4933426M11Rik	5088	NM_001242419	4933426M11Rik	5088	NM_174857	Mamdc2	3338
NM_001099217	Ly6c2	875	NM_008981	Ptprg	9190	NM_027000	Gtpbp4	2579
NM_001099295	1700042G07Rik	419	NM_008982	Ptprj	7634	NM_027001	2610034M16Rik	4192
NM_008981	Ptprg	9190	NM_001242605	Acin1	2454	NM_172413	Rap2c	3562
NM_173363	Eif5	3966	NM_001242606	Acin1	2415	NM_023503	Ing2	2813
NM_008982	Ptprj	7634	NM_172262	Kdm1b	4987	NM_009044	Rel	2584
NM_172262	Kdm1b	4987	NM_172263	Pde8b	4352	NM_172418	Mamstr	2179
NM_172263	Pde8b	4352	NM_008983	Ptprk	6177	NM_001242937	Gm7903	3836
NM_026908	Cab39l	3037	NM_172380	Poglut1	2641	NM_172421	Asxl2	8902
NM_008983	Ptprk	6177	NM_008984	Ptprm	5569	NM_172422	Fastkd2	3205
NM_001242647	Fam187b	1457	NM_172266	Lpgat1	7048	NM_001099785	D3Ertd751e	3457
NM_172380	Poglut1	2641	NM_008985	Ptprn	3536	NM_172426	Slc24a2	10606
NM_172266	Lpgat1	7048	NM_172271	Slc6a17	6326	NM_001100109	Srp54b	3286
NM_172271	Slc6a17	6326	NM_001099307	Gm7173	2832	NM_172440	Stxbp5l	12064
NM_026917	Zdhhc3	6987	NM_172383	Tmem125	1879	NM_001100110	Srp54c	2516
NM_008991	Abcd3	3399	NM_172280	2210018M11Rik	4031	NM_001100116	1700047I17Rik2	3863
NM_172274	Cc2d2a	5463	NM_172283	Fuk	3874	NM_172442	Dtx4	5786
NM_173737	Hmces	1422	NM_172286	6430548M08Rik	5565	NM_027031	Efcab9	767

NM 172279	Mark4	2259	NM 026935	Sult1c2	1465	NM 009075	Rpia	1789
NM 173738	Zfp940	2806	NM 026936	Oxa1l	2534	NM 001111286	Omt2a	1231
NM 001099308	Gm14391	2496	NM 009009	Rad21	3632	NM 172455	Tmprss7	2585
NM_172280	2210018M 11Rik	4031	NM_026943	Snrpd2	508	NM_009082	Rpl29	710
NM 172282	Tmco3	4533	NM 001099314	Msmp	743	NM 172458	Zfp871	10857
NM 026932	Ebna1bp2	2960	NM 009015	Rad54l	3093	NM 172462	Zfp11	3916
NM 009009	Rad21	3632	NM 026950	Ociad2	2201	NM 001100449	Taf4b	5149
NM 172289	Slc36a4	1937	NM 026951	Pex11g	1126	NM 009086	Polr1b	4017
NM 009015	Rad54l	3093	NM 026952	Cdk12	4846	NM 009088	Polr1a	6145
NM_001099323	Zkscan16	2794	NM_172298	Tshz3	5002	NM_027048	1700008P0 2Rik	788
NM 172295	Mab21l3	3558	NM 172385	Zfp536	4411	NM 172467	Zc3hav1l	6538
NM 026950	Ociad2	2201	NM 026962	Kbtbd3	2353	NM 027050	Smim23	606
NM 001099325	Gm10488	902	NM 172304	Tex10	3203	NM 027052	Slc38a4	3932
NM 026952	Cdk12	4846	NM 172306	Cyp4a12b	2395	NM 172470	Wdr35	4409
NM 172298	Tshz3	5002	NM 001099348	Gm13305	1953	NM 172471	Itih5	7942
NM 026955	Vstm5	1988	NM 001099349	Gm14308	4538	NM 172472	Tfe3	3293
NM 009025	Rasa3	4118	NM 001099628	Atad2b	8102	NM 009099	Trim30a	3771
NM 172392	Zfp759	3482	NM 026977	Cnppd1	2738	NM 172476	Tmc7	4493
NM 172302	Cpsf7	3604	NM 001099632	Rnf39	1446	NM 027060	Btbd9	6857
NM 001099328	Zfp831	9807	NM 023500	Xk	5062	NM 009103	Rrm1	3997
NM 026962	Kbtbd3	2353	NM 001099633	Dnah9	13774	NM 009104	Rrm2	2199
NM_172306	Cyp4a12b	2395	NM_001099637	Cep170	6930	NM_001101475	F830016B0 8Rik	3297
NM_172307	Mbtps2	4799	NM_172403	2810021J 22Rik	4966	NM_172480	Mtrr	3692
NM 026965	Comtd1	2241	NM 001099738	Dnajc28	3535	NM 172484	Nckap5	7107
NM_001099347	Gm10230	894	NM_172406	Trak2	6286	NM_027070	1700019A0 2Rik	627
NM 001099348	Gm13305	1953	NM 026994	Cryzl1	1747	NM 172485	Thsd7b	6283
NM 001099349	Gm14308	4538	NM 172410	Nup93	2928	NM 001100458	Fam169a	4570
NM_026977	Cnppd1	2738	NM_027000	Gtpbp4	2579	NM_001101503	A230065H1 6Rik	657
NM 001099631	Sh2d5	3158	NM 009037	Rcn1	3063	NM 172490	Sepsecs	1925
NM 172393	Aim1	7416	NM 172414	Zc2hc1c	4482	NM 023508	Pdcl2	1117
NM_172403	2810021J 22Rik	4966	NM_009044	Rel	2584	NM_009122	Satb1	6228
NM 026989	Srsf11	3103	NM 009048	Reps1	2618	NM 172492	Lrtm2	3579
NM 172405	Fam175a	2499	NM 001242937	Gm7903	3836	NM 027078	Ikbip	1257
NM 001099738	Dnajc28	3535	NM 009052	Bex1	890	NM 172495	Ncoa7	5134
NM 009037	Rcn1	3063	NM 172424	Med13l	9315	NM 172497	Efhb	2661
NM 027007	Zfp397	5487	NM 009056	Rfx2	3278	NM 172499	Mfsd9	3152
NM 172414	Zc2hc1c	4482	NM 172428	Ccdc134	2073	NM 009132	Scin	2708
NM 172421	Asxl2	8902	NM 172435	P2ry10	2827	NM 027086	Ubl7	1330
NM 172426	Slc24a2	10606	NM 172437	Pus7l	2848	NM 009135	Scn7a	7333
NM_009055	Rfx1	4179	NM_001100116	1700047I 17Rik2	3863	NM_009136	Scrg1	700
NM 172430	Sphkap	6512	NM 172442	Dtx4	5786	NM 027088	Bap1	3406
NM 027019	Odf3	924	NM 001100180	Cyp3a57	1589	NM 172508	Dse	4243
NM 001099919	Gm10147	893	NM 001100181	Cyp4a32	1914	NM 009140	Cxcl2	1083
NM 001100109	Srp54b	3286	NM 172443	Tbc1d16	6988	NM 172509	Kctd7	4266
NM 001100110	Srp54c	2516	NM 172444	Thsd4	7604	NM 172513	Fam126b	8695
NM 172442	Dtx4	5786	NM 172445	Wdr37	4555	NM 009145	Nptn	2077
NM 172443	Tbc1d16	6988	NM 001100186	Zscan4d	1992	NM 172515	Zbbx	2446
NM 001100182	Cyp2j12	1944	NM 001100187	Cyp4f37	2642	NM 009147	Sec23a	4108
NM 001242941	Gm3317	1743	NM 009076	Rpl12	643	NM 001101588	Cyp4f40	1578
NM 172445	Wdr37	4555	NM 172451	Galnt6	5711	NM 009148	Exoc4	3723
NM 001100186	Zscan4d	1992	NM 172460	Nphp3	2107	NM 172519	Dis3l	3479
NM 172455	Tmprss7	2585	NM 172461	Nek11	2625	NM 009149	Glg1	3880
NM 172458	Zfp871	10857	NM 009085	Polr1c	1305	NM 172522	Megf11	3669
NM 001101433	Zcchc24	4375	NM 027045	Ccser2	7500	NM 001243002	Spin2d	1015

NM 172462	Zfp11	3916	NM 009088	Polr1a	6145	NM 172530	She	5745
NM 001100449	Taf4b	5149	NM 172465	Zdhhc9	2995	NM 027109	Dnase1l1	1635
NM 172463	Sned1	4474	NM 009090	Polr2c	1580	NM 001243008	Col6a3	10845
NM 027045	Ccser2	7500	NM 027052	Slc38a4	3932	NM 001243009	Col6a3	9024
NM 009088	Polr1a	6145	NM 172470	Wdr35	4409	NM 172536	Zfp609	8602
NM 001100452	Gltscr1l	6579	NM 172471	Itih5	7942	NM 001111317	BC048609	569
NM 172466	Adamts18	4855	NM 001101467	Cyp2a22	2226	NM 027116	Nkpd1	2753
NM 172467	Zc3hav1l	6538	NM 172472	Tfe3	3293	NM 027118	Cdk13	6901
NM_001100455	Lcn11	652	NM_001101472	Serpina3j	1999	NM_009166	Sorbs1	5471
NM 027053	Lrrc51	874	NM 027060	Btbd9	6857	NM 027121	Vkorc1l1	4935
NM_172471	Itih5	7942	NM_009103	Rrm1	3997	NM_001243018	E030019B06Rik	1667
NM_027056	1700012P22Rik	972	NM_172477	Dennd2a	4435	NM_172544	Nrxn3	6529
NM_173747	Gpkow	3591	NM_001101475	F830016B08Rik	3297	NM_172549	Cabin1	7483
NM 001101467	Cyp2a22	2226	NM 009106	Rtkn	2609	NM 023517	Tnfsf13	1679
NM_172475	Frmd4a	6338	NM_001101478	D3Ertd254e	6625	NM_009182	St8sia3	6306
NM 172477	Dennd2a	4435	NM 001101482	Mrap2	1948	NM 173753	Fnip1	6281
NM_001101475	F830016B08Rik	3297	NM_001101488	Gsg1l	3924	NM_027132	Otop3	2422
NM 172480	Mtrr	3692	NM 172485	Thsd7b	6283	NM 027133	Lnp	8692
NM_001101478	D3Ertd254e	6625	NM_001100458	Fam169a	4570	NM_172563	Hlf	5665
NM 172484	Nckap5	7107	NM 009119	Sap18	3472	NM 172564	Tns4	4755
NM 001101486	Fam71f2	1203	NM 172488	Lacc1	2419	NM 009190	Vps4b	3272
NM 001101488	Gsg1l	3924	NM 009122	Satb1	6228	NM 001243041	Adk	1838
NM 023508	Pdcl2	1117	NM 172492	Lrtm2	3579	NM 009191	Clpb	4492
NM 009122	Satb1	6228	NM 027078	Ikbip	1257	NM 027139	Taf9	1240
NM 027081	Dennd6b	4716	NM 027081	Dennd6b	4716	NM 001102411	Kng1	2299
NM_009126	Serpinb3a	1629	NM_009132	Scin	2708	NM_027144	Arhgef12	10483
NM 172497	Efhb	2661	NM 172502	Dcaf15	2312	NM 009198	Slc17a1	2049
NM 172502	Dcaf15	2312	NM 009135	Scn7a	7333	NM 001102412	Kng1	1756
NM 172503	Zswim4	4348	NM 172507	Sh3bgrl2	3501	NM 001102414	Slc2a9	3602
NM 172507	Sh3bgrl2	3501	NM 172508	Dse	4243	NM 172578	Mis18bp1	4038
NM 009138	Ccl25	1073	NM 001101572	Vmn2r82	2598	NM 001102415	Slc2a9	3281
NM 172509	Kctd7	4266	NM 027091	Nup35	2732	NM 172580	Acot6	1822
NM 172513	Fam126b	8695	NM 027097	Klk12	930	NM 172585	Larp4b	4707
NM 172516	Dstyk	6385	NM 009147	Sec23a	4108	NM 001243047	Dlg2	5933
NM 009148	Exoc4	3723	NM 009148	Exoc4	3723	NM 172586	Zfp322a	4844
NM 172522	Megf11	3669	NM 009149	Glg1	3880	NM 001102455	Aplp2	3658
NM 027109	Dnase1l1	1635	NM 009150	Selenbp1	1685	NM 001102456	Aplp2	3526
NM_172534	4932411E22Rik	2665	NM_027104	2310002L09Rik	958	NM_001102458	Azin1	5028
NM 172538	Vezt	4479	NM 172525	Arhgap29	5133	NM 172592	Srek1	4063
NM 009162	Scg5	1227	NM 027109	Dnase1l1	1635	NM 172595	Arl15	3376
NM 027118	Cdk13	6901	NM 001243008	Col6a3	10845	NM 172596	Sec24c	4446
NM_009166	Sorbs1	5471	NM_172534	4932411E22Rik	2665	NM_172601	Rab2b	2770
NM 027121	Vkorc1l1	4935	NM 001243009	Col6a3	9024	NM 009229	Sntb2	4423
NM 001100446	Gm4297	989	NM 001101620	Klra8	1178	NM 172606	6-Mar	6236
NM 172544	Nrxn3	6529	NM 009161	Sgca	1738	NM 172608	Tmem184b	3388
NM 172546	Cnksr3	3448	NM 172538	Vezt	4479	NM 001102582	Vmn2r18	2385
NM 001100596	Gm2002	2680	NM 009168	Shd	1548	NM 001102607	Col6a6	7098
NM_001243021	Zfp939	1980	NM_001243018	E030019B06Rik	1667	NM_001102613	Phldb3	2282
NM 023517	Tnfsf13	1679	NM 172544	Nrxn3	6529	NM 009243	Serpina1a	1724
NM 001100609	Gm10487	907	NM 172546	Cnksr3	3448	NM 172618	Btbd9	6657
NM 172550	Ybey	4538	NM 001100596	Gm2002	2680	NM 172619	Adamts10	4065

NM 001100610	Gm14632	894	NM 172549	Cabin1	7483	NM 001102660	Gm2663	870
NM 172552	Tdg	3218	NM 172550	Ybey	4538	NM 172625	Ino80c	2360
NM 172553	Alx1	2493	NM 009177	St3gal1	5784	NM 172626	Rbm27	6266
NM 009177	St3gal1	5784	NM 009182	St8sia3	6306	NM 001243074	Cep57l1	2233
NM 173753	Fnip1	6281	NM 173753	Fnip1	6281	NM 001243075	Cep57l1	2011
NM 001101804	Gm14434	4538	NM 001101804	Gm14434	4538	NM 172637	Hectd2	4771
NM 027133	Lnp	8692	NM 027133	Lnp	8692	NM 172641	Plekhs1	2385
NM 001102404	Acp5	1420	NM 172561	Spag7	1161	NM 009261	Strbp	16348
NM 001102405	Acp5	1425	NM 172562	Tada2a	2119	NM 001103162	Scap	4138
NM 172563	Hlf	5665	NM 172563	Hlf	5665	NM 172643	Zbtb41	8362
NM 001243039	Gm4070	9037	NM 009190	Vps4b	3272	NM 009262	Spock1	4674
NM 001243040	Gm4070	8843	NM 009191	Clpb	4492	NM 172645	Suco	6308
NM 009191	Clpb	4492	NM 027143	Khnyn	3876	NM 009268	Mucl1	826
NM 027144	Arhgef12	10483	NM 027144	Arhgef12	10483	NM 009269	Sptlc1	2601
NM 001102414	Slc2a9	3602	NM 001102423	Stx16	3756	NM 172655	Hecw2	1132
NM 027148	Exosc8	1270	NM 001102424	Stx16	3717	NM 027192	Ttl	4562
NM 009201	Slc1a5	2758	NM 001102425	Stx16	3705	NM 172658	Slco4c1	2394
NM 001102415	Slc2a9	3281	NM 001102436	Acbd5	3616	NM 173760	Ppip5k2	5568
NM 009202	Slc22a1	1994	NM 172584	Itpk1	2856	NM 001103181	Tulp4	8872
NM 173756	Lin52	2144	NM 001102437	Acbd5	3746	NM 009275	Srprb	2991
NM 172583	Tmem63c	3369	NM 009207	Slc4a2	4202	NM 172664	Tlk1	4050
NM 009206	Slc4a1ap	2445	NM 172585	Larp4b	4707	NM 172666	Agps	7375
NM 172585	Larp4b	4707	NM 001102438	Acbd5	3743	NM 001103367	Rai2	2198
NM 172587	Cdc14b	5859	NM 001243047	Dlg2	5933	NM 009282	Stag1	6010
NM 009210	Hltf	4956	NM 172586	Zfp322a	4844	NM 009285	Stc1	3743
NM 001102455	Aplp2	3658	NM 001102444	Add1	4047	NM 172672	Ganc	4818
NM 027154	Tmbim1	2325	NM 009210	Hltf	4956	NM 027210	Ceacam13	1004
NM 172591	Fcho2	5141	NM 009212	Ighmbp2	5562	NM 009289	Slk	7047
NM 001102456	Aplp2	3526	NM 001243049	Atp6v0a1	3841	NM 027218	Clec4b1	685
NM 001102458	Azin1	5028	NM 009213	Smpd2	1621	NM 172683	Pogz	7642
NM 172593	Mier3	5277	NM 001243050	Atp6v0a1	4001	NM 172684	Rsbn1	6727
NM 027162	Mif4gd	1671	NM 001102458	Azin1	5028	NM 001104547	Vmn2r96	2343
NM 172608	Tmem184b	3388	NM 172592	Srek1	4063	NM 009302	Swap70	4056
NM 027166	Ypel5	2869	NM 001243051	Atp6v0a1	4022	NM 027225	Cobll1	4931
NM 172611	Gramd4	4117	NM 027156	Ddx51	4874	NM 172687	Coq3	2613
NM 172613	Atp13a4	4057	NM 172598	Wdhd1	4190	NM 172694	Megf9	3062
NM_009243	Serpina1a	1724	NM_009225	Snrpb	1121	NM_027237	2010003K11Rik	1189
NM 172619	Adamts10	4065	NM 172604	Scara3	3405	NM 009308	Syt4	3901
NM 001102662	Skint1	1260	NM 009230	Soat1	4785	NM 172697	Prpf38a	1519
NM 001102677	Gm1993	923	NM 172608	Tmem184b	3388	NM 027246	Snrpf	869
NM_001102678	Gm10096	921	NM_027168	Hddc2	718	NM_027251	2010107G23Rik	1731
NM 172626	Rbm27	6266	NM 172614	Tmem44	4241	NM 172705	Phf13	3128
NM_172637	Hectd2	4771	NM_172613	Atp13a4	4057	NM_172706	9330182L06Rik	4627
NM 172639	Dpcd	1353	NM 001102607	Col6a6	7098	NM 173764	Tapt1	3508
NM 009261	Strbp	16348	NM 027171	Camsap3	4303	NM 027258	Rnf157	4655
NM_172643	Zbtb41	8362	NM_009243	Serpina1a	1724	NM_009323	Tbx15	3585
NM 009262	Spock1	4674	NM 172618	Btbd9	6657	NM 172711	Guf1	3978
NM_172645	Suco	6308	NM_009244	Serpina1b	1434	NM_027260	Vrk2	1906
NM 172652	Kansl3	4663	NM 172619	Adamts10	4065	NM 172713	Sdad1	5020
NM_027188	Smyd3	3769	NM_009245	Serpina1c	1424	NM_172714	Lin54	4464
NM 009269	Sptlc1	2601	NM 027174	Col22a1	6128	NM 027261	Taf1d	1381
NM_027192	Ttl	4562	NM_009246	Serpina1d	1442	NM_172716	Pcgf3	3731
NM_173760	Ppip5k2	5568	NM_009247	Serpina1e	1374	NM_009329	Zfp354a	4029

NM_172660	D2Wsu81e	1838	NM_172626	Rbm27	6266	NM_027271	D3Ertd751e	3322
NM 001103181	Tulp4	8872	NM 172628	Sh3tc2	4562	NM 172728	Creb5	2873
NM 009274	Srpk2	6436	NM 001243074	Cep57l1	2233	NM 172730	Ccdc174	1757
NM 172666	Agps	7375	NM 001243075	Cep57l1	2011	NM 001104592	Vmn2r2	2484
NM 172672	Ganc	4818	NM 172633	Cbln2	2201	NM 023543	Chn2	2653
NM 172674	Phf20	5752	NM 172635	Patl1	4221	NM 172734	Stk38l	4617
NM 009289	Slk	7047	NM 172641	Plekhs1	2385	NM 009344	Phlda1	1953
NM 172684	Rsbn1	6727	NM 009261	Strbp	16348	NM 009345	Dntt	2133
NM 172687	Coq3	2613	NM 172643	Zbtb41	8362	NM 172739	Arhgap35	8393
NM 023530	Pla2g12b	1080	NM 009262	Spock1	4674	NM 172742	Mtmr10	5205
NM 009308	Syt4	3901	NM 172652	Kansl3	4663	NM 027288	Manba	3688
NM_027239	1810065E05Rik	925	NM_172656	Stradb	2338	NM_001104615	Vmn2r4	2484
NM 172697	Prpf38a	1519	NM 027192	Ttl	4562	NM 009351	Tep1	8163
NM 172700	Zmpste24	3430	NM 173760	Ppip5k2	5568	NM 172746	Hirip3	2511
NM 009315	Taf6	2264	NM 009273	Srp14	790	NM 009356	Prss40	1301
NM_027251	2010107G23Rik	1731	NM_172660	D2Wsu81e	1838	NM_172752	Sorbs2	6040
NM 172710	Sel1l3	4532	NM 001103181	Tulp4	8872	NM 009359	Tex9	4317
NM 009323	Tbx15	3585	NM 009275	Srprb	2991	NM 001104618	Vmn2r5	2484
NM 172711	Guf1	3978	NM 001104525	Cyp2c69	1770	NM 172759	Ces2e	2699
NM 027260	Vrk2	1906	NM 009286	Sult2a2	741	NM 172760	Elmo3	3060
NM 172714	Lin54	4464	NM 001104531	Cyp2d11	1590	NM 172765	Zbtb44	8828
NM 009329	Zfp354a	4029	NM 172674	Phf20	5752	NM 172767	Vwa5a	4157
NM 001104592	Vmn2r2	2484	NM 172675	Stx16	3768	NM 172768	Gramd1b	2814
NM 172734	Stk38l	4617	NM 009288	Stk10	5082	NM 172771	Dmxl2	10614
NM 172736	Leng8	5094	NM 009294	Stx4a	1372	NM 172772	Fam63b	8081
NM 172739	Arhgap35	8393	NM 172682	Fam160a1	4392	NM 027314	5-Mar	4113
NM 023546	Ssty2	1216	NM 172683	Pogz	7642	NM 173364	Zfp445	6189
NM 172742	Mtmr10	5205	NM 001104547	Vmn2r96	2343	NM 024442	Cyp4f16	2192
NM 009350	Adad1	1972	NM 173762	Cenpe	7813	NM 172776	Slc22a29	2208
NM 027288	Manba	3688	NM 009302	Swap70	4056	NM 172779	Ddx26b	3808
NM 001104615	Vmn2r4	2484	NM 173763	Ccbl2	2246	NM 172780	Slc9a6	4920
NM 172746	Hirip3	2511	NM 027226	Fyttd1	4397	NM 009390	Tll1	4928
NM 172747	Kctd13	1654	NM 172687	Coq3	2613	NM 172788	Sh3rf3	5796
NM 172749	Zfp646	6327	NM 172693	Galnt12	2216	NM 172790	Ankrd52	6615
NM 027293	Dopey2	7331	NM 009308	Syt4	3901	NM 009393	Tnnc1	703
NM 009356	Prss40	1301	NM 172704	Dnajc11	2944	NM 027341	Dzip3	5153
NM_001104618	Vmn2r5	2484	NM_172706	9330182L06Rik	4627	NM_027347	Med23	4928
NM 027301	Sdr9c7	2863	NM 023537	Rab3b	3320	NM 172803	Dock4	8072
NM 172765	Zbtb44	8828	NM 173764	Tapt1	3508	NM 172804	Syt16	2705
NM 172767	Vwa5a	4157	NM 172710	Sel1l3	4532	NM 172806	Btbd7	4325
NM 027307	Golm1	3693	NM 027258	Rnf157	4655	NM 172809	Sacs	3343
NM 009369	Tgfbi	2678	NM 009323	Tbx15	3585	NM 009412	Tpd52	2266
NM 172771	Dmxl2	10614	NM 172711	Guf1	3978	NM 172810	Gucy1b2	2717
NM 172772	Fam63b	8081	NM 172714	Lin54	4464	NM 027375	Gcc2	6528
NM 027314	5-Mar	4113	NM 172716	Pcgf3	3731	NM 172814	Lrp12	4064
NM 172775	Plxnb1	8600	NM 027266	Trmt10b	2023	NM 027379	Far1	4339
NM 172776	Slc22a29	2208	NM 172718	Sgsm1	5171	NM 172819	Dip2b	8127
NM 027321	Lrrc39	2880	NM 009329	Zfp354a	4029	NM 023565	Csel1	3604
NM 001243161	Alg11	4970	NM 172721	Fbxw8	4978	NM 172824	Ccdc14	4107
NM 172779	Ddx26b	3808	NM 172725	Ap5z1	3817	NM 001243199	Gimap4	1365
NM 027326	Mllt3	6129	NM 172731	Fgd5	5636	NM 172832	Pcyox1l	2017
NM_172786	Il20ra	2088	NM_009338	Acat2	2076	NM_172836	9930021J03Rik	8026
NM_009390	Tll1	4928	NM_009341	Tcp10b	2065	NM_001243238	4930444P10Rik	717
NM 027341	Dzip3	5153	NM 172733	Dera	1688	NM 172837	Lipk	2225
NM 009395	Tnfaip1	3709	NM 023543	Chn2	2653	NM 172838	Slc16a12	3870
NM 027347	Med23	4928	NM 172736	Leng8	5094	NM 172840	Vwa2	3885

NM_009403	Tnfsf8	2228	NM_172741	4931406P16Rik	5808	NM_172841	Slco5a1	8526
NM 172800	Sdk2	6604	NM 023546	Ssty2	1216	NM 027399	Steap1	1216
NM 172803	Dock4	8072	NM 009348	Tectb	2725	NM 001104927	Cyp2j8	1520
NM 172805	Kcnh5	4061	NM 027287	Dnajb4	2570	NM 027402	Fndc5	2718
NM 172806	Btbd7	4325	NM 172743	Plekha7	4312	NM 172849	Nyap2	4919
NM 172807	Ppwd1	2367	NM 009351	Tep1	8163	NM 009441	Ttc3	7295
NM 172810	Gucy1b2	2717	NM 172746	Hirip3	2511	NM 172853	Cdh7	3353
NM 027375	Gcc2	6528	NM 172747	Kctd13	1654	NM 001105060	Vmn2r63	2580
NM 027379	Far1	4339	NM 027293	Dopey2	7331	NM 009447	Tuba4a	2053
NM 173769	Zfp641	3673	NM 009358	Ppp2r5d	2926	NM 172857	Exd1	3143
NM_172823	Lmln	4111	NM_172753	Csgalnact1	3943	NM_172858	Pak7	4773
NM 023565	Csel1	3604	NM 009359	Tex9	4317	NM 172860	Cbfa2t2	6100
NM 001243223	Rgsl1	3640	NM 172757	Heatr3	3190	NM 027418	Mapk6	4209
NM 172833	Malt1	5008	NM 172765	Zbtb44	8828	NM 172861	Slc7a14	8869
NM 172834	Loxhd1	6595	NM 172767	Vwa5a	4157	NM 009453	Zrsr2	2812
NM 173770	Fam69c	2153	NM 027307	Golm1	3693	NM 172868	Palm2	8213
NM 172838	Slc16a12	3870	NM 027309	Lysmd2	1200	NM 009457	Uba1	4068
NM 001104927	Cyp2j8	1520	NM 172771	Dmxl2	10614	NM 172869	Frmd3	3977
NM 027400	Lman1	3576	NM 172772	Fam63b	8081	NM 027435	Atad2	5683
NM 001104946	Gm2012	892	NM 172775	Plxnb1	8600	NM 009461	Ubr1	7765
NM 172845	Adamts4	3673	NM 173364	Zfp445	6189	NM 172876	Gpatch3	2003
NM 172850	Ankmy1	3243	NM 172779	Ddx26b	3808	NM 173773	Oog4	1959
NM 027403	Psg21	2159	NM 009390	Tll1	4928	NM 027442	Ddo	3002
NM 009441	Ttc3	7295	NM 172790	Ankrd52	6615	NM 172880	Tmprss11e	3408
NM 024237	Fbln7	2922	NM 009396	Tnfaip2	3590	NM 009471	Umps	3219
NM 172851	Cntnap5b	5120	NM 172796	Slfn9	3856	NM 027450	Glipr2	2019
NM 027405	Gpatch21	3947	NM 009403	Tnfsf8	2228	NM 172881	Ugt2b35	3358
NM 009443	Tgoln1	5025	NM 172800	Sdk2	6604	NM 001105159	Cyp3a41b	2064
NM 027407	Ical1	3667	NM 027355	Rnf168	4468	NM 172882	Wdfy3	14277
NM 009447	Tuba4a	2053	NM 009410	Top3a	3740	NM 001105160	Cyp3a59	1999
NM_027411	Spdl1	2526	NM_172805	Kcnh5	4061	NM_172884	2900026A02Rik	5838
NM 027418	Mapk6	4209	NM 172806	Btbd7	4325	NM 027455	Qpct	1924
NM 172861	Slc7a14	8869	NM 172809	Sacs	3343	NM 172885	Tmem132d	4278
NM 027421	Ints2	5922	NM 027372	Alkbh7	765	NM 023579	Ipo5	4598
NM 172865	Manea	4645	NM 027379	Far1	4339	NM 023580	Epha1	3273
NM 027425	Rufy2	8936	NM 027381	Cactin	2724	NM 173775	Ccdc37	2141
NM 009454	Ube2e3	1562	NM 173769	Zfp641	3673	NM 009488	Vmn2r122	2848
NM 172867	Zfp462	10514	NM 172819	Dip2b	8127	NM 172892	Slc13a4	3426
NM 172868	Palm2	8213	NM 172823	Lmln	4111	NM 027476	Zdhhc24	2562
NM 009457	Uba1	4068	NM 009423	Traf4	3016	NM 009492	Vmn2r122	2953
NM_172870	Bnc2	6453	NM_172825	Gpr128	2701	NM_027482	5730508B09Rik	1603
NM 172872	Kank4	4801	NM 172827	Lnpep	5181	NM 023585	Ube2v2	5892
NM 027442	Ddo	3002	NM 172829	St6gal2	2278	NM 172908	Ovch2	1924
NM 172879	Lrrd1	2951	NM 172830	Slc4a9	2945	NM 027491	Rragd	5047
NM_172880	Tmprss11e	3408	NM_172833	Malt1	5008	NM_027496	Ankrd33b	7471
NM 027448	Lca5	4235	NM 172834	Loxhd1	6595	NM 172916	Hydin	15783
NM_009471	Umps	3219	NM_172838	Slc16a12	3870	NM_027512	3830417A13Rik	1649
NM 172881	Ugt2b35	3358	NM 172840	Vwa2	3885	NM 001105196	Tfe3	3188
NM 009476	Upk2	890	NM 172841	Slco5a1	8526	NM 172924	Peak1	10994
NM 009477	Upp1	1297	NM 027402	Fndc5	2718	NM 001105245	Pcdh19	10309
NM 001105159	Cyp3a41b	2064	NM 172849	Nyap2	4919	NM 172925	Klhl31	6260
NM 027453	Btf3l4	3111	NM 172850	Ankmy1	3243	NM 001105246	Pcdh19	10165
NM 172882	Wdfy3	14277	NM 009441	Ttc3	7295	NM 172928	Dclk3	3498
NM 001105160	Cyp3a59	1999	NM 027405	Gpatch21	3947	NM 027528	Nos1ap	1533
NM_172884	2900026A02Rik	5838	NM_009445	Ttk	2904	NM_173780	Klf8	4457

NM_001243584	Mif4gd	1387	NM_001105059	Vmn2r62	2580	NM_009517	Zmat3	7784
NM_001243586	Mif4gd	1599	NM_001105060	Vmn2r63	2580	NM_173048	Gga3	3703
NM_001243587	Mif4gd	1315	NM_009447	Tuba4a	2053	NM_027534	Kdsr	5582
NM_023579	Ipo5	4598	NM_027410	Tecpr1	4195	NM_172937	Shprh	7385
NM_172887	Fry	10753	NM_172857	Exd1	3143	NM_001105561	Gm11545	2707
NM_172888	Svs1	2778	NM_027412	Ttc9c	3470	NM_027545	Cwf19l2	3953
NM_172890	Slc6a11	4066	NM_172861	Slc7a14	8869	NM_027547	Prdm5	2182
NM_172891	Styk1	3378	NM_172864	Wdr63	3082	NM_009527	Wnt7a	3176
NM_172893	Parp12	3322	NM_027425	Rufy2	8936	NM_001109626	Cdk12	7669
NM_172894	Ppp6r1	3812	NM_172867	Zfp462	10514	NM_001109628	Cdk12	7642
NM_009492	Vmn2r122	2953	NM_172868	Palm2	8213	NM_027556	Cep192	8125
NM_172903	Man2a2	6575	NM_027429	Cenpl	4342	NM_172960	Adck5	2052
NM_172905	E030002003Rik	2350	NM_172870	Bnc2	6453	NM_009533	Xrcc5	2571
NM_023585	Ube2v2	5892	NM_172879	Lrrd1	2951	NM_027562	Clec2g	2466
NM_027490	Dcp2	8643	NM_001243305	Ncmap	1448	NM_001109688	C2cd5	4310
NM_027493	Actr8	2121	NM_001243306	Ncmap	2005	NM_001109690	Phf21a	6328
NM_027496	Ankrd33b	7471	NM_027446	Fam86	2140	NM_001109691	Phf21a	6484
NM_027497	Epc1	3983	NM_027448	Lca5	4235	NM_001109747	Cenpw	1527
NM_172916	Hydin	15783	NM_172882	Wdfy3	14277	NM_001109750	Dgcr2	4090
NM_172918	Zfp317	4050	NM_001105160	Cyp3a59	1999	NM_027582	Akr1cl	1353
NM_172919	Zfp846	2755	NM_172884	2900026A02Rik	5838	NM_172991	Rbm48	2344
NM_173777	Olfm2	1525	NM_027456	Armc9	2178	NM_172992	Phtf2	4839
NM_172920	Dpy19l1	4753	NM_001243757	Pde2a	4115	NM_027587	Micalcl	2397
NM_027513	Nup205	6244	NM_001243758	Pde2a	4112	NM_009557	Zfp46	4637
NM_172924	Peak1	10994	NM_023579	Ipo5	4598	NM_001109756	Adcy7	5937
NM_027518	Gpr137c	2444	NM_001243760	Ciita	5359	NM_001109757	Atp7a	8198
NM_001105245	Pcdh19	10309	NM_001243761	Ciita	5091	NM_027597	Fam71d	1744
NM_172925	Klhl31	6260	NM_172887	Fry	10753	NM_027599	Lpcat2b	2782
NM_001105246	Pcdh19	10165	NM_027469	Gfod2	2036	NM_009562	Zfp62	3934
NM_001243857	Fam124a	3557	NM_173775	Ccdc37	2141	NM_027600	4921504E06Rik	2004
NM_172928	Dclk3	3498	NM_009488	Vmn2r122	2848	NM_027602	Nsun7	2552
NM_173780	Klf8	4457	NM_172891	Styk1	3378	NM_009566	Zfp92	5749
NM_009517	Zmat3	7784	NM_172893	Parp12	3322	NM_173014	Lpcat2	2800
NM_173048	Gga3	3703	NM_027476	Zdhhc24	2562	NM_027604	Usp15	3215
NM_027534	Kdsr	5582	NM_172899	Dmkn	2022	NM_173018	Myo9a	11974
NM_172937	Shprh	7385	NM_009492	Vmn2r122	2953	NM_027613	Mmrn1	4636
NM_027541	Prpf3	2732	NM_172903	Man2a2	6575	NM_173021	Phka1	5938
NM_009522	Wnt3a	2791	NM_172904	Fsd2	2976	NM_027618	Poteg	1706
NM_172947	Lsm12	1224	NM_172905	E030002003Rik	2350	NM_009567	Zfp93	3510
NM_027548	Serpinb7	1894	NM_023585	Ube2v2	5892	NM_009570	Zfy1	2785
NM_027551	Klhl30	3020	NM_172907	Olfml1	1969	NM_009572	Zhx1	5105
NM_001109043	Zfp185	3554	NM_027485	Med26	3040	NM_001109975	Synpo	4496
NM_027554	Usp38	4711	NM_027490	Dcp2	8643	NM_027629	Pgm2l1	8641
NM_009529	Gm4836	917	NM_027491	Rragd	5047	NM_001109985	Nos1ap	2973
NM_027556	Cep192	8125	NM_172910	Dlgap2	4525	NM_173029	Adcy10	5209
NM_172958	Mtmr12	4518	NM_172912	Abcc12	4511	NM_173030	Galnt13	6966
NM_172960	Adck5	2052	NM_027496	Ankrd33b	7471	NM_027635	4931423N10Rik	1842
NM_001109684	Kazn	3841	NM_172916	Hydin	15783	NM_001109991	Col18a1	5642
NM_001109685	Kazn	3859	NM_173776	Maml2	4452	NM_173032	Tbck	3226
NM_001243908	Zfp383	2620	NM_172920	Dpy19l1	4753	NM_001244628	5430401F13Rik	602
NM_027562	Clec2g	2466	NM_001105196	Tfe3	3188	NM_173037	Tango6	4409
NM_009535	Yes1	4608	NM_027513	Nup205	6244	NM_001110015	Wdr36	3281
NM_001109688	C2cd5	4310	NM_172924	Peak1	10994	NM_001110016	Wdr36	2942
NM_172965	Sap130	4015	NM_001105245	Pcdh19	10309	NM_001110017	Dzip3	5771
NM_172966	Sh3rf2	4688	NM_001105246	Pcdh19	10165	NM_027652	Ept1	6769
NM_001109690	Phf21a	6328	NM_001105252	Tmc5	3774	NM_173066	Ep400	10690

NM_001109691	Phf21a	6484	NM_027529	6330409D20Rik	1133	NM_001110131	Cic	6101
NM_009543	Rnf103	3264	NM_172937	Shprh	7385	NM_001110132	Cic	6104
NM_027572	Slc22a16	2377	NM_172938	Scml4	4745	NM_173182	Fndc3b	6875
NM_001109750	Dgcr2	4090	NM_027541	Prpf3	2732	NM_024241	Kif24	5616
NM_009549	Zfp185	3557	NM_172943	Alkbh5	6025	NM_173186	Tbc1d24	7796
NM_172993	Zfp512	3317	NM_027547	Prdm5	2182	NM_027673	Tssk4	1243
NM_001109757	Atp7a	8198	NM_172946	Krt222	2735	NM_173189	Mcph1	4636
NM_027594	Ttll7	6075	NM_009526	Wnt6	2068	NM_001244692	St18	5913
NM_027597	Fam71d	1744	NM_009527	Wnt7a	3176	NM_173370	Cds1	3939
NM_009560	Zfp60	4057	NM_001109043	Zfp185	3554	NM_001244693	St18	5835
NM_173008	Ssc5d	4260	NM_001109626	Cdk12	7669	NM_009613	Adam11	4647
NM_027599	Lpcat2b	2782	NM_001109628	Cdk12	7642	NM_001110195	Echdc1	2946
NM_173013	Map1s	3284	NM_027556	Cep192	8125	NM_001110196	Rnf146	4359
NM_009566	Zfp92	5749	NM_001109657	Gas7	7105	NM_001110197	Rnf146	4420
NM_173014	Lpcat2	2800	NM_027559	Wdr96	5947	NM_001110198	Rnf146	4400
NM_001109873	Cbfa2t3	7678	NM_001109684	Kazn	3841	NM_173375	Fam180a	1528
NM_173016	Vat1l	3655	NM_009533	Xrcc5	2571	NM_009618	Adam2	2551
NM_173018	Myo9a	11974	NM_001243903	Gm14322	4050	NM_173381	6720489N17Rik	2638
NM_173781	Rab6b	4734	NM_001109685	Kazn	3859	NM_173382	Proser1	4322
NM_001109909	Srrt	3018	NM_001243908	Zfp383	2620	NM_027709	Clec12b	1523
NM_173023	Catsperb	3716	NM_027562	Clec2g	2466	NM_001110209	Lnp	8775
NM_001109910	Srrt	3006	NM_001109688	C2cd5	4310	NM_001110212	Gigyf2	5725
NM_001109914	Apold1	3284	NM_027563	Krt34	1605	NM_001110216	Cbx5	8902
NM_009572	Zhx1	5105	NM_172965	Sap130	4015	NM_173384	Sox30	2995
NM_173027	Ip6k3	2305	NM_172966	Sh3rf2	4688	NM_027721	Katnal2	1673
NM_027629	Pgm2l1	8641	NM_172977	Gtf3c4	7021	NM_027724	Cage1	2871
NM_009577	Zik1	4191	NM_001109747	Cenpw	1527	NM_001110227	Kcnj13	1349
NM_173030	Galnt13	6966	NM_027572	Slc22a16	2377	NM_173390	Nhsl1	7335
NM_027643	Naa40	3275	NM_009549	Zfp185	3557	NM_173392	Zfyve16	5451
NM_001110017	Dzip3	5771	NM_172990	Pank4	2598	NM_023595	Dut	2358
NM_027651	Tmem30c	1382	NM_009557	Zfp46	4637	NM_009641	Angpt4	1530
NM_173066	Ep400	10690	NM_027590	Ints10	2410	NM_023596	Slc29a3	5240
NM_009596	Scgb1b27	424	NM_001109756	Adcy7	5937	NM_009643	Ahnak	18100
NM_173181	Zc2hc1a	3361	NM_001243968	Ube2m	1010	NM_001110240	Slc24a2	10516
NM_173182	Fndc3b	6875	NM_001109757	Atp7a	8198	NM_009644	Ahrr	4732
NM_173185	Csnk1g1	7183	NM_027594	Ttll7	6075	NM_173399	Zbtb5	4360
NM_173186	Tbc1d24	7796	NM_027600	4921504E06Rik	2004	NM_173400	Haus6	3719
NM_173189	Mcph1	4636	NM_001109873	Cbfa2t3	7678	NM_027756	Mfap3l	6337
NM_173350	Osbpl9	3148	NM_173018	Myo9a	11974	NM_173404	Bmp3	3112
NM_001244692	St18	5913	NM_027613	Mmrn1	4636	NM_009651	Akap4	2855
NM_001244693	St18	5835	NM_173021	Phka1	5938	NM_001110254	Zfp945	6497
NM_009613	Adam11	4647	NM_009572	Zhx1	5105	NM_027762	Tchhl1	2066
NM_001110195	Echdc1	2946	NM_173026	Zbtb11	4999	NM_173408	Dcun1d3	5637
NM_001110196	Rnf146	4359	NM_001109975	Synpo	4496	NM_027768	5430402E10Rik	798
NM_027699	Lrrc72	660	NM_173028	Vps13a	11178	NM_173410	Gpr26	2799
NM_001110197	Rnf146	4420	NM_001109985	Nos1ap	2973	NM_173413	Rab8b	4751
NM_001110198	Rnf146	4400	NM_173029	Adcy10	5209	NM_009661	Alox8	3273
NM_173376	Rbmx2	1743	NM_027633	Fancd2os	1358	NM_173422	Colec10	4587
NM_027702	4933421I07Rik	1373	NM_173030	Galnt13	6966	NM_027782	Kctd6	1667
NM_001110203	Trim9	4843	NM_027635	4931423N10Rik	1842	NM_173423	Fem1c	2866
NM_027706	Nbas	7256	NM_173031	Rbbp8nl	2319	NM_173425	Fam124b	2862
NM_173378	Trp53bp2	4377	NM_009579	Slc30a1	5230	NM_001110315	Kif1a	8233
NM_173382	Proser1	4322	NM_001109995	Eftud2	3400	NM_173428	Sspo	15586
NM_001110209	Lnp	8775	NM_027643	Naa40	3275	NM_027801	2610015P09Rik	3643
NM_001244916	Sall2	4771	NM_173037	Tango6	4409	NM_173431	Rpgrip1l	6969

NM 173383	Dnd1	1490	NM 009591	Aanat	1370	NM 173432	Pskh1	3317
NM 001110216	Cbx5	8902	NM 173038	Tbcel	4967	NM 027807	Cul5	6023
NM 001110218	Ppm1h	6413	NM 001110101	Lsm2	890	NM 009671	Ankfy1	8034
NM 027722	Nudt4	3231	NM 173181	Zc2hc1a	3361	NM 173433	Kdm4d	2662
NM 027724	Cage1	2871	NM 173182	Fndc3b	6875	NM 027808	Alpk1	4795
NM 009628	Adnp	4903	NM 027669	12-Sep	2576	NM 027810	Bbs7	2598
NM 027726	Dlg5	7809	NM 024241	Kif24	5616	NM 009675	Aoc3	4396
NM 173783	Mageb18	2160	NM 173185	Csnk1g1	7183	NM 009676	Aox1	4382
NM_173391	Tph2	2626	NM_001110148	Mgat1	3165	NM_173435	4930595M18Rik	2645
NM 027730	Rtdr1	1200	NM 001110149	Mgat1	2521	NM 027823	Arhgap42	5404
NM 173784	Ubtd2	3056	NM 173189	Mcph1	4636	NM 173437	Nav1	12767
NM 173392	Zfyve16	5451	NM 001110150	Mgat1	2695	NM 009679	Ap2m1	2057
NM 027740	Poc1b	2727	NM 027678	Zranb3	4224	NM 173441	Iws1	9623
NM 023596	Slc29a3	5240	NM 009610	Actg2	1341	NM 173443	Vcpip1	5192
NM 001110239	Acp1	3107	NM 001244692	St18	5913	NM 173444	Nbeal1	12108
NM 001110240	Slc24a2	10516	NM 173370	Cds1	3939	NM 009685	Apbb1	2730
NM 173399	Zbtb5	4360	NM 001244693	St18	5835	NM 173445	Rccd1	2340
NM 173400	Haus6	3719	NM 173376	Rbmx2	1743	NM 027836	Ms4a7	1336
NM_001110250	Gm14819	894	NM_027702	4933421I07Rik	1373	NM_009686	Apbb2	6671
NM 027756	Mfap3l	6337	NM 001110202	Trim9	4458	NM 027839	Ceacam20	2897
NM 009651	Akap4	2855	NM 027706	Nbas	7256	NM 173450	Rpusd2	5135
NM 001110254	Zfp945	6497	NM 173382	Proser1	4322	NM 009691	Aplp2	3490
NM 024242	Riok1	2744	NM 001110209	Lnp	8775	NM 173451	Arsj	3690
NM 173413	Rab8b	4751	NM 001244936	Gtf2ird1	4321	NM 001110778	Adam11	5129
NM_173414	Lancl3	3920	NM_001110216	Cbx5	8902	NM_001251890	LOC100503280	1693
NM 001110300	Ap1m2	1738	NM 027712	Dlgap1	4289	NM 173868	St18	5725
NM 009657	Aldoc	1702	NM 001110218	Ppm1h	6413	NM 009707	Arhgap6	5515
NM 001110309	Zfp426	3419	NM 027722	Nudt4	3231	NM 027865	Tmem25	2354
NM 173419	Dleu7	1348	NM 173388	Slc43a2	6915	NM 027866	Colec11	1383
NM 009661	Alox8	3273	NM 027725	Daw1	1371	NM 174852	Phf12	4412
NM 173423	Fem1c	2866	NM 001110227	Kcnj13	1349	NM 001110832	Nfya	3728
NM 173424	Zbtb37	3883	NM 173783	Mageb18	2160	NM 027870	Armcx3	3385
NM 001110315	Kif1a	8233	NM 027727	Dym	2471	NM 174868	Fam73a	5355
NM 001110316	Zscan4f	1829	NM 173391	Tph2	2626	NM 027875	Sydel	3288
NM 173428	Sspo	15586	NM 009634	Adsl	3712	NM 027881	Osbpl3	6497
NM_027801	2610015P09Rik	3643	NM_027733	Spata24	726	NM_174876	Impg2	6992
NM 031158	Ank1	8179	NM 173392	Zfyve16	5451	NM 027882	Cic	6104
NM 027802	Obox1	1217	NM 023595	Dut	2358	NM 009726	Atp7a	8195
NM 001110328	Fiz1	2672	NM 001110239	Acp1	3107	NM 027884	Tns1	9966
NM 173432	Pskh1	3317	NM 009643	Ahnak	18100	NM 001111017	Serac1	5630
NM 001110329	Fiz1	2615	NM 173400	Haus6	3719	NM 174990	Gimap4	1814
NM 001110330	Fiz1	2545	NM 173401	Fbxo44	1863	NM 027892	Ppp1r12a	3403
NM 027807	Cul5	6023	NM 027756	Mfap3l	6337	NM 027893	Pvrl4	3509
NM 009671	Ankfy1	8034	NM 027758	Tbc1d9	5149	NM 001111026	Runx1t1	7324
NM 027816	Cyp2u1	2331	NM 173405	Amz1	2685	NM 001111027	Runx1t1	7518
NM_173434	9930111J21Rik2	2403	NM_027762	Tchhl1	2066	NM_001111028	Kctd9	3324
NM 009678	Ap1m2	1732	NM 001110265	Ttk	2711	NM 027897	Rhpn2	3439
NM 173437	Nav1	12767	NM 173408	Dcun1d3	5637	NM 027901	Gtf3c2	4551
NM 027828	Fam110c	2684	NM 173413	Rab8b	4751	NM 009738	Bche	6209
NM 009681	Ap3s1	1479	NM 173414	Lancl3	3920	NM 175002	Mmgt2	1883
NM 173441	Iws1	9623	NM 009658	Akr1b3	1402	NM 027905	Selo	2360
NM_027834	9130008F23Rik	2159	NM_023598	Arid5b	4494	NM_001111048	Fga	2652
NM 173443	Vcpip1	5192	NM 173862	Fam83a	2089	NM 027908	Amdhd1	2421
NM 009686	Apbb2	6671	NM 173423	Fem1c	2866	NM 027909	C2cd2l	4334
NM 173449	Pp2d1	2476	NM 027787	Rfx2	3353	NM 175013	Pgm5	7981
NM 173450	Rpusd2	5135	NM 173425	Fam124b	2862	NM 001111058	Cd33	2571

NM 009691	Aplp2	3490	NM 001110315	Kif1a	8233	NM 027913	Vwce	3540
NM_009694	Apobec2	1218	NM_027793	Amtn	1022	NM_175017	4933427D06Rik	2621
NM 173451	Arsj	3690	NM 001110316	Zscan4f	1829	NM 027915	Ap2b1	5369
NM 174865	Klk15	765	NM 173428	Sspo	15586	NM 175021	Samd4b	4391
NM 001110778	Adam11	5129	NM 031158	Ank1	8179	NM 009759	Bmx	3005
NM 009698	Aprt	866	NM 173431	Rpgrip1l	6969	NM 175025	Atp2c1	4889
NM 001110783	Ank1	8283	NM 009676	Aox1	4382	NM 001111076	Zfp182	5944
NM 173868	St18	5725	NM 027817	Grap	1599	NM 175026	Pyhin1	3497
NM_027854	Tmem248	3669	NM_173435	4930595M18Rik	2645	NM_175027	Fancb	2543
NM 009707	Arhgap6	5515	NM 023605	Fbxo9	2172	NM 175028	Adnp2	4188
NM 174848	Crybg3	6544	NM 173437	Nav1	12767	NM 027924	Pdgfd	1795
NM 027868	Slc41a3	2272	NM 009679	Ap2m1	2057	NM 009766	Brs3	2772
NM 001110831	Dnpep	1930	NM 009681	Ap3s1	1479	NM 001111106	Uba3	2401
NM 009715	Atf2	4203	NM 173441	Iws1	9623	NM 009768	Bsg	1752
NM 174868	Fam73a	5355	NM 009682	Ap3s2	5823	NM 001111107	Zfp322a	4919
NM 027873	Ubiad1	2970	NM 173444	Nbeal1	12108	NM 175032	Galntl6	5103
NM 027874	Csnk1d	3749	NM 009685	Apbb1	2730	NM 009770	Btg3	1389
NM 027878	Dram1	2807	NM 173863	Crtc3	5110	NM 001252067	Gga3	3469
NM 009726	Atp7a	8195	NM 173451	Arsj	3690	NM 001252070	Dnah7a	12272
NM 009728	Atp10a	5469	NM 173866	Gpt2	3596	NM 001252071	Greb1	7868
NM 027886	Stk11ip	4474	NM 001110783	Ank1	8283	NM 001252074	Nrxn3	5157
NM_027889	Vps11	3194	NM_173868	St18	5725	NM_027939	1700006A11Rik	1983
NM 001111017	Serac1	5630	NM 027853	Mettl7b	1227	NM 001252081	Zswim8	6073
NM 009730	Atrn	8740	NM 024243	Fuca1	2523	NM 001252082	Zswim8	5992
NM 027893	Pvrl4	3509	NM 009703	Araf	2405	NM 001111121	Ccdc6	5511
NM 001111026	Runx1t1	7324	NM 001110807	Capn12	2828	NM 027941	Lrrc34	1978
NM_027895	Ulk3	2826	NM_001110826	Ddx6	6065	NM_027944	1700007B14Rik	2044
NM 001111027	Runx1t1	7518	NM 027865	Tmem25	2354	NM 001111267	Ncoa7	2783
NM 001111028	Kctd9	3324	NM 009715	Atf2	4203	NM 175090	Slc31a1	3764
NM 027897	Rhpn2	3439	NM 174868	Fam73a	5355	NM 175091	Tnks	6444
NM 174993	Fmr1nb	958	NM 027875	Syde1	3288	NM 009786	Cacybp	2115
NM 001111030	Acvr1c	9033	NM 027881	Osbpl3	6497	NM 001111289	Caprin1	6141
NM 027901	Gtf3c2	4551	NM 174876	Impg2	6992	NM 001111290	Caprin1	6114
NM 009738	Bche	6209	NM 009726	Atp7a	8195	NM 009793	Camk4	12331
NM 175002	Mmgt2	1883	NM 027883	Dhx34	3801	NM 175106	Tmem177	3302
NM_009742	Bcl2a1a	796	NM_027885	Smug1	3611	NM_175107	2310022A10Rik	2653
NM 175013	Pgm5	7981	NM 009730	Atrn	8740	NM 175111	Hspbap1	4606
NM 009753	Bicd1	9667	NM 027891	Lrwd1	2201	NM 175112	Rae1	1755
NM 027914	Bbs10	2753	NM 009731	Akr1b7	1248	NM 001111314	Ngef	3127
NM 175021	Samd4b	4391	NM 027897	Rhpn2	3439	NM 027973	Cenpu	2467
NM 175023	Rbbp6	2272	NM 027898	Gramd1a	2681	NM 175122	Rab39b	3402
NM 009759	Bmx	3005	NM 001111030	Acvr1c	9033	NM 027976	Acsl5	3167
NM 001111076	Zfp182	5944	NM 027901	Gtf3c2	4551	NM 001252132	Lrch1	4646
NM_175027	Fancb	2543	NM_009738	Bche	6209	NM_027980	2310003H01Rik	3175
NM_027922	Ankle2	5256	NM_023623	Cyp2d40	1129	NM_027981	2310002L09Rik	1930
NM 027924	Pdgfd	1795	NM 001111048	Fga	2652	NM 001112714	Ralgapa1	8277
NM 009767	Chic1	7401	NM 027907	Etnppl	2704	NM 175134	Ankrd46	2480
NM 001111106	Uba3	2401	NM 027908	Amdhd1	2421	NM 175135	Eif2b3	3754
NM 009768	Bsg	1752	NM 009749	Bex2	902	NM 001112721	Ubr5	9773
NM 175032	Galntl6	5103	NM 027910	Klhdc3	2139	NM 175138	Dnaic1	2508
NM 001111110	Cmah	9747	NM 009753	Bicd1	9667	NM 027992	Tmem106b	6099
NM_009771	Btrc	6250	NM_175017	4933427D06Rik	2621	NM_027994	Cand1	8209
NM 001252067	Gga3	3469	NM 001111065	Reps1	2537	NM 001112739	Kcnc1	4336
NM 009772	Bub1	4334	NM 001111076	Zfp182	5944	NM 027996	Zswim8	6094

NM 001252070	Dnah7a	12272	NM 027920	8-Mar	4521	NM 027999	Haus5	2201
NM 001111118	Rad51ap2	3175	NM 175026	Pyhin1	3497	NM 009822	Runx1t1	7256
NM 001252074	Nrxn3	5157	NM 175027	Fancb	2543	NM 009823	Cbfa2t2	6097
NM 001252081	Zswim8	6073	NM 027922	Ankle2	5256	NM 175155	Sash1	7183
NM 001252082	Zswim8	5992	NM 175031	Stk36	5329	NM 024246	Tmem79	2337
NM 175086	Agtr1b	2147	NM 001111107	Zfp322a	4919	NM 178045	Rassf4	6370
NM 175088	Mdfic	3435	NM 175032	Galntl6	5103	NM 175158	Utp20	8831
NM 175089	Nek1	4264	NM 009773	Bub1b	3669	NM 001252207	Kcnj16	3718
NM 027946	Dcaf7	5765	NM 001252070	Dnah7a	12272	NM 001252209	Kcnj16	3685
NM 175091	Tnks	6444	NM 009774	Bub3	2055	NM 001252210	Kcnj16	3652
NM_027948	1700003E16Rik	2050	NM_001252071	Greb1	7868	NM_175162	Stox2	9316
NM 001111279	Wdfy1	4566	NM 001252074	Nrxn3	5157	NM 001252220	Nbr1	4866
NM_009791	Aspm	9866	NM_027939	1700006A11Rik	1983	NM_001252222	Nbr1	4571
NM 001111289	Caprin1	6141	NM 001252081	Zswim8	6073	NM 001252223	Nbr1	4576
NM 001111290	Caprin1	6114	NM 001252082	Zswim8	5992	NM 001113187	Khdc1b	2249
NM 009793	Camk4	12331	NM 001111121	Ccdc6	5511	NM 175164	Arhgap26	7899
NM 001111291	Caprin1	3548	NM 175088	Mdfic	3435	NM 028006	Tube1	2929
NM 001111292	Caprin1	3508	NM 175090	Slc31a1	3764	NM 009832	Ccnk	2632
NM_027964	Zswim2	2062	NM_175091	Tnks	6444	NM_175172	4930506M07Rik	3767
NM 001111311	Lrrfip1	3662	NM 175093	Trib3	2051	NM 175174	Klhl5	3422
NM_175112	Rae1	1755	NM_027952	1700008I05Rik	1776	NM_001113211	Tmem194	6001
NM 175115	Ikzf5	4440	NM 009791	Aspm	9866	NM 175175	Plekhf2	2956
NM 027974	Efhc1	2208	NM 009793	Camk4	12331	NM 001252316	Shmt2	2296
NM 175124	Lrrc28	2978	NM 175104	Fam53c	4475	NM 009846	Cd24a	1825
NM 001252132	Lrch1	4646	NM 175106	Tmem177	3302	NM 175179	Amer1	8476
NM 175127	Fbxo28	4932	NM 009796	Capn7	3562	NM 028026	Wdr73	2132
NM 009808	Casp12	2605	NM 009798	Capzb	1676	NM 175187	Tmem161b	2704
NM 001252153	Tmem106c	1473	NM 175121	Slc38a2	4645	NM 028027	Arhgef25	2261
NM 175134	Ankrd46	2480	NM 001252132	Lrch1	4646	NM 001252374	Nt5c3	1530
NM 175135	Eif2b3	3754	NM 175127	Fbxo28	4932	NM 001113350	Xkr5	2855
NM_001112721	Ubr5	9773	NM_027981	2310002L09Rik	1930	NM_175195	Sppl2b	2887
NM 027986	Cdadc1	2264	NM 001112705	Tlk2	5475	NM 001113362	Tbc1d14	4783
NM_001112731	C030039L03Rik	4648	NM_175132	Synpo2l	3922	NM_001113364	Tbc1d14	3897
NM_001112735	9930012K11Rik	2001	NM_027983	Krt33a	1679	NM_175213	Mei4	3061
NM 027992	Tmem106b	6099	NM 001112721	Ubr5	9773	NM 009869	Cdh9	2903
NM 175146	Tsr2	3805	NM 175138	Dnaic1	2508	NM 001113385	BC021785	3551
NM_175149	2310022B05Rik	3317	NM_175141	1810030007Rik	2572	NM_001252437	Mgrn1	3293
NM 027996	Zswim8	6094	NM 027992	Tmem106b	6099	NM 001113388	BC021785	2981
NM 001112796	Bicd1	9591	NM 175146	Tsr2	3805	NM 009874	Cdk7	7612
NM_009822	Runx1t1	7256	NM_027994	Cand1	8209	NM_175219	C130026I21Rik	1971
NM 001112805	Zfp78	4655	NM 027995	Paqr7	3727	NM 001252442	Phldb2	5655
NM 024246	Tmem79	2337	NM 001112744	Arhgef16	2717	NM 175224	Metap1	2549
NM 009824	Cbfa2t3	7659	NM 027996	Zswim8	6094	NM 001252443	Tfg	1801
NM 178045	Rassf4	6370	NM 001112796	Bicd1	9591	NM 175226	Rnf139	4348
NM 028003	Rpap3	2260	NM 175153	Vwa9	3117	NM 009879	Ift81	2957
NM 001113179	Bub1	4337	NM 001252193	Amz2	2997	NM 001113399	Zfp385b	2685
NM 001252207	Kcnj16	3718	NM 001112805	Zfp78	4655	NM 001252447	Vrk2	1745
NM 001252209	Kcnj16	3685	NM 024246	Tmem79	2337	NM 001113400	Zfp385b	2707
NM 001252210	Kcnj16	3652	NM 009824	Cbfa2t3	7659	NM 001113401	Eaf2	1888
NM 175162	Stox2	9316	NM 175162	Stox2	9316	NM 001252450	Cldn25	2187
NM 175164	Arhgap26	7899	NM 175164	Arhgap26	7899	NM 175229	Srrm2	8613
NM 028009	Rpusd1	1703	NM 028006	Tube1	2929	NM 001252451	Cldn25	2049
NM 009833	Ccnt1	2175	NM 009830	Ccne2	3023	NM 001113405	Eaf2	1530

NM 175168	Ptk7	4255	NM 028007	Itfg1	3355	NM 028058	Fundc1	1950
NM 175170	Pogk	7385	NM 009832	Ccnk	2632	NM 175234	Faxc	9342
NM 028012	Xrcc4	1557	NM 001252266	Ica1	1614	NM 001113412	Fggy	1904
NM 028013	Endod1	4470	NM 175170	Pogk	7385	NM 178371	Slc9a8	4429
NM 175171	Mast4	10671	NM 175171	Mast4	10671	NM 028071	Cotl1	1642
NM_001113211	Tmem194	6001	NM_175174	Klhl5	3422	NM_175250	2810007J24Rik	2147
NM 175175	Plekhf2	2956	NM 028018	Ist1	2352	NM 175251	Arid2	9029
NM 175179	Amer1	8476	NM 001252316	Shmt2	2296	NM 001252463	Mlst8	3366
NM 175180	Wdr44	4135	NM 001113246	Chn1	4050	NM 023645	Kdelc1	2324
NM 175187	Tmem161b	2704	NM 028026	Wdr73	2132	NM 001252464	Mlst8	3341
NM 009852	Cd6	3034	NM 175187	Tmem161b	2704	NM 001252465	Mlst8	3150
NM 028027	Arhgef25	2261	NM 001252372	Mybpc1	3941	NM 024444	Cyp4f18	1702
NM 001252347	Rundc3a	3046	NM 175189	Hepacam	3103	NM 009899	Clca1	3022
NM 001252372	Mybpc1	3941	NM 028030	Rbpms2	1956	NM 175263	Notum	2020
NM 028029	Dnmbp	6109	NM 001113355	Vps26a	3104	NM 001113423	Slain2	4787
NM 001113350	Xkr5	2855	NM 028035	Snx10	2567	NM 175265	Bora	2293
NM 001113355	Vps26a	3104	NM 001252395	Slc25a19	2650	NM 001113424	Amn1	1027
NM 175198	Prox2	2682	NM 001113362	Tbc1d14	4783	NM 023647	Nipa2	4023
NM 001113368	Ceacam2	3827	NM 001113364	Tbc1d14	3897	NM 028083	Chaf1b	1913
NM 001113369	Ceacam2	3287	NM 001113374	Mocs2	1767	NM 175271	Lpar4	4237
NM 028041	Ddx54	3956	NM 028042	Ercc8	2107	NM 028089	Cyp2c55	1989
NM 001113374	Mocs2	1767	NM 001113375	Mocs2	1671	NM 001113470	Ctdsp2	4823
NM 028042	Ercc8	2107	NM 001113385	BC021785	3551	NM 001252486	Usp33	4185
NM_175212	Tmem65	3644	NM_001252438	D16Ertd472e	6160	NM_028097	Tmem68	2605
NM_001113375	Mocs2	1671	NM_001252439	D16Ertd472e	5927	NM_024251	Aplf	3148
NM_009869	Cdh9	2903	NM_001252440	D16Ertd472e	5892	NM_028102	Ddhd2	4267
NM_175215	Lysmd4	2974	NM_175219	C130026I21Rik	1971	NM_009913	Ccr9	4324
NM 028049	Fbxo22	2117	NM 001252442	Phldb2	5655	NM 028106	Zbed3	1894
NM 009874	Cdk7	7612	NM 175224	Metap1	2549	NM 009914	Ccr3	3273
NM 175224	Metap1	2549	NM 001252444	Ppil2	2328	NM 175290	Nlrp4f	3527
NM 001252443	Tfg	1801	NM 001252445	Ppil2	1812	NM 009919	Cnih1	1484
NM 001252444	Ppil2	2328	NM 175226	Rnf139	4348	NM 028112	Sehl1	1783
NM 001252445	Ppil2	1812	NM 009879	Ift81	2957	NM 028119	Ddb2	1929
NM 175226	Rnf139	4348	NM 001252448	Strada	2543	NM 001113554	Nudcd1	3444
NM 001252447	Vrk2	1745	NM 028057	Cyb5r1	1626	NM 175308	Mob3c	2882
NM 001252448	Strada	2543	NM 175231	Whsc1	6933	NM 009928	Col15a1	5335
NM 001252450	Cldn25	2187	NM 028059	Zfp654	4835	NM 001252505	St6gal1	4177
NM 028057	Cyb5r1	1626	NM 175238	Rif1	8502	NM 009929	Col18a1	5058
NM 001252451	Cldn25	2049	NM 028065	Cnpy3	1898	NM 001252506	St6gal1	4044
NM 175231	Whsc1	6933	NM 175240	Fam187b	1485	NM 028127	Frmd6	4695
NM 028058	Fundc1	1950	NM 001252457	Ddx39b	1651	NM 001252515	Ehbp1	5053
NM 028059	Zfp654	4835	NM 175245	Mzt1	2256	NM 009939	Cops2	3332
NM 175234	Faxc	9342	NM 009894	Cideb	1186	NM 175331	Nt5dc3	5967
NM 001113412	Fggy	1904	NM 024444	Cyp4f18	1702	NM 028138	Thumpd2	1856
NM 001113413	Rnf13	2676	NM 028081	Lrif1	1890	NM 175335	Gtf2a1	5691
NM 028065	Cnpy3	1898	NM 001252472	Cd84	3520	NM 009947	Cpne6	2021
NM 175240	Fam187b	1485	NM 175263	Notum	2020	NM 028148	Scaf11	7724
NM 175247	Zfp28	4161	NM 001113424	Amn1	1027	NM 028149	Fbxl20	8607
NM 028074	Ddx42	4028	NM 175268	Fam53b	5148	NM 178050	Atl2	3394
NM 001252463	Mlst8	3366	NM 028089	Cyp2c55	1989	NM 009957	Pcdha7	5215
NM 023645	Kdelc1	2324	NM 175273	Fam219b	3006	NM 009959	Pcdha5	5206
NM 001252464	Mlst8	3341	NM 175274	Ttyh3	4677	NM 175358	Zdhhc15	5517
NM 001252465	Mlst8	3150	NM 181058	Zbtb20	2934	NM 009960	Pcdha11	5254
NM 024444	Cyp4f18	1702	NM 001252486	Usp33	4185	NM 009961	Pcdha10	5242
NM 028081	Lrif1	1890	NM 009913	Ccr9	4324	NM 001114131	Ppp4r1	3813
NM 028082	Cnot2	2693	NM 028105	Adck1	2314	NM 028162	Tbc1d5	5616
NM 023647	Nipa2	4023	NM 009914	Ccr3	3273	NM 009970	Csf2ra	1850

NM 028087	Gcnt3	4301	NM 178283	Asb13	2410	NM 001114311	Stox2	10467
NM 001252478	Slc30a6	2171	NM 175290	Nlrp4f	3527	NM 175370	Als2cr12	1579
NM 028095	Mettl10	1097	NM 175291	Dock10	7308	NM 028171	Ceacam13	946
NM_001252486	Usp33	4185	NM_175293	D630023F18Rik	2201	NM_001114312	4930506M07Rik	4081
NM 028097	Tmem68	2605	NM 175294	Nucks1	6096	NM 001252569	Serpina1a	1939
NM 009907	Cln3	2375	NM 028119	Ddb2	1929	NM 009981	Pcyt1a	4787
NM 024251	Aplf	3148	NM 028126	Strada	2432	NM 028182	Sh2d4a	2767
NM_175280	4930529M08Rik	1561	NM_175314	Adamts9	6056	NM_175380	Gpd1l	4393
NM 028102	Ddhd2	4267	NM 175316	Slco2b1	4381	NM 001252573	Slc35c2	2055
NM 175290	Nlrp4f	3527	NM 175317	Eftud1	3637	NM 001252574	Slc35c2	1990
NM 001252495	Rbbp8	3523	NM 009936	Col9a3	2846	NM 175383	B3gnt1	2035
NM 028110	Dennd2d	4048	NM 175329	Chchd10	948	NM 001252575	Slc35c2	1938
NM 175291	Dock10	7308	NM 175331	Nt5dc3	5967	NM 181732	Aida	3293
NM 001113532	Wtap	4919	NM 028137	Msantd3	1624	NM 001114385	Chrdl1	4060
NM 028115	Trub1	3795	NM 028138	Thumpd2	1856	NM 175386	Lhfp	2107
NM 175294	Nucks1	6096	NM 001252530	Slco2b1	4635	NM 028190	Luc7l	4977
NM 178047	Prom2	4224	NM 001252531	Slco2b1	4578	NM 001114399	Zmym4	7022
NM 175300	Anapc2	3021	NM 009947	Cpne6	2021	NM 028191	Cyp2c65	1941
NM 001113554	Nudcd1	3444	NM 028148	Scaf11	7724	NM 001252580	Usp8	4210
NM 175308	Mob3c	2882	NM 175347	Srl	4978	NM 175388	Rnf169	7157
NM 028122	Slc14a1	3675	NM 001252539	Cyp4a31	2447	NM 028194	Fryl	11488
NM 001113562	Cutc	1308	NM 028149	Fbxl20	8607	NM 001114609	Ino80d	13529
NM_175312	B630005N14Rik	4147	NM_001114096	Smarcc2	4715	NM_028198	Xpo5	3709
NM 028126	Strada	2432	NM 001114097	Smarcc2	4964	NM 001114611	Stxbp5l	11821
NM_001252508	D430019H16Rik	5806	NM_028152	Mms19	3965	NM_178164	Ptbp3	6855
NM 028127	Frmd6	4695	NM 175356	Pi4kb	3692	NM 001114612	Stxbp5l	11992
NM 175314	Adamts9	6056	NM 175358	Zdhhc15	5517	NM 001114613	Stxbp5l	11893
NM 001252515	Ehbp1	5053	NM 009960	Pcdha11	5254	NM 028203	Wdr89	2981
NM_028129	2610020H08Rik	2008	NM_175752	Chn1	3862	NM_001252615	Fut8	2086
NM 028130	Zfp157	5304	NM 001252555	Gstz1	1986	NM 010001	Cyp2c37	1824
NM 175318	Spty2d1	5240	NM 001114131	Ppp4r1	3813	NM 175731	Acer1	2429
NM_175319	Papl	1923	NM_028166	1600014C10Rik	3165	NM_001252620	Spock3	3112
NM 009939	Cops2	3332	NM 178051	Mterfd2	3315	NM 001252621	Spock3	3103
NM 175331	Nt5dc3	5967	NM 001114311	Stox2	10467	NM 028220	Wdr17	4170
NM 009940	Coq7	871	NM 175370	Als2cr12	1579	NM 175408	Tmem139	1148
NM 001252525	Cpeb1	3141	NM 028173	Tram1	2888	NM 177993	Hbp1	3045
NM 028137	Msantd3	1624	NM 001252566	Sez6l2	3773	NM 028228	Pinx1	1351
NM 001114087	Pdlim7	1014	NM 001252567	Sez6l2	3632	NM 028230	Shmt2	2305
NM 028149	Fbxl20	8607	NM 175375	Ankhd1	8251	NM 028231	Kcnmb2	2947
NM_175350	Catsperd	2565	NM_001252569	Serpina1a	1939	NM_010007	Cyp2j5	3651
NM 028153	Eml2	2312	NM 009980	Ctbp2	3047	NM 010008	Cyp2j6	3595
NM 001114131	Ppp4r1	3813	NM 175378	Clip4	2087	NM 028234	Rbm33	9501
NM 175363	Pphln1	3828	NM 028182	Sh2d4a	2767	NM 001114977	U2surp	7736
NM_028170	1700030K09Rik	2441	NM_175380	Gpd1l	4393	NM_001115010	Lin54	4596
NM 178051	Mterfd2	3315	NM 001114361	Eml4	5445	NM 028236	Ceacam18	1952
NM 001114311	Stox2	10467	NM 001114362	Eml4	5238	NM 175432	Tmem132c	5113
NM 175370	Als2cr12	1579	NM 028191	Cyp2c65	1941	NM 001115130	Zbtb44	8882
NM 028173	Tram1	2888	NM 001252582	Zfp410	2606	NM 001115132	Ncaph2	3340
NM 001252566	Sez6l2	3773	NM 001252583	Zfp410	2266	NM 023670	Igf2bp3	4366
NM 001114328	Ccpg1	3033	NM 024253	Nkg7	813	NM 001115149	Uspl1	3772
NM 001252567	Sez6l2	3632	NM 175391	Apol7c	2044	NM 001115150	Uspl1	3603
NM_009978	Cst8	703	NM_028195	Cyth4	2791	NM_175442	A630033H20Rik	3008

NM_028179	2200002D01Rik	522	NM_001114609	Ino80d	13529	NM_001115151	Uspl1	3103
NM_001252569	Serpina1a	1939	NM_028198	Xpo5	3709	NM_010026	Asap1	6281
NM 028182	Sh2d4a	2767	NM 178164	Ptbp3	6855	NM 175445	Rassf2	4825
NM_175381	2700081015Rik	5017	NM_175397	Sp110	1947	NM_028248	Tmem87b	4631
NM 001114361	Eml4	5445	NM 009995	Cyp21a1	1939	NM 175446	Zmat1	3520
NM 001114362	Eml4	5238	NM 028202	Klhl40	2455	NM 175455	Ankrd34b	3956
NM 001114385	Chrdl1	4060	NM 009997	Cyp2a4	1715	NM 010046	Dgat1	1776
NM 001114399	Zmym4	7022	NM 175401	Fbxw17	1832	NM 175456	Abra	1762
NM 001252582	Zfp410	2606	NM 001252615	Fut8	2086	NM 010048	Dgcr2	4099
NM 028193	Brf1	2755	NM 028208	Ptar1	2064	NM 175457	Mroh1	5269
NM 009990	Clip2	5013	NM 028209	Ttc4	2569	NM 028266	Col16a1	5222
NM_001252583	Zfp410	2266	NM_028211	2210016L21Rik	1350	NM_010055	Dlx3	2606
NM 024253	Nkg7	813	NM 175731	Acer1	2429	NM 175472	Zcchc11	6054
NM 001114545	Ksr2	6143	NM 180600	Ube2q2	2952	NM 010060	Dnah11	14072
NM_001114595	Ehbp1l1	3543	NM_001252623	Csgalnact1	4247	NM_028287	Zufsp	2364
NM 028194	Fryl	11488	NM 010004	Cyp2c40	1811	NM 175482	Usp28	4220
NM 001114596	Ehbp1l1	3579	NM 010005	Cyp2d10	1681	NM 028298	Zfp655	4072
NM 001114597	Ehbp1l1	6411	NM 175417	Adtrp	2440	NM 010074	Dpp4	5268
NM_175393	4930555G01Rik	2065	NM_010006	Cyp2d9	1651	NM_175489	Osbpl8	7152
NM 001114609	Ino80d	13529	NM 175418	Mybpc1	3924	NM 175494	Zfp367	3490
NM 175394	Wtap	4967	NM 028230	Shmt2	2305	NM 175499	Slitrk6	4243
NM 028199	Plxdc1	2901	NM 010007	Cyp2j5	3651	NM 010079	Dsg1a	5627
NM 175398	Fam212b	4701	NM 010008	Cyp2j6	3595	NM 028315	Dis3	3722
NM 010002	Cyp2c38	2869	NM 175421	Plekhb2	2794	NM 028316	Zfp444	4211
NM 175403	Mlec	5970	NM 028234	Rbm33	9501	NM 028319	Zfp518a	6840
NM 028221	Fam192a	1812	NM 010011	Cyp4a10	2096	NM 175501	Adamts12	6234
NM 177993	Hbp1	3045	NM 175425	C1qtnf7	1295	NM 028326	Zfp618	2868
NM 175418	Mybpc1	3924	NM 001252653	Ecm1	1897	NM 010085	Adam26a	2510
NM 010007	Cyp2j5	3651	NM 175428	Zbtb21	6011	NM 028339	Tmx1	2418
NM 175424	Prr9	1183	NM 001115010	Lin54	4596	NM 028340	Susd3	1328
NM 010015	Dad1	3506	NM 001115074	Gm101	2511	NM 010093	E2f3	5106
NM 001114977	U2surp	7736	NM 001252662	Dmtn	4254	NM 028343	Tmem135	3693
NM 001115010	Lin54	4596	NM 001252663	Dmtn	4193	NM 001253690	Patz1	3191
NM 175429	Kctd12b	4822	NM 010021	Dazl	2964	NM 001253691	Patz1	3210
NM 023668	Ndel1	2387	NM 175433	Zfp710	4569	NM 001253692	Elmod3	2220
NM 175431	Fam228b	2886	NM 001115130	Zbtb44	8882	NM 010097	Sparcl1	3132
NM 175432	Tmem132c	5113	NM 001252664	Dmtn	4086	NM 010099	Eda	4955
NM 010021	Dazl	2964	NM 001252665	Dmtn	3919	NM 175535	Arhgap20	6365
NM 001115130	Zbtb44	8882	NM 175436	Zfp526	3352	NM 010101	S1pr3	4484
NM 028245	Zfp131	2965	NM 001252666	Dmtn	3952	NM 001253702	St7l	5643
NM 023671	Clns1a	3395	NM 001115149	Uspl1	3772	NM 001253703	St7l	5505
NM 001115149	Uspl1	3772	NM 001115150	Uspl1	3603	NM 028365	Zfp839	3730
NM 001115150	Uspl1	3603	NM 001252684	Smc5	5772	NM 001253706	6-Sep	4737
NM 001252684	Smc5	5772	NM 001115151	Uspl1	3103	NM 028372	Mblac2	3881
NM 001115151	Uspl1	3103	NM 010026	Asap1	6281	NM 028385	Setd5	6470
NM 010026	Asap1	6281	NM 175443	Etnk2	2280	NM 028387	Macrod2	3552
NM 175443	Etnk2	2280	NM 001252685	Smc5	5559	NM 023689	Spock3	3226
NM 001252685	Smc5	5559	NM 175448	Clvs2	2500	NM 001253731	Ints9	2727
NM 175445	Rassf2	4825	NM 001253355	Hs3st5	3122	NM 181579	Pof1b	3909
NM 028248	Tmem87b	4631	NM 175450	Wdr18	2954	NM 001253751	Fdps	1478
NM 175449	Fam26f	1095	NM 028258	Dzip1l	3928	NM 001253752	Zmym5	5290
NM 010048	Dgcr2	4099	NM 001253356	Hs3st5	3063	NM 001253753	Zmym5	5360
NM 175464	Pkp4	4513	NM 175456	Abra	1762	NM 010128	Emp1	2721
NM 175470	Gpr61	3686	NM 028262	Setd3	2725	NM 001253754	Gpm6a	2980
NM 175472	Zcchc11	6054	NM 175457	Mroh1	5269	NM 001253755	Tdrd3	5762
NM 010060	Dnah11	14072	NM 028264	Tmem55a	2280	NM 001253756	Gpm6a	3158

NM 028283	Uaca	4428	NM 175462	Kcnt1	5893	NM 177547	Sgk3	5410
NM 010064	Dync1i2	2534	NM 175464	Pkp4	4513	NM 175645	Xylt1	3070
NM 175480	Zfp612	4902	NM 010060	Dnah11	14072	NM 175646	Txnl4b	1796
NM 175482	Usp28	4220	NM 028283	Uaca	4428	NM 001253783	Def8	3895
NM 028295	Pdia5	1823	NM 175476	Arhgap25	3366	NM 028416	Kremen2	2135
NM 028301	Anks3	2483	NM 028287	Zufsp	2364	NM 001253784	Def8	3873
NM 175489	Osbpl8	7152	NM 010069	Doc2a	2557	NM 175649	Tnfrsf26	2977
NM 175494	Zfp367	3490	NM 175483	Snx33	4564	NM 010143	Ephb3	4164
NM 175498	Pnma2	4518	NM 175484	Coro2b	3626	NM 028421	Zc3hav1	3784
NM 028308	Mob2	1456	NM 028298	Zfp655	4072	NM 028428	Fut11	3344
NM 175499	Slitrk6	4243	NM 028301	Anks3	2483	NM 001253817	Tmem184b	3378
NM 010084	Adam18	2361	NM 175494	Zfp367	3490	NM 001253819	Tmem184b	3357
NM 028319	Zfp518a	6840	NM 175498	Pnma2	4518	NM 001253820	Tmem184b	2881
NM 175512	Dhrs9	2817	NM 010079	Dsg1a	5627	NM 028450	Gulp1	2976
NM 010086	Adam24	2870	NM 010082	Adam28	3110	NM 001253831	Atp2c1	4683
NM 028335	Zfp248	3682	NM 028315	Dis3	3722	NM 001115153	Uspl1	3949
NM 001253392	Smarcad1	4637	NM 010084	Adam18	2361	NM 001253834	Atp2c1	4774
NM 175515	Intu	6387	NM 028319	Zfp518a	6840	NM 001253836	Atp2c1	2482
NM 175516	Lingo2	3669	NM 175501	Adamts12	6234	NM 001122594	Phlpp2	7854
NM 010094	Lefty1	1622	NM 175506	Adamts19	4666	NM 001253843	Prpf19	6218
NM_175533	5830411N06Rik	2897	NM_010085	Adam26a	2510	NM_001122595	A630033H20Rik	2997
NM 175535	Arhgap20	6365	NM 010086	Adam24	2870	NM 001253844	Prpf19	5811
NM_010101	S1pr3	4484	NM_028333	Angptl1	2208	NM_001122596	A630033H20Rik	3044
NM 001253702	St7l	5643	NM 010092	Dyrk1b	2404	NM 001122603	Fcgbp	7957
NM 001253703	St7l	5505	NM 175522	Elfn1	3835	NM 028474	Ptchd4	6890
NM 001253704	Wibg	1220	NM 001253690	Patz1	3191	NM 010178	Srsf10	3436
NM 001253705	Wibg	1324	NM 001253691	Patz1	3210	NM 010180	Fbln1	3359
NM 010103	Edil3	5328	NM 175526	Clec1a	3268	NM 001253885	Apbb1	2922
NM_028370	Pot1b	3499	NM_175528	E330009J07Rik	1951	NM_001253886	Apbb1	2916
NM 028372	Mblac2	3881	NM 010099	Eda	4955	NM 028480	Ceacam5	3248
NM 175542	Rttn	7280	NM 175537	Zbtb38	4091	NM 001253887	Apbb1	1922
NM 028376	Pfn4	1306	NM 010103	Edil3	5328	NM 001122662	Gm2016	1766
NM 175548	Lsamp	2467	NM 028372	Mblac2	3881	NM 001253888	Tssk4	1273
NM 028381	Ccdc94	1324	NM 175542	Rttn	7280	NM 001253889	Tssk4	1420
NM 028385	Setd5	6470	NM 028375	Cxx1c	965	NM 001122667	Mkl2	8281
NM 028387	Macrod2	3552	NM 010112	Efs	3571	NM 175667	Ankef1	2723
NM 028389	Tmem219	959	NM 028384	Ccdc13	2656	NM 001122675	Zcchc2	6681
NM 175562	Rab39	2971	NM 175554	Clspn	5011	NM 001122676	Zcchc2	5819
NM 001253738	Tpm3	2256	NM 028389	Tmem219	959	NM 175674	Vmn2r7	2484
NM 010122	Eif2b4	1752	NM 175558	Zfp446	4617	NM 001122680	Pvrl4	3434
NM 001253740	Tpm3	2256	NM 001253719	Fstl5	4560	NM 028500	Calr3	1670
NM 028399	Ccnt2	3514	NM 028392	Ppp2r2b	2079	NM 010193	Fem1b	6505
NM 175565	Cdv3	4051	NM 181579	Pof1b	3909	NM 001122685	Rhbdd1	3711
NM 181579	Pof1b	3909	NM 175628	A2m	4687	NM 010196	Fga	3373
NM 001253752	Zmym5	5290	NM 177547	Sgk3	5410	NM 175683	Dclre1c	4082
NM 001253753	Zmym5	5360	NM 175639	Wdr43	3450	NM 175692	Snhg11	5864
NM 001253754	Gpm6a	2980	NM 001253768	Ssx2ip	3706	NM 010199	Fgf12	4607
NM 001253755	Tdrd3	5762	NM 001253769	Ssx2ip	3602	NM 001122759	Pde7a	6298
NM 001253756	Gpm6a	3158	NM 028407	Ccser2	5587	NM 028527	Fam177a	3863
NM 177547	Sgk3	5410	NM 001253770	Ssx2ip	3451	NM 176917	Mettl4	2150
NM_001253757	Anp32e	3237	NM_175643	Adamts2	7265	NM_176921	6030419C18Rik	2139
NM 001253758	Anp32e	2904	NM 175646	Txnl4b	1796	NM 176922	Itga11	4937
NM 028402	Zfp493	3274	NM 001253783	Def8	3895	NM 176928	Brwd1	2284
NM 001253768	Ssx2ip	3706	NM 001253784	Def8	3873	NM 001254754	Echdc2	1255
NM 001253769	Ssx2ip	3602	NM 010143	Ephb3	4164	NM 176931	Syt15	1801
NM 028407	Ccser2	5587	NM 175651	Cnpy1	2281	NM 001122873	Ubl7	1382
NM 001253770	Ssx2ip	3451	NM 028421	Zc3hav1	3784	NM 176951	Xkr5	3195

NM_001253777	1110017D15Rik	1119	NM_028428	Fut11	3344	NM_001122896	Hp1bp3	4654
NM_001253778	1110017D15Rik	726	NM_028443	Fam101a	1667	NM_176953	Lig4	4081
NM_010139	Epha2	3948	NM_001253814	Ankle2	5259	NM_176957	Nckap5	2999
NM_175645	Xylt1	3070	NM_001253817	Tmem184b	3378	NM_001122958	Rad54l	2763
NM_175649	Tnfrsf26	2977	NM_001253819	Tmem184b	3357	NM_001122959	Rad54l	2742
NM_028420	Camk2n2	1299	NM_001253820	Tmem184b	2881	NM_010230	Fmn1	11925
NM_001253814	Ankle2	5259	NM_028450	Gulp1	2976	NM_010231	Fmo1	2349
NM_001253817	Tmem184b	3378	NM_010156	Samd9l	5344	NM_176968	Nt5dc1	2716
NM_001253819	Tmem184b	3357	NM_028453	Otud3	1670	NM_001122993	B3galt5	5281
NM_001253820	Tmem184b	2881	NM_001115153	Uspl1	3949	NM_028550	1700074P13Rik	1647
NM_010156	Samd9l	5344	NM_001253843	Prpf19	6218	NM_176972	Usp37	7196
NM_001115153	Uspl1	3949	NM_001253844	Prpf19	5811	NM_001122998	Tiam2	6144
NM_001122594	Phlpp2	7854	NM_010161	Evi2a	1503	NM_001123327	Qser1	8975
NM_001253843	Prpf19	6218	NM_028460	Pear1	4501	NM_176980	Ankar	4573
NM_001253844	Prpf19	5811	NM_001122603	Fcgbp	7957	NM_028572	Vgll3	3331
NM_028460	Pear1	4501	NM_001122639	Galnt9	1561	NM_001123370	9030025P20Rik	2120
NM_010163	Ext2	2875	NM_028474	Ptchd4	6890	NM_023707	1810009J06Rik	856
NM_001122603	Fcgbp	7957	NM_028477	Prl8a1	864	NM_001123372	Gm3435	2061
NM_028462	Fezf1	2308	NM_001253883	Pkm	2432	NM_010243	Fut9	11990
NM_010167	Eya4	4099	NM_001253885	Apbb1	2922	NM_010248	Gab2	6008
NM_001122640	Arhgap17	3551	NM_001253886	Apbb1	2916	NM_177002	Slc22a30	1997
NM_001122641	Arhgap17	3269	NM_001253887	Apbb1	1922	NM_010252	Gabrg1	4771
NM_001122642	Arhgap17	3317	NM_010183	Fbrs	2650	NM_180956	Ndrg3	2683
NM_028474	Ptchd4	6890	NM_028483	Cib4	768	NM_001256005	Gbp4	4538
NM_001122643	Arhgap17	3318	NM_028487	Gpbp1	3624	NM_010256	Gart	3329
NM_010178	Srsf10	3436	NM_001253892	Slc4a2	4006	NM_181316	Bbs9	3382
NM_010180	Fbln1	3359	NM_024260	Ccdc132	3780	NM_001256033	Otud4	7327
NM_010181	Fbn2	10480	NM_001253904	Ncor2	8697	NM_177025	Cobll1	5042
NM_028481	Ccdc18	4682	NM_001253905	Ncor2	8146	NM_028607	Bloc1s2	910
NM_010183	Fbrs	2650	NM_010196	Fga	3373	NM_177028	Oacyl	3431
NM_001122675	Zcchc2	6681	NM_175683	Dclre1c	4082	NM_010264	Nr6a1	6185
NM_175668	Gpr4	2884	NM_175684	Fchsd1	4227	NM_028610	Dppa4	1534
NM_001122676	Zcchc2	5819	NM_028518	Col20a1	4189	NM_001126324	Gm13083	2464
NM_175669	Gpr82	2497	NM_175692	Snhg11	5864	NM_001256048	Mamld1	3346
NM_175674	Vmn2r7	2484	NM_028527	Fam177a	3863	NM_010267	Gdap1	3954
NM_001122680	Pvrl4	3434	NM_028529	Nipsnap3a	1099	NM_010273	Gdi1	2657
NM_028493	Rhobtb3	4937	NM_028532	Tepp	673	NM_010282	Ggps1	2387
NM_001253904	Ncor2	8697	NM_176919	Ppm1h	2947	NM_022305	B4galt1	4535
NM_001253905	Ncor2	8146	NM_024261	1700052N19Rik	2318	NM_178061	Mob3b	6030
NM_001122685	Rhbdd1	3711	NM_001122818	Pnpla6	4502	NM_028629	Kprp	2738
NM_028509	1700034J05Rik	2054	NM_176923	Wdr59	5162	NM_177066	Tnni3k	3042
NM_175683	Dclre1c	4082	NM_180974	Foxn2	5101	NM_028639	Ttc7	4615
NM_028518	Col20a1	4189	NM_001122832	Eps15l1	3397	NM_177070	Fbxw16	1524
NM_175692	Snhg11	5864	NM_001122843	Tnpo2	4884	NM_010295	Gclc	3442
NM_010199	Fgf12	4607	NM_176940	Nwd1	7732	NM_001202500	Armcx4	9014
NM_001122758	Pcdh7	7741	NM_010215	Il4i1	2059	NM_177076	Fbxl13	2556
NM_001122759	Pde7a	6298	NM_176953	Lig4	4081	NM_028654	Tex36	945
NM_176919	Ppm1h	2947	NM_001254953	Ankrd66	1321	NM_177078	Adrbk2	6578
NM_176920	Lrtm1	7318	NM_028543	Zfp763	3385	NM_001256065	Gm5141	3292
NM_024261	1700052N19Rik	2318	NM_001122958	Rad54l	2763	NM_028658	Ppp1r21	3151
NM_001122818	Pnpla6	4502	NM_028544	Rasip1	3169	NM_001127353	Adgb	5218
NM_176923	Wdr59	5162	NM_001122959	Rad54l	2742	NM_028660	Klk9	1475
NM_180974	Foxn2	5101	NM_001122963	Gpbp1	3564	NM_177086	Zmat4	4206

NM 001122830	Klhl26	2886	NM 001255993	Ubox5	3900	NM 028664	Ankrd45	3723
NM_001122832	Eps15l1	3397	NM_010230	Fmn1	11925	NM_028667	D3Ertd751e	3574
NM 176940	Nwd1	7732	NM 001255994	Ubox5	3753	NM 010312	Gnb2	1593
NM 176951	Xkr5	3195	NM 010232	Fmo5	5186	NM 001127576	Gm1564	4577
NM 001254947	BC035044	580	NM 176966	Fsd1l	2198	NM 001127686	Hbb-bh2	559
NM_001122896	Hp1bp3	4654	NM_028546	1700066M21Rik	3008	NM_010317	Gng4	2976
NM_176952	6430573F11Rik	1825	NM_001122992	Gmeb1	6581	NM_177123	Spef2	2995
NM 176953	Lig4	4081	NM 001122998	Tiam2	6144	NM 028696	Nabp1	2838
NM_001254951	Zfp850	6546	NM_028562	1700080E11Rik	871	NM_177130	Glt28d2	2737
NM 001254953	Ankrd66	1321	NM 001123327	Qser1	8975	NM 001128086	Anks1b	7002
NM 176957	Nckap5	2999	NM 028576	Camkmt	1439	NM 001128091	Aadacl2	1590
NM_028538	1700049G17Rik	5159	NM_001123371	Pnp2	1223	NM_177548	Clasp1	7623
NM 001122958	Rad54l	2763	NM 176987	Simc1	4811	NM 001128096	Atp13a3	7310
NM 001122959	Rad54l	2742	NM 010243	Fut9	11990	NM 028713	Rftn2	3689
NM 001255993	Ubox5	3900	NM 176998	Sybu	3255	NM 001128151	Cecr2	9213
NM 001255994	Ubox5	3753	NM 176999	Atp10b	6926	NM 028727	Nol9	2280
NM_010232	Fmo5	5186	NM_177000	C130050018Rik	2006	NM_028728	Nfam1	3819
NM_001122989	Cdc14b	5595	NM_010248	Gab2	6008	NM_177173	A830018L16Rik	2796
NM 001122992	Gmeb1	6581	NM 177002	Slc22a30	1997	NM 028731	Esyt2	5556
NM 176971	Rab9b	3761	NM 023712	Spns1	2706	NM 177178	Lmbrd2	3046
NM 176972	Usp37	7196	NM 010252	Gabrg1	4771	NM 001128609	Dedd	2733
NM_176976	5830418K08Rik	7380	NM_180956	Ndrg3	2683	NM_177184	Vps13c	11523
NM 176980	Ankar	4573	NM 001256005	Gbp4	4538	NM 001128625	Gm364	2167
NM 176987	Simc1	4811	NM 001126318	Gm13011	958	NM 001128634	Tppp2	747
NM_010243	Fut9	11990	NM_181316	Bbs9	3382	NM_028737	4931406B18Rik	3970
NM 001126045	Smok3a	2160	NM 001256033	Otud4	7327	NM 001256122	Limch1	5904
NM 010248	Gab2	6008	NM 178165	Fcrl1	1982	NM 001256130	Nipa2	3269
NM 177002	Slc22a30	1997	NM 010259	Gbp2b	2820	NM 001256131	Nipa2	3148
NM 023712	Spns1	2706	NM 177026	Tmcc3	5470	NM 001256132	Nipa2	3073
NM 010252	Gabrg1	4771	NM 010264	Nr6a1	6185	NM 001256133	Nipa2	2723
NM 001126182	Naip2	4782	NM 177030	Dock6	6577	NM 177191	Sycp2	5688
NM 001126314	Mboat4	1839	NM 010266	Gda	5417	NM 177192	Dennd5b	9833
NM 010264	Nr6a1	6185	NM 010267	Gdap1	3954	NM 177197	Idi2	2243
NM 001126324	Gm13083	2464	NM 177036	Ceacam19	3361	NM 028758	Gga2	4891
NM 177034	Apba1	6649	NM 001126488	Tex28	1420	NM 028759	Dcaf6	3306
NM 010269	Gdap2	2934	NM 177045	Cc2d1b	3286	NM 177208	Dopey1	9124
NM 177038	Trappc8	5178	NM 177054	Casc4	4116	NM 177215	Ocrl	5198
NM_001126488	Tex28	1420	NM_177055	A630001G21Rik	1936	NM_010390	H2-Q1	2044
NM 001256052	Rslcan18	2981	NM 181317	Kcns2	5473	NM 177224	Chd9	11517
NM 001127169	Tceal7	971	NM 028629	Kprp	2738	NM 028768	Armc8	4628
NM 177054	Casc4	4116	NM 177066	Tnni3k	3042	NM 010392	H2-Q2	1100
NM_177055	A630001G21Rik	1936	NM_028636	Man2c1	3221	NM_177230	Ccdc158	3636
NM 181317	Kcns2	5473	NM 001202500	Armcx4	9014	NM 177233	Fam19a4	2182
NM 177068	Olfml2b	3105	NM 028651	Tmtc4	3390	NM 177235	Bend6	2318
NM 028639	Ttc7	4615	NM 028654	Tex36	945	NM 010400	-	4265
NM 001202500	Armcx4	9014	NM 177078	Adrbk2	6578	NM 028776	Scyl3	4386
NM 010299	Gm2a	4257	NM 001127348	Snx10	2464	NM 177240	Trappc11	4338
NM 177078	Adrbk2	6578	NM 001127349	Snx10	2469	NM 028778	Nuak2	3108
NM 028658	Ppp1r21	3151	NM 028659	Eif3k	866	NM 177249	Usp47	5600
NM 001127353	Adgb	5218	NM 001127353	Adgb	5218	NM 028789	Unkl	3414
NM 001127355	Eif2b4	1897	NM 177086	Zmat4	4206	NM 010417	Heph	4693

NM 001127356	Eif2b4	2119	NM 028662	Slc35b2	2138	NM 010418	Herc2	15363
NM 177093	Lrrc58	8592	NM 177088	Cep95	2737	NM 001130868	Kars	2223
NM 177099	Lefty2	2534	NM 177089	Tacc1	7805	NM 177270	Cdkl2	3990
NM 177103	Senp5	6352	NM 001127576	Gm1564	4577	NM 177278	L3mbtl4	4570
NM 177111	Ccdc66	2951	NM 028672	Fam161a	1953	NM 028803	Gbe1	2988
NM 028687	Ccdc175	2630	NM 177103	Senp5	6352	NM 177282	Mical2	6689
NM 010322	Gnpat	2984	NM 177111	Ccdc66	2951	NM 001134226	Ssxb2	780
NM 001128084	Arhgap21	6974	NM 010317	Gng4	2976	NM 028804	Ccdc3	2769
NM 028708	Jakmip3	5987	NM 177123	Spef2	2995	NM 010433	Hipk2	4259
NM 177139	Lypd6	3495	NM 028687	Ccdc175	2630	NM 177284	Nrxn1	9004
NM 010330	Emb	2622	NM 028696	Nabp1	2838	NM 010434	Hipk3	7497
NM 001128093	Siah3	905	NM 010322	Gnpat	2984	NM 001134300	Nol4l	6416
NM 177548	Clasp1	7623	NM 177128	Iqcb1	2284	NM 010437	Hivep2	9788
NM 028711	Slc25a27	2687	NM 001128091	Aadacl2	1590	NM 028815	Cep97	7904
NM 028715	Fcho1	3160	NM 177548	Clasp1	7623	NM 028816	Xpo6	4493
NM_177151	Vps13b	13380	NM_001128096	Atp13a3	7310	NM_028820	1700017B05Rik	4934
NM 001256085	Rnf6	3154	NM 001128132	Cd200r3	1622	NM 028821	Dnal1	5792
NM 001256086	Rnf6	3225	NM 001128133	Cd200r3	1403	NM 001134465	Dennd6a	6666
NM_010343	Gpx5	1629	NM_001128145	5830411N06Rik	2991	NM_177311	Serac1	5540
NM 001128151	Cecr2	9213	NM 177151	Vps13b	13380	NM 001134480	Plcxd2	7352
NM 001256087	Rnf6	3286	NM 010340	Gpr50	1801	NM 028829	Paqr8	4438
NM_010346	Grb7	2413	NM_001128146	5830411N06Rik	3342	NM_175749	Nup153	6021
NM_177162	Tmprss11g	2872	NM_001128151	Cecr2	9213	NM_175930	Rapgef5	3987
NM 001128307	Dock9	8221	NM 028719	Cpne4	3670	NM 175937	Cpeb2	6823
NM 001128308	Dock9	8182	NM 028720	Glyr1	3347	NM 175938	Btn2a2	2983
NM 177167	Ppm1e	6315	NM 010347	Aes	1488	NM 028844	Aven	1432
NM 177171	Heatr5a	7761	NM 028721	Nphp3	5894	NM 175939	Adam29	2742
NM_177173	A830018L16Rik	2796	NM_177162	Tmprss11g	2872	NM_010475	Hsd17b1	1317
NM 028731	Esyt2	5556	NM 028722	Msl1	4358	NM 176836	Fam76b	3672
NM 177178	Lmbrd2	3046	NM 001128307	Dock9	8221	NM 001134829	Lpgat1	7069
NM 177184	Vps13c	11523	NM 001128308	Dock9	8182	NM 176838	Esrp2	2686
NM 001128625	Gm364	2167	NM 028724	Rin2	4668	NM 001134902	AU019823	3362
NM 028735	Ttc21a	4182	NM 177167	Ppm1e	6315	NM 178936	Tmem56	6224
NM 177186	Slc35e2	6333	NM 177171	Heatr5a	7761	NM 001135019	Zfp266	6294
NM_001256122	Limch1	5904	NM_177173	A830018L16Rik	2796	NM_028864	Zc3hav1	3396
NM 001130008	Gspt1	6853	NM 028731	Esyt2	5556	NM 001135115	Gm12250	2702
NM_028744	Pi4k2b	3074	NM_177184	Vps13c	11523	NM_001256259	4930524N10Rik	919
NM 001256130	Nipa2	3269	NM 028735	Ttc21a	4182	NM 028871	Hnrnpr	8025
NM 001256131	Nipa2	3148	NM 001129787	Igsf9b	4239	NM 001135151	Slc39a14	4927
NM 001256132	Nipa2	3073	NM 001256122	Limch1	5904	NM 001135152	Slc39a14	4893
NM 001256133	Nipa2	2723	NM 023729	Asz1	1740	NM 001135192	Asap2	5686
NM 028749	Npl	1403	NM 001130008	Gspt1	6853	NM 028875	Xrcc3	2422
NM_177187	D5Ertd577e	2501	NM_028744	Pi4k2b	3074	NM_176850	Bptf	11506
NM 001130163	Oxr1	4176	NM 028748	Paqr5	3500	NM 001135577	Smim13	4499
NM 010367	Magi1	7604	NM 001256142	Fsbp	4572	NM 177321	Mia2	1628
NM 001130164	Oxr1	2529	NM 177191	Sycp2	5688	NM 028883	Tldc1	2574
NM 177192	Dennd5b	9833	NM 028756	Slc35a5	4200	NM 177323	Rint1	3311
NM 028755	Arpp21	3328	NM 177197	Idi2	2243	NM 177324	Sbf2	7163
NM_001130165	Oxr1	4271	NM_010371	Gzmc	934	NM_028890	4931414P19Rik	2585
NM 028756	Slc35a5	4200	NM 177200	Svop1	2382	NM 001136057	Cpne6	2218
NM 010369	Gypa	1827	NM 028759	Dcaf6	3306	NM 028901	Myo18b	8280
NM 001130166	Oxr1	4616	NM 010376	H13	5202	NM 177338	Hmbox1	2931

NM_177197	Idi2	2243	NM_177205	9430015G10Rik	2660	NM_177340	Synpo	5012
NM 028759	Dcaf6	3306	NM 177208	Dopey1	9124	NM 177341	Trpm3	5157
NM 177204	Strip2	5370	NM 028762	Rbm19	4008	NM 001136065	Hipk2	3972
NM_177205	9430015G10Rik	2660	NM_177214	Snrnp200	6740	NM_010498	Ids	4988
NM 028761	Parn	2902	NM 010381	H2-Ea-ps	887	NM 001136070	Eif2d	1976
NM 177208	Dopey1	9124	NM 010382	H2-Eb1	1669	NM 177346	Gpr149	3336
NM_177214	Snrnp200	6740	NM_001256161	9430069I07Rik	572	NM_028910	Olfr701	3063
NM 028765	Acox1	2876	NM 177224	Chd9	11517	NM 001136076	P4ha2	2278
NM 177224	Chd9	11517	NM 001130194	Best2	1971	NM 177353	Slc9a7	2469
NM 028769	Syvn1	3470	NM 028770	Krt80	1774	NM 028920	Hyal6	3452
NM 028774	Rnf6	3360	NM 177233	Fam19a4	2182	NM 028924	Tc2n	5665
NM 028775	Cyp2s1	2621	NM 028776	Scyl3	4386	NM 001136089	Anxa10	1840
NM 028778	Nuak2	3108	NM 028777	Sec14l1	2885	NM 028932	Eaf1	4238
NM 177243	Slc26a9	3640	NM 177240	Trappc11	4338	NM 177358	Zfp945	6618
NM 001130456	Sema6b	3736	NM 028783	Robo4	3689	NM 028935	Zfp558	3839
NM 028784	F13a1	3879	NM 177249	Usp47	5600	NM 028938	Lrriq3	2650
NM 028785	Dock8	7810	NM 028785	Dock8	7810	NM 023755	Tfcp2l1	9285
NM 001130479	Nucb2	1703	NM 001130479	Nucb2	1703	NM 001136226	Utp14b	3702
NM 177261	Kndc1	7320	NM 028791	Cmtr1	3754	NM 177367	Gemin4	3448
NM 028791	Cmtr1	3754	NM 001130693	Gucy2d	3633	NM 028943	Sgms2	5791
NM 010417	Heph	4693	NM 028793	Acbd5	3619	NM 028947	Arl13a	1742
NM 010418	Herc2	15363	NM 010417	Heph	4693	NM 010561	Ilf3	3603
NM 177270	Cdk12	3990	NM 023740	Zdhhc16	1838	NM 177380	Cyp3a44	1962
NM 028799	Tgm5	2531	NM 010418	Herc2	15363	NM 010572	Irs4	6269
NM 010421	Hexa	1865	NM 177270	Cdk12	3990	NM 001137547	Usp51	2249
NM 028803	Gbe1	2988	NM 177278	L3mbtl4	4570	NM 028966	Samd4	6856
NM 028804	Ccdc3	2769	NM 028805	Katnb1	3425	NM 010574	Irx2	2479
NM 010432	Hipk1	8065	NM 010432	Hipk1	8065	NM 177390	Myo1d	5353
NM 001134299	Gm10220	2984	NM 028806	Phactr3	5100	NM 028979	Cyp2j9	1944
NM 010434	Hipk3	7497	NM 183354	Slc12a1	4660	NM 010582	Itih2	3139
NM 001134300	Nol4l	6416	NM 001134383	Iqsec1	4526	NM 028980	Ppp4r4	3683
NM 010435	Hira	4553	NM 177289	Cbfa2t3	7600	NM 177393	Nalcn	7115
NM 177289	Cbfa2t3	7600	NM 001134384	Iqsec1	6490	NM 028981	Cacna1d	8705
NM_028812	Gtf2e1	3058	NM_010437	Hivep2	9788	NM_028982	8430419L09Rik	4225
NM 001134399	Megf11	6018	NM 028812	Gtf2e1	3058	NM 177397	Atp6v1g3	1887
NM 001134426	Cdv3	4032	NM 177296	Tnpo3	3971	NM 001256380	Prdm2	9410
NM_001134427	Cdv3	3825	NM_028814	2810403A07Rik	2999	NM_028993	Mau2	5369
NM 001134457	Nxpe3	6430	NM 028815	Cep97	7904	NM 001141948	Nmi	1328
NM_028815	Cep97	7904	NM_028820	1700017B05Rik	4934	NM_001141949	Nmi	1368
NM 010443	Hmox2	1274	NM 001134465	Dennd6a	6666	NM 177431	Adamts20	6119
NM 001134458	Il9r	3086	NM 028829	Paqr8	4438	NM 028999	Ppp6r3	4979
NM 028816	Xpo6	4493	NM 175930	Rapgef5	3987	NM 010590	Ajuba	3503
NM 028817	Acsl3	3950	NM 028840	Armc1	2955	NM 029001	Elovl7	5453
NM 177307	Cyp4f39	2618	NM 175937	Cpeb2	6823	NM 177464	R3hcc1l	3979
NM_028824	1700016H13Rik	958	NM_001134733	CK137956	2209	NM_177465	Umodl1	5091
NM 177311	Serac1	5540	NM 176830	Leprel4	1903	NM 177469	Gpr123	4777
NM 028826	14-Sep	1689	NM 176832	Spire1	4761	NM 029012	Sppl3	2817
NM_001134480	Plcxd2	7352	NM_028851	1700080016Rik	1454	NM_177546	Pcyt1b	4864
NM 028829	Paqr8	4438	NM 176833	Ppm1f	4933	NM 029022	Scrn3	3247
NM 175833	Cdv3	3335	NM 001134752	Gm16532	2190	NM 010603	Kcnj12	4599
NM 028839	Tmem110	3754	NM 001134767	Ccdc62	3920	NM 029023	Scpep1	2087
NM 001134697	Ctxn3	1472	NM 176835	Dnajc22	1475	NM 010604	Kcnj16	3623
NM 028847	Mageb5	1251	NM 028854	Tbc1d21	1097	NM 010605	Kcnj5	4496
NM 010472	Agfg1	3137	NM 001134829	Lpgat1	7069	NM 010606	Kcnj6	3086

NM 028850	Chic2	1115	NM 176837	Arhgap18	3616	NM 177571	Gm13103	1833
NM_176832	Spire1	4761	NM_176840	Osbpl11	4521	NM_177573	9930104L06Rik	2942
NM 176833	Ppm1f	4933	NM 028864	Zc3hav1	3396	NM 177577	Dcdc2a	6894
NM_001134791	Osbpl9	3109	NM_001256259	4930524N10Rik	919	NM_029053	4930451C15Rik	2231
NM 001134829	Lpgat1	7069	NM 176846	Exph5	9601	NM 029057	Tbc1d30	5713
NM 176837	Arhgap18	3616	NM 028871	Hnrnpr	8025	NM 010620	Kif15	4840
NM 028860	Mtmr3	5691	NM 001135151	Slc39a14	4927	NM 001142570	Dach2	5991
NM 001135001	Ppp2r5c	2608	NM 001135152	Slc39a14	4893	NM 177586	Eif5a2	4992
NM 178936	Tmem56	6224	NM 001135172	C1qtnf7	1652	NM 001142580	Vipas39	2552
NM 001135019	Zfp266	6294	NM 028873	Dnajc14	4229	NM 177588	Thnsl1	3899
NM 176842	Tprkb	2133	NM 001135192	Asap2	5686	NM 001142581	Vipas39	2495
NM 176845	Ddhd1	5099	NM 028874	Snx19	5541	NM 001163104	Fam46d	3101
NM 028873	Dnajc14	4229	NM 028876	Tmed5	3751	NM 029064	Ttll9	1547
NM 028874	Snx19	5541	NM 177318	Zfp653	2174	NM 177591	Igsf1	4432
NM_176850	Bptf	11506	NM_001135611	3110062M04Rik	3400	NM_029069	Fam71d	1778
NM 177319	Zfyve27	5636	NM 177323	Rint1	3311	NM 029078	Pcf11	5804
NM_001135577	Smim13	4499	NM_028888	4931428F04Rik	3571	NM_029081	C2cd5	4388
NM 001135657	Ptprj	4201	NM 028892	Spag17	7862	NM 177598	Fbxw13	1485
NM 023750	Zfp84	5711	NM 001136054	Ate1	4752	NM 177601	Tmem60	899
NM 028886	Lrguk	3058	NM 028894	Lonrf3	7392	NM 001142731	Kctd1	3456
NM 177324	Sbf2	7163	NM 028897	Mei1	3991	NM 029098	Lmbr1l	2167
NM 028892	Spag17	7862	NM 001136057	Cpne6	2218	NM 029102	Glt8d2	2153
NM 028894	Lonrf3	7392	NM 177328	Grm7	4181	NM 029107	Fam228a	2833
NM 028897	Mei1	3991	NM 177333	Exoc3	4597	NM 029116	Kbtbd11	6996
NM 177328	Grm7	4181	NM 010492	Ica1	2090	NM 177612	Ctnna3	3609
NM 177333	Exoc3	4597	NM 028902	Hsf2bp	2867	NM 029122	Iqca	3210
NM 028901	Myo18b	8280	NM 177340	Synpo	5012	NM 029132	Cep57l1	2306
NM 028904	Rmi1	3522	NM 028906	Dpp8	4799	NM 029134	Lrriq1	2576
NM 177340	Synpo	5012	NM 177341	Trpm3	5157	NM 177642	Igfn1	8996
NM_028905	4932438H23Rik	2684	NM_177351	Hykk	5103	NM_029166	Uhrf1bp1l	6446
NM 177341	Trpm3	5157	NM 010509	Ifnar2	3051	NM 010683	Lamc1	7622
NM 001136066	Hmox2	1258	NM 028915	Lrrcc1	3369	NM 177662	Ctso	3453
NM 010501	Ifit3	1998	NM 028916	Efhc2	2570	NM 001142916	Plod2	3719
NM 177346	Gpr149	3336	NM 177353	Slc9a7	2469	NM 029199	Samt4	963
NM_028910	Olfr701	3063	NM_028923	Gle1	3138	NM_177676	4931409K22Rik	2670
NM 028920	Hyal6	3452	NM 028925	Polr3c	1796	NM 177678	Ablim2	3570
NM 028925	Polr3c	1796	NM 028930	Tmc5	2925	NM 001267593	Kcnj12	4427
NM 028932	Eaf1	4238	NM 028938	Lrriq3	2650	NM 177682	Ccz1	1786
NM 177358	Zfp945	6618	NM 023755	Tfcp2l1	9285	NM 001142937	Moap1	3684
NM 001136222	Acsl3	3392	NM 010549	Il11ra1	1766	NM 029219	Rnf19b	2532
NM 177362	Zfp771	1230	NM 001136226	Utp14b	3702	NM 001142939	Gm20604	3835
NM 023755	Tfcp2l1	9285	NM 010550	Il11ra2	2680	NM 001142943	Zfp949	3497
NM 010549	Il11ra1	1766	NM 001136227	Rtkn	2202	NM 177696	Gdpd4	3050
NM 010550	Il11ra2	2680	NM 177366	Gpr157	4762	NM 177697	Vwa3a	4575
NM 177366	Gpr157	4762	NM 001136236	Fcrl1	1853	NM 177701	Gm4894	2294
NM_028943	Sgms2	5791	NM_010560	Il6st	5452	NM_177702	4833427G06Rik	805
NM 001136476	Slx	924	NM 010561	Ilf3	3603	NM 029248	Taf1d	1148
NM 177412	Tmcc1	5719	NM 177373	Ppfia2	6451	NM 029249	Parpbp	3628
NM 010569	Invs	5674	NM 028953	Tmc1	4073	NM 010709	Eif2d	2122
NM 177380	Cyp3a44	1962	NM 010564	Inha	1463	NM 001142957	Zfp955b	3236
NM_010571	Irs3	2337	NM_010565	Inhbc	1988	NM_177705	4932411N23Rik	3634
NM_010572	Irs4	6269	NM_028961	4933433C11Rik	1430	NM_001142963	Gm10778	4747
NM 028966	Samd4	6856	NM 177412	Tmcc1	5719	NM 001267846	Fign	9817

NM_028971	Lrrc71	3988	NM_010569	Invs	5674	NM_001267847	Fign	9798
NM_177388	Slc41a2	4386	NM_177380	Cyp3a44	1962	NM_177711	Spata31d1d	3827
NM_028975	Tmem33	6247	NM_001136556	Fer1l4	6105	NM_001143683	Mpped2	2662
NM_177389	Mia3	6933	NM_010572	Irs4	6269	NM_001143689	H2-Q4	1793
NM_177393	Nalcn	7115	NM_177383	Gpr21	2297	NM_177727	Lsm14b	2560
NM_028982	8430419L09Rik	4225	NM_028973	Lrrc15	5323	NM_010731	Zbtb7a	5373
NM_177396	Ifnl3	582	NM_177387	Ust	4284	NM_010732	Lrrn2	3346
NM_028990	Tmem168	4548	NM_001141922	Bean1	3406	NM_010733	Lrrn3	3544
NM_028992	Gsdmc4	2084	NM_010582	Itih2	3139	NM_029288	Gm10413	993
NM_001256382	Rims2	7404	NM_001141924	Bean1	3189	NM_010734	Lst1	418
NM_001256383	Rims2	7338	NM_028980	Ppp4r4	3683	NM_177739	Zfp507	6702
NM_177448	Mogat2	1787	NM_177393	Nalcn	7115	NM_001143956	Mettl11b	1053
NM_010590	Ajuba	3503	NM_001141925	Bean1	3191	NM_001270456	Gm3259	2299
NM_029000	Gvin1	9040	NM_177395	Map3k9	3396	NM_010740	Cd93	6638
NM_029001	Elovl7	5453	NM_028981	Cacna1d	8705	NM_001270475	Mical3	9448
NM_177462	Zmym6	4229	NM_028986	Gzf1	4152	NM_177747	Zfp711	4330
NM_177464	R3hcc1l	3979	NM_001141931	Rbms1	2495	NM_177748	Kir3dl2	1734
NM_177466	Rab11fip5	4137	NM_001141932	Rbms1	2543	NM_177750	Frmpd3	1401
NM_177469	Gpr123	4777	NM_001256382	Rims2	7404	NM_001270495	Tmem254b	1447
NM_029012	Sppl3	2817	NM_177431	Adamts20	6119	NM_001270496	Tmem254b	1205
NM_001142322	Myo9b	7296	NM_001256383	Rims2	7338	NM_001270498	Tmem254c	1449
NM_177474	D19Bwg1357e	3462	NM_177450	Cndp1	2829	NM_001270499	Tmem254c	1269
NM_001142323	Myo9b	7142	NM_029001	Elovl7	5453	NM_177752	Eme1	2228
NM_029023	Scpep1	2087	NM_177465	Umodl1	5091	NM_010748	Lyst	11806
NM_010604	Kcnj16	3623	NM_010594	Kap	608	NM_029310	Fabp12	2549
NM_023764	Tollip	3705	NM_029008	4833403I15Rik	3219	NM_010749	M6pr	2258
NM_010608	Kcnk3	3810	NM_001142322	Myo9b	7296	NM_177762	Aak1	19120
NM_001142410	Gm14124	3018	NM_001142323	Myo9b	7142	NM_177764	Vmn2r57	2592
NM_177577	Dcdc2a	6894	NM_010605	Kcnj5	4496	NM_001270553	Gm21119	2514
NM_010615	Kif11	4850	NM_177571	Gm13103	1833	NM_029330	Fpgt	3526
NM_001142570	Dach2	5991	NM_177577	Dcdc2a	6894	NM_001145164	Tgtp2	2816
NM_001142580	Vipas39	2552	NM_010617	Kif13a	6866	NM_010758	Mag	2427
NM_001256432	Epha10	5186	NM_029057	Tbc1d30	5713	NM_177774	Srsf12	2939
NM_001142581	Vipas39	2495	NM_177585	Iqcj	1579	NM_029334	Zc3h14	3556
NM_010629	Kifap3	3907	NM_010620	Kif15	4840	NM_029335	1700026D08Rik	1310
NM_001163104	Fam46d	3101	NM_001142570	Dach2	5991	NM_010764	Man2b1	4326
NM_177592	Tmem164	5316	NM_010623	Kif17	3915	NM_001270644	Gm20765	1246
NM_001142647	Tmem194b	3423	NM_001256432	Epha10	5186	NM_029337	Ep400	10798
NM_001142655	Arpp19	3963	NM_177589	Ulk4	4093	NM_001145209	Hbs1l	2673
NM_029069	Fam71d	1778	NM_010630	Kifc2	3143	NM_177783	Ubr3	8109
NM_029074	Cnep1r1	1832	NM_177592	Tmem164	5316	NM_177784	Klhl23	4621
NM_029078	Pcf11	5804	NM_001142681	Chid1	4239	NM_029347	Fggy	1910
NM_001142691	Dmrtc1c1	1611	NM_029078	Pcf11	5804	NM_001270792	Gm2042	1841
NM_029081	C2cd5	4388	NM_029081	C2cd5	4388	NM_029348	Zbtb4	7824
NM_001142697	Tpgs2	3786	NM_029084	Slamf8	2040	NM_177787	Slc15a5	2461
NM_001142701	Hmha1	3928	NM_001142728	Lrrc9	4739	NM_001270806	Gm5795	971
NM_001142728	Lrrc9	4739	NM_029091	Klc4	2322	NM_010778	Cd46	1213
NM_001142729	Lrrc9	2520	NM_001142729	Lrrc9	2520	NM_029355	Prl7b1	925
NM_177602	Wdr25	2657	NM_001142734	Gm8994	1573	NM_177805	Fam179b	6434
NM_001256480	Gm4724	4538	NM_010644	Klk1b26	873	NM_177806	Prpf39	4096
NM_029100	Sepn1	3461	NM_001256480	Gm4724	4538	NM_177808	Gm5082	1595
NM_177607	4933430I17Rik	1783	NM_177606	Plekhh2	6886	NM_177811	Zfp459	3331
NM_177611	Psd4	4700	NM_029102	Glt8d2	2153	NM_029364	Gns	3855
NM_177612	Ctnna3	3609	NM_010650	Klra8	1105	NM_001145435	4930451C15Rik	2135

NM 010657	Hivep3	9021	NM 029115	Ccdc181	1938	NM 029366	Kxd1	1236
NM 177633	Ubxn7	10318	NM 010656	Sspn	4408	NM 177814	Erc2	6083
NM 001256885	Gm3488	1743	NM 010657	Hivep3	9021	NM 001270988	Asxl2	9046
NM 029148	Tmx4	5162	NM 029122	Iqca	3210	NM 001270996	Adam9	4057
NM 029153	Scamp1	3605	NM 010659	Krt31	1581	NM 023790	Wdr54	1267
NM_177642	Igfn1	8996	NM_177620	Rin3	3880	NM_177824	9830107B12Rik	3639
NM 177646	Dgkd	5694	NM 010662	Krt13	1314	NM 001271008	Fam46d	2997
NM_177647	Cdnf	3013	NM_029132	Cep57l1	2306	NM_001145676	2210408I21Rik	4950
NM 029169	Rbm6	3991	NM 177629	Fam216b	2238	NM 177832	Zfp236	9473
NM 010683	Lamc1	7622	NM 029134	Lrriq1	2576	NM 010795	Mgat3	4682
NM 029195	Ccdc146	3389	NM 177633	Ubxn7	10318	NM 029385	Nudt16	1606
NM_177664	D3Bwg0562e	5690	NM_029141	Tmbim7	1680	NM_001271020	Olfr631	1550
NM_177676	4931409K22Rik	2670	NM_177637	H2-M10.5	1403	NM_010799	Minpp1	2619
NM 001267591	Cant1	2758	NM 029148	Tmx4	5162	NM 177839	Tnn	5825
NM 001267592	Cant1	3068	NM 010668	Krt2	2629	NM 029397	Rbm12	3752
NM 029212	Ccdc33	2555	NM 177639	Dlgap1	6323	NM 177854	Sertm1	3086
NM 029219	Rnf19b	2532	NM 029154	Tmbim7	1446	NM 177855	Med12l	10275
NM_029228	4930564D02Rik	737	NM_177642	Igfn1	8996	NM_001145801	Ctla2b	1157
NM 177687	Crebl2	2536	NM 029157	Sf3a3	1710	NM 178016	Pigk	4885
NM 001142943	Zfp949	3497	NM 177646	Dgkd	5694	NM 010817	Psmd7	1616
NM 001142948	Pogk	7051	NM 177660	Zbtb10	7464	NM 010819	Clec4d	1336
NM 001267622	Ttc28	10702	NM 010683	Lamc1	7622	NM 010820	Mpdz	7515
NM 001142952	Fam46c	2054	NM 001142809	Slc6a8	3977	NM 177869	Fam185a	3082
NM 029236	Bcdin3d	1271	NM 029195	Ccdc146	3389	NM 029413	Morc4	4409
NM 029239	Prkd3	5884	NM 001142810	Slc6a8	3968	NM 177870	Slc5a6	3344
NM 029245	Ankrd53	1737	NM 001142916	Plod2	3719	NM 001145824	Hipk3	7494
NM 001267710	Eef2k	6282	NM 177671	Epha10	1236	NM 177873	Hfm1	5018
NM 001267711	Eef2k	6277	NM 177678	Ablim2	3570	NM 001271412	Nfam1	3892
NM 177700	Atmin	4900	NM 029212	Ccdc33	2555	NM 177884	Gcfc2	3375
NM 029250	Etnk1	6447	NM 001267620	Ankzf1	1218	NM 029436	Klhl24	6683
NM_023774	4930550L24Rik	1602	NM_001142948	Pogk	7051	NM_177894	Fam154b	2788
NM 001267846	Fign	9817	NM 001267622	Ttc28	10702	NM 001145874	Muc20	3673
NM 001267847	Fign	9798	NM 010698	Ldb2	2524	NM 178017	Hmgxb4	3924
NM 177712	Zfp874a	3252	NM 001142952	Fam46c	2054	NM 001271434	Haghl	1338
NM 029270	Arhgap24	3353	NM 001267626	Dpf3	1532	NM 001145878	Gm5082	1432
NM 001143686	Apol11b	2057	NM 177699	Fhod1	3951	NM 029453	Wdr64	3817
NM 029277	Arhgap12	4959	NM 001267710	Eef2k	6282	NM 010852	Myef2	2999
NM 177722	Mcmdc2	2553	NM 001267711	Eef2k	6277	NM 029456	Ppp6r3	4892
NM 001143765	Syce1	1462	NM 177701	Gm4894	2294	NM 177910	Gmppb	1789
NM_010733	Lrrn3	3544	NM_177702	4833427G06Rik	805	NM_029465	Clec4g	1239
NM 177739	Zfp507	6702	NM 029250	Etnk1	6447	NM 177918	AV320801	3698
NM 177741	Ppp1r3b	4229	NM 001142957	Zfp955b	3236	NM 177920	Serpina7	2368
NM 010739	Muc13	2969	NM 010712	Lhx4	1853	NM 010864	Myo5a	11684
NM_029293	Phpt1	583	NM_001267724	Asb13	1935	NM_177921	E230019M04Rik	1509
NM_010740	Cd93	6638	NM_001267846	Fign	9817	NM_029482	4930579G24Rik	2004
NM 001270457	Gm7978	1594	NM 010715	Lig1	3111	NM 010867	Myom1	5613
NM 001144855	Ppfia4	6067	NM 001267847	Fign	9798	NM 029492	Zdhhc20	5173
NM 177747	Zfp711	4330	NM 177712	Zfp874a	3252	NM 001145921	Arhgef40	5499
NM_001144953	Vwa5b2	4548	NM_177717	4732456N10Rik	2534	NM_001145922	Arhgef40	5478
NM 001270495	Tmem254b	1447	NM 010720	Lipg	3787	NM 010871	Naip6	6705
NM 001270496	Tmem254b	1205	NM 001143686	Apol11b	2057	NM 001145930	Yeats2	6143
NM 001270498	Tmem254c	1449	NM 029277	Arhgap12	4959	NM 023799	Mgea5	4797

NM 001270499	Tmem254c	1269	NM 001143765	Syce1	1462	NM 178854	Cnot6l	8574
NM 177752	Eme1	2228	NM 177732	Slc35d1	2893	NM 029509	Gbp8	2459
NM 010748	Lyst	11806	NM 001143802	Fam196a	4637	NM 010879	Nck2	2789
NM_001270511	Gm10352	1580	NM_029290	1700011I03Rik	967	NM_029523	Depdc1a	3327
NM 001270512	Gm3376	1580	NM 177742	Triml1	1850	NM 029529	Slc35d3	2629
NM 001270513	Gm21677	1580	NM 001143848	Pde2a	4220	NM 001145954	Lpp	14876
NM 001270514	Gm21693	1580	NM 001143849	Pde2a	4053	NM 001145957	Vwa5a	4081
NM 001270515	Gm21704	1580	NM 010740	Cd93	6638	NM 029536	Gpr165	3194
NM 177762	Aak1	19120	NM 001270457	Gm7978	1594	NM 178027	Vps26b	6890
NM 001270516	Gm21708	1580	NM 177744	Apol10a	3033	NM 178113	Ncapd3	5519
NM 177763	Lhfpl4	4782	NM 001144855	Ppfia4	6067	NM 178115	Edrf1	5338
NM 001270518	Gm4064	1580	NM 001270475	Mical3	9448	NM 001145970	Map7d1	3186
NM 177765	Ttll13	3010	NM 029295	Cklf	667	NM 001145977	Cadm2	9553
NM 029330	Fpgt	3526	NM 177751	Cnksr2	5434	NM 010901	Nfatc3	6032
NM 010757	Mafk	2825	NM 010748	Lyst	11806	NM 010903	Nfe2l3	2544
NM 001145164	Tgtp2	2816	NM 177756	Colgalt2	4080	NM 001146010	Fchsd2	4360
NM 029332	Akap13	12543	NM 177758	Zscan20	5446	NM 010913	Nfya	3641
NM 010762	Mal	2792	NM 010751	Mxd1	4662	NM 001146024	Zfp444	4331
NM 029334	Zc3h14	3556	NM 010752	Mad1l1	2640	NM 178249	Pramel6	1540
NM 029337	Ep400	10798	NM 177762	Aak1	19120	NM 178250	Pramel7	1860
NM_001145209	Hbs1l	2673	NM_029324	1700018C11Rik	671	NM_029578	Tgds	2265
NM 010767	Masp2	744	NM 177764	Vmn2r57	2592	NM 178119	Agap1	9581
NM 177783	Ubr3	8109	NM 001270553	Gm21119	2514	NM 001146045	Fam169a	4537
NM 029347	Fggy	1910	NM 177771	Klhl18	4573	NM 001146046	Lrrc49	2802
NM 001145390	Gm766	1475	NM 010758	Mag	2427	NM 001146047	Lrrc49	2801
NM 029348	Zbtb4	7824	NM 010761	Ccndbp1	1572	NM 001146081	Fancb	2984
NM_177787	Slc15a5	2461	NM_177774	Srsf12	2939	NM_024271	1700016D06Rik	1266
NM 001270806	Gm5795	971	NM 010762	Mal	2792	NM 178259	Abca13	16011
NM_177789	Vsig4	1432	NM_001145192	A330050F15Rik	2296	NM_010941	Nsdhl	2218
NM 029357	Pcdh1	3902	NM 177779	Ccdc42	1353	NM 001146088	Il1f5	1722
NM 177806	Prpf39	4096	NM 010768	Matk	1980	NM 029609	Lhpp	1607
NM 177811	Zfp459	3331	NM 001270718	Ifitm7	865	NM 029614	Prss23	3226
NM 029364	Gns	3855	NM 029348	Zbtb4	7824	NM 181045	Caln1	2286
NM 177813	Gm5087	1921	NM 001145403	Kcnt1	3771	NM 029619	Msrb2	1204
NM 177814	Erc2	6083	NM 177794	Tmem26	5123	NM 178267	Zfp827	7625
NM 010782	Mcpt9	2000	NM 177802	Slc7a15	1907	NM 010953	Oc90	1799
NM 001270988	Asxl2	9046	NM 177806	Prpf39	4096	NM 181273	Heph	3931
NM 001270996	Adam9	4057	NM 177814	Erc2	6083	NM 178279	Pxk	2840
NM 010790	Melk	2914	NM 177816	Sh2d4b	1296	NM 001146107	Gm10696	2247
NM 001271008	Fam46d	2997	NM 177819	Fam135b	4623	NM 001146117	Smim19	1417
NM_001145676	2210408I21Rik	4950	NM_029374	12-Sep	1290	NM_178855	Tmprss15	4149
NM 029379	Tmem225	993	NM 001145660	Rfx8	2024	NM 029620	Pcolce2	1866
NM 001145780	Use1	1087	NM 010792	Mettl1	887	NM 001146119	Fam49a	4799
NM_177839	Tnn	5825	NM_001145676	2210408I21Rik	4950	NM_029626	Glt8d1	2485
NM 029397	Rbm12	3752	NM 010794	Mgat1	3040	NM 178362	Sorbs1	6320
NM 177855	Med12l	10275	NM 177832	Zfp236	9473	NM 001146176	Max	1978
NM 010813	Mnt	4601	NM 010795	Mgat3	4682	NM 001146183	Cpne6	2192
NM 001145804	Nucks1	6093	NM 177838	Fam163a	3892	NM 178367	Dhx33	5179
NM 178016	Pigk	4885	NM 001271349	Fbxw11	4174	NM 001146196	Scin	3008
NM 029407	Fam227a	5187	NM 029397	Rbm12	3752	NM 178381	Ano9	3012
NM 010820	Mpdz	7515	NM 177845	Pla2g4e	4477	NM 029653	Dapk1	5891
NM 001145824	Hipk3	7494	NM 010808	Mmp24	4304	NM 178384	Zfp74	3728
NM 177872	Adamts3	7582	NM 177854	Sertm1	3086	NM 178388	Tmem202	1285
NM 001271397	Nol8	4655	NM 177855	Med12l	10275	NM 029657	Mgrn1	3290
NM 010826	Mrvi1	5968	NM 001271370	Dyrk1b	2477	NM 011011	Oprk1	4677
NM 010829	Msh3	3945	NM 001145802	Mak	3274	NM 001146308	Dbnl	2369

NM_029425	4833424015Rik	4044	NM_177857	Dennd2c	4997	NM_001146309	Dbnl	2357
NM 010831	Sik1	4512	NM 010813	Mnt	4601	NM 029665	Ipo11	4282
NM 177888	Zfp78	4894	NM 001145803	Mak	3562	NM 001146323	Hps3	3615
NM 029436	Klhl24	6683	NM 001145804	Nucks1	6093	NM 011019	Osmr	4835
NM_177889	Zfp82	2120	NM_010816	Morc1	3050	NM_178399	3110035E14Rik	3187
NM 177893	Fan1	3475	NM 010819	Clec4d	1336	NM 001146324	Hps3	2335
NM 001145863	Gm14139	1728	NM 010820	Mpdz	7515	NM 181849	Fgb	1575
NM 177894	Fam154b	2788	NM 177872	Adamts3	7582	NM 029682	Stambpl1	1978
NM 029440	Wdr95	2482	NM 024267	Ipo4	3611	NM 178407	Arap2	7363
NM 001145874	Muc20	3673	NM 029416	Klf17	3595	NM 011028	P2rx6	2362
NM 029441	Cdyl2	8087	NM 010826	Mrvi1	5968	NM 178593	Rcsd1	2670
NM_177901	4933402J07Rik	1023	NM_177879	Sdk1	8513	NM_001271798	Pcdh9	6129
NM 001145880	Zfp790	4435	NM 001271407	Sorbs3	3061	NM 029690	Actrt3	1892
NM_001145881	Zfp212	2719	NM_029423	Rab11fip1	6402	NM_001146690	Chpt1	1466
NM_029463	4930568D16Rik	1227	NM_001271408	Sorbs3	2945	NM_001146707	Nap1l1	3951
NM 177912	Gsdmc2	2077	NM 001271409	Sorbs3	1926	NM 001271799	Pcdh9	5476
NM_177913	A430089I19Rik	1859	NM_029434	Lca5	2010	NM_029698	Ttc18	3621
NM 177920	Serpina7	2368	NM 177888	Zfp78	4894	NM 178415	Bbs9	3438
NM 010864	Myo5a	11684	NM 177894	Fam154b	2788	NM 001271800	Pcdh9	5454
NM 029478	Vmp1	2773	NM 029441	Cdyl2	8087	NM 183046	Kif20b	5563
NM 177922	Mapk15	1961	NM 001145876	Slc25a44	3538	NM 029701	Spcs3	3482
NM 001145900	Btbd6	1993	NM 001145877	Slc25a44	3456	NM 001271809	Pcdh11x	8637
NM 029489	Samd7	3201	NM 029453	Wdr64	3817	NM 011044	Pck1	2617
NM 029492	Zdhhc20	5173	NM 001145880	Zfp790	4435	NM 001159284	Smtn	3397
NM 178065	Arel1	5476	NM 177911	Tgm4	2767	NM 029709	Clasp1	7675
NM_029494	Rab30	2007	NM_177913	A430089I19Rik	1859	NM_178599	Commd8	3464
NM 029497	Urb1	7381	NM 010860	Myl6	656	NM 029720	Creld2	1364
NM 178142	Lcorl	5657	NM 001145896	Gsel	6584	NM 011052	Pdcd6ip	5961
NM 010871	Naip6	6705	NM 010864	Myo5a	11684	NM 178602	Polr2m	2265
NM_029498	Zmym2	6876	NM_177921	E230019M04Rik	1509	NM_011053	Pdcd11	6062
NM 001145924	Msantd3	1745	NM 001145900	Btbd6	1993	NM 011054	Pde1c	4676
NM 001145925	Msantd3	1606	NM 029485	Spata24	721	NM 178606	Reep3	5473
NM 010872	Naip2	4853	NM 029489	Samd7	3201	NM 029727	Gsdma2	1666
NM 010874	Nat2	1473	NM 177940	Ece2	3349	NM 178610	Krr1	4990
NM 029502	Cant1	3109	NM 177941	Ece2	3436	NM 001159329	Gtpbp8	1117
NM 178078	Cnot1	8375	NM 177942	Ece2	3042	NM 001159331	N6amt1	1649
NM 001145930	Yeats2	6143	NM 029492	Zdhhc20	5173	NM 001159351	Ube2v2	5766
NM 178854	Cnot6l	8574	NM 178065	Arel1	5476	NM 029736	Slc10a7	3644
NM 178080	Pde4dip	6200	NM 029495	Epsti1	1869	NM 001159354	Magi3	6581
NM 029519	Rap2a	4174	NM 178142	Lcorl	5657	NM 001159361	Dip2b	8915
NM_001145950	5730409E04Rik	2855	NM_029498	Zmym2	6876	NM_001159364	Cep97	1118
NM 029529	Slc35d3	2629	NM 001145924	Msantd3	1745	NM 001159365	Cep97	1000
NM 178041	Eif5	3827	NM 001145925	Msantd3	1606	NM 001159366	Cep97	829
NM 029531	Zfp60	3863	NM 178076	Mcf2l	5269	NM 011069	Pex11b	2439
NM 178005	Lrrtm2	5673	NM 001145930	Yeats2	6143	NM 178620	Mfsd11	3172
NM 029532	Snrnp35	1133	NM 001271483	Clip4	2521	NM 029749	Usp42	5151
NM 001145957	Vwa5a	4081	NM 001145931	Yeats2	2347	NM 029752	Bri3bp	7001
NM 024269	Arl2bp	1933	NM 001271484	Clip4	2228	NM 029758	Fam49a	4872
NM_178111	Trp53inp2	3934	NM_178080	Pde4dip	6200	NM_001159384	Mlx	1740
NM 178027	Vps26b	6890	NM 001145948	Ttc39a	2334	NM 001159385	Mlx	1830
NM 178113	Ncapd3	5519	NM 001145954	Lpp	14876	NM 011077	Phex	6265
NM 178115	Edrf1	5338	NM 029532	Snrnp35	1133	NM 011081	Piga	3593

NM_029553	Ttc8	2295	NM_001145957	Vwa5a	4081	NM_178631	Ralyl	2519
NM_001145977	Cadm2	9553	NM_029536	Gpr165	3194	NM_029766	Dtl	4202
NM_010901	Nfatc3	6032	NM_178027	Vps26b	6890	NM_011083	Pik3c2a	8042
NM_010903	Nfe2l3	2544	NM_001145960	Slc37a2	4440	NM_011084	Pik3c2g	3549
NM_029565	Tmem59	1562	NM_178113	Ncapd3	5519	NM_001159407	B3gnt5	4872
NM_001146010	Fchsd2	4360	NM_001145965	Dlgap2	4426	NM_001159408	B3gnt5	4669
NM_029570	Atp11b	4883	NM_010895	Neurod2	3137	NM_001159415	Ces3b	1915
NM_001146025	Rnf44	4008	NM_010901	Nfatc3	6032	NM_001272045	Ces2h	1677
NM_001146026	Rnf44	3933	NM_001145978	Parp4	6391	NM_029777	Rhbdd1	3533
NM_001146027	Rnf44	3799	NM_001145979	Gtpbp2	2947	NM_029779	Ccdc116	3912
NM_178119	Agap1	9581	NM_001145999	Zfp710	4486	NM_029782	Calr3	1371
NM_023815	Trp53rk	4413	NM_029562	Cyp2d26	1645	NM_029784	Fam81a	2883
NM_029584	Zfp773	3025	NM_001146000	Zfp710	4584	NM_011100	Prkacb	4341
NM_029585	Det1	2217	NM_001271544	Slc4a9	3161	NM_178647	Cggbp1	4382
NM_178309	Brip1	6933	NM_001271546	Slc4a9	3059	NM_029790	Mettl15	1891
NM_001146073	Hexdc	1969	NM_001271547	Slc4a9	2965	NM_001159496	Ppm1b	2753
NM_001146081	Fancb	2984	NM_178118	Dixdc1	5718	NM_001159497	Ppm1b	1609
NM_001146084	Fastkd5	3127	NM_029570	Atp11b	4883	NM_001159498	Ppm1b	1580
NM_001146085	Fbxo34	3132	NM_178249	Pramel6	1540	NM_178651	Slc30a9	3411
NM_178259	Abca13	16011	NM_178250	Pramel7	1860	NM_001159503	Tnfsf12Tnfsf13	2229
NM_001146086	Fbxo34	3055	NM_029578	Tgds	2265	NM_001159505	Tnfsf13	1676
NM_010942	Nsg1	2140	NM_178119	Agap1	9581	NM_178653	Sccpdh	1922
NM_010945	Nsmaf	3518	NM_029582	Txndc11	2977	NM_011112	Papola	4518
NM_010947	Ntn3	4912	NM_029584	Zfp773	3025	NM_178656	Pirt	3014
NM_178267	Zfp827	7625	NM_001146045	Fam169a	4537	NM_029802	Arfip2	3277
NM_181273	Heph	3931	NM_001146046	Lrrc49	2802	NM_011118	Prl2c3	868
NM_178281	Trim39	3070	NM_001146047	Lrrc49	2801	NM_178660	Rbms3	7881
NM_001146119	Fam49a	4799	NM_029587	1700012A03Rik	918	NM_011120	Prl7d1	997
NM_178362	Sorbs1	6320	NM_001146057	Acot7	1505	NM_001159527	Wdr35	4376
NM_001146176	Max	1978	NM_001146058	Acot7	1367	NM_011128	Pnliprp2	1593
NM_001146180	Mtss1	4933	NM_001146059	Als2cl	5106	NM_178669	Clrn3	3315
NM_178367	Dhx33	5179	NM_178309	Brip1	6933	NM_178670	8030462N17Rik	3418
NM_001146196	Scin	3008	NM_029599	Sun5	1155	NM_178672	Scfd2	3817
NM_178347	Cdc23	4134	NM_001146060	Als2cl	5186	NM_011136	Pou2af1	2566
NM_001146199	Ptpn21	5562	NM_010935	Npy6r	2567	NM_178674	Fbxl21	1965
NM_029640	Trappc9	3324	NM_001146073	Hexdc	1969	NM_023852	Rab3c	8500
NM_178939	Pdrg1	1229	NM_001146081	Fancb	2984	NM_178675	Slc35f1	4973
NM_029653	Dapk1	5891	NM_001146084	Fastkd5	3127	NM_029835	Ticrr	7213
NM_029654	Atg2b	10173	NM_178259	Abca13	16011	NM_178676	Entpd3	3467
NM_029655	Snx7	2171	NM_029606	Cep112	3516	NM_001159548	Nr6a1	5877
NM_178388	Tmem202	1285	NM_029608	Fam209	649	NM_029837	Mpped2	2375
NM_178391	Setmar	1617	NM_029609	Lhpp	1607	NM_001159549	Nr6a1	5874
NM_001146298	Wac	4908	NM_029610	Lyrm1	1282	NM_001276332	Zbtb34	6482
NM_001146299	Sh3rf2	4784	NM_181324	Ddx6	6033	NM_178682	4933426M11Rik	5025
NM_011016	Orm2	774	NM_181045	Caln1	2286	NM_178684	Mapk1ip1l	4493
NM_001146311	Cln3	2487	NM_029617	Casc5	6525	NM_178685	Pcdh20	5237
NM_178398	Wipi2	3913	NM_181047	Ncmap	1632	NM_011151	Ppm1b	3280
NM_178399	3110035E14Rik	3187	NM_181273	Heph	3931	NM_001159561	P2rx6	2281
NM_001271764	Tpm3	2345	NM_023821	Cmya5	11850	NM_011153	Ppp1r17	1738
NM_029671	1700034015Rik	741	NM_178280	Sall3	6903	NM_001159562	Il1rn	2409
NM_178406	Gpr153	3777	NM_001146119	Fam49a	4799	NM_001276360	Camsap1	8029
NM_029679	Fam65b	5598	NM_029623	3110002H16Rik	2134	NM_001276361	Camsap1	8056
NM_029686	Pkd1l2	7386	NM_180662	Trappc9	4069	NM_029847	Arsk	3657
NM_178417	Zfp867	3412	NM_178363	Ylpm1	7050	NM_029850	Bcl7a	3809
NM_001271809	Pcdh11x	8637	NM_001146183	Cpne6	2192	NM_029851	Dync2h1	13972

NM 001150749	Rdh7	1614	NM 178367	Dhx33	5179	NM 178697	Clca5	3921
NM 011044	Pck1	2617	NM 001146196	Scin	3008	NM 178700	Grsf1	2753
NM_029709	Clasp1	7675	NM_178347	Cdc23	4134	NM_029863	9230110F15Rik	1135
NM 178599	Commd8	3464	NM 029640	Trappc9	3324	NM 001159573	Fip1l1	3082
NM_029714	Catsperg2	3957	NM_178939	Pdrg1	1229	NM_178705	Luzp2	5274
NM 011052	Pdcd6ip	5961	NM 180588	Reep4	1686	NM 001159574	Fip1l1	2947
NM 178602	Polr2m	2265	NM 029653	Dapk1	5891	NM 178707	Zfp592	7292
NM 011053	Pdcd11	6062	NM 029654	Atg2b	10173	NM 001159577	Lnx1	2571
NM 029721	Snx27	6273	NM 001146298	Wac	4908	NM 029869	Zkscan1	8036
NM 178604	Txnl4a	1795	NM 001146299	Sh3rf2	4784	NM 011168	Prl7a2	1277
NM 011054	Pde1c	4676	NM 011011	Oprk1	4677	NM 178709	Rnf214	3782
NM 011055	Pde3b	5314	NM 029661	Spatc1l	1331	NM 001159579	Lnx1	2347
NM 001271860	Add2	3497	NM 178394	Jakmip1	2382	NM 178711	Plscr4	3259
NM 178607	Rnf24	5594	NM 178396	Car12	3716	NM 001159580	Lnx1	2317
NM 178610	Krr1	4990	NM 029665	Ipo11	4282	NM 001276419	Fgf12	4743
NM 001271861	Add2	3298	NM 001146323	Hps3	3615	NM 178714	Lrfn5	3726
NM_178612	Cnpy4	1781	NM_178399	3110035E14Rik	3187	NM_011177	Klk6	1358
NM 178613	Gskip	2787	NM 178404	Zc3h6	4916	NM 178716	Tnpo1	5391
NM 001271873	Ano10	2479	NM 178592	Abhd16a	1945	NM 001276422	Cass4	3633
NM 001159351	Ube2v2	5766	NM 178407	Arap2	7363	NM 178720	Zpld1	2589
NM 178615	Rgmb	2633	NM 029686	Pkd1l2	7386	NM 001159599	Nol9	3750
NM 001159354	Magi3	6581	NM 178593	Rcsd1	2670	NM 029879	Rgs7bp	5828
NM 178616	Psmd11	1743	NM 178412	Chil6	1690	NM 178721	Cadm2	9580
NM 029746	Cog2	2839	NM 001271798	Pcdh9	6129	NM 011182	Cyth3	3749
NM 011069	Pex11b	2439	NM 178415	Bbs9	3438	NM 178722	Zfp438	3209
NM 029749	Usp42	5151	NM 178418	Ccdc144b	2506	NM 029884	Hgsnat	2679
NM 001159376	Dusp15	3121	NM 001271800	Pcdh9	5454	NM 001276443	Gpr155	5022
NM 178624	Fbxl2	3203	NM 029701	Spcs3	3482	NM 178723	Zfp385b	3217
NM_011074	Cdk14	4911	NM_011044	Pck1	2617	NM_029886	9430038I01Rik	1125
NM 029758	Fam49a	4872	NM 001159284	Smtn	3397	NM 001159609	Lrrc57	1915
NM 178627	Poldip3	3249	NM 029709	Clasp1	7675	NM 001159610	Lrrc57	1966
NM 001159392	Tnfaip1	3696	NM 178599	Commd8	3464	NM 029891	Nkrf	3279
NM_001159401	Upp1	1270	NM_029714	Catsperg2	3957	NM_178727	D630039A03Rik	2929
NM 029766	Dtl	4202	NM 011049	Cdk16	3042	NM 011197	Ptgfrn	5786
NM 011083	Pik3c2a	8042	NM 029716	Chn1	3251	NM 029897	C2cd5	4337
NM 001159402	Upp1	1269	NM 011053	Pdcd11	6062	NM 001159627	Heph	4685
NM 011084	Pik3c2g	3549	NM 029721	Snx27	6273	NM 178731	Lrrtm4	3383
NM 001159407	B3gnt5	4872	NM 178605	Nop16	1734	NM 001159628	Heph	4663
NM 001159408	B3gnt5	4669	NM 178606	Reep3	5473	NM 178736	Elmod2	4709
NM 029777	Rhbdd1	3533	NM 011055	Pde3b	5314	NM 178739	Dcaf12l1	3512
NM 178638	Tmem108	3692	NM 178607	Rnf24	5594	NM 011204	Ptpn13	8298
NM 029779	Ccdc116	3912	NM 001159349	Fyttd1	4295	NM 178745	Tmem229b	3904
NM 178640	B3galnt2	3681	NM 011060	Padi3	3068	NM 178746	Slc38a9	8913
NM 029790	Mettl15	1891	NM 001159351	Ube2v2	5766	NM 178749	Stk32a	3765
NM 029791	Bicd2	4659	NM 001271884	Ano7	3899	NM 001159645	Araf	1571
NM 178651	Slc30a9	3411	NM 011063	Pea15a	2477	NM 178754	Arhgap6	4058
NM_001159503	Tnfsf12Tnfsf13	2229	NM_029741	Ppfia3	4882	NM_011211	Ptprd	9127
NM 011109	Pla2g2d	2264	NM 001159361	Dip2b	8915	NM 029928	Ptprb	11912
NM 001159505	Tnfsf13	1676	NM 011068	Pex11a	879	NM 001159646	Dut	2263
NM 178656	Pirt	3014	NM 029746	Cog2	2839	NM 011212	Ptpre	5411
NM 029802	Arfip2	3277	NM 011069	Pex11b	2439	NM 029929	Vps33a	4114
NM 178660	Rbms3	7881	NM 001159374	Krt32	1673	NM 011214	Ptpru	5498
NM_029810	Nt5c2	3769	NM_029752	Bri3bp	7001	NM_178756	E130309F12Rik	3762
NM 001159525	Pex19	2855	NM 178624	Fbxl2	3203	NM 029932	Spns3	1819
NM 011128	Pnliprp2	1593	NM 011074	Cdk14	4911	NM 011216	Ptpro	6634

NM 178669	Clrn3	3315	NM 178625	Tmem209	3422	NM 001159662	Ppp1r16b	6392
NM_178670	8030462N17Rik	3418	NM_029758	Fam49a	4872	NM_011217	Ptprr	3466
NM 011136	Pou2af1	2566	NM 023844	Jam2	4822	NM 029938	H2afv	1636
NM 178674	Fbxl21	1965	NM 029766	Dtl	4202	NM 001159683	Zfp217	5670
NM 023852	Rab3c	8500	NM 011083	Pik3c2a	8042	NM 029942	Prelid2	724
NM 001159548	Nr6a1	5877	NM 178632	Ints7	3252	NM 029943	Apex2	1903
NM 178677	Sec22c	5801	NM 001159407	B3gnt5	4872	NM 001159697	Efcc1	2754
NM 001159549	Nr6a1	5874	NM 001272031	Arvcf	4330	NM 178767	Agmo	2155
NM 001276332	Zbtb34	6482	NM 001159408	B3gnt5	4669	NM 001159706	Folh1	2948
NM 001159553	H13	4499	NM 029773	Spopl	2761	NM 178869	Ttll1	2074
NM 029839	Trub1	4300	NM 001159419	Sidt1	4281	NM 011233	Rad17	2863
NM_178682	4933426M11Rik	5025	NM_029781	Rab36	2620	NM_178772	Nceh1	4394
NM_178684	Mapk1ip1l	4493	NM_001159485	Mcf2l	5198	NM_029955	Ccdc93	7269
NM 178685	Pcdh20	5237	NM 001159486	Mcf2l	5112	NM 001159729	Rab23	4209
NM 001276356	Setmar	1770	NM 011100	Prkacb	4341	NM 178779	Rnf152	8529
NM 178686	Cep120	4572	NM 178647	Cggbp1	4382	NM 178782	Bcorl1	6142
NM 001276360	Camsap1	8029	NM 001159496	Ppm1b	2753	NM 001159750	Tceal1	2724
NM 001276361	Camsap1	8056	NM 178648	Ubxn8	2817	NM 001159751	Tceal1	2620
NM 029847	Arsk	3657	NM 029791	Bicd2	4659	NM 023876	Elp4	3902
NM 029850	Bcl7a	3809	NM 001159497	Ppm1b	1609	NM 011260	Reg3g	774
NM_029858	Ston1	2983	NM_001159498	Ppm1b	1580	NM_029976	Cdkn2aipn1	1880
NM 001159573	Fip1l1	3082	NM 011106	Pkig	1066	NM 178794	Zrsr2	3953
NM 001159574	Fip1l1	2947	NM 011109	Pla2g2d	2264	NM 029983	Sla2	2508
NM 001159577	Lnx1	2571	NM 029794	Cpsf4l	1250	NM 178795	Ppip5k1	5559
NM 011166	Prl6a1	893	NM 029799	Arrdc5	1163	NM 178798	Slc7a6	3730
NM 178709	Rnf214	3782	NM 178658	Fam65b	3161	NM 029988	Pigh	2376
NM 001159579	Lnx1	2347	NM 029802	Arfip2	3277	NM 011269	Rhag	2316
NM 001159580	Lnx1	2317	NM 178659	Jmjd4	4445	NM 178803	Aebp2	5932
NM 001276419	Fgf12	4743	NM 011118	Prl2c3	868	NM 001159907	Gm17296	5060
NM_011177	Klk6	1358	NM_001159523	Shd	1551	NM_029998	6030458C11Rik	3974
NM 178720	Zpld1	2589	NM 178662	Atcay	3677	NM 011276	Rlim	7435
NM 178721	Cadm2	9580	NM 029810	Nt5c2	3769	NM 178870	Hs3st3a1	3933
NM 029881	Tmem200a	4115	NM 001159525	Pex19	2855	NM 178872	Trim36	4556
NM 001159603	Pum1	5361	NM 178664	B3gntl1	2866	NM 178873	Adck2	3539
NM 011185	Psmb1	1036	NM 029811	St5	3004	NM 030016	Trmt13	3159
NM 001159604	Pum1	5367	NM 001159527	Wdr35	4376	NM 011282	Ros1	7401
NM 001159605	Pum1	5079	NM 001276288	Aes	1485	NM 001159952	Pde1c	8837
NM_001159606	Pum1	4635	NM_029816	2610028H24Rik	1763	NM_178876	Ints3	4379
NM_029891	Nkrf	3279	NM_178670	8030462N17Rik	3418	NM_011285	Rpgr	2801
NM 178728	Napepld	3673	NM 011133	Pole2	1695	NM 001159955	Pde1c	4261
NM 029897	C2cd5	4337	NM 029825	Scfd1	2091	NM 001159956	Pde1c	4118
NM_178730	Tmprss11f	4277	NM_178673	Fstl5	4820	NM_001159957	Pde1c	8310
NM 029901	Akr1c21	1222	NM 023852	Rab3c	8500	NM 030033	Crisp4	1192
NM 001159628	Heph	4663	NM 029834	Ppp1r12c	2982	NM 001159960	Pde1c	8738
NM 011200	Ptp4a1	4153	NM 029835	Ticrr	7213	NM 011290	Rpl6	1282
NM 178733	Zfp14	3427	NM 178676	Entpd3	3467	NM 030035	Golgb1	11025
NM_029909	C330018D20Rik	2416	NM_029836	Tspyl2	2776	NM_001159965	Ralgps2	6992
NM 178736	Elmod2	4709	NM 001159548	Nr6a1	5877	NM 030046	Dnajc21	2053
NM 001159631	Nek6	3175	NM 001159549	Nr6a1	5874	NM 001159966	Ralgps2	6890
NM 001276465	Vps13d	15846	NM 178678	Lrrtm3	3852	NM 001159967	Ralgps2	6914
NM 001159633	Slc44a1	1938	NM 001276332	Zbtb34	6482	NM 001159968	Ralgps2	6887
NM 011204	Ptpn13	8298	NM 001159551	H13	5402	NM 178890	Abtb2	4595
NM 029920	Mtus2	7543	NM 011147	Ppef1	2483	NM 011300	Rps7	961

NM_178744	Zbtb1	4001	NM_178682	4933426M11Rik	5025	NM_030059	Cst11	606
NM_011207	Ptpn3	6369	NM_029843	1700109H08Rik	745	NM_178899	Hepacam2	2236
NM 178749	Stk32a	3765	NM 011150	Lgals3bp	2168	NM 030066	Armcx1	2446
NM 001159645	Araf	1571	NM 178685	Pcdh20	5237	NM 011307	Uimc1	2931
NM 178754	Arhgap6	4058	NM 011151	Ppm1b	3280	NM 178906	AI593442	5635
NM 011211	Ptprd	9127	NM 011153	Ppp1r17	1738	NM 178907	Mapkapk3	2816
NM 029928	Ptprb	11912	NM 001276360	Camsap1	8029	NM 030068	Iqch	3375
NM 029929	Vps33a	4114	NM 001276361	Camsap1	8056	NM 001001295	Dis3l	3575
NM 011213	Ptprf	7648	NM 178695	Prrg4	2846	NM 023884	Ralgps2	6932
NM 011214	Ptpru	5498	NM 178696	Slc25a44	3584	NM 001160107	Zc3h14	3088
NM 178760	Gpr107	3926	NM 178697	Clca5	3921	NM 178912	Fancm	7775
NM 029934	Mboat7	2880	NM 029857	Tmco4	3322	NM 001160108	Zc3h14	2357
NM 001276676	Syt6	4285	NM 178701	Lrrc8d	3920	NM 030072	Plb1	3147
NM 029937	Nup210l	5681	NM 001159577	Lnx1	2571	NM 001277239	Zmat4	4247
NM 001159683	Zfp217	5670	NM 011166	Prl6a1	893	NM 030081	Zfyve20	5242
NM 029945	Smpd4	4632	NM 178708	Pcid2	2408	NM 178924	Upk1b	1769
NM 029947	Prdm8	3119	NM 001159578	Lnx1	3098	NM 178925	Nsun3	1344
NM 029948	Pramef12	2460	NM 029869	Zkscan1	8036	NM 178934	Slc2a12	3762
NM 001159706	Folh1	2948	NM 178709	Rnf214	3782	NM 030096	Ddx52	3180
NM 178768	Tmem72	3335	NM 001159579	Lnx1	2347	NM 181277	Col14a1	6465
NM_178771	Klhl26	3069	NM_178711	Plscr4	3259	NM_181278	B230219D22Rik	4691
NM 178772	Nceh1	4394	NM 001159580	Lnx1	2317	NM 011341	Sdf4	5174
NM 029955	Ccdc93	7269	NM 001159582	Pde1a	4514	NM 030131	Cnih4	3283
NM 178779	Rnf152	8529	NM 029878	Tbcd	3912	NM 030132	Utp23	2917
NM 178785	Rasal3	3677	NM 011176	St14	4017	NM 030138	Acap2	6493
NM 001159750	Tceal	2724	NM 178714	Lrfn5	3726	NM 011351	Sema6c	3612
NM_001159751	Tceal	2620	NM_001276422	Cass4	3633	NM_030141	1700061G19Rik	2455
NM 029965	Rnf170	4049	NM 029881	Tmem200a	4115	NM 001160219	Pum2	6311
NM 011251	Rbm6	3720	NM 178722	Zfp438	3209	NM 181348	Prune2	12512
NM 011253	Rbmy	1731	NM 001276443	Gpr155	5022	NM 001160220	Pum2	6305
NM_011258	Rfc1	4731	NM_029886	9430038I01Rik	1125	NM_198117	Pcdha2	5379
NM 178791	Vstm4	2743	NM 178726	Ppm1l	3962	NM 030145	Lsm6	3702
NM 178794	Zrsr2	3953	NM 001159619	Pigp	805	NM 001160221	Pum2	6120
NM 029981	Adamtsl2	3242	NM 001159620	Pigp	846	NM 001160222	Pum2	5883
NM 178797	Far2	3397	NM 001159624	Rnpep	2184	NM 201243	Pcdha8	5242
NM 001159904	Klrb1c	2641	NM 011197	Ptgfrn	5786	NM 001003671	Pcdhac1	5296
NM 178802	Trim65	3050	NM 029897	C2cd5	4337	NM 001003672	Pcdhac2	5422
NM 178803	Aebp2	5932	NM 001159627	Heph	4685	NM 001160229	Zfp275	6283
NM 178851	Vps39	4362	NM 029898	Ankrd55	2781	NM 001277316	Fam134b	2811
NM 178870	Hs3st3a1	3933	NM 178731	Lrrtm4	3383	NM 001277317	Fam134b	2789
NM 178872	Trim36	4556	NM 011199	Pth1r	2238	NM 001277318	Fam134b	2761
NM 023879	Rpgrip1	5238	NM 001159628	Heph	4663	NM 030153	Naa35	2646
NM_001159942	Plekhg1	7356	NM_029906	9530003J23Rik	1219	NM_181395	Pxdn	6632
NM_030013	Cyp20a1	1501	NM_178732	Zfp324	4316	NM_030165	Csgalnact2	3676
NM_030016	Trmt13	3159	NM_029909	C330018D20Rik	2416	NM_001277321	Ilf3	3594
NM 001277179	Gm1966	8675	NM 178736	Elmod2	4709	NM 001277322	Ilf3	3531
NM 001277180	Gm1966	8687	NM 001159631	Nek6	3175	NM 181398	Anks1b	4653
NM_001277182	Gm1966	8825	NM_023865	9530002B09Rik	825	NM_030167	Fam122b	4334
NM 001277183	Gm1966	8959	NM 001276465	Vps13d	15846	NM 181399	Usp6nl	4663
NM 011285	Rpgr	2801	NM 001159633	Slc44a1	1938	NM 030172	Efcab11	2069
NM 001159955	Pde1c	4261	NM 011204	Ptpn13	8298	NM 011369	Shcbp1	2244
NM 001159956	Pde1c	4118	NM 178741	Klhl8	3163	NM 030174	Mctp1	4289
NM 030035	Golgb1	11025	NM 178744	Zbtb1	4001	NM 030178	Brpf1	4648

NM 178883	Gorab	2509	NM 011207	Ptpn3	6369	NM 181517	Ipo7	3117
NM 001159964	Eps15	4244	NM 001159635	Rbm18	2473	NM 030179	Clip4	4055
NM 178884	Obsl1	5787	NM 178749	Stk32a	3765	NM 001160307	Serpinb10	3314
NM_030047	Spata31	3233	NM_178751	Orai2	3851	NM_011373	St6galnac4	3994
NM 030059	Cst11	606	NM 001159645	Araf	1571	NM 030181	Vsig1	2707
NM 178898	Zfp956	2776	NM 011211	Ptprd	9127	NM 181413	Anks1	5823
NM 178899	Hepacam2	2236	NM 029928	Ptprb	11912	NM 001160345	Svip	3077
NM 030066	Armcx1	2446	NM 001159646	Dut	2263	NM 181415	Atrnl1	6596
NM 178906	AI593442	5635	NM 011212	Ptpre	5411	NM 181419	Zfp599	3597
NM 178907	Mapkapk3	2816	NM 178755	Agbl2	3462	NM 001160368	Rnf152	8406
NM_030068	Iqch	3375	NM_029929	Vps33a	4114	NM_001160369	A830018L16Rik	2805
NM_001199357	Tmem164	1748	NM_011213	Ptprf	7648	NM_001160370	A830018L16Rik	6828
NM 011309	S100a1	615	NM 029930	Fam115a	3738	NM 181421	Gm15800	15496
NM_178908	Fam26e	1786	NM_011214	Ptpru	5498	NM_001160371	A830018L16Rik	6181
NM 030070	Lrrc9	3817	NM 001276676	Syt6	4285	NM 001160378	Fam46a	5479
NM 030071	Mroh9	2956	NM 011216	Ptpro	6634	NM 001160379	Fam46a	5450
NM 001277238	Hoxa9	3056	NM 001159662	Ppp1r16b	6392	NM 181423	Supv3l1	2436
NM 001160107	Zc3h14	3088	NM 001159671	Rsph6a	1592	NM 023907	Foxi1	2354
NM 001160108	Zc3h14	2357	NM 029937	Nup210l	5681	NM 001160399	Ccdc112	2818
NM 030074	Zfp687	4632	NM 001159672	Eftud1	3608	NM 181470	Ltv1	1784
NM 178922	Hic2	6360	NM 029943	Apex2	1903	NM 181529	Syt15	3625
NM 001160142	Gm20867	1248	NM 178765	Sybu	3152	NM 001160404	Galnt1	3908
NM 030100	Wibg	1173	NM 029945	Smpd4	4632	NM 024282	Desi2	4214
NM 001160143	Gm20823	1249	NM 001159697	Efcc1	2754	NM 030197	Cacul1	5808
NM 181071	Tanc2	11864	NM 178767	Agmo	2155	NM 030199	Zfp623	2486
NM 181072	Myo1e	4944	NM 001159706	Folh1	2948	NM 030208	Trmt44	3784
NM 001160144	Gm20816	1252	NM 178768	Tmem72	3335	NM 001160424	Tgm7	2133
NM 030109	Sf3b2	3249	NM 178869	Ttll1	2074	NM 011403	Slc4a1	4584
NM_181074	Lingo1	3321	NM_011233	Rad17	2863	NM_024283	1500015010Rik	888
NM 001160145	Tmem9	1595	NM 029952	Zfp955a	3755	NM 030219	Trim42	2465
NM 030110	Micu3	3268	NM 029955	Ccdc93	7269	NM 001161355	Timd2	3153
NM 001160146	Tmem9	1491	NM 178779	Rnf152	8529	NM 001161356	Timd2	2926
NM 030113	Arhgap10	3038	NM 029959	Lcn9	839	NM 001161362	Ppp2r3a	6938
NM 181277	Col14a1	6465	NM 029961	Abcb5	3876	NM 181595	Ppp1r9a	9547
NM 030114	Herc4	4043	NM 011246	Rasgrp1	5155	NM 181649	Gpatch11	4237
NM 011342	Sec22b	1815	NM 001277080	Gas7	6848	NM 001161373	F8	7312
NM 181279	Bre	1410	NM 029965	Rnf170	4049	NM 001161374	F8	7306
NM 181280	Bre	1308	NM 001277096	Pkig	1050	NM 001161406	Grhl1	3487
NM 181281	Bre	1143	NM 178787	Iffo1	2840	NM 030229	Polr3h	2514
NM 181282	Bre	1220	NM 011258	Rfc1	4731	NM 030231	Agbl4	1900
NM 011350	Sema4f	4067	NM 178789	Tmem117	2781	NM 030235	Avl9	6691
NM 030138	Acap2	6493	NM 001159864	Kctd18	2615	NM 181729	Muc6	8553
NM 030139	Zfp449	3756	NM 178795	Ppip5k1	5559	NM 181751	Gpr119	2278
NM 001160215	Fcrla	1661	NM 178797	Far2	3397	NM 001161665	Kif26b	7422
NM 001160219	Pum2	6311	NM 029988	Pigh	2376	NM 181854	Champ1	4016
NM 001160220	Pum2	6305	NM 178803	Aebp2	5932	NM 024287	Rab6a	3243
NM 030145	Lsm6	3702	NM 001159907	Gm17296	5060	NM 181816	Ccdc67	3045
NM_001160221	Pum2	6120	NM_029998	6030458C11Rik	3974	NM_024290	Tnfrsf23	3283
NM 001160222	Pum2	5883	NM 023878	Cldn10	1200	NM 024414	Stx1b	1574
NM 023900	Plekhj1	1276	NM 029999	Lbh	3068	NM 024427	Tpm1	1702
NM 011361	Sgk1	2471	NM 180678	Gars	2390	NM 001161618	Cul5	5942
NM 001160256	Smg7	5867	NM 001159934	Oas1h	1318	NM 001161620	Mpp7	1785
NM 001160257	Smg7	5857	NM 178851	Vps39	4362	NM 011455	Serpinb9g	1936
NM 030172	Efcab11	2069	NM 178872	Trim36	4556	NM 011456	Serpinb9e	1921
NM_011371	St6galnac1	2396	NM_023879	Rpgrip1	5238	NM_011460	Serpinb9d	2101

NM 001160303	Gm4788	2884	NM 178874	Tmcc2	3439	NM 030675	Krit1	6105
NM 181517	Ipo7	3117	NM 030014	Hook1	4252	NM 181989	Sdr16c5	1379
NM_011372	St6galnac3	3885	NM_011282	Ros1	7401	NM_011465	Spta1	8405
NM 030179	Clip4	4055	NM 001159952	Pde1c	8837	NM 001161722	Tfeb	2357
NM_001160307	Serpinb10	3314	NM_030018	Tmem50b	2237	NM_001161723	Tfeb	2351
NM 030180	Usp54	6495	NM 030025	Ccdc150	3807	NM 001161738	Glmn	2001
NM 001277851	Treh	1952	NM 001159957	Pde1c	8310	NM 001161739	Glmn	1823
NM 030184	Armc9	5157	NM 001159960	Pde1c	8738	NM 001161741	Reg3d	644
NM 181412	Zbed4	5403	NM 030035	Golgb1	11025	NM 001278943	Abca16	4617
NM 030185	Zcchc4	1924	NM 001159964	Eps15	4244	NM 030246	Dcaf4	2097
NM 001160319	Ubr4	15901	NM 030051	Gsto2	1258	NM 001278944	Abca16	5082
NM 001277863	Dazl	2913	NM 011299	Rps6ka2	5435	NM 182808	Fam19a1	3252
NM 181413	Anks1	5823	NM 011304	Ruvbl2	1675	NM 030249	Cttnbp2nl	4946
NM 181419	Zfp599	3597	NM 178906	AI593442	5635	NM 030250	Nus1	4614
NM 001160368	Rnf152	8406	NM 001160049	Dusp27	4038	NM 011484	Stam	3720
NM_001160369	A830018L16Rik	2805	NM_001160096	Cldn10	1143	NM_182839	Tppp	5108
NM_001160370	A830018L16Rik	6828	NM_001160097	Cldn10	1092	NM_001281525	Gm5862	804
NM 181421	Gm15800	15496	NM 001160098	Cldn10	1035	NM 182994	Arl5a	5212
NM_001160371	A830018L16Rik	6181	NM_030070	Lrrc9	3817	NM_001161790	Mefv	3249
NM 181423	Supv3l1	2436	NM 030071	Mroh9	2956	NM 011494	Stk16	2885
NM 001160399	Ccdc112	2818	NM 178912	Fancm	7775	NM 001161796	Gucy1b3	3234
NM 181541	Caprin2	3566	NM 001160108	Zc3h14	2357	NM 183016	Cdc42bpb	6718
NM 030197	Cacul1	5808	NM 001277239	Zmat4	4247	NM 001161800	Klhl7	3329
NM 030201	Hspa13	4079	NM 001277245	Acr	1567	NM 030563	N4bp1	6469
NM 181585	Pik3r3	5004	NM 001277246	Acr	1500	NM 011505	Stxbp4	6035
NM 023912	Scyl1	2637	NM 030080	Creb3l4	1553	NM 030595	Nbea	10998
NM 011402	Slc34a2	4185	NM 001277247	Acr	1378	NM 183021	Thada	7896
NM 181588	Cmbl	1069	NM 030081	Zfyve20	5242	NM 001161822	Rgs17	8191
NM 030210	Aacs	3175	NM 011319	Sars	3704	NM 030596	Dsg3	4108
NM 207301	Wrb	2483	NM 178930	Gbf1	6422	NM 183022	Asic4	2650
NM 001277957	Taf1a	2391	NM 181039	Lphn1	8202	NM 011511	Abcc9	7362
NM 001277958	Taf1a	2419	NM 181071	Tanc2	11864	NM 030599	Klrb1b	2212
NM 001277959	Taf1a	2398	NM 011334	Clcn4-2	4620	NM 030601	Clca2	3654
NM 030221	Nadsyn1	2632	NM 181073	Plekhh1	6451	NM 183028	Pcmtd1	5235
NM 030224	Fndc8	2081	NM 030109	Sf3b2	3249	NM 001161837	Ptprr	2860
NM 001161362	Ppp2r3a	6938	NM 181074	Lingo1	3321	NM 001161838	Ptprr	2817
NM 181595	Ppp1r9a	9547	NM 030110	Micu3	3268	NM 011520	Sdc3	4972
NM 001161366	Tdrd6	7062	NM 001160149	Cgref1	1266	NM 001161839	Ptprr	2508
NM 001161367	Tdrd6	7041	NM 181277	Col14a1	6465	NM 183033	Zfp516	7725
NM 011413	C4a	5369	NM 030114	Herc4	4043	NM 001161840	Ptprr	2700
NM_001161373	F8	7312	NM_181278	B230219D22Rik	4691	NM_207302	Zranb1	2127
NM 001161374	F8	7306	NM 011341	Sdf4	5174	NM 001281955	Csmd2	13555
NM 001161406	Grhl1	3487	NM 181279	Bre	1410	NM 183064	Fgf12	5449
NM 011419	Kdm5d	5471	NM 181280	Bre	1308	NM 183087	Fam189a1	4790
NM 030235	Avl9	6691	NM 181281	Bre	1143	NM 001161854	Csde1	4108
NM 181729	Muc6	8553	NM 181282	Bre	1220	NM 030685	Serp1	2457
NM_030236	Fbxo34	2945	NM_030141	1700061G19Rik	2455	NM_183094	Xlr4c	1247
NM 001161431	Slitrk2	7778	NM 181348	Prune2	12512	NM 030687	Slco1a4	3847
NM 181750	R3hdm1	4812	NM 023900	Plekhj1	1276	NM 011541	Tceal	2727
NM 181815	Cep128	4826	NM 181395	Pxdn	6632	NM 183103	Prss46	1257
NM 181854	Champ1	4016	NM 011361	Sgk1	2471	NM 183109	Tmprss12	1349
NM 024287	Rab6a	3243	NM 001277321	Ilf3	3594	NM 001162375	Fam73a	5163
NM_024288	Rmnd5a	6165	NM_001277322	Ilf3	3531	NM_183113	4932414N04Rik	3643
NM 011446	Sox7	3266	NM 001160253	Cabp2	1057	NM 183115	Ccdc125	2705

NM 001161618	Cul5	5942	NM 001160256	Smg7	5867	NM 183116	Slc18b1	2837
NM_011453	Serpinb9c	1903	NM_011365	Itsn2	6001	NM_011549	Tfeb	2298
NM_024461	1810037I17Rik	991	NM_030167	Fam122b	4334	NM_001162387	Pex11b	2019
NM_011454	Serpinb6b	1859	NM_011366	Sorbs3	2948	NM_011550	Mlx	1992
NM_011456	Serpinb9e	1921	NM_001160257	Smg7	5857	NM_183126	6030498E09Rik	1184
NM 011459	Serpinb8	3075	NM 030172	Efcab11	2069	NM 030697	Kank3	2634
NM_011460	Serpinb9d	2101	NM_023906	Asb3	3225	NM_030699	Ntng1	5347
NM 024468	Trim39	3085	NM 181402	Parp11	3724	NM 001162417	Myef2	2402
NM 181988	Rerg	2074	NM 001160268	Plekha6	7085	NM 001162418	Myef2	2927
NM 030675	Krit1	6105	NM 030174	Mctp1	4289	NM 030708	Zfhx4	13874
NM_181989	Sdr16c5	1379	NM_011372	St6galnac3	3885	NM_001282051	Gak	4793
NM 001161714	Tgm1	2755	NM 030179	Clip4	4055	NM 001282052	Gak	4655
NM 001161715	Tgm1	2854	NM 181409	Mtmr11	2812	NM 001282054	Msh4	3242
NM 030239	Abcg3	3034	NM 030180	Usp54	6495	NM 001162465	Dtnb	2355
NM 001278941	Dennd2d	4129	NM 030184	Armc9	5157	NM 011579	Tgtp1	2809
NM 001278943	Abca16	4617	NM 181412	Zbed4	5403	NM 030723	Pum2	6235
NM 030246	Dcaf4	2097	NM 001160319	Ubr4	15901	NM 011584	Nr1d2	4640
NM 001278944	Abca16	5082	NM 001277863	Dazl	2913	NM 001162477	Gab2	6005
NM 011481	Srms	2547	NM 181413	Anks1	5823	NM 030730	Rad54l2	9342
NM 030250	Nus1	4614	NM 181415	Atrnl1	6596	NM 183165	Pyroxd1	2155
NM 011484	Stam	3720	NM 011375	St3gal5	2453	NM 030733	Gpr63	2370
NM 030253	Parp9	3564	NM 001160368	Rnf152	8406	NM 207176	Tes	2442
NM 001161763	Fmo5	5202	NM 011379	Sipa1	3837	NM 001282108	Slc10a7	3518
NM_001161765	Fmo5	5285	NM_001160369	A830018L16Rik	2805	NM_001282109	Slc10a7	3568
NM_001281491	Syngap1	4023	NM_001160370	A830018L16Rik	6828	NM_001282128	Brpf1	4796
NM 030257	Lysmd3	3139	NM 181421	Gm15800	15496	NM 183179	Kcnv2	5092
NM_030258	Gpr146	4065	NM_001160371	A830018L16Rik	6181	NM_011599	Tle1	11550
NM_182997	Prkab2	4278	NM_030192	4930562C15Rik	3829	NM_011603	Tbpl1	2880
NM 183014	Zfp184	2730	NM 030194	Sp110	1921	NM 183185	Zfp300	4658
NM 001161796	Gucy1b3	3234	NM 011385	Ski	5481	NM 001162523	Ceacam12	1505
NM 183015	Ccnb3	4554	NM 181444	Gprc5a	2072	NM 001162524	Ceacam12	1899
NM 183016	Cdc42bpb	6718	NM 023907	Foxi1	2354	NM 183188	Rbfox1	4102
NM 001161800	Klhl7	3329	NM 001160399	Ccdc112	2818	NM 011615	Dedd	2700
NM_011503	Stxbp2	2703	NM_181852	Prl2c5	694	NM_001162537	9330159F19Rik	5644
NM 011505	Stxbp4	6035	NM 001160404	Galnt1	3908	NM 030750	Sgpp1	3324
NM 030564	Rnf34	1980	NM 181544	Pkd1l3	6456	NM 030886	Ankrd17	10066
NM 001161818	Zfp639	1937	NM 181548	Eras	1077	NM 001283011	Rad17	2940
NM 183021	Thada	7896	NM 011397	Slc23a1	3009	NM 183204	Rnf182	3456
NM 030596	Dsg3	4108	NM 030205	Coro7	3518	NM 030889	Sorcs2	5722
NM 030599	Klrb1b	2212	NM 181569	Nprl3	2865	NM 183208	Zmiz1	7526
NM 183028	Pcmtd1	5235	NM 001160421	Optc	1747	NM 011629	Nr2c1	3827
NM 001161845	Sgk1	3121	NM 030208	Trmt44	3784	NM 031174	Dscam	7481
NM 001161847	Sgk1	2622	NM 001160422	Optc	1197	NM 183225	Usp24	10601
NM 001161848	Sgk1	2415	NM 001160424	Tgm7	2133	NM 133626	Rrbp1	2706
NM 001281955	Csmd2	13555	NM 030209	Crispld2	4165	NM 183316	Snapc5	781
NM 001161849	Sgk1	2496	NM 030210	Aacs	3175	NM 183319	Xkrx	2898
NM 183064	Fgf12	5449	NM 030215	Wrnip1	2642	NM 183320	Gm5128	3836
NM_001161850	Sgk1	2603	NM_030218	Misp	2427	NM_001284205	1700029F12Rik	1098
NM 030684	Trim34a	2600	NM 001277957	Tafla	2391	NM 001162884	Igsf10	10084
NM 001161854	Csde1	4108	NM 030219	Trim42	2465	NM 001162908	Sesn1	3182

NM_183091	Tonsl	4239	NM_001277958	Taf1a	2419	NM_031256	Plekha3	3007
NM_183094	Xlr4c	1247	NM_001277959	Taf1a	2398	NM_031258	Chrdl1	4190
NM_030687	Slco1a4	3847	NM_181595	Ppp1r9a	9547	NM_011654	Tuba1b	1747
NM_001281977	Invs	5622	NM_001161365	Rin3	3754	NM_001284319	Ncoa4	4035
NM_011541	Tcea1	2727	NM_001161366	Tdrd6	7062	NM_031374	Tex15	8408
NM_001281978	Invs	5900	NM_001161367	Tdrd6	7041	NM_011657	Tulp3	3401
NM_001162364	Gm14525	890	NM_181649	Gpatch11	4237	NM_183283	Smco1	1267
NM_001162375	Fam73a	5163	NM_011413	C4a	5369	NM_031382	Tex16	3972
NM_001162387	Pex11b	2019	NM_030229	Polr3h	2514	NM_001162926	Fam84b	5563
NM_030696	Slc16a3	2493	NM_011419	Kdm5d	5471	NM_011666	Uba3	2443
NM_011561	Tdg	3119	NM_181682	Dsg1b	3404	NM_031388	Usp26	4133
NM_030703	Cpn1	1792	NM_030233	Saal1	4699	NM_031390	Pramel3	3673
NM_183142	Alg11	4844	NM_181729	Muc6	8553	NM_031391	Gtf2a1	5704
NM_001162417	Myef2	2402	NM_024284	Hagh	1197	NM_031392	Wdr6	4169
NM_030708	Zfhx4	13874	NM_011431	Eftud2	3397	NM_001162938	Pydc3	2987
NM_030712	Cxcr6	1893	NM_011432	Snrpc	775	NM_183335	Igsf1	2513
NM_030713	Zfp202	3877	NM_024454	Rab21	1856	NM_183336	Igsf1	2686
NM_001282054	Msh4	3242	NM_001161665	Kif26b	7422	NM_031395	Sytl3	2308
NM_183146	A530054K11Rik	5180	NM_181816	Ccdc67	3045	NM_183368	Sytl3	1504
NM_011579	Tgtp1	2809	NM_024291	Ky	5455	NM_183370	Sytl3	1722
NM_030722	Pum1	5370	NM_011446	Sox7	3266	NM_183391	Tnfsf18	2064
NM_030723	Pum2	6235	NM_024414	Stx1b	1574	NM_011678	Usp4	3686
NM_011584	Nr1d2	4640	NM_011452	Serpinb9b	1930	NM_031494	Zfp275	6400
NM_011585	Tia1	4477	NM_011454	Serpinb6b	1859	NM_184053	Calu	3229
NM_001162477	Gab2	6005	NM_030675	Krit1	6105	NM_011682	Utrn	12382
NM_030730	Rad54l2	9342	NM_001161714	Tgm1	2755	NM_031870	Msh4	3333
NM_030731	Trim23	4258	NM_001161715	Tgm1	2854	NM_031871	Ghdc	2162
NM_183165	Pyroxd1	2155	NM_011465	Spta1	8405	NM_194058	Nlrp9b	3346
NM_011599	Tle1	11550	NM_001161737	Sival	587	NM_001162950	Hif3a	6916
NM_011603	Tbpl1	2880	NM_182782	Klhl25	2314	NM_001284429	Smtn	3399
NM_183185	Zfp300	4658	NM_024499	Sgta	1966	NM_194061	D630045J12Rik	10986
NM_183188	Rbfox1	4102	NM_011479	Sptlc2	3754	NM_011692	Vbp1	1601
NM_183193	Foxi2	2591	NM_030245	Tada1	2159	NM_194066	Ifi27	1023
NM_030749	Sil1	1715	NM_011481	Srms	2547	NM_194067	Ifi27	918
NM_183194	Gsdmc3	2142	NM_182808	Fam19a1	3252	NM_194068	Ifi27	888
NM_183389	Duxbl1	2028	NM_030248	Cdk5rap3	1892	NM_194069	Ifi27	768
NM_030886	Ankrd17	10066	NM_030249	Cttnbp2nl	4946	NM_001162979	Ccdc81	2691
NM_183201	Slfn5	5989	NM_182839	Tppp	5108	NM_001162980	1700024P16Rik	3136
NM_183208	Zmiz1	7526	NM_030252	Smim12	1037	NM_001162983	Lrrc38	2327
NM_011626	Tmem165	1896	NM_182841	Tmem150c	3100	NM_194334	Tbc1d2b	5951
NM_031176	Tnxb	9569	NM_001281475	Gm1110	2385	NM_011703	Vipr1	4929
NM_011635	Trap1a	1174	NM_001161763	Fmo5	5202	NM_032003	Enpp5	2558
NM_183310	Ccser1	5654	NM_001161765	Fmo5	5285	NM_197940	Wipf2	7589
NM_001162868	Rab11fip3	4880	NM_001161767	Galnt6	5715	NM_001284522	Larp4	6550
NM_001162869	Rab11fip3	5015	NM_182929	Rims3	977	NM_032007	Mmp1b	1997
NM_031185	Akap12	6262	NM_001281491	Syngap1	4023	NM_001284523	Larp4	6543
NM_001001650	Prss48	949	NM_001161768	Galnt6	5712	NM_032008	Slmap	4501
NM_031186	Ndst3	5361	NM_182930	Plekha6	7397	NM_032393	Map1a	11837
NM_001284227	Flot2	2711	NM_030261	Sesn3	2158	NM_032396	Kremen1	4943
NM_001162884	Igsf10	10084	NM_182994	Arl5a	5212	NM_194336	Gbp6	4329
NM_001162896	4930523C07Rik	4035	NM_001161790	Mefv	3249	NM_011717	Wiz	4247
NM_011650	Tsn	3040	NM_182996	Zfp692	1949	NM_032465	Cd96	2203
NM_031251	Ctns	3010	NM_182997	Prkab2	4278	NM_032543	Rnf123	4332

NM 183262	Stk35	6204	NM 001281795	Slc25a44	3459	NM 001163024	Mon2	9300
NM 031256	Plekha3	3007	NM 011494	Stk16	2885	NM 001163025	Mon2	9282
NM 031257	Plekha2	4992	NM 001161796	Gucy1b3	3234	NM 194343	Trim45	4615
NM 031258	Chrdl1	4190	NM 001281816	Sec23b	2421	NM 194344	Sh3tc1	4549
NM 001284319	Ncoa4	4035	NM 030556	Slc19a3	2974	NM 011729	Ercc5	4010
NM 031376	Pik3ap1	2615	NM 183016	Cdc42bpb	6718	NM 011738	Ywhah	1806
NM_183281	2310005G13Rik	3067	NM_030561	BC004004	2809	NM_011741	Zan	16481
NM 031386	Tex14	4776	NM 011504	Stxbp3a	2406	NM 001285446	Cbfa2t2	6076
NM 011666	Uba3	2443	NM 011505	Stxbp4	6035	NM 001163061	Zfp949	3351
NM 031388	Usp26	4133	NM 183019	Arhgef4	2738	NM 001163085	Map3k15	4348
NM 001162932	Rmi2	3739	NM 030595	Nbea	10998	NM 197980	Cox19	778
NM 031393	Sytl1	1940	NM 183021	Thada	7896	NM 011753	Zfp26	11407
NM 001162938	Pydc3	2987	NM 011511	Abcc9	7362	NM 033149	B3galt5	4914
NM_001284374	Atf2	4025	NM_030597	Lsm2	896	NM_197990	1700025G04Rik	9789
NM 031395	Sytl3	2308	NM 030598	Rcan2	3263	NM 197991	Emc10	1855
NM 001284376	Atf2	4182	NM 011512	Surf4	2863	NM 001163144	Pcsk5	5783
NM 183391	Tnfsf18	2064	NM 011513	Med22	3466	NM 001163159	Pcyt1a	4951
NM 011678	Usp4	3686	NM 183027	Ap1s3	2892	NM 197996	Tspan15	3605
NM 031405	Srrt	3039	NM 183028	Pcmtd1	5235	NM 001163160	Pcyt1a	4885
NM 184053	Calu	3229	NM 011520	Sdc3	4972	NM 001163161	Clec4d	1333
NM 011682	Utrn	12382	NM 183033	Zfp516	7725	NM 001285480	Hp1bp3	4615
NM 001284399	Ddhd1	5173	NM 183038	Defb39	252	NM 001285481	Hp1bp3	4813
NM 031870	Msh4	3333	NM 001161845	Sgk1	3121	NM 001163170	Lix1l	2509
NM 031871	Ghdc	2162	NM 001161847	Sgk1	2622	NM 198003	Zfp946	2944
NM 001162947	Nek3	1989	NM 001161848	Sgk1	2415	NM 011762	Zfp59	3815
NM 194058	Nlrp9b	3346	NM 001281955	Csmd2	13555	NM 011764	Zfp90	2632
NM 001162950	Hif3a	6916	NM 001161849	Sgk1	2496	NM 198010	Ankrd17	9313
NM 011686	Vmn2r88	2574	NM 001161850	Sgk1	2603	NM 011768	Zfx	6944
NM 011692	Vbp1	1601	NM 001161851	Fbxo44	1586	NM 011769	Zim1	3528
NM 001162957	Rsph4a	3650	NM 001161852	Fbxo44	1738	NM 198014	Slain1	2757
NM 001162973	Lrrc51	811	NM 183087	Fam189a1	4790	NM 001163195	Gm14692	690
NM 001162974	Lrrc51	939	NM 183089	Dscc1	1327	NM 001285512	Cops2	3380
NM 031997	Tmem2	6702	NM 183091	Tonsl	4239	NM 001285513	Cops2	3128
NM 031998	Cep41	3393	NM 001281977	Invs	5622	NM 001285514	Cnot6l	8570
NM 001162977	Megf6	6876	NM 001281978	Invs	5900	NM 198020	Trmt1	2186
NM_001162980	1700024P16Rik	3136	NM_001162368	Mgat4c	3862	NM_198021	Scyl2	3431
NM 001284520	Cmah	9264	NM 001162369	Mgat4c	3735	NM 001163223	Zfp804b	4032
NM 001162996	Eml2	2255	NM 183111	Rfpl3s	792	NM 001285529	Tle1	11547
NM 001284522	Larp4	6550	NM 001281999	Rasal1	3259	NM 001285530	Tle1	11328
NM 001162999	Fnip2	7095	NM 030690	Rai14	4929	NM 011780	Adam23	6656
NM 001284523	Larp4	6543	NM 001162375	Fam73a	5163	NM 198027	Alkbh6	963
NM_032008	Slmap	4501	NM_183113	4932414N04Rik	3643	NM_198028	Serpinb10	3490
NM 032393	Map1a	11837	NM 030692	Sacm1l	3433	NM 011781	Adam25	2640
NM 011711	Fmnl3	4428	NM 001162387	Pex11b	2019	NM 033590	Pcdhga7	4660
NM 194336	Gbp6	4329	NM 001162388	Pex11b	837	NM 001285785	Arhgap9	2372
NM 194462	Akap9	12105	NM 030695	Lrba	9900	NM 001163267	Ccdc147	3345
NM_011716	Wfs1	3564	NM_183126	6030498E09Rik	1184	NM_033592	Pcdhga9	4717
NM_011717	Wiz	4247	NM_183131	4930451I11Rik	511	NM_001163283	Zbtb5	4737
NM 001163014	Gp6	1152	NM 030697	Kank3	2634	NM 001163284	Zbtb5	5632
NM 194340	Prr14l	10546	NM 011553	Tcp10a	2314	NM 011790	Arih2	3924
NM 032543	Rnf123	4332	NM 030702	Senp3	2442	NM 001163288	Susd1	3295
NM 194343	Trim45	4615	NM 001162414	Nlrp1b	4131	NM 001285806	Adrbk2	6617
NM 194344	Sh3tc1	4549	NM 011565	Tead2	2115	NM 198106	Slc9c1	3879
NM 198110	Gnl3l	4877	NM 183141	Elfn2	4877	NM 011799	Cdc6	4608
NM 194345	Fam160b2	4051	NM 030708	Zfhx4	13874	NM 198111	Akap6	10263
NM 011729	Ercc5	4010	NM 011571	Tesk1	3620	NM 011801	Cfdp1	1215

NM 011734	Siae	3433	NM 001162423	Arhgap4	3288	NM 198113	Ssh3	2728
NM 011738	Ywhah	1806	NM 001162424	Arhgap4	3258	NM 011804	Creg1	2138
NM 194355	Spire1	5033	NM 030713	Zfp202	3877	NM 001163311	Srr	3473
NM 011741	Zan	16481	NM 011576	Tfpi	1492	NM 001163314	Pgap1	10579
NM_011742	Zfp1	1832	NM_183146	A530054K11Rik	5180	NM_198127	Abi2	5790
NM 001163061	Zfp949	3351	NM 030719	Gatsl2	4807	NM 033373	Krt23	1560
NM 194464	Mrvi1	6211	NM 011585	Tia1	4477	NM 011807	Dlg2	7409
NM 011748	Zfp14	3491	NM 183154	Zfyve1	3869	NM 001163328	Ralyl	2426
NM 197980	Cox19	778	NM 001162477	Gab2	6005	NM 198168	Ppp2r5b	2708
NM 033322	Lztfl1	3438	NM 030730	Rad54l2	9342	NM 001163329	Ralyl	2019
NM 001163103	Ppp1r36	1453	NM 001282096	Tjp3	3004	NM 001285839	Osgepl1	1868
NM 011753	Zfp26	11407	NM 183166	Gm4884	2852	NM 001163330	Ralyl	1846
NM 011754	Zfp27	3524	NM 011599	Tle1	11550	NM 198170	Szt2	10958
NM 001163138	Card6	4531	NM 183185	Zfp300	4658	NM 001163332	Cttnbp2nl	4838
NM_197990	1700025G04Rik	9789	NM_011606	Clec3b	1009	NM_001163333	Cttnbp2nl	4830
NM 001163144	Pcsk5	5783	NM 183186	Foxn3	2611	NM 198191	Pip5kl1	1489
NM 197992	Pcgf1	908	NM 001162523	Ceacam12	1505	NM 198192	Qrfpr	1816
NM 011760	Zfp54	2536	NM 183188	Rbfox1	4102	NM 001163348	Ntng1	5179
NM_197995	Arl16	2023	NM_001162537	9330159F19Rik	5644	NM_011820	Ggt5	4137
NM 001285480	Hp1bp3	4615	NM 183193	Foxi2	2591	NM 001163349	Ntng1	5044
NM 001285481	Hp1bp3	4813	NM 001162538	Odf2l	2584	NM 001163350	Ntng1	5170
NM 033570	Cnnm4	4582	NM 001162539	Odf2l	2296	NM 033616	Csprs	2719
NM 001285491	Gnb1l	3621	NM 001162540	Stk32c	1883	NM 011823	Gpr34	1898
NM 011762	Zfp59	3815	NM 001283011	Rad17	2940	NM 198249	Arhgef40	5622
NM 001285493	Gnb1l	3660	NM 183201	Slfn5	5989	NM 033524	Spred1	6319
NM 198006	Coa5	3862	NM 030889	Sorcs2	5722	NM 001285875	Pafah2	3086
NM 198010	Ankrd17	9313	NM 183224	Fam19a3	4317	NM 001285877	Pafah2	2830
NM 011769	Zim1	3528	NM 031174	Dscam	7481	NM 011835	Katna1	1775
NM 198017	Fam175b	2895	NM 183225	Usp24	10601	NM 198295	Tmx3	4611
NM 001285512	Cops2	3380	NM 183300	Zfyve9	2170	NM 198302	Rbm11	2699
NM 001285513	Cops2	3128	NM 133626	Rrbp1	2706	NM 033623	Dcun1d1	4421
NM 001285514	Cnot6l	8570	NM 183311	Tmem145	2298	NM 198303	Eif5b	7797
NM_198020	Trmt1	2186	NM_001162868	Rab11fip3	4880	NM_011845	Mid2	5916
NM_001163223	Zfp804b	4032	NM_001162869	Rab11fip3	5015	NM_198304	Nup188	5670
NM 001285529	Tle1	11547	NM 183320	Gm5128	3836	NM 052973	Strn3	4164
NM 001285530	Tle1	11328	NM 183321	Proser3	2612	NM 011849	Nek4	2714
NM 011780	Adam23	6656	NM 031185	Akap12	6262	NM 052993	C1galt1	2084
NM 001163246	AA987161	4835	NM 001284227	Flot2	2711	NM 052994	Spock2	3931
NM_198028	Serpinb10	3490	NM_011651	Tsks	1828	NM_198409	Rai2	2276
NM 011781	Adam25	2640	NM 001162908	Sesn1	3182	NM 198411	Inf2	4618
NM 011782	Adamts5	8104	NM 183262	Stk35	6204	NM 001163425	Myeov2	458
NM 198033	Setx	10970	NM 001284272	Ttk	2733	NM 001285916	Gmnc	4448
NM 001163283	Zbtb5	4737	NM 031255	Rsph6a	2357	NM 001285917	Dapk1	5398
NM 001163284	Zbtb5	5632	NM 031257	Plekha2	4992	NM 001285918	Gmnc	4179
NM 001285806	Adrbk2	6617	NM 031258	Chrdl1	4190	NM 001163433	Samd4	6600
NM 011795	C1ql1	1260	NM 031260	Mov10l1	4053	NM 053072	Fgd6	8061
NM 198105	Fam120c	7709	NM 031373	Ogfr	2306	NM 001285933	Kctd9	3451
NM 198106	Slc9c1	3879	NM 031374	Tex15	8408	NM 001163457	Mttp	4272
NM_198111	Akap6	10263	NM_183281	2310005G13Rik	3067	NM_198606	Dcaf13	1543
NM 011803	Klf6	4217	NM 031378	Gsdmc	2382	NM 001285949	Fez2	3767
NM_001163311	Srr	3473	NM_031379	Tktl1	2485	NM_001285952	1700024P16Rik	3214
NM_001163314	Pgap1	10579	NM_183283	Smco1	1267	NM_001285953	1700024P16Rik	3069
NM 198127	Abi2	5790	NM 001162924	Pkp3	2926	NM 001163467	Phtf1	2936

NM 033612	Cela1	1124	NM 001284359	Nprl3	2904	NM 001163468	Phtf1	3183
NM 001163319	Tubgcp6	6957	NM 001284360	Nprl3	2735	NM 001163469	Phtf1	3207
NM 011807	Dlg2	7409	NM 031386	Tex14	4776	NM 198612	Gxylt2	1826
NM 198170	Szt2	10958	NM 001284370	Atf2	3679	NM 011878	Tiam2	6128
NM 011813	Fiz1	2656	NM 183289	Tcergl1	2569	NM 001163470	Trafd1	2498
NM 011814	Fxr2	3002	NM 001284371	Atf2	3616	NM 053092	Kars	2045
NM 198176	Fastkd5	3164	NM 011669	Usp12	4274	NM 198614	C2cd4c	6764
NM 001163336	Atp2a3	4520	NM 001284374	Atf2	4025	NM 001163471	Hectd2	4774
NM 001163337	Atp2a3	4606	NM 031395	Sytl3	2308	NM 011882	Rnasel	4758
NM 198192	Qrfpr	1816	NM 001284376	Atf2	4182	NM 001163475	Zfp746	3651
NM 011818	Gmcl1	2967	NM 011674	Ugt8a	3599	NM 053103	Entpd7	6050
NM 001285852	Sgip1	5589	NM 183368	Sytl3	1504	NM 053105	Klhl1	4087
NM 011821	Gpc6	6750	NM 183370	Sytl3	1722	NM 001285988	Cdc27	5798
NM 001163354	Rhot1	4233	NM 183390	Klhl6	2405	NM 198631	Zc3h4	6107
NM 001285862	Sgip1	4851	NM 031403	Dbr1	2103	NM 001285989	Cdc27	5780
NM 001163355	Rhot1	4260	NM 183391	Tnfsf18	2064	NM 001285990	Cdc27	5703
NM 001163356	Fam212b	4554	NM 011682	Utrn	12382	NM 198632	Trim67	8832
NM 011825	Grem2	3745	NM 031863	Cenpq	1442	NM 011894	Sh3bp5	2487
NM 001285864	Pphln1	3679	NM 031871	Ghdc	2162	NM 001285991	Tbc1d5	5682
NM 033524	Spred1	6319	NM 001284429	Smtn	3399	NM 001285992	Scoc	1943
NM 001163359	Fignl1	2974	NM 011686	Vmn2r88	2574	NM 011899	Srp54a	4264
NM_001163360	Fignl1	2844	NM_011692	Vbp1	1601	NM_198642	5031414D18Rik	2651
NM 011832	Insrr	5102	NM 001162957	Rsph4a	3650	NM 011902	Tekt2	1500
NM 001285875	Pafah2	3086	NM 001162965	Sgsm1	3825	NM 011904	Tll2	3369
NM 001285877	Pafah2	2830	NM 001162970	Aim1l	4781	NM 198649	Ablim3	4545
NM 011836	Lamc3	5930	NM 031997	Tmem2	6702	NM 001163501	Elmsan1	6856
NM 001285888	Zmym6	4236	NM 001162979	Ccdc81	2691	NM 001286003	Scyl3	4411
NM 001285890	Pde1b	3245	NM 194333	Slc23a3	2077	NM 001163502	Elmsan1	7266
NM_011843	Esyt1	3687	NM_001162980	1700024P16Rik	3136	NM_053117	Pard6g	3143
NM 198302	Rbm11	2699	NM 001162989	Phax	1783	NM 001163507	Mmrn1	4633
NM 198303	Eif5b	7797	NM 001162998	Smim6	975	NM 001286009	Tubgcp2	2894
NM 011846	Mmp17	3489	NM 032008	Slmap	4501	NM 001286011	Tubgcp2	2925
NM 198305	Klhl17	3014	NM 032393	Map1a	11837	NM 198657	Gm5148	1215
NM 001163401	Il11ra1	1761	NM 032394	Myo7b	6591	NM 001163511	Spata5	2910
NM 011848	Nek3	2044	NM 011711	Fmnl3	4428	NM 198658	Nr1h5	2835
NM 198306	Galnt9	2891	NM 011712	Wbp5	1018	NM 198659	Stpg2	2818
NM 011849	Nek4	2714	NM 194462	Akap9	12105	NM 198661	Oog2	1929
NM 198311	Ttc8	2287	NM 001163013	Ythdc2	6299	NM 011916	Xrn1	5515
NM 198322	Zfp273	2321	NM 011718	Wnt10b	2272	NM 011919	Ing1	2813
NM 052993	C1galt1	2084	NM 032465	Cd96	2203	NM 198674	Fbxw26	1824
NM 053009	Zfp91	5624	NM 001163014	Gp6	1152	NM 198675	Fdxacb1	1978
NM 001163419	Fam172a	3912	NM 194340	Prr14l	10546	NM 011922	Anxa10	1780
NM 001163420	Fam172a	3720	NM 032610	Sptbn4	8737	NM 011930	Clcn7	4063
NM 001285916	Gmnc	4448	NM 198108	Morn4	1817	NM 011932	Dapp1	3088
NM_198417	C030039L03Rik	4628	NM_001163026	Dnajc13	7697	NM_001163548	Cyth3	4073
NM 001285917	Dapk1	5398	NM 001285435	Adamtsl5	3041	NM 011940	Ifi202b	1793
NM 001285918	Gmnc	4179	NM 011729	Ercc5	4010	NM 198865	Slitrk5	4829
NM 198418	Tdrd6	7059	NM 001163029	Cd97	2971	NM 001163559	Lrriq1	5232
NM 001163433	Samd4	6600	NM 001163030	Cd97	3118	NM 001163567	Fam102b	5702
NM 011858	Tenm4	10633	NM 001163031	Cd97	2965	NM 011958	Orc4	4029
NM 001285927	Dock10	7324	NM 011737	Map3k19	3936	NM 001163572	Tmem170b	7170
NM 053072	Fgd6	8061	NM 194348	Atg2a	6340	NM 001286214	Zfp386	4544
NM 001163447	Mapk8ip3	5576	NM 011738	Ywhah	1806	NM 011962	Plod3	3278
NM 198424	Orai3	1996	NM 194355	Spire1	5033	NM 001163575	Parp10	3307
NM 001285933	Kctd9	3451	NM 194464	Mrvi1	6211	NM 001163576	Parp10	3411
NM 001163448	Mapk8ip3	5551	NM 033320	Glce	4626	NM 011964	Psg19	2547
NM 001163449	Mapk8ip3	5510	NM 033322	Lztfl1	3438	NM 001286263	Ralgapa1	8421
NM 001163450	Mapk8ip3	5486	NM 001163103	Ppp1r36	1453	NM 011965	Psma1	1255
NM 001163451	Mapk8ip3	5483	NM 011753	Zfp26	11407	NM 011966	Psma4	1096

NM 001163453	Mapk8ip3	5468	NM 011754	Zfp27	3524	NM 198931	Ppm1m	1848
NM 198599	Map6d1	3290	NM 001163143	C2cd4a	1352	NM 001163592	Nhsl1	6812
NM_053083	Loxl4	5444	NM_197990	1700025G04Rik	9789	NM_011972	Poli	2482
NM 011869	Med24	3427	NM 033321	P2rx5	2293	NM 011976	Sema4g	4310
NM 054037	Scgb3a1	540	NM 001163161	Clec4d	1333	NM 198942	Dhx57	4935
NM 053085	Tcf23	4101	NM 197999	Ces2g	2859	NM 001286376	Stox2	10521
NM 198606	Dcaf13	1543	NM 033268	Actn2	3091	NM 001286382	Vkorc1l1	4797
NM_001285952	1700024P16Rik	3214	NM_198006	Coa5	3862	NM_198962	Hcrtr2	3728
NM 198609	Rsl24d1	1522	NM 001163175	Begain	2728	NM 053149	Hemgn	2310
NM 053088	Ifitm5	734	NM 033314	Slco2a1	4033	NM 001163625	Sult6b1	2060
NM_001285953	1700024P16Rik	3069	NM_198012	Trim68	2389	NM_053155	Clmn	11962
NM 053089	Naa15	6090	NM 033574	Pcdhgb1	4498	NM 053156	Allc	1460
NM 011877	Ptpn21	5697	NM 011768	Zfx	6944	NM 001163630	Satb1	6337
NM 001163471	Hectd2	4774	NM 001163189	Enthd1	2110	NM 001163631	Satb1	6205
NM 011880	Rgs7	2424	NM 011769	Ziml	3528	NM 199012	Fchsd2	4432
NM 001163472	Cyp2d22	2788	NM 033576	Pcdhgb4	4480	NM 001163632	Satb1	6178
NM 011882	Rnasel	4758	NM 011771	Ikzf3	4098	NM 001163635	Tnks2	6503
NM 011883	Rnf13	1116	NM 033579	Pcdhgb7	4531	NM 199016	Enpp4	4558
NM 001163475	Zfp746	3651	NM 198021	Scyl2	3431	NM 001286395	Npr3	6540
NM 053103	Entpd7	6050	NM 033581	Pcdhgc3	4687	NM 001163637	Jakmip2	3511
NM 011889	3-Sep	4731	NM 001163223	Zfp804b	4032	NM 053165	Clec2h	2160
NM 053105	Klhl1	4087	NM 033583	Pcdhgc5	4708	NM 001163638	Ttc18	3632
NM 011891	Sgcd	1399	NM 001285529	Tle1	11547	NM 001163639	Ttc18	3551
NM 011892	Sgcg	3515	NM 011779	Coro1c	3436	NM 001163640	Chn2	3150
NM 053107	Gpr45	3860	NM 001285530	Tle1	11328	NM 011990	Slc7a11	9227
NM 198634	Tigd3	2147	NM 198024	Ranbp3l	4298	NM 011991	Cops3	1583
NM 011899	Srp54a	4264	NM 011780	Adam23	6656	NM 199018	Stard8	4983
NM_198642	5031414D18Rik	2651	NM_001163262	Cmip	4539	NM_001163645	Osbpl3	6779
NM 198647	Tbc1d22b	3505	NM 011781	Adam25	2640	NM 011994	Abcd2	5540
NM 053114	Pabpc5	2434	NM 001163266	Lsm10	825	NM 199021	Dpp10	4648
NM 198649	Ablim3	4545	NM 001163267	Ccdc147	3345	NM 001163663	Rab6a	3243
NM 053118	Gprc5d	1324	NM 198033	Setx	10970	NM 053171	Csmd1	14278
NM 001163505	Atl3	6485	NM 198034	Sidt1	4266	NM 199025	Zbtb26	5290
NM 001286009	Tubgcp2	2894	NM 198035	Zbtb39	5970	NM 053178	Acsbg1	2775
NM 001286011	Tubgcp2	2925	NM 033594	Pcdhga11	4764	NM 199028	Bend3	6049
NM 198658	Nr1h5	2835	NM 001163288	Susd1	3295	NM 012003	Cops7a	1775
NM 001163513	Dlg5	7878	NM 198095	Bst2	866	NM 012005	Med14	5083
NM_198660	E230008N13Rik	5081	NM_001285806	Adrbk2	6617	NM_199036	Fbxw15	1520
NM 011918	Ldb3	5040	NM 011795	C1ql1	1260	NM 053185	Col4a6	6648
NM 198667	BC061212	1625	NM 198105	Fam120c	7709	NM 012008	Ddx3y	4640
NM_198680	Serpinb3b	1632	NM_198106	Slc9c1	3879	NM_199057	Rusc2	5278
NM 011924	Avpr1b	4148	NM 033602	Peli2	5849	NM 053187	Gopc	4295
NM 198702	Lphn3	5812	NM 011799	Cdc6	4608	NM 199068	Foxk1	7462
NM 011930	Clcn7	4063	NM 198111	Akap6	10263	NM 053191	Pi15	6946
NM 001163533	Rtdr1	1203	NM 198113	Ssh3	2728	NM 001286469	Xkr5	3928
NM 001163534	Rtdr1	1190	NM 198114	Dagla	5652	NM 012014	Gprin1	4141
NM 001163535	Rtdr1	1187	NM 198119	Lrrc24	1904	NM 001163702	Fbxo27	1966
NM 001286080	Nin	6791	NM 033609	Med15	3413	NM 053197	Sfxn3	2974
NM 011937	Gnpda1	2300	NM 001163314	Pgap1	10579	NM 053198	Sfxn4	2731
NM 198863	Slitrk2	7754	NM 001285815	Tmem53	1103	NM 001163703	Dcun1d3	5618
NM_001163550	1700016H13Rik	787	NM_001285816	Tmem53	883	NM_145079	Ugt1a6a	3302
NM 011941	Mapkbp1	6954	NM 198160	Smarcc2	4622	NM 012024	Ppp2r5e	4841
NM 001163552	Ap4b1	2701	NM 033354	Sec16b	4611	NM 012026	Arhgef28	6259
NM 001163553	Ap4b1	2830	NM 033373	Krt23	1560	NM 199146	Trim30d	3770
NM 011946	Map3k2	10791	NM 001163319	Tubgcp6	6957	NM 012032	Serinc3	3694

NM_001163559	Lrriq1	5232	NM_011807	Dlg2	7409	NM_012035	Trpc7	3339
NM_001163567	Fam102b	5702	NM_198167	Tmem63b	3237	NM_012038	Vsnl1	1951
NM_001163570	Exosc8	1282	NM_198170	Szt2	10958	NM_012042	Cul1	3171
NM_011957	Creb3l1	2284	NM_001285841	Sybu	2295	NM_001163748	Pde9a	2061
NM_011958	Orc4	4029	NM_001163332	Cttnbp2nl	4838	NM_012055	Asns	1927
NM_011959	Orc5	2059	NM_198171	Ces2b	1912	NM_199157	Ifnk	872
NM_001163572	Tmem170b	7170	NM_001285842	Sybu	2439	NM_001163756	Ces2e	2007
NM_001286214	Zfp386	4544	NM_001285843	Sybu	2506	NM_001286629	Gimap8	3158
NM_001163575	Parp10	3307	NM_001163333	Cttnbp2nl	4830	NM_001163759	Dhx57	5094
NM_001163576	Parp10	3411	NM_011814	Fxr2	3002	NM_001286647	Atl2	3924
NM_011964	Psg19	2547	NM_001285844	Sybu	2398	NM_001163767	Tctex1d1	1997
NM_011965	Psma1	1255	NM_198176	Fastkd5	3164	NM_001163768	Tctex1d1	1904
NM_011972	Poli	2482	NM_001163336	Atp2a3	4520	NM_199305	Tmem39b	1845
NM_001286362	Tcf25	3001	NM_001163337	Atp2a3	4606	NM_001163776	Tmprss3	2878
NM_001163608	Plxdc1	2922	NM_198191	Pip5kl1	1489	NM_053255	Elac1	4971
NM_198942	Dhx57	4935	NM_001163346	Cdc42ep4	2960	NM_013415	Atp1b2	2959
NM_011980	Zfp146	2045	NM_033474	Arvcf	4738	NM_001163794	Pdik1l	4639
NM_001286376	Stox2	10521	NM_011825	Grem2	3745	NM_001286750	Olfm3	4285
NM_001286382	Vkorc1l1	4797	NM_033524	Spred1	6319	NM_013485	C9	1767
NM_198962	Hcrtr2	3728	NM_011829	Impdh1	2436	NM_053268	Rasa2	5699
NM_001286385	Slc7a15	1086	NM_011830	Impdh2	1632	NM_001286759	Rap1gds1	3671
NM_001286386	Trim9	4810	NM_198292	Tex2	4947	NM_013489	Cd84	3290
NM_053155	Clmn	11962	NM_001285875	Pafah2	3086	NM_199446	Phkb	4640
NM_199009	Fam160a2	10853	NM_001285877	Pafah2	2830	NM_001163847	Tbc1d24	7728
NM_001163630	Satb1	6337	NM_011835	Katna1	1775	NM_001163848	Tbc1d24	7266
NM_001163631	Satb1	6205	NM_001285881	D630023F18Rik	2183	NM_001163849	Tbc1d24	7180
NM_199012	Fchsd2	4432	NM_198295	Tmx3	4611	NM_199467	Mms221	4482
NM_001163632	Satb1	6178	NM_011836	Lamc3	5930	NM_001163850	Tbc1d24	7710
NM_001163635	Tnks2	6503	NM_033541	Oas1c	2286	NM_001163851	Tbc1d24	7248
NM_199016	Enpp4	4558	NM_198296	9130011E15Rik	2896	NM_054040	Tulp4	9714
NM_001286395	Npr3	6540	NM_001285884	Med15	3417	NM_001163852	Tbc1d24	7946
NM_001013616	Trim6	3827	NM_198297	Trat1	684	NM_001163853	Tbc1d24	7774
NM_011990	Slc7a11	9227	NM_198298	Helz	6240	NM_199471	Prss43	1995
NM_011994	Abcd2	5540	NM_001285890	Pde1b	3245	NM_199476	Rrm2b	4532
NM_001286415	Fert2	2948	NM_033623	Dcun1d1	4421	NM_054046	Def8	3786
NM_199021	Dpp10	4648	NM_198303	Eif5b	7797	NM_013513	Epb4.2	3472
NM_001163663	Rab6a	3243	NM_011845	Mid2	5916	NM_201258	Oog3	1868
NM_011996	Adh4	1312	NM_198304	Nup188	5670	NM_054052	B3gnt5	4548
NM_053171	Csmd1	14278	NM_198305	Klhl17	3014	NM_054053	Gpr98	19328
NM_053173	Kifc5b	2627	NM_001163401	Il11ra1	1761	NM_201353	Slc6a7	3362
NM_199025	Zbtb26	5290	NM_198306	Galnt9	2891	NM_054056	Pawr	1828
NM_199027	Zfp335	4610	NM_011849	Nek4	2714	NM_013523	Fshr	2360
NM_199028	Bend3	6049	NM_052976	Ophn1	3932	NM_054064	Psg29	5441
NM_199032	Cep135	5334	NM_001163416	Ptx4	1492	NM_054066	Plcz1	2182
NM_199033	Tsen2	1802	NM_052994	Spock2	3931	NM_054068	Vsx1	3579
NM_001286448	Rtn1	2096	NM_011852	Oas1g	1993	NM_013529	Gfpt2	2986
NM_053183	Ddx50	2507	NM_011854	Oasl2	3136	NM_054070	Afg3l1	4288
NM_199038	Casc4	3948	NM_198411	Inf2	4618	NM_054072	Pcdha1	5248
NM_012007	Ctsj	1282	NM_001163425	Myeov2	458	NM_013533	Gpr162	2676
NM_199042	Thap1	2267	NM_001285917	Dapk1	5398	NM_054078	Baz2a	8369
NM_012009	Sh2d1b1	1433	NM_198418	Tdrd6	7059	NM_201375	Kng2	2305
NM_199068	Foxk1	7462	NM_053015	Mlph	4484	NM_001164058	Prl7a1	977
NM_053191	Pi15	6946	NM_011858	Tenm4	10633	NM_054084	Calcb	962
NM_001286466	Btrc	6126	NM_001285927	Dock10	7324	NM_054088	Pnpla3	4649
NM_053193	Cpsf1	4538	NM_053072	Fgd6	8061	NM_054089	Tgs1	4389
NM_199079	Ddx17	2860	NM_198421	Usp49	5598	NM_201406	Pigs	2505
NM_053194	Ric8	2967	NM_001163447	Mapk8ip3	5576	NM_201641	Ugt1a10	3242
NM_001286469	Xkr5	3928	NM_198423	Bahcc1	10756	NM_201642	Ugt1a7c	3294

NM 199080	Ddx17	4772	NM 001163448	Mapk8ip3	5551	NM 001164090	Bcap29	1791
NM_001163695	4932438H23Rik	2436	NM_053077	Slc45a2	2983	NM_001164089	Cops7a	1327
NM 145079	Ugt1a6a	3302	NM 001163449	Mapk8ip3	5510	NM 201410	Ugt1a6b	2293
NM 053199	Cadm3	4159	NM 001163450	Mapk8ip3	5486	NM 201645	Ugt1a1	3229
NM 001163704	Fbxo6	1429	NM 001163451	Mapk8ip3	5483	NM 201529	Lmo7	6123
NM 001163705	Fbxo6	1213	NM 001163452	Trpc4ap	3219	NM 201518	Flrt2	7099
NM 001163706	Fbxo6	1205	NM 001163453	Mapk8ip3	5468	NM 201519	Map4k5	4490
NM 012024	Ppp2r5e	4841	NM 001285942	Eif3k	767	NM 057172	Fubp1	6491
NM 001163707	Fbxo6	1240	NM 001285943	Eif3k	933	NM 001164159	Ppp6r3	4693
NM 199146	Trim30d	3770	NM 001163455	Tbck	3396	NM 001164167	Pias2	4942
NM 199148	BC051665	1382	NM 001285945	Zranb3	4085	NM 078478	Ghitm	2906
NM_199150	BC049730	1200	NM_001285952	1700024P16Rik	3214	NM_001164168	Pias2	4984
NM 053214	Myo1f	3833	NM 011874	Psmc4	1436	NM 013562	Ifrd1	1836
NM 001286546	Ccpg1	3019	NM 011875	Psmd13	1563	NM 201619	Nr1h5	2811
NM_053217	2010002M12Rik	3365	NM_001285953	1700024P16Rik	3069	NM_031167	Il1rn	2464
NM 012040	Pnck	1582	NM 011878	Tiam2	6128	NM 201622	Nr1h5	2661
NM 001286560	Ergic2	3826	NM 198613	Ap2s1	798	NM 013566	Itgb7	2696
NM 012043	Islr	2243	NM 011880	Rgs7	2424	NM 080420	Lpo	2927
NM 199152	Obscn	23006	NM 001163472	Cyp2d22	2788	NM 001164197	Mmp19	3275
NM 001163734	Rrp1b	4493	NM 011882	Rnasel	4758	NM 203507	Rwdd4a	2971
NM 001286578	C2cd5	4216	NM 198623	Ubqln3	2369	NM 013584	Lifr	9663
NM_012051	Etv3	5140	NM_198630	1810024B03Rik	1266	NM_203508	Eddm3b	1272
NM 001163754	Rab3gap2	7003	NM 198631	Zc3h4	6107	NM 001164198	Prkacb	4337
NM 013458	Add2	8024	NM 011892	Sgcg	3515	NM 080443	Asb7	4893
NM 199199	Tmem199	1353	NM 011894	Sh3bp5	2487	NM 001164199	Prkacb	4210
NM 001163759	Dhx57	5094	NM 053109	Clec2d	1214	NM 001164200	Prkacb	4302
NM 199303	Bpifb6	1667	NM 198635	Gm5134	2838	NM 013586	Loxl3	4049
NM 001163775	Taok2	5270	NM 011899	Srp54a	4264	NM 205810	Mrgprb1	4185
NM 001163776	Tmprss3	2878	NM 011903	Tlk2	5379	NM 080448	Srgap3	8887
NM 199306	Wdtc1	4175	NM 198649	Ablim3	4545	NM 205820	Tlr13	4014
NM 053252	Ehbp1l1	3888	NM 001163501	Elmsan1	6856	NM 013589	Ltbp2	6620
NM 199308	Mast3	5334	NM 001286003	Scyl3	4411	NM 080450	Gjc3	3515
NM_013475	Apoh	1202	NM_198651	4430402I18Rik	2152	NM_205822	Omt2b	735
NM 053255	Elac1	4971	NM 001163502	Elmsan1	7266	NM 206537	Cyp2c54	1781
NM_001163793	C530008M17Rik	6835	NM_001163505	Atl3	6485	NM_001164222	Col4a3bp	5285
NM 001163794	Pdik1l	4639	NM 001163507	Mmrn1	4633	NM 206856	Tacc2	3906
NM_001163797	Zfp68	4725	NM_198660	E230008N13Rik	5081	NM_080462	Hnmt	1653
NM 053263	Hnrnpa3	5176	NM 011913	Best1	2087	NM 080464	Ppp1r3a	7120
NM_013483	Btn1a1	3454	NM_198661	Oog2	1929	NM_001164235	1700007B14Rik	2162
NM 001286750	Olfm3	4285	NM 198667	BC061212	1625	NM 080471	Ankrd6	4573
NM 053268	Rasa2	5699	NM 011921	Aldh1a7	2060	NM 001164249	Tpm1	1702
NM 001286759	Rap1gds1	3671	NM 011925	Cd97	3253	NM 001164250	Tpm1	1702
NM 199447	Rrp12	4344	NM 198710	Sypl	4615	NM 001164252	Tpm1	1675
NM 001286784	Magi1	7803	NM 011929	Clcn6	2910	NM 080556	Tm9sf2	3062
NM 053271	Rims2	7278	NM 011930	Clcn7	4063	NM 001164253	Tpm1	1675
NM 001286785	Magi1	7931	NM 001286080	Nin	6791	NM 080557	Snx4	2442
NM 053272	Dhcr24	4028	NM 001163542	Plec	14972	NM 206958	Ltbp1	6771
NM 001163847	Tbc1d24	7728	NM 001163549	Plec	15018	NM 080558	Ssfa2	5109
NM 013497	Creb3	1582	NM 011941	Mapkbp1	6954	NM 013626	Pam	4149
NM 001163848	Tbc1d24	7266	NM 011946	Map3k2	10791	NM 001164258	Xrra1	2631
NM 199466	Eml4	5125	NM 011947	Map3k3	3161	NM 013629	Phtf1	3342
NM 001163849	Tbc1d24	7180	NM 001163559	Lrriq1	5232	NM 080561	Rnf216	4521
NM 199467	Mms22l	4482	NM 001163567	Fam102b	5702	NM 001164263	Plekhs1	2385
NM 001163850	Tbc1d24	7710	NM 001163571	Senp3	2230	NM 001164272	Cpsf7	3577

NM 001163851	Tbc1d24	7248	NM 001163572	Tmem170b	7170	NM 207110	Rnf216	4692
NM 054040	Tulp4	9714	NM 001163579	Lrrcc1	5675	NM 080634	Hps3	4011
NM 013500	Hapln1	5055	NM 001163580	Lrrcc1	5627	NM 080644	Cacng5	3678
NM 001163852	Tbc1d24	7946	NM 198931	Ppm1m	1848	NM 080708	Bmp2k	6585
NM 001163853	Tbc1d24	7774	NM 001286348	Ldb2	2667	NM 001164312	Gm4847	3012
NM 199477	Mettl7a2	1808	NM 011975	Rpl27a	1112	NM 080727	Tmprss3	2738
NM 001163996	Zfp605	3158	NM 001286363	Tcf25	2776	NM 001164325	Trpm7	7142
NM 201256	Eif4ebp3	1151	NM 011976	Sema4g	4310	NM 080793	Setd7	7356
NM 054050	Rapgef1	5837	NM 198942	Dhx57	4935	NM 013659	Sema4b	3946
NM 013513	Epb4.2	3472	NM 001163611	Nps	860	NM 013661	Sema5b	4556
NM 201258	Oog3	1868	NM 011980	Zfp146	2045	NM 001164355	Ska1	2605
NM 054052	B3gnt5	4548	NM 001286376	Stox2	10521	NM 013663	Srsf3	2585
NM 013515	Stom	2816	NM 198967	Tmtc1	8346	NM 013675	Sptb	10417
NM 054053	Gpr98	19328	NM 001286383	Cklf	571	NM 013678	Surf2	1170
NM 054054	Brdt	4710	NM 001163626	Noxa1	1649	NM 130452	Bbox1	1642
NM 201359	Tmem106c	1641	NM 053155	Clmn	11962	NM 001164376	Ctnna3	3681
NM 054058	Psg20	1428	NM 001286387	Trim9	4677	NM 001164401	Ptpro	6550
NM 201361	Rmdn2	1802	NM 001163628	Lyrm5	1623	NM 001164402	Ptpro	4180
NM 201362	Ccdc68	1525	NM 199009	Fam160a2	10853	NM 001164403	Ptpro	4096
NM_201363	Serpinb3c	1659	NM_001286388	Trim9	4734	NM_130457	Cntnap4	4863
NM 054064	Psg29	5441	NM 001163630	Satb1	6337	NM 001164411	Cnot4	5613
NM 054066	Plcz1	2182	NM 001163631	Satb1	6205	NM 001164412	Cnot4	3335
NM 054070	Afg3l1	4288	NM 011981	Zfp260	3851	NM 001164413	Cnot4	3548
NM 013533	Gpr162	2676	NM 001163632	Satb1	6178	NM 001164414	Cnot4	3344
NM 201371	Prmt8	2413	NM 199013	Irgc1	1831	NM 013691	Thbs3	3205
NM 013536	Emg1	1086	NM 011985	Mmp23	1434	NM 013694	Tnp2	724
NM 054078	Baz2a	8369	NM 053166	Trim7	1366	NM 013699	Ubp1	3749
NM 054079	Iltifb	1111	NM 001163640	Chn2	3150	NM 001164441	Ankrd33b	7498
NM_201376	Serpinb3d	1164	NM_053167	Trim9	4680	NM_013701	Ugt1a2	3206
NM 013546	Hebp1	1086	NM 199018	Stard8	4983	NM 001164472	Nip7	2895
NM 054087	Slc19a2	3571	NM 001163645	Osbpl3	6779	NM 130879	Usp48	5766
NM 054088	Pnpla3	4649	NM 001286415	Fert2	2948	NM 207208	Clca6	2998
NM 054089	Tgs1	4389	NM 199021	Dpp10	4648	NM 001164483	Synj1	7085
NM 001164078	Tia1	4450	NM 012000	Cln8	6432	NM 001164491	Ablim3	4226
NM 201641	Ugt1a10	3242	NM 199028	Bend3	6049	NM 207216	Ugt3a1	2250
NM 201642	Ugt1a7c	3294	NM 012002	Cops6	1094	NM 001164497	Papd5	4642
NM 001164099	Add3	4449	NM 199029	Zfp395	4229	NM 001164498	Papd5	4616
NM 001164100	Add3	4415	NM 199032	Cep135	5334	NM 001164499	Papd5	4513
NM 001164101	Add3	4353	NM 199033	Tsen2	1802	NM 013724	Nrk	6604
NM 054097	Pip4k2c	3374	NM 053182	Pag1	8197	NM 001164503	Akap11	9416
NM_201410	Ugt1a6b	2293	NM_199034	4922502D21Rik	1596	NM_013726	Dbf4	2444
NM 201645	Ugt1a1	3229	NM 012006	Acot1	1559	NM 130903	Cd209c	1569
NM 201411	Flrt1	5876	NM 199038	Casc4	3948	NM 130904	Cd209d	880
NM 054102	Ivns1abp	3513	NM 199042	Thap1	2267	NM 013734	Atp1a4	3485
NM 201530	Sly	918	NM 053191	Pi15	6946	NM 133186	Steap3	2769
NM 057172	Fubp1	6491	NM 012011	Eif2s3y	1801	NM 207234	Rexo4	2354
NM 201609	Zfp652	2668	NM 053193	Cpsf1	4538	NM 001164559	Trmt1	2159
NM_078478	Ghitm	2906	NM_199145	3110062M04Rik	3725	NM_001164560	Trmt1	2083
NM 078484	Slc35a2	1569	NM 001163703	Dcun1d3	5618	NM 207237	Man1c1	4715
NM 201619	Nr1h5	2811	NM 012023	Ppp2r5c	4238	NM 207238	Fbxo27	1961
NM 201622	Nr1h5	2661	NM 001163709	Bri3	847	NM 013751	Hrasls	2915
NM 001164173	Cpsf1	4513	NM 001163729	Klhdc3	1973	NM 001164573	Myo1h	4631
NM 201646	Btbd6	2075	NM 012035	Trpc7	3339	NM 133204	Zscan5b	1969
NM 013566	Itgb7	2696	NM 012039	Zw10	2917	NM 001164575	Zfp169	4170
NM 080434	Apoa5	2321	NM 012040	Pnck	1582	NM 207244	Cd200r4	1700
NM 203319	Dhx37	4235	NM 012043	Islr	2243	NM 013760	Dnajb9	1865
NM 001164197	Mmp19	3275	NM 199152	Obscn	23006	NM 013761	Srr	3699
NM 203508	Eddm3b	1272	NM 001286578	C2cd5	4216	NM 133207	Kcnh7	3798

NM_080443	Asb7	4893	NM_001163748	Pde9a	2061	NM_207246	Rasgrp3	4720
NM_013589	Ltbp2	6620	NM_001163749	Camsap3	4306	NM_001164583	Dnajc6	5220
NM_080451	Synpo2	7116	NM_012055	Asns	1927	NM_001164584	Dnajc6	5165
NM_001164207	Tmem176b	1421	NM_012060	Bcap31	1214	NM_001164585	Dnajc6	5230
NM_001164208	Tmem176b	1069	NM_001163754	Rab3gap2	7003	NM_207263	Pglyrp4	2226
NM_001164209	Tmem176b	1277	NM_013455	Acr	1504	NM_001164606	Ccdc116	3926
NM_001164212	Rerg	2254	NM_013457	Add1	3954	NM_133220	Sgk3	5517
NM_013596	Mc5r	1162	NM_001163759	Dhx57	5094	NM_133222	Eltd1	4106
NM_001164214	Rerg	2197	NM_001163760	6430548M08Rik	5544	NM_001164621	Rnf14	3369
NM_001164222	Col4a3bp	5285	NM_001163761	6430548M08Rik	5535	NM_001164622	Rnf14	2904
NM_001164225	Fbxl16	3499	NM_001163766	Wdr90	6068	NM_013784	Pign	6777
NM_080461	Zfp358	1999	NM_199302	Lrsam1	4002	NM_207281	Mettl21e	3181
NM_080462	Hnmt	1653	NM_013469	Anxa11	2413	NM_013785	Ip6k1	4429
NM_080463	Pofut1	5618	NM_013473	Anxa8	1892	NM_133238	Cd209a	1484
NM_001164236	1700034J05Rik	2051	NM_199308	Mast3	5334	NM_001164639	Slk	6954
NM_001164242	Prpsap2	1771	NM_199309	Zdhhc19	1310	NM_001287224	Lrit3	2046
NM_001164243	Prpsap2	1814	NM_013475	Apoh	1202	NM_013791	Mkln1	3973
NM_001164244	Prpsap2	1718	NM_013415	Atp1b2	2959	NM_133247	Usp33	4209
NM_080471	Ankrd6	4573	NM_001163793	C530008M17Rik	6835	NM_133248	Glmn	2015
NM_013624	Otog	10043	NM_013477	Atp6v0d1	1617	NM_013797	Slco1a1	3967
NM_080558	Ssfa2	5109	NM_001163797	Zfp68	4725	NM_001164655	9530053A07Rik	7922
NM_080562	Ubox5	3758	NM_053266	Gtf2ird2	3468	NM_013809	Cyp2g1	1853
NM_080563	Rnf144a	5115	NM_199322	Dot1l	5694	NM_013810	Dbn1	2360
NM_001164272	Cpsf7	3577	NM_053268	Rasa2	5699	NM_013811	Dnah8	14583
NM_080637	Nme5	829	NM_001286757	Tiam2	3186	NM_001287817	Fmn1	9027
NM_080638	Mvp	2810	NM_199323	Tacc1	6581	NM_001164669	Dnah6	12652
NM_080639	Timp4	1416	NM_199366	Gal3st2	2846	NM_001287818	Fmn1	7767
NM_080640	Baalc	4177	NM_199446	Phkb	4640	NM_013814	Galnt1	3996
NM_080644	Cacng5	3678	NM_001163833	Msl3l2	2140	NM_013815	Baz1a	6179
NM_013648	Rtn2	1994	NM_053271	Rims2	7278	NM_001164676	Zfp229	4541
NM_001164311	Loxl4	5447	NM_053273	Ttyh2	3456	NM_207541	Zfp81	2253
NM_080708	Bmp2k	6585	NM_199465	Nexn	2580	NM_001288626	Arid3a	5355
NM_080727	Tmprss3	2738	NM_199466	Eml4	5125	NM_001288627	Emp1	2826
NM_013651	Sf3a2	1674	NM_001286786	Magi1	5727	NM_001164677	Pdcd6ip	5976
NM_001164316	Ccser1	5602	NM_001286788	Magi1	5099	NM_001288628	Emp1	2832
NM_001164325	Trpm7	7142	NM_054040	Tulp4	9714	NM_001164678	Pdcd6ip	5970
NM_080793	Setd7	7356	NM_013500	Hapln1	5055	NM_001164679	Ano8	3773
NM_080795	Lnx2	4543	NM_001163852	Tbc1d24	7946	NM_013829	Plcb4	3692
NM_013662	Sema6b	3847	NM_001163853	Tbc1d24	7774	NM_133488	Ntng1	5110
NM_013663	Srsf3	2585	NM_001163996	Zfp605	3158	NM_001164696	Klk6	1350
NM_080850	Pask	5119	NM_054046	Def8	3786	NM_001164697	Klk6	1346
NM_001164363	Nt5c2	4181	NM_054051	Pip4k2b	5068	NM_001164698	Klk6	1247
NM_001164365	Nt5c2	3838	NM_013513	Epb4.2	3472	NM_133643	Edaradd	8103
NM_013675	Sptb	10417	NM_054052	B3gnt5	4548	NM_013835	Trove2	8736
NM_130449	Colec12	3322	NM_013514	Dmtn	4129	NM_133653	Mat1a	3486
NM_001164376	Ctnna3	3681	NM_013515	Stom	2816	NM_001164717	Sh3pxd2a	10380
NM_130456	Nphs2	3108	NM_201355	Nat14	1612	NM_133659	Erg	2214
NM_001164411	Cnot4	5613	NM_001287056	Vegfa	3547	NM_133664	Lad1	2992
NM_144954	Ppil2	2230	NM_201357	Tssc1	1692	NM_013848	Ermap	4573
NM_001164433	Mical1	3344	NM_201360	Cyp2d12	1624	NM_207625	Acsl4	5280
NM_013700	Usp5	3176	NM_201363	Serpinb3c	1659	NM_013850	Abca7	6607
NM_001164441	Ankrd33b	7498	NM_054068	Vsx1	3579	NM_013852	Abcf3	3288
NM_013701	Ugt1a2	3206	NM_013529	Gfpt2	2986	NM_013854	Abcf1	3207
NM_207162	Gm20738	1209	NM_054070	Afg3l1	4288	NM_133674	Arhgef5	5420
NM_013705	Zfp30	2604	NM_013533	Gpr162	2676	NM_001164767	Robo3	4726
NM_207202	Ccdc120	3641	NM_013534	Leprel2	2779	NM_133676	Osgep	1608

NM 207209	Sec24b	4702	NM 054076	Optc	1906	NM 013861	Tpk1	2562
NM 001164483	Synj1	7085	NM 054078	Baz2a	8369	NM 001164772	Fbxw2	2532
NM 001164491	Ablim3	4226	NM 001164053	Pkig	1148	NM 001164785	Adamts20	6988
NM 001164493	Klhl29	7048	NM 001164055	Pkig	983	NM 001164786	Adamts20	1964
NM 207214	Exoc5	4659	NM 013546	Hebp1	1086	NM 133685	Rab31	3476
NM 130885	Oxr1	4240	NM 054087	Slc19a2	3571	NM 001164787	Sprr2a2	3547
NM 013724	Nrk	6604	NM 054088	Pnpla3	4649	NM 013865	Ndrg3	2644
NM 001164503	Akap11	9416	NM 054089	Tgs1	4389	NM 013867	Bcar3	3316
NM 013726	Dbf4	2444	NM 201407	Dennd4b	5399	NM 001164793	Polr2m	2189
NM 130903	Cd209c	1569	NM 001164078	Tia1	4450	NM 001164797	Hephl1	5261
NM 013734	Atp1a4	3485	NM 001164079	Tia1	4174	NM 013870	Smtn	3346
NM_001164524	Serpinb9c	1932	NM_201640	Cyp4a31	2509	NM_013873	Sult4a1	2446
NM 207232	Ptpdc1	4329	NM 201411	Flrt1	5876	NM 001164804	Coro2a	3887
NM_207234	Rexo4	2354	NM_001164117	Serpinb6a	1600	NM_013874	Dpf1	2278
NM 133187	Fam198b	6627	NM 201518	Flrt2	7099	NM 001164805	Thsd7a	11019
NM 001164531	Zfyve27	5498	NM 001164139	Spert	1615	NM 013876	Rnf11	2132
NM 013745	Nufip1	3974	NM 001164140	Spert	1534	NM 133700	Btbd10	2563
NM 001164559	Trmt1	2159	NM 054102	Ivns1abp	3513	NM 013879	Cabp1	1181
NM 001164560	Trmt1	2083	NM 001164141	Spert	1623	NM 013886	Hdgfrp3	5868
NM 013747	Golga5	2689	NM 057172	Fubp1	6491	NM 133710	Ctdspl	4627
NM 001164562	Sec22c	5676	NM 201600	Myo5b	6647	NM 207671	Zfp318	13926
NM 001164565	Acnat1	5048	NM 058212	Dpf3	1529	NM 133711	Spata4	1087
NM 013751	Hrasls	2915	NM 201609	Zfp652	2668	NM 013891	Spdef	1824
NM 001164569	Rffl	3650	NM 078477	Klf16	2652	NM 013893	Reg3d	782
NM 001164570	Rffl	3592	NM 001164167	Pias2	4942	NM 001165930	Glt8d1	1956
NM 001164571	Rffl	3425	NM 078478	Ghitm	2906	NM 207683	Pik3c2g	6358
NM 001164572	Snrk	4810	NM 001164168	Pias2	4984	NM 001165948	Pogz	7615
NM 001164574	Kbtbd3	2321	NM 001164169	Pias2	2073	NM 133734	Dcaf11	2515
NM 013758	Add3	4261	NM 001164170	Pias2	2089	NM 001165953	Trim45	4668
NM 001164578	Tsr2	3814	NM 080288	Elmo1	5576	NM 013913	Angptl3	1604
NM 207245	Zfp870	5321	NM 001164173	Cpsf1	4513	NM 133737	Lancl2	3657
NM 013761	Srr	3699	NM 201637	Chd8	8190	NM 001165954	Phc3	11051
NM 133207	Kcnh7	3798	NM 201639	Synm	7866	NM 001165955	Phc3	10967
NM 207246	Rasgrp3	4720	NM 201646	Btbd6	2075	NM 001165956	Phc3	10925
NM 001164581	Zfp961	2718	NM 080420	Lpo	2927	NM 013918	Usp25	4432
NM 133208	Zfp287	5021	NM 001164187	Nagk	1321	NM 013920	Hnf4g	4180
NM 001164583	Dnajc6	5220	NM 013570	Krt33b	1596	NM 207708	Syngr1	4192
NM 001164584	Dnajc6	5165	NM 203507	Rwdd4a	2971	NM 013922	Zfp354c	5367
NM 001164585	Dnajc6	5230	NM 080442	Tssk3	1034	NM 013923	Rnf19a	4276
NM 013766	Prl3c1	835	NM 001164198	Prkacb	4337	NM 013924	Abt1	4456
NM 133213	Xpnpep2	3498	NM 080443	Asb7	4893	NM 211138	Pcyt1b	4875
NM 133214	Smco4	954	NM 001164199	Prkacb	4210	NM 212433	Fbxo3	4659
NM 001164597	Znf512b	5390	NM 001164200	Prkacb	4302	NM 133755	Tubgcp2	3085
NM 133218	Zfp704	13849	NM 001164203	Plec	14959	NM 133756	Gpn1	1847
NM 001164606	Ccdc116	3926	NM 080448	Srgap3	8887	NM 212438	Wiz	3903
NM 133220	Sgk3	5517	NM 080451	Synpo2	7116	NM 133758	Usp47	5540
NM 001164609	Smpd4	4584	NM 001164210	Sptssb	1784	NM 015729	Acox1	3992
NM 001164610	Smpd4	4545	NM 001164220	Trim13	1640	NM 212444	Gyk	4427
NM 133222	Eltd1	4106	NM 080458	Bsnd	2716	NM 133762	Ncapg2	6604
NM 001164611	Smpd4	4542	NM 080463	Pofut1	5618	NM 212449	AU019823	3335
NM 207270	Ptprh	4020	NM 080464	Ppp1r3a	7120	NM 133764	Atp6v0e2	1849
NM 001164612	Atp13a4	4114	NM 080467	Atp6v0a4	3300	NM 133765	Fbxo31	4372
NM 001164614	Ccdc159	1545	NM 001164237	Rnf41	4394	NM 015734	Col5a1	8420
NM 133228	Zfp87	2710	NM 080471	Ankrd6	4573	NM 212486	Gimap8	3650
NM 013782	Ptdss2	2172	NM 013624	Otog	10043	NM 133776	Gpr110	3504
NM 001287206	BC030499	3245	NM 080558	Ssfa2	5109	NM 133787	Nmd3	1739
NM 013784	Pign	6777	NM 001164255	Tpm1	1577	NM 001165968	Pglyrp4	2229
NM 013785	Ip6k1	4429	NM 080559	Sh3bgrl3	721	NM 015764	Greb1	8274
NM 133236	Glcci1	6201	NM 001164256	Tpm1	1550	NM 015765	Hspa14	1791
NM 013786	Hsd17b6	1479	NM 207031	Ano7	4019	NM 001001178	Ccdc148	3992

NM 001164639	Slk	6954	NM 001164263	Plekhs1	2385	NM 015768	Prok2	1513
NM 001287224	Lrit3	2046	NM 080562	Ubox5	3758	NM 133815	Lbr	3568
NM 207522	Mettl25	2039	NM 080563	Rnf144a	5115	NM 001001183	Tmem204	1865
NM 133247	Usp33	4209	NM 013632	Pnp	2941	NM 015773	Spag6	2483
NM_001164658	Catsperg1	3566	NM_013635	Sypl	4587	NM_015774	Ero1l	4435
NM 013811	Dnah8	14583	NM 080634	Hps3	4011	NM 133818	AI597479	3476
NM 001164671	Dnaja1	5632	NM 080636	Hars2	3075	NM 015776	Mfap5	1413
NM 001164672	Dnaja1	5489	NM 001164289	Phf11c	1590	NM 213727	Amer3	4367
NM 001164676	Zfp229	4541	NM 001164306	Gm4846	1744	NM 001001144	Scap	4226
NM 207541	Zfp81	2253	NM 001164312	Gm4847	3012	NM 001001321	Slc35d2	2276
NM 001164677	Pdcd6ip	5976	NM 001164320	Chadl	2547	NM 133833	Dst	17210
NM 001164678	Pdcd6ip	5970	NM 080788	Ttbk2	10906	NM 015791	Fbxo8	3527
NM 001164679	Ano8	3773	NM 001164325	Trpm7	7142	NM 133837	Cdc123	1623
NM 001164682	Mpp4	2992	NM 001164327	Phf11b	1163	NM 001166000	Lingo2	4101
NM 001164689	Gm6710	3788	NM 001164329	Gm6904	898	NM 133847	Tm9sf4	3895
NM 001164696	Klk6	1350	NM 013661	Sema5b	4556	NM 015794	Fbxl17	17001
NM 001164697	Klk6	1346	NM 001164352	Efemp2	1802	NM 133853	Magi3	6672
NM 001164698	Klk6	1247	NM 001164355	Ska1	2605	NM 001001186	Zfp456	4079
NM 133643	Edaradd	8103	NM 013663	Srsf3	2585	NM 133871	Ifi44	2916
NM 013835	Trove2	8736	NM 013666	St8sia5	2114	NM 015802	Dlc1	6184
NM 001164709	Syvn1	3103	NM 080850	Pask	5119	NM 133880	Pafah2	3183
NM 001164717	Sh3pxd2a	10380	NM 001164363	Nt5c2	4181	NM 133882	C8b	2239
NM 001164725	Fcrl6	1914	NM 001164365	Nt5c2	3838	NM 001166064	Syde2	5495
NM 013844	Zfp68	4731	NM 080855	Zcchc14	6476	NM 015804	Atp11a	7443
NM_001164727	Gm10406	1955	NM_001164367	Rab11fip2	5373	NM_001166065	Gcnt4	5107
NM 207583	Brinp2	4117	NM 080858	Asb12	1260	NM 001166066	Mroh2b	5232
NM 013848	Ermap	4573	NM 013675	Sptb	10417	NM 015805	Atp9b	5444
NM 013852	Abcf3	3288	NM 130448	Pcdh18	5168	NM 015806	Mapk6	4205
NM 133672	Vps26a	3031	NM 013678	Surf2	1170	NM 001166067	Slc4a5	5141
NM 013853	Abcf2	2573	NM 130454	Recql5	4001	NM 133895	Slc15a4	2720
NM 133673	Tor1b	2997	NM 001164401	Ptpro	6550	NM 015811	Rgs1	1343
NM 207652	Tsc22d1	4588	NM 001164402	Ptpro	4180	NM 001166206	Erv3	3378
NM 133681	Tspan1	1971	NM 130456	Nphs2	3108	NM 133897	Lrrc8c	2829
NM 133683	Tmem19	3010	NM 001164403	Ptpro	4096	NM 001166207	Dpy19l2	3441
NM_207657	5031410I06Rik	2947	NM_130457	Cntnap4	4863	NM_001001327	Vkorc1l1	4825
NM 133686	Qprt	1211	NM 001164412	Cnot4	3335	NM 001001804	Ephx4	1262
NM 001164793	Polr2m	2189	NM 130796	Snx18	4466	NM 133906	Zkscan1	8261
NM 001164797	Hephl1	5261	NM 144954	Ppil2	2230	NM 133907	Ube3c	5033
NM 133693	Rbm42	1701	NM 001164413	Cnot4	3548	NM 015822	Fbxl3	4265
NM 001164805	Thsd7a	11019	NM 001164414	Cnot4	3344	NM 133910	Tbc1d14	4602
NM 013876	Rnf11	2132	NM 001164426	Kcnk13	3075	NM 133919	Aff1	8323
NM 207659	Hook3	12836	NM 130859	Card10	4748	NM 001166365	Fam122b	4331
NM 133700	Btbd10	2563	NM 013694	Tnp2	724	NM 133921	Nfxl1	3671
NM 013886	Hdgfrp3	5868	NM 001164427	Kcnk13	3112	NM 001001450	Ssxb2	957
NM 133710	Ctdspl	4627	NM 001164441	Ankrd33b	7498	NM 015828	Gne	5384
NM 207671	Zfp318	13926	NM 001164472	Nip7	2895	NM 133925	Rbm28	4201
NM 001165253	Ctage5	2832	NM 207204	Ninl	5104	NM 016661	Ahcy	2560
NM 001165254	Ctage5	2831	NM 001164480	Sipa1	3614	NM 001001489	BC021785	2372
NM 207678	Ccnl2	2442	NM 001164482	Sipa1	3553	NM 001166377	Armcx1	2491
NM 001165929	Ccdc78	1651	NM 001164481	Sipa1	3868	NM 001166378	Armcx1	2190
NM 207683	Pik3c2g	6358	NM 130878	Cdhr1	4364	NM 001166379	Armcx1	2306
NM 133733	Clmp	2929	NM 013718	Trappc3	1315	NM 133941	Dhx32	2962
NM 001165953	Trim45	4668	NM 001164491	Ablim3	4226	NM 001166380	Armcx1	2353
NM 133737	Lancl2	3657	NM 207214	Exoc5	4659	NM 133942	Plekha1	3321
NM 001165954	Phc3	11051	NM 130886	Card14	3958	NM 016706	Coil	2609
NM 013914	Snai3	1589	NM 207217	Itfg3	2751	NM 133953	Sf3b3	4299
NM 001165955	Phc3	10967	NM 001164518	Iglon5	2567	NM 001001798	Atp11c	5965
NM 001165956	Phc3	10925	NM 013734	Atp1a4	3485	NM 133955	Rhou	3367
NM 013918	Usp25	4432	NM 133185	Rogdi	1392	NM 001166397	Armcx2	3683

NM 207708	Syngr1	4192	NM 013741	Smok2a	1787	NM 001166398	Armcx2	3626
NM 013921	Prss30	1469	NM 001164532	Cpsf4l	1390	NM 133969	Cyp4v3	2930
NM 133741	Snrk	4860	NM 013745	Nufip1	3974	NM 133973	Cog4	2754
NM 013931	Mapk8ip3	5596	NM 133193	Il1rl2	1950	NM 001166413	Arhgef25	2199
NM_133755	Tubgcp2	3085	NM_013748	Clnk	1586	NM_001001881	2510009E07Rik	5164
NM 133756	Gpn1	1847	NM 001164565	Acnat1	5048	NM 001166416	Med23	4946
NM 013933	Vapa	1658	NM 001164568	Sipa1	3583	NM 001001883	Hecw2	11179
NM 015728	Slc33a1	3122	NM 133199	Scn4a	6598	NM 001166453	Pls3	3137
NM 212438	Wiz	3903	NM 013753	Pald1	4274	NM 001001980	Limch1	6142
NM 212444	Gyk	4427	NM 001164574	Kbtbd3	2321	NM 001166454	Pls3	3132
NM 212485	Krt73	2154	NM 001164578	Tsr2	3814	NM 001001981	Utp14b	3695
NM 133774	Stard4	2985	NM 207246	Rasgrp3	4720	NM 133994	Gstt3	1873
NM 015742	Myo9b	7100	NM 001164581	Zfp961	2718	NM 001166457	Brcc3	4338
NM 212487	Krt78	3434	NM 133208	Zfp287	5021	NM 133998	Fam207a	2444
NM_001002005	Panx2	3434	NM_207248	4930433I11Rik	2022	NM_001001984	Kdm2a	7268
NM 133777	Ube2s	988	NM 133210	Sertad3	1356	NM 001001986	Nol4l	5631
NM 133781	Cab39	3800	NM 207260	Ankrd34c	2475	NM 001166463	Spock1	4665
NM 133783	Ptges2	1969	NM 133213	Xpnpep2	3498	NM 001166464	Spock1	3140
NM 133787	Nmd3	1739	NM 013769	Tjp3	3043	NM 001166465	Spock1	3131
NM 015756	Shroom3	7210	NM 133215	Mtmr4	5710	NM 016677	Hpcal1	1561
NM 015767	Ttpa	3053	NM 133220	Sgk3	5517	NM 001166466	Spock1	4671
NM 001001178	Ccdc148	3992	NM 001164609	Smpd4	4584	NM 001166493	Rasgrp3	4728
NM 001001180	Zfp941	3909	NM 001164610	Smpd4	4545	NM 134040	Ddx1	2488
NM 015772	Sall2	5058	NM 133222	Eltd1	4106	NM 134044	Vipas39	2509
NM 001001183	Tmem204	1865	NM 001164611	Smpd4	4542	NM 016703	Preb	5505
NM 015774	Ero1l	4435	NM 001164612	Atp13a4	4114	NM 134054	Sptssa	1363
NM 133826	Atp6v1h	1976	NM 001164613	Atp13a4	2903	NM 134062	Dapk1	5304
NM 133829	Mfsd6	4770	NM 001164614	Ccdc159	1545	NM 001002897	Atg9b	3902
NM 015785	Zpbp	3845	NM 133225	Acbd3	3487	NM 016690	Hnrnpdl	2761
NM 133833	Dst	17210	NM 013777	Akr1c12	1231	NM 001166501	Dennd1b	8206
NM 133834	Hnrnpf	2179	NM 013778	Akr1c13	1232	NM 134071	Ankrd32	3742
NM 001165999	Lingo2	4642	NM 133227	Nup155	6420	NM 134073	Kctd9	3174
NM 133837	Cdc123	1623	NM 133229	Ripply3	1554	NM 001003717	Osbpl8	7115
NM 015792	Fbxo18	3539	NM 013783	Mmel1	2948	NM 001003719	Ralgapa1	8249
NM 001166000	Lingo2	4101	NM 013784	Pign	6777	NM 134079	Adk	1795
NM 001166001	Lingo2	3562	NM 207281	Mettl21e	3181	NM 001003893	Masp2	3061
NM 001001130	Zfp85	2218	NM 013785	Ip6k1	4429	NM 016787	Bnip2	5806
NM 001001333	Hexdc	2164	NM 133236	Glcci1	6201	NM 001166531	Sfmbt1	7743
NM 015794	Fbxl17	17001	NM 001164640	Apol7a	2292	NM 001003911	Adamts7	5424
NM 015796	Fbxo17	1984	NM 013790	Abcc5	5826	NM 001003915	Slc5a12	5104
NM 133853	Magi3	6672	NM 013792	Naglu	2504	NM 134099	Fbxo4	3479
NM 015797	Fbxo6	1955	NM 133247	Usp33	4209	NM 134111	Eaf2	1903
NM 133857	Usp53	4014	NM 001164641	Trappc9	4688	NM 001003934	Rtn3	5038
NM 001001152	Zfp458	3488	NM 001164642	Trappc9	3127	NM 001003933	Rtn3	4981
NM 133865	Dclre1b	4309	NM 013799	Ate1	4768	NM 134112	Kctd1	1772
NM 001001186	Zfp456	4079	NM 013800	Barx2	1791	NM 134115	Stk38	3288
NM 015801	Pnpla6	4455	NM 133255	Hook2	2617	NM 016762	Matn2	3520
NM 001166030	Mylk4	5641	NM 013809	Cyp2g1	1853	NM 134127	Cyp4f15	2157
NM 133871	Ifi44	2916	NM 133348	Acot7	1494	NM 134136	Fbxo38	4416
NM 015802	Dlc1	6184	NM 133350	Mapre3	1921	NM 016809	Rbm3	2900
NM 133880	Pafah2	3183	NM 013811	Dnah8	14583	NM 001004025	Ppp3r2	2985
NM 133881	Tmem54	1126	NM 001287817	Fmn1	9027	NM 001004066	Zfp386	4680
NM 001166064	Syde2	5495	NM 133352	Tm9sf3	6144	NM 134144	Cyp2c50	1834
NM 001166063	Ccdc152	798	NM 001164669	Dnah6	12652	NM 001004139	Zfp619	5978
NM 001166066	Mroh2b	5232	NM 001287818	Fmn1	7767	NM 001004364	Asap2	5557
NM 015806	Mapk6	4205	NM 001164671	Dnaja1	5632	NM 134163	Mbnl3	8671
NM 001166138	Armc8	3629	NM 013814	Galnt1	3996	NM 001004154	Rragb	2308
NM 133887	Stx12	2320	NM 001164672	Dnaja1	5489	NM 016739	Caprin1	6181
NM 133891	Slc44a1	3102	NM 013818	Gtpbp1	3444	NM 016911	Srpx	2456
NM 133895	Slc15a4	2720	NM 001164682	Mpp4	2992	NM 001004176	Maml3	6514

NM 001001187	Zfp738	4523	NM 013832	Rasal1	3235	NM 016748	Ctps	2726
NM 015814	Dkk3	3357	NM 133641	Rtkn	3113	NM 001004184	Slc28a1	2846
NM 133897	Lrrc8c	2829	NM 133643	Edaradd	8103	NM 001004185	Whamm	3069
NM_001166207	Dpy19l2	3441	NM_013835	Trove2	8736	NM_001004187	2610020H08Rik	1823
NM 001001327	Vkorc1l1	4825	NM 001164717	Sh3pxd2a	10380	NM 016751	Clec4f	2400
NM 133903	Spon2	2237	NM 001164725	Fcrl6	1914	NM 001004357	Cntnap2	7002
NM 001166218	Zfp712	3970	NM 133657	Cyp2a12	1675	NM 016813	Nxf1	2312
NM 001001334	BC061194	1651	NM 013844	Zfp68	4731	NM 016770	Folh1	3047
NM 015821	Fbxl8	1901	NM 013845	Ror1	3542	NM 016782	Cntnap1	5360
NM 015822	Fbxl3	4265	NM 013848	Ermap	4573	NM 016786	Ube2k	4866
NM 133917	Mlxip	7321	NM 207625	Acsl4	5280	NM 001004367	Cxxc4	5619
NM 133919	Aff1	8323	NM 013850	Abca7	6607	NM 001004468	Tacc2	9103
NM 133921	Nfxl1	3671	NM 013852	Abcf3	3288	NM 001004761	Gpr158	7143
NM 015827	Copb2	3043	NM 133672	Vps26a	3031	NM 001004762	Pla2g4c	3374
NM 133924	Snx21	1520	NM 133673	Tor1b	2997	NM 016792	Txnl1	2558
NM 001166371	Zfml	4410	NM 207649	Rcan2	3389	NM 001005371	Gm13084	1814
NM 015830	Capn15	3288	NM 133675	Sptssb	1753	NM 017369	Gabre	2791
NM 133925	Rbm28	4201	NM 001164767	Robo3	4726	NM 001128094	Atp13a3	7220
NM 133931	Pot1a	3153	NM 133676	Osgep	1608	NM 001005420	Ppp1r26	5485
NM 001001492	Lca5l	3170	NM 013860	Limd1	4864	NM 001005423	Mreg	2493
NM 001166377	Armcx1	2491	NM 133679	Cryzl1	1809	NM 016854	Ppp1r3c	2696
NM 001166378	Armcx1	2190	NM 001164768	Fbxw2	2846	NM 016859	Bysl	3854
NM 001166379	Armcx1	2306	NM 133680	Hiatl1	3472	NM 016868	Hif3a	6947
NM 001166380	Armcx1	2353	NM 001164769	Fbxw2	2294	NM 001005605	Aebp2	6192
NM 133945	Vrk3	1790	NM 001164770	Fbxw2	2813	NM 001005846	Mcoln2	2496
NM 001166384	Rbmy	1580	NM 001164785	Adamts20	6988	NM 001005854	Gm609	2122
NM 001001792	Zfp239	2828	NM 001164787	Sprr2a2	3547	NM 016872	Vamp5	1648
NM 133951	Rrp8	2890	NM 013865	Ndrg3	2644	NM 016877	Cnot4	5622
NM 001166391	F13a1	3854	NM 133688	Lyrm5	1386	NM 001007461	Gsdma3	1477
NM 133953	Sf3b3	4299	NM 013867	Bcar3	3316	NM 001005860	Clec4a4	1107
NM 001001798	Atp11c	5965	NM 001164797	Hephl1	5261	NM 134249	Timd2	3035
NM 133954	Usb1	1764	NM 013870	Smtn	3346	NM 001007077	Gm13023	1838
NM 133966	Taf5l	2893	NM 013875	Pde7b	4127	NM 134256	Slc22a27	2165
NM 133969	Cyp4v3	2930	NM 013876	Rnf11	2132	NM 017383	Cntn6	3613
NM 001166413	Arhgef25	2199	NM 001164806	Bend4	7935	NM 017391	Slc5a3	10918
NM 001166416	Med23	4946	NM 207659	Hook3	12836	NM 016912	Cdkl2	4079
NM 016708	Npy5r	2314	NM 013878	Cabp2	934	NM 134437	Il17rd	8220
NM 001001883	Hecw2	11179	NM 207663	Synm	6960	NM 134448	Dst	23252
NM 001166427	Hnrnpf	2297	NM 133705	Pycr2	1615	NM 017395	Rfx5	4189
NM 001166428	Hnrnpf	2236	NM 013886	Hdgfrp3	5868	NM 017396	Cyp3a41a	2060
NM 133979	Ano10	2653	NM 207671	Zfp318	13926	NM 017397	Ddx20	2710
NM 001166429	Hnrnpf	2197	NM 013890	Fbxw2	2905	NM 138310	Apobr	3615
NM 001166430	Hnrnpf	2548	NM 133717	Rab43	4311	NM 138313	Bmf	4654
NM 001166431	Hnrnpf	2487	NM 207678	Ccnl2	2442	NM 001007576	Gucy2f	9998
NM 001166432	Hnrnpf	2288	NM 013900	Mfi2	4133	NM 016843	Atxn10	2425
NM 001166453	Pls3	3137	NM 133722	Abhd17c	2252	NM 138584	Spg21	2690
NM 001001980	Limch1	6142	NM 013901	Slc39a1	2344	NM 016851	Irf6	4140
NM 001166454	Pls3	3132	NM 013906	Adamts8	3632	NM 001009546	Naaladl1	2238
NM 001166456	Slc38a1	7059	NM 207683	Pik3c2g	6358	NM 138594	D6Wsu163e	2238
NM 016684	Zscan12	4435	NM 013908	Fbxw5	2359	NM 001009547	Adam26b	2405
NM_001166458	Slc38a1	7266	NM_133732	4931406C07Rik	2616	NM_019396	Cyhr1	4121
NM 001001984	Kdm2a	7268	NM 001165948	Pogz	7615	NM 138628	Txlnb	4457
NM 133999	Fig4	3278	NM 001165949	Pias3	2938	NM 001007584	Gm11541	1681
NM 001001985	Nat8l	6529	NM 133734	Dcaf11	2515	NM 017479	Kat6b	7347
NM 001001986	Nol4l	5631	NM 013925	Adat1	3159	NM 017475	Rragc	2621
NM 001166463	Spock1	4665	NM 013929	Siva1	782	NM 019401	Nmi	1263
NM 001166464	Spock1	3140	NM 013931	Mapk8ip3	5596	NM 017469	Gucy1b3	3251
NM 001003666	Zfp457	2117	NM 013932	Ddx25	1675	NM 017466	Ccrl2	2110
NM 001166465	Spock1	3131	NM 133758	Usp47	5540	NM 001008238	Bnip2	5770
NM 001166466	Spock1	4671	NM 133759	Zbtb3	1917	NM 001008420	Cdh12	5666

NM 001002008	Zfp948	2600	NM 133762	Ncapg2	6604	NM 138661	Pcdha9	5341
NM 016711	Tmod2	9893	NM 015731	Atp9a	3529	NM 018818	Chm	4867
NM 001166493	Rasgrp3	4728	NM 015734	Col5a1	8420	NM 138662	Pcdha3	5230
NM 134044	Vipas39	2509	NM 212452	Rxfp1	2277	NM 138663	Pcdha12	5254
NM 134046	Cenpo	4343	NM 133766	Efr3a	5210	NM 138664	Dnajc28	3365
NM 134058	Pelo	1588	NM 212473	Fam53b	5211	NM 018738	Igtp	2064
NM 134060	Slc35b3	1929	NM 212483	Krt42	1547	NM 001008506	Zc3h14	3163
NM 134062	Dapk1	5304	NM 212485	Krt73	2154	NM 138675	Med9	3013
NM 134064	Rnf44	3941	NM 213730	Krt39	1449	NM 138674	Pkhd1l1	12750
NM 001166501	Dennd1b	8206	NM 015742	Myo9b	7100	NM 018732	Scn3a	9560
NM 134071	Ankrd32	3742	NM 015745	Rbp3	5276	NM 019414	Selenbp2	1695
NM 134072	Akr1c14	2459	NM 133791	Wwc2	5042	NM 138679	Ash1l	11293
NM 134073	Kctd9	3174	NM 133795	Ttc1	1469	NM 001009575	Armcx5	2835
NM 134074	Dock9	7760	NM 015764	Greb1	8274	NM 138684	Wfdc12	800
NM 001003717	Osbpl8	7115	NM 015765	Hspa14	1791	NM 019421	Cd320	2209
NM 134077	Rbm26	4105	NM 015767	Ttpa	3053	NM 001009950	Slc38a8	2648
NM 001003893	Masp2	3061	NM 001001180	Zfp941	3909	NM 001166581	BC005561	5181
NM 016787	Bnip2	5806	NM 133810	Stk17b	3298	NM 001009981	Slc10a7	3560
NM 134086	Slc38a1	7752	NM 015774	Ero1l	4435	NM 001010825	Ficd	3192
NM 001166531	Sfmbt1	7743	NM 015776	Mfap5	1413	NM 018773	Skap2	1656
NM 134095	Desi1	2945	NM 213727	Amer3	4367	NM 138755	Phf21a	6346
NM 134097	Topors	3842	NM 213733	Npepl1	2110	NM 138756	Slc25a36	3061
NM_001166556	Abca6	1523	NM_133825	D1Ertd622e	2738	NM_018759	Zfp326	2672
NM 001166557	Abca6	1441	NM 001165997	Spopl	2858	NM 019429	Prss16	2152
NM 134115	Stk38	3288	NM 001165998	Spopl	2722	NM 018745	Azin1	4868
NM 016762	Matn2	3520	NM 133833	Dst	17210	NM 019430	Cacng3	1540
NM 134123	Scaf8	4892	NM 133834	Hnrnpf	2179	NM 018817	Smarcal1	6715
NM 134125	Trip10	2290	NM 133835	Ubac1	3450	NM 001166625	Ccr9	4392
NM_001003955	Rab11fip5	6156	NM_015791	Fbxo8	3527	NM_019437	Rfk	2482
NM 001003971	Senp7	4802	NM 133837	Cdc123	1623	NM 001166629	Dynlt1a	4978
NM 134136	Fbxo38	4416	NM 001166000	Lingo2	4101	NM 019438	Ncapg	3698
NM 016809	Rbm3	2900	NM 133838	Ehd4	3387	NM 019440	Irgm2	3537
NM 001004025	Ppp3r2	2985	NM 001001333	Hexdc	2164	NM 001166635	Midlip1	2059
NM 001004066	Zfp386	4680	NM 015794	Fbxl17	17001	NM 139134	Chodl	2530
NM 001004361	Tpgs2	4040	NM 015801	Pnpla6	4455	NM 139138	Emr4	3491
NM 001004139	Zfp619	5978	NM 001166033	Gtf3c4	7127	NM 139140	Spats2	2075
NM 001004141	Cyp2j11	1960	NM 133880	Pafah2	3183	NM 001166645	Zfp882	5651
NM 134150	Otub1	1677	NM 015803	Atp8a2	3685	NM 139145	Hlcs	4833
NM 134154	Slc25a45	1833	NM 001166066	Mroh2b	5232	NM 001166648	Zfp280c	4035
NM 001004148	Slc13a5	3285	NM 015805	Atp9b	5444	NM 001166649	Zfp280c	4056
NM 134160	Mcoln3	1712	NM 133886	AU040320	4384	NM 001166650	Zfp280c	3875
NM 134163	Mbnl3	8671	NM 001166067	Slc4a5	5141	NM 139270	Pth2r	2414
NM 134164	Syt12	3277	NM 133891	Slc44a1	3102	NM 019451	Il1f5	1759
NM_001004155	9930012K11Rik	2004	NM_001166173	Dmkn	2070	NM_018813	Cpsf3	2560
NM 001004173	Sgpp2	3998	NM 133893	Oas1d	1750	NM 001166653	Cdk13	2749
NM 016739	Caprin1	6181	NM 001166174	Dmkn	1977	NM 018803	Syt10	1845
NM 016911	Srpx	2456	NM 015814	Dkk3	3357	NM 139200	Cytip	5735
NM 001004177	Celsr2	10401	NM 001001326	St5	4255	NM 018804	Syt11	4585
NM 016759	Rundc3a	2430	NM 015816	Lsm4	953	NM 144731	Galnt7	4363
NM_001004184	Slc28a1	2846	NM_133900	Psph	1554	NM_001011531	Olfr329-ps	1300
NM_001004187	2610020H08Rik	1823	NM_133904	Acacb	8497	NM_018814	Pcnx	12139
NM 001004190	Zfp560	4732	NM 001166218	Zfp712	3970	NM 139229	Cog8	2134
NM 016736	Nub1	3455	NM 133906	Zkscan1	8261	NM 018779	Pde3a	4205
NM 016745	Atp2a3	4593	NM 133910	Tbc1d14	4602	NM 139232	Fgd4	3229
NM 001004357	Cntnap2	7002	NM 001001444	Defb29	405	NM 018786	Prpf40b	3182
NM 001004363	Nuak1	5032	NM 133914	Rasa4	2933	NM 139269	Pla2g16	3523

NM_016770	Folh1	3047	NM_001166360	6030458C11Rik	1987	NM_019458	Paf1	1953
NM 001004366	Scube3	2982	NM 133917	Mlxip	7321	NM 019460	Sfmbt1	7694
NM 001004436	Wapal	4585	NM 001166364	Fam219b	3055	NM 139296	Moxd2	1860
NM 016796	Vamp4	2532	NM 133918	Emilin1	3466	NM 018825	Sh2b2	2848
NM 134189	Galnt10	4725	NM 015825	Sh3bgr	1321	NM 139303	Kif18a	3487
NM 001004761	Gpr158	7143	NM 001166365	Fam122b	4331	NM 018805	Hs3st3b1	6004
NM 001004762	Pla2g4c	3374	NM 015828	Gne	5384	NM 018807	Plagl2	5407
NM 016876	Eif3g	1068	NM 133925	Rbm28	4201	NM 139307	Vasn	3189
NM 016803	Chst3	6129	NM 016661	Ahcy	2560	NM 018797	Plxnc1	7059
NM 001005370	Spin2c	1323	NM 133931	Pot1a	3153	NM 139309	Fktn	3382
NM 016900	Cav2	2748	NM 001001489	BC021785	2372	NM 018769	Dfna5	2133
NM 017367	Ccni	2830	NM 001001492	Lca5l	3170	NM 139310	Otoa	3673
NM 016878	Dnpep	1754	NM 001001495	Tnip3	3769	NM 144491	Dph1	2103
NM 017370	Hp	1365	NM 133942	Plekha1	3321	NM 144511	Ces3b	2065
NM 001005420	Ppp1r26	5485	NM 001001565	Chpf	3250	NM 144524	Angel1	3867
NM 001005425	Zfp663	3337	NM 016706	Coil	2609	NM 144525	Tmem214	2762
NM 001005506	Txlna	4869	NM 016669	Crym	1256	NM 018829	Ap3m1	4424
NM 001005507	Smg7	5855	NM 001001735	Whsc1l1	3747	NM 144535	Ap5m1	3165
NM 001005508	Arhgap30	4509	NM 133951	Rrp8	2890	NM 018874	Pnliprp1	1619
NM 016856	Cpsf2	5315	NM 133952	Unc45a	3285	NM 144539	Slamf7	3977
NM 016854	Ppp1r3c	2696	NM 001001798	Atp11c	5965	NM 144548	Il23r	2488
NM_016859	Bysl	3854	NM_001166394	4931428F04Rik	3174	NM_144556	Lgi4	3444
NM 016868	Hif3a	6947	NM 001166397	Armcx2	3683	NM 019477	Acsl4	4990
NM 001005605	Aebp2	6192	NM 001166398	Armcx2	3626	NM 144785	Slc22a19	1984
NM 016909	Tsnax	2391	NM 133962	Arhgef18	5300	NM 144786	Ggt7	2611
NM 016862	Vti1a	3405	NM 001166408	Rai14	4843	NM 019481	Slc13a1	2495
NM_001005858	I830012016Rik	2013	NM_016662	Mxd3	1299	NM_018861	Slc1a4	3926
NM 016877	Cnot4	5622	NM 016708	Npy5r	2314	NM 144788	Hectd1	8988
NM 017379	Tuba8	1432	NM 001166427	Hnrnpf	2297	NM 018864	Impa1	2601
NM 016924	Rwdd2b	2104	NM 001166428	Hnrnpf	2236	NM 018878	Paxip1	5864
NM 134256	Slc22a27	2165	NM 001166429	Hnrnpf	2197	NM 144792	Sgms1	3624
NM 017390	Svs2	1570	NM 001166430	Hnrnpf	2548	NM 001167679	Tbc1d31	3503
NM 017391	Slc5a3	10918	NM 001166431	Hnrnpf	2487	NM 018865	Wisp1	5022
NM 017392	Celsr2	10543	NM 001166432	Hnrnpf	2288	NM 001011800	Olfr212	2672
NM 016912	Cdkl2	4079	NM 001166453	Pls3	3137	NM 018881	Fmo2	3336
NM 134437	Il17rd	8220	NM 001001980	Limch1	6142	NM 144803	Chrna2	3527
NM 016918	Nudt5	1590	NM 001166454	Pls3	3132	NM 001167730	Rad18	2701
NM 134448	Dst	23252	NM 016683	Zkscan5	3829	NM 144807	Chpt1	3239
NM 017395	Rfx5	4189	NM 001001981	Utp14b	3695	NM 144808	Slc39a14	4927
NM 017396	Cyp3a41a	2060	NM 133995	Upb1	3042	NM 018879	Nprl2	1473
NM 001007465	Rffl	3506	NM 133998	Fam207a	2444	NM 001167746	Dnah17	14889
NM 138312	Fam172a	4036	NM 016716	Cul3	2764	NM 144812	Tnrc6b	17334
NM 016860	Actr1a	2742	NM 001166463	Spock1	4665	NM 019490	Uso1	3914
NM 138315	Mical1	3563	NM 001166466	Spock1	4671	NM 144818	Ncaph	2702
NM 001007576	Gucy2f	9998	NM 134015	Fbxw11	4339	NM 001167777	Asxl3	11697
NM 138583	Tango2	1529	NM 001002012	Hspa2	2550	NM 144825	Taok1	12411
NM 016851	Irf6	4140	NM 016712	Tmod4	1241	NM 144834	Serpina10	1570
NM_138589	Ubfd1	4831	NM_134022	6330403K07Rik	1626	NM_144835	Heatr1	6776
NM 001007578	Armcx6	2034	NM 016711	Tmod2	9893	NM 019535	Sh3gl2	2817
NM 001007580	Fndc3c1	4830	NM 001166493	Rasgrp3	4728	NM 001167828	Trim30d	3294
NM 017463	Pbx2	2801	NM 134040	Ddx1	2488	NM 019516	Lgals12	2755
NM_138594	D6Wsu163e	2238	NM_134054	Sptssa	1363	NM_144842	Zmym5	5351
NM 138598	Fam104a	2635	NM 134062	Dapk1	5304	NM 144845	Ugt3a2	2196
NM 017473	Rdh7	1598	NM 001002898	Sirpb1a	1232	NM 001167860	Wipf3	4204
NM 001007583	Best3	2719	NM 134074	Dock9	7760	NM 001167861	Wipf3	4102
NM 019396	Cyhr1	4121	NM 001166506	Sec14l1	4736	NM 019539	Cts7	1493
NM 017477	Copg1	4014	NM 134082	Farp1	4857	NM 019540	Pfpl	3918

NM 001009548	Adam20	2589	NM 134083	Rcbtb2	3406	NM 001167875	Cyp2c50	1657
NM 001007584	Gm11541	1681	NM 001166507	Sec14l1	4380	NM 001167877	Cyp2c50	1891
NM 001007596	Rtn1	1551	NM 016920	Atp6v0a1	4019	NM 144857	Rrp36	1212
NM 017469	Gucy1b3	3251	NM 001166531	Sfmbt1	7743	NM 144861	Rprd1a	4297
NM 001008232	Asap3	5582	NM 016742	Cdc37	2456	NM 144863	Wdr36	2161
NM 001008238	Bnip2	5770	NM 134091	Sgsm3	3013	NM 001167883	Ankrd50	4915
NM 001008421	Nol10	3004	NM 001003919	Ddx11	4167	NM 144866	Etf1	3718
NM 138664	Dnajc28	3365	NM 001003939	BC030307	1865	NM 144868	Pcnxl3	7281
NM 001008506	Zc3h14	3163	NM 001003947	Cyp4x1	1524	NM 019549	Plek	4117
NM 138672	Stab1	7995	NM 134115	Stk38	3288	NM 144880	Ppp2r5a	3082
NM 138675	Med9	3013	NM 134125	Trip10	2290	NM 019574	Patz1	3100
NM 018732	Scn3a	9560	NM 016744	Pde1a	4309	NM 019565	Zfp386	4575
NM 138679	Ash1l	11293	NM 134135	Slc39a3	3479	NM 144893	Slc35c2	2009
NM 001009575	Armcx5	2835	NM 001003971	Senp7	4802	NM 001167939	Mau2	5365
NM 018764	Pcdh7	5610	NM 134136	Fbxo38	4416	NM 144901	Csde1	4201
NM 138741	Sdpr	3069	NM 016809	Rbm3	2900	NM 144904	Ptbp3	6880
NM 001009947	Dock11	6665	NM 016738	Rpl13	794	NM 019632	Napb	3693
NM 138744	Ssx2ip	3454	NM 001004364	Asap2	5557	NM 144910	Cnot6l	8564
NM 001166581	BC005561	5181	NM 016785	Tpmt	2715	NM 144915	Daglb	3257
NM 018824	Slc23a2	6487	NM 001004150	A4galt	2105	NM 144917	Elmod3	2251
NM 138750	Prom2	4266	NM 001004152	Psg22	1885	NM 001011873	Xkr9	1926
NM 001010825	Ficd	3192	NM 001004154	Rragb	2308	NM 019867	Ngef	2764
NM 138755	Phf21a	6346	NM 001004176	Maml3	6514	NM 001167981	Galnt7	4363
NM 018745	Azin1	4868	NM 001004185	Whamm	3069	NM 144925	Tnrc6a	8493
NM 138949	Zfp286	3006	NM 001004190	Zfp560	4732	NM 019637	Styx	4258
NM 001166619	Fmr1nb	820	NM 001004193	Rhox8	1206	NM 144941	Map7d1	3402
NM 001166620	Fmr1nb	742	NM 016745	Atp2a3	4593	NM 019717	Atl2	3639
NM 018774	Phc2	3960	NM 016771	Sult1d1	2410	NM 019648	Metap2	4990
NM 139059	Csnk1d	3686	NM 001004357	Cntnap2	7002	NM 001012363	Slc2a9	3403
NM 001166640	Csrp2bp	3265	NM 001004363	Nuak1	5032	NM 145125	Brwd1	8547
NM 139139	Dnajc17	1026	NM 016770	Folh1	3047	NM 019813	Dbn1	2906
NM 001166644	Zfp651	6353	NM 016782	Cntnap1	5360	NM 145132	Mchr1	2205
NM 001166645	Zfp882	5651	NM 016786	Ube2k	4866	NM 001168273	Sec24c	4218
NM 019447	Hgfac	2103	NM 001004435	Pik3r6	3225	NM 001164659	Trank1	10553
NM 139270	Pth2r	2414	NM 134189	Galnt10	4725	NM 019667	Stam2	4124
NM 018830	Asah2	4835	NM 001004761	Gpr158	7143	NM 001012450	Ankrd6	4733
NM 018810	Mkrn1	2936	NM 016803	Chst3	6129	NM 145135	Rnh1	1807
NM 019455	Hpgds	3298	NM 001005343	Sp9	2813	NM 001012451	Ankrd6	4444
NM 139153	Agap3	3207	NM 017367	Ccni	2830	NM 145137	Mgl2	1550
NM 139200	Cytip	5735	NM 001005371	Gm13084	1814	NM 145138	Nek9	5403
NM 139272	Galnt2	4131	NM 001128094	Atp13a3	7220	NM 019770	Tmed2	2029
NM 139233	Fgd4	2216	NM 017370	Hp	1365	NM 145145	Pomt1	2838
NM 139236	Nol6	4522	NM 001005420	Ppp1r26	5485	NM 019671	Net1	3995
NM 019460	Sfmbt1	7694	NM 017375	Ostf1	1714	NM 145148	Frmd4b	5043
NM 139296	Moxd2	1860	NM 001005506	Txlna	4869	NM 001012667	Smim19	1476
NM 139303	Kif18a	3487	NM 017376	Tef	4267	NM 019831	Zmym3	5430
NM 018805	Hs3st3b1	6004	NM 001005507	Smg7	5855	NM 001014974	Ttll4	4341
NM 139308	Stard7	3118	NM 001005508	Arhgap30	4509	NM 019708	Scoc	1946
NM 018797	Plxnc1	7059	NM 016856	Cpsf2	5315	NM 001024205	Nufip2	10399
NM 139310	Otoa	3673	NM 016859	Bysl	3854	NM 019735	Apip	924
NM 144500	Osbpl2	2745	NM 001005605	Aebp2	6192	NM 001081548	Ubr3	8081
NM 018828	Fnbp4	4691	NM 016862	Vti1a	3405	NM 001168386	Ccdc125	2496
NM 144516	Zmynd11	3970	NM 016880	Krt35	1742	NM 145218	Wbscr17	4196
NM 018737	Ctps2	5606	NM 134246	Acot3	2594	NM 145222	B3gnt7	2608
NM 144517	Tbc1d19	2688	NM 001005916	Zbtb9	2804	NM 001013026	Ttf2	4555
NM 018816	Apom	731	NM 016919	Col5a3	6086	NM 019689	Arid3b	3807
NM 144519	Zfp639	2038	NM 134257	Rgs3	3751	NM 145223	Alms1	10005
NM 018831	Dclre1a	4074	NM 017383	Cntn6	3613	NM 019763	Spen	12299
NM 018796	Eef1b2	1933	NM 017391	Slc5a3	10918	NM 145227	Oas2	3873
NM 144529	Arhgap17	3503	NM 001007154	Phactr3	4643	NM 019792	Cyp3a25	2002
NM 144531	Kazn	4914	NM 016912	Cdkl2	4079	NM 001013370	Sesn1	2612
NM 144539	Slamf7	3977	NM 016926	Sart3	3586	NM 001013371	Dtx3l	5181

NM 144541	Bre	1299	NM 001007220	Adam22	2777	NM 145360	Idi1	2883
NM 001011780	Olfr287	1776	NM 001007221	Adam22	2669	NM 001014973	Snx13	6239
NM 144548	Il23r	2488	NM 134448	Dst	23252	NM 001168504	Pla2g4c	3390
NM 144551	Trib2	4198	NM 016867	Gipc2	1286	NM 001081436	Ino80d	13438
NM 144552	Stxbp6	4325	NM 138309	Cd99l2	3548	NM 001013378	Uspl1	3272
NM 144558	Bivm	3571	NM 138310	Apobr	3615	NM 001013379	Zfp930	2996
NM 001167588	Gm17677	351	NM 016860	Actr1a	2742	NM 001013380	Dync1li2	4647
NM_019481	Slc13a1	2495	NM_001007574	A830010M20Rik	6756	NM_019820	Cbln3	4723
NM 018864	Impa1	2601	NM 001007576	Gucy2f	9998	NM 145374	Mios	3724
NM 018878	Paxip1	5864	NM 016851	Irf6	4140	NM 001029934	Usp32	6978
NM 144792	Sgms1	3624	NM 138592	Usp39	2237	NM 001168516	Zdhhc24	2269
NM 018884	Pdzrn3	4106	NM 001009547	Adam26b	2405	NM 001168517	Zdhhc24	2316
NM 018865	Wisp1	5022	NM 019396	Cyhr1	4121	NM 001024950	Zfp563	6383
NM 018868	Nop58	1976	NM 138630	Arhgap4	3321	NM 001168525	Sgms1	4056
NM 144798	Slc30a6	2156	NM 017477	Copg1	4014	NM 001168526	Sgms1	3280
NM 144800	Mtss1	4975	NM 001009548	Adam20	2589	NM 001013387	Zfp182	5920
NM 144803	Chrna2	3527	NM 001007584	Gm11541	1681	NM 145389	BC016579	2192
NM 144806	Prpsap2	1867	NM 017479	Kat6b	7347	NM 019744	Ncoa4	3953
NM 001167736	Chchd6	1042	NM 138650	Dgkg	5521	NM 001024927	Pitpnm3	6682
NM 144807	Chpt1	3239	NM 017476	Akap8l	2097	NM 001029937	Sec14l3	3928
NM 018872	Tmem131	6563	NM 001008498	Wdr34	1812	NM 019793	Tspan3	1747
NM 144809	Prdm9	3460	NM 017469	Gucy1b3	3251	NM 145398	Casd1	3760
NM 018879	Nprl2	1473	NM 017466	Ccrl2	2110	NM 145400	Ube4a	6067
NM 001167746	Dnah17	14889	NM 138654	Sugct	1727	NM 001013391	Cpsf6	6534
NM 144812	Tnrc6b	17334	NM 017467	Zfp316	6893	NM 019727	Snx1	2079
NM 144813	Slc24a1	5244	NM 017461	1-Sep	1466	NM 019760	Serinc1	2889
NM 001167750	Ccdc132	5072	NM 138659	Prpf8	7500	NM 019785	Actr10	4022
NM 144818	Ncaph	2702	NM 138661	Pcdha9	5341	NM 019803	Ube2g2	1982
NM 019491	Rala	2662	NM 138663	Pcdha12	5254	NM 019647	Rpl21	1871
NM 001167775	Rbm10	3715	NM 138664	Dnajc28	3365	NM 019649	Clptm1	4147
NM 001167776	Rbm10	3200	NM 018827	Crlf1	1646	NM 001013608	Ercc6l2	5589
NM 144825	Taok1	12411	NM 138669	Eif4a3	1489	NM 001081652	Nacad	4914
NM 144827	Spata20	2606	NM 019409	Omg	2226	NM 001168620	Enpp5	2472
NM 019496	Ammecr1	3109	NM 001008548	Pde2a	4208	NM 001168624	C2cd4c	6638
NM 144833	Zfp410	2697	NM 138672	Stab1	7995	NM 145432	Heatr6	5677
NM_144834	Serpina10	1570	NM_001008549	Zfp658	3381	NM_001013757	Vwde	3995
NM 144835	Heatr1	6776	NM 001008550	Zfyve26	9368	NM 145436	Cdc27	5783
NM 144837	Ice1	7675	NM 138674	Pkhd1l1	12750	NM 019729	Usp8	4177
NM 019508	Il17b	692	NM 019414	Selenbp2	1695	NM 001168658	Ccdc127	5764
NM 001167828	Trim30d	3294	NM 138679	Ash1l	11293	NM 001013761	Gmnc	4354
NM 144842	Zmym5	5351	NM 138680	Luc7l2	2723	NM 019753	Cdh17	3475
NM 144843	Mtmr6	3845	NM 018822	Sgsh	4324	NM 001029988	Fat2	14423
NM 019501	Pdss1	1638	NM 001009575	Armcx5	2835	NM 019650	Gosr2	3078
NM 019505	Dgke	5208	NM 019418	Tnfsf14	1869	NM 001170401	Tmem229b	3695
NM 019536	Dnah10	14038	NM 018748	Golga4	7532	NM 001170419	Prok2	1324
NM 019540	Pfpl	3918	NM 138721	Lsm10	927	NM 019664	Kcnj15	5222
NM 001167877	Cyp2c50	1891	NM 138741	Sdpr	3069	NM 001170433	Ppfibp1	4893
NM 019519	Rabggta	2547	NM 001009947	Dock11	6665	NM 001013771	Gm973	3615
NM 144861	Rprd1a	4297	NM 138744	Ssx2ip	3454	NM 001170454	Tada2b	3791
NM 144866	Etf1	3718	NM 018824	Slc23a2	6487	NM 019678	Tfg	1948
NM 144868	Pcnxl3	7281	NM 001166583	Fam122b	3412	NM 019679	Fmnl1	3873
NM 019549	Plek	4117	NM 018773	Skap2	1656	NM 001170489	Aplf	3184
NM 144880	Ppp2r5a	3082	NM 018745	Azin1	4868	NM 019683	Ankrd49	1778
NM 019566	Rhog	1264	NM 001166603	Chn1	3876	NM 001170552	Krit1	6034
NM 001167913	Smok2b	2305	NM 018817	Smarcal1	6715	NM 145456	Zswim6	5473
NM 144887	Zdhhc5	4740	NM 001166604	Chn1	3688	NM 001013776	Fbxw24	1506
NM 019565	Zfp386	4575	NM 001166625	Ccr9	4392	NM 145457	Paip1	4676
NM 019587	Plxnb3	6024	NM 019438	Ncapg	3698	NM 145458	Pxk	2807
NM 144899	Adamtsl4	3856	NM 139140	Spats2	2075	NM 019688	Rapgef4	4166
NM 144901	Csde1	4201	NM 001166644	Zfp651	6353	NM 001170638	Slc17a1	1957

NM 144902	Slc35a3	4027	NM 001166645	Zfp882	5651	NM 001170643	Rnf144b	4540
NM 144906	Sgip1	5445	NM 001010941	Gpr12	2485	NM 019883	Uba52	524
NM_001199308	Gm14440	3382	NM_139270	Pth2r	2414	NM_001013784	E130309D14Rik	4002
NM 144910	Cnot6l	8564	NM 001166656	Cdk13	2030	NM 001170669	Pde8b	4619
NM 001167963	G2e3	6703	NM 018800	Syt6	1682	NM 001170691	Elmod2	4704
NM 001167964	G2e3	6352	NM 018815	Nup210	7043	NM 001013786	Zscan26	3990
NM 019645	Pkp1	4486	NM 018812	Pias3	2833	NM 145484	Zfp758	2988
NM 144920	Plekha5	5133	NM 001166685	Cep95	2608	NM 145486	2-Mar	3452
NM 144926	Sez6l2	3812	NM 139229	Cog8	2134	NM 145489	AI661453	5032
NM 019631	Tmem45a	2698	NM 139232	Fgd4	3229	NM 020016	Magea2	1685
NM 144933	Med17	3914	NM 001011733	Olfr288	2083	NM 020019	Magea6	1201
NM 144937	Usp3	2201	NM 139233	Fgd4	2216	NM 020020	Magea8	1646
NM 019651	Ptpn9	4087	NM 139234	Fgd4	2406	NM 001013820	Slc22a28	2004
NM 144942	Csad	2265	NM 018786	Prpf40b	3182	NM 001018079	Lce3f	627
NM 019640	Pitpnb	2761	NM 018788	Extl3	5995	NM 145493	Cplx4	1767
NM 001012325	Zfp708	2567	NM 019458	Paf1	1953	NM 145495	Rin1	4176
NM 144944	Prokr2	4533	NM 139293	Ece2	3129	NM 020005	Kat2b	4654
NM 019643	Fam60a	2640	NM 019460	Sfmbt1	7694	NM 001170787	Cntn5	4270
NM 001017393	Gm21943	1206	NM 139296	Moxd2	1860	NM 001170800	Ipcef1	5561
NM 019660	Mycbp	1485	NM 139303	Kif18a	3487	NM 145505	Fam160b1	5630
NM 001012330	Zbtb18	3848	NM 139308	Stard7	3118	NM 020025	B3galt2	4578
NM 145122	Pex16	1470	NM 018797	Plxnc1	7059	NM 020026	B3galnt1	2283
NM 001012363	Slc2a9	3403	NM 018769	Dfna5	2133	NM 001013824	Gm5662	1842
NM 001168255	Slc45a4	3737	NM 139310	Otoa	3673	NM 001170849	Luc7l2	5062
NM 145129	Chrna3	3123	NM 139311	Mllt6	7271	NM 001170855	Trim36	4555
NM 001168260	Foxred2	4910	NM 144515	Zfp52	3164	NM 019958	Rgs17	8121
NM 001164659	Trank1	10553	NM 144516	Zmynd11	3970	NM 001170884	Trim43b	2262
NM 019667	Stam2	4124	NM 144517	Tbc1d19	2688	NM 145529	Cstf3	2852
NM 001168274	Gsdmc2	2312	NM 144522	Tbc1d10b	3614	NM 001170953	Rnmt	4853
NM 001012448	Zfp708	2479	NM 144523	Zfp622	2804	NM 145540	Ints3	4460
NM 001012449	Zfp708	2471	NM 018829	Ap3m1	4424	NM 001170960	Dtwd2	2857
NM 001012450	Ankrd6	4733	NM 018831	Dclre1a	4074	NM 001014997	Gm156	682
NM 001012451	Ankrd6	4444	NM 018772	Bri3	963	NM 145544	Rap1gds1	3527
NM 145138	Nek9	5403	NM 144532	Cabp4	1472	NM 145545	Gbp7	5622
NM 145140	Abcc10	5472	NM 144541	Bre	1299	NM 019912	Ube2d2a	2483
NM 145141	Fcrla	1658	NM 001011780	Olfr287	1776	NM 001015889	Taf9	1248
NM 145143	Mpp4	2422	NM 144548	Il23r	2488	NM 145551	Slc5a9	4370
NM 145146	Afm	2048	NM 144550	Spice1	4342	NM 019917	Vmn2r26	2568
NM 019671	Net1	3995	NM 144556	Lgi4	3444	NM 019919	Ltbp1	7910
NM 145150	Prc1	3049	NM 144558	Bivm	3571	NM 001171000	Ahcyl2	5215
NM 019743	Rybp	4376	NM 019477	Acsl4	4990	NM 145559	Slc2a9	3276
NM 019811	Acss2	2833	NM 144788	Hectd1	8988	NM 001171001	Ahcyl2	4928
NM 145209	Oasl1	2124	NM 018864	Impa1	2601	NM 001018002	Dppa4	1378
NM 001024205	Nufip2	10399	NM 018878	Paxip1	5864	NM 001171003	Mgam	6488
NM 145217	Diras1	2948	NM 001167669	Spag7	1131	NM 019991	Prl2a1	886
NM 001081548	Ubr3	8081	NM 018865	Wisp1	5022	NM 001018042	Sp3	4191
NM 019754	Tagln3	1269	NM 144796	Susd4	2035	NM 145565	Sds	1164
NM 001013022	Odf3b	1075	NM 018867	Cpxm2	3506	NM 001171034	Tmbim6	2612
NM 145220	Appl2	2958	NM 144799	Lmcd1	1742	NM 145570	Eva1a	1768
NM 001013024	Usp13	4686	NM 144803	Chrna2	3527	NM 001171035	Tmbim6	2468
NM 001168475	Ttc23	2231	NM 001167730	Rad18	2701	NM 001171036	Tmbim6	2433
NM 001168476	Ttc23	2031	NM 019487	Hebp2	1910	NM 001024137	Pla2g4d	3311
NM 019792	Cyp3a25	2002	NM 001167736	Chchd6	1042	NM 001024139	Adamts15	5946
NM 019658	Shoc2	4116	NM 144808	Slc39a14	4927	NM 001024142	Fbxo10	4655
NM 001013371	Dtx3l	5181	NM 018872	Tmem131	6563	NM 001024606	Pdp2	3514
NM 019666	Syncrip	2782	NM 144809	Prdm9	3460	NM 145584	Spon1	6166
NM 001029935	Trim38	1480	NM 144810	Klhdc8a	4269	NM 145589	Prr14	2325
NM 001013373	Tmprss13	3245	NM 018879	Nprl2	1473	NM 001024468	Bcat1	7849
NM 019774	Akap8	3695	NM 001167746	Dnah17	14889	NM 001024478	Cdhr3	2953
NM 001013375	Utp18	2613	NM 144813	Slc24a1	5244	NM 019988	Mlst8	3361
NM 001014973	Snx13	6239	NM 001167750	Ccdc132	5072	NM 001024508	Brd9	2494

NM 001168504	Pla2g4c	3390	NM 019490	Uso1	3914	NM 019980	Litaf	2240
NM 001015099	G2e3	6634	NM 144817	Camk1g	2467	NM 001024526	Larp4	6548
NM 001081436	Ino80d	13438	NM 018859	Akr1e1	1729	NM 019989	Sh3bgrl	2856
NM 001168505	Shoc2	4075	NM 144819	Ccdc92	2256	NM 001024539	Shc2	3974
NM 145370	Gps1	2000	NM 001167777	Asxl3	11697	NM 001172064	Lrrc56	2554
NM 001013378	Uspl1	3272	NM 019492	Rgs3	4705	NM 020012	Rnf14	3056
NM 019653	Wsb1	2490	NM 019581	Gtpbp2	2982	NM 001172065	Lrrc56	2417
NM 001024950	Zfp563	6383	NM 144825	Taok1	12411	NM 001024618	Xirp2	11955
NM 001013382	Lrrc52	1167	NM 144829	Aarsd1	1346	NM 145609	Msantd4	2762
NM 001168525	Sgms1	4056	NM 144833	Zfp410	2697	NM 001172092	Depdc1a	3333
NM_001168526	Sgms1	3280	NM_144834	Serpina10	1570	NM_001172093	Depdc1a	2502
NM 001168535	Cdadc1	3209	NM 144843	Mtmr6	3845	NM 145611	Kank2	4853
NM 001168536	Cdadc1	1907	NM 019497	Grk4	2408	NM 001172096	Cacul1	5718
NM 145382	Fam193b	4356	NM 019501	Pdss1	1638	NM 001172097	Cacul1	5579
NM 001168537	Cdadc1	2221	NM 019505	Dgke	5208	NM 001172098	Strn3	3912
NM 001024206	Trappc1	745	NM 001167860	Wipf3	4204	NM 145616	Lrrc49	3000
NM 001013386	Rasl10b	3481	NM 019536	Dnah10	14038	NM 001172100	Rnh1	1656
NM 001013387	Zfp182	5920	NM 019537	Psmg1	1017	NM 001172101	Rnh1	1659
NM 001168568	Ctps2	5474	NM 001167861	Wipf3	4102	NM 145617	Herc1	15229
NM 019744	Ncoa4	3953	NM 144851	Senp1	6415	NM 145618	Ice2	4070
NM 001168569	Ctps2	5531	NM 019542	Nagk	1330	NM 001172107	Gramd1c	3276
NM 001168571	Ctps2	5453	NM 019515	Nmu	828	NM 001024703	Mctp2	5810
NM 001168577	Nat2	1375	NM 144858	Dus3l	2321	NM 020049	Slc6a14	3433
NM 019793	Tspan3	1747	NM 144861	Rprd1a	4297	NM 019994	Ralgapa1	8280
NM 019760	Serinc1	2889	NM 144865	Reep2	1926	NM 001024706	Gm5458	1004
NM 019767	Arpc1a	1588	NM 144866	Etf1	3718	NM 001172121	Rbms3	7409
NM 019785	Actr10	4022	NM 001167891	Nrg2	2839	NM 001172122	Rbms3	7884
NM 001013411	Nkain2	3205	NM 144872	Eml3	3194	NM 145622	Zfp65	3960
NM 019823	Cyp2d22	2782	NM 144878	Fmo4	1787	NM 001172123	Rbms3	7932
NM 001013577	Inip	3221	NM 001167910	Mbtps1	4244	NM 001172124	Rbms3	7804
NM 019716	Orc6	1663	NM 019575	Scamp4	1802	NM 145628	Uspl1	3464
NM 019673	Actl6a	1969	NM 019567	Acin1	2519	NM 020252	Nrxn1	9040
NM 001013608	Ercc6l2	5589	NM 019574	Patz1	3100	NM 001172126	Rbms3	7756
NM 145422	Rtcb	1979	NM 144887	Zdhhc5	4740	NM 145633	Ankrd27	4292
NM_001168590	2010106E10Rik	1287	NM_019577	Ccl24	1289	NM_020255	Scand1	780
NM 001081652	Nacad	4914	NM 144892	Ncoa5	3151	NM 001024720	Hmcn1	18316
NM 001168600	Gnl3l	4844	NM 019576	Thsd1	4432	NM 020263	Cacna2d2	5533
NM 145424	BC089597	1281	NM 019587	Plxnb3	6024	NM 001172152	Wdr17	4147
NM 001013749	Tmem151b	4853	NM 144899	Adamts14	3856	NM 020267	Trim44	5612
NM 001168602	Smok3a	2723	NM 001167944	Zkscan5	3610	NM 020265	Dkk2	3705
NM 019654	Socs5	4460	NM 144904	Ptbp3	6880	NM 145760	Arfgap1	2721
NM 001013753	Pcdh17	9509	NM 019632	Napb	3693	NM 001024806	Cebpz	4091
NM_001013755	5730409E04Rik	2850	NM_144907	Sesn2	2677	NM_001024846	Zfp62	4034
NM 001168658	Ccdc127	5764	NM 144908	Galnt11	2541	NM 020260	Arhgap31	7722
NM 001013760	Gm4937	1040	NM 019645	Pkp1	4486	NM 145745	Adam34	2524
NM 001013761	Gmnc	4354	NM 144919	Hdac11	2494	NM 145823	Pitpnc1	6350
NM 019741	Slc2a5	3096	NM 144920	Plekha5	5133	NM 020276	Nsmf	2880
NM 001013765	Zscan4c	2276	NM 144922	Hnrnpul1	2884	NM 020274	Htr3b	2411
NM 001029988	Fat2	14423	NM 001167991	Hook2	2614	NM 145838	St8sia6	3166
NM 145441	Ubxn2a	2511	NM 144925	Tnrc6a	8493	NM 020330	Adam21	2911
NM 019650	Gosr2	3078	NM 144926	Sez6l2	3812	NM 001024911	10-Sep	2549
NM 001045530	Ccnjl	2709	NM 019631	Tmem45a	2698	NM 001024922	Ddx49	2104
NM 001170430	Slc35b3	1951	NM 019651	Ptpn9	4087	NM 001024928	Zfp667	3671
NM 019664	Kcnj15	5222	NM 001168225	Morf4l2	1947	NM 020495	Slco1b2	3296
NM 001170431	Slc35b3	2086	NM 001168226	Morf4l2	1880	NM 145852	Ropn1l	910
NM 001013769	Rsl1	2046	NM 001168227	Morf4l2	1832	NM 020487	Prss21	1077
NM 001170433	Ppfibp1	4893	NM 001168228	Morf4l2	1831	NM 020507	Tob2	3994
NM 001170454	Tada2b	3791	NM 144942	Csad	2265	NM 145920	Evc2	4111
NM 019678	Tfg	1948	NM 019873	Fkbpl	1173	NM 001079931	Gm5347	2564

NM 001170488	Tprkb	2241	NM 001168229	Morf4l2	1803	NM 001024945	Qsox1	3399
NM 001170489	Aplf	3184	NM 001168230	Morf4l2	1741	NM 145928	Tspan14	2500
NM 001170552	Krit1	6034	NM 001012330	Zbtb18	3848	NM 145930	AW549877	5094
NM 019684	Srpk3	1983	NM 145123	Crtac1	2616	NM 145931	Zc3h7a	3857
NM 001170561	Sbf1	6268	NM 019747	Zfp113	6486	NM 145933	St6gal1	4508
NM 001040669	Gm5169	917	NM 001168255	Slc45a4	3737	NM 145937	Sumf1	2601
NM 145467	Itgbl1	2357	NM 019813	Dbn1	2906	NM 145938	Rpp40	2736
NM 001170643	Rnf144b	4540	NM 019874	Dnajb5	2327	NM 001025085	Gm5797	1028
NM_001013784	E130309D14Rik	4002	NM_001164659	Trank1	10553	NM_145939	Alg3	1425
NM 001170669	Pde8b	4619	NM 145133	Tifa	2164	NM 145943	Sde2	3203
NM 001170691	Elmod2	4704	NM 001012450	Ankrd6	4733	NM 145947	Slc26a7	2371
NM 019697	Kcnd2	4562	NM 001012451	Ankrd6	4444	NM 020558	C1d	2957
NM 001013786	Zscan26	3990	NM 145137	Mgl2	1550	NM 001081642	Xlr4a	1191
NM 019711	Rbms2	5207	NM 145138	Nek9	5403	NM 145950	Osgin2	2647
NM 145483	Zfp160	3809	NM 001168292	R3hdm2	4051	NM 001025156	Ccdc93	7272
NM 001170742	Wdr59	5111	NM 145142	Chst10	3135	NM 020584	Terf2ip	3599
NM 001170743	Wdr59	5168	NM 145143	Mpp4	2422	NM 020604	Jph1	4809
NM_020010	Cyp51	4353	NM_001168294	Serpina3f	2254	NM_145956	Brcc3	4335
NM 001013802	Macrod2	1893	NM 145149	Rasgrp4	4621	NM 020605	Jph3	3903
NM 145491	Rhoq	4159	NM 145150	Prc1	3049	NM 145958	Kbtbd2	3868
NM 001013817	Sp140	2417	NM 019743	Rybp	4376	NM 145962	Pank3	7365
NM 001013820	Slc22a28	2004	NM 145152	Lrrc3	4311	NM 020608	Crampl1	7534
NM 020005	Kat2b	4654	NM 145153	Oas1f	1480	NM 020610	Nrip3	3913
NM 145497	Nmrk1	1637	NM 019821	Gltp	1622	NM 020579	B4galt3	1998
NM 001017394	Gm20815	1796	NM 001168333	Tinagl1	1954	NM 145968	Tagap	3076
NM 145505	Fam160b1	5630	NM 145158	Emilin2	3910	NM 001025261	Tpd52	2432
NM 020025	B3galt2	4578	NM 019676	Plcd1	2651	NM 001025262	Tpd52	2363
NM_145509	5430435G22Rik	2539	NM_019831	Zmym3	5430	NM_001025263	Tpd52	2308
NM 001170849	Luc7l2	5062	NM 019707	Cdh13	2290	NM 145972	BC027231	2866
NM 145513	Tiprl	4395	NM 145211	Oas1a	1889	NM 001025264	Tpd52	2293
NM 145514	Wdr26	7063	NM 001014390	Dyrk2	2137	NM 145973	Ell3	1745
NM 145516	Plekhb2	3317	NM 001168403	Ankrd55	2887	NM 001025286	Nkain2	1233
NM 001170855	Trim36	4555	NM 001168404	Ankrd55	1005	NM 145977	Slc45a3	3348
NM 019945	Mast1	4922	NM 001168405	Ankrd55	1556	NM 001025296	Dffa	2467
NM 001170869	Wibg	1165	NM 001013024	Usp13	4686	NM 020626	Tmem27	1262
NM 145528	Atg13	3573	NM 145221	Appl1	6961	NM 020594	Zc3h8	1615
NM 001170911	Prr13	1111	NM 001013026	Ttf2	4555	NM 020570	Xrcc2	3230
NM 145532	Mall	1967	NM 145223	Alms1	10005	NM 020588	Tmem183a	3169
NM 019984	Tgm1	2803	NM 001168469	Ube2m	1228	NM 001025372	Adcyap1r1	6178
NM 019986	Habp4	2584	NM 019763	Spen	12299	NM 020578	Ehd3	3658
NM 001170960	Dtwd2	2857	NM 145226	Oas3	4718	NM 020593	Fbxo3	1404
NM 001014997	Gm156	682	NM 001013362	Npcd	4891	NM 001025377	Arhgap15	1816
NM 019918	Vmn2r1	2739	NM 001013360	Npcd	4600	NM 001025378	Orc2	3030
NM 145544	Rap1gds1	3527	NM 145227	Oas2	3873	NM 021293	Cd33	2488
NM 019933	Ptpn4	4707	NM 145228	Oas1h	1708	NM 001025605	Gm527	1491
NM 145546	Gtf2b	1267	NM 019792	Cyp3a25	2002	NM 146009	Cep290	8006
NM 001014996	Cenpj	4385	NM 001013370	Sesn1	2612	NM 146011	Arhgap9	1811
NM 019912	Ube2d2a	2483	NM 019658	Shoc2	4116	NM 146012	Ctdsp2	4136
NM_001015681	E130308A19Rik	3720	NM_145356	Zbtb7c	4591	NM_146013	Sec14l4	2956
NM 145548	Cyp2j13	4374	NM 001168498	Ncmap	1635	NM 021301	Slc15a2	4007
NM 145551	Slc5a9	4370	NM 019768	Morf4l2	1954	NM 146019	Chd3	7296
NM_145557	9430015G10Rik	2510	NM_001168500	Ncmap	1837	NM_001025380	Adam39	2441
NM 001171000	Ahcyl2	5215	NM 145362	Alg1	1772	NM 021306	Ecel1	2898
NM 145559	Slc2a9	3276	NM 019782	Lepre1	3030	NM 001025382	Fam196b	5371
NM 001171001	Ahcyl2	4928	NM 019783	Lepre1	3210	NM 021307	Zfp112	3507
NM 019946	Mgst1	943	NM 019791	Maged1	2752	NM 021312	Wdr12	2946
NM 001017966	Ddi2	3539	NM 001081436	Ino80d	13438	NM 146042	Rnf144b	4576

NM 001017983	Foxred2	4812	NM 001168505	Shoc2	4075	NM 146047	Clptm1l	2378
NM 001171003	Mgam	6488	NM 145368	Acnat2	1659	NM 146068	Spidr	3303
NM 001171004	Prkd3	5887	NM 001015039	Zfyve28	3980	NM 146081	Ppp4r1	3864
NM 001018031	Otol1	2161	NM 019828	Trpc4ap	3195	NM 001025568	Pde1c	8864
NM 001171005	Prkd3	5083	NM 001013378	Uspl1	3272	NM 146089	Haus1	1004
NM 001018042	Sp3	4191	NM 001013380	Dync1li2	4647	NM 021343	Spata5	2907
NM 019928	Klk4	1237	NM 019820	Cbln3	4723	NM 001025572	Ankrd12	8991
NM 001171010	Slc14a1	3880	NM 145374	Mios	3724	NM 021345	Ptplad1	2738
NM 001171011	Slc14a1	3615	NM 019653	Wsb1	2490	NM 021346	Zfp318	3921
NM 001171034	Tmbim6	2612	NM 001029934	Usp32	6978	NM 146097	Cbwd1	1944
NM 001171035	Tmbim6	2468	NM 001168515	Rpgrip1	4626	NM 021041	Abcc9	7538
NM 001171036	Tmbim6	2433	NM 001168516	Zdhhc24	2269	NM 021042	Abcc9	7433
NM 019922	Crtap	1714	NM 001168517	Zdhhc24	2316	NM 001025575	Cfhr2	1619
NM 001024139	Adamts15	5946	NM 145378	Pla2g4b	3859	NM 001025576	Ccdc141	6329
NM 145576	Zfp212	2722	NM 001024950	Zfp563	6383	NM 021353	Slc26a3	2911
NM_145582	Ctu1	2476	NM_001168557	A830010M20Rik	4787	NM_146112	Gigyf2	5743
NM 001171187	Mal	2624	NM 001013387	Zfp182	5920	NM 001029893	Psg26	2082
NM 019940	Zfp111	7134	NM 145389	BC016579	2192	NM 021362	Pappa	11027
NM 019967	Brinp1	3354	NM 145390	Tnpo2	4866	NM 001025581	Kcnc2	6196
NM 019969	Plag1	4635	NM 019759	Dpt	1636	NM 021364	Clec5a	3745
NM 145591	Zfp958	2361	NM 001024927	Pitpnm3	6682	NM 001025582	Dram2	2461
NM 001024478	Cdhr3	2953	NM 001029937	Sec14l3	3928	NM 146126	Sord	2259
NM 001171512	Obscn	24175	NM 145398	Casd1	3760	NM 001025584	Kcnj6	2356
NM 019988	Mlst8	3361	NM 019822	Adrm1	1429	NM 001025585	Kcnj6	2049
NM 145598	Nxnl1	2785	NM 019727	Snx1	2079	NM 001025590	Kcnj6	3638
NM 019936	Cript	1171	NM 019785	Actr10	4022	NM 001025599	Trim26	3231
NM 001024526	Larp4	6548	NM 019803	Ube2g2	1982	NM 021371	Caln1	2586
NM 001024539	Shc2	3974	NM 001013411	Nkain2	3205	NM 021372	Sertad2	5730
NM 019956	Krt71	2193	NM 145409	Chtf18	3163	NM 001025610	Ms4a7	1279
NM 019998	Alg2	3062	NM 019823	Cyp2d22	2782	NM 001025613	Otud7b	8101
NM_145604	D230025D16Rik	2873	NM_019709	Mbtps1	4393	NM_001025614	Otud7b	8043
NM 145606	Chmp1a	2188	NM 145417	Rnpep	2301	NM 001025779	Cdc6	4532
NM 145607	Ttc13	3049	NM 001045525	Cyb5d1	794	NM 146156	Pdik1l	4766
NM 145608	BC021891	5692	NM 145419	Hkdc1	3497	NM 021385	Rad18	2560
NM 145609	Msantd4	2762	NM 145422	Rtcb	1979	NM 146159	Haus3	1980
NM 001024624	Cdkl5	3246	NM 001013741	Ddn	3740	NM 001029838	Pknox2	3550
NM 145612	Zfp810	3238	NM 019861	Ctsf	1975	NM 146175	Zfp282	5222
NM 001172096	Cacul1	5718	NM 001168615	Tifab	3498	NM 001029867	Ugt2b36	1888
NM 001172097	Cacul1	5579	NM 001045521	Prss38	1239	NM 001029876	Urb2	5773
NM 001024952	Rc3h1	11011	NM 001013753	Pcdh17	9509	NM 001029877	Nova2	8372
NM 145617	Herc1	15229	NM 019693	Ddx39b	1756	NM 001029889	Gm608	12622
NM_001024672	Mettl7a2 Higd1c	1449	NM_001081644	Gm428	1325	NM_021412	Mmp19	3410
NM 145620	Rrp9	1537	NM 001168679	Tspan8	1380	NM 001029912	Zswim5	5397
NM 019914	Mllt11	2514	NM 019753	Cdh17	3475	NM 146208	Neil3	2271
NM 001024706	Gm5458	1004	NM 001168680	Tspan8	1416	NM 146213	Ces4a	2433
NM 001172121	Rbms3	7409	NM 001168684	Tmcc3	5451	NM 146215	Cmtr2	3577
NM 001172122	Rbms3	7884	NM 001013765	Zscan4c	2276	NM 021429	Hs1bp3	2958
NM 145622	Zfp65	3960	NM 019778	Zbtb20	3156	NM 021431	Nudt11	2649
NM 001172123	Rbms3	7932	NM 001013767	Capn11	2575	NM 146224	Zfp280d	4374
NM 145627	Rbm10	3431	NM 019650	Gosr2	3078	NM 021434	Gpr180	2877
NM 001172124	Rbms3	7804	NM 001045527	Hsf5	4158	NM 001029982	Sec23ip	4578
NM 145628	Usp11	3464	NM 001170433	Ppfibp1	4893	NM 146234	Mmgt1	4084
NM 145632	Polr2h	787	NM 001013771	Gm973	3615	NM 001029983	Man1b1	3748
NM 001172126	Rbms3	7756	NM 001170454	Tada2b	3791	NM 001029990	Mettl17	1637
NM 001024720	Hmcn1	18316	NM 001170479	Loh12cr1	1635	NM 146241	Trhde	5722
NM 020283	B3galt1	5222	NM 001170485	Arpc4	2040	NM 021450	Trpm7	7145
NM 020273	Gmeb1	6518	NM 019679	Fmnl1	3873	NM 001030289	Mmp27	1656
NM 020267	Trim44	5612	NM 001170486	Arpc4	2152	NM 021453	Pga5	1416
NM 020265	Dkk2	3705	NM 145450	BC022687	1915	NM 001030293	Spry3	4542

NM 020284	Ctsr	1335	NM 001013774	Kpna7	2248	NM 001030296	Prr7	1360
NM 020278	Lgi1	4285	NM 001170552	Krit1	6034	NM 001037742	Tyw5	1448
NM 145708	Obox2	1343	NM 145456	Zswim6	5473	NM 021463	Prps1	1998
NM 001024731	Gm20939	2514	NM 019684	Srpk3	1983	NM 001045523	Bahd1	4428
NM 020260	Arhgap31	7722	NM 145457	Paip1	4676	NM 001031664	Nudt10	1772
NM 145745	Adam34	2524	NM 145460	Oxnad1	2062	NM 001045515	Synj1	7012
NM 145823	Pitpnc1	6350	NM 145462	Haus4	1588	NM 001031851	Agxt2	1825
NM 145831	Dmrt2	2337	NM 001013777	Zfp488	4318	NM 021477	Rbfox1	5204
NM 020330	Adam21	2911	NM 145470	Deptor	8549	NM 001033208	Myzap	2283
NM 145851	Cables2	2945	NM 145473	Csdc2	2507	NM 021492	Ap3b2	3816
NM 020506	Xpo4	3456	NM 145474	Cyp2d34	1622	NM 021493	Arhgap23	5450
NM 020507	Tob2	3994	NM 001170669	Pde8b	4619	NM 001033300	Gmps	4567
NM 001079931	Gm5347	2564	NM 001170691	Elmod2	4704	NM 021504	Ngly1	2901
NM 001024932	Pilrb2	1807	NM 145476	Tbc1d22a	3248	NM 001033432	Heca	3667
NM 001024954	Pbx4	1359	NM 001013785	Akr1c19	1242	NM 001033043	Rnf17	5148
NM 145930	AW549877	5094	NM 001170694	Rcbtb2	3411	NM 021508	Myoz1	1294
NM 145931	Zc3h7a	3857	NM 145479	Klhl22	2633	NM 021509	Moxd1	3083
NM 145934	Stap2	1532	NM 001170742	Wdr59	5111	NM 021510	Hnrnph1	2242
NM 001045529	Morc3	4237	NM 001170743	Wdr59	5168	NM 001033135	Rnf149	2440
NM 145938	Rpp40	2736	NM 001170744	Ctbp2	4170	NM 021518	Rab2a	2057
NM 001025085	Gm5797	1028	NM 020010	Cyp51	4353	NM 001033142	Rnf166	1783
NM 145940	Wipi1	1817	NM 145491	Rhoq	4159	NM 001037221	Samd4	7120
NM 001025093	Atf2	4300	NM 020016	Magea2	1685	NM 001033156	Fbxo33	3446
NM 020601	Tbl1x	2302	NM 020018	Magea5	1563	NM 001033162	Shcbp1l	2045
NM 001081642	Xlr4a	1191	NM 001013813	Maml2	6548	NM 001033167	Slc22a23	6251
NM 145950	Osgin2	2647	NM 001013816	Gm5622	1609	NM 021535	Smu1	3273
NM_001025156	Ccdc93	7272	NM_020019	Magea6	1201	NM_001033176	1700039E15Rik	1732
NM 001025163	Zfp78	4751	NM 020020	Magea8	1646	NM 001033178	Tmem181a	4430
NM 020561	Smpdl3a	1791	NM 001013820	Slc22a28	2004	NM 021547	Stard3	2100
NM 020584	Terf2ip	3599	NM 145492	Zfp521	6152	NM 001033194	Gtf3c3	3005
NM 020604	Jph1	4809	NM 145497	Nmrk1	1637	NM 001033192	C78339	1381
NM 145958	Kbtbd2	3868	NM 145499	Cyp2c70	1709	NM 021554	Mettl9	1850
NM 020619	Mogs	2798	NM 001017394	Gm20815	1796	NM 021557	Rdh11	1574
NM 001025286	Nkain2	1233	NM 001170800	Ipcef1	5561	NM 001033199	AI747448	2997
NM 145977	Slc45a3	3348	NM 019963	Stat2	4402	NM 001033207	Nlrc5	6945
NM 020626	Tmem27	1262	NM 145505	Fam160b1	5630	NM 021566	Jph2	4235
NM 001025312	Dclre1b	4143	NM 145510	Rabif	2005	NM 001033209	Xylb	3860
NM 001025364	Rtn2	1080	NM 145511	BC003331	3255	NM 001033210	Pls1	3697
NM 020585	Golga7	2031	NM 001170848	Luc7l2	2693	NM 021604	Agrn	7379
NM 020588	Tmem183a	3169	NM 001170849	Luc7l2	5062	NM 001081641	Pitpnm3	6634
NM 001025373	Zfp943	2736	NM 001170855	Trim36	4555	NM 001033222	Pdzd8	7076
NM 146003	Senp6	4938	NM 145523	Gca	3288	NM 001033237	Yeats2	6024
NM 001025378	Orc2	3030	NM 001170884	Trim43b	2262	NM 021716	Fign	9759
NM 144761	Crygb	633	NM 145528	Atg13	3573	NM 001033241	Gm382	4070
NM 001080941	Zfp429	2033	NM 145534	Btbd3	4979	NM 001033243	Ccdc114	2453
NM 145076	Trim24	6191	NM 019984	Tgm1	2803	NM 001033249	Zfp583	2765
NM 001025607	Gm5168	852	NM 001170953	Rnmt	4853	NM 021792	Iigp1	2966
NM 146016	Eml6	8080	NM 145539	Tm4sf4	1417	NM 021880	Prkar1a	3323
NM_001076791	Zfp874b	3792	NM_001170954	A4galt	2145	NM_001033258	D10Bwg1379e	8216
NM 021301	Slc15a2	4007	NM 001014997	Gm156	682	NM 021884	Tsg101	1832
NM 146019	Chd3	7296	NM 145548	Cyp2j13	4374	NM 001033259	Mcu	2872
NM 021303	Noc2l	2785	NM 145552	Gnl2	2350	NM 021897	Trp53inp1	5392
NM 001025380	Adam39	2441	NM 145555	Ncmap	1834	NM 021899	Foxj2	5013
NM_021306	Ecel1	2898	NM_145557	9430015G10Rik	2510	NM_001033274	Brd1	5137
NM 001025382	Fam196b	5371	NM 001171000	Ahcyl2	5215	NM 001033275	Gxylt1	6534
NM 001025383	Gm14685	2773	NM 001171001	Ahcyl2	4928	NM 001033283	Oosp3	823
NM 001025384	DXBay18	2862	NM 145562	Parm1	2100	NM 001033285	Cdc42bpa	8656
NM 146034	Ctage5	2930	NM 001171003	Mgam	6488	NM 001033291	Usp40	5049
NM 021309	Sh2d2a	4758	NM 001171005	Prkd3	5083	NM 001033294	Ddx31	3328

NM 146039	Wdr60	3725	NM 001018042	Sp3	4191	NM 022018	Fam129a	3956
NM 021312	Wdr12	2946	NM 145564	Fbxo21	3909	NM 001033296	Sel1l2	2219
NM_146042	Rnf144b	4576	NM_001171024	Nup62-il4il	2320	NM_022022	Ube4b	5535
NM 021315	Noc3l	3977	NM 145569	Mat2a	2804	NM 001033299	Zfp217	5685
NM 021318	Fhl5	1135	NM 145570	Eva1a	1768	NM 022026	Aqp9	2660
NM_146052	Lrrc3b	1676	NM_001024139	Adamts15	5946	NM_001033304	5330417C22Rik	3326
NM 146062	Pphln1	4035	NM 001024141	Nxf3	2978	NM 001033311	Vsig10	4054
NM 146066	Gspt1	6856	NM 145577	Zfp772	2772	NM 001033318	Cog7	2884
NM 021328	Bin3	1727	NM 019996	Phax	1893	NM 001033322	Gucy1a2	13727
NM 146068	Spidr	3303	NM 020006	Cdc42ep4	3263	NM 022323	Moap1	3728
NM 146069	Nrros	3559	NM 145578	Ube2m	1295	NM 001033331	Gas2l3	6745
NM 021330	Acp1	3107	NM 001024145	Pla2g4f	3125	NM 001033346	Lrrc55	2900
NM_001025444	Aptx	5554	NM_001171187	Mal	2624	NM_001033347	D430041D05Rik	10124
NM 146071	Muc20	2555	NM 019940	Zfp111	7134	NM 022424	Fndc4	2270
NM 001025445	Aptx	5549	NM 145587	Sbk1	4092	NM 001033349	Ankub1	1759
NM 146078	Ubr2	7696	NM 019965	Dnajb12	1789	NM 145066	Gpr85	2971
NM 146079	Guca1b	1681	NM 001024458	Add1	3917	NM 001033361	Pgr15l	5379
NM 146081	Ppp4r1	3864	NM 145589	Prr14	2325	NM 022655	Ireb2	5808
NM_146083	Srsf7	2301	NM_001024468	Bcat1	7849	NM_001033375	A230046K03Rik	5863
NM 001025571	Sh2d2a	4734	NM 019967	Brinp1	3354	NM 022881	Rgs18	1859
NM 146091	Atl3	6387	NM 019969	Plag1	4635	NM 022883	Lpin3	3473
NM 146092	Taf6l	2203	NM 145594	Fgl1	1120	NM 022884	Bhmt2	2090
NM 146093	Ubxn1	1048	NM 001171512	Obscn	24175	NM 001033400	Gm806	2548
NM 146104	Aph1a	3215	NM 145597	Tmem161a	2994	NM 022890	Cldn12	3794
NM 146105	Cnst	4568	NM 001024508	Brd9	2494	NM 001033409	Lgr6	3623
NM 146106	Lyplal1	1577	NM 019956	Krt71	2193	NM 001033419	Ceacam16	1635
NM 021353	Slc26a3	2911	NM 019998	Alg2	3062	NM 001033430	Kdm7a	9553
NM 001029893	Psg26	2082	NM 145603	Ces2c	1978	NM 001033439	Lrch1	4727
NM_021362	Pappa	11027	NM_145604	D230025D16Rik	2873	NM_001033444	Capn13	2614
NM 001025581	Kcnc2	6196	NM 001024618	Xirp2	11955	NM 001033445	Garem	4783
NM 021366	Klf13	6396	NM 001172073	Ttc4	2570	NM 001033447	Gm960	2262
NM 146130	Hnrnpa3	5242	NM 145607	Ttc13	3049	NM 001033457	Nom1	3542
NM_146133	Golph3l	2846	NM_145616	Lrrc49	3000	NM_001033465	6430531B16Rik	1161
NM 146134	Aph1a	2882	NM 001024645	Lrrc16b	4620	NM 001033475	Tmed8	7179
NM 021369	Chrna6	2921	NM 001024952	Rc3h1	11011	NM 001033477	Gm1322	1966
NM 021370	Asic5	1657	NM 145617	Herc1	15229	NM 001033479	Gm1527	2120
NM 001025617	Cant1	2957	NM 145619	Parp3	2633	NM 001033485	BC048644	2260
NM 001025618	Cant1	2915	NM 001172107	Gramd1c	3276	NM 001033484	Iqgap3	5676
NM 146156	Pdik1l	4766	NM 001024703	Mctp2	5810	NM 001033500	Wdr72	5234
NM 146157	Emc1	6237	NM 020049	Slc6a14	3433	NM 001033532	AW554918	5375
NM 021387	Vstm2b	1455	NM 001045536	Zzef1	11150	NM 001033534	Layn	1786
NM 001026214	Entpd5	4987	NM 001024719	Cyp2c67	1707	NM 001033542	Slc47a2	2377
NM 146161	Arhgap24	3159	NM 001024720	Hmcn1	18316	NM 001033600	Acsl4	4987
NM 146162	Tmem119	2132	NM 020283	B3galt1	5222	NM 001033621	Myot	2207
NM 146167	Gimap7	1289	NM 020270	Scamp5	3307	NM 001033634	Zyg11b	10654
NM 146169	Paip2b	3330	NM 020263	Cacna2d2	5533	NM 001033773	Ube2u	1517
NM 146173	Tspan33	1959	NM 020273	Gmeb1	6518	NM 001033794	Fbxw18	1519
NM 001029850	Magi1	7806	NM 145700	Ackr4	2109	NM 001033805	Tmem253	1227
NM_146175	Zfp282	5222	NM_020267	Trim44	5612	NM_001033880	4930415L06Rik	2618
NM 021402	Ube2j2	3520	NM 020265	Dkk2	3705	NM 001033988	Ncoa4	4043
NM_146185	Zfp790	4525	NM_145760	Arfgap1	2721	NM_001034037	1700024G13Rik	399
NM_146186	Wdr62	4744	NM_145710	Obox6	1336	NM_001034097	Tnfsf12Tnfsf13	2235
NM 001029876	Urb2	5773	NM 145711	Tox	2852	NM 001034851	Fam134b	3139

NM 146189	Mybpc2	3624	NM 020258	Slc37a2	4383	NM 001034859	Gm4841	2838
NM 021408	Ush2a	15891	NM 001024806	Cebpz	4091	NM 001034868	Mrgprx2	4559
NM 146190	Tubgcp5	3681	NM 001029987	Doxl2	2288	NM 001037167	Samt2	1011
NM 001029889	Gm608	12622	NM 145745	Adam34	2524	NM 001034905	Spag11b	1740
NM 021412	Mmp19	3410	NM 145824	Ranbp10	5308	NM 001034906	Trim43a	1836
NM 001029977	Gm4788	2887	NM 020264	Pate4	1016	NM 001034962	Sorbs1	5282
NM 021416	Fam184b	4136	NM 020295	Lmbr1	4949	NM 001034963	Sorbs1	5312
NM 146202	Zfp768	2268	NM 020296	Rbms1	2688	NM 001034964	Sorbs1	5275
NM 146208	Neil3	2271	NM 020274	Htr3b	2411	NM 001035239	Trpm3	6742
NM 146218	Rfwd3	4443	NM 145838	St8sia6	3166	NM 001035240	Trpm3	6742
NM 146221	Zfp426	3557	NM 001024856	Ttbk2	11152	NM 001035242	Trpm3	6706
NM 021434	Gpr180	2877	NM 001024857	Ttbk2	11078	NM 001035510	Zcchc18	3327
NM 021435	Slc35b4	3697	NM 001024910	10-Sep	1821	NM 001035854	Ap2b1	5411
NM_021437	1700123020Rik	1889	NM_001024911	10-Sep	2549	NM_001036740	B3gnt8	1475
NM 021439	Chst11	5532	NM 145841	Sgcz	2389	NM 001037136	Agap1	9422
NM 146240	Rassf9	2118	NM 001024928	Zfp667	3671	NM 001037539	Prok2	1450
NM 146241	Trhde	5722	NM 145916	Zfp7	2785	NM 001037709	Rusc2	5317
NM 021450	Trpm7	7145	NM 145920	Evc2	4111	NM 001037746	Prps1l3	4010
NM 021455	Mlxipl	3649	NM 001079931	Gm5347	2564	NM 001037717	Slc38a6	2959
NM 001030293	Spry3	4542	NM 145921	Olah	1522	NM 001037723	Adcy7	6033
NM 001030294	Olfm4	1640	NM 145925	Pttg1ip	2275	NM 001037724	Adcy7	5969
NM 146248	Cchcr1	2676	NM 145929	Gga1	3034	NM 001037725	Fam117b	5534
NM 146253	Zbtb6	4535	NM 145931	Zc3h7a	3857	NM 001146153	Homer3	2445
NM 001037745	Zfp791	2359	NM 020486	Bcam	2407	NM 027626	Psd3	9228
NM 001037748	Gm20736	956	NM 001045529	Morc3	4237	NM 146339	Olfr77	2671
NM 001031621	Abca17	5202	NM 001025083	Rhox12	935	NM 001290141	Ccdc169	2365
NM 001081657	Gm5935	904	NM 020572	Aurkc	1332	NM 010214	Fhl4	2670
NM 021466	Taf1a	2430	NM 001025093	Atf2	4300	NM 147098	Olfr630	1545
NM 001031664	Nudt10	1772	NM 001025103	Efcab4a	1687	NM 030263	Psd3	9570
NM 001045515	Synj1	7012	NM 145943	Sde2	3203	NM 001165256	Dcaf4	2040
NM 021477	Rbfox1	5204	NM 020580	Nelfcd	2279	NM 177644	Rasal2	10046
NM 001032413	Pear1	4367	NM 020603	Wdr46	2235	NM 207551	Olfr209	918
NM 001032414	Pear1	4297	NM 001025156	Ccdc93	7272	NM 001290138	Ccdc169	2890
NM 021481	Treh	2053	NM 001025163	Zfp78	4751	NM 178790	Abi3bp	4394
NM_001033145	1190002N15Rik	3950	NM_145952	Tbc1d12	4209	NM_001162489	Mroh1	5111
NM_021492	Ap3b2	3816	NM_020595	Otor	929	NM_001204905	4930558K02Rik	1013
NM 001033319	Ppfia1	5184	NM 020604	Jph1	4809	NM 001204916	H60c	2817
NM_021502	Trappc2l	634	NM_145959	D15Ertd621e	5621	NM_001014422	Abi3bp	4454
NM 001033043	Rnf17	5148	NM 020606	Parva	4450	NM 207555	Olfr372	1016
NM 021512	Nup160	5684	NM 145962	Pank3	7365	NM 178737	AW551984	4293
NM 021516	Mark3	3388	NM 020577	As3mt	1752	NM 016888	B3gnt2	2699
NM 001037221	Samd4	7120	NM 145970	Cc2d1a	3349	NM 021471	Slco1c1	3178
NM 001033156	Fbxo33	3446	NM 145973	Ell3	1745	NM 001169114	B3gnt2	2576
NM 001033158	Rasl12	2430	NM 145976	Tifab	3434	NM 001014423	Abi3bp	4694
NM 001033159	Zfp597	5630	NM 020619	Mogs	2798	NM 001168270	Qars	2155
NM 021532	Dact1	3650	NM 001080550	Wfdc8	2549	NM 172777	Gbp9	3633
NM 021536	Rhot1	4137	NM 020565	Sult3a1	1096	NM 001014399	Abi3bp	3884
NM 021541	Cryba2	740	NM 020570	Xrcc2	3230	NM 011984	Homer3	2436
NM 001033178	Tmem181a	4430	NM 001025365	Miip	1830	NM 199257	Tpte	2549
NM 021548	Arpp19	4032	NM 020588	Tmem183a	3169	NM 178247	Dppa1	2350
NM 001033194	Gtf3c3	3005	NM 020578	Ehd3	3658	NM 177698	Psd3	11334
NM 001033192	C78339	1381	NM 001025378	Orc2	3030	NM 001163358	Dppa1	2264
NM 001033199	AI747448	2997	NM 144761	Crygb	633	NM 001177772	Slco1c1	3175
NM 001033202	Usp30	2790	NM 146006	Lss	3192	NM 001162910	Gm3604	2919
NM 001033207	Nlrc5	6945	NM 021295	Lancl1	4277	NM 021550	C1galt1c1	1507
NM 001033210	Pls1	3697	NM 146010	Tspan8	1522	NM 001162917	Dennd4a	8655
NM 021604	Agrn	7379	NM 145076	Trim24	6191	NM 001014424	Abi3bp	4502
NM 021605	Nek7	3366	NM 146013	Sec14l4	2956	NM 148943	Usp9y	8094

NM 031164	F13b	2378	NM 146015	Efemp1	2036	NM 001081473	Zxdb	5623
NM 021606	Nek6	3168	NM 146016	Eml6	8080	NM 023617	Aox3	4360
NM 001033219	Slc45a4	4659	NM 146019	Chd3	7296	NM 173761	Ythdf1	3199
NM 001033221	G6b	2252	NM 021302	Stk32c	2183	NM 177680	Ythdc1	3034
NM 001033225	Pnrc1	1940	NM 001025380	Adam39	2441	NM 023631	Aox4	4976
NM 001033231	Fam195b	1286	NM 146027	Scrn2	1534	NM 001008419	Aox2	4776
NM 001033237	Yeats2	6024	NM 001025382	Fam196b	5371	NM 027494	Zcchc8	4286
NM 021716	Fign	9759	NM 146037	Kcnk13	3061	NM 178897	Tyw1	4698
NM 001033241	Gm382	4070	NM 021312	Wdr12	2946	NM 145857	Nod2	4621
NM 001033242	Cln5	2447	NM 146050	Oit1	1251	NM 175836	Sptbn1	8265
NM 001033249	Zfp583	2765	NM 146052	Lrrc3b	1676	NM 010587	Itsn1	13976
NM 021793	Tmem8	3480	NM 021322	Wdr4	3906	NM 010349	Grik2	4872
NM 021878	Jarid2	5716	NM 001025431	Btbd3	4677	NM 001024726	Zfp607	3511
NM 021879	Oca2	3120	NM 146063	Krt79	2113	NM 133740	Prmt3	2495
NM 001033256	Spata5l1	2328	NM 146065	Atf7	2867	NM 175266	Epm2aip1	7273
NM 001033257	Phactr2	8284	NM 146066	Gspt1	6856	NM 009231	Sos1	8919
NM_001033258	D10Bwg1379e	8216	NM_021327	Tnip1	2838	NM_001110214	Dclre1c	3731
NM 021885	Tub	5941	NM 021328	Bin3	1727	NM 008967	Ptgir	3334
NM 021889	Syt9	3816	NM 021330	Acp1	3107	NM 009214	Sms	3377
NM 021891	Fignl1	2964	NM 001025444	Aptx	5554	NM 007641	Ms4a1	2057
NM 001033271	Tmem55b	2753	NM 001025445	Aptx	5549	NM 181549	Clec18a	1836
NM_021897	Trp53inp1	5392	NM_146075	Lemd2	2603	NM_013495	Cpt1a	4331
NM 001033275	Gxylt1	6534	NM 146078	Ubr2	7696	NM 053011	Lrp1b	14743
NM 001033285	Cdc42bpa	8656	NM 146081	Ppp4r1	3864	NM 145404	Prmt7	2269
NM 001033288	Sbspon	2748	NM 001025568	Pde1c	8864	NM 001082414	Sh3d19	6414
NM_022016	Impg1	3587	NM_021340	Rgr	1659	NM_001122767	2310022A10Rik	1885
NM 022022	Ube4b	5535	NM 146089	Haus1	1004	NM 018747	Akap7	3140
NM 001033299	Zfp217	5685	NM 001025572	Ankrd12	8991	NM 032540	Kel	2524
NM 001033301	Fhdc1	5930	NM 021344	Tesc	942	NM 028611	Ndufaf7	2303
NM 001033306	Shb	2762	NM 146091	Atl3	6387	NM 001081252	Uggt2	6514
NM 001033314	Ccdc61	2010	NM 146093	Ubxn1	1048	NM 001077359	Git2	4944
NM 022315	Smoc2	2832	NM 021041	Abcc9	7538	NM 172748	Fbxl19	3431
NM 001033318	Cog7	2884	NM 021042	Abcc9	7433	NM 001005510	Syne2	21718
NM 001033321	Tmem231	2887	NM 001025576	Ccdc141	6329	NM 026149	Nudcd1	3447
NM 001033322	Gucy1a2	13727	NM 021362	Pappa	11027	NM 010333	S1pr2	2815
NM 022378	Foxb1	2984	NM 146126	Sord	2259	NM 009664	Ambn	1860
NM 001033342	Cdc42bpg	5994	NM 021366	Klf13	6396	NM 001111268	Grik2	4785
NM 001033343	Sec31b	3923	NM 146131	Pbxip1	4481	NM 175137	Vars2	4441
NM 022420	Gprc5b	4525	NM 146135	Pias3	2975	NM 008615	Me1	3257
NM 001033361	Pgr15l	5379	NM 021372	Sertad2	5730	NM 001114663	Plcl1	6555
NM 022655	Ireb2	5808	NM 001025613	Otud7b	8101	NM 010449	Hoxa1	2474
NM 001033369	Acvr1c	8666	NM 146151	Tesk2	3032	NM 019834	Git2	5027
NM_001033375	A230046K03Rik	5863	NM_001025614	Otud7b	8043	NM_009592	Abcb7	5759
NM 022801	Mark3	3343	NM 146152	Ipo13	3588	NM 001114332	Slc16a10	5394
NM_001033378	A430078G23Rik	2970	NM_021382	Tacr3	3764	NM_146114	Dclre1c	3759
NM 001033399	Gfod1	6654	NM 021383	Rqcd1	3285	NM 025812	Hmg20a	3562
NM 022889	Pes1	2911	NM 001025779	Cdc6	4532	NM 010690	Lats1	7222
NM 001033405	Treml2	4509	NM 146157	Emc1	6237	NM 172148	B9d2	1058
NM 022890	Cldn12	3794	NM 021385	Rad18	2560	NM 009782	Cacna1e	12697
NM 001033408	Rbm44	3851	NM 146159	Haus3	1980	NM 177733	E2f2	4746
NM 001033421	Pate2	2215	NM 001026214	Entpd5	4987	NM 009260	Sptbn1	9049
NM 001033424	Fnd3c2	3658	NM 021392	Ap4m1	1734	NM 001077360	Git2	4940
NM 001033430	Kdm7a	9553	NM 146169	Paip2b	3330	NM 053080	Aldh1a3	3423
NM 001033434	Gm884	4576	NM 021398	Slc43a3	2601	NM 008611	Mmp8	2453
NM 001033439	Lrch1	4727	NM 146175	Zfp282	5222	NM 029447	Nln	3780
NM 001033443	Cdk14	2931	NM 021402	Ube2j2	3520	NM 001098425	Sp3	4100
NM 001033444	Capn13	2614	NM 146182	Klc3	1849	NM 010844	Muc5ac	8505

NM 001033446	Arl14epl	2247	NM 001029872	Itgad	3975	NM 025718	Dnase1l2	1236
NM 001033460	Drc1	2485	NM 146185	Zfp790	4525	NM 178691	Yod1	3843
NM 001033475	Tmed8	7179	NM 146186	Wdr62	4744	NM 145452	Rasal	5101
NM 001033488	Mfsd2b	3125	NM 001029877	Nova2	8372	NM 001081242	Tln2	12150
NM 001033493	Gpr111	3283	NM 146189	Mybpc2	3624	NM 198100	Tbkbp1	3358
NM 001033498	Gramd2	1368	NM 021408	Ush2a	15891	NM 008048	Igfbp7	1120
NM 001033499	Sh2d1b2	399	NM 001029889	Gm608	12622	NM 153566	Yrdc	1558
NM 001033536	Rfx7	8000	NM 146193	Btbd1	3020	NM 008000	Fert2	2383
NM 001033542	Slc47a2	2377	NM 001029895	Ate1	5020	NM 011360	Sgce	1711
NM 001033606	Acsl3	3810	NM 001029912	Zswim5	5397	NM 001159518	Igfbp7	1213
NM 001033633	Slc2a13	6541	NM 021416	Fam184b	4136	NM 133727	Kptn	1665
NM 001033634	Zyg11b	10654	NM 146215	Cmtr2	3577	NM 173874	Clcn3	5781
NM 001033668	Dnah1	12872	NM 021429	Hs1bp3	2958	NM 007811	Cyp26a1	1781
NM 001033759	Tmem2	6620	NM 146223	Cplx3	2859	NM 175406	Atp6v0d2	2518
NM 001033773	Ube2u	1517	NM 146227	Prss50	1455	NM 001142741	Pcdh15	8974
NM 001033791	Gm12888	1181	NM 146228	Als2cl	5103	NM 178414	Acsm4	2153
NM 001033795	Zcchc16	3634	NM 146232	Slc22a26	2016	NM 018743	Agpat6	3944
NM_001033799	4930428D18Rik	1103	NM_146235	Ercc6l	4198	NM_175236	Adhfe1	3139
NM 001033813	Zfp872	2960	NM 001029983	Man1b1	3748	NM 023115	Pcdh15	9064
NM 001033820	Zfp551	2336	NM 001029990	Mettl17	1637	NM 008569	Anapc1	8967
NM_001033880	4930415L06Rik	2618	NM_001029993	Zc3h18	3814	NM_001142738	Pcdh15	8923
NM 001033978	H2-Eb2	3380	NM 021448	Elp2	2789	NM 001130188	Sgce	1819
NM 001033988	Ncoa4	4043	NM 021450	Trpm7	7145	NM 001142736	Pcdh15	9043
NM_001034097	Tnfsf12Tnfsf13	2235	NM_001030289	Mmp27	1656	NM_015763	Lpin1	5581
NM 001034859	Gm4841	2838	NM 021452	Kcnmb4	1032	NM 025710	Uqcrfs1	1313
NM 001034862	Erich1	1579	NM 146246	Rpl11	6811	NM 007711	Clcn3	5529
NM 001034863	Tmem136	3500	NM 146252	Tbc1d13	3487	NM 010922	Mrpl40	1055
NM 001034864	Gm4907	1310	NM 146253	Zbtb6	4535	NM 013880	Plcl2	4068
NM 001034893	Zfp936	3112	NM 001037749	Slc22a14	2229	NM 001130412	Lpin1	5626
NM 001034895	Zfr2	3358	NM 021466	Tafla	2430	NM 024124	Hdac9	4461
NM 001034899	Gm16432	1663	NM 001031664	Nudt10	1772	NM 173873	Clcn3	5705
NM 001034900	Zfp345	2706	NM 021468	Unc13b	6302	NM 013770	Slc25a10	4000
NM 001034902	Gm5878	4643	NM 021474	Efemp2	1561	NM 001012310	Slc25a47	1609
NM 001037168	Psg27	1989	NM 021477	Rbfox1	5204	NM 001142737	Pcdh15	8836
NM 001034962	Sorbs1	5282	NM 001032413	Pear1	4367	NM 001142739	Pcdh15	8983
NM 001034963	Sorbs1	5312	NM 001032414	Pear1	4297	NM 026658	Mto1	2364
NM 001034964	Sorbs1	5275	NM 001032727	Sybu	2948	NM 178386	Slc25a31	2110
NM_001035122	Golm1	3557	NM_001033157	5730507C01Rik	2470	NM_016687	Sfrp4	1780
NM 001035239	Trpm3	6742	NM 001033201	Ncbp1	3167	NM 011690	Vars	4224
NM 001035231	Zfp748	4615	NM 001033208	Myzap	2283	NM 001142735	Pcdh15	9049
NM 001035241	Trpm3	6712	NM 021492	Ap3b2	3816	NM 001127338	Aldh7a1	3105
NM 001035240	Trpm3	6742	NM 001033300	Gmps	4567	NM 173876	Clcn3	5250
NM 001035242	Trpm3	6706	NM 021499	Wdr8	1600	NM 001142740	Pcdh15	9034
NM 001035243	Trpm3	6676	NM 001033319	Ppfia1	5184	NM 172950	Lpin1	5482
NM 001035245	Trpm3	1637	NM 001033432	Heca	3667	NM 027930	Mtfr2	2307
NM 001035246	Trpm3	3851	NM 001033128	Bbs1	5646	NM 001081340	Setd2	8350
NM 001035509	Zcchc18	2856	NM 001033525	Kcnk6	4089	NM 172737	Shisa7	6058
NM 001035510	Zcchc18	3327	NM 021516	Mark3	3388	NM 021377	Sorcs1	7241
NM 001036293	Nrbf2	1789	NM 001033149	Ttc9	2042	NM 145629	Pls3	3251
NM 001037098	Nacc2	6893	NM 001033162	Shcbp1l	2045	NM 007400	Adam12	7675
NM 001037143	Pinlyp	730	NM 021538	Cope	1043	NM 008861	Pkd2	5221
NM_001037136	Agap1	9422	NM_001033172	Rab11fip2	5304	NM_011705	Vrk1	3796
NM 001037321	Fbxo40	2518	NM 001033174	Osbp	4590	NM 025863	Trim59	2920
NM 001037294	Alpk2	7250	NM 021542	Kcnk5	3364	NM 001198933	Me1	3335
NM 001037493	Slc41a3	2240	NM 145070	Hip1r	4402	NM 001166375	1-Mar	3942
NM 001037665	Zfp1	1900	NM 001033192	C78339	1381	NM 028232	Sgol1	3609
NM 001037707	Zfp27	3529	NM 021554	Mettl9	1850	NM 007628	Ccna1	1581

NM 001037712	Kcnh6	2853	NM 021557	Rdh11	1574	NM 009836	Cct3	1957
NM 001037740	Strip2	5286	NM 021566	Jph2	4235	NM 022654	Pidd1	2900
NM 028446	Trip11	6448	NM 001081641	Pitpnm3	6634	NM 001204334	Cyp4f14	2051
NM 027626	Psd3	9228	NM 021605	Nek7	3366	NM 010345	Grb10	5089
NM 010214	Fhl4	2670	NM 021606	Nek6	3168	NM 010774	Mbd4	3584
NM 178382	Flrt3	3984	NM 001033213	Ttc7b	3458	NM 001098203	Hic1	3253
NM 030263	Psd3	9570	NM 001033219	Slc45a4	4659	NM 138657	Socs7	7015
NM 001206392	Hk3	3099	NM 021710	Ap4s1	1113	NM 001100461	Pnma5	2290
NM_001195437	Gm590	499	NM_001033233	Tmprss11a	2657	NM_008636	Mtf1	7631
NM 172456	Exog	3870	NM 001033237	Yeats2	6024	NM 010430	Hic1	4247
NM 172284	Ddx19b	6639	NM 021716	Fign	9759	NM 001029844	Vrk1	3664
NM 001165256	Dcaf4	2040	NM 001033241	Gm382	4070	NM 172818	Ttll8	2976
NM 172740	Zfp420	3334	NM 001033243	Ccdc114	2453	NM 008561	Mc3r	2623
NM 177644	Rasal2	10046	NM 001033247	Wdr52	6583	NM 175188	1-Mar	4391
NM 001190490	Dmpk	2683	NM 021792	Iigp1	2966	NM 028959	Cep72	3632
NM 178790	Abi3bp	4394	NM 021878	Jarid2	5716	NM 177290	Itgb8	3096
NM 001163073	Lcorl	4970	NM 021880	Prkar1a	3323	NM 001029843	Vrk1	3724
NM 001081379	Ankrd11	8719	NM 021879	Oca2	3120	NM 021414	Ahcyl2	5218
NM_001114679	9930111J21Rik1	3863	NM_001033257	Phactr2	8284	NM_008784	Igbp1	1518
NM_001014422	Abi3bp	4454	NM_001033258	D10Bwg1379e	8216	NM_153179	Pkhd1	12928
NM 013643	Ptpn5	3134	NM 001033259	Mcu	2872	NM 007756	Cplx1	2217
NM 001163518	Ccdc166	2414	NM 021889	Syt9	3816	NM 011798	Xcr1	4453
NM 001206391	Hk3	2894	NM 021890	Fads3	3269	NM 022981	Zfp110	3549
NM 178737	AW551984	4293	NM 001033267	Qrich2	2162	NM 001166372	1-Mar	4223
NM_146059	Ccdc166	2301	NM_021897	Trp53inp1	5392	NM_145443	L2hgdh	3338
NM 001164661	Cyfip1	6524	NM 001033272	Spata13	7533	NM 001204333	Cyp4f14	2054
NM 001025241	Gm20747	1123	NM 021899	Foxj2	5013	NM 201369	N4bp2l2	8998
NM 130866	Olfr78	3496	NM 001033275	Gxylt1	6534	NM 023651	Pex13	4122
NM 001014423	Abi3bp	4694	NM 001033285	Cdc42bpa	8656	NM 001162943	Dchs1	10651
NM 001161816	Gm15455	3016	NM 022018	Fam129a	3956	NM 001161843	Il18r1	1793
NM 022724	Suv39h2	4282	NM 001033303	Ampd1	2342	NM 001242379	Slc4a10	5424
NM 021507	Sqrdl	1995	NM 022026	Aqp9	2660	NM 020282	Nqo2	3974
NM 032418	Dmpk	2761	NM 001033312	Fbxl18	2831	NM 010391	H2-Q10	1473
NM 001014399	Abi3bp	3884	NM 001033316	Ffar3	1625	NM 146684	Olfr1440	948
NM 177698	Psd3	11334	NM 022315	Smoc2	2832	NM 022882	Lpin2	5651
NM 001013575	Olfr112	3175	NM 001033321	Tmem231	2887	NM 009270	Sqle	2748
NM 001206390	Hk3	3029	NM 001033322	Gucy1a2	13727	NM 001253879	Mtf2	4347
NM 001190800	Ddx19b	6634	NM 022319	Clstn2	4494	NM 028063	Trmu	1520
NM 001168503	Olfr78	2627	NM 001033323	Igsf9b	2857	NM 001111050	Cd151	1666
NM 011050	Pdcd4	2418	NM 001033331	Gas2l3	6745	NM 011215	Ptprn2	4688
NM 001172160	Flrt3	4537	NM 022416	Stk32b	3458	NM 029702	Arfrp1	2590
NM 001162917	Dennd4a	8655	NM 001033344	Dusp27	4042	NM 134007	Cisd1	965
NM 001198561	H2-Q7	1134	NM 022420	Gprc5b	4525	NM 001253878	Mtf2	4424
NM_001014424	Abi3bp	4502	NM_001033347	D430041D05Rik	10124	NM_008428	Kcnj8	2188
NM 001190491	Dmpk	2624	NM 022427	Gpr88	3450	NM 016917	Slc40a1	3380
NM 148943	Usp9y	8094	NM 022811	Polr1e	3547	NM 001252459	Cyfip2	6622
NM_001289661	2810474019Rik	6163	NM_001033360	Gpr101	5425	NM_173010	Ube3a	3888
NM 026184	Ero1lb	4255	NM 001033361	Pgr15l	5379	NM 001242383	Slc4a10	5421
NM_001289662	2810474019Rik	6076	NM_001033365	Mroh5	2242	NM_010551	Il16	4989
NM 027494	Zcchc8	4286	NM 001033369	Acvr1c	8666	NM 001163241	Nqo2	3892
NM_145393	Ythdf2	4078	NM_001033375	A230046K03Rik	5863	NM_024166	Chchd2	910
NM 178897	Tyw1	4698	NM 022801	Mark3	3343	NM 019674	Ppp4c	1332
NM 175836	Sptbn1	8265	NM 022879	Myl7	599	NM 001081105	Rhoh	4960
NM 010587	Itsn1	13976	NM 001033393	Tmem104	4583	NM 007819	Cyp3a13	2955

NM 001024726	Zfp607	3511	NM 022884	Bhmt2	2090	NM 138599	Tomm70a	3778
NM 175266	Epm2aip1	7273	NM 001033400	Gm806	2548	NM 001165991	Arfrp1	2596
NM 009231	Sos1	8919	NM 001033416	Gal3st4	3162	NM 134156	Actn1	3739
NM 027241	Polr3gl	1402	NM 001033418	Bsph1	822	NM 001253877	Mtf2	4705
NM 009525	Wnt5b	2384	NM 001033419	Ceacam16	1635	NM 025773	Ube2w	3139
NM 007825	Cyp7b1	2166	NM 001033427	Lyg2	2564	NM 033552	Slc4a10	5463
NM 178759	Timd4	2175	NM 001033430	Kdm7a	9553	NM 182959	Slc17a8	4452
NM 001113565	Serbp1	6640	NM 001033439	Lrch1	4727	NM 001242380	Slc4a10	5553
NM 145573	Mrps35	1067	NM 001033457	Nom1	3542	NM 011680	Usf2	2419
NM 013495	Cpt1a	4331	NM 001033463	Tatdn2	2646	NM 011983	Homer2	10999
NM 001082414	Sh3d19	6414	NM 001033466	Zbtb2	3227	NM 001242382	Slc4a10	5511
NM 172402	Slc25a32	2945	NM 001033485	BC048644	2260	NM 001253880	Mtf2	4062
NM 025652	Gtf3a	1315	NM 001033484	Iqgap3	5676	NM 001242381	Slc4a10	5460
NM 001039530	Parp14	7251	NM 001033488	Mfsd2b	3125	NM 001164086	Homer2	10966
NM 011193	Pstpip1	1853	NM 001033496	Zfp213	2686	NM 015730	Chrna4	4509
NM 001099624	Rapgef2	6544	NM 001033500	Wdr72	5234	NM 007430	Nr0b1	1794
NM 010348	Grik1	3573	NM 001033533	Ccdc102a	2309	NM 011989	Slc27a4	4008
NM_032540	Kel	2524	NM_001033535	Tnfaip8l3	2225	NM_010930	Nov	2366
NM 001037128	Musk	3384	NM 001033536	Rfx7	8000	NM 025969	Timm21	1315
NM 153591	Nars2	3770	NM 001033600	Acsl4	4987	NM 009472	Unc5c	9296
NM 146072	Grik1	3660	NM 001033621	Myot	2207	NM 001161842	Il18r1	1919
NM_001042612	Nlrp9c	3020	NM_001033630	2310061I04Rik	1252	NM_019836	Noa1	2303
NM 007936	Epha4	6328	NM 001033634	Zyg11b	10654	NM 001102430	Arfgef1	7042
NM 007497	Atf1	2329	NM 001033668	Dnah1	12872	NM 207221	Jmjd1c	8382
NM 028946	Slc9b1	2245	NM 001033711	Evi2a	2316	NM 145997	Kdm5a	10969
NM 001081256	Kdm3b	7112	NM 001033759	Tmem2	6620	NM 025695	Smc6	5603
NM_080436	Rdh1	3890	NM_001033764	4931408C20Rik	4331	NM_001165995	Arfrp1	2483
NM_028611	Ndufaf7	2303	NM_001033775	4933422H20Rik	1932	NM_001271016	Ube2w	1551
NM_013826	Mocs2	1889	NM_001033819	9130409I23Rik	1898	NM_001164087	Homer2	10982
NM_009739	Bckdk	2857	NM_001033880	4930415L06Rik	2618	NM_001163687	Naaa	2418
NM_175550	Ap4e1	6524	NM_001033908	Med22	1367	NM_001276425	St6galnac4	3641
NM 001005510	Syne2	21718	NM 001034874	Shisa6	7487	NM 145960	Mtrf1	1862
NM_026149	Nudcd1	3447	NM_001034856	4930430D24Rik	1194	NM_001271760	Adra1a	3814
NM 010333	S1pr2	2815	NM 001034858	Armc2	4865	NM 001271017	Ube2w	3060
NM 010944	Musk	3354	NM 001034864	Gm4907	1310	NM 001271709	Lrrn3	3539
NM 016901	Oaz3	1136	NM 001034868	Mrgprx2	4559	NM 001177629	Grb10	4764
NM 175137	Vars2	4441	NM 001034891	Ermard	1580	NM 001271587	Eps8	4561
NM 001114663	Plcl1	6555	NM 001037167	Samt2	1011	NM 001271694	Kcnj15	5061
NM 008622	Mpv17	1712	NM 001034895	Zfr2	3358	NM 001276292	Wwp1	6296
NM 001113566	Serbp1	6622	NM 001034902	Gm5878	4643	NM 025430	Mrpl35	3695
NM 030260	Zxdc	5172	NM 001035122	Golm1	3557	NM 133769	Cyfip2	6385
NM 001034866	Pnldc1	1689	NM 001035228	St3gal5	2241	NM 008420	Kcnb1	11153
NM 009592	Abcb7	5759	NM 001035239	Trpm3	6742	NM 028052	Synpr	2551
NM 028801	Muc5b	14963	NM 001035231	Zfp748	4615	NM 001276315	Cdkl2	4256
NM 010952	Oaz2	1861	NM 001035241	Trpm3	6712	NM 008404	Itgb2	2862
NM 146114	Dclre1c	3759	NM 001035240	Trpm3	6742	NM 001277925	Ecel1	2906
NM 010690	Lats1	7222	NM 001035242	Trpm3	6706	NM 001271526	Orc2	3555
NM 175384	Cdca2	3568	NM 001035243	Trpm3	6676	NM 001281803	Def8	3877
NM 009260	Sptbn1	9049	NM 001035244	Trpm3	3653	NM 001271687	Kcnj15	5210
NM 173002	Zxdc	4122	NM 001035245	Trpm3	1637	NM 022723	Scube1	7842
NM 013897	Timm8b	527	NM 001035246	Trpm3	3851	NM 001277992	Stk16	2857
NM 001098425	Sp3	4100	NM 001036740	B3gnt8	1475	NM 173442	Gcnt1	4612
NM 010844	Muc5ac	8505	NM 001037134	Ccne2	3113	NM 013461	Adra1a	4277
NM 008507	Sh2b3	2498	NM 001037136	Agap1	9422	NM 001271689	Kcnj15	5177

NM 178691	Yod1	3843	NM 001037294	Alpk2	7250	NM 001271691	Kcnj15	5118
NM 145452	Rasa1	5101	NM 001037746	Prpsl13	4010	NM 178657	Oog1	1840
NM 011992	Rcn2	2042	NM 001037723	Adcy7	6033	NM 001271692	Kcnj15	5106
NM 001110162	Cdca2	3865	NM 001037724	Adcy7	5969	NM 145820	Veph1	6450
NM 001037130	Musk	3330	NM 001037725	Fam117b	5534	NM 009842	Cd151	1909
NM 175485	Prtg	8764	NM 001037727	Arhgap25	3695	NM 001271595	Eps8	4566
NM 138591	Gfm1	2598	NM 001290122	Rbm4	2594	NM 001111049	Cd151	1791
NM_198100	Tbkbp1	3358	NM_001290128	Rbm14-rbm4	2420	NM_001271693	Kcnj15	5073
NM_001037129	Musk	3360	NM_001290127	Rbm14-rbm4	2843	NM_001011684	Nms	1019
NM 153566	Yrdc	1558	NM 001290124	Rbm4	1912	NM 001286007	Tubgcp2	3158
NM 008000	Fert2	2383	NM 001290125	Rbm4	1948	NM 001174086	Shisa9	4796
NM 178667	Tfdp2	7384	NM 001290123	Rbm4	2583	NM 028277	Shisa9	4844
NM 001142760	Pcdh15	9135	NM 009032	Rbm4	2630	NM 001286987	Dram2	2597
NM 175406	Atp6v0d2	2518	NM 028446	Trip11	6448	NM 029404	Phf14	7436
NM 001142746	Pcdh15	9150	NM 001168561	Zfp446	4498	NM 001033351	Grin3a	7667
NM 015824	Orc3	6313	NM 001177782	Gpr174	4955	NM 001286255	Nadk2	3590
NM 001161797	Phactr4	4504	NM 027626	Psd3	9228	NM 001271695	Kcnj15	5020
NM 008231	Hdgf	2257	NM 001200002	Tmppe	6872	NM 027462	Wars2	4388
NM 001163538	Pgam5	2079	NM 001033251	Gpr174	5050	NM 001286986	Dram2	2719
NM_173745	Dusp18	4632	NM_001167593	Spata31d1b	4281	NM_001271690	Kcnj15	5138
NM 175236	Adhfe1	3139	NM 001256050	Esp24	433	NM 001079849	Paip1	4458
NM 008569	Anapc1	8967	NM 178382	Flrt3	3984	NM 001114339	Pank1	7419
NM 001145821	Ggta1	3550	NM 030263	Psd3	9570	NM 001287167	Nup98	3961
NM 181325	Slc25a15	3469	NM 001206392	Hk3	3099	NM 001289615	Btn2a2	2924
NM 133718	Tmem30a	3680	NM 001177659	Odf2	3919	NM 147176	Homer1	4804
NM 025562	Fis1	802	NM 001199235	Sptbn4	4861	NM 001271566	Cdc7	3137
NM 010631	Kifc3	3288	NM 001105189	Vmn2r78	2562	NM 001277315	Fam134b	3228
NM_181315	Car5b	3465	NM_001195435	1190003K10Rik	1097	NM_028779	Ampd2	3694
NM 030112	Rtf1	4680	NM 001195348	Bbip1	2248	NM 001081006	Etl4	7413
NM 028273	Pgam5	2076	NM 001190490	Dmpk	2683	NM 001290375	Camk1d	7183
NM 001163565	Ptpn5	3049	NM 201387	Plec	14946	NM 001290374	Camk1d	7108
NM 001162503	Sqrdl	1676	NM 029419	Apol7a	2392	NM 133690	Atp1b4	4278
NM 009061	Rgs2	3009	NM 001011802	Olfr1318	1110	NM 001290392	Sntg1	6999
NM 001145832	Kifc3	2945	NM 001195338	Bbip1	2509	NM 001290368	Nmt2	4429
NM 016897	Timm23	1162	NM 001206391	Hk3	2894	NM 177236	Atp2b3	6748
NM 001145831	Kifc3	3431	NM 017468	Enam	5490	NM 001290419	Snapc4	4507
NM 011690	Vars	4224	NM 147108	Olfr979	1045	NM 026887	Ap1s2	3327
NM 175306	Phactr4	4585	NM 001164661	Cyfip1	6524	NM 177343	Camk1d	7226
NM 010283	Ggta1	3586	NM 021471	Slco1c1	3178	NM 008708	Nmt2	4461
NM 001163243	Fis1	966	NM 001033215	Zfp957	2751	NM 027671	Sntg1	7267
NM 001159563	Orc3	6310	NM 172921	Nxpe4	3173	NM 023739	Nfx1	4556
NM 001081340	Setd2	8350	NM 001205339	Psat1	2295	NM 026293	Spaca1	1073
NM 172737	Shisa7	6058	NM 032418	Dmpk	2761	NM 001080549	Hdx	4261
NM 020043	Igdcc4	6227	NM 001177781	Gpr174	5211	NM 153125	Sec16a	8754
NM 009554	Zfp37	3585	NM 001163583	Prom1	2965	NM 001290468	Hs6st2	4433
NM 021377	Sorcs1	7241	NM 147106	Olfr980	1036	NM 001009949	Slc25a51	4438
NM 001290351	Zfp37	3588	NM 177859	Aknad1	2603	NM 001290502	Ttc14	9572
NM 001290356	Sorcs1	3650	NM 178247	Dppa1	2350	NM 001077202	Hs6st2	4792
NM 001290353	Zfp37	3495	NM 001162911	Zfp934	2235	NM 010336	Lpar1	3362
NM 001252501	Sorcs1	4396	NM 177698	Psd3	11334	NM 015819	Hs6st2	4313
NM 007395	Acvr1b	3277	NM 001206390	Hk3	3029	NM 026167	Klhl13	3251
NM 145629	Pls3	3251	NM 175252	Zfp934	2103	NM 027619	Ttc14	7692
NM 016675	Cldn2	3079	NM 001163358	Dppa1	2264	NM 172989	Lpar1	3451
NM 007400	Adam12	7675	NM 001177772	Slco1c1	3175	NM 001083912	Plekhg2	5067
NM 009813	Casq1	1874	NM 183158	Cyp2ab1	2735	NM 001290572	Ralgps1	6131
NM_178619	1810026J23Rik	3170	NM_001163577	Prom1	3766	NM_001029868	Pdzd4	3991
NM 026500	Ddx59	2228	NM 001177570	Zfp616	4818	NM 001290539	Vstm2a	3507

NM 011705	Vrk1	3796	NM 011050	Pdcd4	2418	NM 138752	Plekhg2	5070
NM 008732	Slc1a2	3041	NM 001172160	Flrt3	4537	NM 029730	Mospd2	4095
NM 001166375	1-Mar	3942	NM 008935	Prom1	3739	NM 027642	Phf6	4420
NM 007628	Ccna1	1581	NM 001168562	Zfp446	4914	NM 025949	Rps6ka6	4683
NM 029092	Trmt10c	1846	NM 001190491	Dmpk	2624	NM 001047604	Ttc21b	4470
NM 020599	Rlbp1	2202	NM 001290181	Nudt7	2839	NM 146148	C8a	3636
NM 021456	Ces1g	2289	NM 148943	Usp9y	8094	NM 172409	Fmnl2	5851
NM 172554	Zdhhc17	4563	NM 023617	Aox3	4360	NM 001166661	Ccdc85a	5340
NM 133784	Wwtr1	4767	NM 173761	Ythdf1	3199	NM 181577	Ccdc85a	5460
NM 001029844	Vrk1	3664	NM 001145919	Ythdf3	5157	NM 018731	Atp4a	3470
NM_001198886	Dpp6	4615	NM_029252	4930563D23Rik	2679	NM_178256	Reps2	7698
NM 022986	Irak1bp1	1893	NM 172677	Ythdf3	5190	NM 001172148	Rbm41	2229
NM 175188	1-Mar	4391	NM 177680	Ythdc1	3034	NM 134471	Kif2c	2840
NM 001146161	Slc1a2	4373	NM 023631	Aox4	4976	NM 133669	Rp2h	4422
NM_207282	Dpp6	4719	NM_001289661	2810474019Rik	6163	NM_018733	Scn1a	8287
NM_010075	Dpp6	4866	NM_001289662	2810474019Rik	6076	NM_001038999	Atp8a1	8176
NM 001029843	Vrk1	3724	NM 027494	Zcchc8	4286	NM 011308	Ncor1	8651
NM 001177307	Aldoa	1597	NM 027857	Acy3	1505	NM 001163017	Gprasp2	3761
NM 134059	Ddx41	2213	NM 019634	Tspan7	1791	NM 172511	Abhd10	2860
NM 172261	Ppp1r9b	4345	NM 145983	Kcna5	3032	NM 001167949	Atp2b4	8292
NM 001198811	Frem1	9524	NM 175836	Sptbn1	8265	NM 001285965	Skint2	1277
NM 021414	Ahcyl2	5218	NM 010587	Itsn1	13976	NM 001110846	Cacna2d1	7400
NM 144879	Vash2	4045	NM 001024726	Zfp607	3511	NM 025972	Naaa	2424
NM 010194	Fes	2780	NM 001110214	Dclre1c	3731	NM 001286155	Zfp322a	5197
NM 001173483	Rlbp1	2138	NM 009214	Sms	3377	NM 001286413	Mtus1	6432
NM 001168240	Irak1bp1	1827	NM 007641	Ms4a1	2057	NM 026061	Ndufb8	641
NM 011798	Xcr1	4453	NM 001005421	Amica1	2472	NM 011707	Vtn	1763
NM 022981	Zfp110	3549	NM 013495	Cpt1a	4331	NM 001286031	Dhx32	2878
NM 007438	Aldoa	1520	NM 053011	Lrp1b	14743	NM 001289720	Ampd2	3458
NM 001136060	Dpp6	4531	NM 027221	Krtcap3	922	NM 001288625	Arid3a	5207
NM 001166372	1-Mar	4223	NM 020333	Slc12a5	6042	NM 177694	Ano5	7783
NM 001167878	Skint5	4434	NM 001082414	Sh3d19	6414	NM 001271708	Lrrn3	3573
NM 145443	L2hgdh	3338	NM 009197	Slc16a2	4180	NM 001285473	Carf	5291
NM 001177308	Aldoa	1470	NM 001039530	Parp14	7251	NM 001285511	Cnot6l	8696
NM 001168281	Wwtr1	4688	NM 080466	Kcnn3	7617	NM 001285463	Carf	5701
NM 001167876	Skint5	4497	NM 025392	Bccip	1260	NM 001252460	Cyfip2	6677
NM 001162425	Efna1	1447	NM 153591	Nars2	3770	NM 009727	Atp8a1	8131
NM 025553	Mrpl11	2882	NM 026246	Mrpl49	1722	NM 175275	Cntln	5542
NM 021359	Itgb6	4788	NM 153534	Adcy2	4211	NM 001289462	Mme	6088
NM 023651	Pex13	4122	NM 010254	Galr2	1957	NM 001289696	Zc4h2	2268
NM 177863	Frem1	9307	NM 007936	Epha4	6328	NM 001284525	Chit1	1851
NM 001162943	Dchs1	10651	NM 001081252	Uggt2	6514	NM 001284345	Atp8a1	8176
NM 001037127	Musk	3422	NM 013826	Mocs2	1889	NM 008538	Marcks	4186
NM 001242379	Slc4a10	5424	NM 133182	Prmt2	2052	NM 001287165	Nup98	4086
NM 020282	Nqo2	3974	NM 001005510	Syne2	21718	NM 009863	Cdc7	2968
NM 133908	Rita1	1668	NM 023514	Mrps9	1445	NM 001164885	Lpin2	5775
NM 029096	Rita1	1586	NM 001077638	Prmt2	2055	NM 001110843	Cacna2d1	7472
NM 010391	H2-Q10	1473	NM 001110276	Itsn1	6154	NM 001271879	Ano5	7633
NM 016778	Bok	1432	NM 001101038	Siglec15	1029	NM 213616	Atp2b4	8101
NM 009270	Sqle	2748	NM 016901	Oaz3	1136	NM 001160292	Celf2	7605
NM 178693	Coq4	1987	NM 001113213	Odf2	3921	NM 001128103	Ano3	6026
NM 001253879	Mtf2	4347	NM 018883	Camkk1	3466	NM 010265	Gcnt1	4580
NM 001164533	Dap3	4538	NM 010469	Hoxd4	2560	NM 001083120	Enah	12280
NM 001164082	Polr3d	2017	NM 009592	Abcb7	5759	NM 001285483	Htr1d	2901
NM 029702	Arfrp1	2590	NM 173012	Letm2	2115	NM 001163239	Nqo2	3804
NM 001167914	Atxn3	1032	NM 010952	Oaz2	1861	NM 023792	Pank1	6719
NM 026612	Ndufb2	455	NM 001008231	Daam2	6045	NM 001289463	Mme	5872
NM 001033245	Hk3	3219	NM 146114	Dclre1c	3759	NM 001003916	Zc4h2	2236
NM 001194922	Cldn18	2849	NM 010690	Lats1	7222	NM 001160293	Celf2	8766

NM 001253878	Mtf2	4424	NM 009782	Cacna1e	12697	NM 001136484	Gcnt1	4539
NM 008428	Kcnj8	2188	NM 011181	Cyth2	2527	NM 001286825	Uspl1	3714
NM 177371	Tnfsf15	5828	NM 023224	Cblc	1677	NM 008365	Il18r1	4158
NM 008225	Hcls1	2031	NM 025961	Gatm	2357	NM 001271588	Eps8	4492
NM 008249	Tfb2m	2448	NM 009260	Sptbn1	9049	NM 028603	Zbtb8a	2229
NM 001195003	Kat7	5966	NM 153176	Spg7	2558	NM 001285874	Pafah2	3136
NM 001252459	Cyfip2	6622	NM 009040	Rdh16	3208	NM 001163242	Nqo2	3821
NM 011783	Agr2	760	NM 053080	Aldh1a3	3423	NM 010135	Enah	12337
NM 025474	Mrps14	1970	NM 001110275	Itsn1	5386	NM 152134	Homer1	4287
NM 001242383	Slc4a10	5421	NM 001098425	Sp3	4100	NM 177327	Wwp1	6458
NM 001164662	Cyfip1	6465	NM 010844	Muc5ac	8505	NM 001083121	Enah	11548
NM 053159	Mrpl3	1531	NM 011224	Pygm	2874	NM 001285482	Htr1d	3079
NM 019815	Cldn18	2842	NM 178691	Yod1	3843	NM 028716	Phf19	3743
NM 146054	Fermt2	3243	NM 008130	Gli3	8428	NM 001287166	Nup98	3930
NM 001163618	Tars2	2281	NM 145452	Rasa1	5101	NM 001287164	Nup98	6705
NM 001001335	Plekha8	6628	NM 001112701	Cyth2	2524	NM 001289442	Grap2	5380
NM 001163241	Nqo2	3892	NM 011992	Rcn2	2042	NM 138648	Olr1	3581
NM 178877	Slc9b2	2243	NM 175485	Prtg	8764	NM 133809	Kmo	2512
NM 175461	Fam78b	4668	NM 138591	Gfm1	2598	NM 001284279	Rnf146	3932
NM 029272	Ndufs7	758	NM 198100	Tbkbp1	3358	NM 153567	Slain2	4865
NM 001168622	Znrf1	5466	NM 009801	Car2	1807	NM 001110844	Cacna2d1	7436
NM 001168623	Znrf1	3650	NM 032002	Nrg4	1952	NM 018776	Crlf3	2396
NM 001195298	Kifc1	2330	NM 001142760	Pcdh15	9135	NM 001286384	Kars	2283
NM 001081105	Rhoh	4960	NM 029353	Malsu1	785	NM 001286030	Dhx32	3009
NM_028732	4632428N05Rik	4846	NM_001142746	Pcdh15	9150	NM_022979	Nup98	4137
NM 029227	Tomm20l	596	NM 001142741	Pcdh15	8974	NM 133788	Icmt	4952
NM 019880	Mtch1	1918	NM 133987	Slc6a8	3992	NM 172987	Nkain3	2906
NM 025784	Bcs1l	1829	NM 015824	Orc3	6313	NM 001286364	Tc2n	3711
NM 144811	Cbx7	2893	NM 008231	Hdgf	2257	NM 001284428	Smtn	3454
NM 138599	Tomm70a	3778	NM 001163538	Pgam5	2079	NM 029320	Pibf1	3297
NM 001165991	Arfrp1	2596	NM 009845	Cd22	3972	NM 001100451	Msl2	4899
NM 001164361	Plekha8	6723	NM 018743	Agpat6	3944	NM 001165992	Arfrp1	2577
NM 001159564	Itgb6	4894	NM 001163578	Prom1	2728	NM 022997	Vps35	3169
NM 029705	Atxn3	5376	NM 133803	Dpp3	2683	NM 011955	Nubp1	2139
NM 011340	Serpinf1	1497	NM 001146212	Mrps10	1035	NM 009183	St8sia4	5437
NM 011699	Lin7c	4137	NM 001043317	Cd22	4097	NM 001166385	Sp4	9011
NM 011529	Tank	2018	NM 001135112	Dnaja3	2549	NM 001285993	Tbc1d5	5583
NM 025876	Cdk5rap1	1970	NM 173745	Dusp18	4632	NM 001286641	AI593442	5684
NM 001253877	Mtf2	4705	NM 023115	Pcdh15	9064	NM 013827	Mtf2	4343
NM 008273	Hoxd11	3959	NM 008569	Anapc1	8967	NM 010368	Gusb	2456
NM 001195004	Kat7	5876	NM 022653	Thop1	2585	NM 001110845	Cacna2d1	7421
NM 028810	Rnd3	2827	NM 133718	Tmem30a	3680	NM 019921	Akap10	3879
NM 013667	Slc22a2	2138	NM 201394	Plec	15003	NM 172339	Snapc4	4381
NM 001253390	Mtfr1	2842	NM 201385	Plec	14856	NM 025508	Gmpr	1554
NM 009785	Cacna2d3	3710	NM 001142738	Pcdh15	8923	NM 001286156	Zfp322a	5122
NM 033552	Slc4a10	5463	NM 001142736	Pcdh15	9043	NM 032000	Trps1	10210
NM 182959	Slc17a8	4452	NM 024248	Cars2	1867	NM 008680	Enah	12292
NM 001242380	Slc4a10	5553	NM 010922	Mrpl40	1055	NM 001285484	Htr1d	2808
NM 011983	Homer2	10999	NM 001037711	Cgn	5039	NM 007945	Eps8	4567
NM 001163619	Tars2	3322	NM 010631	Kifc3	3288	NM 001289697	Zc4h2	2215
NM 001242382	Slc4a10	5511	NM 201390	Plec	14855	NM 001271433	Hagh1	1336
NM 001081231	Lhfpl3	2981	NM 013880	Plcl2	4068	NM 001289740	Mturn	5307
NM 001253391	Mtfr1	3004	NM 001163582	Prom1	2653	NM 009239	Sp4	9017
NM 001253880	Mtf2	4062	NM 133947	Numa1	7214	NM 139150	Carf	5574
NM 001160261	Fam78b	4666	NM 027514	Pvr	2902	NM 053122	Immp2l	1163
NM 009250	Serpini1	3133	NM 133923	Ttll3	3125	NM 009784	Cacna2d1	7415
NM 139299	Il31ra	3680	NM 027134	Mtfmt	2042	NM 001271589	Eps8	3562
NM 001168621	Znrf1	4451	NM 011117	Plec	14921	NM 008044	Fxn	1095
NM 001242381	Slc4a10	5460	NM 028273	Pgam5	2076	NM 001242396	Jmjd1c	8617
NM 001164086	Homer2	10966	NM 001142743	Pcdh15	6338	NM 001289719	Ampd2	3608

NM_001159572	4632428N05Rik	4843	NM_001142737	Pcdh15	8836	NM_001286726	Trim26	3250
NM_001038500	Esp1	1049	NM_001142739	Pcdh15	8983	NM_001286727	Trim26	3199
NM_011370	Cyfip1	6471	NM_001145832	Kifc3	2945	NM_007726	Cnr1	5807
NM_026182	Mtfr1	2852	NM_201393	Plec	14946	NM_152825	Usp45	6018
NM_172382	Kdm4a	3376	NM_001145831	Kifc3	3431	NM_001002004	2610507B11Rik	7554
NM_177229	Ncor1	3186	NM_001142735	Pcdh15	9049	NM_001142411	Zfp937	6920
NM_022994	Dap3	4628	NM_001163584	Prom1	3557	NM_001285426	Cacnb4	7792
NM_021361	Nova1	7000	NM_023646	Dnaja3	2666	NM_146123	Cacnb4	7928
NM_008275	Hoxd13	2483	NM_001162946	Pcx	4148	NM_021460	Lipa	2991
NM_001194921	Cldn18	2774	NM_001146211	Mrps10	1032	NM_018760	Slc4a4	7492
NM_019659	Kcnj1	3073	NM_001142742	Pcdh15	6303	NM_001004194	Nlrp4e	3382
NM_025461	Cox16	1997	NM_001142740	Pcdh15	9034	NM_001285428	Cacnb4	7717
NM_009996	Cyp24a1	3296	NM_001159563	Orc3	6310	NM_026201	Ccar1	4478
NM_001102430	Arfgef1	7042	NM_001163585	Prom1	3602	NM_001136260	Slc4a4	7395
NM_181407	Me3	4370	NM_201391	Plec	15003	NM_022656	Nisch	5606
NM_207221	Jmjd1c	8382	NM_001081340	Setd2	8350	NM_007833	Dcn	1794
NM_001165995	Arfrp1	2483	NM_172737	Shisa7	6058	NM_001289788	Slc26a5	4378
NM_011812	Fbln5	5893	NM_029361	Wnk2	6932	NM_031182	Tfap4	2113
NM_001163617	Tars2	2446	NM_020043	Igdcc4	6227	NM_026030	Eif2s2	2513
NM_001271564	Armc3	2956	NM_001252501	Sorcs1	4396	NM_012054	Aoah	2923
NM_001271444	Ndufs8	1054	NM_145629	Pls3	3251	NM_001244952	Col25a1	7358
NM_008161	Gpx3	1517	NM_016675	Cldn2	3079	NM_001111100	Lipa	2987
NM_019402	Pabpn1	1793	NM_007400	Adam12	7675	NM_001033636	Prex2	6540
NM_001164087	Homer2	10982	NM_009813	Casq1	1874	NM_011352	Sema7a	3290
NM_145960	Mtrf1	1862	NM_010407	Hck	2107	NM_030727	Slc26a5	4616
NM_001271709	Lrrn3	3539	NM_008830	Abcb4	4083	NM_029838	Col25a1	7421
NM_133885	Osbpl9	3342	NM_001025600	Cadm1	4270	NM_008021	Foxm1	4391
NM_177385	Cntln	2441	NM_027862	Atp5h	574	NM_183427	Glra2	2775
NM_010107	Efna1	1483	NM_028232	Sgol1	3609	NM_026192	Calcoco1	2840
NM_001277928	Lamb3	4016	NM_008817	Peg3	6624	NM_001003948	Pid1	2667
NM_001278274	Rcn2	1031	NM_007628	Ccna1	1581	NM_001190451	Dcn	1886
NM_133767	Mtif2	3017	NM_001113354	Phf8	6359	NM_001197147	Slc4a4	7465
NM_001271587	Eps8	4561	NM_020599	Rlbp1	2202	NM_198711	Col25a1	6753
NM_001282120	Mtif2	3103	NM_010345	Grb10	5089	NM_013803	Casr	4549
NM_001271694	Kcnj15	5061	NM_138657	Socs7	7015	NM_008559	Mc1r	3670
NM_001276292	Wwp1	6296	NM_001206369	Gsn	2601	NM_029525	Prex2	11057
NM_133769	Cyfip2	6385	NM_026203	Ahi1	4885	NM_001037099	Cacnb4	8008
NM_001271443	Ndufs8	814	NM_054044	Gpr124	5520	NM_001281854	Aoah	936
NM_001277100	Add3	4511	NM_133784	Wwtr1	4767	NM_183139	Pld6	1769
NM_028052	Synpr	2551	NM_018770	Cadm1	4354	NM_001290010	Ache	2227
NM_001276315	Cdk12	4256	NM_177201	Phf8	3732	NM_175211	Ralgps1	6341
NM_146023	Evi2a-evi2b	3964	NM_001198886	Dpp6	4615	NM_001190886	Kcnip1	2247
NM_001277925	Ecel1	2906	NM_001177879	Phka2	4528	NM_001290707	Pde10a	7763
NM_001271526	Orc2	3555	NM_172783	Phka2	4714	NM_001290685	Shroom2	4826
NM_001271574	Tktl2	6368	NM_001198866	Dctn1	4247	NM_023146	Ranbp17	5002
NM_144870	Ndufs8	1057	NM_026782	Ccdc167	1383	NM_001190885	Kcnip1	2291
NM_001271687	Kcnj15	5210	NM_015735	Ddb1	4171	NM_172441	Shroom2	7519
NM_172797	Mmp28	2269	NM_207282	Dpp6	4719	NM_011866	Pde10a	7717
NM_173442	Gcnt1	4612	NM_011721	Wrn	6383	NM_011855	Tenm1	8363
NM_001271689	Kcnj15	5177	NM_010075	Dpp6	4866	NM_001290686	Shroom2	4633
NM_001271691	Kcnj15	5118	NM_025470	Mptx1	803	NM_029090	Naa60	2439
NM_001283030	Spin1	4254	NM_001122822	Wrn	6273	NM_016965	Nckap1	4403
NM_001271692	Kcnj15	5106	NM_001198811	Frem1	9524	NM_145525	Osbpl6	8085
NM_027931	Tars2	2524	NM_021414	Ahcyl2	5218	NM_013757	Sytl4	3770
NM_146197	Acsm2	6527	NM_144879	Vash2	4045	NM_172699	Foxj3	4808
NM_001271595	Eps8	4566	NM_016699	Exosc10	2803	NM_001113353	Synj2	5886
NM_001013759	Gas2l2	2943	NM_207676	Cadm1	4303	NM_026023	Nudcd2	1599
NM_011462	Spin1	4159	NM_153179	Pkhd1	12928	NM_027398	Kcnip1	2258
NM_010456	Hoxa9	3229	NM_001173483	Rlbp1	2138	NM_139306	Acer2	4206

NM 001177978	Acsm2	6542	NM 001170866	Ncald	3538	NM 172420	Ppp1r1c	3071
NM 001271693	Kcnj15	5073	NM 172551	Polrmt	3762	NM 207228	Tsga10	3607
NM 001286652	Tmem176b	1181	NM 001177878	Phka2	4341	NM 212484	Cnot6	5726
NM 001026211	Dnajc19	1281	NM 001198867	Dctn1	4172	NM 001197024	Unkl	4775
NM 001289509	Tfr2	3597	NM 001136060	Dpp6	4531	NM 001290719	Sytl4	2958
NM 001286007	Tubgcp2	3158	NM 207675	Cadm1	4387	NM 001290744	Ppp1r1c	2975
NM 001160262	Fam78b	4986	NM 134093	Letmd1	2579	NM 001077712	Stag2	5977
NM 001042743	Mast2	5724	NM 001168281	Wwtr1	4688	NM 001113352	Synj2	5751
NM 023114	Apoc3	527	NM 053100	Trim8	3262	NM 001290687	Shroom2	4815
NM 010166	Eya3	5210	NM 025553	Mrpl11	2882	NM 021465	Stag2	5837
NM 001289565	Mpv17l	3106	NM 021359	Itgb6	4788	NM 172979	Muc15	3277
NM 001286013	Dlk2	1544	NM 177863	Frem1	9307	NM 175255	Sec24a	6798
NM 001286049	Entpd5	4962	NM 001162943	Dchs1	10651	NM 009702	Aqr	4897
NM 001282119	Mtif2	2661	NM 001161843	Il18r1	1793	NM 153387	Tubgcp4	4278
NM 007739	Col8a1	5110	NM 001242379	Slc4a10	5424	NM 197997	Zgrf1	7064
NM 009363	Tff2	571	NM 201392	Plec	14829	NM 173733	Suox	2349
NM 001271695	Kcnj15	5020	NM 133254	Slc5a2	2259	NM 001290808	Haus2	3100
NM 001286465	Btrc	6340	NM 025806	Plbd1	1974	NM 025475	Haus2	3170
NM 013727	Azi2	4631	NM 001256112	Mtfr1l	2035	NM 030266	Inpp4a	5748
NM 001284312	Ube2j2	3284	NM 001252396	Slc25a19	2305	NM 030234	Wdr76	4294
NM 001284228	Flot2	2574	NM 001163290	Adck3	3928	NM 001290987	Wdr76	4131
NM 001289755	Apoc3	631	NM 019514	Astn2	4675	NM 008441	Kif1b	7066
NM 015800	Crim1	5995	NM 183086	Mrps10	1125	NM 001005511	Lmtk3	4867
NM 001271690	Kcnj15	5138	NM 001163741	Ccdc167	4625	NM 001290990	Lmtk3	5022
NM 001168354	Kcnj1	3075	NM 013841	Vps45	2643	NM 207682	Kif1b	10118
NM 001289630	Mgme1	3107	NM 010462	Hoxc10	1914	NM 028802	Gpcpd1	3716
NM 001271757	Wnt5b	2174	NM 001081178	Gpr116	8535	NM 025978	Ttc14	9334
NM 001114339	Pank1	7419	NM 001044744	Gcdh	2140	NM 001042672	Gpcpd1	3392
NM 028984	Mgme1	2836	NM 001033245	Hk3	3219	NM 001291060	Lgals8	2664
NM 011802	Clpx	2886	NM 029759	Mtfr1l	1986	NM 008186	Gtf2h1	2749
NM 001044389	Clpx	2844	NM 008428	Kcnj8	2188	NM 019703	Pfkp	4033
NM 001005865	Mtus1	4169	NM 008225	Hcls1	2031	NM 001199043	Lgals8	2996
NM 001271857	Add2	8223	NM 016917	Slc40a1	3380	NM 023502	Elf2	5900
NM 001285797	Zfp27	3801	NM 001195003	Kat7	5966	NM 138651	Cds2	8412
NM 147176	Homer1	4804	NM 001168645	Slc6a18	4037	NM 173754	Usp43	4462
NM 001168492	Pdcd4	1806	NM 201388	Plec	14908	NM 011822	Pigq	3115
NM 001271566	Cdc7	3137	NM 001252459	Cyfip2	6622	NM 001291075	Gtf2h1	2716
NM 028181	Ccpg1	2753	NM 177267	Dcaf5	5707	NM 001013385	Grm4	3231
NM 001081006	Etl4	7413	NM 001039373	Mtcp1	1594	NM 018886	Lgals8	2810
NM 008001	Fgd1	4166	NM 018780	Sfrp5	1900	NM 019553	Ddx21	4763
NM 133690	Atp1b4	4278	NM 001242383	Slc4a10	5421	NM 011302	Rs1	5855
NM 008651	Mybl1	4984	NM 001164662	Cyfip1	6465	NM 212441	Acsm3	2650
NM 001122982	Ccnc	1476	NM 146054	Fermt2	3243	NM 008810	Pdha1	2778
NM 001290392	Sntg1	6999	NM 001205346	Sun2	3803	NM 011127	Prrx1	4911
NM 016746	Ccnc	3391	NM 010551	Il16	4989	NM 011758	Zfp39	3890
NM 001290368	Nmt2	4429	NM 001001335	Plekha8	6628	NM 001030274	Ndufs5	540
NM 001033383	Zfp865	3958	NM 011254	Rbp1	2628	NM 029530	Lamp5	1965
NM 011270	Rhd	1530	NM 001252293	Mest	2401	NM 013904	Hey2	2549
NM 026887	Ap1s2	3327	NM 001168623	Znrf1	3650	NM 145941	Eif4g1	5481
NM 009105	Rsu1	1607	NM 001113379	Lrrc32	3817	NM 001033962	Ube3a	4910
NM_008708	Nmt2	4461	NM_028732	4632428N05Rik	4846	NM_013671	Sod2	3824
NM 001033329	Arhgef9	5086	NM 001204152	C1qtnf6	2277	NM 021419	Rnf8	2046
NM 027671	Sntg1	7267	NM 019880	Mtch1	1918	NM 172696	Inadl	7305
NM 030718	Abo	1795	NM 009760	Bnip3	1756	NM 007760	Crat	4393
NM 172988	Fbxl4	2563	NM 007819	Cyp3a13	2955	NM 027297	Prpf4	5237
NM 016886	Gria3	5213	NM 001206368	Gsn	2556	NM 175686	Prrx1	4983
NM 023739	Nfx1	4556	NM 001168382	Phf14	3381	NM 030721	Acox3	3977
NM 026293	Spaca1	1073	NM 144789	Prdm15	6346	NM 016755	Atp5j	797
NM 020488	Gabrq	2258	NM 207109	Astn2	4831	NM 027869	Pnpt1	2704
NM 001290427	Frmpd4	8223	NM 153744	Prkag3	2835	NM 177566	Arhgef15	4213
NM 146178	Ccdc106	2016	NM 001040692	Slc6a18	4314	NM 025301	Mrpl17	6712

NM 001281929	Gria3	5224	NM 001164361	Plekha8	6723	NM 176982	Fbxo48	2117
NM 033134	Inpp5e	4186	NM 001159564	Itgb6	4894	NM 146141	Ppa2	1225
NM 207670	Gripap1	2928	NM 134156	Actn1	3739	NM 172698	Efcab14	4320
NM 001033330	Frmpd4	8612	NM 201373	Trim56	3637	NM 145426	Mfap3	4864
NM 145355	Rnf185	3508	NM 001168646	Slc6a18	4284	NM 023175	Nit2	1292
NM 001283063	Ewsr1	3216	NM 011699	Lin7c	4137	NM 177364	Sh3pxd2b	7432
NM 001290489	Med27	1002	NM 001252283	Ogdh	6663	NM 172762	Rbm34	3445
NM 172781	Klhl4	3884	NM 130892	Rtn4ip1	2981	NM 175337	Mlh3	5448
NM 001290495	Trub2	3941	NM 194342	Sun2	3713	NM 001031814	Smg1	15553
NM 026896	Med27	1276	NM 008590	Mest	2579	NM 001005331	Eif4g1	5460
NM 001290478	Klhl4	3785	NM 008273	Hoxd11	3959	NM 024262	Smg8	3236
NM 027409	Mospd1	2254	NM 001195004	Kat7	5876	NM 172381	AI314180	5919
NM 178694	Zer1	4280	NM 001159626	Hagh	1101	NM 172287	Spire2	2399
NM 001290502	Ttc14	9572	NM 001252477	Prmt1	1028	NM 001005784	Inadl	3335
NM 010336	Lpar1	3362	NM 027244	Ndufa11	2661	NM 029735	Eprs	4850
NM 145520	Trub2	4003	NM 033552	Slc4a10	5463	NM 008847	Pip5k1a	2553
NM 026167	Klhl13	3251	NM 001242380	Slc4a10	5553	NM 027346	Taco1	1351
NM 001017959	Lamp2	1788	NM 013885	Clic4	4065	NM 008655	Gadd45b	1305
NM 013668	Kdm5c	11030	NM 010888	Ndufs6	526	NM 011286	Rph3a	4143
NM_010685	Lamp2	3615	NM_029472	Gstt4	964	NM_028299	2700029M09Rik	1210
NM 001080924	Znrf3	6306	NM 144946	Neto1	3531	NM 053124	Smarca5	4658
NM 172989	Lpar1	3451	NM 011983	Homer2	10999	NM 212442	Acsm3	2557
NM 001290473	Rnf185	3370	NM 026310	Mrpl18	959	NM 025942	Ola1	2189
NM 138604	Otud5	4259	NM 001242382	Slc4a10	5511	NM 175194	Slc25a16	3141
NM 172496	Cobl	5633	NM 010311	Gnaz	2435	NM 180599	Mfap3	4828
NM 001290539	Vstm2a	3507	NM 001195710	Tmem95	531	NM 144804	Depdc7	1734
NM 001081052	Nhs	5319	NM 145990	Cdk5rap2	5603	NM 178778	Scai	10705
NM 029730	Mospd2	4095	NM 025558	Cyb5b	4294	NM 011668	Ube3a	5097
NM 001163610	Nhsl2	12971	NM 009250	Serpini1	3133	NM 145999	Rhot2	2825
NM 027642	Phf6	4420	NM 001081059	Mcur1	4368	NM 001038607	Kcnh1	7062
NM_001177653	Hist1h2be	2483	NM_001168621	Znrf1	4451	NM_009609	Actg1	1931
NM 153586	Rbm41	5539	NM 001242381	Slc4a10	5460	NM 030091	Ola1	1019
NM 012018	Cntrl	7612	NM 001164086	Homer2	10966	NM 016776	Mybbp1a	4215
NM 001047604	Ttc21b	4470	NM 015730	Chrna4	4509	NM 207636	Fndc3a	6177
NM_001172147	Rbm41	5621	NM_001159572	4632428N05Rik	4843	NM_016870	Acsm3	2777
NM 001100591	Rc3h2	8981	NM 013568	Kcna6	5853	NM 008326	Irgm1	2227
NM 172409	Fmnl2	5851	NM 011370	Cyfip1	6471	NM 001033876	Kcnk9	1209
NM 001290635	Cntrl	1854	NM 172382	Kdm4a	3376	NM 011568	Alyref	1132
NM 018731	Atp4a	3470	NM 010930	Nov	2366	NM 010600	Kcnh1	7143
NM 001290655	Ahsa2	3716	NM 008097	Gcdh	2197	NM 027769	Cpne3	5646
NM 178256	Reps2	7698	NM 021361	Nova1	7000	NM 001038641	Slx4ip	2840
NM 026527	Chac2	1330	NM 153168	Lars2	3881	NM 013676	Supt5	3561
NM 030000	Cntrl	5970	NM 019659	Kcnj1	3073	NM 010833	Msn	3840
NM 177345	Mapkap1	3452	NM 009996	Cyp24a1	3296	NM 023525	Cad	7159
NM 172391	Ahsa2	3697	NM 001161842	Il18r1	1919	NM 028638	Gadl1	3652
NM 133669	Rp2h	4422	NM 001102430	Arfgef1	7042	NM 011468	Sprr2a1	3547
NM_178194	Hist1h2be	2509	NM_181407	Me3	4370	NM_026577	Arl13b	3541
NM 198294	Tanc1	7939	NM 145997	Kdm5a	10969	NM 172417	Perm1	3849
NM 139061	Vps54	4569	NM 023341	Adck3	4026	NM 175201	Rnf38	4989
NM 001194923	Cldn18	2786	NM 201386	Plec	14917	NM 198957	Rbm12b2	3457
NM 001038999	Atp8a1	8176	NM 025695	Smc6	5603	NM 177325	Tsr1	3395
NM 001168491	Pdcd4	2330	NM 001204153	C1qtnf6	2217	NM 001083884	Lypd8	1122
NM 008641	Mast2	5706	NM 011812	Fbln5	5893	NM 011604	Tlr6	2600
NM 172511	Abhd10	2860	NM 033174	Snurf	1971	NM 007451	Slc25a5	1238
NM 001167949	Atp2b4	8292	NM 001252384	Slc25a19	2638	NM 019434	Mcm3ap	6400
NM 001289563	Mpv17l	3052	NM 019402	Pabpn1	1793	NM 007923	Elk4	3671
NM 001285965	Skint2	1277	NM 001164087	Homer2	10982	NM 007889	Dvl3	2953
NM 001164071	Tank	2227	NM 001271760	Adra1a	3814	NM 201644	Ugt1a9	3216

NM 001286058	Entpd5	4889	NM 177385	Cntln	2441	NM 022814	Svep1	11275
NM 001286034	Stx2	2807	NM 025595	Mrpl51	1515	NM 175518	Tmem245	6895
NM 001276710	Agxt	1553	NM 001277296	Adck1	2393	NM 028233	Lrpprc	4638
NM_001044719	D17Wsu92e	3587	NM_134094	Ncald	3728	NM_019840	Pde4b	4371
NM 177694	Ano5	7783	NM 028331	C1qtnf6	2443	NM 001008542	Mxi1	5156
NM 001282118	Mtif2	2739	NM 001010973	Hrh2	2789	NM 009391	Ran	2288
NM 001271708	Lrrn3	3573	NM 133767	Mtif2	3017	NM 001110229	Celf2	7761
NM 001285473	Carf	5291	NM 001177629	Grb10	4764	NM 001081330	Dnah2	13704
NM 001285511	Cnot6l	8696	NM 017478	Copg2	4016	NM 010847	Mxi1	4996
NM 001285463	Carf	5701	NM 001282120	Mtif2	3103	NM 001081149	Kat6a	9126
NM 001271704	Kcns2	5105	NM 001271761	Adra1a	2417	NM 001008543	Mxi1	4843
NM 033564	Mpv17l	3328	NM 026442	Cmc1	1188	NM 001040131	Eif4g2	7646
NM 001271758	Wnt5b	2117	NM 133769	Cyfip2	6385	NM 011339	Cxcl15	2127
NM 001252460	Cyfip2	6677	NM 008420	Kcnb1	11153	NM 001033268	Fam120a	4919
NM 001161823	Kdm4a	4616	NM 028052	Synpr	2551	NM 175473	Fras1	15848
NM 009727	Atp8a1	8131	NM 001271455	Tnip1	2719	NM 007840	Ddx5	3520
NM 138595	Gldc	3792	NM 001276315	Cdk12	4256	NM 030732	Tbl1xr1	8127
NM 210071	Eya3	5171	NM 001168644	Slc6a18	4151	NM 028521	Phospho2	2104
NM 011244	Rarg	2973	NM 001271526	Orc2	3555	NM 033618	Supt16	4491
NM_001289631	Mgme1	2778	NM_001271574	Tktl2	6368	NM_027860	0610010F05Rik	4140
NM 175275	Cntln	5542	NM 001281803	Def8	3877	NM 173778	Sh2d7	2759
NM 001289462	Mme	6088	NM 022723	Scube1	7842	NM 013505	Dsc2	4416
NM 133206	Znrf1	5562	NM 001277992	Stk16	2857	NM 001081430	Naa30	4597
NM 001271859	Add2	8006	NM 173442	Gcnt1	4612	NM 001110228	Celf2	7662
NM 001289675	Fbxo41	6513	NM 013461	Adra1a	4277	NM 029546	Pwp2	3871
NM 001038695	Kdm3a	4830	NM 001206367	Gsn	2640	NM 001081324	Neto2	5646
NM 001284345	Atp8a1	8176	NM 145820	Veph1	6450	NM 027799	Ankrd40	3493
NM 008538	Marcks	4186	NM 146206	Tpcn2	2909	NM 009886	Celsr1	11050
NM 001289511	Tfr2	3455	NM 146197	Acsm2	6527	NM 133239	Crb1	4702
NM_001033279	D17Wsu92e	3812	NM_001252476	Prmt1	1325	NM_007603	Capn6	3579
NM 001164072	Tank	2019	NM 015810	Polg2	1585	NM 008997	Rab11b	6065
NM 009863	Cdc7	2968	NM 013771	Yme1l1	4571	NM 009712	Arsb	3989
NM 001271858	Add2	8220	NM 001177978	Acsm2	6542	NM 025776	Rbm22	2079
NM 001289674	Fbxo41	6519	NM 001252394	Slc25a19	2291	NM 010411	Hdac3	2005
NM 001289567	Mpv17l	3382	NM 001005864	Mtus1	4156	NM 001005863	Mtus1	6554
NM 001271879	Ano5	7633	NM 001160262	Fam78b	4986	NM 001110230	Celf2	7749
NM 213616	Atp2b4	8101	NM 001042743	Mast2	5724	NM 001083126	Lhx6	3309
NM 001289756	Apoc3	601	NM 010166	Eya3	5210	NM 001102474	Skint3	1444
NM 001285798	Zfp27	3755	NM 001277297	Adck1	2205	NM 025279	Hnrnpk	1978
NM 001128103	Ano3	6026	NM 001285498	Tead2	2157	NM 010160	Celf2	8867
NM 010265	Gcnt1	4580	NM 001289565	Mpv17l	3106	NM 053119	Echs1	1491
NM 001083120	Enah	12280	NM 001168643	Slc6a18	4121	NM 007450	Slc25a4	1142
NM 001163239	Nqo2	3804	NM 029404	Phf14	7436	NM 009442	Ttf1	4170
NM 023792	Pank1	6719	NM 001033351	Grin3a	7667	NM 011338	Ccl9	3008
NM 013789	Sergef	1460	NM 001286049	Entpd5	4962	NM 008112	Gdi2	2681
NM 025945	Polr3d	2049	NM 026071	Slc25a19	2475	NM 026375	Ahctf1	8831
NM 001289463	Mme	5872	NM 172500	Syne3	5097	NM 177867	Spata21	2513
NM 010078	Drp2	6983	NM 001282119	Mtif2	2661	NM 172565	Klhl11	2436
NM 001136484	Gcnt1	4539	NM 007739	Col8a1	5110	NM 019702	Hbs1l	2793
NM 001286825	Uspl1	3714	NM 001286255	Nadk2	3590	NM 001042593	Hbs1l	2784
NM 011134	Pon1	1534	NM 001284312	Ube2j2	3284	NM 021022	Abcb11	4899
NM 001286468	Arhgap24	2751	NM 001285845	Paqr7	3922	NM 028044	Cnn3	2003
NM 001271588	Eps8	4492	NM 001284228	Flot2	2574	NM 172268	Nup214	8195
NM 001285874	Pafah2	3136	NM 011243	Rarb	3041	NM 008771	P2rx1	2441
NM 001163242	Nqo2	3821	NM 015800	Crim1	5995	NM 172436	Slc25a12	2924
NM 010135	Enah	12337	NM 001168354	Kcnj1	3075	NM 024212	Rpl4	1422
NM 133839	Mmadhc	1302	NM 001079849	Paip1	4458	NM 001083125	Lhx6	3395
NM 001286685	Lrif1	3644	NM 001287167	Nup98	3961	NM 008475	Krt4	2147
NM 175389	Trmt10a	3935	NM 001271386	Hdac9	8195	NM 001102611	Smyd4	3517

NM 152134	Homer1	4287	NM 001005865	Mtus1	4169	NM 177185	Ubn2	14671
NM 001286844	Etv3	5459	NM 011791	Ash2l	3303	NM 008500	Lhx6	3499
NM 177327	Wwp1	6458	NM 001285847	Paqr7	3811	NM 033074	Tars	2636
NM 001285452	Tmem33	6404	NM 147176	Homer1	4804	NM 009655	Alcam	4979
NM 001286454	Pkd1l3	6576	NM 001168492	Pdcd4	1806	NM 009648	Akap1	3721
NM 001282966	Cd63	999	NM 001285849	Paqr7	3695	NM 001081383	Kmt2c	16820
NM 001083121	Enah	11548	NM 028779	Ampd2	3694	NM 007988	Fasn	9938
NM 001286508	Azi2	4549	NM 001290375	Camk1d	7183	NM 009059	Rgl2	2967
NM 001289442	Grap2	5380	NM 001290374	Camk1d	7108	NM 028918	Ttc25	2818
NM 001278534	Rgag4	4330	NM 133690	Atp1b4	4278	NM 018793	Tyk2	4822
NM 133809	Kmo	2512	NM 001290392	Sntg1	6999	NM 016805	Hnrnpu	3670
NM 001284279	Rnf146	3932	NM 016746	Ccnc	3391	NM 028834	Slx4ip	1437
NM 001286651	Tmem176b	1274	NM 177236	Atp2b3	6748	NM 010326	Gp1ba	2732
NM 009184	Ptk6	2286	NM 001290416	Eps8l1	3868	NM 008255	Hmgcr	4444
NM 018776	Crlf3	2396	NM 026146	Eps8l1	3774	NM 025884	Zfp830	3278
NM 080453	Mmp28	2311	NM 022017	Trpv4	3247	NM 145438	Llgl2	3557
NM 133788	Icmt	4952	NM 177343	Camk1d	7226	NM 023230	Ube2v1	1950
NM 211357	Eya3	4990	NM 001033329	Arhgef9	5086	NM 008300	Hspa4	4657
NM 029320	Pibf1	3297	NM 027671	Sntg1	7267	NM 183031	Gpr183	2942
NM 001100451	Msl2	4899	NM 001290427	Frmpd4	8223	NM 153064	Ndufs2	1606
NM 001165992	Arfrp1	2577	NM 133991	Ftsj1	3425	NM 016777	Nasp	3087
NM 029931	Mllt3	4802	NM 153125	Sec16a	8754	NM 027349	Rbm25	4244
NM 138950	Wdr81	6976	NM 207670	Gripap1	2928	NM 183173	Sowaha	3644
NM 001289562	Mpv17l	3089	NM 001114664	Iqsec2	5995	NM 026041	Rrp15	1228
NM 009183	St8sia4	5437	NM 001033330	Frmpd4	8612	NM 025855	Echdc1	3048
NM 201245	Mprip	8718	NM 001290504	Mid1	3714	NM 028360	Ttc19	3419
NM 001166385	Sp4	9011	NM 148928	Gtf3c5	2805	NM 010324	Got1	2065
NM 001284519	Cmah	9909	NM 146142	Tdrd7	3656	NM 001083587	Tns3	7483
NM 001286641	AI593442	5684	NM 001177648	Fnbp1	4743	NM 008234	Hells	3039
NM 013827	Mtf2	4343	NM 001177649	Fnbp1	4548	NM 175310	Pds5b	7311
NM 010368	Gusb	2456	NM 019406	Fnbp1	1983	NM 207515	Mbnl2	4525
NM 001286079	Nin	6771	NM 001290495	Trub2	3941	NM 001083127	Lhx6	3205
NM 173001	Kdm3a	4857	NM 001290505	Mid1	3938	NM 031179	Sf3b1	6192
NM 032000	Trps1	10210	NM 001177650	Fnbp1	4395	NM 172695	Plaa	6815
NM 008680	Enah	12292	NM 001290506	Mid1	3824	NM 013830	Prpf4b	4482
NM 007945	Eps8	4567	NM 001290468	Hs6st2	4433	NM 001081090	Esf1	3392
NM 001289740	Mturn	5307	NM 001038700	Fnbp1	4545	NM 009819	Ctnna2	4046
NM_007941	Stx2	2937	NM_030207	Sfi1	4025	NM_172616	C330027C09Rik	3974
NM 009239	Sp4	9017	NM 001290502	Ttc14	9572	NM 009779	C3ar1	4299
NM 001284369	Atf2	4140	NM 001077202	Hs6st2	4792	NM 009418	Tpp2	4649
NM 139150	Carf	5574	NM 010797	Mid1	3860	NM 153544	BC030867	2543
NM 008044	Fxn	1095	NM 015819	Hs6st2	4313	NM 007658	Cdc25a	3643
NM 001242396	Jmjd1c	8617	NM 001005475	Iqsec2	5208	NM 145451	Gpx6	1262
NM_001271511	D17Wsu92e	3483	NM_145520	Trub2	4003	NM_152895	Kdm5b	6300
NM 019796	Syncrip	6933	NM 013668	Kdm5c	11030	NM 013491	Clcn1	3545
NM 009374	Tgm3	4144	NM 027619	Ttc14	7692	NM 009530	Atrx	10244
NM 177619	Kat7	5699	NM 001080924	Znrf3	6306	NM 001042541	Akap1	3758
NM_001002004	2610507B11Rik	7554	NM_008824	Pfkfb1	1753	NM_146024	Ankrd40	1468
NM 001271446	Ly6a	982	NM 001252503	Aftph	3860	NM 198653	Iars2	3540
NM 021460	Lipa	2991	NM 138604	Otud5	4259	NM 027975	Fam83d	2280
NM 018760	Slc4a4	7492	NM 001290572	Ralgps1	6131	NM 008710	Nnt	5355
NM 001004194	Nlrp4e	3382	NM 172496	Cobl	5633	NM 009989	Cyct	652
NM 018851	Samhd1	3887	NM 001029868	Pdzd4	3991	NM 008883	Plxna3	6964
NM 001286182	Lox	4494	NM 181411	Aftph	3984	NM 027339	Lypd8	1057
NM_177981	Hap1	3225	NM_001163610	Nhsl2	12971	NM_001100394	4930505A04Rik	944
NM 026201	Ccar1	4478	NM 010832	Msl3	3338	NM 001081395	Amotl1	8951
NM_001271417	Ly6a	987	NM_177576	Sun3	1205	NM_001081183	1110037F02Rik	7115

NM 001139520	Samhd1	4010	NM 001290520	Sun3	1101	NM 009109	Ryr1	15362
NM 010728	Lox	4914	NM 008085	Gapdhs	1476	NM 144826	Utp6	3871
NM 001136260	Slc4a4	7395	NM 153586	Rbm41	5539	NM 001110232	Celf2	7735
NM 001110146	Prg4	1884	NM 001290643	Rp2h	1399	NM 178589	Tnfrsf21	3626
NM 008902	Endou	2404	NM 012018	Cntrl	7612	NM 023480	Fahd1	1408
NM 054043	Msi2	6505	NM 001047604	Ttc21b	4470	NM 025914	Actr6	1703
NM 001168693	Endou	2405	NM 001172147	Rbm41	5621	NM 139227	Atxn7	6870
NM 007833	Dcn	1794	NM 146148	C8a	3636	NM 175549	Robo2	8047
NM 001289788	Slc26a5	4378	NM 172409	Fmnl2	5851	NM 023153	Cwc15	1152
NM 008332	Ifit2	3949	NM 001024707	Lrp3	4007	NM 139300	Mylk	7809
NM 001276684	Arc	3056	NM 001290635	Cntrl	1854	NM 175341	Mbnl2	4579
NM 001199113	Slc29a1	2107	NM 018731	Atp4a	3470	NM 007465	Birc2	3155
NM 010738	Ly6a	971	NM 030000	Cntrl	5970	NM 013507	Eif4g2	7760
NM 001199115	Slc29a1	2046	NM 001172148	Rbm41	2229	NM 028398	Phykpl	1896
NM 001244952	Col25a1	7358	NM 177345	Mapkap1	3452	NM 173033	Tstd2	3726
NM 001111100	Lipa	2987	NM 133669	Rp2h	4422	NM 008685	Nfe2	1793
NM 001271418	Ly6a	966	NM 018733	Scn1a	8287	NM 001015046	Rap1gap2	6410
NM 030727	Slc26a5	4616	NM 198294	Tanc1	7939	NM 001039939	Asxl1	6674
NM 001271419	Ly6a	956	NM 001038999	Atp8a1	8176	NM 146155	Ahdc1	6602
NM 021400	Prg4	4278	NM 011308	Ncor1	8651	NM 001110231	Celf2	9108
NM 001195632	Arhgap32	10633	NM 001168491	Pdcd4	2330	NM 001081321	Pds5a	4146
NM 177379	Arhgap32	9969	NM 008641	Mast2	5706	NM 177293	Mtap7d3	2795
NM 029838	Col25a1	7421	NM 001163017	Gprasp2	3761	NM 183221	Fat4	16109
NM 001286181	Lox	4537	NM 001289563	Mpv17l	3052	NM 009797	Capza1	3187
NM 008021	Foxm1	4391	NM 001136087	Slc6a18	4200	NM 001109764	Ctnna2	4148
NM 001199116	Slc29a1	2006	NM 001110846	Cacna2d1	7400	NM 021523	Huwe1	14637
NM 001003948	Pid1	2667	NM 001286155	Zfp322a	5197	NM 027123	Fastkd3	2362
NM 001271416	Ly6a	1002	NM 001286413	Mtus1	6432	NM 030256	Bcl9l	5341
NM 001190451	Dcn	1886	NM 001286058	Entpd5	4889	NM 134012	Mbtd1	5231
NM 010404	Hap1	3774	NM 025400	Nat9	1130	NM 001142950	Nars	2663
NM 001159889	Ociad1	3344	NM 001286034	Stx2	2807	NM 001122738	Kansl1l	4655
NM 001197147	Slc4a4	7465	NM 175276	Fhod3	6251	NM 011247	Rbbp6	6199
NM 198711	Col25a1	6753	NM 001289720	Ampd2	3458	NM 001141982	Rbm43	1991
NM_022880	Slc29a1	2047	NM_001044719	D17Wsu92e	3587	NM_009380	Thrb	6083
NM 018790	Arc	3059	NM 177694	Ano5	7783	NM 001114541	Pced1a	2918
NM_001199114	Slc29a1	1946	NM_028075	Tnfrsf13c	1906	NM_001134459	Prickle2	7878
NM 029525	Prex2	11057	NM 001282118	Mtif2	2739	NM 001128607	Epb4.1	5067
NM 183139	Pld6	1769	NM 001285473	Carf	5291	NM 008777	Pah	2152
NM 001290772	Tro	3242	NM 181848	Optn	2413	NM 019927	Arih1	6435
NM 001290707	Pde10a	7763	NM 001285463	Carf	5701	NM 153539	Brinp3	3118
NM 001290685	Shroom2	4826	NM 001271704	Kcns2	5105	NM 001163642	Setdb1	4641
NM 023146	Ranbp17	5002	NM 033564	Mpv17l	3328	NM 001164035	Ntf3	1368
NM 001166437	Tbc1d25	2452	NM 001287388	Tal1	4254	NM 010023	Eci1	1091
NM 001099641	Gabra6	2505	NM 001252460	Cyfip2	6677	NM 007464	Birc3	2820
NM 172441	Shroom2	7519	NM 001161823	Kdm4a	4616	NM 001039104	Trpm1	5711
NM 011514	Suv39h1	3116	NM 009727	Atp8a1	8131	NM 023731	Ccdc86	3003
NM 019548	Tro	3859	NM 210071	Eya3	5171	NM 001141981	Rbm43	2072
NM 011866	Pde10a	7717	NM 175275	Cntln	5542	NM 023336	Brd3	5429
NM 011855	Tenm1	8363	NM 001286422	Fut10	3729	NM 017480	Icos	3272
NM 001290686	Shroom2	4633	NM 133206	Znrf1	5562	NM 001164034	Ntf3	1453
NM 016965	Nckap1	4403	NM 001080793	Ash2l	3213	NM 009515	Was	2094
NM 007740	Col9a1	3888	NM 001284345	Atp8a1	8176	NM 013889	Zfp292	9977
NM_001290699	Zfp451	3792	NM_001033279	D17Wsu92e	3812	NM_001113573	Brd3	5269
NM 133817	Zfp451	4030	NM 001286038	Klc3	1944	NM 177645	Kansl1l	4628
NM 145525	Osbpl6	8085	NM 026884	Fam57b	1894	NM 011961	Plod2	3656
NM 025667	Tmem222	1497	NM 001278219	Displ	4902	NM 178617	Necab1	4936
NM 175190	Swi5	780	NM 013896	Timm9	1035	NM 027478	Tmem230	1606
NM 139306	Acer2	4206	NM 001272028	Arvcf	4587	NM 018752	Trpm1	2928
NM 001002272	Tro	7204	NM 001289567	Mpv17l	3382	NM 146001	Hip1	7883

NM 212484	Cnot6	5726	NM 001110843	Cacna2d1	7472	NM 001113574	Brd3	5320
NM 001290770	Tro	7153	NM 001271879	Ano5	7633	NM 011968	Psma6	997
NM 001080969	Thg1l	3797	NM 022320	Gpr35	4233	NM 001159948	Als2	6681
NM 008068	Gabra6	2475	NM 001285798	Zfp27	3755	NM 027350	Nars	2660
NM 001077712	Stag2	5977	NM 001128103	Ano3	6026	NM 008287	Hrsp12	1011
NM 053123	Smarca1	4055	NM 010265	Gcnt1	4580	NM 011588	Trim28	3255
NM 001290687	Shroom2	4815	NM 001083120	Enah	12280	NM 001145959	Ndrg2	2115
NM 021465	Stag2	5837	NM 001161844	Cblc	1530	NM 028226	Rbm12b1	3313
NM 172979	Muc15	3277	NM 027186	Rpain	1213	NM 026849	Mtmr14	2676
NM 019790	Tmeff2	3339	NM 010078	Drp2	6983	NM 008133	Glud1	3158
NM 173870	Mgat4a	7251	NM 001136484	Gcnt1	4539	NM 001114140	Tcf20	7364
NM 001033298	Kiz	2150	NM 001286825	Uspl1	3714	NM 173748	Nudcd3	3977
NM 001290784	Ldlrad3	3907	NM 008365	Il18r1	4158	NM 183428	Epb4.1	6565
NM 009702	Aqr	4897	NM 001288613	Atg9a	4104	NM 153553	Npas4	3292
NM 001100454	Wfikkn1	2536	NM 001278220	Displ	4785	NM 001113417	Thrb	5997
NM 178886	Ldlrad3	3910	NM 001285874	Pafah2	3136	NM 178645	Blmh	2355
NM 027917	Shroom1	3312	NM 010135	Enah	12337	NM 013836	Tcf20	7241
NM 001290992	Ctdspl2	6733	NM 001286685	Lrif1	3644	NM 019975	Hacl1	2618
NM 030234	Wdr76	4294	NM 152134	Homer1	4287	NM 178654	Pkn2	6207
NM 053168	Trim11	2279	NM 001286454	Pkd1l3	6576	NM 024475	Ublcp1	2155
NM 010863	Myo1b	4966	NM 029104	Mss51	1829	NM 023603	Sfpq	3500
NM 212450	Ctdspl2	6737	NM 001083121	Enah	11548	NM 211355	Smek1	4555
NM 001290987	Wdr76	4131	NM 001287166	Nup98	3930	NM 001128606	Epb4.1	5141
NM 001161817	Myo1b	5053	NM 001289654	Fhod3	5798	NM 013864	Ndrg2	2157
NM 175154	Galk2	2629	NM 001278534	Rgag4	4330	NM 001145807	Brinp3	2760
NM 025978	Ttc14	9334	NM 001024853	Timm9	997	NM 024188	Oxct1	3496
NM 198214	Snph	4750	NM 001289595	Slc25a40	2766	NM 029936	Ddx10	3368
NM 175193	Golim4	4467	NM 009226	Snrpd1	837	NM 019573	Wwox	2251
NM 001291060	Lgals8	2664	NM 009184	Ptk6	2286	NM 025729	Tab3	6381
NM 001291043	Tmem220	1593	NM 001110844	Cacna2d1	7436	NM 018877	Setdb1	4644
NM 001199043	Lgals8	2996	NM 001289760	Rarb	3158	NM 030243	Rbm43	1965
NM 138651	Cds2	8412	NM 018776	Crlf3	2396	NM 033565	Aff4	10165
NM 173754	Usp43	4462	NM 178766	Slc25a40	2838	NM 008892	Pola1	5350
NM 007403	Adam8	3034	NM 001285932	Dhx34	4076	NM 008926	Prkg2	4696
NM 018886	Lgals8	2810	NM 133788	Icmt	4952	NM 178762	Pced1a	3505
NM 025676	Mcm8	3397	NM 001289757	Rnf32	1606	NM 011915	Wif1	2242
NM 177392	Tmem220	1669	NM 211357	Eya3	4990	NM 010022	Dbt	3292
NM 013910	Kdm2b	3541	NM 001284428	Smtn	3454	NM 134034	Smek2	5111
NM 026295	Ctdp1	3700	NM 029320	Pibf1	3297	NM 011714	Baz1b	6446
NM 175471	Cyb5r1	1638	NM 001286203	Timm9	899	NM 013762	Rpl3	1361
NM 011302	Rs1	5855	NM 001100451	Msl2	4899	NM 025912	Fam210b	3609
NM 008810	Pdha1	2778	NM 001278218	Displ	4942	NM 028132	Pgm2	2334
NM 023231	Stoml2	1565	NM 172469	Clic6	3743	NM 138600	Aldh7a1	2911
NM 011758	Zfp39	3890	NM 011527	Tal1	4237	NM 001163641	Setdb1	4682
NM 001030274	Ndufs5	540	NM 138950	Wdr81	6976	NM 029924	Mbd5	7055
NM 177618	Wscd1	2761	NM 001286207	Ash2l	3186	NM 053099	Setbp1	9981
NM 170669	Rps15a	5158	NM 001289562	Mpv17l	3089	NM 001291115	Rbm39	2598
NM 001033962	Ube3a	4910	NM 201245	Mprip	8718	NM 008180	Gss	1978
NM 013671	Sod2	3824	NM 001166385	Sp4	9011	NM 133242	Rbm39	2821
NM 026313	Luc7l3	3327	NM 001083118	Terf2	2477	NM 023355	Timm22	2641
NM 030717	Lactb	2031	NM 001286641	AI593442	5684	NM 001109906	Stau1	3625
NM 027297	Prpf4	5237	NM 001289761	Rarb	3077	NM 019818	Timm22	2838
NM 026154	Mrpl10	1682	NM 001282095	Tjp3	2941	NM 001291151	Ptprt	12126
NM 145131	Pitrm1	3547	NM 001286039	Klc3	1820	NM 001109905	Stau1	3607
NM 030721	Acox3	3977	NM 001286079	Nin	6771	NM 021464	Ptprt	12099
NM 010880	Ncl	8257	NM 001110845	Cacna2d1	7421	NM 011490	Stau1	3601
NM 177566	Arhgef15	4213	NM 001252288	Ogdh	6678	NM 001291197	Tpd52l2	3625
NM 025301	Mrpl17	6712	NM 001285805	Zbtb20	3209	NM 001291201	Tpd52l2	3565
NM 172698	Efcab14	4320	NM 001289762	Rarb	2687	NM 001291204	Tpd52l2	3371
NM 145426	Mfap3	4864	NM 001286156	Zfp322a	5122	NM 025482	Tpd52l2	3622
NM 177364	Sh3pxd2b	7432	NM 032000	Trps1	10210	NM 019765	Clip1	5877
NM 172762	Rbm34	3445	NM 001042699	Syne3	5452	NM 198656	Cdh26	3022

NM 001031814	Smg1	15553	NM 008680	Enah	12292	NM 026624	Fam166a	1130
NM 175244	Hectd3	4582	NM 001289740	Mturn	5307	NM 008746	Ntrk3	2978
NM 001002764	Smg6	5817	NM 007941	Stx2	2937	NM 001141971	Tmem230	1546
NM 001005866	Kdm2b	5026	NM 009239	Sp4	9017	NM 016978	Oat	2155
NM 001003953	Kdm2b	5184	NM 001205345	Sun2	3809	NM 029600	Abcc3	5002
NM 172287	Spire2	2399	NM 001284369	Atf2	4140	NM 001159301	Lgals9	1493
NM 029735	Eprs	4850	NM 139150	Carf	5574	NM 001081103	Stim2	4930
NM 001039084	Mrpl1	1579	NM 001289713	Cpvl	1622	NM 001145936	Zfp691	1585
NM 001025102	Arl14ep	3309	NM 009784	Cacna2d1	7415	NM 010491	Iapp	725
NM 011286	Rph3a	4143	NM 001277076	Fer1l5	6318	NM 001037905	Dab2	3866
NM 053124	Smarca5	4658	NM 001286944	Jund	2894	NM 001164466	Dpys	2110
NM 008017	Smc2	5337	NM 001285840	Sybu	3054	NM 009873	Cdk6	2470
NM 025942	Ola1	2189	NM 001289719	Ampd2	3608	NM 029037	Pomk	3601
NM_175451	Ckap4	2915	NM_001271511	D17Wsu92e	3483	NM_139206	Arap3	5266
NM 173750	Arl14ep	3263	NM 001285846	Paqr7	3848	NM 023140	Glrx3	1651
NM 152824	Rbm17	1583	NM 001009573	Unc13d	3967	NM 001190343	Ccm2	1701
NM 145139	Eif3l	1893	NM 009374	Tgm3	4144	NM 011794	Bpnt1	2182
NM 175194	Slc25a16	3141	NM 001285931	Dhx34	4310	NM 198703	Wnk1	10575
NM 144903	Aldob	1993	NM 001111141	Sprtn	2116	NM 010907	Nfkbia	1592
NM 172404	Ccbl1	1944	NM 001289655	Fhod3	6155	NM 175094	Pdhx	2527
NM 180599	Mfap3	4828	NM 177619	Kat7	5699	NM 023116	Cacnb2	3945
NM 029409	Mff	1920	NM 001024854	Timm9	813	NM 028099	Dusp11	6445
NM_026515	2810417H13Rik	2387	NM_001166382	Rad52	1695	NM_028903	Scara5	3809
NM 178778	Scai	10705	NM 001166383	Rad52	1626	NM 183140	Zfp691	2338
NM 011668	Ube3a	5097	NM 021460	Lipa	2991	NM 133648	Slc12a6	5970
NM 146101	Habp2	2119	NM 018760	Slc4a4	7492	NM 001002896	Bfsp2	1570
NM 145999	Rhot2	2825	NM 018873	Srcin1	6965	NM 178761	Zfp672	2789
NM 207636	Fndc3a	6177	NM 017464	Nedd9	4384	NM 008742	Ntf3	1342
NM 080435	Adcy4	3414	NM 001166381	Rad52	1698	NM 001242358	Trnt1	2327
NM 007520	Bach1	5858	NM 173379	Leprel1	2248	NM 001190344	Ccm2	1760
NM 010091	Dvl1	3365	NM 026201	Ccar1	4478	NM 001102400	Dab2	3803
NM 009818	Ctnna1	3713	NM 001136260	Slc4a4	7395	NM 001205336	Arap3	5425
NM 212468	Ssbp1	1489	NM 008902	Endou	2404	NM 019825	Ncoa6	6872
NM 027769	Cpne3	5646	NM 022656	Nisch	5606	NM 145537	Edem2	2294
NM 001001184	Primpol	3813	NM 011236	Rad52	1695	NM 011327	Scp2	2664
NM 028358	Ssbp1	1063	NM 007833	Dcn	1794	NM 145567	Hibadh	1748
NM 001038641	Slx4ip	2840	NM 033622	Tnfsf13b	1710	NM 007728	Coch	2683
NM 010833	Msn	3840	NM 008332	Ifit2	3949	NM 001008702	Dab2	3792
NM_025588	Exoc2	4277	NM_001289924	2010111I01Rik	3843	NM_027357	Psmd1	3256
NM 019758	Mtch2	2420	NM 012054	Aoah	2923	NM 022722	Dpys	2471
NM 008986	Ptrf	3218	NM 001111100	Lipa	2987	NM 001177793	Snap23	2145
NM 016714	Nup50	4766	NM 001039537	Lif	4114	NM 001198835	Coch	2484
NM 172843	Tor1aip2	5857	NM 011352	Sema7a	3290	NM 031199	Tgfa	4229
NM 028638	Gadl1	3652	NM 172719	Gcn1l1	8546	NM 001142804	Acss3	2130
NM 001289430	Cipc	4169	NM 008021	Foxm1	4391	NM 001033422	Thoc2	7631
NM 026252	Cpeb4	7655	NM 183427	Glra2	2775	NM 001145935	Zfp691	1721
NM 001289432	Cipc	4217	NM 001190451	Dcn	1886	NM 010917	Nid1	6046
NM 001289431	Cipc	4343	NM 001197147	Slc4a4	7465	NM 010708	Lgals9	1586
NM 173735	Cipc	4116	NM 029525	Prex2	11057	NM 028679	Irak3	2969
NM 001290676	Cpeb4	7631	NM 001111324	Nedd9	4603	NM 027963	Wdr16	2201
NM 009205	Slc3a1	2307	NM 001033431	Nlrp12	4329	NM 001185020	Wnk1	9828
NM 001290678	Cpeb4	7580	NM 175211	Ralgps1	6341	NM 001256516	Zfp672	2950
NM 172417	Perm1	3849	NM 001190886	Kcnip1	2247	NM 001171680	Myt1	5495
NM 001289429	Cipc	4270	NM 011856	Tenm2	9749	NM 001177792	Snap23	2223
NM 007608	Car5a	1248	NM 001290772	Tro	3242	NM 009222	Snap23	2190
NM 013581	Cog1	3060	NM 001199696	Bai2	5112	NM 138670	Mpst	1426
NM 177325	Tsr1	3395	NM 011398	Slc25a14	1764	NM 177306	Rfx6	3088
NM 001085407	Sdccag3	2045	NM 001290707	Pde10a	7763	NM 027275	Ptcd3	2601
NM 027238	Ttc39b	8764	NM 026721	Slc39a13	2377	NM 001252313	Ncor1	8852

NM 001085408	Sdccag3	1976	NM 001190885	Kcnip1	2291	NM 001198998	Vdac3	1423
NM 019434	Mcm3ap	6400	NM 001099641	Gabra6	2505	NM 146014	Ccm2	1875
NM 026737	Phf5a	1648	NM 172441	Shroom2	7519	NM 001185021	Wnk1	10029
NM 008944	Psma2	963	NM 011514	Suv39h1	3116	NM 001162492	Mpst	1322
NM 201644	Ugt1a9	3216	NM 019548	Tro	3859	NM 027296	Trnt1	2262
NM 011313	S100a6	719	NM 011866	Pde10a	7717	NM 010028	Ddx3x	4571
NM 175518	Tmem245	6895	NM 011855	Tenm1	8363	NM 019410	Pfn2	2020
NM 028233	Lrpprc	4638	NM 001290731	Hoxd8	1683	NM 138944	Pou4f2	3212
NM 009391	Ran	2288	NM 016965	Nckap1	4403	NM 023118	Dab2	4520
NM 027470	Pak4	2898	NM 145224	Tbx22	1630	NM 024175	Rps23	572
NM 001081330	Dnah2	13704	NM 001290699	Zfp451	3792	NM 011317	Khdrbs1	3762
NM 001034878	Dnaic2	2871	NM 133817	Zfp451	4030	NM 001252532	Llgl2	3629
NM 025959	Psmc6	1564	NM 001290715	Bai2	5011	NM 009878	Cdkn2d	1271
NM 001081149	Kat6a	9126	NM 181319	Tbx22	1734	NM 029704	Ttc19	3397
NM 025835	Pccb	2291	NM 172699	Foxj3	4808	NM 010301	Gna11	3262
NM 001110311	Snx12	1124	NM 027398	Kcnip1	2258	NM 011696	Vdac3	1420
NM 001081387	Ctcfl	3217	NM 172420	Ppp1r1c	3071	NM 133649	Slc12a6	6631
NM 001040131	Eif4g2	7646	NM 173071	Bai2	5241	NM 019582	Cacna1f	6075
NM 011339	Cxcl15	2127	NM 001290702	Tenm2	9656	NM 001160214	Smek1	4594
NM 001033268	Fam120a	4919	NM 001002272	Tro	7204	NM 134469	Fdps	1262
NM 175473	Fras1	15848	NM 212484	Cnot6	5726	NM 001162493	Mpst	1292
NM 175181	Prr5l	4245	NM 008276	Hoxd8	2055	NM 008552	Mas1	2475
NM 030732	Tbl1xr1	8127	NM 001290770	Tro	7153	NM 177710	Ssh2	9040
NM 033618	Supt16	4491	NM 001080969	Thg1l	3797	NM 175531	Mrgprb2	2089
NM 019833	Fam69b	1636	NM 001166450	Slc25a14	1677	NM 001033276	Kmt2d	19827
NM 010441	Hmga2	4226	NM 008068	Gabra6	2475	NM 178665	Lpp	15669
NM 173778	Sh2d7	2759	NM 001290744	Ppp1r1c	2975	NM 011817	Gadd45g	1081
NM 013505	Dsc2	4416	NM 001077712	Stag2	5977	NM 021881	Qk	6718
NM 001083810	Prr5l	4296	NM 053123	Smarca1	4055	NM 001166585	Tead1	9480
NM 172859	Dzank1	6831	NM 011644	Xntrpc	4506	NM 134000	Traf3ip2	2867
NM 009031	Rbbp7	2272	NM 021465	Stag2	5837	NM 001168521	Sarm1	5164
NM 133231	Rfxap	2185	NM 001290705	Slc25a14	1432	NM 024236	Qdpr	1394
NM 001081430	Naa30	4597	NM 023536	Mrto4	1239	NM 019408	Nfkb2	3205
NM 001081269	Whsc1l1	9945	NM 019790	Tmeff2	3339	NM 001165941	Nsun6	3042
NM 010197	Fgf1	3909	NM 001290783	Wdr13	4199	NM 146921	Olfr1	1248
NM 029546	Pwp2	3871	NM 001103178	Ablim1	5942	NM 027216	Slc39a11	2721
NM 177652	Ryr3	15410	NM 020492	Glra1	2389	NM 028108	Naa50	4526
NM 177195	Atp8b5	4474	NM 173870	Mgat4a	7251	NM 134129	Prpf19	6161
NM 009709	Arnt	4325	NM 001103177	Ablim1	6400	NM 172685	Slc25a24	3383
NM 013559	Hsph1	3478	NM 175255	Sec24a	6798	NM 172795	Sarm1	5044
NM 001081324	Neto2	5646	NM 178688	Ablim1	6638	NM 011630	Nr2c2	7637
NM 027799	Ankrd40	3493	NM 130863	Adrbk1	3451	NM 198932	Pou2f1	12906
NM 009886	Celsr1	11050	NM 001290826	Cpeb3	6259	NM 021473	Akr1a1	1435
NM 133239	Crb1	4702	NM 197997	Zgrf1	7064	NM 001177982	Pde4b	3482
NM 007603	Capn6	3579	NM 026137	Wdr13	4233	NM 029197	Meiob	1703
NM 001081123	Arhgap36	3039	NM 198300	Cpeb3	5970	NM 033218	Srebf2	4570
NM 175113	Trmt6	2820	NM 008443	Kif3a	5450	NM 033370	Copb1	3315
NM 001024622	Pcnp	2300	NM 001290813	Ablim1	6245	NM 021714	Wbp11	2722
NM 172560	Cntrob	3950	NM 001290806	Kif3a	5429	NM 001039143	Nlrp5	3463
NM 001102474	Skint3	1444	NM 001290820	Zscan29	2409	NM 178082	Insig2	2475
NM 011917	Xrn2	3383	NM 001290992	Ctdspl2	6733	NM 198007	Ascc3	7477
NM 001045520	Clint1	3398	NM 030234	Wdr76	4294	NM 001042527	Blm	4598
NM 201358	Lyrm4	1515	NM 033042	Tnfrsf25	1611	NM 001160016	Gnb1	3140
NM 021521	Med12	8464	NM 010863	Myo1b	4966	NM 009124	Atxn1	10599
NM 009442	Ttf1	4170	NM 212450	Ctdspl2	6737	NM 008364	Il1rap	4576
NM 011338	Ccl9	3008	NM 133728	Asnsd1	2450	NM 001171616	Myt1	5415
NM 026563	Sdccag3	2195	NM 001290987	Wdr76	4131	NM 177578	Skint3	3627
NM 172565	Klhl11	2436	NM 023051	Clstn1	4505	NM 016895	Ak2	1781
NM 019702	Hbs1l	2793	NM 001161817	Myo1b	5053	NM 001256519	Zfp672	3130
NM 001042593	Hbs1l	2784	NM 207682	Kif1b	10118	NM 001145952	Lpp	15462
NM 021022	Abcb11	4899	NM 028802	Gpcpd1	3716	NM 008825	Pfkfb2	7308
NM 010722	Lmnb2	3389	NM 025978	Ttc14	9334	NM 001170985	Kirrel	7287

NM 172268	Nup214	8195	NM 011428	Snap25	2135	NM 010403	Hao1	2029
NM 181734	Ttpal	4548	NM 001291053	Ypel1	2599	NM 001159647	Cntn1	5817
NM 009096	Rps6	1394	NM 138651	Cds2	8412	NM 001136085	Uba1	4073
NM 001081158	Cluh	5399	NM 001291047	Ypel1	3005	NM 001033966	Ak2	1308
NM 024177	Mrpl38	1411	NM 173754	Usp43	4462	NM 001164681	Ces3a	1908
NM 001102611	Smyd4	3517	NM 009548	Rnf112	3098	NM 019710	Smc1a	3980
NM 001037737	Arnt	4370	NM 001291024	Rnf112	3029	NM 011386	Skil	7066
NM 177185	Ubn2	14671	NM 013910	Kdm2b	3541	NM 001204229	Clec16a	6238
NM 025830	Wwp2	4319	NM 027181	Pin4	478	NM 001163016	Gprasp2	3776
NM 001081383	Kmt2c	16820	NM 027130	Afg3l2	3086	NM 013903	Mmp20	3276
NM 007988	Fasn	9938	NM 026295	Ctdp1	3700	NM 001199084	Wnk1	11346
NM 030026	Mccc2	2146	NM 011302	Rs1	5855	NM 153526	Insig1	2667
NM 010515	Igf2r	8948	NM 028276	Utp14a	2431	NM 175475	Cyp26b1	4751
NM_026057	Zfp422	3165	NM_177320	Pik3r5	4363	NM_172679	4932438A13Rik	15883
NM 028834	Slx4ip	1437	NM 023231	Stoml2	1565	NM 011294	Sub1	3321
NM 199019	Sppl2c	2225	NM 028479	Mrgbp	1192	NM 021531	Carm1	3231
NM 153796	Peo1	3515	NM 013904	Hey2	2549	NM 010897	Nf1	11847
NM 001079694	Srsf5	1536	NM 001025570	Prrx1	4098	NM 021280	Plcg1	4435
NM 023230	Ube2v1	1950	NM 170669	Rps15a	5158	NM 001199304	Atxn1	10474
NM 008300	Hspa4	4657	NM 145941	Eif4g1	5481	NM 011715	Wdr1	2878
NM 009408	Top1	3859	NM 026313	Luc7l3	3327	NM 198672	Ces3a	2049
NM 183031	Gpr183	2942	NM 172696	Inadl	7305	NM 023144	Nono	2468
NM 010726	Phyh	1474	NM 030717	Lactb	2031	NM 009062	Rgs4	2952
NM 016777	Nasp	3087	NM 053177	Mcoln1	2039	NM 001030307	Dkc1	2803
NM 172862	Frem2	12368	NM 145131	Pitrm1	3547	NM 144958	Eif4a1	1897
NM 028994	Pck2	3400	NM 010880	Ncl	8257	NM 001199083	Wnk1	11319
NM 183173	Sowaha	3644	NM 001039201	Hdhd2	2357	NM 022028	Sav1	2524
NM 001008502	Bbs12	2463	NM 012065	Pde6g	899	NM 026197	Mettl16	2718
NM 009298	Surf6	2593	NM 008925	Prkcsh	2030	NM 019826	Ivd	2067
NM 025855	Echdc1	3048	NM 008385	Inpp5b	3826	NM 023737	Ehhadh	3010
NM 145395	Duoxa1	1513	NM 172698	Efcab14	4320	NM 001177981	Pde4b	3746
NM 001079695	Srsf5	1662	NM 199022	Shc4	4820	NM 001271531	Insig2	2468
NM 172709	Otop1	3171	NM 177364	Sh3pxd2b	7432	NM 008665	Myt1	5529
NM 001081216	Phip	10729	NM 172762	Rbm34	3445	NM 025716	Spryd4	1570
NM 001083587	Tns3	7483	NM 001031814	Smg1	15553	NM 013930	Aass	3701
NM 026497	Nudt12	2280	NM 133191	Eps8l2	3079	NM 007727	Cntn1	5720
NM 146145	Jak1	5299	NM 175244	Hectd3	4582	NM 001159375	Eif4a1	1886
NM 001040435	Tacc3	2637	NM 001005331	Eif4g1	5460	NM 029049	Ptchd3	3238
NM 175310	Pds5b	7311	NM 001002764	Smg6	5817	NM 001163032	Synpr	2657
NM 021506	Sh3rf1	5199	NM 177565	Zfp3	1838	NM 025478	Isoc1	2563
NM 026035	Mrpl55	780	NM 024262	Smg8	3236	NM 172923	AI118078	2407
NM 021718	Ms4a4b	1282	NM 029814	Chmp5	1372	NM 028866	Wdr33	6206
NM 027977	Apmap	2190	NM 001005866	Kdm2b	5026	NM 144521	Snap47	1837
NM 011172	Prodh	2283	NM 001003953	Kdm2b	5184	NM 001167898	Frem3	6626
NM 010730	Anxa1	1395	NM 183020	Atxn2l	3764	NM 008073	Gabrg2	3935
NM 007792	Csrp2	889	NM 026944	Alkbh3	1247	NM 030686	Dhrs4	962
NM 026998	Snx6	1879	NM 001005784	Inadl	3335	NM 027034	Izumo3	1263
NM 172695	Plaa	6815	NM 029735	Eprs	4850	NM 001276317	Uba1	4068
NM 008670	Naip1	5361	NM 133738	Antxr2	3776	NM 008142	Gnb1	3143
NM 007432	Akp3	1680	NM 025310	Ftsj3	2865	NM 001276316	Uba1	4165
NM 009819	Ctnna2	4046	NM 053124	Smarca5	4658	NM 172453	Pif1	3680
NM 013825	Ly75	5172	NM 025942	Ola1	2189	NM 020520	Slc25a20	1783
NM 025655	Tmigd1	1366	NM 153516	Bcl2l13	6933	NM 001164528	Ildr2	8260
NM_172616	C330027C09Rik	3974	NM_175451	Ckap4	2915	NM_175510	Unc80	13533
NM 009779	C3ar1	4299	NM 029826	Hdhd2	2318	NM 172815	Rspo2	3340
NM 009418	Tpp2	4649	NM 175194	Slc25a16	3141	NM 178634	Csrnp3	10510
NM 025974	Rpl14	935	NM 029004	Rasgef1c	2248	NM 001271532	Insig2	2321
NM 007658	Cdc25a	3643	NM 172521	Nutm1	3731	NM 023612	Esm1	2163
NM 145451	Gpx6	1262	NM 146101	Habp2	2119	NM 001256517	Zfp672	2919
NM 026844	Cmc2	1440	NM 001038607	Kcnh1	7062	NM 001159389	Rfx6	3457

NM 145732	Ctnna2	4004	NM 030091	Ola1	1019	NM 010481	Hspa9	3067
NM 009530	Atrx	10244	NM 177406	Cyp4a12a	2422	NM 134042	Aldh6a1	3346
NM 008046	Fst	2337	NM 001029994	Zc3h18	3742	NM 011031	P4ha2	2284
NM 011960	Parg	4391	NM 001039202	Hdhd2	1716	NM 001199305	Atxn1	10623
NM 172673	Frmd5	4218	NM 080435	Adcy4	3414	NM 001171615	Myt1	5305
NM 029512	Ttpal	4551	NM 016668	Bhmt	2081	NM 029568	Mfap4	1513
NM 009159	Srsf5	1532	NM 019966	Mlycd	2116	NM 028717	Als2	6512
NM 025993	Mis12	2766	NM 009818	Ctnna1	3713	NM 025918	Ccdc43	2307
NM 025510	Adprm	1492	NM 001033876	Kcnk9	1209	NM 009523	Wnt4	3823
NM 026688	Ndufs3	940	NM 010600	Kcnh1	7143	NM 178663	Bend7	3837
NM 009840	Cct8	2391	NM 212468	Ssbp1	1489	NM 011521	Sdc4	2460
NM_001081183	1110037F02Rik	7115	NM_001001184	Primpol	3813	NM_008839	Pik3ca	8917
NM 053200	Ces1d	1966	NM 028358	Ssbp1	1063	NM 144520	Sec14l2	2529
NM 011495	Plk4	3567	NM 010341	Nmur1	1218	NM 011184	Psma3	1434
NM 001110849	Prr5l	4159	NM 013676	Supt5	3561	NM 025494	Atp6v1c1	2117
NM 001081124	Map7d2	3854	NM 010833	Msn	3840	NM 011653	Tuba1a	1591
NM 198831	Mrpl48	968	NM 029291	Ascc2	2623	NM 001166503	Slc39a11	2742
NM 009278	Ssb	2088	NM 201226	Lrrc47	3395	NM 173180	Pmpca	3125
NM 138747	Nop2	2626	NM 021287	Sptbn2	8254	NM 001256520	Zfp672	2821
NM 001033305	Ndufb6	534	NM 026172	Decr1	3082	NM 007550	Blm	4869
NM 172839	Ccnj	3819	NM 023525	Cad	7159	NM 011691	Vav1	4168
NM 175549	Robo2	8047	NM 001081054	Qrsl1	1970	NM 011137	Pou2f1	12978
NM 001033348	Ralgapa2	9633	NM 145126	Chil4	1510	NM 009060	Rgn	1574
NM 001013829	Shf	1564	NM 172843	Tor1aip2	5857	NM 001034875	Bpifb4	2115
NM 007465	Birc2	3155	NM 028638	Gadl1	3652	NM 009346	Tead1	9899
NM 011062	Pdpk1	7175	NM 011468	Sprr2a1	3547	NM 001037938	Dhrs4	1408
NM 028887	Smchd1	7053	NM 026252	Cpeb4	7655	NM 021458	Fzd3	12742
NM 013507	Eif4g2	7760	NM 144884	Tor1a	1452	NM 028142	Nsun4	3526
NM_011948	Map3k4	5305	NM_023249	Ypel1	2879	NM_198662	9430007A20Rik	1406
NM 025641	Uqcrh	562	NM 001290676	Cpeb4	7631	NM 001162412	Cysltr2	1745
NM 011086	Pikfyve	11255	NM 009892	Chil3	1567	NM 001243769	Cdc42	1521
NM 001110145	Ssb	2061	NM 001290678	Cpeb4	7580	NM 177562	Clec16a	6262
NM 028398	Phykpl	1896	NM 175538	Nim1k	4103	NM 025970	Zbtb8os	660
NM 173033	Tstd2	3726	NM 001289523	Cad	6954	NM 172288	Nup133	5808
NM 029956	Mmab	3021	NM 001289522	Cad	6924	NM 009861	Cdc42	2151
NM 028185	Lsm11	6453	NM 007608	Car5a	1248	NM 024272	Ssbp2	6656
NM 001039939	Asxl1	6674	NM 198957	Rbm12b2	3457	NM 008448	Kif5b	6030
NM 026009	Ccdc47	3270	NM 177325	Tsr1	3395	NM 001163015	Gprasp2	3814
NM 177293	Mtap7d3	2795	NM 177303	Lrrn4	3475	NM 198933	Pou2f1	12978
NM 183221	Fat4	16109	NM 001085555	Tcp11	1764	NM 011860	Nlrp5	3511
NM 009797	Capza1	3187	NM 007451	Slc25a5	1238	NM 001163816	Vav1	4051
NM 001109764	Ctnna2	4148	NM 001110211	Anxa6	2643	NM 001166584	Tead1	9962
NM 021523	Huwe1	14637	NM 019434	Mcm3ap	6400	NM 008650	Mut	3675
NM 177782	Prex1	6530	NM 145935	Glyat	1310	NM 013486	Cd2	1144
NM 030256	Bcl9l	5341	NM 007889	Dvl3	2953	NM 001165943	Nsun6	2746
NM_178609	E2f7	5476	NM_175543	Rab11fip4	3145	NM_001160017	Gnb1	3091
NM 001024926	Cyb5d2	2393	NM 019840	Pde4b	4371	NM 010124	Eif4ebp2	1786
NM 198899	Uggt1	9045	NM 001081330	Dnah2	13704	NM 153141	Carm1	3151
NM 023210	Anp32e	3273	NM 025959	Psmc6	1564	NM 001177713	Cyp26b1	4700
NM 021384	Rsad2	3785	NM 001081149	Kat6a	9126	NM 011581	Thbs2	5922
NM 029840	Tstd3	1612	NM 133757	Pgs1	2298	NM 001252533	Cacnb2	3814
NM 001163792	Fam76a	2971	NM 176965	Efcab5	4654	NM 029870	Crebrf	7455
NM 026044	Dph7	2952	NM 172678	Acad9	3913	NM 027896	Coasy	2386
NM 007464	Birc3	2820	NM 033618	Supt16	4491	NM 001177980	Pde4b	4206
NM_179203	Atad3a	2426	NM_027860	0610010F05Rik	4140	NM_016682	Uba2	2524
NM 001113564	Serbp1	6667	NM 172859	Dzank1	6831	NM 001205312	Tyk2	4873
NM 001166594	Eif5a	1284	NM 001081430	Naa30	4597	NM 130867	Kirrel	7284
NM 144844	Pcca	2603	NM 001081269	Whsc1l1	9945	NM 001039090	Skil	6928

NM 017480	Icos	3272	NM 001038589	Usp14	4080	NM 028950	Nsun6	2929
NM 019879	Suclg1	1407	NM 028757	Nebl	2980	NM 153409	Csrnp3	10672
NM 172161	Irak2	3209	NM 029546	Pwp2	3871	NM 001165942	Nsun6	2420
NM 178617	Necab1	4936	NM 177652	Ryr3	15410	NM 001163815	Vav1	4096
NM 177785	BC049635	1656	NM 183286	Dhrs13	1744	NM 023047	Dpysl5	5077
NM 026175	Sf3a1	4916	NM 013559	Hsph1	3478	NM 011661	Tyr	3307
NM 001130485	Rpl35a	503	NM 001081324	Neto2	5646	NM 207213	Snx25	3067
NM 010209	Fh1	1644	NM 029804	Hnrnpm	2532	NM 010688	Lasp1	3433
NM 025814	Serbp1	6685	NM 133239	Crb1	4702	NM 175523	Ppm1k	5560
NM 001081050	Pard3b	8409	NM 175362	Card11	4112	NM 198934	Pou2f1	13050
NM 001159317	Il1rap	4031	NM 007603	Capn6	3579	NM 011582	Thbs4	3202
NM 001167994	Trmt2b	3337	NM 013472	Anxa6	2661	NM 031377	Pramel1	2440
NM 001159948	Als2	6681	NM 001104617	Rdx	1835	NM 018785	Prpf40a	3565
NM 172799	Ttll6	3223	NM 175113	Trmt6	2820	NM 133720	Cysltr2	1802
NM 028459	Wasl	4348	NM 177570	Slfnl1	1762	NM 026728	Echdc2	1323
NM 145519	Farp2	3929	NM 001083921	Rbck1	2410	NM 019794	Dnaja2	2839
NM 134147	Macrod1	1284	NM 175324	Acad11	3348	NM 133748	Insig2	2711
NM 001145959	Ndrg2	2115	NM 175517	Fam221b	1763	NM 001159648	Cntn1	5671
NM 146251	Pnpla7	4592	NM 018869	Grk5	3182	NM 001252518	Nono	2465
NM 009095	Rps5	768	NM 024439	Vimp	1208	NM 013681	Syn2	3858
NM 001114140	Tcf20	7364	NM 001005863	Mtus1	6554	NM 001077694	Dysf	6845
NM 145746	Odf4	1289	NM 025279	Hnrnpk	1978	NM 001289461	Hgf	2645
NM 009582	Map3k12	5352	NM 021521	Med12	8464	NM 001113545	Lima1	4245
NM 008303	Hspe1	788	NM 007450	Slc25a4	1142	NM 011273	Xpr1	7651
NM 020569	Park7	920	NM 009442	Ttf1	4170	NM 001100395	Otof	6907
NM 008565	Mcm4	3589	NM 001111043	Serpinh1	2273	NM 009856	Cd83	2141
NM 013836	Tcf20	7241	NM 026375	Ahctf1	8831	NM 001285906	Cd2bp2	3381
NM 025826	Acadsb	3348	NM 021522	Usp14	4185	NM 053170	Trim33	8870
NM 172540	Trmt2b	3261	NM 172565	Klhl11	2436	NM 029774	Ttll11	3191
NM 015747	Slc20a1	3316	NM 145542	Ahcyl1	3866	NM 010470	Hp1bp3	4852
NM 145553	Fam76a	3058	NM 026623	Nudt21	1111	NM 201232	Nipbl	10762
NM 007519	Baat	1961	NM 010722	Lmnb2	3389	NM 031875	Otof	7125
NM 013864	Ndrg2	2157	NM 001110512	Mettl8	1906	NM 001081146	Prickle2	7799
NM 029936	Ddx10	3368	NM 001081158	Cluh	5399	NM 001029936	Speccl	6749
NM 146124	Arhgap1	3113	NM 008475	Krt4	2147	NM 001285479	Hp1bp3	4849
NM 001166592	Eif5a	1378	NM 001102611	Smyd4	3517	NM 001289459	Hgf	2827
NM 025729	Tab3	6381	NM 009648	Akap1	3721	NM 001285784	Pars2	2245
NM 001164357	Slc25a25	3323	NM 001081383	Kmt2c	16820	NM 016812	Banp	5498
NM 033565	Aff4	10165	NM 007988	Fasn	9938	NM 021469	Dysf	6663
NM 001029856	Atad5	7272	NM 013631	Pklr	2787	NM 001134461	Prickle2	7695
NM 008254	Hmgcl	1416	NM 178618	Fam83g	4786	NM 001289458	Hgf	2838
NM 010497	Idh1	2270	NM 009825	Serpinh1	2400	NM 001286421	Otof	7065
NM 008892	Pola1	5350	NM 028093	Entpd8	2306	NM 027353	Cd2bp2	3361
NM 001111320	Idh1	2292	NM 009112	S100a10	650	NM 027707	Nipbl	10216
NM 008926	Prkg2	4696	NM 153796	Peo1	3515	NM 001289915	Cd83	2134
NM 021338	Rpl35a	549	NM 023230	Ube2v1	1950	NM 001080755	Zzz3	7607
NM 010022	Dbt	3292	NM 019779	Cyp11a1	1774	NM 001159516	Qk	5438
NM 030693	Atf5	1753	NM 008300	Hspa4	4657	NM 007594	Calu	3229
NM 001163643	Map3k12	5528	NM 029103	Manf	2206	NM 172272	Pars2	2228
NM 011592	Timm44	1798	NM 009408	Top1	3859	NM 001285478	Hp1bp3	4911
NM 172478	Tbc1d25	2659	NM 021335	Snrpb2	1230	NM 001285905	Cd2bp2	3367
NM 001130484	Rpl35a	519	NM 001081134	Kcng1	2316	NM 010427	Hgf	2810
NM 001113553	Irak2	3065	NM 172862	Frem2	12368	NM 001083887	Pars2	3077
NM 001159318	Il1rap	4320	NM 011879	Ik	1996	NM 145558	Hadhb	2045
NM 138600	Aldh7a1	2911	NM 028994	Pck2	3400	NM 001077495	Pik3r1	6928
NM 001159517	Qk	4658	NM 013807	Plk3	2474	NM 001256518	Zfp672	3201
NM 146090	Zadh2	3321	NM 017462	Polg	4596	NM 176841	Ccdc88a	8862
NM 027434	Rprd1b	4517	NM 183173	Sowaha	3644	NM 008525	Alad	4536
NM 001291136	Rprd1b	4387	NM 026041	Rrp15	1228	NM 001081204	B3glct	4543
NM 010198	Fgf11	2618	NM 025855	Echdc1	3048	NM 133780	Tpr	7550
NM 001291182	Ush1c	3042	NM 013895	Timm13	1225	NM 013468	Ankrd1	1765
NM 007461	Apba2	3334	NM 028360	Ttc19	3419	NM 016889	Insm1	3038

NM 001252585	Zmynd8	5072	NM 010324	Got1	2065	NM 009186	Tra2b	2135
NM 153677	Ush1c	3065	NM 009041	Rdx	4580	NM 013528	Gfpt1	6248
NM 025996	Tomm34	2319	NM 009281	Zfp143	3013	NM 008518	Ltb	1122
NM 023649	Ush1c	2044	NM 001081216	Phip	10729	NM 011652	Ttn	101674
NM 001163733	Ush1c	3073	NM 001083587	Tns3	7483	NM 001081211	Ptafr	1820
NM 001291151	Ptprt	12126	NM 025745	Erlec1	3501	NM 001005385	Gprasp1	5697
NM 001252584	Zmynd8	5180	NM 001111044	Serpinh1	2229	NM 011605	Tmpo	3821
NM 027230	Zmynd8	5423	NM 021559	Zfp191	3143	NM 010053	Dlx1	2779
NM_021464	Ptprt	12099	NM_026091	1700037H04Rik	1241	NM_008890	Pnmt	888
NM 011048	Pcsk6	4298	NM 001040435	Tacc3	2637	NM 008769	Otc	2265
NM 153594	Pcmtd2	3568	NM 007590	Calm3	2233	NM 001159731	Rxrg	1695
NM 008746	Ntrk3	2978	NM 175310	Pds5b	7311	NM 001004359	Gprasp1	5811
NM 018888	Uqcc1	2668	NM 207515	Mbnl2	4525	NM 001276446	Alad	4447
NM 007454	Ap1b1	4109	NM 009717	Neurod6	2154	NM 001083334	Bin1	2135
NM 181591	Nme8	2088	NM 177041	Flad1	2223	NM 001110100	Banp	5489
NM 010588	Jag2	4054	NM 001004157	Scarf1	2884	NM 011183	Psen2	2026
NM 001252107	Cadps2	3148	NM 031179	Sf3b1	6192	NM 001079830	Trim33	8819
NM 029600	Abcc3	5002	NM 009847	Cd2ap	5427	NM 009347	Tecta	7338
NM 020027	Prrc2a	6913	NM 007790	Smc3	4851	NM 001134460	Prickle2	8200
NM 001198985	Tjp2	4637	NM 133345	Ing4	1617	NM 013708	Nr2e3	1999
NM_001160360	A130010J15Rik	3630	NM_013830	Prpf4b	4482	NM_001170847	Rbm20	6607
NM 172532	Aldh5a1	5647	NM 001081090	Esf1	3392	NM 001167680	Rhbdf2	3713
NM 008359	Il17ra	3970	NM 018819	Mpc1	907	NM 030168	Rictor	9328
NM 009873	Cdk6	2470	NM 009819	Ctnna2	4046	NM 138749	Plxnb2	6519
NM 139206	Arap3	5266	NM 013825	Ly75	5172	NM 001284506	Plxnb2	6469
NM 001081141	Gabbr2	3998	NM 030689	Nptxr	4973	NM 026081	Gprasp1	5900
NM 023116	Cacnb2	3945	NM 145524	Mettl8	2370	NM 172572	Rhbdf2	3927
NM_028099	Dusp11	6445	NM_172616	C330027C09Rik	3974	NM_001122897	Hp1bp3	4891
NM 028903	Scara5	3809	NM 009418	Tpp2	4649	NM 145541	Rap1a	2474
NM 009677	Ap1g1	6817	NM 013687	Tcp11	2001	NM 009158	Mapk10	7203
NM 053207	Egln1	3524	NM 145732	Ctnna2	4004	NM 009107	Rxrg	2151
NM 145381	Lactb2	1889	NM 009530	Atrx	10244	NM 153054	Slc18a1	3118
NM 001252108	Cadps2	2232	NM 001042541	Akap1	3758	NM 175460	Nmnat2	4548
NM 001252106	Cadps2	4663	NM 023059	Sigirr	1990	NM 001081567	Mapk10	7198
NM 007780	Csf2rb	4776	NM 007481	Arf6	3740	NM 134157	Atp6v1b1	1945
NM 001199274	Mat2b	2066	NM 133975	Trip12	9632	NM 019924	Rps6ka4	3152
NM 001205336	Arap3	5425	NM 008149	Gpam	3852	NM 007555	Bmp5	2528
NM 001081391	Csmd3	13022	NM 008710	Nnt	5355	NM 021423	Shank3	7131
NM 001122893	Fyn	3544	NM 025993	Mis12	2766	NM 001285783	Pars2	3094
NM 028940	Clvs1	3628	NM 029044	Lrrc48	1858	NM 028004	Ttn	81931
NM 029371	Htatsf1	2793	NM 007573	C1qbp	1177	NM 145575	Cald1	4035
NM 001081172	Frmpd1	4827	NM 001081395	Amotl1	8951	NM 025350	Cpa1	1578
NM_001160359	A130010J15Rik	3782	NM_009840	Cct8	2391	NM_001159521	Plxnb2	6555
NM_172416	Ostm1	3009	NM_001081183	1110037F02Rik	7115	NM_001285907	Cd2bp2	3509
NM 001252105	Cadps2	4849	NM 053200	Ces1d	1966	NM 009481	Usp9x	11903
NM 001177572	Slc25a13	3130	NM 009109	Ryr1	15362	NM 009668	Bin1	2468
NM 153163	Cadps2	4969	NM 009307	Syt2	3386	NM 001281818	Specc1	6966
NM 009895	Cish	2161	NM 001081124	Map7d2	3854	NM 001128605	Psen2	2017
NM 010741	Ly6c1	865	NM 144826	Utp6	3871	NM 001103157	Steap2	10696
NM 001033422	Thoc2	7631	NM 019705	Rbck1	2251	NM 033562	Derl2	3668
NM 001252055	Ly6c1	880	NM 001104616	Rdx	4174	NM 001126331	Trp73	4468
NM 010917	Nid1	6046	NM 016847	Avpr1a	2671	NM 011344	Sell1	6150
NM 011597	Tjp2	4627	NM 133354	Sumo2	998	NM 016976	Grm1	6922
NM 001243043	Ap1b1	4336	NM 023480	Fahd1	1408	NM 021415	Cacna1h	8240
NM 001199431	Dnmt1	5370	NM 001080815	Gipr	1792	NM 001290661	Sh3kbp1	3850
NM 022314	Tpm3	2239	NM 001033348	Ralgapa2	9633	NM 020505	Vav3	5023
NM 026573	Upf3b	2188	NM 139300	Mylk	7809	NM 001103156	Steap2	10341

NM 030228	Gas2l1	3986	NM 175341	Mbnl2	4579	NM 010928	Notch2	10506
NM 024255	Hsdl2	2632	NM 007465	Birc2	3155	NM 001110796	Pclo	16895
NM 133807	Lrrc59	2810	NM 028887	Smchd1	7053	NM 001135727	Sh3kbp1	5168
NM 010049	Dhfr	5307	NM 013507	Eif4g2	7760	NM 001163676	Abcc4	5504
NM 001171680	Myt1	5495	NM 011948	Map3k4	5305	NM 008855	Prkcb	8830
NM 008563	Mcm3	2886	NM 011086	Pikfyve	11255	NM 001290664	Sh3kbp1	3721
NM 001190406	Gas2l1	3631	NM 028398	Phykpl	1896	NM 027180	Arap1	4929
NM 026062	Fam69a	2724	NM 020279	Ccl28	3753	NM 001163691	Cacna1h	8187
NM 009963	Cry2	4019	NM 028185	Lsm11	6453	NM 001290542	Plekhg2	5290
NM 010150	Nr2f6	2218	NM 001015046	Rap1gap2	6410	NM 009700	Aqp4	5082
NM 010132	Emx2	2598	NM 001039939	Asxl1	6674	NM 010788	Mecp2	10233
NM 130888	Nxf7	1863	NM 177293	Mtap7d3	2795	NM 001290729	Taf1	8033
NM 026194	Ufl1	4335	NM 183221	Fat4	16109	NM 016966	Phgdh	1871
NM 031180	Klb	3439	NM 009797	Capza1	3187	NM 011642	Trp73	5046
NM 001081125	Gli2	6708	NM 001109764	Ctnna2	4148	NM 146139	Vav3	3061
NM 010213	Fhl3	1685	NM 172812	Htr2a	2971	NM 001285470	Steap2	10215
NM 011317	Khdrbs1	3762	NM 021523	Huwe1	14637	NM 001033336	Abcc4	5729
NM 001199044	Prrc2a	6910	NM 031165	Hspa8	2104	NM 001290675	Scn9a	9832
NM 010301	Gna11	3262	NM 001099779	Pklr	2661	NM 001039089	Sell1	6300
NM 173765	Aasdh	3694	NM 177782	Prex1	6530	NM 001081122	Cep63	2696
NM 019582	Cacna1f	6075	NM 025471	Smim8	927	NM 054098	Steap4	3140
NM_182809	Ntrk3	3847	NM_001110133	1700093K21Rik	1338	NM_020623	Pth	717
NM 029426	Brsk2	4047	NM 013463	Gla	3032	NM 001135728	Sh3kbp1	4170
NM_026262	4930524B15Rik	1959	NM_178609	E2f7	5476	NM_001003908	Cltc	6178
NM 175438	Aldh4a1	3355	NM 019656	Tspan6	1763	NM 019454	Dll4	3451
NM 016980	Rpl5	2036	NM 023741	Prl8a8	908	NM 013686	Tcp1	2499
NM 008552	Mas1	2475	NM 029946	Efcab6	5031	NM 001285469	Steap2	10250
NM 010072	Dpm1	2293	NM 008686	Nfe2l1	4654	NM 001126330	Trp73	4756
NM 177710	Ssh2	9040	NM 001161629	Efcab6	2412	NM 001290666	Csrnp3	10266
NM 001252110	Cadps2	4834	NM 144940	Uroc1	3365	NM 011995	Pclo	20099
NM 146116	Tubb4b	1600	NM 001167922	Gtf2e2	1664	NM 033324	Dgcr8	4226
NM 009030	Rbbp4	4407	NM 011247	Rbbp6	6199	NM 010080	Dspp	4431
NM 001009930	Brsk2	4507	NM 009900	Clcn2	3670	NM 001040111	Arap1	5837
NM 013839	Nr1h3	1955	NM 001024926	Cyb5d2	2393	NM 021389	Sh3kbp1	4733
NM 178665	Lpp	15669	NM 023190	Acin1	4751	NM 176930	Nrcam	7572
NM 001166585	Tead1	9480	NM 026584	Gtf2e2	1550	NM 001083927	Tle3	5202
NM 134000	Traf3ip2	2867	NM 001134459	Prickle2	7878	NM 011345	Sele	2912
NM 001110859	Crem	2362	NM 019698	Aldh18a1	3544	NM 009389	Tle3	5172
NM 024236	Qdpr	1394	NM 023523	Pecr	1205	NM 001114333	Grm1	7007
NM 001165941	Nsun6	3042	NM 001128607	Epb4.1	5067	NM 001083928	Tle3	5148
NM 001159714	Prpf31	3125	NM 001130149	Drosha	4584	NM 001291147	Derl2	3703
NM 133801	Gtf2f1	1720	NM 008777	Pah	2152	NM 001163675	Abcc4	5711
NM 028108	Naa50	4526	NM 019927	Arih1	6435	NM 001081979	Mecp2	10152
NM 028151	Skiv2l2	3309	NM 025647	Cmpk1	1880	NM 001291148	Derl2	3665
NM 134129	Prpf19	6161	NM 001163642	Setdb1	4641	NM 028734	Steap2	10398
NM 001033341	Zfp407	7909	NM 172543	Fam117a	2334	NM 016779	Dmp1	2760
NM 011630	Nr2c2	7637	NM 026044	Dph7	2952	NM 007809	Cyp17a1	1841
NM 001252200	Map4k4	5782	NM 001130454	Nfe2l1	3514	NM 001146031	Nrcam	7362
NM 008876	Pld2	3801	NM 010274	Gpd2	5620	NM 153413	Dock3	9067
NM 198932	Pou2f1	12906	NM 001085473	Acin1	4715	NM 001290393	Sntg1	7213
NM 001110857	Crem	2026	NM 007464	Birc3	2820	NM 021396	Pdcd1lg2	3415
NM 001243240	Cacfd1	2205	NM 023850	Chst1	2682	NM 001290986	Wdr76	4246
NM_027041	1700003M02Rik	1716	NM_001039104	Trpm1	5711	NM_001040112	Arap1	5314
NM 001166591	Eif5a	1391	NM 023731	Ccdc86	3003	NM 001039484	Kcnj10	5407
NM 175730	Hoxc5	2730	NM 172303	Jade1	5584	NM 001285811	Dtna	2152
NM 001034894	Foxr2	3208	NM 023336	Brd3	5429	NM 001081356	Vma21	4195
NM 001166590	Eif5a	1427	NM 026653	Rpa1	3058	NM 019464	Sh3glb1	5903
NM 011708	Vwf	8834	NM 019879	Suclg1	1407	NM 007610	Casp2	3463
NM 144931	Nae1	1808	NM 172778	Maob	2428	NM 001282037	Sh3glb1	5966

NM 133808	Hdlbp	6238	NM 029629	Fahd2a	1242	NM 001290540	Pdzd4	3646
NM 001271447	Enox2	3977	NM 023057	Zak	3365	NM 001290712	Tcp1	2413
NM 001110852	Crem	1440	NM 207242	Npc1l1	4571	NM 001290501	Znrf3	7199
NM 198007	Ascc3	7477	NM 001145820	Gpd2	5745	NM 001271472	Scube1	7932
NM 001042527	Blm	4598	NM 013889	Zfp292	9977	NM 001290733	Osbpl6	8301
NM 001252455	Ptprs	5644	NM 001113573	Brd3	5269	NM 001289787	Slc26a5	4430
NM 181417	Csrp2bp	3748	NM 011961	Plod2	3656	NM 001271678	Wnk3	10400
NM 007937	Epha5	7789	NM 007512	Atpif1	552	NM 001271533	Spag16	3589
NM 009124	Atxn1	10599	NM 178617	Necab1	4936	NM 001291203	Tpd52l2	3520
NM 008364	Il1rap	4576	NM 177785	BC049635	1656	NM 001286040	Anks1	7092
NM 181582	Eif5a	1361	NM 026175	Sf3a1	4916	NM 001287195	Esp6-esp5	1229
NM 010313	Gnb5	2249	NM 030189	Stpg1	2380	NM 001285994	Enpp2	3536
NM 001171616	Myt1	5415	NM 010209	Fh1	1644	NM 001291189	Cdh26	2965
NM 031869	Prkab1	2051	NM 001130451	Nfe2l1	3530	NM 001291045	Grm4	3987
NM 019766	Ptges3	1954	NM 001130186	Jade1	5524	NM 001286032	Dhx32	2448
NM 001255992	Bbs12	2347	NM 001159317	Il1rap	4031	NM 001285872	Pafah2	3194
NM 029286	Ccdc30	3482	NM 028958	Taf7l	1935	NM 001290390	Sntg1	7264
NM 177578	Skint3	3627	NM 001113574	Brd3	5320	NM 001291055	Lgals8	2522
NM 001145952	Lpp	15462	NM 010892	Nek2	3218	NM 001285983	Banp	5372
NM 008825	Pfkfb2	7308	NM 001130185	Jade1	5566	NM 001290645	C8a	3532
NM 001159647	Cntn1	5817	NM 176996	Smo	3977	NM 001252513	Lipi	2082
NM 001136085	Uba1	4073	NM 175177	Bdh1	3114	NM 001285963	Skint2	1258
NM 019710	Smc1a	3980	NM 172799	Ttll6	3223	NM 001291057	Lgals8	2718
NM 011386	Skil	7066	NM 133933	Rpn1	3642	NM 001289731	Mcf2	4227
NM 008696	Map4k4	5620	NM 008287	Hrsp12	1011	NM 001290633	Reps2	7695
NM 007638	Cct7	2452	NM 008797	Pcx	4125	NM 001290476	Klhl13	3232
NM 001145902	Arhgap1	2904	NM 001130184	Jade1	5486	NM 001271473	Scube1	7599
NM_172679	4932438A13Rik	15883	NM_009532	Xrcc1	2155	NM_027096	Gpcpd1	3516
NM 027382	Hdac8	1724	NM 011588	Trim28	3255	NM 001290807	Haus2	3070
NM 019677	Plcb1	7116	NM 146251	Pnpla7	4592	NM 001271601	Ncaph2	3343
NM 011294	Sub1	3321	NM 028226	Rbm12b1	3313	NM 175272	Nav2	11332
NM 001145779	Kif2a	3753	NM 026849	Mtmr14	2676	NM 001286041	Anks1	7029
NM 001110856	Crem	2587	NM 008133	Glud1	3158	NM 007566	Birc6	15787
NM 021280	Plcg1	4435	NM 001167921	Gtf2e2	1562	NM 001134743	Lrrtm4	3054
NM 001199304	Atxn1	10474	NM 173748	Nudcd3	3977	NM 001290570	Ralgps1	6077
NM 029556	Clybl	1257	NM 183428	Epb4.1	6565	NM 001290541	Acer2	4068
NM 011078	Phf2	5260	NM 153553	Npas4	3292	NM 001290389	Atp1b4	4266
NM 001009929	Brsk2	4142	NM 178084	Zak	1303	NM 001271600	Ncaph2	3247
NM 010949	Numb	3235	NM 009582	Map3k12	5352	NM 001291161	Timm22	2757
NM 010822	Mpg	1745	NM 029402	Cul2	4158	NM 001190400	Bend7	3834
NM 007382	Acadm	2062	NM 008565	Mcm4	3589	NM 001290448	Nfx1	4368
NM 001244903	Celf1	7796	NM 001164223	Rpa1	3121	NM 001290369	Nmt2	4411
NM 010066	Dnmt1	5367	NM 011997	Casp8ap2	6805	NM 001271810	Pcdh11x	8607
NM 011424	Ncor2	8745	NM 172573	Engase	3922	NM 001276764	Dst	24271
NM 001168501	Zfp57	1712	NM 178654	Pkn2	6207	NM 001277106	Crlf3	2283
NM 146118	Slc25a25	3342	NM 011291	Rpl7	937	NM 001291050	Gpcpd1	3592
NM_009211	Smarcc1	5684	NM_175326	D330045A20Rik	3738	NM_001276680	Syt6	1857
NM 001243062	Nr1i3	1322	NM 001128606	Epb4.1	5141	NM 001282126	Brpf1	4883
NM 178892	Tiparp	4154	NM 001164358	Slc25a25	3581	NM 001276398	Ms4a7	1576
NM 001039507	Lipe	2785	NM 028094	Ugt2a3	2117	NM 001289735	Nxf2	2395
NM 199473	Col8a2	4331	NM 146124	Arhgap1	3113	NM 001291190	Ssh2	9176
NM 001030307	Dkc1	2803	NM 001122978	Casp8ap2	6558	NM 177408	Gabrg2	3911
NM 152801	Arhgef6	4580	NM 011267	Rgs16	2351	NM 001287139	Zzz3	7546
NM 001252642	Hrh1	3804	NM 001164357	Slc25a25	3323	NM 030706	Trim2	7207
NM 026079	Ikbkap	6160	NM 018877	Setdb1	4644	NM 001277122	Hnrnpr	7861
NM 001164791	Zak	6704	NM 024433	Mtap	2558	NM 001271413	Nfam1	3801
NM 011551	Ubtf	4704	NM 133198	Pygl	2821	NM 001272024	Sema6c	4140
NM 008665	Myt1	5529	NM 001130450	Nfe2l1	4596	NM 001113360	Plch2	5067
NM 134141	Ciapin1	4327	NM 008254	Hmgcl	1416	NM 001291114	Rbm39	2803
NM 080728	Myh7	6054	NM 008892	Pola1	5350	NM 001271726	Trim2	7144

NM 001244891	Celf1	7849	NM 025674	Tcf19	1664	NM 001040434	Rgag1	4142
NM 013930	Aass	3701	NM 008926	Prkg2	4696	NM 001290747	Tbx22	3073
NM 181819	Wfikkn2	3392	NM 001130452	Nfe2l1	3497	NM 001290780	Vma21	4322
NM 001110850	Crem	2404	NM 008214	Hars	1999	NM 001290713	Stag2	5814
NM 007727	Cntn1	5720	NM 007589	Calm2	1216	NM 001276248	Cp	4561
NM 029049	Ptchd3	3238	NM 134103	Il1rap	2352	NM 001290524	Mospd2	2706
NM 001163032	Synpr	2657	NM 010613	Khsrp	4001	NM 001289707	Clec2i	2331
NM 001166596	Eif5a	1293	NM 010022	Dbt	3292	NM 001276397	Ube2d2b	1616
NM 001271449	Enox2	3966	NM 198094	Brd4	2731	NM 001166028	Slfn14	3704
NM 172923	AI118078	2407	NM 001163643	Map3k12	5528	NM 001290824	Tubgcp4	4275
NM 010019	Dapk2	1792	NM 001130453	Nfe2l1	3547	NM 001285831	Far1	4339
NM 001167898	Frem3	6626	NM 011767	Zfr	4612	NM 001276704	Nxf1	4035
NM 015829	Slc25a13	3133	NM 013762	Rpl3	1361	NM 001291025	Pigq	3295
NM 008073	Gabrg2	3935	NM 001163764	Tcf19	1616	NM 001271491	Tfe3	2908
NM 175467	Sptlc3	2759	NM 001159318	Il1rap	4320	NM 001290718	Sytl4	3325
NM 011766	Zfpm2	4974	NM 001122683	Bdh1	3133	NM 001290674	Scn9a	9865
NM 001042503	Trim71	5197	NM 026799	Drosha	4580	NM 001290523	Mospd2	2487
NM 001110858	Crem	1990	NM 001013766	Samd3	2620	NM 001271568	Cdc7	2851
NM 011192	Psme3	2620	NM 010721	Lmnb1	2871	NM 001286002	Scyl3	4160
NM 001276317	Uba1	4068	NM 001163763	Tcf19	1773	NM 001291111	Gss	1843
NM 001276316	Uba1	4165	NM 001163641	Setdb1	4682	NM 001290745	Nckap1	4421
NM 001164528	Ildr2	8260	NM 001161628	Efcab6	2924	NM 001271414	Nfam1	3889
NM 198683	Celf1	4721	NM 146090	Zadh2	3321	NM 001271759	Adra1a	4118
NM 028506	Ccdc30	2730	NM 027434	Rprd1b	4517	NM 001276489	Ism1	2965
NM 008740	Nsf	3766	NM 008180	Gss	1978	NM 001276250	Cp	4712
NM 199469	Nploc4	4589	NM 001291136	Rprd1b	4387	NM 001271567	Cdc7	2872
NM 028244	Rrp1b	4809	NM 001291182	Ush1c	3042	NM 001271489	Tfe3	3274
NM 134042	Aldh6a1	3346	NM 007461	Apba2	3334	NM 001291150	Ptprt	12129
NM 175151	Tatdn1	1366	NM 001252585	Zmynd8	5072	NM 001271679	Wnk3	10259
NM 001110853	Crem	2270	NM 153677	Ush1c	3065	NM 001290786	Muc15	3222
NM 001013745	Zfp57	1846	NM 001098799	Tox2	1675	NM 001277121	Hnrnpr	8022
NM 001252202	Map4k4	5542	NM 023649	Ush1c	2044	NM 001291052	Gpcpd1	3640
NM 001136075	Numb	3382	NM 182840	Emilin3	3588	NM 001291059	Elf2	5864
NM 001199305	Atxn1	10623	NM 001163733	Ush1c	3073	NM 001284189	Homer1	4768
NM 001171615	Myt1	5305	NM 001291151	Ptprt	12126	NM 001271436	Haghl	1150
NM 001166589	Eif5a	1455	NM 001291152	Sgk2	2436	NM 001290690	Kcnip1	2005
NM 009474	Uox	1843	NM 001252584	Zmynd8	5180	NM 001276467	Asap1	6110
NM 029568	Mfap4	1513	NM 027230	Zmynd8	5423	NM 001285995	Enpp2	3605
NM 001271503	Crem	2137	NM 001037713	Xaf1	2598	NM 001285427	Cacnb4	8088
NM 008285	Hrh1	3782	NM 021464	Ptprt	12099	NM 001290500	Ttc14	7536
NM 028717	Als2	6512	NM 013731	Sgk2	2554	NM 001290644	Rp2h	4581
NM 198005	Dcaf17	1811	NM 198894	Abr	5124	NM 001285459	Fmn1	11147
NM 145354	Nsun2	4280	NM 001291197	Tpd52l2	3625	NM 001276462	Asap1	6181
NM 145951	Enox2	3921	NM 001291201	Tpd52l2	3565	NM 001290665	Csrnp3	10644
NM 009803	Nr1i3	1428	NM 001291204	Tpd52l2	3371	NM 001291049	Usp43	4447
NM 009551	Zfand5	7385	NM 025482	Tpd52l2	3622	NM 001290370	Nmt2	4368
NM 007550	Blm	4869	NM 153594	Pcmtd2	3568	NM 001290387	Pdp1	4149
NM 011691	Vav1	4168	NM 198018	Abr	5238	NM 178893	Coro2a	4112
NM 133662	Ier3	1090	NM 198895	Abr	5245	NM 001105197	Tfe3	2979
NM 001145830	Plcb1	6998	NM 198656	Cdh26	3022	NM 001271437	Haghl	1145
NM 011137	Pou2f1	12978	NM 001291222	Kif7	4525	NM 001282102	Lrrtm4	3629
NM 001252643	Hrh1	3868	NM 010626	Kif7	4522	NM 001290698	Synj2	4779
NM 001034875	Bpifb4	2115	NM 001291236	Tlcd1	1619	NM 001290385	Arhgef9	5532
NM 019641	Stmn1	1070	NM 009839	Cct6b	1817	NM 001277941	Rad51d	6937
NM 009346	Tead1	9899	NM 018888	Uqcc1	2668	NM 001277170	Rbm46	3586
NM 138719	Gnb5	2225	NM 001252659	Ldlr	4546	NM 001289470	Cd84	3287
NM 001029890	Mex3a	5776	NM 181591	Nme8	2088	NM 001193266	Mdga2	9834
NM 153540	Pomgnt2	2369	NM 019572	Hdac7	4240	NM 001271727	Trim2	7413
NM 001168502	Zfp57	2088	NM 001177732	Plch1	6294	NM 001290656	Mbd5	5301
NM 013716	G3bp1	2685	NM 010588	Jag2	4054	NM 001285507	Cops2	3353
NM 172523	Slc18a2	3803	NM 001252107	Cadps2	3148	NM 001284196	Srsf10	3433
NM 170592	Ntmt1	1217	NM 016978	Oat	2155	NM 001290785	Sec24a	6795

NM 001252453	Ptprs	5656	NM 029600	Abcc3	5002	NM 001277123	Hnrnpr	7856
NM 021458	Fzd3	12742	NM 133219	Gcnt2	4021	NM 001085410	Nadk2	3740
NM 001170967	Wdr33	1148	NM 008395	Itch	5187	NM 001290378	Ap1s2	2217
NM 001166593	Eif5a	1304	NM 001177733	Plch1	6289	NM 177203	Ccdc169	2269
NM 177082	Sp8	4480	NM 001081959	Klc1	2480	NM 001271490	Tfe3	3446
NM 009074	Mst1r	4706	NM 001170785	Mthfd1l	3743	NM 001277867	Mphosph9	7850
NM 028142	Nsun4	3526	NM 024434	Lap3	2267	NM 001285471	Steap2	4624
NM_198662	9430007A20Rik	1406	NM_139206	Arap3	5266	NM_001284190	Agbl4	1659
NM 001243769	Cdc42	1521	NM 001085521	Syndig1	2073	NM 001174047	Cacna2d2	5539
NM 001166595	Eif5a	1226	NM 008256	Hmgcs2	3294	NM 001291063	Elf2	5495
NM 013498	Crem	2423	NM 198162	Morc2a	4443	NM 001111016	Nav2	11070
NM 172288	Nup133	5808	NM 001170975	Dbndd1	1540	NM 001290435	Gabrq	4943
NM 001252201	Map4k4	5581	NM 133648	Slc12a6	5970	NM 184088	Dennd4c	7793
NM 009861	Cdc42	2151	NM 001162416	Pfkfb2	2382	NM 001282015	Tyrp1	2734
NM 001195023	Nploc4	4685	NM 023887	Gcnt2	4361	NM 001291200	Tpd52l2	3580
NM 001167909	Nme8	1704	NM 007780	Csf2rb	4776	NM 001276463	Asap1	6145
NM 008448	Kif5b	6030	NM 001252572	Cttn	3112	NM 001290736	Unkl	4787
NM 001270446	Ccdc30	2723	NM 001170786	Mthfd1l	3623	NM 001276423	Cass4	3627
NM 198933	Pou2f1	12978	NM 001252545	Sec23b	2781	NM 001284524	Chit1	1580
NM 001170970	Wdr33	1152	NM 008742	Ntf3	1342	NM 001291039	Cds2	8089
NM 028242	Htatsf1	2855	NM 009100	Rptn	4146	NM 010081	Dst	8788
NM 001163816	Vav1	4051	NM 001205336	Arap3	5425	NM 001290425	Usp45	5874
NM 001166584	Tead1	9962	NM 019830	Prmt1	1379	NM 001174048	Cacna2d2	5521
NM 001003920	Brsk1	3014	NM 023138	Map2k2	2387	NM 001290376	Camk1d	6742
NM 001252456	Ptprs	5618	NM 001252544	Sec23b	2792	NM 001278671	Kbtbd12	2042
NM 001165943	Nsun6	2746	NM 011327	Scp2	2664	NM 001291160	Stau1	3651
NM 001110851	Crem	1587	NM 028146	Dbndd1	1681	NM 001291229	Clip1	5649
NM_028754	0610037L13Rik	1551	NM_001081391	Csmd3	13022	NM_001291051	Gpcpd1	3622
NM 134017	Mat2b	1870	NM 029371	Htatsf1	2793	NM 001290410	Nkain3	4668
NM 008867	Pla2r1	7112	NM 001081172	Frmpd1	4827	NM 001284229	Nasp	2037
NM 017366	Acadvl	2205	NM 001159288	Morc2a	5085	NM 001291202	Tpd52l2	3562
NM 001081081	Gls	4974	NM 027748	Taf3	4821	NM 001290449	Nfx1	4899
NM 011581	Thbs2	5922	NM 008105	Gcnt2	4383	NM 001290459	Hdx	4114
NM 008008	Fgf7	2672	NM 026410	Cdca5	1906	NM 001271843	Aqp9	2646
NM 001252533	Cacnb2	3814	NM 001177793	Snap23	2145	NM 001271883	Vmn2r124	2568
NM 029870	Crebrf	7455	NM 001146100	Hk1	4157	NM 001290995	Kif1b	10288
NM 001270435	Ccdc30	3467	NM 001252658	Ldlr	4393	NM 001290741	Cnot6	5372
NM 007998	Fech	2901	NM 007803	Cttn	3223	NM 001271725	Trim2	7108
NM 011026	P2rx4	1995	NM 001110504	Capn1	3067	NM 001287530	Arhgap6	4147
NM 017368	Celf1	4873	NM 008450	Klc1	2398	NM 001276461	Asap1	6101
NM 001039090	Skil	6928	NM 183191	Plch1	6266	NM 001289624	St7	2210
NM 028950	Nsun6	2929	NM 010700	Ldlr	4549	NM 001271772	Skil	6609
NM 025816	Tax1bp1	3307	NM 001025360	Klc1	2504	NM 001290443	Spaca1	1177
NM 001044383	Ubtf	4458	NM 133807	Lrrc59	2810	NM 001276355	Grin3a	7727
NM 001165942	Nsun6	2420	NM 172308	Mthfd1l	3731	NM 001289626	St7	2141
NM 001163815	Vav1	4096	NM 027963	Wdr16	2201	NM 001290734	Osbpl6	7968
NM 001243239	Cacfd1	1286	NM 001204277	Hdac7	4258	NM 001290684	Shroom2	7501
NM 001271448	Enox2	3822	NM 001171680	Myt1	5495	NM 001291062	Elf2	5459
NM 027328	Prpf31	3143	NM 011099	Pkm	2432	NM 001040395	Nadk2	3674
NM 010688	Lasp1	3433	NM 001177792	Snap23	2223	NM 001290308	Col12a1	11719
NM 001243044	Ap1b1	4118	NM 009222	Snap23	2190	NM 001113356	C1rb	2166
NM 146109	Als2	2977	NM 026062	Fam69a	2724	NM 001290283	Pld6	1773
NM 175523	Ppm1k	5560	NM 001252313	Ncor1	8852	NM 001284354	Shprh	6832
NM 198934	Pou2f1	13050	NM 177420	Psat1	2172	NM 001290689	Naa60	2323
NM_181048	A130010J15Rik	3792	NM_177354	Vash1	6189	NM_001271411	Nfam1	3962
NM 025358	Ndufa9	1322	NM 026194	Ufl1	4335	NM 001282127	Brpf1	4546
NM 011582	Thbs4	3202	NM 013831	Pstpip2	3138	NM 001290627	Atp4a	3497
NM 173169	Plk4	3486	NM 019787	Sec23b	2810	NM 001286824	Uspl1	3730
NM 001177730	Nr1h3	1802	NM 008471	Krt19	1509	NM 001285458	Fmn1	11523

NM 018785	Prpf40a	3565	NM 024457	Rap1b	1937	NM 001290391	Pdp1	4184
NM 001033328	BC023829	1989	NM 010836	Msx3	2287	NM 001290379	Ap1s2	2226
NM 016910	Ppm1d	2911	NM 013565	Itga3	4870	NM 001290797	Inpp4a	5862
NM 001159648	Cntn1	5671	NM 010363	Gstz1	1683	NM 001285425	Tcf24	3766
NM 011695	Vdac2	1662	NM 001252543	Sec23b	2799	NM 001290662	Kif2c	2792
NM 011218	Ptprs	6874	NM 007600	Capn1	3119	NM 001290788	Aqr	4414
NM 011221	Purb	8478	NM 009878	Cdkn2d	1271	NM 001290781	Vma21	4496
NM_015812	Rgs6	2626	NM_026760	2310036022Rik	934	NM_001289728	Tmem255a	3362
NM 007791	Csrp1	1770	NM 029704	Ttc19	3397	NM 001276359	Camsap1	7996
NM 026125	Fam132a	1339	NM 133649	Slc12a6	6631	NM 001290467	Hs6st2	4672
NM 001243063	Nr1i3	1425	NM 019582	Cacna1f	6075	NM 001289627	St7	1944
NM 026494	Ppcs	1440	NM 029426	Brsk2	4047	NM 001291149	Ptprt	12159
NM 011655	Tubb5	2645	NM 001242407	Fam73b	3394	NM 001291146	Derl2	3959
NM_012027	Mprip	8784	NM_026262	4930524B15Rik	1959	NM_001276406	Nup88	2467
NM 001286664	Ssbp1	2047	NM 001142744	Atat1	1463	NM 001290669	Ttc21b	4402
NM 001039138	Camk2g	3693	NM 001142745	Atat1	2068	NM 001289629	St7	1875
NM 001289579	Chfr	3253	NM 153524	Mrgpra4	1503	NM 175556	Plch2	5598
NM 172545	Ehmt1	5086	NM 028476	Atat1	1999	NM 172997	Idua	4289
NM 007985	Fancc	3134	NM 010072	Dpm1	2293	NM 001277942	Rad51d	6910
NM 001289461	Hgf	2645	NM 177710	Ssh2	9040	NM 001285981	Banp	5381
NM 172464	Daam1	5888	NM 175531	Mrgprb2	2089	NM 001276444	Gpr155	4885
NM 026102	Daam1	6023	NM 001033276	Kmt2d	19827	NM 001290717	Sytl4	3352
NM 009196	Slc16a1	4430	NM 024219	Hsbp1	1178	NM 001271898	Acox1	3992
NM 001100395	Otof	6907	NM 021600	Chrnd	3003	NM 001271728	Trim2	7211
NM 001283029	Spin1	4395	NM 146116	Tubb4b	1600	NM 001271435	Hagh1	1240
NM 009856	Cd83	2141	NM 001012309	Ccdc55	3132	NM 001290696	Foxj3	4706
NM 146043	Spin1	4455	NM 001009930	Brsk2	4507	NM 001276257	Lcn5	804
NM 001077266	Hnrnpd	6754	NM 013839	Nr1h3	1955	NM 001284427	Smtn	3181
NM 001289559	Pomgnt2	2426	NM 178665	Lpp	15669	NM 001291071	Pfkp	4033
NM 053170	Trim33	8870	NM 001170867	Ncald	3472	NM 001290291	Shisa7	5956
NM 029774	Ttll11	3191	NM 001166585	Tead1	9480	NM 001290697	Nudcd2	1524
NM 001048054	Dusp16	4146	NM 008856	Prkch	3301	NM 001104648	Vmn2r56	2340
NM 010470	Hp1bp3	4852	NM 001159714	Prpf31	3125	NM 001282014	Tyrp1	2740
NM 028472	Bmper	3806	NM 173867	Rcc2	3790	NM 019423	Elovl2	3837
NM 001012518	Ehmt1	5107	NM 146120	Gsn	2663	NM 027959	Pdia6	2125
NM 031875	Otof	7125	NM 001168668	Fam114a2	2608	NM 029344	Acyp2	671
NM 001029936	Specc1	6749	NM 001204276	Hdac7	4387	NM 019973	Son	7287
NM 001285479	Hp1bp3	4849	NM 134129	Prpf19	6161	NM 178880	Son	8451
NM 001289459	Hgf	2827	NM 011630	Nr2c2	7637	NM 010279	Gfra1	4664
NM 019748	Sae1	2017	NM 001252200	Map4k4	5782	NM 008857	Prkci	4465
NM 001109686	Ehmt1	4948	NM 183306	Taok3	4343	NM 021356	Gab1	4877
NM 016812	Banp	5498	NM 008876	Pld2	3801	NM 001276409	Fn1	8077
NM 001289458	Hgf	2838	NM 198932	Pou2f1	12906	NM 130895	Adarb1	6572
NM 001286421	Otof	7065	NM 009437	Tst	1096	NM 008055	Fzd4	3685
NM 001025559	Sox6	8567	NM 001177982	Pde4b	3482	NM 001136067	Ikbkg	6918
NM 001289560	Pomgnt2	2522	NM 009287	Stim1	3609	NM 031191	Prl2c2	790
NM_001289915	Cd83	2134	NM_027041	1700003M02Rik	1716	NM_001276412	Fn1	7792
NM 001039139	Camk2g	3624	NM 012025	Racgap1	3071	NM 028066	F11	2246
NM 178597	Camk2g	3726	NM 033218	Srebf2	4570	NM 001289644	Scgb3a2	549
NM 172665	Pdk1	5202	NM 153508	Clstn3	4007	NM 011265	Rfx3	9187
NM 007516	Hnrnpd	6664	NM 024258	Usp16	2926	NM 009316	Map3k7	5763
NM 011445	Sox6	8715	NM 026342	Fam114a2	2590	NM 008482	Lamb1	5778
NM 001285892	Sae1	1751	NM 207264	BC052040	2752	NM 018753	Ywhab	2775
NM 001289558	Pomgnt2	2526	NM 001253809	Racgap1	2930	NM 177798	Frs2	5701
NM 001080755	Zzz3	7607	NM 011708	Vwf	8834	NM 007858	Diap1	4378
NM 001289460	Hgf	1633	NM 001099634	Myof	7094	NM 001276413	Fn1	7522
NM 182990	Ssrp1	2858	NM 001252058	Ly6c1	707	NM 153552	Thoc1	3855
NM 001109687	Ehmt1	4963	NM 144931	Nae1	1808	NM 001113198	Mitf	4890
NM 008969	Ptgs1	2881	NM 133808	Hdlbp	6238	NM 029933	Bcl9	6102

NM 007594	Calu	3229	NM 001271447	Enox2	3977	NM 001276411	Fn1	7807
NM 030147	Brd8	4730	NM 001039143	Nlrp5	3463	NM 001276408	Fn1	8152
NM 001077267	Hnrnpd	6607	NM 001204280	Hdac7	4084	NM 001161421	Ikbkg	6915
NM 001285863	Pphln1	3900	NM 001162415	Pfkfb2	3689	NM 001146295	Celf4	2258
NM 001287261	Mst1r	1796	NM 016881	Pmm2	1802	NM 009573	Zic1	3350
NM 130447	Dusp16	5112	NM 198007	Ascc3	7477	NM 010681	Lama4	6046
NM 001285478	Hp1bp3	4911	NM 001042527	Blm	4598	NM 001038610	Dach1	5310
NM 001285891	Sae1	1850	NM 001252455	Ptprs	5644	NM 172688	Map3k7	5682
NM 001277328	Sox6	8427	NM 009124	Atxn1	10599	NM 026183	Slc47a1	2634
NM 001025560	Sox6	8681	NM 001198984	Tcof1	4679	NM 025435	Thoc7	991
NM 001077265	Hnrnpd	6811	NM 008364	Il1rap	4576	NM 001024837	Adarb1	6602
NM 001289578	Chfr	2964	NM 001171616	Myt1	5415	NM 007826	Dach1	5466
NM 010427	Hgf	2810	NM 031869	Prkab1	2051	NM 011887	Scn11a	5837
NM 001286663	Ssbp1	2047	NM 001170868	Ncald	3420	NM 172493	Diap2	8456
NM 001080773	Pdpk1	1783	NM 007909	Efna2	2153	NM 053248	Slc5a5	2943
NM 001282061	Rgs6	2345	NM 144897	Apoa1bp	905	NM 009411	Tpbpa	1372
NM 001136081	Ssrp1	2729	NM 177578	Skint3	3627	NM 001161424	Ikbkg	6785
NM_001111015	Syn2	3343	NM_001243192	Serpinb6a	1504	NM_001025067	Lrig2	7134
NM 001277327	Sox6	8722	NM 025697	Spryd7	1690	NM 011101	Prkca	8385
NM 001289580	Chfr	3347	NM 008098	Mtpn	4042	NM 001161423	Ikbkg	6788
NM 053204	Erc1	8760	NM 010278	Gfi1	2862	NM 178590	Ikbkg	6811
NM 176841	Ccdc88a	8862	NM 001145952	Lpp	15462	NM 146191	Lrrk1	7467
NM 133656	Crk	6005	NM 008825	Pfkfb2	7308	NM 007392	Acta2	2572
NM 001277326	Sox6	8662	NM 001170985	Kirrel	7287	NM 001166414	Rfx3	9146
NM 001080809	Cps1	5585	NM 001159647	Cntn1	5817	NM 009756	Bmp10	1317
NM 145375	Tm6sf1	2234	NM 019710	Smc1a	3980	NM 198037	Cachd1	4981
NM 001291282	Tm6sf1	1726	NM 177394	Endov	5164	NM 001285457	Gfra1	4425
NM 009186	Tra2b	2135	NM 001102615	Kif19a	3433	NM 010547	Ikbkg	6814
NM 203280	Sphk2	3552	NM 008696	Map4k4	5620	NM 001285780	Thoc7	1069
NM 001042673	Fancc	3168	NM 007638	Cct7	2452	NM 001276410	Fn1	7882
NM 177472	Slx4	5747	NM 001173550	C5ar1	2540	NM 001289643	Scgb3a2	615
NM 001033263	Agap2	5633	NM 001145902	Arhgap1	2904	NM 008305	Hspg2	14201
NM 001286744	Pacsin1	4156	NM 001163016	Gprasp2	3776	NM 001178049	Mitf	4777
NM 021365	Xlr4b	1304	NM 175475	Cyp26b1	4751	NM 001282992	Pcnt	9476
NM 011652	Ttn	101674	NM 001081308	Taok3	4258	NM 013869	Tnfrsf19	4639
NM_001163757	Skor1	3613	NM_172679	4932438A13Rik	15883	NM_001242368	F10	2693
NM 001163758	Skor1	3597	NM 027382	Hdac8	1724	NM 007972	F10	2503
NM 172717	Chfr	3177	NM 019677	Plcb1	7116	NM 001025305	Tfap2b	6167
NM 013467	Aldh1a1	2032	NM 011294	Sub1	3321	NM 011867	Slc26a4	3083
NM_001005385	Gprasp1	5697	NM_001190445	2610002J02Rik	1076	NM_145991	Cdc73	2692
NM 010916	Nhlh1	2506	NM 021531	Carm1	3231	NM 008481	Lama2	9734
NM 016891	Ppp2r1a	2256	NM 010897	Nf1	11847	NM 001122953	Nfia	9399
NM 009368	Tgfb3	3401	NM 001145779	Kif2a	3753	NM 001278447	Rab18	3688
NM 011605	Tmpo	3821	NM 021280	Plcg1	4435	NM 001081171	Lama5	11404
NM 053110	Gpnmb	3798	NM 001199304	Atxn1	10474	NM 007557	Bmp7	3670
NM 009684	Apaf1	6514	NM 153126	Nat10	3856	NM 001286340	Tfap2b	3831
NM 001283028	Spin1	4530	NM 011078	Phf2	5260	NM 009334	Tfap2b	6132
NM 019971	Pdgfc	3512	NM 001009929	Brsk2	4142	NM 194268	Onecut2	13407
NM_023624	Lrat	5349	NM_001164118	Serpinb6a	1654	NM_008601	Mitf	4581
NM 028351	Rspo3	2411	NM 010949	Numb	3235	NM 010905	Nfia	9528
NM 001004359	Gprasp1	5811	NM 013863	Bag3	2607	NM 029522	Gpsm2	3510
NM 008888	Phox2b	2856	NM 001161667	Acox2	2448	NM 008957	Ptch1	4305
NM 001172561	Sphk2	3481	NM 010956	Ogdh	6630	NM 009745	Bcl7b	1678
NM 013540	Gria2	6841	NM 199299	Jade2	6100	NM 001135559	Sos2	5612
NM 001110100	Banp	5489	NM 007382	Acadm	2062	NM 008516	Lrrn1	3756
NM 001199016	Slc7a9	1702	NM 001244903	Celf1	7796	NM 009807	Casp1	1533
NM 001079830	Trim33	8819	NM 011424	Ncor2	8745	NM 011977	Slc27a1	2795
NM 009347	Tecta	7338	NM 146118	Slc25a25	3342	NM 010233	Fn1	8425

NM 001042558	Apaf1	6557	NM 009211	Smarcc1	5684	NM 001161422	Ikbkg	6736
NM 030168	Rictor	9328	NM 153596	Tmem17	1370	NM 007697	Chl1	7751
NM 138749	Plxnb2	6519	NM 198652	Hjurp	8449	NM 181070	Rab18	3670
NM 001039195	Gria2	3491	NM 001165980	Dcaf17	7739	NM 008787	Pcnt	9530
NM 001284506	Plxnb2	6469	NM 199473	Col8a2	4331	NM 001164155	Tnfrsf19	4036
NM 020011	Sphk2	3790	NM 152801	Arhgef6	4580	NM 001122739	Inppl1	4778
NM 019510	Trpc3	3694	NM 027256	Ints4	3260	NM 021530	Slc4a8	11811
NM 026081	Gprasp1	5900	NM 026079	Ikbkap	6160	NM 026969	Sec31a	4244
NM 031197	Slc2a2	2571	NM 001164791	Zak	6704	NM 001122952	Nfia	9289
NM 001122897	Hp1bp3	4891	NM 031159	Apobec1	2265	NM 007624	Cbx3	1851
NM 001017985	C2cd3	7813	NM 022028	Sav1	2524	NM 010567	Inppl1	5012
NM 001286028	Dlk2	1415	NM 026197	Mettl16	2718	NM 181850	Grm3	3536
NM 011356	Frzb	2920	NM 019826	Ivd	2067	NM 001008791	Whrn	4089
NM 001199015	Slc7a9	1814	NM 144896	Pet112	2307	NM 001003824	Kcnq2	3007
NM 001286452	Daam1	6028	NM 023737	Ehhadh	3010	NM 011043	Pcdh10	4658
NM 008772	P2ry1	3899	NM 001177981	Pde4b	3746	NM 053094	Cd163	4409
NM 001083806	Gria2	6841	NM 001177604	Ak4	4949	NM 013719	Eif2ak4	5220
NM 175460	Nmnat2	4548	NM 001204281	Hdac7	3791	NM 178804	Slit2	8492
NM 030744	Ropn1	1030	NM 008665	Myt1	5529	NM 009988	Cxadr	1753
NM 009780	C4b	5427	NM 080728	Myh7	6054	NM 001291227	Slit2	8555
NM 010406	Hc	5448	NM 001244891	Celf1	7849	NM 001003825	Kcnq2	2992
NM 001017426	Kdm6b	6654	NM 198190	Ntf5	2027	NM 001276263	Cxadr	1386
NM 001163755	Skor1	3616	NM 181819	Wfikkn2	3392	NM 001098170	Pcdh10	6064
NM 028004	Ttn	81931	NM 001253808	Racgap1	2940	NM 053208	Egln2	2124
NM 145575	Cald1	4035	NM 001177890	Cacna1g	8097	NM 009987	Cx3cr1	3753
NM 146128	Dlgap4	4875	NM 001163032	Synpr	2657	NM 001289575	Tsc1	7732
NM 001276763	Brsk2	4113	NM 183181	Abhd16b	1775	NM 010576	Itga4	9833
NM 001159521	Plxnb2	6555	NM 001271449	Enox2	3966	NM 001170395	Cd163	4492
NM 009481	Usp9x	11903	NM 153554	Aldh18a1	3521	NM 008974	Ptp4a2	3413
NM 172446	Skor1	3669	NM 028866	Wdr33	6206	NM 001006674	Kcnq2	2454
NM 001281818	Specc1	6966	NM 001167898	Frem3	6626	NM 001025192	Cxadr	5566
NM 021291	Slc7a9	1929	NM 027990	Lypd6b	2285	NM 001006669	Kcnq2	2899
NM 016702	Agxt	1553	NM 001112813	Cacna1g	8169	NM 174854	Disc1	2597
NM 133948	Psip1	3255	NM 027034	Izumo3	1263	NM 028640	Whrn	4056
NM 001103157	Steap2	10696	NM 001037761	Capzb	1789	NM 001006678	Kcnq2	1274
NM 001126331	Trp73	4468	NM 027151	Dctn2	1918	NM 027742	Lrrfip2	3215
NM 001285808	Dtna	6231	NM 183288	Arhgap27	2099	NM 010585	Itpr1	9877
NM 016976	Grm1	6922	NM 001276317	Uba1	4068	NM 001038619	Dnm3	7534
NM 021415	Cacna1h	8240	NM 021475	Adamdec1	2412	NM 011578	Tgfbr3	6074
NM 001103156	Steap2	10341	NM 001164528	Ildr2	8260	NM 010680	Lama3	10343
NM 001285807	Dtna	3954	NM 198683	Celf1	4721	NM 011569	Tekt1	1411
NM 010928	Notch2	10506	NM 178634	Csrnp3	10510	NM 174853	Disc1	2408
NM 145830	Ehmt2	4070	NM 001168318	Scara5	3296	NM 010229	Flt3	3664
NM 001110796	Pclo	16895	NM 199469	Nploc4	4589	NM 001164745	Ptp4a2	3382
NM 001163676	Abcc4	5504	NM 001204278	Hdac7	4228	NM 001006680	Kcnq2	2291
NM 008855	Prkcb	8830	NM 134188	Acot2	2215	NM 011394	Slc20a2	3658
NM 001111276	Igf1	6987	NM 001252202	Map4k4	5542	NM 001164838	Lrrfip2	3260
NM 001163691	Cacna1h	8187	NM 001243712	Itch	5389	NM 001006675	Kcnq2	2939
NM 009700	Aqp4	5082	NM 001136075	Numb	3382	NM 001278256	Prnp	3732
NM 008302	Hsp90ab1	2507	NM 001199305	Atxn1	10623	NM 001282006	Tekt1	1459
NM 001285817	Dtna	6210	NM 001171615	Myt1	5305	NM 001177806	Eif2ak4	4916
NM 011642	Trp73	5046	NM 001164231	Pwwp2a	4096	NM 029062	Pihld3	1449
NM 001285810	Dtna	6219	NM 001164636	Endov	5112	NM 023868	Ryr2	16813
NM 024449	Sost	2066	NM 053115	Acox2	2573	NM 001081147	Oxtr	4568
NM 001285470	Steap2	10215	NM 025918	Ccdc43	2307	NM 011132	Pole	7167
NM_001033336	Abcc4	5729	NM_177674	2010015L04Rik	3019	NM_001282007	Tekt1	1408
NM 177704	Sytl5	6623	NM 008839	Pik3ca	8917	NM 009571	Zfy2	2816
NM 001290675	Scn9a	9832	NM 028698	Ccdc30	1073	NM 001291228	Slit2	8504
NM 011171	Procr	1504	NM 144520	Sec14l2	2529	NM 001289704	Cflar	6943
NM 001285813	Dtna	6376	NM 145951	Enox2	3921	NM 001289576	Tsc1	7714
NM 054098	Steap4	3140	NM 008792	Pcsk2	4766	NM 001008793	Whrn	4053

NM 022563	Ddr2	8306	NM 153543	Aldh1l2	6030	NM 011104	Prkce	6254
NM 001003908	Cltc	6178	NM 146214	Tat	2377	NM 178642	Ano1	4614
NM 019454	Dll4	3451	NM 009551	Zfand5	7385	NM 011170	Prnp	2191
NM 001165989	Ckap5	6584	NM 007550	Blm	4869	NM 019736	Acot9	1548
NM 010512	Igf1	7121	NM 024472	Gltpd1	2662	NM 022887	Tsc1	7700
NM 001285469	Steap2	10250	NM 001145830	Plcb1	6998	NM 001006668	Kcnq2	2971
NM 001126330	Trp73	4756	NM 001252109	Cadps2	1512	NM 001098171	Pcdh10	8263
NM 011995	Pclo	20099	NM 001252282	Ogdh	6675	NM 001006676	Kcnq2	1864
NM 147151	Ehmt2	3805	NM 011137	Pou2f1	12978	NM 001006677	Kcnq2	2059
NM 001111274	Igf1	7039	NM 010438	Hk1	4100	NM 178591	Nrg1	2103
NM 019521	Gas6	2594	NM 019641	Stmn1	1070	NM 011643	Trpc1	3014
NM 018789	Foxo4	3162	NM 009783	Cacna1g	8238	NM 207653	Cflar	7232
NM 008973	Ptn	2021	NM 009346	Tead1	9899	NM 009865	Cdh10	3405
NM 001290422	Ccnc	3485	NM 001033320	Rltpr	2504	NM 010305	Gnai1	3193
NM 008260	Foxa3	2039	NM 153540	Pomgnt2	2369	NM 001006679	Kcnq2	1734
NM 001290625	Mapkap1	3126	NM 013716	G3bp1	2685	NM 001098172	Pcdh10	4241
NM 029437	Ckap5	6521	NM 170592	Ntmt1	1217	NM 011056	Pde4d	7049
NM 033270	E2f6	2446	NM 001252453	Ptprs	5656	NM 001123362	Prdm12	2471
NM 001111275	Igf1	7069	NM 021458	Fzd3	12742	NM 010611	Kcnq2	2777
NM 176930	Nrcam	7572	NM 001113326	Msr1	1467	NM 008070	Gabrb2	7568
NM_001083927	Tle3	5202	NM_009254	Serpinb6a	1432	NM_001242349	Ano1	4605
NM 009389	Tle3	5172	NM 172288	Nup133	5808	NM 009370	Tgfbr1	5735
NM 001114333	Grm1	7007	NM 001252201	Map4k4	5581	NM 001008792	Whrn	4068
NM 001083928	Tle3	5148	NM 001190453	Dctn2	1924	NM 145967	Vstm2a	3629
NM 001163675	Abcc4	5711	NM 001195023	Nploc4	4685	NM 009946	Cplx2	4928
NM 001127318	Gucy2c	3951	NM 024272	Ssbp2	6656	NM 025323	Sf3b6	1133
NM 207650	Dtna	6332	NM 001167909	Nme8	1704	NM 001291452	Lrrc7	7321
NM 026972	Cd209b	2068	NM 008448	Kif5b	6030	NM 001081358	Lrrc7	7462
NM 026697	Rab14	3100	NM 001163015	Gprasp2	3814	NM 177006	Nwd2	8539
NM 145067	Gucy2c	3879	NM 198933	Pou2f1	12978	NM 001291453	Lrrc7	7184
NM 001037800	Cd209b	1978	NM 007906	Eef1a2	2111	NM 029852	Cep83	3006
NM 028734	Steap2	10398	NM 011860	Nlrp5	3511	NM 001284219	Agtpbp1	4488
NM 007809	Cyp17a1	1841	NM 028242	Htatsf1	2855	NM 001284218	Agtpbp1	3065
NM 009897	Ckmt1	1589	NM 007577	C5ar1	2481	NM 001284221	Agtpbp1	4364
NM 001146031	Nrcam	7362	NM 008972	Ptma	1192	NM 001048008	Agtpbp1	2940
NM 153413	Dock3	9067	NM 001166584	Tead1	9962	NM 023328	Agtpbp1	4417
NM 001290393	Sntg1	7213	NM 001252456	Ptprs	5618	NM 009994	Cyp1b1	5128
NM 021396	Pdcd1lg2	3415	NM 001199685	Taok3	4366	NM 133972	Armc6	2215
NM 008823	Cfp	1524	NM 001033313	Pdap1	2203	NM 001081249	Vcan	12432
NM 008818	Rhox5	869	NM 176847	Ush1g	3183	NM 010016	Cd55	2527
NM 001290986	Wdr76	4246	NM 001168667	Fam114a2	2587	NM 009072	Rock2	8012
NM 007522	Bad	1458	NM 001177888	Cacna1g	8148	NM 007424	Acan	7355
NM 001040112	Arap1	5314	NM 146255	Slc1a7	3005	NM 009697	Nr2f2	4216
NM 001290477	Klhl4	3866	NM 009199	Slc1a1	3708	NM 183261	Nr2f2	4164
NM 001290501	Znrf3	7199	NM 009157	Map2k4	3694	NM 007619	Cbl	5083
NM 001290733	Osbpl6	8301	NM 008867	Pla2r1	7112	NM 008045	Fshb	1622
NM 001284328	Syncrip	6930	NM 153141	Carm1	3151	NM 001017525	Btbd11	3955
NM 001290537	Otud5	3879	NM 031195	Msr1	3643	NM 026792	Agpat5	3829
NM 001290455	Gripap1	3021	NM 001081081	Gls	4974	NM 008343	Igfbp3	2456
NM 001289787	Slc26a5	4430	NM 001177713	Cyp26b1	4700	NM 199197	Rbfa	1361
NM 001271678	Wnk3	10400	NM 011581	Thbs2	5922	NM 007758	Cr2	6207
NM 001286662	Pdpk1	7078	NM 001190454	Dctn2	1933	NM 009284	Stat6	3805
NM 001286040	Anks1	7092	NM 029870	Crebrf	7455	NM 012013	Figla	759
NM 001285994	Enpp2	3536	NM 001170976	Dbndd1	1344	NM 007831	Dcc	10325
NM 001290716	Suv39h1	3097	NM 001177980	Pde4b	4206	NM 010684	Lamp1	2265
NM 001291184	Pcsk6	4259	NM 011506	Sucla2	2108	NM 026152	Hoga1	1635
NM 001282947	Apaf1	6497	NM 130867	Kirrel	7284	NM 031404	Actl6b	1577
NM 001291155	Tomm34	1940	NM 017368	Celf1	4873	NM 133353	Oosp1	873
NM 001290691	Col9a1	3097	NM 011552	Tcof1	4571	NM 152944	Mmp21	1858
NM 001285997	Prc1	3040	NM 025816	Tax1bp1	3307	NM 198029	Fermt1	5255
NM 001285860	Sgip1	4944	NM 153409	Csrnp3	10672	NM 175502	Tmem74	1472

NM 001285872	Pafah2	3194	NM 001044383	Ubtf	4458	NM 009292	Stra8	1455
NM 001290390	Sntg1	7264	NM 001252287	Ogdh	6706	NM 009983	Ctsd	1979
NM 001291055	Lgals8	2522	NM 001165942	Nsun6	2420	NM 001001932	Eea1	7836
NM 001285983	Banp	5372	NM 011384	Six6	3956	NM 001081131	Dhtkd1	3535
NM 001252513	Lipi	2082	NM 001271448	Enox2	3822	NM 001011707	Cyp2c66	1630
NM 001285963	Skint2	1258	NM 027328	Prpf31	3143	NM 134013	Psme4	6454
NM 001291167	Apba2	3283	NM 010168	F2	1988	NM 001081290	Prrc2c	10444
NM 001277286	Ank1	8191	NM 146109	Als2	2977	NM 175513	Zfp804a	4196
NM 001291057	Lgals8	2718	NM 001204275	Hdac7	4339	NM 172621	Clic5	5870
NM_001282994	Cobl	5464	NM_028504	1700034F02Rik	2185	NM_027432	Wdr77	3553
NM 001290633	Reps2	7695	NM 198934	Pou2f1	13050	NM 001033345	Catip	2101
NM 001277186	Dlgap4	4866	NM 033041	Hes7	964	NM 028291	Pan3	4628
NM 001290667	Chac2	1294	NM 031377	Pramel1	2440	NM 001252327	Pan2	4369
NM_001290476	Klhl13	3232	NM_001163521	1700034F02Rik	2152	NM_001252326	Pan2	4423
NM 001285453	Bad	941	NM 001267621	Gfi1	2650	NM 133992	Pan2	4450
NM 001290320	Slc26a8	3674	NM 001177730	Nr1h3	1802	NM 001001602	Dab2ip	6424
NM 001285998	Prc1	2972	NM 028868	Cxxc1	2537	NM 001099644	Htr3a	2071
NM 001290420	Ccnc	1500	NM 001177602	Ak4	5136	NM 001127260	Trp63	5288
NM 001287211	Cd209b	2041	NM 011695	Vdac2	1662	NM 008216	Has2	4262
NM 175272	Nav2	11332	NM 011218	Ptprs	6874	NM 008591	Met	6652
NM 001286041	Anks1	7029	NM 011221	Purb	8478	NM 009974	Csnk2a2	3766
NM 007566	Birc6	15787	NM 001145898	BC052040	2770	NM 013881	Ulk2	5813
NM 001290630	Rbm41	5611	NM 015812	Rgs6	2626	NM 001127264	Trp63	4707
NM 001289510	Tecpr2	7793	NM 001177605	Ak4	4927	NM 001134474	Vcan	7215
NM 001290541	Acer2	4068	NM 009647	Ak4	4894	NM 001127259	Trp63	5382
NM 001290389	Atp1b4	4266	NM 001204279	Hdac7	4156	NM 016697	Gpc3	2272
NM 177715	Kctd12	6076	NM 144557	Myrip	4762	NM 001013779	Aim2	2541
NM 001290448	Nfx1	4368	NM 021336	Snrpa1	1250	NM 027286	Ace2	3418
NM 001290369	Nmt2	4411	NM 016925	Fanca	4541	NM 013919	Usp21	2200
NM 001271810	Pcdh11x	8607	NM 012027	Mprip	8784	NM 133957	Nfat5	13451
NM 001289569	Rpap2	2336	NM 001159319	Cacnb1	1738	NM 009654	Alb	2043
NM 001289514	Tbc1d1	4802	NM 001286664	Ssbp1	2047	NM 009509	Vil1	3117
NM 001291066	Adam8	3055	NM 001024955	Pik3r1	5839	NM 001002268	Gpr126	6505
NM 001290982	Myo1b	4879	NM 001077694	Dysf	6845	NM 178594	Vtcn1	2622
NM 001277284	Ank1	8055	NM 001289579	Chfr	3253	NM 001033874	Ak8	1584
NM 001276764	Dst	24271	NM 001159320	Cacnb1	1600	NM 013561	Htr3a	2089
NM 001289735	Nxf2	2395	NM 007985	Fancc	3134	NM 009621	Adamts1	4904
NM 001291069	Golim4	4551	NM 008155	Gpi1	2887	NM 001005509	Eif2a	2322
NM 001290514	Mospd1	2251	NM 030880	Pacsin3	1860	NM 008356	Il13ra2	1558
NM 001291190	Ssh2	9176	NM 001100395	Otof	6907	NM 007808	Cycs	3059
NM 177408	Gabrg2	3911	NM 031173	Cacnb1	1873	NM 001002894	Nlrp14	3297
NM 001287139	Zzz3	7546	NM 016903	Esd	1988	NM 018823	Nfat5	13389
NM 030706	Trim2	7207	NM 001282977	Cacnb1	3524	NM 009511	Vipr2	3403
NM 001286729	Glcci1	5525	NM 001289559	Pomgnt2	2426	NM 011264	Rev3l	10666
NM 001277289	Ank1	8269	NM 053170	Trim33	8870	NM 008626	Mrc2	5801
NM 001290503	Zer1	4241	NM 001170966	Wdr33	2548	NM 018729	Cd244	3758
NM 001277756	Cav2	1116	NM 001289678	Pacsin3	1864	NM 177600	Ccdc73	3944
NM 001290536	Otud5	4262	NM 001048054	Dusp16	4146	NM 011613	Tnfsf11	2243
NM 001290496	Trub2	3866	NM 028472	Bmper	3806	NM 001136104	Abl2	10402
NM 001271726	Trim2	7144	NM 020508	Brd4	5948	NM 178357	Klf11	3982
NM 001290642	Rc3h2	8962	NM 008828	Pgk1	1840	NM 010123	Eif3a	5176
NM 001040434	Rgag1	4142	NM 001283048	Tmpo	3294	NM 001163018	Suz12	4380
NM 001290747	Tbx22	3073	NM 201232	Nipbl	10762	NM 011770	Ikzf2	9457
NM 001290713	Stag2	5814	NM 031875	Otof	7125	NM 199196	Suz12	4449
NM 001276248	Cp	4561	NM 001025363	Klc1	2406	NM 010555	Il1r2	1360
NM 001290524	Mospd2	2706	NM 001289677	Pacsin3	1865	NM 138745	Mthfd1	3324
NM 001286743	Pacsin1	4250	NM 001081146	Prickle2	7799	NM 020008	Clec7a	2277
NM 001290708	Smarca1	4024	NM 001029936	Specc1	6749	NM 001114125	Dab2ip	5388
NM_001285885	Zmym6	5036	NM_001080746	Gtf2i	4457	NM_175128	4930430F08Rik	2419

NM 001277187	Dlgap4	4824	NM 001159641	Taspl	2148	NM 001127262	Trp63	4625
NM 001285831	Far1	4339	NM 001159640	Taspl	2259	NM 009951	Igf2bp1	8382
NM 001277219	Crk	5835	NM 001114665	Fnbp1l	5377	NM 011641	Trp63	4710
NM 001290337	Prickle4	1392	NM 028733	Pacsin3	1994	NM 027102	Esam	1859
NM 001284372	Atf2	4006	NM 001080130	Tmpo	3491	NM 011499	Strap	2644
NM 001290674	Scn9a	9865	NM 145121	Cacnb1	3389	NM 178763	Zfp750	3254
NM 001286033	Stx2	2812	NM 021469	Dysf	6663	NM 172132	Kdm4b	4610
NM 001290523	Mospd2	2487	NM 001134461	Prickle2	7695	NM 053079	Slc15a1	3123
NM 001271568	Cdc7	2851	NM 001286421	Otof	7065	NM 009595	Abl2	10714
NM 001291158	Zmynd8	7345	NM 001286654	Rcan2	3107	NM 023662	Pcm1	8398
NM 001290527	Psip1	1903	NM 001025559	Sox6	8567	NM 001130513	Ace2	3566
NM 001272076	Trim24	5771	NM 001289560	Pomgnt2	2522	NM 001122756	Corin	4733
NM 001290991	Ctdspl2	6734	NM 001282978	Cacnb1	1714	NM 016869	Corin	4890
NM 001291077	Snph	4543	NM 027707	Nipbl	10216	NM 011065	Per1	4663
NM 001291042	Tmem220	1597	NM 172665	Pdk1	5202	NM 172955	Vcan	3745
NM 001290654	Ahsa2	3692	NM 011445	Sox6	8715	NM 024215	Zfp593	1991
NM 001290745	Nckap1	4421	NM 001289558	Pomgnt2	2526	NM 178753	Spin4	4176
NM 001290526	Nhs	5382	NM 172203	Nox1	2563	NM 010570	Irs1	9163
NM 001290771	Tro	3350	NM 001080755	Zzz3	7607	NM 028709	Btbd11	5808
NM 001286573	Ehmt2	3968	NM 182990	Ssrp1	2858	NM 001159367	Per1	4648
NM 001276250	Cp	4712	NM 001134391	Apobec1	2209	NM 009867	Cdh4	6393
NM 001271567	Cdc7	2872	NM 153118	Fnbp1l	5203	NM 001281859	Cysltr1	2960
NM 001110855	Crem	2010	NM 001080132	Tmpo	3284	NM 001122997	Dnmt3b	4229
NM 001271504	Crem	2534	NM 001025358	Klc1	2430	NM 172932	Nlgn3	3899
NM 001024849	Gm14325	4396	NM 130447	Dusp16	5112	NM 010825	Meis2	4621
NM 001291150	Ptprt	12129	NM 001289845	Palb2	1590	NM 001256195	Eif4g3	6241
NM 001271679	Wnk3	10259	NM 001277328	Sox6	8427	NM 001286718	Tsc2	6134
NM 001290786	Muc15	3222	NM 001025560	Sox6	8681	NM 001159567	Meis2	4737
NM 001290546	Phf6	4134	NM 175225	Taspl	2526	NM 009622	Adcy1	12259
NM 001285887	Zmym6	3953	NM 010365	Gtf2i	4400	NM 019471	Mmp10	1734
NM 001290628	Vps54	4533	NM 001286653	Rcan2	3230	NM 016971	Il22	1088
NM 001290659	Tanc1	7763	NM 001286663	Ssbp1	2047	NM 001003963	Dnmt3b	4152
NM 001276455	Slc19a2	3457	NM 001282061	Rgs6	2345	NM 001080971	Tubb1	2004
NM 001277167	Gm12429	4146	NM 133666	Ndufv1	1608	NM 019786	Tbk1	3031
NM 001284189	Homer1	4768	NM 001025361	Klc1	2458	NM 001271747	Dnmt3b	3977
NM 001291054	Mcm8	3481	NM 001136081	Ssrp1	2729	NM 001112703	Abl1	7162
NM 001284270	Tmem201	3737	NM 001077495	Pik3r1	6928	NM 153410	Gpsm1	3389
NM 001276467	Asap1	6110	NM 001277327	Sox6	8722	NM 008361	Il1b	1328
NM 001290428	Frmpd4	8156	NM 001289843	Palb2	3253	NM 001199147	Gpsm1	3458
NM 001285995	Enpp2	3605	NM 008525	Alad	4536	NM 011647	Tsc2	6266
NM_001272064	Trim24	6089	NM_001277326	Sox6	8662	NM_026088	1700022I11Rik	3726
NM 001276681	Syt6	4416	NM 001080809	Cps1	5585	NM 001033420	Dock1	6815
NM 001289577	Chfr	3180	NM 133780	Tpr	7550	NM 001172095	Kdm4c	4207
NM 001290644	Rp2h	4581	NM 007491	Art5	1715	NM 007389	Chrna1	4320
NM 001271450	Enox2	3933	NM 001291349	Anapc15	800	NM 001003961	Dnmt3b	4341
NM 001285459	Fmn1	11147	NM 001291352	Anapc15	843	NM 007616	Cav1	2542
NM 001271505	Crem	2612	NM 001291353	Anapc15	843	NM 010796	Clec10a	1498
NM 001276462	Asap1	6181	NM 027532	Anapc15	885	NM 013862	Rabgap1l	3373
NM 001291076	Snph	4651	NM 001291348	Anapc15	850	NM 001039363	Tsc2	6137
NM 001282443	Mroh6	3372	NM 001291354	Art5	1662	NM 001283045	Abl1	6039
NM 001291049	Usp43	4447	NM 021355	Fmod	2859	NM 008378	Impact	3432
NM 001290370	Nmt2	4368	NM 203280	Sphk2	3552	NM 001037999	Dbi	593
NM 001290387	Pdp1	4149	NM 001042673	Fancc	3168	NM 173398	Gpr171	2225
NM 198958	Nox3	1792	NM 177472	Slx4	5747	NM 001256198	Eif4g3	6069
NM 001290385	Arhgef9	5532	NM 022331	Herpud1	1871	NM 194262	Arid4b	6113
NM 001277941	Rad51d	6937	NM 001033263	Agap2	5633	NM 001243064	Cav1	2638
NM 001290728	Sytl5	6557	NM 001282943	Ccne2	3026	NM 001199146	Gpsm1	3209
NM 001277170	Rbm46	3586	NM 011652	Ttn	101674	NM 001199676	Camkk2	4903
NM 001193266	Mdga2	9834	NM 001163757	Skor1	3613	NM 001159570	Meis2	4989
NM 001271727	Trim2	7413	NM 001163758	Skor1	3597	NM 025887	Rab5a	2364
NM 001282093	Wac	5058	NM 001081211	Ptafr	1820	NM 010000	Cyp2b9	1877

NM 001277221	Crk	5469	NM 013467	Aldh1a1	2032	NM 172142	Nfkbid	2010
NM 001290737	Thg1l	3740	NM 001005385	Gprasp1	5697	NM 001271745	Dnmt3b	4166
NM 001285507	Cops2	3353	NM 148935	Foxn4	2970	NM 001003960	Dnmt3b	4281
NM 001286158	Mllt3	4730	NM 001160317	Nfasc	9597	NM 010638	Klf9	3263
NM 001284196	Srsf10	3433	NM 001025362	Klc1	1928	NM 001136072	Meis2	4716
NM 001289607	Brd8	4520	NM 053110	Gpnmb	3798	NM 019687	Slc22a4	2260
NM 001290814	Aff3	7251	NM 001164081	Timeless	4479	NM 001276328	Ms4a2	2648
NM 001290485	Lamp2	2206	NM 001080129	Tmpo	3611	NM 001281862	Cysltr1	2802
NM 001290378	Ap1s2	2217	NM 019971	Pdgfc	3512	NM 001039153	Tctn1	4087
NM 001277847	Treh	1943	NM 023624	Lrat	5349	NM 001025074	Ntrk2	8744
NM 001110854	Crem	2046	NM 001004359	Gprasp1	5811	NM 001195046	Pak3	8242
NM 001111016	Nav2	11070	NM 001276446	Alad	4447	NM 144787	Kdm4c	4271
NM 001271451	Enox2	3918	NM 001083334	Bin1	2135	NM 021476	Cysltr1	2979
NM 001290435	Gabrq	4943	NM 001160318	Nfasc	9647	NM 001159568	Meis2	4642
NM 184088	Dennd4c	7793	NM 001172561	Sphk2	3481	NM 001286720	Tsc2	6353
NM 001291211	Pcmtd2	3572	NM 013540	Gria2	6841	NM 172703	Eif4g3	6145
NM 001282016	P2ry1	3624	NM 001012236	Trex1	1060	NM 001283046	Abl1	7367
NM 001276463	Asap1	6145	NM 011183	Psen2	2026	NM 009594	Abl1	5933
NM 001291039	Cds2	8089	NM 001079830	Trim33	8819	NM 001286714	Tsc2	6261
NM 010081	Dst	8788	NM 009347	Tecta	7338	NM 007830	Dbi	587
NM 001277281	Ank1	7980	NM 001134460	Prickle2	8200	NM 001159569	Meis2	5010
NM 001284314	Ube2j2	3139	NM 138579	Triobp	7214	NM 008628	Msh2	3056
NM 001290397	Mybl1	4804	NM 001039156	Triobp	7352	NM 001282961	Ntrk2	8711
NM 001286575	Ehmt2	3907	NM 177350	Gldn	4649	NM 015743	Nr4a3	1884
NM 001290801	Mgat4a	7172	NM 001285776	Serpinh1	2313	NM 019397	Egfl6	2700
NM 001286544	Ccpg1	3087	NM 030168	Rictor	9328	NM 145358	Camkk2	4860
NM 001272055	Numb	3349	NM 138749	Plxnb2	6519	NM 001001979	Megf10	7514
NM 001290706	Tmem54	1148	NM 001039195	Gria2	3491	NM 001271746	Dnmt3b	4037
NM 001290410	Nkain3	4668	NM 001284506	Plxnb2	6469	NM 001195048	Pak3	8352
NM 001291104	Fgf11	2534	NM 020011	Sphk2	3790	NM 001286716	Tsc2	6132
NM 001290449	Nfx1	4899	NM 008198	Cfb	2767	NM 026821	Lurap1l	1699
NM 001277280	Ank1	7794	NM 011618	Tnnt1	1027	NM 183138	Tet3	10907
NM 001271883	Vmn2r124	2568	NM 026081	Gprasp1	5900	NM 001286713	Tsc2	6335
NM 001290741	Cnot6	5372	NM 007657	Cd9	1306	NM 001039556	Rad54b	2925
NM 001271725	Trim2	7108	NM 001136082	Timeless	4476	NM 001276329	Ms4a2	2429
NM 001287530	Arhgap6	4147	NM 031197	Slc2a2	2571	NM 001276330	Ms4a2	2660
NM 001276461	Asap1	6101	NM 001017985	C2cd3	7813	NM 007818	Cyp3a11	2053
NM 001282993	Cobl	5558	NM 145541	Rap1a	2474	NM 010921	Nkx3-1	3137
NM 001291002	Galk2	2523	NM 011255	Rbp4	930	NM 010068	Dnmt3b	4092
NM 001285494	Gnb1l	3525	NM 182716	Nfasc	9795	NM 022434	Cyp4f14	2225
NM 001271772	Skil	6609	NM 001142706	Cfb	2763	NM 021887	Il21r	2607
NM 001290443	Spaca1	1177	NM 009158	Mapk10	7203	NM 001271744	Dnmt3b	4226
NM 001290734	Osbpl6	7968	NM 001159487	Rbp4	1243	NM 011638	Tfrc	4920
NM 001290684	Shroom2	7501	NM 001083806	Gria2	6841	NM 008003	Fgf15	1809
NM 001271578	Sec1	2470	NM 175460	Nmnat2	4548	NM 008778	Pak3	8225
NM 001291166	Apba2	3337	NM 001081567	Mapk10	7198	NM 172473	Hace1	3785
NM 001290308	Col12a1	11719	NM 134157	Atp6v1b1	1945	NM 001195049	Pak3	8616
NM 001290283	Pld6	1773	NM 011634	Traip	2697	NM 001204252	Clec10a	1501
NM 001277340	Aldoart2	1692	NM 001080131	Tmpo	3395	NM 001198809	Sfmbt2	1901
NM 001284354	Shprh	6832	NM 001024716	Triobp	2568	NM 013516	Ms4a2	2759
NM 153319	Amot	6974	NM 009780	C4b	5427	NM 001283047	Abl1	7306
NM 001290627	Atp4a	3497	NM 001014976	Espl1	6630	NM 001195047	Pak3	8197
NM 001290472	Rnf185	3389	NM 011637	Trex1	1074	NM 016696	Gpc1	3552
NM 001286824	Uspl1	3730	NM 021423	Shank3	7131	NM 008625	Mrc1	5322
NM 001289606	Brd8	4511	NM 011589	Timeless	4422	NM 001289798	Hadhb	2092
NM 001276502	Vps13d	15762	NM 001017426	Kdm6b	6654	NM 001289799	Hadhb	2088
NM 001290391	Pdp1	4184	NM 001164080	Timeless	4476	NM 001033239	Csta1	2731
NM 001290379	Ap1s2	2226	NM 001163755	Skor1	3616	NM 026817	Rab12	1650
NM 001290788	Aqr	4414	NM 028004	Ttn	81931	NM 030554	Rab27b	6977
NM 001276359	Camsap1	7996	NM 001276763	Brsk2	4113	NM 016858	Rab33b	3488
NM 001282942	Fancc	2937	NM 025350	Cpa1	1578	NM 001082553	Rab27b	6876
NM 001290384	Arhgef9	4708	NM 001159521	Plxnb2	6555	NM 001033181	Jrkl	2952

NM 001272056	Numb	3432	NM 009481	Usp9x	11903	NM 001291748	Xlr	1860
NM 001286972	Dnajc19	1871	NM 172446	Skor1	3669	NM 001291768	Tfdp1	2456
NM 001271506	Crem	2723	NM 010111	Efnb2	4319	NM 001291766	Tfdp1	2517
NM 001285859	Sgip1	5004	NM 009668	Bin1	2468	NM 001291787	Map4k2	4653
NM 001291134	Rprd1b	4520	NM 009388	Tkt	3242	NM 009006	Map4k2	4679
NM 001291135	Rprd1b	4616	NM 001281818	Speccl	6966	NM 007771	Cry1	3035
NM 001290988	Trim11	2327	NM 001160316	Nfasc	9406	NM 001033960	Rabgap1	4238
NM 001291149	Ptprt	12159	NM 001128605	Psen2	2017	NM 011564	Sry	1188
NM_001290552	Swi5	635	NM_001103157	Steap2	10696	NM_029432	4930402H24Rik	4975
NM 001286728	Glcci1	5528	NM 033562	Derl2	3668	NM 010581	Cd47	1928
NM 001290669	Ttc21b	4402	NM 001081278	Tbc1d4	6641	NM 144860	Mib1	3793
NM 001286100	Fam65b	5466	NM 001285808	Dtna	6231	NM 133786	Smc4	4101
NM 172997	Idua	4289	NM 021415	Cacna1h	8240	NM 027184	Ipmk	5432
NM 001277942	Rad51d	6910	NM 001079847	Gpr64	4672	NM 028307	Tdrkh	2665
NM 001290437	Inpp5e	3855	NM 001103156	Steap2	10341	NM 008866	Lypla1	2447
NM 001286545	Ccpg1	2962	NM 001285807	Dtna	3954	NM 145953	Cth	1815
NM 001285981	Banp	5381	NM 016721	Iqgap1	7377	NM 177864	Skint9	1373
NM 001284373	Atf2	3943	NM 010928	Notch2	10506	NM 018826	Irx5	2421
NM 001271728	Trim2	7211	NM 145830	Ehmt2	4070	NM 145987	Tmem82	2066
NM 001271563	Armc3	2968	NM 001110796	Pclo	16895	NM 021308	Piwil2	4913
NM 001281926	Zmynd8	5079	NM 001163676	Abcc4	5504	NM 015766	Ebi3	1187
NM 001195413	Cngb1	6201	NM 008855	Prkcb	8830	NM 177239	Mysm1	7420
NM 001290350	Zfp37	3597	NM 001163691	Cacna1h	8187	NM 010270	Mrps33	1061
NM 001290315	Igdcc4	6230	NM 010788	Mecp2	10233	NM 008324	Ido1	1555
NM 029615	Med27	1709	NM 008302	Hsp90ab1	2507	NM 010544	Ihh	2464
NM 001290291	Shisa7	5956	NM 001285817	Dtna	6210	NM 018744	Sema6a	6901
NM 001083916	Kdf1	1804	NM 001290729	Taf1	8033	NM 177905	Piwil4	2637
NM 019776	Snd1	3482	NM 001290827	Cpeb3	5908	NM 001039243	Erich4	910
NM 133707	Kdf1	1745	NM 011642	Trp73	5046	NM 177572	Rimkla	4093
NM 019423	Elovl2	3837	NM 001285810	Dtna	6219	NM 001081240	Prmt10	2781
NM 001195730	Dcdc2b	864	NM 001285470	Steap2	10215	NM 007757	Cpox	3186
NM 175441	Mylk3	3070	NM 007836	Gadd45a	1224	NM 001168297	Fbxo30	4943
NM 019973	Son	7287	NM 001033336	Abcc4	5729	NM 027968	Fbxo30	4977
NM 178880	Son	8451	NM 001136068	Klrc1	1993	NM 001077354	C77370	10891
NM 145531	Spg11	7665	NM 177704	Sytl5	6623	NM 027664	Rimklb	4017
NM 001198560	H2-Q7	1419	NM 001290675	Scn9a	9832	NM 207215	Mycbp2	15269
NM 133806	Uap1	2283	NM 001285813	Dtna	6376	NM 001195748	Rgs22	4008
NM 010394	H2-Q7	1536	NM 007783	Csk	2292	NM 016807	Sdcbp	2550
NM 177378	Rnf150	9685	NM 022563	Ddr2	8306	NM 145423	Slc5a8	5346
NM 012012	Exo1	5506	NM 001288664	Klrc1	1891	NM 011125	Pltp	1806
NM 008857	Prkci	4465	NM 001081091	Cep152	5768	NM 001039521	Rrn3	3607
NM 021356	Gab1	4877	NM 001165989	Ckap5	6584	NM 001113180	Gria4	5458
NM 008695	Nid2	4913	NM 001290445	Gpr64	4675	NM 009722	Atp2a2	4353
NM 130895	Adarb1	6572	NM 001285469	Steap2	10250	NM 144534	Tmem38a	2109
NM 133195	Celf4	3947	NM 001126330	Trp73	4756	NM 007993	Fbn1	9900
NM 001170983	Hnrnpc	2784	NM 001290666	Csrnp3	10266	NM 007409	Adh1	1348
NM 008055	Fzd4	3685	NM 011995	Pclo	20099	NM 001111051	Dclk1	6956
NM 012061	Cadps	5478	NM 147151	Ehmt2	3805	NM 009538	Plagl1	5279
NM 001042502	Pitx2	2317	NM 008396	Itga2	4235	NM 144873	Uhrf2	3595
NM 001174074	Celf4	3863	NM 001002842	Pram1	2290	NM 007664	Cdh2	4646
NM 009375	Tg	8462	NM 033324	Dgcr8	4226	NM 008605	Mmp12	3607
NM 011607	Tnc	7100	NM 001290422	Ccnc	3485	NM 001111099	Cdkn1a	1936
NM 001190704	Dlk1	4120	NM 001290625	Mapkap1	3126	NM 011416	Smarca2	5831
NM 139218	Dppa3	818	NM 027444	Bbx	8742	NM 024448	Rab12	2010
NM 007633	Ccne1	2022	NM 001079857	Gpr64	4681	NM 011035	Pak1	3081
NM_001289644	Scgb3a2	549	NM_029437	Ckap5	6521	NM_153117	9530068E07Rik	2467
NM 011265	Rfx3	9187	NM 176930	Nrcam	7572	NM 138952	Ripk2	1923
NM 009316	Map3k7	5763	NM 001083927	Tle3	5202	NM 001081309	Pik3r4	4884
NM 008482	Lamb1	5778	NM 011345	Sele	2912	NM 177059	Fstl4	3117
NM 130860	Cdk9	3389	NM 009389	Tle3	5172	NM 153579	Sv2b	5548

NM 001159696	Tfap2c	2794	NM 001083928	Tle3	5148	NM 001109753	Sv2b	5341
NM 001190705	Dlk1	4186	NM 001291147	Derl2	3703	NM 008679	Ncoa3	7571
NM 001113198	Mitf	4890	NM 001163675	Abcc4	5711	NM 009251	Serpina3g	2030
NM 009328	Tcf15	949	NM 008172	Grin2d	4383	NM 011364	Sh2d1a	819
NM 001252434	Dlg1	4612	NM 008236	Hes2	2521	NM 177668	Skint10	1729
NM 010202	Fgf4	3030	NM 001081979	Mecp2	10152	NM 009012	Rad50	5153
NM 009417	Tpo	3291	NM 001290469	Atp1a3	3609	NM 029564	Tax1bp3	1417
NM 001039522	Leo1	2192	NM 178712	Gpr64	4723	NM 001081142	Kcnq4	2512
NM 007862	Dlg1	4777	NM 001291148	Derl2	3665	NM 008377	Lrig1	4966
NM 011785	Akt3	4735	NM 028734	Steap2	10398	NM 001040026	Sco1	4280
NM 016884	Hnrnpc	2844	NM 001146031	Nrcam	7362	NM 001033238	Cblb	6323
NM 001146295	Celf4	2258	NM 010652	Klrc1	1942	NM 001013023	Mterf1a	1376
NM 010681	Lama4	6046	NM 001290393	Sntg1	7213	NM 007976	F5	7406
NM 001146293	Celf4	3950	NM 001290986	Wdr76	4246	NM 010937	Nras	4470
NM 021457	Fzd1	4395	NM 001079848	Gpr64	4642	NM 026003	Smarca2	1964
NM 001286942	Pitx2	1907	NM 019464	Sh3glb1	5903	NM 029789	Cers2	2048
NM 009005	Rab7	624	NM 007610	Casp2	3463	NM 001111053	Dclk1	6026
NM 001170982	Hnrnpc	2779	NM 026869	Pygo2	2911	NM 019588	Plce1	9525
NM 172688	Map3k7	5682	NM 001282037	Sh3glb1	5966	NM 007526	Barx1	1366
NM 001164792	Tpbg	3509	NM 001276679	Syt6	1599	NM 008858	Prkd1	3778
NM 001252433	Dlg1	4675	NM 001290540	Pdzd4	3646	NM 001081143	Gnat3	1174
NM 010052	Dlk1	4339	NM 001290345	Map4k3	4176	NM 001098230	Pdp1	4233
NM 001024837	Adarb1	6602	NM 001290501	Znrf3	7199	NM 001042655	Tbc1d17	2601
NM 011887	Scn11a	5837	NM 001290558	Slc25a25	3195	NM 026206	Prl7c1	1016
NM 172493	Diap2	8456	NM 001271472	Scube1	7932	NM 011376	Sim1	7355
NM 009411	Tpbpa	1372	NM 001290537	Otud5	3879	NM 001031808	Mrpl41	2423
NM 011101	Prkca	8385	NM 001290455	Gripap1	3021	NM 010595	Kcna1	8970
NM 146191	Lrrk1	7467	NM 001278259	Prn	2162	NM 148948	Dicer1	9851
NM 194263	Tbx20	6436	NM 001271678	Wnk3	10400	NM 031168	Il6	1087
NM 001170981	Hnrnpc	2805	NM 001291203	Tpd52l2	3520	NM 030238	Dync1h1	14398
NM 001166414	Rfx3	9146	NM 001286040	Anks1	7092	NM 011348	Sema3e	6877
NM 007798	Ctsb	4756	NM 001285423	Esd	2121	NM 001025597	Ikzf1	5163
NM 028597	Thoc3	2326	NM 001290714	Bai2	5185	NM 020568	Plin4	5755
NM 001190703	Dlk1	4054	NM 001290716	Suv39h1	3097	NM 027631	Cabs1	1520
NM 177360	Dmrt3	2401	NM 001291189	Cdh26	2965	NM 178655	Ank2	5790
NM 001289643	Scgb3a2	615	NM 001290989	Clstn1	4475	NM 001113181	Gria4	2338
NM 008305	Hspg2	14201	NM 001285997	Prc1	3040	NM 008796	Pctp	1971
NM 001178049	Mitf	4777	NM 001271348	Fbxw11	4237	NM 010120	Eif1a	2879
NM 011098	Pitx2	1946	NM 001285872	Pafah2	3194	NM 016964	Stag3	4246
NM 001042504	Pitx2	1808	NM 001290475	Tdrd7	3658	NM 013630	Pkd1	14170
NM 009510	Ezr	3070	NM 001290390	Sntg1	7264	NM 001111052	Dclk1	7030
NM 011627	Tpbg	3618	NM 001290645	C8a	3532	NM 007888	Dvl2	2937
NM 008970	Pthlh	1512	NM 001291167	Apba2	3283	NM 011594	Timp2	3635
NM 013869	Tnfrsf19	4639	NM 001277286	Ank1	8191	NM 001040396	Selt	3575
NM 001242368	F10	2693	NM 001282994	Cobl	5464	NM 013813	Epb4.1l3	4051
NM 007972	F10	2503	NM 001291154	Sgk2	3068	NM 008170	Grin2a	4512
NM 001170984	Hnrnpc	2781	NM 001289731	Mcf2	4227	NM 008171	Grin2b	7515
NM 001025305	Tfap2b	6167	NM 001271473	Scube1	7599	NM 026157	Mtpap	2651
NM 145624	Zfp709	3990	NM 001290819	Zscan29	5941	NM 025574	Pyurf	5410
NM 145991	Cdc73	2692	NM 028542	Wdr13	4485	NM 010493	Icam1	2540
NM 008481	Lama2	9734	NM 027096	Gpcpd1	3516	NM 001033453	Pdp1	4198
NM 001278447	Rab18	3688	NM 001290545	Aftph	4065	NM 018763	Chst2	6256
NM 008996	Rab1	2657	NM 001285998	Prc1	2972	NM 009706	Arhgap5	5197
NM 009334	Tfap2b	6132	NM 001278258	Prn	3400	NM 001034168	Ank2	5487
NM 194268	Onecut2	13407	NM 175272	Nav2	11332	NM 001080118	Med1	6518
NM 008601	Mitf	4581	NM 001286041	Anks1	7029	NM 177342	Taf5	3258
NM 009335	Tfap2c	2853	NM 007566	Birc6	15787	NM 021050	Cftr	6305
NM 029522	Gpsm2	3510	NM 001290311	Wnk2	8496	NM 011240	Ranbp2	9477
NM 001042617	Cadps	5475	NM 001134743	Lrrtm4	3054	NM 007669	Cdkn1a	1910
NM 008957	Ptch1	4305	NM 001290630	Rbm41	5611	NM 173752	Lgals1	3635
NM 001252435	Dlg1	4370	NM 001289510	Tecpr2	7793	NM 172135	Mterf1a	1357
NM 001287048	Pitx2	2317	NM 001290570	Ralgps1	6077	NM 009578	Ikzf1	4902

NM 001146292	Celf4	4007	NM 001277904	Tnnt1	994	NM 009635	Avil	3033
NM 009922	Cnn1	1987	NM 001290389	Atp1b4	4266	NM 010575	Itga2b	3437
NM 175647	Dmrta1	4073	NM 001290448	Nfx1	4368	NM 007578	Cacna1a	7929
NM 177356	Lamp3	3343	NM 001291186	Abr	4463	NM 007567	Bsn	15953
NM 181070	Rab18	3670	NM 001290818	Adrbk1	3379	NM 177780	Dock5	10335
NM 001164155	Tnfrsf19	4036	NM 001289514	Tbc1d1	4802	NM 001098227	Sdcbp	2553
NM 001252436	Dlg1	4513	NM 001290982	Myo1b	4879	NM 010370	Gzma	878
NM 001122739	Inppl1	4778	NM 001277284	Ank1	8055	NM 019978	Dclk1	7865
NM 019670	Diap3	3516	NM 001276764	Dst	24271	NM 008418	Kcna3	1968
NM 011897	Spry2	2103	NM 001277106	Crlf3	2283	NM 025730	Lrrk2	8231
NM 021530	Slc4a8	11811	NM 001291050	Gpcpd1	3592	NM 009383	Tial1	4292
NM_001081343	3110043021Rik	3198	NM_001276432	Wfdc8	3235	NM_153071	Gprc6a	2856
NM 001146294	Celf4	3980	NM 001291190	Ssh2	9176	NM 013634	Med1	6383
NM 010567	Inppl1	5012	NM 001287139	Zzz3	7546	NM 001098231	Pdp1	4149
NM 134255	Elovl5	2802	NM 030706	Trim2	7207	NM 027498	Sik3	6296
NM 183147	Sprn	3172	NM 001286729	Glcci1	5525	NM 019691	Gria4	5458
NM 001291029	Prdm16	8436	NM 001285853	Matk	1847	NM 010865	Myoc	2093
NM 001286601	Clasp2	5644	NM 001277122	Hnrnpr	7861	NM 001113414	Ebf3	5016
NM 001286602	Clasp2	5690	NM 001110506	Efcab12	2551	NM 001163669	Tnnt3	1069
NM 001271402	Ephx2	2142	NM 001113360	Plch2	5067	NM 010824	Mpo	2570
NM 010153	Erbb3	4020	NM 001271406	Capzb	1641	NM 001081499	Tbc1d8b	5907
NM 001290549	Tek	4699	NM 001277289	Ank1	8269	NM 008372	Il7r	3227
NM 013719	Eif2ak4	5220	NM 001271405	Capzb	1655	NM 177175	Tmem215	3218
NM 001038845	P2rx7	1633	NM 001290536	Otud5	4262	NM 016674	Cldn1	3263
NM 001290551	Tek	4549	NM 134151	Yars	2874	NM 001160039	Ndufs1	2778
NM 001113209	Nfib	9270	NM 001290496	Trub2	3866	NM 001198894	Gpr56	3605
NM 001114347	Clasp2	6369	NM 001271726	Trim2	7144	NM 010096	Ebf3	4908
NM 178804	Slit2	8492	NM 001040434	Rgag1	4142	NM 001085495	Arfgef2	8777
NM 001271403	Ephx2	1987	NM 001276677	Syt6	1665	NM 001013405	Tcaim	3053
NM 010250	Gabra1	4702	NM 001290747	Tbx22	3073	NM 001113415	Ebf3	5043
NM 007691	Chek1	3397	NM 001290713	Stag2	5814	NM 001160139	Kcnq5	6992
NM 001291227	Slit2	8555	NM 001290708	Smarca1	4024	NM 172579	Sipa1l1	7508
NM 001098170	Pcdh10	6064	NM 001276397	Ube2d2b	1616	NM 011111	Serpinb2	2007
NM 001146689	Ezh2	2653	NM 001285885	Zmym6	5036	NM 025383	Necap2	1909
NM 001252260	Npm1	1401	NM 001289572	Spata7	1975	NM 001163670	Tnnt3	1024
NM 011415	Snai2	2084	NM 001290821	Glra1	2413	NM 001114386	Nedd4l	8163
NM 029633	Clasp2	5624	NM 001277273	Fancl	1783	NM 001159595	Ints8	4676
NM 001289575	Tsc1	7732	NM 001281845	Anxa8	1814	NM 080575	Acss1	3594
NM 008974	Ptp4a2	3413	NM 001285831	Far1	4339	NM 001163668	Tnnt3	1057
NM 001113210	Nfib	9243	NM 001276704	Nxf1	4035	NM 145518	Ndufs1	2674
NM 001025192	Cxadr	5566	NM 001271491	Tfe3	2908	NM 001166027	Skint11	1393
NM 001177995	Prdm16	8433	NM 001291213	Myo18a	7504	NM 172729	Nod1	4327
NM 008687	Nfib	8932	NM 001290765	Slc39a13	2416	NM 023630	Gtf2a1l	1619
NM 026842	Ubqln1	3693	NM 001284372	Atf2	4006	NM 018882	Gpr56	3555
NM 007940	Ephx2	2062	NM 001290674	Scn9a	9865	NM 025877	Slc25a23	3376
NM 011027	P2rx7	4936	NM 001286033	Stx2	2812	NM 009163	Sgpl1	4133
NM 030677	Gpx2	1071	NM 001291212	Myo18a	7396	NM 175750	Plxna4	12602
NM 001038619	Dnm3	7534	NM 001285854	Matk	1844	NM 178112	Ints8	3434
NM 001284402	P2rx7	4764	NM 001291158	Zmynd8	7345	NM 011401	Slc2a3	3973
NM 010229	Flt3	3664	NM 001272030	Arvcf	4569	NM 010957	Ogg1	1556
NM 001164745	Ptp4a2	3382	NM 001272076	Trim24	5771	NM 001164193	Mtm1	770
NM 201255	Krt9	2580	NM 001286002	Scyl3	4160	NM 001163665	Tnnt3	1108
NM 010306	Gnai3	3294	NM 001290991	Ctdspl2	6734	NM 001160040	Ndufs1	2832
NM 029726	Trdn	4514	NM 001271456	Tnip1	2817	NM 010442	Hmox1	1634
NM 008722	Npm1	1440	NM 001290745	Nckap1	4421	NM 172555	Papolg	3776
NM 001038839	P2rx7	2568	NM 001290771	Tro	3350	NM 010881	Ncoa1	7346
NM 001286127	Nfib	9154	NM 001271759	Adra1a	4118	NM 001081370	Shank2	5913
NM 011587	Tie1	3883	NM 001276489	Ism1	2965	NM 023872	Kcnq5	6935
NM 019413	Robo1	7568	NM 001286573	Ehmt2	3968	NM 010054	Dlx2	2492
NM 001177806	Eif2ak4	4916	NM 001291237	Tlcd1	1327	NM 010176	Fah	1597
NM 001291228	Slit2	8504	NM 001290626	Mapkap1	3124	NM 011620	Tnnt3	1036

NM	001081177	Kif13b	5603	NM	001271489	Tfe3	3274	NM	001113373	Shank2	6359
NM	017374	Ppp2cb	1836	NM	001024849	Gm14325	4396	NM	011146	Pparg	1769
NM	001289576	Tsc1	7714	NM	001291150	Ptprt	12129	NM	181322	Ctcf	3848
NM	007971	Ezh2	2665	NM	001271679	Wnk3	10259	NM	026582	Wls	2696
NM	010010	Cyp46a1	2146	NM	001277121	Hnrnpr	8022	NM	172566	Rundc1	3230
NM	011104	Prkce	6254	NM	001291052	Gpcpd1	3640	NM	033601	Bcl3	1835
NM	001291026	Prdm16	8602	NM	001290829	Cpeb3	5758	NM	001001983	Pi4ka	6449
NM	022887	Tsc1	7700	NM	001285500	Tead2	2010	NM	001110140	Atp2a2	4565
NM	001098404	Nr1i2	2417	NM	001289589	Zfp12	5276	NM	001174170	Serpinb2	1988
NM	027504	Prdm16	8605	NM	001290659	Tanc1	7763	NM	080853	Slc17a6	4338
NM	001286131	Nfib	9021	NM	001285800	Polr1e	3691	NM	001122889	Epha7	3272
NM	178591	Nrg1	2103	NM	001290313	Wnk2	7202	NM	001163664	Tnnt3	1108
NM	001110205	Acvr1	2790	NM	001276455	Slc19a2	3457	NM	001160038	Ndufs1	2936
NM	001122829	Upf1	4631	NM	001284189	Homer1	4768	NM	001114334	Rps6kb1	5395
NM	010936	Nr1i2	2540	NM	001290446	Gpr64	4714	NM	001127330	Pparg	1857
NM	019935	Ovol1	2900	NM	001290810	Mrto4	1242	NM	001163666	Tnnt3	1081
NM	001110204	Acvr1	3312	NM	001167164	Gm4214	945	NM	031881	Nedd4l	8212
NM	030680	Upf1	4598	NM	001277079	Gas7	6934	NM	010887	Ndufs4	1534
NM	013690	Tek	4702	NM	001290690	Kcnip1	2005	NM	001167983	Sipa1l1	7717
NM	001271421	Ephx2	2037	NM	001276467	Asap1	6110	NM	001163667	Tnnt3	1096
NM	007394	Acvr1	3067	NM	001290428	Frmpd4	8156	NM	007538	Opn1sw	2420
NM	023842	Dsp	9592	NM	001290805	Kif3a	5441	NM	173447	Ephb1	4686
NM	023478	Upk3a	1106	NM	001272064	Trim24	6089	NM	028882	Sema3d	6521
NM	001123362	Prdm12	2471	NM	001290500	Ttc14	7536	NM	178633	Klhl2	3319
NM	001081960	Clasp2	5627	NM	001276681	Syt6	4416	NM	001291804	Dsc1	4961
NM	019482	Panx1	2112	NM	001286630	Brd4	5995	NM	013504	Dsc1	5007
NM	024264	Cyp27a1	1890	NM	001290644	Rp2h	4581	NM	001165984	Ubap2l	4033
NM	008070	Gabrb2	7568	NM	001271450	Enox2	3933	NM	025436	Msmo1	1824
NM	001286600	Clasp2	5678	NM	001285459	Fmn1	11147	NM	001165986	Ubap2l	3720
NM	009370	Tgfbr1	5735	NM	001290665	Csrnp3	10644	NM	138666	Nlgn1	5630
NM	152234	Ubqln1	3609	NM	133946	Nlrp6	4438	NM	001165988	Ubap2l	3627
NM	011102	Prkcg	3131	NM	001271347	Fbxw11	4276	NM	001165983	Ubap2l	4073
NM	013755	Gyg	1736	NM	001291145	Emilin3	3447	NM	001165987	Ubap2l	3652
NM	145967	Vstm2a	3629	NM	001291049	Usp43	4447	NM	028475	Ubap2l	4070
NM	009946	Cplx2	4928	NM	001286148	Lepre1	3231	NM	183088	Zbed5	2492
NM	001291434	Prkcg	2978	NM	001105197	Tfe3	2979	NM	153489	Ubap2l	3956
NM	001291440	Rad51c	3122	NM	001282102	Lrrtm4	3629	NM	001163387	Nlgn1	4603
NM	001291444	Pacs2	5486	NM	001290385	Arhgef9	5532	NM	134063	Fam208b	8356
NM	001291452	Lrrc7	7321	NM	001277941	Rad51d	6937	NM	009962	Ptgdr2	2644
NM	001081358	Lrrc7	7462	NM	001290728	Sytl5	6557	NM	001165985	Ubap2l	3745
NM	177006	Nwd2	8539	NM	001193266	Mdga2	9834	NM	020259	Hhip	9094
NM	001291453	Lrrc7	7184	NM	001271727	Trim2	7413	NM	013823	Kl	5124
NM	001291445	Pacs2	5390	NM	001282093	Wac	5058	NM	001109749	Cntn4	5262
NM	001289652	Gid8	4412	NM	001290737	Thg1l	3740	NM	009912	Ccr1	2831
NM	001081170	Pacs2	5480	NM	001286610	Arhgap25	3495	NM	001048139	Bdnf	4161
NM	029607	Gid8	4932	NM	001290430	Ftsj1	3419	NM	001166667	Rtel1	4316
NM	001284221	Agtpbp1	4364	NM	001290828	Cpeb3	5901	NM	010890	Nedd4	5494
NM	001038593	Glrx2	3506	NM	001290785	Sec24a	6795	NM	008229	Hdac2	2004
NM	001038594	Glrx2	3445	NM	001289821	Lrrc43	2025	NM	009050	Ret	6064
NM	022721	Fzd5	6930	NM	001271545	Slc4a9	3092	NM	001166668	Rtel1	4208
NM	001081249	Vcan	12432	NM	001277123	Hnrnpr	7856	NM	013454	Abca1	10260
NM	009072	Rock2	8012	NM	001290814	Aff3	7251	NM	008809	Pdgfrb	5410
NM	175539	Dcaf12l2	2881	NM	001085410	Nadk2	3740	NM	012046	Spo11	1716
NM	010151	Nr2f1	2432	NM	001271490	Tfe3	3446	NM	016701	Nes	6143
NM	001038592	Glrx2	3603	NM	001277867	Mphosph9	7850	NM	207239	Gtf3c1	6891
NM	207680	Bcl2l11	5040	NM	001272029	Arvcf	4720	NM	008884	Pml	5240
NM	009697	Nr2f2	4216	NM	001289446	Atp9a	3469	NM	080470	Smc1b	4056
NM	001024385	Crls1	1939	NM	001277903	Tnnt1	1030	NM	011169	Prlr	10328
NM	183261	Nr2f2	4164	NM	001174047	Cacna2d2	5539	NM	001048141	Bdnf	4001
NM	025777	Duoxa2	1592	NM	001111016	Nav2	11070	NM	001146268	Pdgfrb	5413
NM	008045	Fshb	1622	NM	001271451	Enox2	3918	NM	001252458	Prkd2	3340
NM	001042659	Fzd5	6823	NM	001289448	Rad1	4136	NM	009194	Slc12a2	6520

NM 026792	Agpat5	3829	NM 001291211	Pcmtd2	3572	NM 207668	Acpp	4488
NM 010719	Lipe	3221	NM 001291010	Tnfrsf25	1661	NM 001252330	Slc6a15	3563
NM 008343	Igfbp3	2456	NM 001291200	Tpd52l2	3580	NM 010612	Kdr	5464
NM 010776	Mbl2	1069	NM 001276423	Cass4	3627	NM 021420	Stk4	5189
NM 007758	Cr2	6207	NM 001291039	Cds2	8089	NM 007499	Atm	11964
NM 178793	Ccbe1	5742	NM 010081	Dst	8788	NM 008706	Nqo1	1552
NM 001081107	Helq	3688	NM 001277281	Ank1	7980	NM 007666	Cdh6	2632
NM 027770	Col24a1	7137	NM 001284314	Ube2j2	3139	NM 178087	Pml	5378
NM 010684	Lamp1	2265	NM 001174048	Cacna2d2	5521	NM 001001882	Rtel1	4451
NM 001083894	Liph	3811	NM 001290376	Camk1d	6742	NM 013556	Hprt	1349
NM 028995	Nipal3	5025	NM 001286575	Ehmt2	3907	NM 011828	Hs2st1	4811
NM 001082960	Itgam	4682	NM 001290801	Mgat4a	7172	NM 007884	Epyc	1723
NM 145399	Scgn	1425	NM 001291137	Ralgapb	8426	NM 173004	Cntn4	2931
NM 178681	Dgkb	5544	NM 001272055	Numb	3349	NM 013806	Abcc2	5389
NM 008401	Itgam	4679	NM 001291051	Gpcpd1	3622	NM 007540	Bdnf	4302
NM 198029	Fermt1	5255	NM 001291242	Cct6b	1886	NM 007668	Cdk5	2058
NM 175502	Tmem74	1472	NM 001291202	Tpd52l2	3562	NM 001083960	Spo11	1602
NM 146200	Eif3c	2896	NM 001290449	Nfx1	4899	NM 175328	Slc6a15	3660
NM 030678	Gys1	3681	NM 001277280	Ank1	7794	NM 009917	Ccr5	2926
NM 207681	Bcl2l11	4872	NM 001271843	Aqp9	2646	NM 008960	Pten	8229
NM 020021	Mos	1449	NM 001271883	Vmn2r124	2568	NM 001048142	Bdnf	4009
NM 001079869	Hoxb3	3390	NM 001290995	Kif1b	10288	NM 011515	Vamp7	2534
NM 009754	Bcl2l11	4782	NM 001290741	Cnot6	5372	NM 001109751	Cntn4	3019
NM 001081131	Dhtkd1	3535	NM 001271725	Trim2	7108	NM 007734	Col4a3	8609
NM 001081212	Irs2	4015	NM 001289472	Pitpnm2	6941	NM 001166666	Rtel1	4334
NM 001081290	Prrc2c	10444	NM 001276461	Asap1	6101	NM 175260	Myh10	7783
NM 175513	Zfp804a	4196	NM 001282993	Cobl	5558	NM 001083959	Spo11	1641
NM 008066	Gabra2	2392	NM 001291056	Snap25	2135	NM 010511	Ifngr1	2099
NM 172621	Clic5	5870	NM 001272032	Arvcf	4312	NM 001166665	Rtel1	4433
NM 010458	Hoxb3	3313	NM 001289844	Palb2	2661	NM 011749	Zfp148	9431
NM 023505	Glrx2	3482	NM 001276355	Grin3a	7727	NM 001080780	Ret	7341
NM 025626	Fam107b	3148	NM 001285812	Tmem53	1020	NM 009915	Ccr2	3589
NM 013526	Gdf6	3532	NM 001290730	Hoxd8	2052	NM 178900	Prkd2	3625
NM 001113383	Gls	4419	NM 001290684	Shroom2	7501	NM 139001	Cspg4	8050
NM 001001602	Dab2ip	6424	NM 001040395	Nadk2	3674	NM 001100185	Cyp4a30b	1527
NM 007482	Arg1	1489	NM 001289445	Atp9a	3739	NM 001271431	Cd82	1625
NM 133753	Errfi1	3034	NM 001290704	Slc25a14	1857	NM 133828	Creb1	8389
NM 001127260	Trp63	5288	NM 001291166	Apba2	3337	NM 001271462	Cd82	1724
NM 008216	Has2	4262	NM 001290308	Col12a1	11719	NM 001080926	Lrp8	7296
NM 008591	Met	6652	NM 001285855	Matk	1912	NM 013842	Xbp1	2264
NM 009431	Ctr9	4317	NM 001284354	Shprh	6832	NM 181414	Pik3c3	3102
NM 001159544	Frk	4825	NM 153319	Amot	6974	NM 001276485	Dag1	5378
NM 001164635	Slc22a8	3323	NM 001290627	Atp4a	3497	NM 001253375	Kcnma1	6020
NM 013881	Ulk2	5813	NM 001286824	Uspl1	3730	NM 001163530	Prl	892
NM 001127264	Trp63	4707	NM 001290631	Gapdhs	1458	NM 001099298	Scn2a1	8690
NM 011076	Abcb1a	4977	NM 001285458	Fmn1	11523	NM 146261	Fam199x	8308
NM 001127263	Trp63	1729	NM 001276502	Vps13d	15762	NM 009888	Cfh	4365
NM 001127259	Trp63	5382	NM 001291153	Xaf1	2313	NM 010931	Uhrf1	3583
NM 010237	Frk	4669	NM 001025359	Klc1	2457	NM 001276474	Cited1	1020
NM 016697	Gpc3	2272	NM 001281871	Pbdc1	1244	NM 001276492	Dag1	5582
NM 028039	Esco2	2899	NM 029589	Tmem53	921	NM 145963	Kcnj14	2639
NM 001013779	Aim2	2541	NM 001276359	Camsap1	7996	NM 001253368	Kcnma1	6119
NM 027286	Ace2	3418	NM 001282942	Fancc	2937	NM 133365	Dnah5	15616
NM 173053	Limk2	4668	NM 001290384	Arhgef9	4708	NM 008139	Gnaq	5634
NM 178750	Ss18l1	4395	NM 001271343	Ate1	5020	NM 011045	Pcna	1260
NM 133957	Nfat5	13451	NM 001167165	Gm4175	945	NM 028673	Zdbf2	12621
NM 027439	Atp6ap2	2376	NM 001290624	Prickle3	2410	NM 023566	Muc2	7368
NM 001127265	Trp63	1717	NM 001272056	Numb	3432	NM 009152	Sema3a	6850
NM 027126	Hfe2	2021	NM 001290467	Hs6st2	4672	NM 001164192	Mtm1	3286
NM 001145886	Tiam1	7277	NM 001290743	Ppp1r1c	3804	NM 001195539	Dclk1	7004
NM 007770	Crx	2931	NM 001291134	Rprd1b	4520	NM 019739	Foxo1	5552
NM 026217	Atg12	2492	NM 001290816	Ablim1	6301	NM 001036684	Atp2b2	6922

NM 028288	Cul4b	5020	NM 001291135	Rprd1b	4616	NM 028053	Tmem38b	2861
NM 009621	Adamts1	4904	NM 001291149	Ptprt	12159	NM 001159769	Nr5a2	3635
NM 001145887	Tiam1	4210	NM 001291146	Derl2	3959	NM 001001490	Oxgr1	3732
NM 010791	Meox1	2235	NM 001286728	Glcci1	5528	NM 001253366	Kcnma1	4979
NM 009672	Anp32a	2105	NM 001290669	Ttc21b	4402	NM 001253860	Scn5a	8287
NM 001113330	Crx	2903	NM 175556	Plch2	5598	NM 011198	Ptgs2	4150
NM 018775	Tbc1d8	4451	NM 172997	Idua	4289	NM 029658	Fam101b	3533
NM 007952	Pdia3	2658	NM 001277942	Rad51d	6910	NM 016975	Gja3	2696
NM 001005509	Eif2a	2322	NM 001290703	Slc25a14	1866	NM 029455	Fam227b	1858
NM 008356	Il13ra2	1558	NM 001284373	Atf2	3943	NM 010325	Got2	2345
NM 018823	Nfat5	13389	NM 001290815	Ablim1	6259	NM 001078167	Srsf1	5560
NM 001110780	Syn1	3211	NM 001281466	Mroh2a	7502	NM 016694	Park2	3202
NM 019389	Vcan	9552	NM 001276444	Gpr155	4885	NM 010908	Nfkbib	1945
NM 011264	Rev3l	10666	NM 146103	Tmem185b	2830	NM 001167886	Suv420h1	6002
NM 008626	Mrc2	5801	NM 001290519	Sun3	1178	NM 178029	Setd1a	5934
NM 013680	Syn1	3249	NM 001271728	Trim2	7211	NM 021544	Scn5a	8455
NM 007872	Dnmt3a	9735	NM 001290696	Foxj3	4706	NM 144859	Pja2	4520
NM 018729	Cd244	3758	NM 001291138	Ralgapb	8390	NM 009969	Csf2	1033
NM 001136104	Abl2	10402	NM 001284427	Smtn	3181	NM 001276481	Dag1	5534
NM 011612	Tnfrsf9	2134	NM 001285814	Tmem53	1085	NM 026742	Ndufaf4	3632
NM 183032	Rnase9	1187	NM 001281926	Zmynd8	5079	NM 009674	Anxa7	2965
NM 010123	Eif3a	5176	NM 001195413	Cngb1	6201	NM 007709	Cited1	1107
NM 001127177	Ptpn2	8423	NM 001290315	Igdcc4	6230	NM 001037726	Creb1	8267
NM 010208	Fgr	3353	NM 001290291	Shisa7	5956	NM 010568	Insr	9357
NM 011770	Ikzf2	9457	NM 001290484	Gtf3c5	2823	NM 001128170	Cyld	8023
NM 001077509	Tnfrsf9	2115	NM 174991	Bai1	6227	NM 153075	Catsper2	2247
NM 020008	Clec7a	2277	NM 001104648	Vmn2r56	2340	NM 001199136	Macf1	23495
NM 001114125	Dab2ip	5388	NM 008947	Psmc1	1502	NM 001253371	Kcnma1	5031
NM 001127262	Trp63	4625	NM 026236	Wdr48	3821	NM 027533	Tspan2	4261
NM 009951	Igf2bp1	8382	NM 181545	Slfn8	4011	NM 201242	Lims1	4351
NM 010029	Ddx4	2772	NM 011739	Ywhaq	2110	NM 009723	Atp2b2	7111
NM 011641	Trp63	4710	NM 145531	Spg11	7665	NM 001253365	Kcnma1	4982
NM_027426	Draxin	5217	NM_053264	4930444G20Rik	1771	NM_022984	Retn	1139
NM 001025602	Il1rl1	5080	NM 001243138	Gm13247	3068	NM 008716	Notch3	7943
NM 013743	Pdk4	3453	NM 001243139	Gm13247	2953	NM 001081445	Ncam1	6192
NM 001127261	Trp63	2380	NM 001177767	Rex2	3998	NM 008607	Mmp13	2675
NM 019712	Rbx1	1655	NM 012012	Exo1	5506	NM 021297	Tlr4	3847
NM 009757	Bmp15	3086	NM 010279	Gfra1	4664	NM 001252060	Cacna1a	7778
NM 009982	Ctsc	2472	NM 001276409	Fn1	8077	NM 145581	Siglec5	2529
NM_001077508	Tnfrsf9	1999	NM_013723	Podxl	5330	NM_001131021	D630003M21Rik	4523
NM 009595	Abl2	10714	NM 130895	Adarb1	6572	NM 001253358	Kcnma1	5063
NM 023662	Pcm1	8398	NM 008055	Fzd4	3685	NM 172689	Ddx58	4943
NM 011516	Sycp1	3437	NM 012061	Cadps	5478	NM 145556	Tardbp	7454
NM 001130513	Ace2	3566	NM 001042502	Pitx2	2317	NM 001253376	Kcnma1	6020
NM 001122756	Corin	4733	NM 009375	Tg	8462	NM 001253362	Kcnma1	5130
NM 016869	Corin	4890	NM 001177883	Elavl2	4182	NM 001081414	Grm5	8428
NM 009826	Rb1cc1	7046	NM 001136067	Ikbkg	6918	NM 007429	Agtr2	2872
NM 008110	Gdf9	1805	NM 011607	Tnc	7100	NM 008026	Fli1	3087
NM 029770	Unc5b	5867	NM 133237	Apcdd1	2799	NM 001025432	Crebbp	7507
NM 010113	Egf	4757	NM 031191	Prl2c2	790	NM 019948	Clec4e	2519
NM 172955	Vcan	3745	NM 001276412	Fn1	7792	NM 001111316	Ptprc	5568
NM 022023	Gmfb	4171	NM 011265	Rfx3	9187	NM 001113204	Ncam1	6993
NM 178753	Spin4	4176	NM 009316	Map3k7	5763	NM 007399	Adam10	4605
NM 013569	Kcnh2	4221	NM 001008425	Thoc6	1461	NM 020256	Zbtb33	5122
NM 001145885	Ddx4	2850	NM 008482	Lamb1	5778	NM 010408	Hcn1	7911
NM 009758	Bmpr1a	5481	NM 130860	Cdk9	3389	NM 001199137	Macf1	17414
NM 009794	Capn2	3205	NM 011324	Scnn1a	3515	NM 001164191	Mtm1	3379
NM 009867	Cdh4	6393	NM 018753	Ywhab	2775	NM 001253363	Kcnma1	5127
NM 001110142	Cul4b	5079	NM 177798	Frs2	5701	NM 001276466	Cited1	904
NM 028125	Zbtb46	2577	NM 007858	Diap1	4378	NM 019926	Mtm1	3368

NM 153587	Rps6ka5	4406	NM 001159696	Tfap2c	2794	NM 001243072	Sema3a	6582
NM 001286718	Tsc2	6134	NM 001276413	Fn1	7522	NM 001252488	Catsper3	1199
NM 009622	Adcy1	12259	NM 018741	Igfbpl1	2729	NM 001164201	Cers3	3251
NM 023735	Actr3	2746	NM 013855	Abca3	6515	NM 028390	Anln	5421
NM 173394	Ticam2	3264	NM 013607	Myh11	6632	NM 001164190	Mtm1	3488
NM 001281976	Ctla4	1823	NM 001039581	Abca3	6442	NM 001146200	Pik3cg	6634
NM 001252470	Cpt1c	2809	NM 172395	Cdc42se1	3128	NM 001252498	Rapgef6	4843
NM 009384	Tiam1	7312	NM 029933	Bcl9	6102	NM 144871	Suv420h1	6062
NM 033325	Loxl2	5186	NM 009417	Tpo	3291	NM 001111080	Uhrf1	3415
NM 011938	Grk6	2100	NM 010100	Edar	3697	NM 001128171	Cyld	8148
NM 029466	Arl5b	3594	NM 001276411	Fn1	7807	NM 175307	Fam46b	2288
NM 001080971	Tubb1	2004	NM 008106	Opn1mw	1221	NM 030676	Nr5a2	3720
NM 001253710	Mbnl1	4436	NM 010580	Itgb5	3275	NM 001171007	Nod1	4076
NM 001172136	Exog	3905	NM 011785	Akt3	4735	NM 001253370	Kcnma1	6107
NM 013456	Actn3	2887	NM 001276408	Fn1	8152	NM 001253360	Kcnma1	5051
NM 001289582	Liph	3805	NM 001161421	Ikbkg	6915	NM 007426	Angpt2	3560
NM 019786	Tbk1	3031	NM 010681	Lama4	6046	NM 001003918	Usp7	5427
NM 153410	Gpsm1	3389	NM 021457	Fzd1	4395	NM 010431	Hif1a	4775
NM 001286066	Grk6	2093	NM 001038610	Dach1	5310	NM 001168296	Ephb1	4563
NM 001285794	Wnt11	2780	NM 001110825	Foxp4	4020	NM 001161791	Mefv	3094
NM 001199147	Gpsm1	3458	NM 001286942	Pitx2	1907	NM 008914	Ppp3cb	3881
NM 011647	Tsc2	6266	NM 172688	Map3k7	5682	NM 177821	Ep300	8749
NM 001034030	Limk2	3898	NM 001039666	Krt40	1320	NM 001253377	Kcnma1	4944
NM 008831	Phb	1815	NM 134438	Gpr37l1	2267	NM 001163028	Bcmo1	2532
NM 001172095	Kdm4c	4207	NM 008103	Gcm1	2034	NM 013729	Mixl1	2294
NM 007389	Chrna1	4320	NM 008483	Lamb2	5624	NM 001166009	Tmem215	2276
NM 001205386	Actr3	2551	NM 207685	Elavl2	3889	NM 001276400	Rad21l	1659
NM 001253708	Mbnl1	5485	NM 008079	Galc	3723	NM 009068	Ripk1	4512
NM 172785	Zc3h12d	4093	NM 001024837	Adarb1	6602	NM 011857	Tenm3	10978
NM 001039363	Tsc2	6137	NM 007826	Dach1	5466	NM 001253369	Kcnma1	5037
NM 009519	Wnt11	2788	NM 011887	Scn11a	5837	NM 001271432	Cd82	1920
NM_001163728	4930563E22Rik	3313	NM_010486	Elavl2	3780	NM_010017	Dag1	5591
NM 009843	Ctla4	1933	NM 010172	F7	1868	NM 001271623	Gja3	2668
NM 001199146	Gpsm1	3209	NM 001024918	Rfx4	3940	NM 173788	Npr2	3660
NM 001285792	Wnt11	3022	NM 172493	Diap2	8456	NM 010119	Ehd1	3182
NM 010000	Cyp2b9	1877	NM 001145920	Runx2	6475	NM 008727	Npr1	4065
NM 153403	Ago1	7065	NM 009411	Tpbpa	1372	NM 001243073	Sema3a	6422
NM 176860	Ubash3b	3243	NM 001161424	Ikbkg	6785	NM 145979	Chd4	6438
NM 001253711	Mbnl1	4454	NM 001025067	Lrig2	7134	NM 011631	Hsp90b1	2759
NM 009640	Angpt1	4316	NM 011101	Prkca	8385	NM 021486	Bcmo1	2327
NM 009659	Alox12b	2347	NM 001038708	Cdc42se1	2758	NM 126166	Tlr3	4327
NM 001164634	Slc22a8	3415	NM 001161423	Ikbkg	6788	NM 010157	Esr2	3308
NM 153178	Ago2	8031	NM 178590	Ikbkg	6811	NM 173369	Cyld	8014
NM 008462	Klra2	1787	NM 146191	Lrrk1	7467	NM 016984	Trpc4	3490
NM 144787	Kdm4c	4271	NM 001145884	Itgb5	3304	NM 001252059	Cacna1a	7788
NM 001205385	Actr3	2554	NM 194263	Tbx20	6436	NM 001079513	Zbtb33	4948
NM 021877	Hr	5501	NM 009755	Bmp1	3769	NM 011526	Tagln	1586
NM 025992	Herc6	5279	NM 007392	Acta2	2572	NM 010586	Itpr2	11740
NM 001289581	Liph	3870	NM 001080968	Golga2	4396	NM 178717	Rxfp3	4252
NM 001286063	Grk6	2996	NM 001166414	Rfx3	9146	NM 001195540	Dclk1	5175
NM 031194	Slc22a8	3495	NM 198037	Cachd1	4981	NM 001271019	Siglec5	2427
NM 001286720	Tsc2	6353	NM 001285457	Gfra1	4425	NM 001033228	Itgal	5984
NM 001253713	Mbnl1	4534	NM 010547	Ikbkg	6814	NM 008371	Il7	2475
NM 001286062	Angpt1	4313	NM 001276410	Fn1	7882	NM 133249	Ppargc1b	3656
NM 001286714	Tsc2	6261	NM 028597	Thoc3	2326	NM 010983	Olfr2	1250
NM 007830	Dbi	587	NM 010186	Fcgr1	2589	NM 009506	Vegfc	1881
NM 001286260	Nfat5	4725	NM 001197041	Ccdc7	1306	NM 007552	Bmi1	3594
NM 133698	Hrnr	10658	NM 001161775	Myh11	6593	NM 029370	Spata25	783
NM 001112711	Grk6	3014	NM 029926	Irak4	2825	NM 009320	Slc6a6	6169
NM 001289813	Dgcr6	1364	NM 008305	Hspg2	14201	NM 020272	Pik3cg	6811
NM 001001979	Megf10	7514	NM 011098	Pitx2	1946	NM 001253372	Kcnma1	5031

NM 010718	Limk2	4537	NM 010402	Hand2	2235	NM 177781	Trpa1	4263
NM 001170851	Klra2	1886	NM 001282992	Pcnt	9476	NM 001276486	Dag1	5435
NM 153679	Cpt1c	2811	NM 009820	Runx2	5707	NM 001271628	Gja5	3180
NM 001253709	Mbnl1	4426	NM 001042504	Pitx2	1808	NM 001253781	Prlr	3916
NM 001286065	Grk6	2912	NM 207686	Elavl2	3741	NM 001276493	Dag1	5539
NM 153404	Liph	3721	NM 009510	Ezr	3070	NM 008121	Gja5	3180
NM 001286716	Tsc2	6132	NM 001110824	Foxp4	4056	NM 001252061	Cacna1a	7921
NM 183138	Tet3	10907	NM 133852	Golga2	4477	NM 007981	Acsl1	3891
NM 001286713	Tsc2	6335	NM 145624	Zfp709	3990	NM 007656	Cd82	1833
NM 001039556	Rad54b	2925	NM 008481	Lama2	9734	NM 001193303	Lims1	4473
NM 152807	Ccdc137	2760	NM 011539	Tbxas1	1992	NM 001025309	Pja2	4706
NM 020007	Mbnl1	5580	NM 001081171	Lama5	11404	NM 080465	Kcnn2	2046
NM 001038018	Grk6	3016	NM 028767	Foxp4	4017	NM 009504	Vdr	4354
NM 007818	Cyp3a11	2053	NM 009335	Tfap2c	2853	NM 001276494	Dag1	5430
NM 010047	Dgcr6	1552	NM 001081332	Slc9a5	5222	NM 010513	Igf1r	11978
NM 011638	Tfrc	4920	NM 001042617	Cadps	5475	NM 001167887	Suv420h1	6071
NM 001285795	Wnt11	2624	NM 009745	Bcl7b	1678	NM 001252481	Smad2	8863
NM 172473	Hace1	3785	NM 001287048	Pitx2	2317	NM 001271730	Xbp1	2238
NM 153743	Dnmt3a	8913	NM 011977	Slc27a1	2795	NM 001271430	Cd82	1757
NM 177330	Ghsr	4433	NM 010233	Fn1	8425	NM 019923	Itpr2	11839
NM 001271753	Dnmt3a	9689	NM 178777	Nhlh2	3353	NM 172856	Cers6	3930
NM 011326	Scnn1g	2965	NM 001161422	Ikbkg	6736	NM 001177567	Otogl	6984
NM 001289814	Dgcr6	1355	NM 134126	Ift140	5814	NM 024469	Bhlhe41	5995
NM 016696	Gpc1	3552	NM 008787	Pcnt	9530	NM 001267872	Zdbf2	12571
NM 021411	Rab37	2193	NM 001146038	Runx2	5740	NM 001276473	Cited1	773
NM 001013028	Tmem263	3778	NM 001122739	Inppl1	4778	NM 011673	Ugcg	3719
NM 198861	Lrrc75a	2047	NM 001271627	Runx2	5904	NM 001146201	Pik3cg	6690
NM 001163753	Rab37	2581	NM 021530	Slc4a8	11811	NM 001252487	Catsper3	1352
NM 177716	Ccdc184	2345	NM 007624	Cbx3	1851	NM 009480	Usf1	1822
NM 030554	Rab27b	6977	NM 010567	Inppl1	5012	NM 009428	Trpc5	4158
NM 173864	C1s2	2662	NM 001286601	Clasp2	5644	NM 177909	Slc9a9	3492
NM 001013792	Otulin	1491	NM 001286602	Clasp2	5690	NM 008575	Mdm4	3490
NM 001289651	Gid8	4446	NM 001271402	Ephx2	2142	NM 026148	Lims1	4327
NM 001082553	Rab27b	6876	NM 007982	Ptk2	4414	NM 011210	Ptprc	5249
NM 009547	Zbtb14	3602	NM 001290549	Tek	4699	NM 001145937	Tenm3	10958
NM 011725	Xlr	1056	NM 013719	Eif2ak4	5220	NM 001081111	Tmf1	6781
NM 001291747	Xlr	1008	NM 001290551	Tek	4549	NM 001143834	Grm5	8524
NM_001166497	3110052M02Rik	4367	NM_001114347	Clasp2	6369	NM_001167905	Cyp2c44	1596
NM 007720	Ccr8	1156	NM 178804	Slit2	8492	NM 010610	Kcnma1	5118
NM 001033960	Rabgap1	4238	NM 001271403	Ephx2	1987	NM 173374	Srsf1	5364
NM 026826	Mrps18c	526	NM 172454	Panx3	2518	NM 001253361	Kcnma1	5139
NM 007979	F9	2733	NM 010250	Gabra1	4702	NM 001253364	Kcnma1	6194
NM 009424	Traf6	5536	NM 001291227	Slit2	8555	NM 026212	Agpat2	1574
NM 144860	Mib1	3793	NM 001276263	Cxadr	1386	NM 001136055	Cd82	1701
NM 027184	Ipmk	5432	NM 001098170	Pcdh10	6064	NM 001111078	Uhrf1	3439
NM 009444	Tgoln2	2265	NM 029633	Clasp2	5624	NM 027059	Smco2	1268
NM 008866	Lypla1	2447	NM 011145	Ppard	3240	NM 133821	Phlpp1	6123
NM 008756	Ocln	3192	NM 001130409	Ptk2	3598	NM 001253378	Kcnma1	4941
NM 026490	Mrpl19	5016	NM 001286599	Clasp2	2279	NM 009952	Creb1	8431
NM 001025577	Maf	3642	NM 007864	Dlg4	3339	NM 001111079	Uhrf1	3559
NM 028304	Pus10	3590	NM 007940	Ephx2	2062	NM 145475	Cerk	4562
NM 001009948	Nrsn2	1437	NM 001289791	Asic1	4288	NM 001276482	Dag1	5447
NM 199011	Dgkq	4612	NM 001163263	Rnf20	4312	NM 010754	Smad2	8752
NM 018826	Irx5	2421	NM 011027	P2rx7	4936	NM 001110794	Anxa7	2850
NM 009500	Vav2	3498	NM 027742	Lrrfip2	3215	NM 001001446	Cyp2c44	1964
NM 183426	Sbno2	4679	NM 010585	Itpr1	9877	NM 001253373	Kcnma1	4962
NM 201368	Xkr8	4238	NM 001038619	Dnm3	7534	NM 011164	Prl	895
NM 134020	Tmed4	1645	NM 011578	Tgfbr3	6074	NM 001253359	Kcnma1	5060
NM 010689	Lat	1260	NM 001284402	P2rx7	4764	NM 009373	Tgm2	3549
NM 011942	Lypla2	1593	NM 010229	Flt3	3664	NM 001271461	Cd82	1712
NM 133658	Ercc3	2673	NM 025910	Mina	2159	NM 025481	Smurf2	5346

NM 028956	Pus10	3187	NM 010306	Gnai3	3294	NM 011349	Sema3f	3393
NM 026370	Kat8	1503	NM 029726	Trdn	4514	NM 011896	Spry1	2490
NM 010687	Large	3669	NM 001277875	Tpm2	2108	NM 001167885	Suv420h1	6131
NM 001033654	Pus10	3177	NM 001164838	Lrrfip2	3260	NM 001276279	Cyld	7599
NM 001031667	Gsk3a	2276	NM 001038839	P2rx7	2568	NM 001195538	Dclk1	7817
NM 198608	Aars2	3368	NM 019413	Robo1	7568	NM 177730	Impad1	4636
NM 177905	Piwil4	2637	NM 001177806	Eif2ak4	4916	NM 001253367	Kcnma1	4970
NM 030743	Rnf114	3001	NM 029062	Pih1d3	1449	NM 019453	Mefv	3126
NM 133684	2-Mar	1883	NM 023868	Ryr2	16813	NM 025802	Pnpla2	2467
NM 008109	Gdf5	2317	NM 029457	Senp2	4499	NM 001163689	Pnpla2	2635
NM 177572	Rimkla	4093	NM 172646	Dnm3	4590	NM 138667	Tab2	4278
NM 001081240	Prmt10	2781	NM 001081147	Oxtr	4568	NM 009120	Sar1a	2602
NM 207215	Mycbp2	15269	NM 001109752	Dlg4	3330	NM 001243132	Tspan2	4066
NM 001195748	Rgs22	4008	NM 001291228	Slit2	8504	NM 011427	Snai1	1613
NM 013557	Eif2ak1	2805	NM 001081177	Kif13b	5603	NM 133360	Acaca	9054
NM 145423	Slc5a8	5346	NM 001289704	Cflar	6943	NM 001268286	Ptprc	5177
NM 016963	Tmod3	3590	NM 010010	Cyp46a1	2146	NM 207707	Esr2	3362
NM 009632	Parp2	1834	NM 182999	Rnf20	4159	NM 021406	Trem1	3025
NM 001039521	Rrn3	3607	NM 207225	Hdac4	3960	NM 024200	Mfn1	4531
NM 177595	Mkx	3201	NM 011819	Gdf15	1084	NM 029772	Catsper3	1313
NM 001033177	Krt76	2312	NM 009416	Tpm2	3250	NM 001271768	Bhlhe41	6124
NM 009868	Cdh5	4004	NM 022312	Tnr	5395	NM 021281	Ctss	1357
NM 008072	Gabrd	1896	NM 001098171	Pcdh10	8263	NM 177137	Gnal	5654
NM 007993	Fbn1	9900	NM 011936	Fto	3586	NM 031202	Tyrp1	2755
NM 008652	Mybl2	3702	NM 001033141	Ecscr	1111	NM 001287180	Atf4	1463
NM 001111051	Dclk1	6956	NM 080553	Itpr3	9012	NM 001286684	Slc8a1	15847
NM 008605	Mmp12	3607	NM 010894	Neurod1	2494	NM 008860	Prkcz	4311
NM 007824	Cyp7a1	4172	NM 001122829	Upf1	4631	NM 001285418	Bdnf	3866
NM 008829	Pgr	6889	NM 207653	Cflar	7232	NM 001289744	Lrrc4c	3440
NM 001077684	Ccdc173	1731	NM 030680	Upf1	4598	NM 007579	Cacna1b	9655
NM 026376	Plxnd1	6913	NM 013690	Tek	4702	NM 001277970	Itga6	5888
NM 011416	Smarca2	5831	NM 001271421	Ephx2	2037	NM 001285937	Zdbf2	12648
NM 024448	Rab12	2010	NM 022030	Sv2a	3920	NM 207010	Mdga2	9813
NM 027415	Tmem70	1729	NM 023842	Dsp	9592	NM 007807	Cybb	4750
NM 001081309	Pik3r4	4884	NM 001277876	Tpm2	2108	NM 019466	Rcan1	2195
NM 177059	Fstl4	3117	NM 001081960	Clasp2	5627	NM 177259	Dab1	5344
NM 153579	Sv2b	5548	NM 024264	Cyp27a1	1890	NM 001112798	Slc8a1	18573
NM 001033270	Slc4a7	7389	NM 008070	Gabrb2	7568	NM 001042528	Cacna1b	9772
NM 173415	Nyx	3635	NM 001286600	Clasp2	5678	NM 001285416	Bdnf	4078
NM 001109753	Sv2b	5341	NM 009370	Tgfbr1	5735	NM 001285422	Bdnf	4123
NM 011121	Plk1	2203	NM 011102	Prkcg	3131	NM 001282000	Rbl2	4806
NM 008091	Gata3	3257	NM 009946	Cplx2	4928	NM 009716	Atf4	1746
NM 011364	Sh2d1a	819	NM 001291434	Prkcg	2978	NM 001277149	Chd7	11306
NM 001109661	Bach2	8493	NM 001291444	Pacs2	5486	NM 001285419	Bdnf	3855
NM 177668	Skint10	1729	NM 001291452	Lrrc7	7321	NM 146250	Gpr1	1798
NM 013672	Sp1	7818	NM 001081358	Lrrc7	7462	NM 001136077	Enpp2	3617
NM 009012	Rad50	5153	NM 009575	Zic3	4035	NM 001285420	Bdnf	3743
NM 007702	Cidea	1164	NM 001291453	Lrrc7	7184	NM 145434	Nr1d1	2782
NM 001040026	Sco1	4280	NM 172963	Mtcl1	7238	NM 001039079	Prkcz	4111
NM 008597	Mgp	614	NM 009734	Cep131	3589	NM 001285833	Nox4	3873
NM 010937	Nras	4470	NM 001291445	Pacs2	5390	NM 011323	Scn8a	11241
NM 001111053	Dclk1	6026	NM 001114098	Mtcl1	7297	NM 001285835	Nox4	3756
NM 019588	Plce1	9525	NM 001289652	Gid8	4412	NM 001289742	Lrrc4c	3860
NM 008858	Prkd1	3778	NM 001081170	Pacs2	5480	NM 018781	Egr3	3872
NM 001083967	Tcf4	7135	NM 029607	Gid8	4932	NM 011406	Slc8a1	18609
NM 001098230	Pdp1	4233	NM 013506	Eif4a2	2369	NM 011235	Rad51d	7072
NM 153142	Slc35e4	3059	NM 001123038	Eif4a2	3185	NM 015760	Nox4	3760
NM 010247	Xrcc6	2113	NM 001123037	Eif4a2	2229	NM 001286018	Klrk1	3197
NM 026038	L3hypdh	1705	NM 001038593	Glrx2	3506	NM 001282001	Rbl2	4905
NM 007505	Atp5a1	2443	NM 001038594	Glrx2	3445	NM 001289925	Egr3	3925
NM 001031808	Mrpl41	2423	NM 146171	Ncapd2	4527	NM 001277938	Rad51d	7057
NM 026002	Mtdh	3680	NM 178405	Atp1a2	6227	NM 010307	Gnal	5489

NM 148948	Dicer1	9851	NM 007591	Calr	1943	NM 001285936	Zdbf2	12705
NM 026392	Tmem70	1732	NM 018749	Eif3d	1910	NM 001083322	Klrk1	3179
NM 028794	Nudt9	1334	NM 001082961	Snrpn	2032	NM 001285417	Bdnf	3875
NM 030238	Dync1h1	14398	NM 001081249	Vcan	12432	NM 011250	Rbl2	4935
NM 026886	Srrm4	7476	NM 009072	Rock2	8012	NM 001289927	Egr3	3799
NM 011348	Sema3e	6877	NM 011436	Sorl1	6938	NM 178725	Lrrc4c	3492
NM 001042487	Dlgap4	3631	NM 007544	Bid	1906	NM 001285421	Bdnf	3941
NM 001025597	Ikzf1	5163	NM 001038592	Glrx2	3603	NM 001289743	Lrrc4c	3644
NM 001039048	Trim63	1886	NM 001001322	Adamts13	4580	NM 033078	Klrk1	3272
NM 008886	Pms2	3273	NM 001081110	Cd8a	3130	NM 001267695	Ctss	1360
NM 133217	Bco2	2093	NM 009597	Asic1	3800	NM 015744	Enpp2	3461
NM 178655	Ank2	5790	NM 009469	Ulk1	5215	NM 008397	Itga6	6018
NM 008796	Pctp	1971	NM 001017525	Btbd11	3955	NM 001077499	Scn8a	11340
NM 010120	Eif1a	2879	NM 026179	Abhd5	3148	NM 175770	Taf7	3302
NM 010338	Gpr37	3205	NM 010323	Gnrhr	1220	NM 001277266	Adam17	4508
NM_013685	Tcf4	7462	NM_027040	1700007K13Rik	833	NM_001291871	Adam17	4470
NM 013630	Pkd1	14170	NM 010719	Lipe	3221	NM 138751	Tmem47	4082
NM 011245	Rasgrf1	4243	NM 007758	Cr2	6207	NM 009615	Adam17	4451
NM 001111052	Dclk1	7030	NM 178793	Ccbe1	5742	NM 028747	Them7	1598
NM 008171	Grin2b	7515	NM 012013	Figla	759	NM 001081020	Adamts6	4822
NM_021500	Maea	2128	NM_028995	Nipal3	5025	NM_001162906	2410089E03Rik	10551
NM 025574	Pyurf	5410	NM 001082960	Itgam	4682	NM 001110516	Samd11	2610
NM 001033453	Pdp1	4198	NM 152944	Mmp21	1858	NM 011482	Nhp2l1	1257
NM 001080118	Med1	6518	NM 178681	Dgkb	5544	NM 001081088	Lrp2	15467
NM 021050	Cftr	6305	NM 008401	Itgam	4679	NM 013539	Spsb2	1179
NM 001001445	Trpv1	2520	NM 145433	Mrm1	2490	NM 001033385	Tbc1d32	7269
NM 001045489	Mfge8	2032	NM 009904	Clgn	2307	NM 001159638	Them7	1513
NM 146150	Nrd1	4155	NM 001012638	Acd	1425	NM 008882	Plxna2	11049
NM 007537	Bcl2l2	3476	NM 146200	Eif3c	2896	NM 009624	Adcy9	7840
NM 053102	15-Sep	1515	NM 007679	Cebpd	2260	NM 001291910	Adcy9	7632
NM 007462	Apc	12453	NM 145635	Adig	665	NM 182939	Ppp4r2	3641
NM 008594	Mfge8	2143	NM 146174	Fam115c	4257	NM 008367	Il2ra	4428
NM 009578	Ikzf1	4902	NM 001001932	Eea1	7836	NM 207654	Efna5	5259
NM 197979	Uqcr10	444	NM 010412	Hdac5	3813	NM 010467	Hoxd1	1878
NM 010478	Hspa1b	2810	NM 015771	Lats2	5213	NM 010855	Myh4	6037
NM 007567	Bsn	15953	NM 001082962	Snrpn	2076	NM 007865	Dll1	3444
NM 007913	Egr1	3072	NM 007545	Hrk	5366	NM 001164041	Smad5	6988
NM 177780	Dock5	10335	NM 001011707	Cyp2c66	1630	NM 013545	Ptpn6	2219
NM 011158	Prkar2b	3358	NM 011923	Angptl2	3444	NM 007523	Bak1	2017
NM 019978	Dclk1	7865	NM 001081290	Prrc2c	10444	NM 183197	Serpinb9f	1920
NM 010162	Ext1	3478	NM 001081271	Calhm1	1047	NM 008816	Pecam1	3305
NM 008813	Enpp1	3224	NM 013670	Snrpn	1970	NM 013720	Mga	13931
NM 025730	Lrrk2	8231	NM 023505	Glrx2	3482	NM 027030	Dcps	1194
NM 011237	Rad9a	2046	NM 001077696	Hdac5	3816	NM 027835	Ifih1	5519
NM 009383	Tial1	4292	NM 008587	Mertk	3564	NM 001081049	Kmt2a	16439
NM 013634	Med1	6383	NM 026271	Fibin	2069	NM 057171	Bag6	3858
NM 001098231	Pdp1	4149	NM 001252327	Pan2	4369	NM 013927	Cngb3	4708
NM 027498	Sik3	6296	NM 025626	Fam107b	3148	NM 001256224	Wnt5a	3731
NM 007856	Dhcr7	2845	NM 026449	Galntl5	1599	NM 010137	Epas1	5352
NM 007842	Dhx9	4623	NM 001252326	Pan2	4423	NM 001206382	Mavs	2962
NM 028404	Top1mt	2011	NM 133992	Pan2	4450	NM 001110350	Sin3a	5130
NM 010865	Myoc	2093	NM 001099644	Htr3a	2071	NM 008737	Nrp1	5921
NM 029094	Pik3cb	4854	NM 007597	Canx	4292	NM 001161456	Cbfb	2776
NM 008160	Gpx1	1066	NM 133775	Il33	2534	NM 001014981	Wdr7	7109
NM 026910	Tnik	7146	NM 008962	Ptgdr	3187	NM 009810	Casp3	2605
NM 021099	Kit	5189	NM 009857	Cd8a	3099	NM 008077	Gad1	3231
NM 001163008	Tnik	7035	NM 009431	Ctr9	4317	NM 172301	Ccnb1	2316
NM 016741	Scarb1	2534	NM 013881	Ulk2	5813	NM 011374	St8sia1	8995
NM 001130444	Hras	2272	NM 027694	Golga7b	2830	NM 001081117	Mki67	10098
NM 010050	Dio2	5843	NM 011076	Abcb1a	4977	NM 011261	Reln	11702

NM 011400	Slc2a1	2573	NM 018870	Pgam2	906	NM 010927	Nos2	3990
NM 009297	Supt6	6201	NM 001134474	Vcan	7215	NM 001197322	Foxp1	6649
NM 001198826	App	3323	NM 001141983	Golga7b	2839	NM 009765	Brca2	10940
NM 010763	Man1a2	7960	NM 201389	Plec	15440	NM 001164042	Smad5	6608
NM 011179	Psap	2676	NM 027711	Iqgap2	5771	NM 019721	Mettl3	2035
NM 008513	Lrp5	5172	NM 201370	Wee2	2886	NM 144888	Mavs	3099
NM 001159571	Ephb4	4361	NM 145431	Nle1	1771	NM 021527	Mkks	3215
NM 023858	Mtmr2	3036	NM 027286	Ace2	3418	NM 009019	Rag1	6669
NM 001034871	Clpsl2	444	NM 009654	Alb	2043	NM 008541	Smad5	6585
NM 001198823	App	3377	NM 026415	Cysrt1	781	NM 009602	Chrnb2	5405
NM 023223	Cdc20	1793	NM 001002268	Gpr126	6505	NM 001252469	Bag6	3568
NM 001122733	Kit	5205	NM 001145886	Tiam1	7277	NM 001271584	Dnajc5	4378
NM 013612	Slc11a1	2304	NM 178594	Vtcn1	2622	NM 146254	Wdr78	3793
NM 008881	Plxna1	9045	NM 026217	Atg12	2492	NM 172512	Gabpb2	8606
NM 001142922	Tcf7l2	4138	NM 001110499	Canx	4288	NM 009414	Tph1	4581
NM 001159595	Ints8	4676	NM 008774	Pabpc1	2842	NM 009764	Brca1	6648
NM 080575	Acss1	3594	NM 018871	Ywhag	3592	NM 133752	Opa1	5948
NM 001146121	Psap	2673	NM 009699	Aqp2	1416	NM 009397	Tnfaip3	4437
NM 008284	Hras	2043	NM 013561	Htr3a	2089	NM 001077705	Ptpn6	2221
NM 001166027	Skint11	1393	NM 009621	Adamts1	4904	NM 001206383	Mavs	2696
NM 010144	Ephb4	4334	NM 001145887	Tiam1	4210	NM 001286981	Mkks	3003
NM 001142921	Tcf7l2	4029	NM 010791	Meox1	2235	NM 011249	Rbl1	4909
NM 001142923	Tcf7l2	4014	NM 007812	Cyp2a5	1746	NM 010065	Dnm1	3843
NM 011544	Tcf12	4707	NM 001111304	Tbc1d9	5864	NM 007667	Cdh8	4786
NM 013625	Pafah1b1	5540	NM 024441	Hspb2	910	NM 001077411	Gba	1779
NM 175750	Plxna4	12602	NM 001163540	Plec	15455	NM 001099635	Myh3	5992
NM 010602	Kcnj11	3115	NM 007808	Cycs	3059	NM 011623	Top2a	5217
NM 178112	Ints8	3434	NM 001002894	Nlrp14	3297	NM 001285914	Cdh8	3166
NM 001163476	Gins1	677	NM 009511	Vipr2	3403	NM 008094	Gba	1938
NM 011346	Sell	2341	NM 011264	Rev3l	10666	NM 001001309	Itga8	5782
NM 007471	App	3152	NM 008626	Mrc2	5801	NM 053202	Foxp1	7177
NM 001146124	Psap	2583	NM 001113518	Arhgef7	4665	NM 001286983	Mkks	1831
NM 001198824	App	3320	NM 007872	Dnmt3a	9735	NM 011632	Traf3	7164
NM 053269	Rad51c	2954	NM 010130	Emr1	3245	NM 001171052	Mta3	2673
NM 001142924	Tcf7l2	3465	NM 177600	Ccdc73	3944	NM 146095	Rorb	8755
NM 016671	Il27ra	2671	NM 001110500	Canx	4256	NM 011281	Rorc	2503
NM_001146122	Psap	2664	NM_198637	1700016K19Rik	941	NM_146146	Lepr	4126
NM 031247	Gimap3	2145	NM 009463	Ucp1	1644	NM 007378	Abca4	7268
NM 001142920	Tcf7l2	4037	NM 011489	Stat5b	5255	NM 008167	Grid2	3024
NM 008798	Pdcd1	1972	NM 017402	Arhgef7	4488	NM 001199177	Opa1	6002
NM 001110140	Atp2a2	4565	NM 178357	Klf11	3982	NM 010566	Inpp5d	4938
NM 001146123	Psap	2640	NM 009733	Axin1	3809	NM 134028	Tubg2	1777
NM 080853	Slc17a6	4338	NM 027185	Def6	2294	NM 022309	Cbfb	2893
NM 001142918	Tcf7l2	4163	NM 007776	Crygd	655	NM 009524	Wnt5a	4354
NM 009333	Tcf7l2	4065	NM 001127177	Ptpn2	8423	NM 198438	Ssbp3	3130
NM 001031772	Lin28b	5420	NM 011770	Ikzf2	9457	NM 008056	Fzd6	4090
NM 001163007	Tnik	7122	NM 145482	Setd4	1827	NM 001043354	Rorb	9289
NM 001037915	Ripply1	679	NM 001113517	Arhgef7	4946	NM 134024	Tubg1	1609
NM 001146120	Psap	2667	NM 009951	Igf2bp1	8382	NM 001285913	Cdh8	3203
NM 001205082	Scarb1	2405	NM 010344	Gsr	2692	NM 175367	Ston2	9896
NM 001205083	Scarb1	2196	NM 001025602	Il1rl1	5080	NM 001161458	Cbfb	2924
NM 001130443	Hras	2190	NM 007390	Chrna7	2091	NM 001164274	Mga	13304
NM 001198825	App	3266	NM 001113563	Stat5b	4923	NM 021896	Gucy1a3	4671
NM 001142919	Tcf7l2	4112	NM 011488	Stat5a	3910	NM 001289546	Amph	3247
NM 028882	Sema3d	6521	NM 011499	Strap	2644	NM 010109	Efna5	5178
NM 001168304	Cdk19	5828	NM 008580	Map3k5	5393	NM 016780	Itgb3	5795
NM 001291817	Cdk19	5770	NM 023662	Pcm1	8398	NM 016961	Mapk9	4682
NM 198164	Cdk19	5696	NM 011516	Sycp1	3437	NM 001289921	Rorb	8808
NM 001291816	Cdk19	5752	NM 001130513	Ace2	3566	NM 008002	Fgf10	4572
NM 025436	Msmo1	1824	NM 008608	Mmp14	2597	NM 144783	Wt1	3092
NM 001114879	Fam208a	7307	NM 025757	Gid4	4437	NM 001110193	Inpp5d	4755

NM 001195579	Trpc5os	3581	NM 001164062	Stat5a	3628	NM 009828	Ccna2	2827
NM 028945	Fam208a	7581	NM 144547	Amhr2	1947	NM 001252468	Bag6	3804
NM 134063	Fam208b	8356	NM 009826	Rb1cc1	7046	NM 001166402	Tnfaip3	4352
NM 020259	Hhip	9094	NM 172802	Fscn2	1718	NM 007561	Bmpr2	11094
NM 001077404	Nrp2	6724	NM 029770	Unc5b	5867	NM 001081306	Ptprz1	8068
NM 008898	Por	2457	NM 010113	Egf	4757	NM 139146	Satb2	5299
NM 010939	Nrp2	6688	NM 001033471	Cpxcr1	1596	NM 053069	Atg5	2352
NM 001109749	Cntn4	5262	NM 024215	Zfp593	1991	NM 178143	Prkaa2	8201
NM 001166667	Rtel1	4316	NM 007703	Elovl3	1879	NM 001081001	Brca2	11134
NM 007460	Ap3d1	4730	NM 013569	Kcnh2	4221	NM 001271585	Dnajc5	4405
NM 009050	Ret	6064	NM 008252	Hmgb2	2692	NM 139304	Gatad2b	1912
NM 001166668	Rtel1	4208	NM 019925	Gpr132	2439	NM 001081432	Ptprq	7058
NM 013454	Abca1	10260	NM 029182	Rasd2	2810	NM 008192	Gucy2e	8331
NM 001024673	Ifnl2	582	NM 008872	Plat	2548	NM 207692	Mapk9	4677
NM 011412	Slit3	5017	NM 001012765	Adcy5	5060	NM 019579	Mpp5	5529
NM 001077403	Nrp2	6739	NM 028709	Btbd11	5808	NM 021433	Stx6	2459
NM 010811	Ndst2	3907	NM 009758	Bmpr1a	5481	NM 009829	Ccnd2	5772
NM 009425	Tnfsf10	4944	NM 009794	Capn2	3205	NM 177369	Myh8	6149
NM 001025439	Camk2d	4299	NM 009867	Cdh4	6393	NM 001161457	Cbfb	2797
NM 016701	Nes	6143	NM 007907	Eef2	3126	NM 175007	Amph	3235
NM 001141975	Tpx2	4161	NM 001164724	Il33	2555	NM 001110351	Sin3a	4998
NM 010288	Gja1	3105	NM 001281859	Cysltr1	2960	NM 139294	Braf	9728
NM 194346	Rnf31	3443	NM 010825	Meis2	4621	NM 173740	Maoa	4161
NM 207239	Gtf3c1	6891	NM 153587	Rps6ka5	4406	NM 028136	Dhx36	4975
NM 009409	Top2b	5652	NM 178666	Themis	3981	NM 011723	Xdh	4623
NM 008884	Pml	5240	NM 001286718	Tsc2	6134	NM 007396	Acvr2a	5681
NM 016679	Keap1	4470	NM 001159567	Meis2	4737	NM 001206385	Mavs	2871
NM 011609	Tnfrsf1a	2186	NM 009622	Adcy1	12259	NM 001136084	Tph1	4270
NM 011169	Prlr	10328	NM 023735	Actr3	2746	NM 009693	Apob	13931
NM 010147	Epn1	2582	NM 010220	Fkbp5	3884	NM 013722	Syn3	8797
NM 001077405	Nrp2	6673	NM 001286064	Grk6	3001	NM 001039154	Cdh8	3822
NM 001141976	Tpx2	4177	NM 001281976	Ctla4	1823	NM 001163672	Mapk9	4682
NM 023913	Ern1	3976	NM 001252470	Cpt1c	2809	NM 001032378	Pecam1	3248
NM 001110306	Keap1	3319	NM 009384	Tiam1	7312	NM 008937	Prox1	4148
NM 010954	Ncam2	4893	NM 033325	Loxl2	5186	NM 011159	Prkdc	12674
NM 001025438	Camk2d	4286	NM 011938	Grk6	2100	NM 001163671	Mapk9	4677
NM 001252330	Slc6a15	3563	NM 010598	Kcnab2	3604	NM 001284409	Casp3	2610
NM 021420	Stk4	5189	NM 029466	Arl5b	3594	NM 001110192	Inpp5d	4935
NM 007499	Atm	11964	NM 001191027	Dync1i1	2658	NM 007700	Chuk	3500
NM 009916	Ccr4	2787	NM 001080971	Tubb1	2004	NM 008964	Ptger2	3768
NM 001077407	Nrp2	4696	NM 001002011	Lmna	3189	NM 001162494	Fzd6	4086
NM 001110305	Keap1	3434	NM 001122950	Hoxa10	2177	NM 001013367	Prkaa1	4655
NM 007500	Atoh1	2118	NM 009938	Copa	4629	NM 001164477	Ifih1	5372
NM 178087	Pml	5378	NM 001037298	Piezo1	8216	NM 011378	Sin3a	5229
NM 001001882	Rtel1	4451	NM 001114079	Pabpc1l	2580	NM 023672	Ssbp3	3211
NM 001252454	Epn1	2411	NM 011775	Zp2	2200	NM 029216	Chd5	9375
NM 011828	Hs2st1	4811	NM 001112703	Abl1	7162	NM 008656	Myf5	2083
NM 001110307	Keap1	3151	NM 001042620	Dhx15	3009	NM 001286122	Traf3	6978
NM 001141977	Tpx2	4365	NM 001286066	Grk6	2093	NM 001081376	Chd5	9486
NM 028109	Tpx2	4352	NM 011647	Tsc2	6266	NM 016775	Dnajc5	4325
NM 023813	Camk2d	4197	NM 001284249	Hdac5	3925	NM 001162410	Chuk	3483
NM 173004	Cntn4	2931	NM 001004062	Crtc1	5674	NM 029885	Gabpb2	8703
NM 010664	Krt18	1400	NM 001033420	Dock1	6815	NM 001197321	Foxp1	7042
NM 010496	Id2	1289	NM 001284250	Hdac5	3817	NM 053247	Lyve1	2607
NM 175328	Slc6a15	3660	NM 001159598	Axin1	3671	NM 207243	Muc19	22806
NM 008960	Pten	8229	NM 001205386	Actr3	2551	NM 011426	Siglec1	6427
NM 001109751	Cntn4	3019	NM 001252654	Kcnab2	3562	NM 001163493	Stard13	5638
NM 007734	Col4a3	8609	NM 001256145	Rad54b	4483	NM 146258	Stard13	5581
NM 001166666	Rtel1	4334	NM 009537	Yy1	2324	NM 001005248	Hps5	4804
NM 008261	Hnf4a	4371	NM 001039363	Tsc2	6137	NM 001290640	Dab2ip	6380
NM 008078	Gad2	5625	NM 130869	Nobox	1893	NM 001160403	Il1rapl1	2739

NM_008610	Mmp2	3070	NM_001163728	4930563E22Rik	3313	NM_170689	Ank3	9971
NM 001166665	Rtel1	4433	NM 009843	Ctla4	1933	NM 001290636	Dock7	7139
NM 008524	Lum	2075	NM 194262	Arid4b	6113	NM 001123382	Il1r1	4808
NM 001141978	Tpx2	4164	NM 148925	Fyco1	7949	NM 170690	Ank3	9962
NM 011749	Zfp148	9431	NM 175210	Abca12	8323	NM 007601	Capn3	3167
NM 009887	Cer1	1742	NM 001199676	Camkk2	4903	NM 001136086	Dpysl3	5252
NM 001077406	Nrp2	4711	NM 001159570	Meis2	4989	NM 001291432	Dido1	4717
NM 001080780	Ret	7341	NM 028451	Larp1	6617	NM 011602	Tln1	8560
NM 009915	Ccr2	3589	NM 010000	Cyp2b9	1877	NM 001109761	Capn3	2624
NM 008245	Hhex	1771	NM 001190870	Kcne3	1333	NM 001290486	Lpar1	3247
NM 001033481	Myrf	5613	NM 008851	Pitpnm1	4533	NM 001256001	Cacna1c	13643
NM 133828	Creb1	8389	NM 001284248	Hdac5	4332	NM 022432	Sirt2	1863
NM 181414	Pik3c3	3102	NM 001191026	Dync1i1	2718	NM 011331	Ccl12	537
NM 001271500	Il15ra	1466	NM 025663	Gpatch4	1771	NM 001111023	Runx1	7138
NM 172524	Nipal4	3295	NM 001191025	Dync1i1	2691	NM 001109743	Skor2	3141
NM 010696	Lcp2	3348	NM 001111102	Lmna	2086	NM 178220	Arrb1	7088
NM 009534	Yap1	4152	NM 153403	Ago1	7065	NM 011388	Slc10a2	1629
NM 001163530	Prl	892	NM 019390	Lmna	1537	NM 008891	Pnn	3469
NM 001099298	Scn2a1	8690	NM 001286857	Gpatch4	2250	NM 001113389	Dyrk1a	5776
NM 146261	Fam199x	8308	NM 010638	Klf9	3263	NM 010227	Flna	8347
NM 008125	Gjb2	2404	NM 008745	Ntrk2	7049	NM 009821	Runx1	6946
NM 010931	Uhrf1	3583	NM 001103158	Gm13242	4003	NM 008904	Ppargc1a	6464
NM 001271993	Fut2	3073	NM 001277926	Casp8	2528	NM 001290639	Dab2ip	6075
NM 001253862	Tcf12	4578	NM 001136072	Meis2	4716	NM 022983	Lpar3	2494
NM 011045	Pcna	1260	NM 177386	Sfmbt2	7850	NM 010889	Neb	22489
NM 001159555	Cd36	3539	NM 001190871	Kcne3	1097	NM 001291068	Polr2a	6736
NM 178710	Sik2	3561	NM 001281862	Cysltr1	2802	NM 026082	Dock7	7049
NM 023635	Rab27a	2971	NM 146176	Cnot3	2923	NM 019680	Elf4	5847
NM 028673	Zdbf2	12621	NM 153178	Ago2	8031	NM 012016	Ern2	2973
NM 023566	Muc2	7368	NM 001190950	Kcne3	816	NM 147779	Sftpb	1564
NM 029210	Sv2c	4252	NM 001110809	Gpatch4	1636	NM 001255997	Cacna1c	13340
NM 001013833	Prkg1	6992	NM 021476	Cysltr1	2979	NM 010332	Ednra	3643
NM 013628	Pcsk1	2516	NM 001205385	Actr3	2554	NM 001163700	Nr1h4	1985
NM 001164192	Mtm1	3286	NM 001159568	Meis2	4642	NM 001159535	Cacna1c	13586
NM 008006	Fgf2	695	NM 001286063	Grk6	2996	NM 011842	Mta2	3149
NM 133854	Snapin	1916	NM 001286720	Tsc2	6353	NM 146005	Ank3	10025
NM 013838	Trpc6	3259	NM 001283046	Abl1	7367	NM 010747	Lyn	3393
NM 001195539	Dclk1	7004	NM 145077	Ucn2	1002	NM 009781	Cacna1c	13340
NM 007649	Cd48	1142	NM 001286714	Tsc2	6261	NM 001204134	C1qtnf3	2544
NM 177357	Kalrn	15439	NM 001159569	Meis2	5010	NM 010141	Epha7	6750
NM 019981	Tex101	1054	NM 001112711	Grk6	3014	NM 001114124	Dab2ip	6540
NM 009925	Col10a1	3139	NM 153795	Fermt3	2587	NM 011805	Dido1	4769
NM 175035	Gimap5	1943	NM 198122	Arid4b	5852	NM 020009	Mtor	8612
NM 001253366	Kcnma1	4979	NM 145358	Camkk2	4860	NM 053084	Trim32	3196
NM 013892	Pcsk1n	2208	NM 001029985	Kcp	4905	NM 010177	Fasl	1935
NM 007472	Aqp1	2760	NM 153679	Cpt1c	2811	NM 007901	S1pr1	3029
NM 029658	Fam101b	3533	NM 001286065	Grk6	2912	NM 170728	Ank3	9320
NM 013703	Vldlr	8327	NM 001286716	Tsc2	6132	NM 008413	Jak2	5055
NM 178719	Mief1	5099	NM 183138	Tet3	10907	NM 001285980	Skp2	3140
NM 009871	Cdk5r1	4166	NM 001276450	Ahsg	1503	NM 199239	Sema6d	5627
NM 016849	Irf3	2053	NM 001286713	Tsc2	6335	NM 133990	Il13ra1	3686
NM 011912	Vax2	1233	NM 009438	Rpl13a	1039	NM 198052	Tbx3	4783
NM 017382	Rab11a	2333	NM 001038018	Grk6	3016	NM 001290424	Fmr1	4351
NM 001159557	Cd36	3450	NM 001276449	Ahsg	1122	NM 001290997	Sema6d	6178
NM 144859	Pja2	4520	NM 007818	Cyp3a11	2053	NM 001290335	Cacna1c	12656
NM 026742	Ndufaf4	3632	NM 009497	Vamp2	2164	NM 177231	Arrb1	7112
NM 001037726	Creb1	8267	NM 021367	Tslp	1143	NM 010485	Elavl1	6030
NM 010568	Insr	9357	NM 001191023	Dync1i1	2742	NM 026509	Murc	2062
NM 001128170	Cyld	8023	NM 026346	Fbxo32	6936	NM 001161782	Trim32	3193
NM 001199136	Macf1	23495	NM 001198808	Sfmbt2	7826	NM 009127	Scd1	4844
NM 011163	Eif2ak2	4343	NM 011638	Tfrc	4920	NM 148945	Rps6ka3	7320

NM 010173	Faah	3816	NM 001252655	Kcnab2	3591	NM 172537	Sema6d	6335
NM 001253371	Kcnma1	5031	NM 010063	Dync1i1	2751	NM 007548	Prdm1	5143
NM 138301	Trpm2	7292	NM 172473	Hace1	3785	NM 001025947	Dnm1l	4034
NM 009652	Akt1	2707	NM 001190869	Kcne3	1332	NM 008714	Notch1	9497
NM 008358	Il15ra	1664	NM 013465	Ahsg	1524	NM 027384	Tet1	13986
NM 023128	Palm	2651	NM 013488	Cd4	3095	NM 013787	Skp2	3210
NM 001253365	Kcnma1	4982	NM 001283047	Abl1	7306	NM 170729	Ank3	9908
NM 022984	Retn	1139	NM 153743	Dnmt3a	8913	NM 009670	Ank3	6966
NM 008716	Notch3	7943	NM 177330	Ghsr	4433	NM 001159533	Cacna1c	13592
NM 021297	Tlr4	3847	NM 020574	Kcne3	1031	NM 001111021	Runx1	5803
NM 001253358	Kcnma1	5063	NM 001271753	Dnmt3a	9689	NM 170687	Ank3	7554
NM 001256115	Sun1	4022	NM 007839	Dhx15	3061	NM 009468	Dpysl3	5433
NM 172689	Ddx58	4943	NM 001110253	Fyco1	7702	NM 001048177	Jak2	4972
NM 001253362	Kcnma1	5130	NM 001252656	Kcnab2	3633	NM 011443	Sox2	2457
NM 001171147	Yap1	4200	NM 001013028	Tmem263	3778	NM 001160415	Apobec3	2459
NM 001081414	Grm5	8428	NM 198861	Lrrc75a	2047	NM 144802	Hnrnpll	3050
NM 007429	Agtr2	2872	NM 001289651	Gid8	4446	NM 139144	Ogt	5415
NM 008026	Fli1	3087	NM 139147	Rab40b	1865	NM 008719	Npas2	4180
NM 001025432	Crebbp	7507	NM 001172117	Hck	2107	NM 001177656	Grin1	3922
NM 170779	Wwc1	3315	NM 001291777	Map2k7	1672	NM 001111022	Runx1	5611
NM 007399	Adam10	4605	NM 001291778	Map2k7	1563	NM 001163504	Nr1h4	2189
NM 020256	Zbtb33	5122	NM 001164172	Map2k7	3556	NM 199241	Sema6d	5756
NM 009933	Col6a1	3990	NM 011944	Map2k7	3515	NM 145840	Rgs9bp	6604
NM 010408	Hcn1	7911	NM 001291783	Map2k7	3394	NM 008360	Il18	866
NM 001199137	Macf1	17414	NM 146121	Rabgap1	4967	NM 007868	Dmd	13857
NM 001164191	Mtm1	3379	NM 007931	Endog	1032	NM 001284410	Bcl2l11	2327
NM 001253363	Kcnma1	5127	NM 133786	Smc4	4101	NM 001290637	Dab2ip	4152
NM 025427	Rgcc	934	NM 198628	Stkld1	2198	NM 008031	Fmr1	4426
NM 019926	Mtm1	3368	NM 025531	Slmo2	1403	NM 030888	C1qtnf3	2350
NM 024197	Ndufa10	1222	NM 134023	Tbc1d10a	1929	NM 010414	Htt	13237
NM 001161420	Vldlr	8243	NM 009444	Tgoln2	2265	NM 001177657	Grin1	3859
NM 018876	Fut2	2969	NM 001033786	Smim9	703	NM 001005247	Hps5	4828
NM 001163743	Nlrx1	3706	NM 177864	Skint9	1373	NM 001177799	Capn3	2891
NM 028390	Anln	5421	NM 172285	Plcg2	4345	NM 007417	Adra2a	3818
NM 001164190	Mtm1	3488	NM 009500	Vav2	3498	NM 001167923	Col6a5	9297
NM 001271501	Il15ra	1373	NM 021308	Piwil2	4913	NM 152816	Dnm1l	4073
NM 001146200	Pik3cg	6634	NM 201368	Xkr8	4238	NM 001282071	Sftpb	1492
NM 001252498	Rapgef6	4843	NM 027660	Tekt3	1739	NM 030255	Apobec3	2360
NM 013552	Hmmr	3914	NM 011942	Lypla2	1593	NM 008689	Nfkb1	4128
NM 001111080	Uhrf1	3415	NM 177239	Mysm1	7420	NM 001159534	Cacna1c	13592
NM 001128171	Cyld	8148	NM 026370	Kat8	1503	NM 008512	Lrp1	14907
NM 028224	Faim2	4668	NM 010218	Fjx1	2446	NM 199240	Sema6d	5699
NM 001253864	Tcf12	3957	NM 001024702	C1ql4	717	NM 007453	Prdx6	2334
NM 028643	Micu2	2310	NM 198608	Aars2	3368	NM 001077514	Slc1a2	11571
NM 001256116	Sun1	3878	NM 030728	Cemip	7114	NM 001253857	Tet1	14082
NM 007475	Rplp0	1360	NM 001039243	Erich4	910	NM 009108	Nr1h4	1973
NM 001276301	Ampd3	3979	NM 008109	Gdf5	2317	NM 001291016	Bcl2l11	2304
NM 001253360	Kcnma1	5051	NM 177572	Rimkla	4093	NM 001290421	Flna	8369
NM 001003918	Usp7	5427	NM 001168297	Fbxo30	4943	NM 010508	Ifnar1	7025
NM 010431	Hif1a	4775	NM 001100183	Cyp4a29	1530	NM 170730	Ank3	6656
NM 021274	Cxcl10	1120	NM 027968	Fbxo30	4977	NM 001167864	Hps5	4729
NM 025377	Ska2	1349	NM 001290273	1-Mar	2148	NM 001122766	Sirt2	1653
NM 001253377	Kcnma1	4944	NM 001077354	C77370	10891	NM 016678	Reck	4450
NM 001163028	Bcmo1	2532	NM 027664	Rimklb	4017	NM 001291000	Sema6d	6509
NM 008987	Ptx3	1924	NM 207215	Mycbp2	15269	NM 001276341	Dnm1l	3829
NM 001256117	Sun1	3764	NM 011075	Abcb1b	4344	NM 031178	Tlr9	3471
NM 013598	Kitl	5450	NM 019459	Nphs1	5661	NM 001290434	Epha7	6738
NM 011857	Tenm3	10978	NM 145423	Slc5a8	5346	NM 008169	Grin1	4326
NM 001253865	Tcf12	4204	NM 009632	Parp2	1834	NM 001290535	Ogt	5218
NM 001253369	Kcnma1	5037	NM 001113180	Gria4	5458	NM 001077515	Slc1a2	10998
NM 138652	Atp12a	3954	NM 009722	Atp2a2	4353	NM 008362	Il1r1	4739
NM 010870	Naip5	5368	NM 144534	Tmem38a	2109	NM 001111096	Lyn	3456

NM 145979	Chd4	6438	NM 001008700	Il4ra	5122	NM 001081304	Atf6	7463
NM 011631	Hsp90b1	2759	NM 177595	Mkx	3201	NM 007890	Dyrk1a	6035
NM 021486	Bcmo1	2327	NM 030683	Slc14a2	2054	NM 001276340	Dnm1l	4085
NM 010157	Esr2	3308	NM 138606	Pim2	2054	NM 170688	Ank3	9437
NM 173369	Cyld	8014	NM 009868	Cdh5	4004	NM 001290641	Dab2ip	6673
NM 024451	Sun1	4133	NM 007993	Fbn1	9900	NM 001255998	Cacna1c	13415
NM 001168253	Fam83h	4545	NM 008691	Nefm	3244	NM 013735	Trp53bp1	9652
NM 001079513	Zbtb33	4948	NM 001111051	Dclk1	6956	NM 199238	Sema6d	6296
NM 011526	Tagln	1586	NM 008036	Fosb	3783	NM 001256002	Cacna1c	13841
NM 010586	Itpr2	11740	NM 008605	Mmp12	3607	NM 001255999	Cacna1c	13340
NM 001101471	Akap5	6685	NM 029745	Tbc1d9b	5225	NM 011535	Tbx3	4647
NM 001195540	Dclk1	5175	NM 172669	Ambra1	5329	NM 008339	Cd79b	1232
NM 011160	Prkg1	6915	NM 007824	Cyp7a1	4172	NM 001122765	Sirt2	1816
NM 009506	Vegfc	1881	NM 008829	Pgr	6889	NM 025295	Btd	1956
NM 007552	Bmi1	3594	NM 011416	Smarca2	5831	NM 054071	Fgfrl1	2656
NM 009320	Slc6a6	6169	NM 011035	Pak1	3081	NM 001256000	Cacna1c	13634
NM 020272	Pik3cg	6811	NM 001081309	Pik3r4	4884	NM 001290830	Trp53bp1	9502
NM 001253372	Kcnma1	5031	NM 207651	Slc14a2	4045	NM 001205243	Fasl	1889
NM 001199249	Kat5	1423	NM 153579	Sv2b	5548	NM 019930	Ranbp9	3064
NM 177781	Trpa1	4263	NM 001033270	Slc4a7	7389	NM 001291455	Dpysl3	5502
NM 001025309	Pja2	4706	NM 010242	Fut4	3664	NM 008949	Psmc3ip	984
NM 009502	Vcl	5229	NM 001109753	Sv2b	5341	NM 001104543	Vmn2r94	2529
NM 001040398	Setd1b	8868	NM 025540	Sln	536	NM 001081405	Vmn2r28	2744
NM 009504	Vdr	4354	NM 008363	Irak1	3831	NM 053146	Pcdhb21	2712
NM 010513	Igf1r	11978	NM 008679	Ncoa3	7571	NM 053141	Pcdhb16	5278
NM 001161747	Palm	2519	NM 001110267	Vegfa	2785	NM 001085544	Gm14744	800
NM_001252481	Smad2	8863	NM_176829	4931440F15Rik	3274	NM_001270900	Gm8764	1217
NM 019923	Itpr2	11839	NM 009012	Rad50	5153	NM 001105074	Vmn2r44	2586
NM 008899	Pou3f2	6272	NM 025455	Ccdc28b	856	NM 001105070	Vmn2r38	2586
NM 013476	Ar	2999	NM 001040026	Sco1	4280	NM 001205282	Gm14496	2550
NM 011034	Prdx1	1417	NM 207525	Opa3	2260	NM 001104551	Vmn2r99	2571
NM 001033367	Nlrc4	3838	NM 001033238	Cblb	6323	NM 001126325	Gm13088	1819
NM 181753	Opn5	1824	NM 008071	Gabrb3	5568	NM 001012266	Olfr835	936
NM_024469	Bhlhe41	5995	NM_001001982	A430105I19Rik	4716	NM_146824	Olfr273	954
NM 001267872	Zdbf2	12571	NM 007976	F5	7406	NM 146826	Olfr969	936
NM_011724	Xirp1	5839	NM_010937	Nras	4470	NM_001011734	Olfr1116-ps	924
NM 178420	Nlrx1	3694	NM 019426	Atf7ip	4543	NM 207560	Olfr924	927
NM 203491	Chrm2	2036	NM 145383	Rho	3249	NM 134203	Vmn1r73	912
NM 133872	Kdm1a	3030	NM 019588	Plce1	9525	NM 146841	Olfr617	957
NM 001146201	Pik3cg	6690	NM 001112697	Chrm1	6301	NM 207575	Olfr1480	948
NM 144549	Trib1	4026	NM 029972	Ermn	3550	NM 001005568	Olfr1281	918
NM 001159556	Cd36	3399	NM 019552	Abcb10	4325	NM 147110	Olfr570	939
NM 025788	Nacc1	4332	NM 008858	Prkd1	3778	NM 146605	Olfr828	939
NM 177909	Slc9a9	3492	NM 001025257	Vegfa	3343	NM 053251	Gm4736	1027
NM 001271499	Il15ra	1565	NM 001025250	Vegfa	3547	NM 146346	Olfr397	948
NM 001204959	Retn	607	NM 007738	Col7a1	9222	NM 146608	Olfr984	945
NM 008859	Prkcq	3313	NM 028773	Sash3	2584	NM 146862	Olfr763	930
NM 001163742	Nlrx1	3605	NM 001042655	Tbc1d17	2601	NM 001011767	Olfr299	993
NM 001145937	Tenm3	10958	NM 011376	Sim1	7355	NM 134226	Vmn1r89	951
NM 001081111	Tmf1	6781	NM 001082531	Pla2g2a	793	NM 146347	Olfr390	936
NM 001143834	Grm5	8524	NM 001031808	Mrpl41	2423	NM 134231	Vmn1r185	960
NM 183276	Nbeal2	8803	NM 148948	Dicer1	9851	NM 001011773	Olfr1287	918
NM 001159558	Cd36	3566	NM 144918	Smyd5	2507	NM 146621	Olfr308	927
NM 153079	Nmur2	3276	NM 178650	Tbc1d10c	1781	NM 134245	Vmn1r216	897
NM 010610	Kcnma1	5118	NM 025836	Plin3	2159	NM 001012265	Olfr857	930
NM 205783	Chrm5	1746	NM 028794	Nudt9	1334	NM 146372	Olfr509	966
NM 001253361	Kcnma1	5139	NM 026825	Lrrc16a	5158	NM 001011792	Olfr207	918
NM 023544	Rsc1a1	2154	NM 030238	Dync1h1	14398	NM 146636	Olfr1487	948
NM 181420	Fn3krp	2028	NM 007386	Aco1	4319	NM 146637	Olfr1141	936

NM	009541	Zbtb17	3415	NM	007698	Chrm1	6209	NM	146377	Olfr127	972
NM	001111078	Uhrf1	3439	NM	001110513	Ebf4	2687	NM	146380	Olfr593	984
NM	007949	Ercc2	3547	NM	009505	Vegfa	3475	NM	001011799	Olfr453	954
NM	001253378	Kcnma1	4941	NM	020568	Plin4	5755	NM	146390	Olfr1323	930
NM	009952	Creb1	8431	NM	008886	Pms2	3273	NM	146904	Olfr870	936
NM	010934	Npy1r	3007	NM	020277	Trpm5	4383	NM	001011808	Olfr198	921
NM	001111079	Uhrf1	3559	NM	009812	Casp8	2585	NM	001011809	Olfr728	936
NM	007643	Cd36	3542	NM	178655	Ank2	5790	NM	146653	Olfr435	942
NM	001204411	Kcnj11	2667	NM	008473	Krt1	2462	NM	146906	Olfr853	918
NM	010754	Smad2	8752	NM	013630	Pkd1	14170	NM	146398	Olfr1325	948
NM	019757	Fzr1	2258	NM	011245	Rasgrf1	4243	NM	146911	Olfr1377	924
NM	001165894	Akt1	2657	NM	001111052	Dclk1	7030	NM	146403	Olfr1295	939
NM	009257	Serpinb5	2565	NM	011594	Timp2	3635	NM	146407	Olfr1079	948
NM	001253863	Tcf12	4029	NM	001040396	Selt	3575	NM	001011825	Olfr1105	939
NM	027011	Krt5	2190	NM	008170	Grin2a	4512	NM	001011826	Olfr967	933
NM	008292	Hsd17b4	2685	NM	001110268	Vegfa	2653	NM	146670	Olfr815	951
NM	001271498	Il15ra	1695	NM	009593	Abcg1	5821	NM	001011828	Olfr304	1002
NM	001256118	Sun1	3602	NM	021500	Maea	2128	NM	001011829	Olfr761	972
NM	178060	Thra	2246	NM	001080126	Casp8	2412	NM	146928	Olfr808	939
NM	133836	Il15ra	1717	NM	010493	Icam1	2540	NM	146675	Olfr820	939
NM	001253373	Kcnma1	4962	NM	001110266	Vegfa	2857	NM	146681	Olfr1424	942
NM	011164	Prl	895	NM	026559	Txndc17	1446	NM	001011841	Olfr1465	924
NM	001253359	Kcnma1	5060	NM	018763	Chst2	6256	NM	146433	Olfr994	945
NM	030559	Vps16	3206	NM	009706	Arhgap5	5197	NM	146688	Olfr262	939
NM	001038658	Faim2	4632	NM	001034168	Ank2	5487	NM	146434	Olfr995	948
NM	025481	Smurf2	5346	NM	028782	Lonp1	2970	NM	146435	Olfr993	945
NM	001276279	Cyld	7599	NM	011240	Ranbp2	9477	NM	146692	Olfr1454	924
NM	001195538	Dclk1	7817	NM	011743	Zfp106	9100	NM	146947	Olfr338	921
NM	177730	Impad1	4636	NM	146150	Nrd1	4155	NM	207236	Olfr1283	918
NM	001253367	Kcnma1	4970	NM	173752	Lgalsl	3635	NM	001011853	Olfr1425	936
NM	134087	Fam83h	4513	NM	001002927	Penk	1442	NM	146443	Olfr382	939
NM	001039546	Myo6	7868	NM	197979	Uqcr10	444	NM	146952	Olfr522	939
NM	022025	Slc5a7	5198	NM	009635	Avil	3033	NM	146956	Olfr525	930
NM	009792	Camk2a	4268	NM	010575	Itga2b	3437	NM	001011860	Olfr1222	936
NM	019439	Gabbr1	4486	NM	001007572	Trpv5	2601	NM	021368	Olfr1264	927
NM	001253374	Kcnma1	4820	NM	007567	Bsn	15953	NM	146451	Olfr164	948
NM	207707	Esr2	3362	NM	172644	Dars2	3581	NM	001011866	Olfr309	927
NM	176979	Topbp1	5112	NM	011066	Per2	5845	NM	001011868	Olfr1178	972
NM	024200	Mfn1	4531	NM	019978	Dclk1	7865	NM	146458	Olfr1199	933
NM	172627	Pggt1b	2736	NM	021501	Pias4	1676	NM	207254	Olfr1286	918
NM	001271768	Bhlhe41	6124	NM	008418	Kcna3	1968	NM	146971	Olfr1228	972
NM	033374	Dock2	6431	NM	001080754	Ambra1	5056	NM	146466	Olfr165	942
NM	021281	Ctss	1357	NM	009383	Tial1	4292	NM	146983	Olfr1256	921
NM	052977	Adarb2	5633	NM	172931	Hsf3	1972	NM	146731	Olfr599	948
NM	008854	Prkaca	2300	NM	019691	Gria4	5458	NM	146993	Olfr176	957
NM	001110830	Rbfox2	6562	NM	010824	Mpo	2570	NM	146750	Olfr689	963
NM	001284394	4-Sep	1670	NM	011693	Vcam1	3398	NM	147005	Olfr395	939
NM	001282087	Trpc6	3235	NM	001177846	Tirap	4731	NM	146760	Olfr672	939
NM	175387	Rbfox2	6980	NM	021099	Kit	5189	NM	146508	Olfr959	936
NM	001282086	Trpc6	3025	NM	054096	Tirap	4741	NM	147021	Olfr1055	948
NM	001286684	Slc8a1	15847	NM	012045	Pla2g2f	2437	NM	146514	Olfr96	942
NM	008860	Prkcz	4311	NM	001081499	Tbc1d8b	5907	NM	146769	Olfr1110	939
NM	053104	Rbfox2	7032	NM	175001	Mrpl22	753	NM	147026	Olfr532	930
NM	007715	Clock	9801	NM	011400	Slc2a1	2573	NM	146522	Olfr854	948
NM	001286419	Rbfox2	6469	NM	009297	Supt6	6201	NM	146527	Olfr849	939
NM	001136079	Ptger4	3327	NM	001198826	App	3323	NM	146272	Olfr930	927
NM	011861	Pacsin1	4215	NM	080849	Nek8	2832	NM	145843	Vmn1r72	921
NM	001289826	Clock	9758	NM	001177847	Tirap	4677	NM	146291	Olfr1484	948
NM	001289533	Fhl2	1417	NM	025374	Glo1	1958	NM	147064	Olfr449	936
NM	011129	4-Sep	1723	NM	001162475	Stra6	2784	NM	147067	Olfr829	966
NM	001277898	Prkaca	2257	NM	153159	Zc3h12a	2805	NM	207549	Olfr192	924
NM	001285488	Mknk1	2598	NM	001085495	Arfgef2	8777	NM	146391	Olfr1058	951

NM 007579	Cacna1b	9655	NM 008513	Lrp5	5172	NM 207561	Olfr1040	942
NM 001286418	Rbfox2	6280	NM 029232	Tmco5b	1254	NM 146766	Olfr1109	939
NM 001285937	Zdbf2	12648	NM 031867	Tas1r1	2814	NM 147017	Olfr1045	948
NM 001079822	Tcf7l1	2882	NM 026058	Cers4	3668	NM 146405	Olfr228	942
NM 001042488	Dlgap4	2930	NM 172579	Sipa1l1	7508	NM 146269	Olfr247	945
NM 207010	Mdga2	9813	NM 001198823	App	3377	NM 207133	Olfr741	1144
NM 177407	Camk2a	4966	NM 028025	Mageb16	1854	NM 147060	Olfr667	981
NM 019466	Rcan1	2195	NM 011111	Serpinb2	2007	NM 147078	Olfr1062	956
NM 001112798	Slc8a1	18573	NM 001122733	Kit	5205	NM 146905	Olfr851	939
NM 001042528	Cacna1b	9772	NM 008881	Plxna1	9045	NM 146890	Olfr126	960
NM 032005	Tbx19	2662	NM 178661	Creb3l2	3318	NM 146767	Olfr1104	933
NM 010212	Fhl2	1566	NM 183424	Qrfp	2863	NM 146851	Olfr295	930
NM 001282000	Rbl2	4806	NM 009234	Sox11	8451	NM 146293	Olfr1143	945
NM 001289530	Adarb2	5628	NM 001159595	Ints8	4676	NM 146616	Olfr305	960
NM 019483	Smad9	5380	NM 001164177	Psmd10	1377	NM 146350	Olfr1123	972
NM 009539	Zap70	2247	NM 013917	Pttg1	1698	NM 146478	Olfr891	951
NM 008965	Ptger4	3264	NM 001161798	Mthfr	6085	NM 053237	Vmn1r14	912
NM 001136077	Enpp2	3617	NM 172729	Nod1	4327	NM 146994	Olfr201	927
NM 001110828	Rbfox2	6574	NM 001177845	Tirap	4749	NM 146569	Olfr1014	918
NM 001039079	Prkcz	4111	NM 001113560	Glo1	918	NM 146640	Olfr1153	957
NM 007560	Bmpr1b	5376	NM 178112	Ints8	3434	NM 001038995	Gm4787	2511
NM 001284398	4-Sep	1357	NM 011401	Slc2a3	3973	NM 146577	Olfr1043	945
NM 011323	Scn8a	11241	NM 001029837	Pik3cd	4909	NM 146397	Olfr190	924
NM 001277220	Bmpr1b	5357	NM 172555	Papolg	3776	NM 146298	Olfr746	945
NM 001284392	4-Sep	1426	NM 010881	Ncoa1	7346	NM 146907	Olfr1282	918
NM 001277216	Bmpr1b	5183	NM 001081370	Shank2	5913	NM 001099309	Gm12887	649
NM 011406	Slc8a1	18609	NM 001113734	Mageb16	1725	NM 001085414	Pramef6	1487
NM 011235	Rad51d	7072	NM 001113471	Plin1	1879	NM 001166835	Vmn1r79	921
NM 178365	Pacsin1	4233	NM 007471	App	3152	NM 001166731	Vmn1r77	921
NM 001289766	Zap70	2255	NM 001198824	App	3320	NM 001166735	Vmn1r224	897
NM 001282001	Rbl2	4905	NM 175640	Plin1	1935	NM 001167540	Vmn1r184	945
NM 001286809	Camk2a	4245	NM 001113373	Shank2	6359	NM 001104641	Vmn2r25	2568
NM 021461	Mknk1	2554	NM 011146	Pparg	1769	NM 001166639	Gm4305	910
NM 001277938	Rad51d	7057	NM 181322	Ctcf	3848	NM 001104550	Vmn2r98	2574
NM 001277218	Bmpr1b	5222	NM 028259	Rps6kb1	3283	NM 001104623	Vmn2r12	2496
NM 001289765	Zap70	2290	NM 026364	Prpsap1	1868	NM 001104637	Vmn2r22	2562
NM 001285936	Zdbf2	12705	NM 001001983	Pi4ka	6449	NM 001104541	Vmn2r92	2583
NM 009332	Tcf7l1	2840	NM 001110140	Atp2a2	4565	NM 001011784	Olfr1039	960
NM 177326	Pak2	5741	NM 001174170	Serpinb2	1988	NM 146406	Olfr1076	942
NM 001110829	Rbfox2	6565	NM 080853	Slc17a6	4338	NM 001011735	Olfr1066	942
NM 009188	Sin3b	4135	NM 016883	Psmd10	1456	NM 001105186	Vmn2r73	2556
NM_001277939	Rad51d	1722	NM_001031772	Lin28b	5420	NM_001161855	4933416C03Rik	2254
NM 001277217	Bmpr1b	5209	NM 001136056	Cntfr	2021	NM 001104638	Vmn2r23	2550
NM 001289612	Zap70	1343	NM 011671	Ucp2	4133	NM 009491	Vmn2r10	2544
NM 001285487	Mknk1	2704	NM 001127330	Pparg	1857	NM 001104624	Vmn2r13	2496
NM 011250	Rbl2	4935	NM 001162479	Stra6	2889	NM 001105062	Vmn2r31	2586
NM 008131	Glul	2822	NM 177861	Tmem67	3456	NM 147114	Olfr575	957
NM 001081549	Rcan1	2349	NM 001164268	Kalrn	6430	NM 001104632	Vmn2r19	2565
NM 001110827	Rbfox2	7020	NM 008469	Krt15	1720	NM 001104542	Vmn2r93	2574
NM 001267695	Ctss	1360	NM 008871	Serpine1	3024	NM 207695	Olfr224	945
NM 015744	Enpp2	3461	NM 001167983	Sipa1l1	7717	NM 146537	Olfr311	927
NM 001077499	Scn8a	11340	NM 175364	Lyrm2	1344	NM 001104639	Vmn2r24	2565
NM 001277266	Adam17	4508	NM 001198825	App	3266	NM 001104539	Vmn2r90	2592
NM 001291871	Adam17	4470	NM 173447	Ephb1	4686	NM 001104566	Vmn2r104	2583
NM 009615	Adam17	4451	NM 001291804	Dsc1	4961	NM 146846	Olfr1087	942
NM 001003817	Erbb2	4998	NM 013504	Dsc1	5007	NM 001105055	Vmn2r58	2589
NM 026985	Mcemp1	1132	NM 001291818	Rhbdf1	2836	NM 146899	Olfr1219	936
NM 008306	Ndst1	6088	NM 010117	Rhbdf1	2955	NM 001104567	Vmn2r105	2583
NM 001174079	Smarca4	6364	NM 001114879	Fam208a	7307	NM 001104540	Vmn2r91	2571
NM 001174078	Smarca4	6376	NM 138666	Nlgn1	5630	NM 001105179	Vmn2r51	2559
NM 001033547	Ccdc185	2055	NM 001127685	Rd3l	761	NM 001166636	Gm4312	914

NM 001081020	Adamts6	4822	NM 183088	Zbed5	2492	NM 001104537	Vmn2r83	2595
NM 011417	Smarca4	6367	NM 001163387	Nlgn1	4603	NM 001166641	Gm4307	910
NM 001100415	Gm14430	4538	NM 028945	Fam208a	7581	NM 146903	Olfr871	1147
NM 001081088	Lrp2	15467	NM 134063	Fam208b	8356	NM 001105056	Vmn2r59	2598
NM 080285	Cttnbp2	5931	NM 020259	Hhip	9094	NM 001104634	Vmn2r20	2562
NM 030747	Btnl6	1620	NM 001077404	Nrp2	6724	NM 001102580	Vmn2r76	2562
NM 030746	Btnl4	1761	NM 010939	Nrp2	6688	NM 001104568	Vmn2r106	2589
NM 009624	Adcy9	7840	NM 009660	Alox15	2414	NM 001105188	Vmn2r77	2565
NM 001282945	Adora1	5056	NM 001109749	Cntn4	5262	NM 001166637	Gm4301	914
NM 001291928	Adora1	4588	NM 010148	Epn2	4266	NM 001104572	Vmn2r110	2577
NM 001039510	Adora1	5022	NM 008053	Fxr1	2079	NM 001102581	Vmn2r95	2538
NM 001291910	Adcy9	7632	NM 080437	Celsr3	11558	NM 009490	Vmn2r30	2559
NM 001291930	Adora1	4624	NM 025666	Ubr7	3247	NM 207694	Olfr220	978
NM 001008533	Adora1	5837	NM 001166667	Rtel1	4316	NM 001104626	Vmn2r15	2568
NM 001164171	Myh6	6079	NM 007460	Ap3d1	4730	NM 001005524	Olfr194	921
NM 182939	Ppp4r2	3641	NM 001166668	Rtel1	4208	NM 001104614	Vmn2r3	2670
NM 008367	Il2ra	4428	NM 010290	Gjd2	2907	NM 001104562	Vmn2r100	2553
NM 010467	Hoxd1	1878	NM 001252413	Rpain	1072	NM 147047	Olfr618	957
NM 007459	Ap2a2	4639	NM 013454	Abca1	10260	NM 001104635	Vmn2r21	2562
NM 010855	Myh4	6037	NM 001159536	Adcy3	4668	NM 001105076	Vmn2r46	2430
NM 008084	Gapdh	1444	NM 011412	Slit3	5017	NM 001113468	Vmn2r29	2559
NM_183197	Serpinb9f	1920	NM_001077403	Nrp2	6739	NM_001104569	Vmn2r107	2586
NM 133721	Itga9	7001	NM 001010833	Mdc1	7372	NM 001104591	Vmn2r120	2571
NM 013720	Mga	13931	NM 008809	Pdgfrb	5410	NM 001105057	Vmn2r60	2562
NM_027971	Serpinb12	1687	NM_009425	Tnfsf10	4944	NM_001011837	Olfr748	924
NM 027835	Ifih1	5519	NM 001025439	Camk2d	4299	NM 001105065	Vmn2r33	2586
NM 033652	Lmx1a	3338	NM 016701	Nes	6143	NM 001011810	Olfr485	954
NM 001171053	Mta3	1740	NM 010288	Gja1	3105	NM 001166638	Gm4303	910
NM 001081049	Kmt2a	16439	NM 001033244	Fancd2	4780	NM 001104549	Vmn2r97	2586
NM 057171	Bag6	3858	NM 207239	Gtf3c1	6891	NM 001103368	Vmn2r80	2592
NM 001039545	Myh2	6083	NM 008884	Pml	5240	NM 001011771	Olfr1089	936
NM 001256081	Myo7a	7481	NM 010251	Gabra4	4123	NM 001105058	Vmn2r61	2598
NM 001171054	Mta3	1737	NM 138305	Adcy3	4348	NM 146753	Olfr1195	927
NM 001206382	Mavs	2962	NM 080470	Smc1b	4056	NM 146647	Olfr1154	933
NM 001170537	Mef2c	6427	NM 011169	Prlr	10328	NM 001105151	Vmn2r47	2559
NM 016700	Mapk8	5770	NM 010147	Epn1	2582	NM 146384	Olfr208	918
NM 199007	Sgol2	5314	NM 001077405	Nrp2	6673	NM 001270898	Gm21293	1217
NM 010703	Lef1	3657	NM 001252189	Epn2	4230	NM 001270901	Gm21304	1217
NM 001161456	Cbfb	2776	NM 001146268	Pdgfrb	5413	NM 001270642	Gm21312	1246
NM 011058	Pdgfra	6553	NM 001252458	Prkd2	3340	NM 001167539	Vmn1r238	921
NM 172301	Ccnb1	2316	NM 007719	Ccr7	1975	NM 001005780	-	939
NM 011374	St8sia1	8995	NM 009194	Slc12a2	6520	NM 020015	Magea1	1272
NM 001163622	Prepl	3830	NM 001025438	Camk2d	4286	NM 001167534	Vmn1r2	921
NM 010927	Nos2	3990	NM 207668	Acpp	4488	NM 147028	Olfr1124	957
NM 011952	Mapk3	1772	NM 001102407	Rbm8a	2631	NM 001105063	Vmn2r32	2559
NM 001197322	Foxp1	6649	NM 001252414	Rpain	896	NM 001104619	Vmn2r6	2484
NM 009765	Brca2	10940	NM 021420	Stk4	5189	NM 001105191	Vmn2r52	2559
NM 144888	Mavs	3099	NM 007499	Atm	11964	NM 001085526	Krtap1-3	917
NM 033614	Pde6c	2967	NM 001113189	Fxr1	2171	NM 001277184	Gm14499	860
NM 025282	Mef2c	6325	NM 001077407	Nrp2	4696	NM 008545	Mageb3	2322
NM_001161538	Islr2	4087	NM_001252188	Epn2	4474	NM_001161773	4930544G11Rik	1467
NM 170702	Cd40	1596	NM 178087	Pml	5378	NM 207155	Olfr117	954
NM 001013580	Pard3	3629	NM 001001882	Rtel1	4451	NM 207622	Olfr403	942
NM 134214	Vmn1r214	1104	NM 008873	Plau	2343	NM 001011736	Olfr205	918
NM 008499	Lhx5	2736	NM 176912	C5ar2	3522	NM 146802	Olfr902	1056
NM_031166	Id4	1660	NM_007884	Epyc	1723	NM_146720	Olfr421-ps1	1050
NM 001286343	Bcl11b	7341	NM 023813	Camk2d	4197	NM 207145	Olfr845	942
NM 009602	Chrnb2	5405	NM 029575	Tgfbr2	4728	NM 001167535	Vmn1r3	921

NM 001252469	Bag6	3568	NM 001159537	Adcy3	4727	NM 134198	Vmn1r234	990
NM 008478	L1cam	5288	NM 009115	S100b	1676	NM 001011777	Olfr1042	1097
NM 146254	Wdr78	3793	NM 013806	Abcc2	5389	NM 001025240	Gm5346	2211
NM 001276402	Lef1	3532	NM 007724	Cnga2	3073	NM 134200	Vmn1r237	870
NM 009414	Tph1	4581	NM 011909	Usp18	1778	NM 146822	Olfr640	945
NM 009764	Brca1	6648	NM 007668	Cdk5	2058	NM 053145	Pcdhb20	3418
NM 133752	Opa1	5948	NM 008917	Ppt1	2493	NM 001011854	Olfr605	1011
NM 001289726	Gapdh	1296	NM 008960	Pten	8229	NM 145842	Vmn1r69	1732
NM 009397	Tnfaip3	4437	NM 009371	Tgfbr2	4803	NM 020017	Magea3	1107
NM 170704	Cd40	1678	NM 001166666	Rtel1	4334	NM 134229	Vmn1r67	999
NM 001206383	Mavs	2696	NM 008261	Hnf4a	4371	NM 147100	Olfr613	5137
NM 011249	Rbl1	4909	NM 175260	Myh10	7783	NM 146594	Olfr1100	966
NM 007667	Cdh8	4786	NM 008078	Gad2	5625	NM 177256	Prdx6b	2889
NM 001099635	Myh3	5992	NM 001113188	Fxr1	2339	NM 146883	Olfr876	1048
NM 011623	Top2a	5217	NM 021282	Cyp2e1	1759	NM 207632	Olfr1118	996
NM 009688	Xiap	6550	NM 008610	Mmp2	3070	NM 147120	Olfr638	966
NM 053202	Foxp1	7177	NM 019449	Unc93b1	2277	NM 146865	Olfr992	930
NM 023279	Tubb3	1758	NM 001166665	Rtel1	4433	NM 001270685	Gm21637	778
NM 001171052	Mta3	2673	NM 001252415	Rpain	1119	NM 146752	Olfr1106	939
NM 146095	Rorb	8755	NM 001146005	C5ar2	3429	NM 146282	Olfr846	942
NM 011281	Rorc	2503	NM 011749	Zfp148	9431	NM 010293	Gykl1	1915
NM 001199177	Opa1	6002	NM 025875	Rbm8a	2628	NM 146872	Olfr908	933
NM 008092	Gata4	3393	NM 009569	Zfpm1	3393	NM 146416	Olfr290	948
NM 022309	Cbfb	2893	NM 001077406	Nrp2	4711	NM 146370	Olfr47	966
NM 013613	Nr4a2	3172	NM 013464	Ahr	5494	NM 146885	Olfr1294	939
NM 010025	Dcx	8982	NM 008553	Ascl1	2259	NM 001011791	Olfr193	930
NM 001043354	Rorb	9289	NM 009915	Ccr2	3589	NM 146536	Olfr313	1074
NM_001077264	Ap2a1	3391	NM_178900	Prkd2	3625	NM_001205242	A430107P09Rik	2238
NM 001161458	Cbfb	2924	NM 018768	Stx8	4123	NM 134192	Vmn1r228	1443
NM 001252192	Eya1	4238	NM 001161428	Unc93b1	2003	NM 207618	V1rd18	1157
NM 001256082	Myo7a	7214	NM 010121	Eif2ak3	4512	NM 146486	Olfr203	921
NM 001164274	Mga	13304	NM 054090	Olfr73	942	NM 001204913	Gm20172	1551
NM_009071	Rock1	6187	NM_001271431	Cd82	1625	NM_146654	Olfr237-ps1	933
NM 001013581	Pard3	3233	NM 133828	Creb1	8389	NM 146908	Olfr1280	918
NM 009718	Neurog2	2244	NM 001271462	Cd82	1724	NM 147066	Olfr1389	1073
NM 016780	Itgb3	5795	NM 001080926	Lrp8	7296	NM 146459	Olfr1215	943
NM 016961	Mapk9	4682	NM 001038698	Elavl4	4008	NM 001005230	Olfr1024	984
NM 001167920	Slc8a3	4943	NM 177262	Pkn1	3436	NM 146270	Olfr370	1096
NM 001289921	Rorb	8808	NM 172524	Nipal4	3295	NM 053143	Pcdhb18	5039
NM 008002	Fgf10	4572	NM 001252579	Sulf2	3794	NM 001177513	Gm7102	1624
NM 001277230	Zbtb24	2554	NM 010696	Lcp2	3348	NM 146693	Olfr1462	924
NM 144783	Wt1	3092	NM 011032	P4hb	2538	NM 146442	Olfr934	933
NM 008663	Myo7a	7361	NM 008869	Pla2g4a	2846	NM 146699	Olfr1445	945
NM_009828	Ccna2	2827	NM_001164051	Pik3cd	4942	NM_029229	4930557A04Rik	550
NM 001252468	Bag6	3804	NM 001163530	Prl	892	NM 146539	Olfr373	945
NM 009376	Ift88	3097	NM 010275	Gdnf	3509	NM 146901	Olfr1217	1105
NM 001166402	Tnfaip3	4352	NM 001099298	Scn2a1	8690	NM 146763	Olfr1406	1063
NM 007561	Bmpr2	11094	NM 001205313	Stat1	5150	NM 053136	Pcdhb11	3615
NM 001081306	Ptprz1	8068	NM 145963	Kcnj14	2639	NM 001011721	Olfr102	1027
NM 133985	Oxsr1	4647	NM 009452	Tnfsf4	1609	NM 147062	Olfr124	1122
NM 053069	Atg5	2352	NM 133365	Dnah5	15616	NM 145848	Vmn1r71	2037
NM 001083316	Pdgfra	6570	NM 001271538	Myh14	6515	NM 146986	Olfr38	954
NM 001081001	Brca2	11134	NM 001034115	Shank1	7596	NM 146991	Olfr206	921
NM 010546	Ikbkb	4540	NM 009667	Ampd3	4098	NM 206816	Olfr128	927
NM 010856	Myh6	6015	NM 023635	Rab27a	2971	NM 146487	Olfr130	954
NM 172692	Gba2	3552	NM 028673	Zdbf2	12621	NM 207550	Olfr199	927
NM 001081432	Ptprq	7058	NM 023566	Muc2	7368	NM 146875	Olfr895	951
NM 008192	Gucy2e	8331	NM 029210	Sv2c	4252	NM 001166634	Gm4302	927
NM 207692	Mapk9	4677	NM 001013833	Prkg1	6992	NM 146488	Olfr137	939

NM_054082	Mta3	2115	NM_172269	Vps18	4054	NM_027634	4931431F19Rik	1911
NM 001139509	Nr4a2	3026	NM 001142337	Lmo2	1529	NM 001011819	Olfr312	927
NM 001256083	Myo7a	7196	NM 001195539	Dclk1	7004	NM 147118	Olfr635	966
NM 008584	Meox2	2348	NM 001036684	Atp2b2	6922	NM 001024135	Kbtbd7	4529
NM 001161536	Islr2	4319	NM 019827	Gsk3b	8298	NM 146408	Olfr1065	992
NM 011537	Tbx5	3941	NM 177357	Kalrn	15439	NM 146558	Olfr866	1077
NM 008540	Smad4	3361	NM 001001490	Oxgr1	3732	NM 146525	Olfr847	939
NM 177369	Myh8	6149	NM 009925	Col10a1	3139	NM 146918	Olfr1180	1063
NM_001161457	Cbfb	2797	NM_001253860	Scn5a	8287	NM_025634	2310042E22Rik	1286
NM 023794	Etv5	3847	NM 011198	Ptgs2	4150	NM 146273	Olfr448	933
NM 021284	Kras	4670	NM 033073	Krt7	1602	NM 147050	Olfr659	969
NM 139294	Braf	9728	NM 013703	Vldlr	8327	NM 146593	Olfr1111	940
NM 177129	Cntn2	9535	NM 178719	Mief1	5099	NM 146582	Olfr1046	951
NM 011611	Cd40	1683	NM 001142335	Lmo2	1519	NM 207557	Olfr681	948
NM 001170959	Pde6c	2892	NM 146055	Ccar2	3705	NM 001011532	Olfr1037	1125
NM 028136	Dhx36	4975	NM 001078167	Srsf1	5560	NM 146490	Olfr1411	972
NM 001079883	Bcl11b	7923	NM 001252635	Cdh23	11090	NM 146303	Olfr1449	945
NM 021399	Bcl11b	7707	NM 001199247	Kat5	1872	NM 146620	Olfr292	1077
NM 011723	Xdh	4623	NM 016694	Park2	3202	NM 001105557	Zfp938	2031
NM 001161539	Islr2	4049	NM 009871	Cdk5r1	4166	NM 147035	Olfr711	1359
NM 001110224	Dcx	8997	NM 178029	Setd1a	5934	NM 146559	Olfr868	1132
NM_001206385	Mavs	2871	NM_001252578	Sulf2	3948	NM_001159775	Olfr391-ps	1120
NM 033620	Pard3	5830	NM 021544	Scn5a	8455	NM 134201	Vmn1r236	1121
NM 001136084	Tph1	4270	NM 144859	Pja2	4520	NM 001127725	Sec14l5	2337
NM 007458	Ap2a1	3457	NM 054039	Foxp3	3765	NM 146578	Olfr1033	3744
NM_001199213	Serpinb12	1619	NM_001168655	Socs2	2195	NM_001033530	AW146154	3411
NM 009693	Apob	13931	NM 172898	Kirrel2	3122	NM 146698	Olfr1443	4361
NM 013722	Syn3	8797	NM 016673	Cntfr	2000	NM 001104579	Vmn2r115	2574
NM 001039154	Cdh8	3822	NM 001037726	Crebl	8267	NM 001104575	Vmn2r112	2574
NM 011448	Sox9	4146	NM 001198566	Sulf1	4544	NM 146415	Olfr291	1159
NM 001163672	Mapk9	4682	NM 010568	Insr	9357	NM 001164284	C87414	2719
NM 170703	Cd40	1621	NM 001199136	Macf1	23495	NM 001164285	C87414	2572
NM 030679	Myh1	6058	NM 011163	Eif2ak2	4343	NM 201643	Ugt1a5	3208
NM 001139516	Rbl1	2400	NM 010173	Faah	3816	NM 146809	Olfr1426	1727
NM 008937	Prox1	4148	NM 201242	Lims1	4351	NM 146923	Olfr20	1062
NM 011159	Prkdc	12674	NM 015748	Slit1	5300	NM 009485	Vmn2r123	3045
NM 001163671	Mapk9	4677	NM 138301	Trpm2	7292	NM 001033767	Gm4951	2648
NM 001110222	Dcx	9078	NM 009652	Akt1	2707	NM 147042	Olfr1353	1266
NM 001013367	Prkaa1	4655	NM 009723	Atp2b2	7111	NM 146696	Olfr1477	1100
NM 001164477	Ifih1	5372	NM 007706	Socs2	2222	NM 146922	Olfr376	1079
NM 001113514	Itga9	5180	NM 021297	Tlr4	3847	NM 001104581	Vmn2r117	2574
NM 010164	Eya1	4354	NM 001177595	Slc8b1	2725	NM 147009	Olfr389	1395
NM 001177867	Sgol2	4337	NM 008493	Lep	3257	NM 001013823	Krtap4-16	972
NM 033217	Ngfr	3409	NM 001161413	Slc3a2	2813	NM 053126	Pcdhb1	2457
NM 080440	Slc8a3	4964	NM 001113559	Sox5	7187	NM 147006	Olfr392	1069
NM 029216	Chd5	9375	NM 001177976	Irak1	2478	NM 001172686	Olfr376	1210
NM 001164495	Syn3	4020	NM 008417	Kcna2	11582	NM 146898	Olfr1213	1297
NM 001122850	Pard3	4178	NM 001081414	Grm5	8428	NM 146697	Olfr1442	1065
NM 016769	Smad3	5090	NM 145099	Trpv3	2440	NM 183167	AI987944	2664
NM 001110223	Dcx	9000	NM 007429	Agtr2	2872	NM 001004182	Tmem178b	3092
NM 145526	P2rx3	4190	NM 001177974	Irak1	2574	NM 001102584	Vmn2r114	2571
NM 001081376	Chd5	9486	NM 008026	Fli1	3087	NM 001037922	Cks1brt	2397
NM 011143	Pou4f1	3801	NM 010071	Dok2	1717	NM 053172	Mepe	1682
NM 001197321	Foxp1	7042	NM 001243163	Sox5	7079	NM 146326	Olfr943	1079
NM 010806	Mllt4	7401	NM 013645	Pvalb	904	NM 001104573	Vmn2r111	2574
NM 053247	Lyve1	2607	NM 001177594	Slc8b1	2823	NM 001104578	Vmn2r113	2571
NM 001007567	Slc7a6os	1525	NM 001025432	Crebbp	7507	NM 198669	Prb1	1635
NM 001161537	Islr2	4310	NM 001111316	Ptprc	5568	NM 001199330	AI987944	2948

NM_207243	Muc19	22806	NM_010840	Mthfr	6008	NM_001085511	4932429P05Rik	3093
NM 011426	Siglec1	6427	NM 170779	Wwc1	3315	NM 147071	Olfr1352	1174
NM 001163493	Stard13	5638	NM 010408	Hcn1	7911	NM 175349	Ldhal6b	1463
NM 146258	Stard13	5581	NM 001199137	Macf1	17414	NM 001163836	Pabpc6	3208
NM 001005248	Hps5	4804	NM 025427	Rgcc	934	NM 001160386	Dnah7b	12318
NM 001290640	Dab2ip	6380	NM 001198565	Sulf1	4639	NM 030742	Vmn1r63	2172
NM 001290994	Usp29	7540	NM 013912	Apln	3148	NM 020513	Olfr1508	1865
NM 170689	Ank3	9971	NM 001161420	Vldlr	8243	NM 001085540	Pramef17	1745
NM 009986	Cux1	12912	NM 008173	Nr3c1	6345	NM 001126317	Gm1140	782
NM 139065	Rbm47	4705	NM 001164052	Pik3cd	4636	NM 001085524	Gm5634	842
NM 144822	Micu1	2358	NM 001252488	Catsper3	1199	NM 009489	Vmn2r37	3102
NM 007778	Csf1	4192	NM 001163743	Nlrx1	3706	NM 001085525	Gm14511	842
NM 001113569	Stxbp1	4005	NM 021893	Cd274	3653	NM 001100616	Vmn2r121	3062
NM_170690	Ank3	9962	NM_172450	4930539E08Rik	4594	NM_001001319	Pramel4	2399
NM 007601	Capn3	3167	NM 008577	Slc3a2	2572	NM 027568	Krtap31-1	971
NM 001291035	Mapk7	3265	NM 001146200	Pik3cg	6634	NM 134170	Vmn1r32	2442
NM 011602	Tln1	8560	NM 001271540	Myh14	6457	NM 001033541	Gm5127	1809
NM 001290710	Ebf1	4999	NM 001163399	Elavl4	4052	NM 001033440	Gm1587	1354
NM 001109761	Capn3	2624	NM 172294	Sulf1	4623	NM 177703	Fbxw19	1462
NM 026162	Plxdc2	6963	NM 026810	Mlh1	2598	NM 172844	Fmo9	3065
NM 001291226	Rbm47	4940	NM 007746	Map3k8	2507	NM 177843	Gm14461	1780
NM 001290486	Lpar1	3247	NM 001177667	Sptan1	8039	NM 001270899	Gm6763	1217
NM 001256001	Cacna1c	13643	NM 007405	Adcy6	6038	NM 029106	Spin2-ps1	778
NM 001281966	Itk	4283	NM 028643	Micu2	2310	NM 009487	Vmn2r89	2962
NM 009204	Slc2a4	2822	NM 001163397	Elavl4	3763	NM 001177586	Esp31	1311
NM 001290758	Tlr7	3875	NM 001171007	Nod1	4076	NM 001177587	Esp36	1117
NM 178446	Rbm47	5147	NM 010635	Klf1	1534	NM 001244651	Esp15	1176
NM 001168277	Jazf1	2926	NM 001276301	Ampd3	3979	NM 001244763	Esp18	1257
NM 178220	Arrb1	7088	NM 001142336	Lmo2	1605	NM 001013751	Syna	2918
NM 001290709	Ebf1	5206	NM 011444	Sox5	7331	NM 001177652	Gm5886	647
NM 146194	Picalm	4174	NM 010431	Hif1a	4775	NM 177359	Zfp799	6025
NM 001291442	Micu1	2376	NM 001177975	Irak1	3835	NM 001293301	Clasp1	7651
NM_009399	Tnfrsf11a	5022	NM_001168296	Ephb1	4563	NM_001293300	Clasp1	7951
NM 001252523	Picalm	4009	NM 001161791	Mefv	3094	NM 178071	Nme7	2385
NM 013822	Jag1	5493	NM 008914	Ppp3cb	3881	NM 001293313	Nme7	1737
NM 001252524	Picalm	3985	NM 001168656	Socs2	2153	NM 138314	Nme7	1740
NM 173406	Jazf1	2925	NM 146119	Fam129b	3693			
NM 001291433	Dido1	7311	NM 008987	Ptx3	1924			
NM 008904	Ppargc1a	6464	NM 028021	Myh14	6392			
NM 001290756	Tlr7	3862	NM 013598	Kitl	5450			
NM 183308	Pon2	1850	NM 001276400	Rad21l	1659			
NM 033269	Chrm3	3168	NM 011857	Tenm3	10978			
NM 001290639	Dab2ip	6075	NM 001271432	Cd82	1920			
NM 010889	Neb	22489	NM 008143	Gnb2l1	1254			
NM 001113529	Csf1	3307	NM 001252651	Csf3r	3583			
NM 001255997	Cacna1c	13340	NM 173788	Npr2	3660			
NM 001291012	Nphp1	2300	NM 010119	Ehd1	3182			
NM 001159535	Cacna1c	13586	NM 011103	Prkcd	2790			
NM 146005	Ank3	10025	NM 010870	Naip5	5368			
NM 010789	Meis1	3346	NM 001168657	Socs2	2074			
NM 001281968	Itk	4271	NM 145979	Chd4	6438			
NM 001281965	Itk	4292	NM 023370	Cdh23	11096			
NM 009781	Cacna1c	13340	NM 001168253	Fam83h	4545			
NM 001114124	Dab2ip	6540	NM 010586	Itpr2	11740			
NM 008514	Lrp6	9368	NM 011160	Prkg1	6915			
NM 213659	Stat3	4487	NM 008371	Il7	2475			
NM 020009	Mtor	8612	NM 009045	Rela	2709			
NM 001271599	Sort1	6950	NM 133249	Ppargc1b	3656			
NM 008786	Pcmt1	1917	NM 007552	Bmi1	3594			

NM 007901	S1pr1	3029	NM 009320	Slc6a6	6169
NM 170728	Ank3	9320	NM 020272	Pik3cg	6811
NM 008413	Jak2	5055	NM 001199249	Kat5	1423
NM 009598	Ace	3129	NM 001113418	Ppara	7216
NM 001252522	Picalm	4024	NM 001253781	Prlr	3916
NM 133990	Il13ra1	3686	NM 145907	Porcn	1878
NM 001290424	Fmr1	4351	NM 001199248	Kat5	1971
NM 001290335	Cacna1c	12656	NM 007656	Cd82	1833
NM 177231	Arrb1	7112	NM 001193303	Lims1	4473
NM 001113530	Csf1	2324	NM 001025309	Pja2	4706
NM 001291443	Micu1	2370	NM 009502	Vcl	5229
NM 010485	Elavl1	6030	NM 011333	Ccl2	806
NM 007548	Prdm1	5143	NM 001040398	Setd1b	8868
NM 021323	Usp29	7476	NM 009504	Vdr	4354
NM 011486	Stat3	4437	NM 010513	Igf1r	11978
NM 009295	Stxbp1	3874	NM 001271430	Cd82	1757
NM 009007	Rac1	2284	NM 019923	Itpr2	11839
NM 008714	Notch1	9497	NM 011034	Prdx1	1417
NM 133211	Tlr7	3794	NM 028072	Sulf2	3790
NM 001281819	Ace	5091	NM 172856	Cers6	3930
NM 170729	Ank3	9908	NM 001205314	Stat1	5168
NM 009670	Ank3	6966	NM 001177567	Otogl	6984
NM 001159533	Cacna1c	13592	NM 008653	Mybpc3	4163
NM 170687	Ank3	7554	NM 001177973	Irak1	3955
NM 007897	Ebf1	5203	NM 010175	Fadd	2871
NM 001048177	Jak2	4972	NM 001267872	Zdbf2	12571
NM 021782	Il21	3090	NM 178420	Nlrx1	3694
NM 013646	Rora	10878	NM 203491	Chrm2	2036
NM 011123	Plp1	3473	NM 001076554	Sptan1	8042
NM 011518	Syk	5148	NM 133872	Kdm1a	3030
NM 001164663	Soga1	13232	NM 001146201	Pik3cg	6690
NM 001198977	Syk	5144	NM 008100	Gcg	1091
NM 139144	Ogt	5415	NM 009778	C3	5147
NM 001289916	Rora	10869	NM 001252487	Catsper3	1352
NM 001127382	Rbm47	4947	NM 009428	Trpc5	4158
NM 001290755	Tlr7	3943	NM 177909	Slc9a9	3492
NM 020567	Gmnn	995	NM 008859	Prkcq	3313
NM 001290757	Tlr7	3830	NM 008575	Mdm4	3490
NM 213660	Stat3	4484	NM 026148	Lims1	4327
NM 145840	Rgs9bp	6604	NM 133221	Slc8b1	2893
NM 001040400	Tet2	9199	NM 001163742	Nlrx1	3605
NM 001290637	Dab2ip	4152	NM 021311	Piwil1	3944
NM 001193271	Meis1	3441	NM 011210	Ptprc	5249
NM 008031	Fmr1	4426	NM 001145937	Tenm3	10958
NM 175551	Dido1	8476	NM 010488	Elavl4	4094
NM 010414	Htt	13237	NM 139301	Catsper1	2061
NM 001005247	Hps5	4828	NM 001081111	Tmf1	6781
NM 001177799	Capn3	2891	NM 001143834	Grm5	8524
NM 016902	Nphp1	2303	NM 172816	Slc30a8	1906
NM 028898	Rptor	6956	NM 020013	Fgf21	947
NM 133212	Tlr8	3158	NM 001160353	Grm2	3338
NM 001113331	Shc1	3569	NM 001167905	Cyp2c44	1596
NM 019972	Sort1	6851	NM 173374	Srsf1	5364
NM 001159520	Pax3	3376	NM 181420	Fn3krp	2028
NM 010104	Edn1	2344	NM 001136055	Cd82	1701
NM 008808	Pdgfa	1019	NM 001177668	Sptan1	7979
NM 001159534	Cacna1c	13592	NM 008840	Pik3cd	5102
NM 008512	Lrp1	14907	NM 009952	Creb1	8431
NM 001077514	Slc1a2	11571	NM 016913	Porcn	1863
NM 001291037	Mapk7	3264	NM 010934	Npy1r	3007
NM 010508	Ifnar1	7025	NM 023638	Porcn	1896
NM 007434	Akt2	2957	NM 001165894	Akt1	2657

NM 170730	Ank3	6656	NM 001199348	Foxp3	3690
NM 001289917	Rora	1422	NM 027011	Krt5	2190
NM_001167864	Hps5	4729	NM_028158	1700021F07Rik	641
NM 001291041	Il21	3178	NM 145908	Porcn	1881
NM 016678	Reck	4450	NM 001001446	Cyp2c44	1964
NM 001290535	Ogt	5218	NM 011164	Prl	895
NM 011841	Mapk7	2999	NM 001164050	Pik3cd	4915
NM 001290269	Rgs21	827	NM 001271461	Cd82	1712
NM 001077515	Slc1a2	10998	NM 011896	Spry1	2490
NM 008781	Pax3	3868	NM 001195538	Dclk1	7817
NM 145833	Lin28a	3480	NM 177730	Impad1	4636
NM 001252520	Picalm	4159	NM 001199347	Foxp3	3832
NM 001291013	Nphp1	2297	NM 019453	Mefv	3126
NM 207624	Ace	4906	NM 145522	Rabepk	1858
NM 183355	Pbx1	7163	NM 001162476	Stra6	3095
NM 016981	Slc9a1	4606	NM 024245	Kif23	3439
NM 001291234	Cux1	13146	NM 025802	Pnpla2	2467
NM 170688	Ank3	9437	NM 001163689	Pnpla2	2635
NM 001252521	Picalm	4150	NM 024196	Tbc1d20	1936
NM 001290641	Dab2ip	6673	NM 007782	Csf3r	3907
NM 001255998	Cacna1c	13415	NM 138667	Tab2	4278
NM 010583	Itk	4274	NM 008505	Lmo2	1646
NM 001290561	Plp1	3368	NM 054081	Mta1	2775
NM 013735	Trp53bp1	9652	NM 134087	Fam83h	4513
NM 001291509	Pbx1	6890	NM 019439	Gabbr1	4486
NM 001256002	Cacna1c	13841	NM 009283	Stat1	5183
NM 001255999	Cacna1c	13340	NM 178637	Kat5	2028
NM 001291036	Mapk7	3114	NM 001164049	Pik3cd	4921
NM 009744	Bcl6	3326	NM 133360	Acaca	9054
NM 021451	Pmaip1	2654	NM 009291	Stra6	3054
NM 008783	Pbx1	3889	NM 001268286	Ptprc	5177
NM 019771	Dstn	1918	NM 176979	Topbp1	5112
NM 001290711	Ebf1	5322	NM 029772	Catsper3	1313
NM 001291033	Mapk7	3049	NM 011144	Ppara	7454
NM 177852	Dido1	7259	NM 033374	Dock2	6431
NM 001291034	Mapk7	3124	NM 001277293	Grip1	3959
NM 001291508	Pbx1	7050	NM 001277294	Grip1	5018
NM 001256000	Cacna1c	13634	NM 019695	Pard6a	1284
NM 001290830	Trp53bp1	9502	NM 052977	Adarb2	5633
NM 011368	Shc1	3171	NM 001109897	Trpc2	3342
NM 001110208	Akt2	3199	NM 013599	Mmp9	3174
NM 001291233	Cux1	13507	NM 001286684	Slc8a1	15847
NM 019930	Ranbp9	3064	NM 007715	Clock	9801
NM 001290562	Plp1	3399	NM 001047435	Pard6a	1281
NM_001160129	LOC100040786	1179	NM_001289744	Lrrc4c	3440
NM 001104543	Vmn2r94	2529	NM 011223	Pxn	3722
NM_001100416	OTTMUSG00000016609	2592	NM_013605	Muc1	2243
NM 001270510	Gm10256	1580	NM 001080927	Rbpj	5508
NM 001205282	Gm14496	2550	NM 001289826	Clock	9758
NM 001105071	Vmn2r39	2586	NM 007579	Cacna1b	9655
NM 001105178	Vmn2r50	2586	NM 001285937	Zdbf2	12648
NM 001105072	Vmn2r40	2559	NM 001289842	Palb2	3347
NM 028970	Rbm31y	1958	NM 001277116	Rbpj	5590
NM 001105073	Vmn2r41	2559	NM 207010	Mdga2	9813
NM 001104551	Vmn2r99	2571	NM 007807	Cybb	4750
NM 134190	Vmn1r229	921	NM 001277295	Grip1	3758
NM 146831	Olfr133	939	NM 001285870	Trim3	2892
NM 146832	Olfr134	939	NM 133442	Grip1	5080

NM 134197	Vmn1r230	951	NM 177259	Dab1	5344
NM 146332	Olfr135	939	NM 001112798	Slc8a1	18573
NM 134212	Vmn1r200	939	NM 001042528	Cacna1b	9772
NM 001011757	Olfr663	1035	NM 001282000	Rbl2	4806
NM 147110	Olfr570	939	NM 001277149	Chd7	11306
NM 146604	Olfr716	945	NM 001289530	Adarb2	5628
NM 134231	Vmn1r185	960	NM 009035	Rbpj	5410
NM 134239	Vmn1r217	897	NM 001285873	Trim3	2529
NM 001011787	Olfr1307	939	NM 001288578	Adh5	1586
NM 146631	Olfr120	993	NM 028736	Grip1	5219
NM 146632	Olfr116	966	NM 009539	Zap70	2247
NM 146377	Olfr127	972	NM 001285871	Trim3	3077
NM 146379	Olfr654	1026	NM 001047436	Pard6a	1297
NM 001011809	Olfr728	936	NM 018880	Trim3	2866
NM 001011811	Olfr487	945	NM 001080928	Rbpj	5394
NM 146398	Olfr1325	948	NM 007410	Adh5	1604
NM 146913	Olfr1348	939	NM 011323	Scn8a	11241
NM 001011829	Olfr761	972	NM 001287057	Vegfa	3475
NM 146423	Olfr887	930	NM 001289742	Lrrc4c	3860
NM 146424	Olfr888	945	NM 018781	Egr3	3872
NM 001011840	Olfr1463	933	NM 011406	Slc8a1	18609
NM 146692	Olfr1454	924	NM 001277292	Grip1	5161
NM 146701	Olfr1448	945	NM 011235	Rad51d	7072
NM 146703	Olfr1447	930	NM 001286018	Klrk1	3197
NM 146705	Olfr1451	933	NM 130891	Grip1	4937
NM 001011866	Olfr309	927	NM 001289766	Zap70	2255
NM 146981	Olfr1260	933	NM 001282001	Rbl2	4905
NM 146482	Olfr889	930	NM 001287058	Vegfa	3343
NM 146777	Olfr818	957	NM 019745	Pdcd10	1923
NM 134166	Vmn1r36	918	NM 022410	Myh9	7439
NM 134168	Vmn1r38	909	NM 001289925	Egr3	3925
NM 134169	Vmn1r33	918	NM 001277938	Rad51d	7057
NM 145844	Vmn1r189	939	NM 001286345	Pard6a	1194
NM 134172	Vmn1r26	1020	NM 001289765	Zap70	2290
NM 146814	Olfr665	951	NM 001285936	Zdbf2	12705
NM 134180	Vmn1r28	909	NM 177382	Cyp2r1	1630
NM 146391	Olfr1058	951	NM 001083322	Klrk1	3179
NM 134195	Vmn1r227	1026	NM 001081238	Palb2	3750
NM 207135	Olfr1084	954	NM 009188	Sin3b	4135
NM 146819	Olfr466	927	NM 001289612	Zap70	1343
NM 001011803	Olfr1306	939	NM 133915	Pxn	3824
NM 146890	Olfr126	960	NM 011250	Rbl2	4935
NM 146853	Olfr1341	939	NM 001289927	Egr3	3799
NM 146577	Olfr1043	945	NM 008131	Glul	2822
NM 001013773	Nxpe5	2312	NM 178725	Lrrc4c	3492
NM 053137	Pcdhb12	3034	NM 001286344	Pard6a	1266
NM 001101450	Gm44	993	NM 001289743	Lrrc4c	3644
NM 013621	Olfr69	1357	NM 033078	Klrk1	3272
NM 001142690	Dmrtc1c2	1611	NM 001038701	Gabrb3	5516
NM 001166720	Vmn1r39	918	NM 001077499	Scn8a	11340
NM 175126	Zcchc3	3089	NM 001003817	Erbb2	4998
NM 001167540	Vmn1r184	945	NM 001291894	Lilrb4	1502
NM 026351	Ttc39d	2027	NM 001033217	Prickle1	4085
NM 001033878	Vmn2r66	2556	NM 013532	Lilrb4	1497
NM 001104550	Vmn2r98	2574	NM 001174079	Smarca4	6364
NM 001200055	AU018829	2901	NM 029939	Ccdc151	2218
NM 001104541	Vmn2r92	2583	NM 001291891	Adam19	6421
NM 146406	Olfr1076	942	NM 001174078	Smarca4	6376
NM 001103365	Vmn2r86	2547	NM 001081020	Adamts6	4822
NM 001011735	Olfr1066	942	NM 001145100	Btbd18	3520
NM_001104565	Vmn2r103	2571	NM_001162906	2410089E03Rik	10551

NM 001105186	Vmn2r73	2556	NM 001291893	Gp49a	1197
NM 001105152	Vmn2r48	2568	NM 001163787	Ccdc151	2221
NM 009491	Vmn2r10	2544	NM 011417	Smarca4	6367
NM 147114	Olfr575	957	NM 001291892	Gp49a	1487
NM 001011525	Olfr1415	936	NM 001291890	Adam19	6226
NM 001104542	Vmn2r93	2574	NM 009616	Adam19	6411
NM 001103366	Vmn2r87	2547	NM 001013393	Gm11487	1323
NM 001102579	Vmn2r67	2556	NM 001100415	Gm14430	4538
NM 001104539	Vmn2r90	2592	NM 013539	Spsb2	1179
NM 001104566	Vmn2r104	2583	NM 008147	Gp49a	1482
NM 001102578	Vmn2r75	2562	NM 080285	Cttnbp2	5931
NM 001105182	Vmn2r69	2553	NM 001033385	Tbc1d32	7269
NM 001104540	Vmn2r91	2571	NM 008882	Plxna2	11049
NM 001104537	Vmn2r83	2595	NM 009623	Adcy8	5064
NM 001102602	Vmn2r85	2553	NM 001291903	Adcy8	4974
NM 001105183	Vmn2r70	2565	NM 009624	Adcy9	7840
NM 001104572	Vmn2r110	2577	NM 001291910	Adcy9	7632
NM 001105156	Vmn2r49	2559	NM 001111075	Cdc25b	3028
NM 001102581	Vmn2r95	2538	NM 001164171	Myh6	6079
NM 001104614	Vmn2r3	2670	NM 001253718	Atg7	3895
NM 001104562	Vmn2r100	2553	NM 008367	Il2ra	4428
NM 001105184	Vmn2r71	2568	NM 001163430	Sirt6	1668
NM_134211	Vmn1r-ps103	972	NM_010855	Myh4	6037
NM 001104569	Vmn2r107	2586	NM 001204202	Spp1	1480
NM 001104627	Vmn2r16	2553	NM 017372	Lyz2	1057
NM 001166210	Myh15	6340	NM 013720	Mga	13931
NM_001104591	Vmn2r120	2571	NM_027971	Serpinb12	1687
NM 001105057	Vmn2r60	2562	NM 033652	Lmx1a	3338
NM 001104549	Vmn2r97	2586	NM 016885	Emcn	1437
NM 001104621	Vmn2r9	2547	NM 001081049	Kmt2a	16439
NM 001104564	Vmn2r102	2574	NM 009386	Tjp1	7054
NM 001105068	Vmn2r36	2559	NM 009848	Entpd1	4197
NM 207280	Ccdc121	1835	NM 011382	Six4	5572
NM 007448	Ang5	708	NM 001206382	Mavs	2962
NM 001101623	Gm20826	758	NM 194054	Rtn4	6165
NM 001160135	Gm20806	999	NM 199007	Sgol2	5314
NM 001199331	Gm20822	687	NM 010703	Lef1	3657
NM 001105063	Vmn2r32	2559	NM 011701	Vim	1834
NM 001104619	Vmn2r6	2484	NM 001110350	Sin3a	5130
NM 001105181	Vmn2r68	2556	NM 011380	Six2	2108
NM 001105185	Vmn2r72	2571	NM 008737	Nrp1	5921
NM 001167542	Vmn1r221	939	NM 175678	Npsr1	3791
NM_001166709	Vmn1r207-ps	939	NM_011058	Pdgfra	6553
NM 001081476	Gm14725	828	NM 011261	Reln	11702
NM 001099330	Scgb1b30	491	NM 001163622	Prepl	3830
NM 181274	Zfp869	3262	NM 010927	Nos2	3990
NM_001161773	4930544G11Rik	1467	NM_011952	Mapk3	1772
NM 134191	Vmn1r226	897	NM 001197322	Foxp1	6649
NM 146802	Olfr902	1056	NM 010891	2-Sep	3223
NM 001025240	Gm5346	2211	NM 009864	Cdh1	4413
NM 053144	Pcdhb19	4714	NM 019721	Mettl3	2035
NM 146329	Olfr642	945	NM 144888	Mavs	3099
NM 174885	Adam6a	2507	NM 033614	Pde6c	2967
NM 146854	Olfr982	966	NM 170702	Cd40	1596
NM 147120	Olfr638	966	NM 001013580	Pard3	3629
NM 146867	Olfr131	945	NM 009019	Rag1	6669
NM 198617	Tspyl3	3087	NM 001109992	Ptpn11	5616
NM 010293	Gykl1	1915	NM 001159719	2-Sep	3267

NM 146808	Olfr1240	1074	NM 008478	L1cam	5288
NM 146430	Olfr742	1120	NM 010195	Lgr5	4711
NM_001270700	4930402K13Rik	1781	NM_001271584	Dnajc5	4378
NM 147038	Olfr1416	939	NM 001276402	Lef1	3532
NM 001009545	Adam6b	2271	NM 172512	Gabpb2	8606
NM 146699	Olfr1445	945	NM 009606	Acta1	1481
NM 134230	Vmn1r66	1524	NM 144801	Tmem143	2184
NM 053136	Pcdhb11	3615	NM 009764	Brca1	6648
NM 146467	Olfr1388	936	NM 133752	Opa1	5948
NM 001011753	Olfr115	995	NM 010277	Gfap	2733
NM 001005481	Olfr132	942	NM 009397	Tnfaip3	4437
NM 147037	Olfr1413	1082	NM 024226	Rtn4	3271
NM 147062	Olfr124	1122	NM 170704	Cd40	1678
NM 001160136	Gm20917	1004	NM 001206383	Mavs	2696
NM 145848	Vmn1r71	2037	NM 007418	Adra2c	3214
NM 001011830	Olfr119	1033	NM 011202	Ptpn11	5628
NM 001199332	Gm20877	1212	NM 001110496	Tmem87a	2924
NM 146360	Olfr574	1041	NM 007667	Cdh8	4786
NM 206816	Olfr128	927	NM 001081028	Sipa1l3	7713
NM 213721	Olfr118	966	NM 001099635	Myh3	5992
NM 146748	Olfr661	960	NM 011623	Top2a	5217
NM 146472	Olfr1384	1034	NM 001131020	Gfap	2600
NM 147118	Olfr635	966	NM 001001309	Itga8	5782
NM 001024135	Kbtbd7	4529	NM 053202	Foxp1	7177
NM 134208	Vmn1r78	942	NM 201638	Mettl14	2625
NM 146287	Olfr114	939	NM 023279	Tubb3	1758
NM 146289	Olfr113	939	NM 146095	Rorb	8755
NM 146296	Olfr437	933	NM 001039167	Rbfox3	4545
NM 146300	Olfr827	969	NM 011281	Rorc	2503
NM 001105557	Zfp938	2031	NM 146146	Lepr	4126
NM 147035	Olfr711	1359	NM 007378	Abca4	7268
NM 146559	Olfr868	1132	NM 001199177	Opa1	6002
NM 134201	Vmn1r236	1121	NM 194051	Rtn4	5558
NM 147039	Olfr1414	1189	NM 010566	Inpp5d	4938
NM 001270542	Scgb1b7	421	NM 031170	Krt8	1805
NM 001270543	Scgb1b20	421	NM 009029	Rb1	4642
NM 146560	Olfr872	1124	NM 001204201	Spp1	1475
NM 001033783	Gm5615	2342	NM 001110497	Tmem87a	2921
NM 146578	Olfr1033	3744	NM 010025	Dcx	8982
NM 009493	Vmn2r42	3623	NM 198438	Ssbp3	3130
NM 007535	Bcl2a1c	444	NM 008089	Gata1	1902
NM 146698	Olfr1443	4361	NM 001163574	Tjp1	6891
NM 177622	Zfp595	3909	NM 001043354	Rorb	9289
NM 201643	Ugt1a5	3208	NM 134024	Tubg1	1609
NM 146520	Olfr536	3915	NM 001159717	2-Sep	3219
NM 146809	Olfr1426	1727	NM 175367	Ston2	9896
NM 020563	Scgb1b2	425	NM 023117	Cdc25b	3106
NM 009485	Vmn2r123	3045	NM 001252192	Eya1	4238
NM 001277181	Gm1966	8759	NM 001256082	Myo7a	7214
NM 146782	Olfr921	1520	NM 001164274	Mga	13304
NM 145707	Obox3	1603	NM 015775	Tmprss2	3175
NM 177899	Zfp866	5780	NM 001289921	Rorb	8808
NM 146288	Olfr122	1030	NM 008002	Fgf10	4572
NM 001039965	Zfp869	3194	NM 028116	Pygo1	1745
NM 001039967	Zfp869	3302	NM 001204233	Spp1	1517
NM 001039241	-	2819	NM 013636	Ppp1cc	2379
NM 016686	Vezf1	4597	NM 001110193	Inpp5d	4755
NM_207674	Olfr1082	990	NM_145561	Tmprss11d	2046
NM 001099329	Scgb1b24	400	NM 001166402	Tnfaip3	4352
NM 134199	Vmn1r235	1781	NM 053069	Atg5	2352

NM 146629	Olfr121	1253
NM 001160131	Gm20854	1005
NM 001160137	Gm20809	1001
NM 146494	Olfr722	1655
NM 001081662	C86695	1887
NM 144934	Mbd3l2	2155
NM 146989	Olfr1496	1067
NM 001037922	Cks1brt	2397
NM 019485	Olfr70	2435
NM 178875	Zfp935	2500
NM 001256066	Scgb1b29	404
NM 001136496	Zfp935	2439
NM_001085511	4932429P05Rik	3093
NM 001256073	Scgb1b3	419
NM 001033253	Plekhg1	7190
NM_001009544	4921501E09Rik	3916
NM 001199270	Aldoart1	2052
NM 001160386	Dnah7b	12318
NM 001126322	Gm11595	1200
NM 053138	Pcdhb13	3693
NM 146309	Olfr1337	951
NM 001164739	Fam47c	1648
NM 001100616	Vmn2r121	3062
NM_001033807	4930567H17Rik	840
NM 198961	Vmn2r43	2855
NM 001033197	AU015228	2982
NM 172844	Fmo9	3065
NM 001160141	Gm20865	866
NM 009487	Vmn2r89	2962
NM 009486	Vmn2r89	2737
NM 001177586	Esp31	1311
NM 001177585	Esp34	1413
NM 001045553	Zfp868	2449
NM 172754	Zfp868	2464
NM_001292066	Zfp708	2436
NM 001103182	Lin9	3178
NM 001177652	Gm5886	647
NM 001293057	Gm21379	1958
NM 001293229	Zfp799	717
NM 177359	Zfp799	6025
NM 001293301	Clasp1	7651
NM 001293300	Clasp1	7951
NM 001293300	Clasp1	7951

NM 001083316	Pdgfra	6570
NM 178143	Prkaa2	8201
NM 001271585	Dnajc5	4405
NM 194053	Rtn4	3804
NM 001037859	Csf1r	3875
NM 023043	Prnd	3178
NM 010043	Des	3065
NM 010856	Myh6	6015
NM 181586	Sirt6	1703
NM 008192	Gucy2e	8331
NM 001256083	Myo7a	7196
NM 021433	Stx6	2459
NM_001128599	Opn4	2259
NM 008584	Meox2	2348
NM 001278520	Prnd	1919
NM_008029	Flt4	5853
NM 001163522	Emcn	1476
NM 173368	Chd6	10573
NM 001110351	Sin3a	4998
NM 021284	Kras	4670
NM 139294	Braf	9728
NM 177129	Cntn2	9535
NM 001126338	Prnd	1940
NM_011263	Rest	4266
NM 001278257	Prnd	1970
NM 011611	Cd40	1683
NM 001170959	Pde6c	2892
NM 028136	Dhx36	4975
NM 194052	Rtn4	3747
NM 001110224	Dcx	8997
NM 013683	Tap1	2950
NM 001206385	Mavs	2871
NM 173734	Tmem87a	2927
NM 001039168	Rbfox3	4401
NM_001199213	Serpinb12	1619
NM 009693	Apob	13931
NM 001039154	Cdh8	3822
NM 001159718	2-Sep	3192
NM 170703	Cd40	1621
NM 030679	Myh1	6058
NM 008937	Prox1	4148
NM 001204203	Spp1	1423
NM 001177810	Rapgef3	3825
NM 011159	Prkdc	12674
NM 009263	Spp1	1428
NM 001110192	Inpp5d	4935
NM 001161730	Tap1	2866
NM 001110222	Dcx	9078
NM 011949	Mapk1	5099
NM 001253717	Atg7	3925
NM 001013367	Prkaa1	4655
NM 010164	Eya1	4354
NM 019584	Becn1	2031
NM 011378	Sin3a	5229
NM 023672	Ssbp3	3211
NM 001177867	Sgol2	4337
NM 033217	Ngfr	3409
NM 028835	Atg7	3774

NM 029216	Chd5	9375
NM 001272041	Acta1	1571
NM 001122850	Pard3	4178
NM 001110223	Dcx	9000
NM 001081376	Chd5	9486
NM 016775	Dnajc5	4325
NM 029885	Gabpb2	8703
NM 207222	Lmo3	2101
NM 001197321	Foxp1	7042
NM 144850	Rapgef3	3801
NM 010806	Mllt4	7401
NM 053247	Lyve1	2607
NM 207243	Muc19	22806
NM 001177811	Rapgef3	3774
NM 001163493	Stard13	5638
NM 146258	Stard13	5581
NM 001290994	Usp29	7540
NM 023258	Pycard	2135
NM 001164112	Nfatc1	4604
NM 009986	Cux1	12912
NM 001290636	Dock7	7139
NM 001289830	Nanog	2020
NM 001123382	Il1r1	4808
NM 001113569	Stxbp1	4005
NM 007601	Capn3	3167
NM 001136086	Dpysl3	5252
NM 011586	Myo18a	7360
NM 001291432	Dido1	4717
NM 011602	Tln1	8560
NM 001290710	Ebf1	4999
NM 001109761	Capn3	2624
NM 010804	Mllt10	4940
NM 001291004	H6pd	4678
NM 001164111	Nfatc1	4315
NM 001256001	Cacna1c	13643
NM 001168514	Mapk14	3605
NM 027089	Eqtn	1287
NM 198429	Nfatc1	4607
NM 011710	Wars	1842
NM 009204	Slc2a4	2822
NM 001287176	Alpl	2672
NM 008929	Dnajc3	5190
NM 001111023	Runx1	7138
NM 001033324	Zbtb16	5114
NM 001290709	Ebf1	5206
NM 146194	Picalm	4174
NM 001252523	Picalm	4009
NM 013822	Jag1	5493
NM 001252524	Picalm	3985
NM 001113389	Dyrk1a	5776
NM 010227	Flna	8347
NM 001168508	Mapk14	3560
NM 001291433	Dido1	7311
NM 009821	Runx1	6946
NM 001290623	Eqtn	1164
NM 010889	Neb	22489
NM 001291068	Polr2a	6736
NM 026082	Dock7	7049
NM 011042	Pcbp2	2829
NM 001255997	Cacna1c	13340
NM 010332	Ednra	3643
NM 001159535	Cacna1c	13586

NM 011842	Mta2	3149
NM 001079908	Fgfr1	5035
NM 010747	Lyn	3393
NM 010789	Meis1	3346
NM 198602	Cux1	3074
NM 009781	Cacna1c	13340
NM 001204134	C1qtnf3	2544
NM 001289831	Nanog	2004
NM 010141	Epha7	6750
NM 011805	Dido1	4769
NM 008514	Lrp6	9368
NM 001168513	Mapk14	3161
NM 001291238	Cux1	3080
NM 011393	Slc1a2	2127
NM 020009	Mtor	8612
NM 001290464	Adamts13	3474
NM 007901	S1pr1	3029
NM 008413	Jak2	5055
NM 001252522	Picalm	4024
NM 199239	Sema6d	5627
NM 133990	Il13ra1	3686
NM 030150	Dhx58	2427
NM 001290997	Sema6d	6178
NM 013492	Clu	1808
NM 001290335	Cacna1c	12656
NM 009127	Scd1	4844
NM 001164110	Nfatc1	4896
NM 172537	Sema6d	6335
NM 021323	Usp29	7476
NM 009295	Stxbp1	3874
NM 008714	Notch1	9497
NM 176933	Dusp4	2427
NM 001159533	Cacna1c	13592
NM 016756	Cdk2	2288
NM 001035226	Xpo1	4906
NM 001111021	Runx1	5803
NM 009468	Dpysl3	5433
NM 007897	Ebf1	5203
NM 001048177	Jak2	4972
NM 001291240	Cux1	3369
NM 013646	Rora	10878
NM 175642	Bai3	5522
NM 007987	Fas	1486
NM 001164663	Soga1	13232
NM 001291215	Myo18a	6417
NM 001289916	Rora	10869
NM 001111022	Runx1	5611
NM 183417	Cdk2	2432
NM 010206	Fgfr1	5025
NM 199241	Sema6d	5756
NM 001164314	Wars	2831
NM 145840	Rgs9bp	6604
NM 001289828	Nanog	2220
NM 001040400	Tet2	9199
NM 007614	Ctnnb1	3640
NM 007868	Dmd	13857
NM 001193271	Meis1	3441
NM 030888	C1qtnf3	2350
NM 007431	Alpl	2524
NM 175551	Dido1	8476
NM 010414	Htt	13237
NM 001177799	Capn3	2891

NM 001290760	Tbc1d9b	5271
NM 001252560	Mllt10	5065
NM 001167923	Col6a5	9297
NM 008689	Nfkb1	4128
NM 001159534	Cacna1c	13592
NM 008512	Lrp1	14907
NM 199240	Sema6d	5699
NM 007453	Prdx6	2334
NM 001077514	Slc1a2	11571
NM 011808	Ets1	5060
NM 001079909	Fgfr1	4758
NM 001290421	Flna	8369
NM 010508	Ifnar1	7025
NM 007434	Akt2	2957
NM 021524	Nampt	4547
NM 001290759	Tbc1d9b	5268
NM 173371	H6pd	4746
NM 001291214	Myo18a	6351
NM 001291000	Sema6d	6509
NM 031178	Tlr9	3471
NM 001290434	Epha7	6738
NM 001290463	Adamts13	4496
NM 001164488	Wars	2764
NM 001077515	Slc1a2	10998
NM 134014	Xpo1	5144
NM 001252520	Picalm	4159
NM 001103165	Pcbp2	2868
NM 008362	Il1r1	4739
NM 008509	Lpl	4049
NM 001111096	Lyn	3456
NM 001081304	Atf6	7463
NM 007890	Dyrk1a	6035
NM 001291234	Cux1	13146
NM 001252521	Picalm	4150
NM 001287172	Alpl	2549
NM 001255998	Cacna1c	13415
NM 011951	Mapk14	3560
NM 001290465	Adamts13	3447
NM 199238	Sema6d	6296
NM 001103166	Pcbp2	2775
NM 001291239	Cux1	3363
NM 001256002	Cacna1c	13841
NM 001255999	Cacna1c	13340
NM 001165902	Ctnnb1	3440
NM 009744	Bcl6	3326
NM 001290711	Ebf1	5322
NM 177852	Dido1	7259
NM 001256000	Cacna1c	13634
NM 001110208	Akt2	3199
NM 028016	Nanog	2223
NM 001291233	Cux1	13507
NM 001174073	Pcbp2	2724
NM 001252561	Mllt10	5116
NM 001038642	Ets1	4799
NM 001291455	Dpysl3	5502
NM 001104543	Vmn2r94	2529
NM 053146	Pcdhb21	2712
NM 053141	Pcdhb16	5278
NM 024170	Cxx1a	1276
NM 001105074	Vmn2r44	2586
NM 001199062	Gm4461	1091
NM 001105070	Vmn2r38	2586

NM 001205282	Gm14496	2550
NM 001105075	Vmn2r45	2559
NM 001105071	Vmn2r39	2586
NM 001105178	Vmn2r50	2586
NM 001184981	Sult2a7	897
NM 001105061	Gm9268	2565
NM 001105072	Vmn2r40	2559
NM 001105066	Vmn2r34	2559
NM 001105073	Vmn2r41	2559
NM 001104551	Vmn2r99	2571
NM 001126325	Gm13088	1819
NM 147083	Olfr622	954
NM 146836	Olfr1132	927
NM 134222	Vmn1r218	897
NM 146860	Olfr161	942
NM 134227	Vmn1r87	888
NM 146625	Olfr355	933
NM 146371	Olfr1450	978
NM 001011794	Olfr1322	933
NM 146637	Olfr1141	936
NM 146638	Olfr1151	927
NM 146639	Olfr1138	936
NM 001011798	Olfr884	930
NM 146394	Olfr1278	942
NM 146653	Olfr435	942
NM 001011811	Olfr487	945
NM 001011813	Olfr93	939
NM 146911	Olfr1377	924
NM 001011842	Olfr1474	945
NM 001011857	Olfr685	951
NM 146458	Olfr1199	933
NM 001011869	Olfr452	954
NM 001011534	Olfr988	930
NM 146995	Olfr202	924
NM 147005	Olfr395	939
NM 206823	Olfr153	924
NM 146769	Olfr1110	939
NM 147064	Olfr449	936
NM 146301	Olfr1475	945
NM 146658	Olfr1131	930
NM 146690	Olfr1472	945
NM 146766	Olfr1109	939
NM 001039128	Tas2r122	930
NM 146770	Olfr259	939
NM 146917	Olfr1179	924
NM 146786	Olfr914	951
NM 146819	Olfr466	927
NM 207132	Olfr1471	945
NM 146660	Olfr1135	933
NM 053135	Pcdhb10	2842
NM 013619	Olfr67	1242
NM 146718	Olfr430	954
NM 146994	Olfr201	927
NM 146673	Olfr823	948
NM 146584	Olfr1026	924
NM 001038995	Gm4787	2511
NM 146456	Olfr92	939
NM 001111318	Cldn24	663
NM 053137	Pcdhb12	3034
NM 134436	Vmn1r27	912
NM 001101533	Vmn1r20	912
NM 146805	Olfr907	933

NM 010998	Olfr55	948
NM 153093	AF366264	2881
NM 001166835	Vmn1r79	921
NM 001166837	Vmn1r114	966
NM 001166838	Vmn1r126	966
NM 001166732	Vmn1r60	903
NM 001166741	Vmn1r121	945
NM 001167160	Gm10665	966
NM 001167573	Gm10666	966
NM 001033878	Vmn2r66	2556
NM 001104550	Vmn2r98	2574
NM 001104541	Vmn2r92	2583
NM 001104570	Vmn2r108	2568
NM 001100184	Cyp2t4	1512
NM 001103365	Vmn2r86	2547
NM 001104565	Vmn2r103	2571
NM 001011517	Olfr1193	957
NM 001164286	Gm5415	3748
NM 001105186	Vmn2r73	2556
NM_001243118	2010315B03Rik	2688
NM 001105152	Vmn2r48	2568
NM 001104624	Vmn2r13	2496
NM 001104632	Vmn2r19	2565
NM 001104542	Vmn2r93	2574
NM 001103366	Vmn2r87	2547
NM 001102579	Vmn2r67	2556
NM 207547	Vmn1r93	966
NM 001104539	Vmn2r90	2592
NM 001104566	Vmn2r104	2583
NM 001104625	Vmn2r14	2547
NM 001105055	Vmn2r58	2589
NM 001081449	Vmn2r54	2421
NM 001102578	Vmn2r75	2562
NM 001105182	Vmn2r69	2553
NM 001104567	Vmn2r105	2583
NM 001104540	Vmn2r91	2571
NM 001105179	Vmn2r51	2559
NM 001104537	Vmn2r83	2595
NM 001102602	Vmn2r85	2553
NM 001105056	Vmn2r59	2598
NM 146844	Olfr1107	1109
NM 001105183	Vmn2r70	2565
NM 001102580	Vmn2r76	2562
NM 001104568	Vmn2r106	2589
NM 001105188	Vmn2r77	2565
NM 001104572	Vmn2r110	2577
NM 001105156	Vmn2r49	2559
NM 001102581	Vmn2r95	2538
NM 009490	Vmn2r30	2559
NM 001104626	Vmn2r15	2568
NM 001104562	Vmn2r100	2553
NM 001105184	Vmn2r71	2568
NM 134173	Vmn1r24	891
NM 001105076	Vmn2r46	2430
NM 001113468	Vmn2r29	2559
NM 001104569	Vmn2r107	2586
NM 001104582	Vmn2r118	2562
NM 001104627	Vmn2r16	2553
NM 001104591	Vmn2r120	2571
NM 001105057	Vmn2r60	2562
NM 146367	Olfr976	2813

NM 001104563	Vmn2r101	2574
NM 001105065	Vmn2r33	2586
NM 001011810	Olfr485	954
NM 001104622	Vmn2r11	2586
NM 001104549	Vmn2r97	2586
NM 001105067	Vmn2r35	2586
NM 001103368	Vmn2r80	2592
NM 001105190	Vmn2r79	2556
NM 001104628	Vmn2r17	2550
NM 001105058	Vmn2r61	2598
NM_001243117	2010315B03Rik	2700
NM 146753	Olfr1195	927
NM 001105151	Vmn2r47	2559
NM 001104564	Vmn2r102	2574
NM 001105068	Vmn2r36	2559
NM 001195672	Ccdc170	2151
NM 001162903	Ccdc71l	4155
NM 001167539	Vmn1r238	921
NM 020015	Magea1	1272
NM 001167534	Vmn1r2	921
NM 001099312	Gm11569	869
NM 147028	Olfr1124	957
NM 001105063	Vmn2r32	2559
NM 001105191	Vmn2r52	2559
NM 001105181	Vmn2r68	2556
NM 001105185	Vmn2r72	2571
NM 173412	Cypt4	648
NM_001277487	LOC101055863	1240
NM_001277512	LOC100502896	1240
NM_001277531	LOC100862015	1240
NM 001277575	Gm11236	1240
NM 147088	Olfr569	945
NM 001167535	Vmn1r3	921
NM 001025240	Gm5346	2211
NM 053144	Pcdhb19	4714
NM 001199956	Gm9839	1437
NM 020017	Magea3	1107
NM 177256	Prdx6b	2889
NM 001270685	Gm21637	778
NM_146873	Olfr911-ps1	940
NM 146872	Olfr908	933
NM 001034881	Gm5464	2156
NM 146369	Olfr434	966
NM 146370	Olfr47	966
NM 001099313	Gm11554	777
NM 001005418	-	336
NM 001011795	Olfr1275	939
NM 146304	Olfr1340	948
NM 053127	Pcdhb2	2789
NM 053128	Pcdhb3	3787
NM 001204913	Gm20172	1551
NM 146817	Olfr1156	1076
NM 053129	Pcdhb4	3719
NM 147105	Olfr978	936
NM 146642	Olfr1140	1020
NM 001177504	Gm6588	2313
NM 001166433	AU022751	2203

NM_027087	Krtap4-13	819
NM_001163141	Krtap24-1	1571
NM 053134	Pcdhb9	3055
NM 146674	Olfr824	948
NM 001011831	Olfr1500	936
NM 146852	Olfr1339	948
NM 147000	Olfr173	1164
NM 207175	Olfr239	948
NM 146950	Olfr341	942
NM 207631	Olfr1321	960
NM_178243	5830403L16Rik	1659
NM 053147	Pcdhb22	3067
NM 146881	Olfr1404	1034
NM 001011871	Olfr506	945
NM 001011753	Olfr115	995
NM 146290	Olfr125	1118
NM 146988	Olfr447	933
NM 146477	Olfr90	1046
NM 182714	Olfr91	939
NM 146741	Olfr1497	945
NM 207550	Olfr199	927
NM 146488	Olfr137	939
NM 198621	Cct8l1	1967
NM 146771	Olfr1176	948
NM 025741	Trpd52l3	2231
NM 146918	Olfr1180	1063
NM 147056	Olfr646	939
NM 146287	Olfr114	939
NM 010115	Egfbp2	858
NM 146315	Olfr62	948
NM 001001809	Olfr218	942
NM 146303	Olfr1449	945
NM 199062	Zfp781	4089
NM 001105557	Zfp938	2031
NM 028705	Herc3	4742
NM 146578	Olfr1033	3744
NM 001011523	Olfr913	1275
NM 009493	Vmn2r42	3623
NM 001039239	Zfp808	3114
NM 001003670	Gm5414	1659
NM_001243119	2010315B03Rik	2625
NM 001166062	Gm16381	689
NM 146520	Olfr536	3915
NM 146809	Olfr1426	1727
NM 009485	Vmn2r123	3045
NM 001033767	Gm4951	2648
NM 177899	Zfp866	5780
NM 001018063	Cxx1b	1246
NM 001039241	-	2819
NM 016686	Vezf1	4597
NM 053131	Pcdhb6	2319
NM 009620	Adam4	2330
NM 001033789	Gm5820	2187
NM 146898	Olfr1213	1297
NM_029308	1700010B08Rik	896
NM 053172	Mepe	1682
NM 019485	Olfr70	2435

NM 001104578	Vmn2r113	2571
NM 020280	Magea4	948
NM_001009544	4921501E09Rik	3916
NM 001277919	Gm11780	1685
NM 001160386	Dnah7b	12318
NM 001085553	Gm10921	1960
NM 001126322	Gm11595	1200
NM 020513	Olfr1508	1865
NM 053140	Pcdhb15	2795
NM 053138	Pcdhb13	3693
NM 001159275	Slc25a2	1346
NM 001256481	Gm11237	1237
NM 009489	Vmn2r37	3102
NM_001276278	1110025L11Rik	594
NM 001100616	Vmn2r121	3062
NM_177001	9130023H24Rik	3581
NM_054100	2310034C09Rik	943
NM 001033541	Gm5127	1809
NM 053150	-	1147
NM 198961	Vmn2r43	2855
NM 172844	Fmo9	3065
NM 001039219	Gm6086	4068
NM 001039944	Cypt10	540
NM 001039942	Cypt9	533
NM 001085534	Gm5938	797
NM_029106	Spin2-ps1	778
NM 009487	Vmn2r89	2962
NM 009486	Vmn2r89	2737
NM 001177587	Esp36	1117
NM 001287179	Kif14	8338
NM 001293301	Clasp1	7651
NM 001293300	Clasp1	7951
